# Immunoinformatics and molecular dynamics approaches: Next generation vaccine design against West Nile virus

**DOI:** 10.1371/journal.pone.0253393

**Published:** 2021-06-17

**Authors:** Md Tahsin Khan, Rahatul Islam, Tarhima Jahan Jerin, Araf Mahmud, Sahara Khatun, Ahasanul Kobir, Md Nahidul Islam, Arzuba Akter, Shakhinur Islam Mondal

**Affiliations:** 1 Department of Genetic Engineering and Biotechnology, Shahjalal University of Science and Technology, Sylhet, Bangladesh; 2 Department of Biotechnology and Genetic Engineering, Mawlana Bhashani Science and Technology University, Tangail, Bangladesh; 3 UPMC Hillman Cancer Center, University of Pittsburgh, Pittsburgh, Pennsylvania, United States of America; 4 Department of Biochemistry, School of Natural Sciences, National University of Ireland Galway, Galway, Ireland; 5 Department of Biochemistry and Molecular Biology, Shahjalal University of Science and Technology, Sylhet, Bangladesh; University of Alabama at Birmingham, UNITED STATES

## Abstract

West Nile Virus (WNV) is a life threatening flavivirus that causes significant morbidity and mortality worldwide. No preventive therapeutics including vaccines against WNV are available for human use. In this study, immunoinformatics approach was performed to design a multi epitope-based subunit vaccine against this deadly pathogen. Human (HLA) and Mice (H-2) allele specific potential T-cell and B-cell epitopes were shortlisted through a stringent procedure. Molecular docking showed selected epitopes that have stronger binding affinity with human TLR-4. Molecular dynamics simulation confirmed the stable nature of the docked complex. Furthermore, *in silico* cloning analysis ensures efficient expression of desired gene in the microbial system. Interestingly, previous studies showed that two of our selected epitopes have strong immune response against WNV. Therefore, selected epitopes could be strong vaccine candidates to prevent WNV infections in human. However, further *in vitro* and *in vivo* investigations could be strengthening the validation of the vaccine candidate against WNV.

## Introduction

West Nile virus (WNV) is a single-stranded RNA virus (family Flaviviridae; genus *Flavivirus*) [1,2] and its infection can cause physical, neurologic, and cognitive disabilities [3]. Generally, WNV infection is asymptomatic. A common febrile illness occurs in ≈20% of the population and a small portion (<1%) develop severe West Nile neuroinvasive disease (WNND); characterized by encephalitis, meningitis, and/or acute flaccid paralysis [4,5]. The virus was initially identified in Uganda but now prevalent almost in all continents except Antarctica [6,7]. Since it was first detected in New York City in 1999, it causes thousands of cases each year in the United States and around 7 million total human infections were reported in the continental US [1,8]. Between 1999 and 2019, Centers for Disease Control and Prevention (CDC) has reported more than 48,000 affected cases and over 2,300 deaths in US due to WNV infection [9]. WNV infection, mainly caused by WNV lineage 1 strains, has been an endemic in Eastern Europe such as Romania and Russia for over 50 years. Between 2004 to 2010, mammalian infections by WNV lineage 2 strains were reported in Hungary, Austria, and Russia. Other countries such as Greece, Macedonia, Romania, Serbia, Turkey, and Ukraine reported WNV infections in mammal after 2010 [10]. Since last few decades, emergence of WNV infections had been also reported in Southeast Asia, especially in several districts of India such as Assam [11], Kerala [12], West Bengal [13], Tamil Nadu [14], Madhya Pradesh [15], Andhra Pradesh [16], and Pune [17]. The economic burden of hospitalized WNV patients in US is $56 million, and short- or long- term costs can even exceed $700,000 per patient [18,19]. Considering the overall clinical and economic impacts caused by WNV, currently it takes more concern and considered one of the most vital zoonotic diseases to the US population.

The genome of WNV virus is approximately 11kb that encodes three structural proteins: Capsid protein (C), Pre-membrane/membrane protein (prM/M), and envelope protein (E). WNV virus also encodes seven non-structural proteins (NS1, NS2A, NS2B, NS3, NS4A, NS4B and NS5); however their role yet to be understood [20]. The heterodimer forms of the prM and E protein are the two main immunogens of this virus [21]. The E protein is the major surface component for receptor binding and viral entry into host cells through membrane fusion [22]. Capsid protein is involved in viral RNA replication and packaging [20,23]. Based on the molecular information of WNV and research advancements, so far, a total 23 versatile vaccines preparations have been developed that include 4 DNA, 4 recombinant vectors, 11 inactivated/killed, and 4 live/attenuated (http://www.violinet.org/). Some of those vaccine candidates are under pre-clinical studies and a very few are under clinical trials; however, none of the WNV vaccines has yet been approved to be used in human host. Currently, only the YFV-based vaccine (ChimeriVax-WN02) has been evaluated in Phase II trial [24]. Other human vaccines are in phase I clinical trial: WN/DEN4D30 (recombinant vaccine), VRC-WNVDNA017-00-VP (DNA vaccine), and WN-80E (Recombinant subunit vaccine) [25–27]. None of the studies yet developed a potential vaccine where all the structural and non-structural viral proteins can be considered as antigens. In fact, only one or two proteins of the virus, mostly PrM/E, were used as antigens for formulating these vaccines. Also, in case of live attenuated virus (LAV) strategy, there is associated risk of reversion to the original pathogenic form of the virus.

The growing advances in the field of bioinformatics open a new era to design next generation vaccines through the knowledge of immunoinformatics; the detailed protocol and immune responses have been widely reviewed in published literatures [28–31]. In recent years, reverse vaccinology approach has been utilized to design epitope based multi-subunit vaccines against different pathogens and cancers [32–41]. Sufficient cellular and humoral immune responses triggered by multi-epitope based vaccine against various pathogens including Epstein–Barr virus, Crimean-Congo Hemorrhagic Fever Virus and Chlamydia trachomatis [42–45]. These vaccines works successfully both *in vitro* and *in vivo* murine model and thereby reducing time, costs and repetition of error trails of traditional vaccine development. In this study, by utilizing immunoinformatics and reverse vaccinology approaches, we design a candidate multi-epitope chimeric vaccine against the WNV virus that is likely to be safe and immunogenic against the WNV infection.

## Methods

### Retrieval of protein sequences and physiochemical properties analysis

In this study, we adopted computational methods to predict the effective vaccine candidates against WNV Virus where UniProt ID- Q9Q6P4 was considered as the reference strain. All the proteins of this strain including structural (Capsid protein, prM, small envelope protein M and envelope protein) and non-structural (NS1, NS2B, NS2A, NS4A, NS3, NS4B, and NS5) viral proteins were retrieved in FASTA format from UniProt (Universal Protein Resource) database (http://www.uniprot.org/uniprot). Further ProtParam tool at Expasy server (http://expasy.org/cgi-bin/protpraram) was used to characterize the functional physiochemical parameters of all the proteins of reference strain [46].

### Prediction of linear B cell epitope

Linear B cell epitopes were predicted by combining several B cell epitope prediction server including ABCpred (http://crdd.osdd.net/raghava/abcpred/) [47], Bepipred (http://tools.iedb.org/bcell/) [48], Emini Surface Accessibility Prediction tool of IEDB (Immune Epitope Database) (http://tools.iedb.org/bcell/) [49], Karplus and Schulz flexibility tool (http://tools.iedb.org/bcell/) [50] and Parker Hydrophilicity Prediction method of IEDB (http://tools.iedb.org/bcell/) [51]. Combination of all methods increases the accuracy of B cell epitope prediction to a greater extent.

ABCpred is a recurrent neural network (ANN) algorithm for predicting B-cell epitopes (random peptides) of maximum length of 20 residues. The server was trained on 700 B-cell epitopes and 700 non B-cell epitopes for predicting B-cell lymphomas. The predicted accuracy achieved by this server is 65.93% using recurrent neural network. We set the default threshold of 0.51 in ABCpred server for BCL prediction. The BepiPred-2.0 server, a Random Forest algorithm predicts B-cell epitopes by comparing a set of trained epitopes and non-epitope from crystal structures. Epitopes with threshold value >0.5 are considered and colored in yellow on the graph. Emini Surface Accessibility tool calculates the surface accessible epitopes by using a formula Sn = (n+4+i) (0.37)-6 where n is the value of fractional surface probability, Sn refers the surface probability and values of i varies from 1 to 6. Peptide sequence with Sn greater than 1.0 indicates the probability of being as surface accessible epitopes. The output of Karplus and Schulz flexibility tool provides seven amino acids window lengths of epitope based on three scales for flexibility calculation. Parker Hydrophilicity Prediction tool predicts the hydrophilicity of peptides by calculating the retention times of peptides during high-performance liquid chromatography (HPLC) on a reversed-phase column. Hydrophilic epitope regions were predicted by a window of seven residues score. Again, predicted B cell epitopes were also screened through VaxiJen, AllerTOP, ToxinPred and TMHMM webserver for final candidate selection.

### Prediction of CD8+ T cell (CTL) epitope

T cell epitopes were identified by using a non-linear artificial neural networks-based server, NetCTL 1.2 server (http://www.cbs.dtu.dk/services/NetCTL/) [52] where HLA class I alleles are sub-grouped into 12 super-families (A1, A2, A3, A24, A26, B7, B8, B27, B39, B44, B58, B62). All of the amino acid sequences of all WNV viral proteins were screened against each of the HLA class I superfamily (a total of 8 × 12 = 96 queries). The threshold values used for WNV CTL prediction was 0.75. All of the epitopes beyond the threshold value were then selected for further analysis.

VaxiJen server (http://www.ddg-pharmfac.net/vaxijen/) was used to analyze the antigenicity of the proteins [53]. Epitopes that cannot meet the threshold level of the VaxiJen server, which is 0.4, were discarded. AllerTOP was also employed to determine the allergenicity of the selected T-cell epitopes [54]. Additionally, ToxinPred webserver was used to identify the toxicity of the epitopes [55]. Immunogenicity of the predicted epitopes was examined by IEDB MHC I immunogenicity prediction tool (http://tools.iedb.org/immunogenicity/) [56]. Finally transmembrane topology of the predicted epitopes was determined by using the TMHMM server (http://www.cbs.dtu.dk/services/TMHMM/) [57]. After analyzing the entire parameters NetMHC 4.0 server (http://www.cbs.dtu.dk/services/NetMHC/) was used for cross checking with the selected epitopes for mouse allele (H-2) binding [58,59]. Epitopes showing positive results were further cross-checked with IEDB class I immunogenicity server for Human HLA class-I binding. Finally, most HLA covering epitopes were selected as final epitopes and subjected to population coverage analysis.

### Prediction of CD4+ T cell (HTL) epitope

IEDB MHC II binding database (http://tools.iedb.org/mhcii/) [60] was used for prediction of Mouse H-2-I allele binding epitope from all the 10 proteins of WNV. IEDB recommended method was selected as the prediction method and a window length of 15 mer was selected for CD4+ T cell (HTL) epitope prediction at IEDB MHC II binding interface.

Further these epitopes were screened through VaxiJen, AllerTOP, ToxinPred and TMHMM webserver. After cross checking with these servers final selected epitopes were then screened through IFNepitope webserver (http://crdd.osdd.net/raghava/ifnepitope/) [61]. This server is used for the prediction of IFN-γ inducing epitope in a set of peptide library. The amino acid sequences of all the selected epitopes from previous screening were submitted as query for IFN-γ inducing peptide prediction. Finally, epitopes showing positive results were further cross-checked with IEDB class II immunogenicity server for Human HLA class-II binding. As like as CTL screening, most HLA class-II covering epitopes were selected as final HTL epitopes and subjected to population coverage analysis.

### Conservancy analysis

In case of *in silico* vaccinology approach, conservancy analysis is used to determine the degree of epitope distribution in the homologous protein set. We used the epitope conservancy analysis tool (http://tools.iedb.org/conservancy/) at the IEDB (Immune Epitope Database) for the analysis of conservancy pattern of the desired epitopes. The sequence identity threshold was set at 100%, and a set of 100 homologous sequences of the selected 10 antigenic protein were retrieved from the NCBI (National Center for Biotechnology Information) database with BLASTp tool against the WNV Virus, for using as homologous proteins set in the conservancy tool [62].

However, in South East Asia, Europe and USA closely related flaviviruses are circulating. These viruses does not protect each other; there can be a possibility of cross protective immunity as all these viruses shares the same family. Furthermore, as a rule in flaviviruses, virus neutralizing antibodies is necessary. That’s why the conserved B cell epitopes of WNV virus were then again ran in the conservancy analysis tool to look for homology in the Dengue Virus (Type-1,2,3 and 4) and Japanese encephalitis viruses reference polyprotein to check if any of the B cell epitope is conserved and similar in those polyproteins or not. If our predicted epitopes are matched in these polyproteins, there may be a cross reaction possibility can be occurred.

### Population coverage analysis

For effective vaccination, a vaccine molecule must provide a broad-spectrum protection against different populations around the world. However extreme polymorphic behavior of MHC molecules (Near around 6000) causes different MHC derived pool/frequencies in individuals of different ethnicities/country. Thus, selecting multiple peptides with different HLA binding capacities can increase coverage of population around the world. To address this issue, IEDB population coverage tool (http://tools.iedb.org/population/) [63] was utilized to calculate the individual fractions response to a given set of epitopes based on HLA genotypic frequencies. However, the program provides three different calculation options for population coverage analysis: (1) class I separate, (2) class II separate, and (3) class I and class II combined. We used all of these options for predicting the population coverage of the proposed CTL and HTL epitopes found from preceding analysis.

To do that, all parameters in IEDB population coverage interface kept at default (1), the projected population coverage (2), the average number of HLA combinations recognized by the populations (3), and minimum number of HLA combinations recognized by 90% of the population (PC_90_) were calculated. [63]. Additionally, the corresponding MHC allele of the CTL and HTL were derived from IEDB MHC-I and MHC II prediction server.

### Vaccine construction

For the construction of the final vaccine protein, predicted B cell epitopes and T cell epitopes were joined by suitable fusion protein linkers. Then Universal Pan HLA DR sequence (PADRE) sequence was attached to overcome the problems caused by highly polymorphic HLA class-2 alleles in the final vaccine construct [64]. C-terminal invasin sequence of *Yersinia* was also added at the C-terminal end of the construct. Finally, an Adjuvant peptide was also incorporated in the final vaccine protein. Adjuvants can interact with toll like receptors (TLRs) to stimulate robust immune-reaction [65]. Beta-defensin-3 was used as a toll like receptor agonist in the final vaccine construct. The linkers used in the final vaccine construct were EAAAK, GGGS, GPGP, KK and EGGE. Each of the different linkers has different role. However the EAAAK linker can act as rigid linkers in the final vaccine construct whereas GGGS, GPGP, KK and EGGE provide flexibility to fused the vaccine candidates consequently [66]. Thus utilized linkers can ensure effective separation of individual epitopes [67].

### Prediction of physicochemical and immunogenic properties

ProtParam, a tool available on ExPASy server (http://expasy.org/cgi-bin/protpraram) [46] was used to characterize the functional physiochemical parameters of our vaccine constructs. Various physicochemical properties of the vaccine proteins were resolved including isoelectric pH, aliphatic index, molecular weight, GRAVY values, instability index, hydropathicity, and estimated half-life. Physiochemical parameters are analyzed by calculating the pKa values of each amino acid present in the protein. Further Protein-sol server was used to calculate the solubility of the two vaccine proteins in terms of surface distribution of charge, hydrophobicity and stability [68]. This server can detect those sequence features which has the strongest impact on protein solubility. The experimental dataset (PopAvrSol) in Protein-sol server had a population average of 0.45. So, any solubility scores larger than 0.45 were expected to have a higher solubility than the average soluble *Eschericia coli (E*.*coli)* proteins from the experimental solubility dataset and vice versa [68]. Additionally AlgPred (http://crdd.osdd.net/raghava/algpred/) was used for prediction of allergenicity of the vaccine construct [69].

### Secondary structure prediction

Secondary structure is the general two-dimensional form of local segments of a protein or nucleic acid sequence. In proteins, secondary structure can be defined by the hydrogen bond patterns between backbone amino and carboxyl groups of proteins which is found in an atomic-resolution structure.

PSIPRED (http://bioinf.cs.ucl.ac.uk/psipred/) [70] an artificial neural network was utilized to predict the alpha helix, beta sheet and coil structure of the vaccine construct. PSIPRED used PSI-BLAST (Position-Specific Iterated–BLAST) for secondary structure prediction. PSIPRED predicts the secondary structure of protein by two feed forward neural network processes, a) First network and b) Second network. The first network is utilized for initial prediction where the second network refines the structure obtained from the first prediction [70].

### Tertiary structure prediction, refinement, and validation

The tertiary structure of multi-epitope vaccine subunit vaccine was predicted by using Robetta (http://robetta.bakerlab.org/), a protein structure prediction online server. Robetta is continually evaluated through CAMEO (continuous automated model evaluation) for tertiary structure prediction. Also, Robetta server utilizes custom sequence alignments for homology modeling, constraints, local fragments and so on. However, it uses the PDB100 template database and a co-evolution based model database (MDB) which is updated weekly.

On the other side, protein model structure produced by different protein structure prediction strategies mostly relies on similarity between the input and available template structure of PDB. So, the whole protein structure needs to be refined for improving the template-based protein model structure beyond the precision level. Therefore, ModRefiner server (https://zhanglab.ccmb.med.umich.edu/ModRefiner/) was used to enhance the accuracy of the predicted 3D modeled structure [71]. Furthermore, PROCHECK (https://servicesn.mbi.ucla.edu/PROCHECK/) [72] was used to analyze residual geometry of the refined vaccine construct and predict the improved stereochemical quality of the construct for validation.

### Discontinuous B-cell epitope prediction

Discontinuous or conformational epitopes are those that consist of multiple, distinct segments from the primary amino-acid sequence. Conformational epitopes residues are distantly placed in the sequence and brought together by physicochemical folding of the Protein’s tertiary structure. [73]. Most of the acknowledged epitopes in a protein sequences are discontinuous [74]. So, analysis of discontinuous epitope in the final vaccine construct is prerequisite.

ElliPro (http://tools.iedb.org/ellipro/) [75] a discontinuous B cell epitope prediction server at IEDB was used for discontinuous/conformational B cell epitope prediction in the final vaccine construct. ElliPro scored each output epitope based on PI (Protrusion Index) value of epitope residues. The PI value of epitope residues is the criteria for discontinuous epitopes identification in a protein. Tertiary structure of the refined vaccine model was provided in the ElliPro server for conformational epitope prediction.

### Molecular docking of the vaccine candidate with immune receptor

Molecular docking is an *in silico* approach that can evaluate the binding correlation between a ligand and a receptor molecule [76]. We assessed PatchDock server (https://bioinfo3d.cs.tau.ac.il/PatchDock/) for analyzing protein-protein docking interaction to determine the binding affinity of our designed peptide vaccine with immune receptor TLR-3, 4, 7 and 8 (PDB ID: 1ZIW, 4G8A, 5gmf and 3W3J respectively) [77]. PDB files of vaccine protein and all the TLRs receptors were uploaded to the PatchDock servers for docking interaction. Later, FireDock output refinement of PatchDock server was used for refining the complexes.

### Molecular dynamics simulation

Molecular dynamics simulation is recognized as an effective method for molecular analysis of biological systems [9,78,79]. In previous studies it was used to check the stability of different protein complexes [80–82]. However we used the Linux-based GROMACS [83] software for molecular dynamics simulation to understand the structural properties and interaction between TLR-4 and predicted vaccine protein at microscopic level. GROMACS can be performed using a variety of force fields, such as GROMOS, OPLSS, AMBER, and CHARMM [84]. In our study, the simulation was executed with GROMACS 2020.1 package with the force field as CHARMM36 [85]. The protein salvation was executed with SPC water model in a cubic box. About 83842 water molecules were added to the system and 25 CL molecules were added to neutralize the system. Meanwhile the system was equilibrated for 100ps.

The simulation of docked vaccine was performed to study interaction pattern of vaccine and changes in the complex after molecular docking with respective ligand. Additionally, the molecular dynamics simulation was carried out for 10ns long.

We analyzed the RMSD (Root Mean Square Deviation of the atomic position), RMSF (Root Mean Square Fluctuations) and Rg (Radius Gyration) of the vaccine-receptor complex. The RMSD, RMSF and Rg can be used to obtain information about our Biosystems. The RMSD includes the calculation concerning the reference structure (backbone) of the average atom location in a molecule. This method is used to calculate the average changes in atom displacement for evaluating the conformational shifts and Biosystems stability. The average deviation of a particle (for example, a protein residue) from the reference location (usually the average particle location) over time is calculated with root-mean-square fluctuation (RMSF). RMSF therefore analyzes the structural portions which fluctuate more (or less) from their mean structure. The gyration radius (Rg) represents the compactness of the structure. The lower degree of fluctuation with its simulation stability indicates the higher compactness and rigidity of the device.

### *In Silico* development of adenovirus based vaccine

To construct an adenovirus based vaccine, Kozak consensus sequence containing codon for methionine was added at the N-terminal end of vaccine protein. Codon optimization was done in GenScript server for *Homo sapiens* expression system. Four restriction sites (*BglII*, *EcoRV*, *PmeI* and *PacI*) were avoided from the optimized cDNA sequence. GC content, CAI values were again evaluated, and only highly efficient cDNA sequence was inserted in pAdTrack-CMV shuttle vector [86] in between *BglII* and *EcoRV* restriction site under strong CMV promoter through SnapGene v5.1 [87] restriction cloning module. RNAfold server was also employed to analyze the translation efficiency and thermodynamic stability of expressed mRNA sequence [88].

## Results

### Retrieval of protein sequences

The amino acid sequences of structural and non-structural proteins of WNV virus (UniProt ID- Q9Q6P4) were retrieved from UniProt Database. The physiochemical properties (i.e. Molecular weight, Aliphatic Index, Theoretical pI, Grand average of hydropathicity and Instability index) of each protein were analyzed using ExPASy ProtParam tools (Table 1).

**Table 1:** Physiochemical parameters of structural and non-structural protein of West Nile virus.

Protein Name	Number of amino acids	Molecular weight (Da)	Theoretical pI	Instability index	Aliphatic index	Grand average of hydropathicity
**Capsid C**	105	11718.20	12.31	46.07	92.95	-0.209
**Protein prM**	167	18465.41	9.10	45.03	88.08	0.037
**Envelope**	501	53620.34	7.66	23.78	84.45	0.058
**NS1**	352	39749.80	5.73	45.87	73.10	-0.575
**NS2A**	231	25391.57	9.67	42.24	133.90	0.790
**NS2B**	131	14466.79	4.22	28.53	96.11	0.369
**NS3**	619	68995.37	7.32	31.42	76.56	-0.457
**NS4A**	126	13685.60	6.70	33.78	120.00	0.660
**NS4B**	255	27588.36	8.93	30.82	109.02	0.430
**NS5**	905	103683.06	8.63	36.25	72.87	-0.580

### Linear B Cell (BCL) epitope prediction

**Fig 1:**
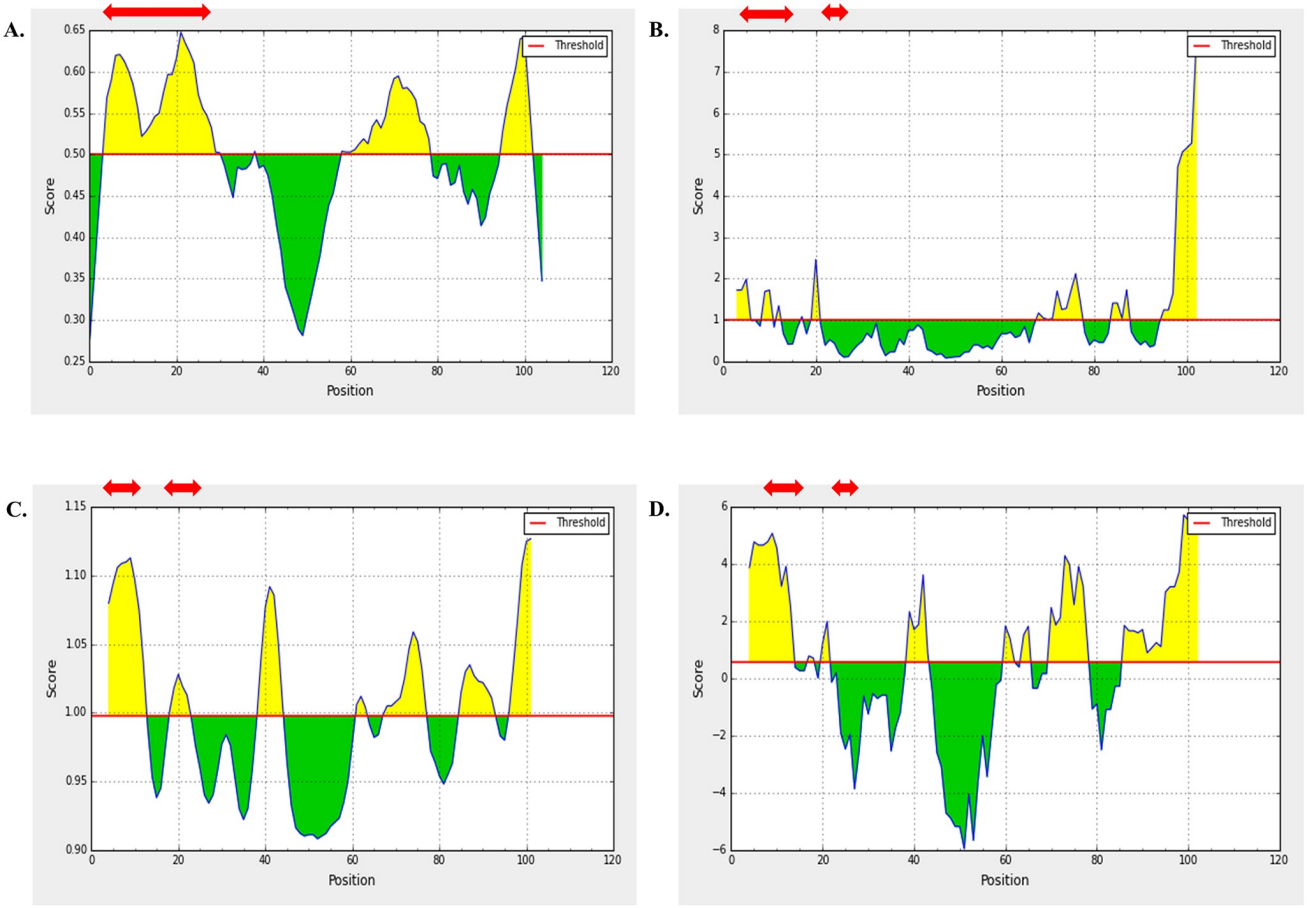
B cell Epitope prediction of Capsid C protein at IEDB linear epitope prediction tool. Predicted peptide regions above the threshold values (yellow region) are shown in red arrow. Methods used for prediction are enlisted as: (A) BepiPred 2.0, (B) Emini Surface Accessibility tool, (C) Karplus & Schulz Flexibility tool, and (D) Parker Hydrophilicity tool.

**Fig 2:**
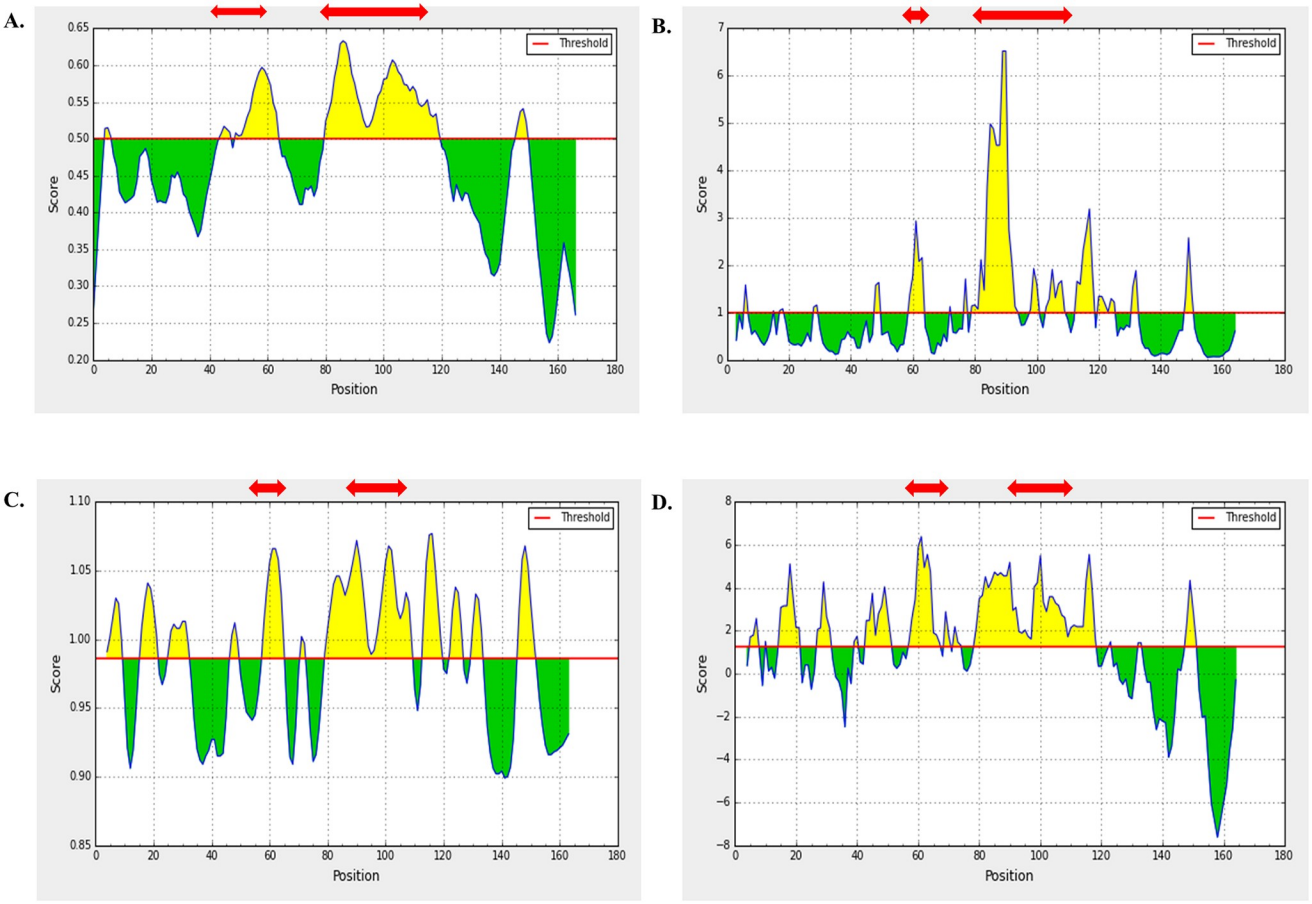
B cell Epitope prediction of prM protein at IEDB linear epitope prediction tool. Predicted peptide regions above the threshold values (yellow region) are shown in red arrow. Methods used for prediction are enlisted as: (A) BepiPred 2.0, (B) Emini Surface Accessibility tool, (C) Karplus & Schulz Flexibility tool, and (D) Parker Hydrophilicity tool.

**Fig 3:**
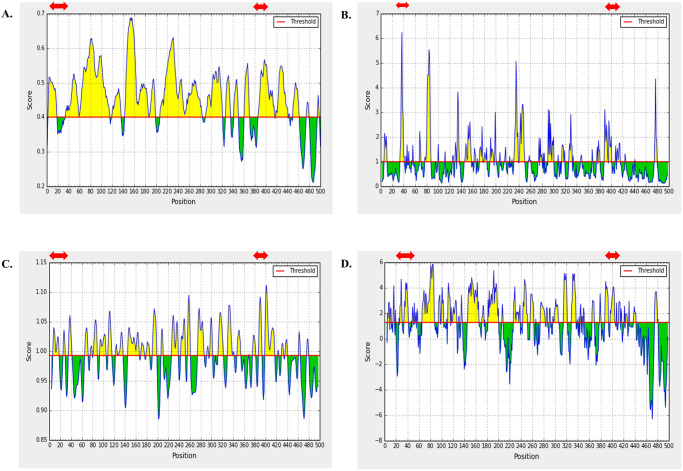
B cell Epitope prediction of Envelope protein at IEDB linear epitope prediction tool. Predicted peptide region above the threshold values (yellow region) are shown in red arrow. Methods used for prediction are enlisted as: (A) BepiPred 2.0, (B) Emini Surface Accessibility tool, (C) Karplus & Schulz Flexibility tool, and (D) Parker Hydrophilicity tool.

Linear B cell epitopes were screened only from the three structural proteins. Several B cell epitopes prediction server including ABCpred, Bepipred, and Emini Surface Accessibility Prediction tool of IEDB were used for identification of potential B cell epitopes from WNV structural proteins. Linear B cell epitopes of 16-mer predicted through ABCpred server were cross-checked with BepiPred 2.0 and Emini Surface Accessibility Prediction tool of IEDB server and overlapped epitopes were shortlisted (S1 File). Moreover, we analyzed the antigenicity, non-toxicity, and non-allergenicity of the overlapped peptides. Based on the best score of the analysis, two epitopes from each protein was selected as the final B cell epitopes. As these proteins are located in the outer surface of the virus and thus highly mutable, selection of two epitopes were considered. Finally, these 6 epitopes were further analyzed through Karplus & Schulz Flexibility and Parker Hydrophilicity Prediction tool at IEDB. All of the predicted peptide fragments were obtained to have satisfactory surface accessibility and flexibility (Figs 1–3).

### CD8+ T cell (CTL) epitope prediction

NetCTL 1.2 server was used to predict the T cell epitopes of corresponding WNV proteins (both structural and non-structural). Full screening of epitope is provided in Supplementary S2 File. Using 75% threshold value, NetCTL 1.2 server predicted a total of 791 epitopes for all 10 proteins against 12 MHC supertypes of humans. Among those epitopes, 88 were predicted as antigenic, immunogenic, non-allergenic, non-toxic and showed outside topology of transmembrane helices. After cross checking in NetMHC 4.0 server a total of 32 epitopes were revealed that had capacity to bind to different mouse alleles; indicating those epitopes able to illicit immunogenicity in both humans and mice. However, NS4B protein failed to generate any common epitope. One epitope from each protein was then shortlisted on the basis of respective human HLA alleles (epitopes which can bind to most HLA alleles under low percentile rank e.g. IEDB percentile rank ≤ 1) for additional analysis (S2 File).

### CD4+ T cell (HTL) epitope prediction

After CD8+ T cell prediction, IDEB MHC II binding database predicted a total of 497 epitopes from all 10 proteins of WNV that can bind at least one mouse H-2-I allele. Among these epitopes, only 124 were selected that predicted to be antigenic, non-allergenic, non-toxic, and showed outside topology. Furthermore, IFNepitope server predicted 77 epitopes to be IFN-γ positive. Therefore, these 77 epitopes were also capable of inducing Th1 humoral responses. However, we found no IFN-γ inducing epitope from NS1 protein. Full screening is provided in supplementary S3 File. Based on respective human HLA alleles (e.g. epitopes which can bind to most HLA alleles under low percentile rank [IEDB percentile rank <10]), at most one IFN-γ positive epitope from each protein was shortlisted for further analysis (S3 File).

### Conservancy analysis of shortlisted epitopes

Conserved epitopes against multiple serovars are prerequisite to design a universal broad-spectrum vaccine. Hence, each of the CTL, HTL, and BCL epitopes predicted from previous pipelines were screened with respective 100 homologous protein sequences of all other serovars of WNV. IEDB conservation analysis tool was used for conservancy analysis of epitopes against other serovars of WNV. At 90% sequence identity threshold, all the CTL, HTL, and BCL epitopes showed 100% conservancy (S1–S3 Files). The dataset for 100 homologous protein sequences of all other serovars of WNV can be found in S4 File.

To investigate whether the conserved B cell epitopes (as predicted by the conservancy analysis) have similar sequence homology IEDB conservancy analysis tool was applied and compared against the reference polyproteins of Dengue and Japanese encephalitis viruses (NCBI accession no: NP_059433.1, NP_056776.2, YP_001621843.1, NP_073286.1 and UniprotKB ID: P0DOH7). Similarity in epitopes indicate possible cross-reaction. None of these epitopes showed conservancy against the reference proteome of both Dengue and Japanese encephalitis viruses (S1 File); indicating that no cross-reactivity of this epitope with other arboviruses. The dataset for referral polyprotein of Dengue and Zika viruses can be found in S4 File.

### World population coverage

MHC-I and MHC-II prediction server at IEDB predicted the corresponding allele for each of the proposed nine CD8+ (CTL) and nine CD4+ (HTL) epitopes. The output was given in units of IC50 nM. Peptides with IC50 values of <50, <500 and <5000 nM are considered to have high, intermediate and low affinity respectively. Here, alleles with IC50 value <500 were selected as optimum binders for the population coverage analysis.

**Fig 4:**
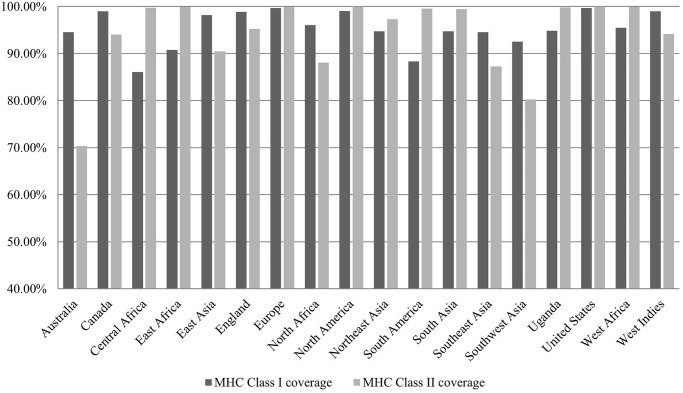
Population coverage analysis of finalized epitopes (MHC-I and MHC-II). Europe, England, North America, United States, and West Africa showed the highest (100%) population coverage.

Population coverage was calculated with corresponding HLA alleles (IC50 <500 nm) of selected nine CTL and nine HTL epitopes (one epitope from each protein) through population coverage tool at IEDB interface. We observed 98.55% (CTLs) and 98.99% (HTLs) of world population coverage. A strong immune response requires activation of both CD4+ and CD8+ T cells. Population coverage analysis of the combined MHC-I and MHC-II epitopes revealed an average of 99.99% coverage of the population worldwide. Whereas, the Europe, England, North America, United States, and West Africa showed the highest (100%) population coverage of the combined MHC-I and MHC-II epitopes (Fig 4).

### Vaccine construction

**Fig 5:**
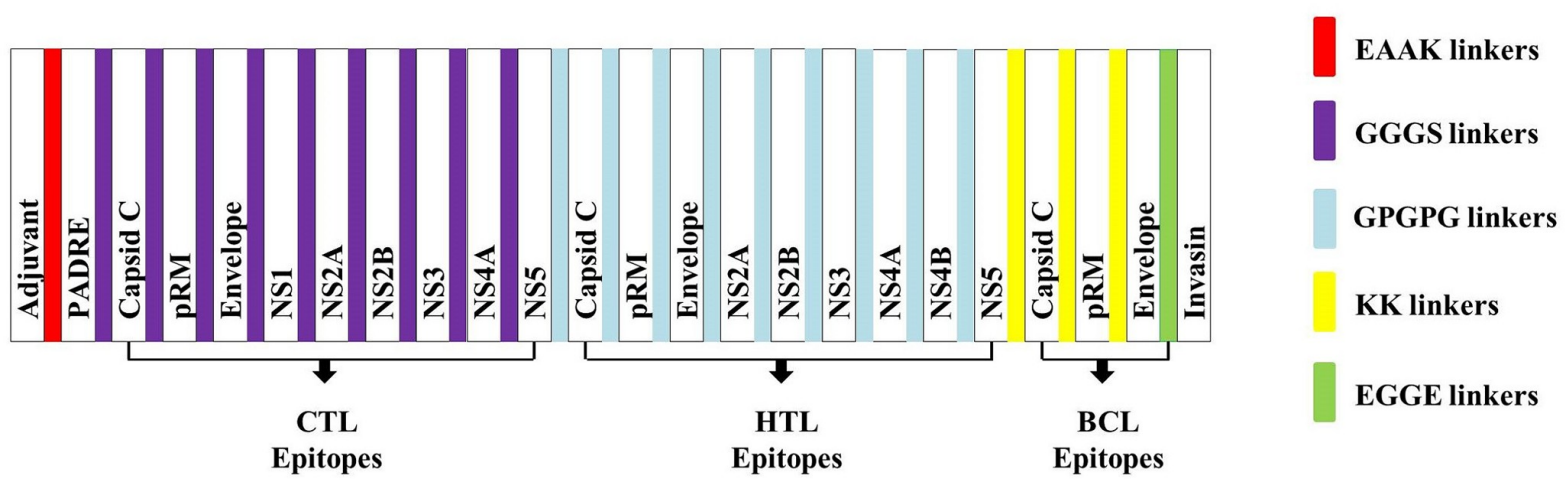
Schematic representation of WNV vaccine construct: A 488 amino acids long multi-epitope vaccine sequence consisting an adjuvant at N-terminal end linked with a multi-epitope sequence with the help of EAAAK linker (Red) followed by PADRE sequence. CTL epitopes were linked through GGGS linkers (blue). HTL epitopes were linked through GPGPG linkers (Sky). BCL epitopes were linked through KK linkers (Yellow). Invasin molecule linked through EGGE linker (Green).

The chimeric vaccine was constructed with final nine CTL, nine HTL, and six B cell epitopes (BCL). Epitopes were joined based on genomic arrangement of their corresponding proteins in virus. GGGS linkers were added to separate each CTL epitope from other CTL epitopes, whereas GPGPG linkers were used to separate each HTL epitope from other HTL epitopes. KK linker was added to separate each BCL epitope from other BCL epitopes. Beta defensin-3 (GIINTLQKYYCRVRGGRCAVLSCLPKEEQIGKCSTRGRKCCRRKK), a TLR4 agonist, was added at N-terminal end of the construct followed by EAAAK linker and PADRE sequence (AKFVAAWTLKAAA). GGGS linker was used to separate PADRE sequence from CTL. The C-terminal invasin sequence (TAKSKKFPSYTATYQF) of *Yersinia* was added at the C-terminal end of the construct which was separated from the BCL epitope by an EGGE linker. These linkers would ensure maximal immunity and efficient presentation of epitopes in the body while PADRE sequence would induce CTL responses and ensure maximal MHC-II allele coverage. The final construct of multi-subunit vaccine comprises 488 amino acid residues and is represented in Fig 5 and S5 File.

### Immunogenic and physiochemical profiles

The final vaccine construct was found antigenic (VaxiJen score 0.7) in nature. Physiochemical analysis revealed that the construct was highly stable (Instability index 29.55), basic in nature (theoretical PI 10.21), and highly thermostable (AI value 84.36). The estimated half-life was found 30 hours in mammalian reticulocytes (*in vitro*), more than 20 hours in case of yeast cell (*in vivo*) and over 10 hours if expressed in *E*. *coli* (*in vivo*). Molecular weight (50.914 kDa) of the construct was in favorable range. The recombinant vaccine protein was also predicted to be soluble (0.551) in *E*. *coli* heterologous expression system. Furthermore, AlgPred predicted the construct to be non-allergenic in nature (Table 2).

**Table 2:** Physiochemical, immunogenic and solubility properties of vaccine protein.

Parameter	Value	Comment
**Molecular Weight (kDa)**	50.914	Suitable
**Theoretical PI**	10.21	Basic in nature
**Estimated Half Life**	30 h	Mammalian reticulocyte (*in vitro*)
	>20 h	Yeast (*in vivo*)
	>10 h	*E*. *coli* (*in vivo*)
**Instability Index**	29.55 (stable)	Stable
**Aliphatic Index**	84.36	Thermostable
**GRAVY**	0.170	Slightly hydrophobic
**VaxiJen Score**	0.7042	Antigenic
**AlgPred**	-	Non-allergic
**Protein-Sol**	0.551	Highly soluble in *E*. *coli*

### Secondary and tertiary structure prediction, refinement and validation

Secondary structure was predicted on the basis of the amino acid sequence of the final vaccine construct using PSIPRED. It was revealed that the predicted structure of the vaccine protein has 103 alpha helix (21%), 145 sheet (29%), and 240 (50%) coil regions.

**Fig 6:**
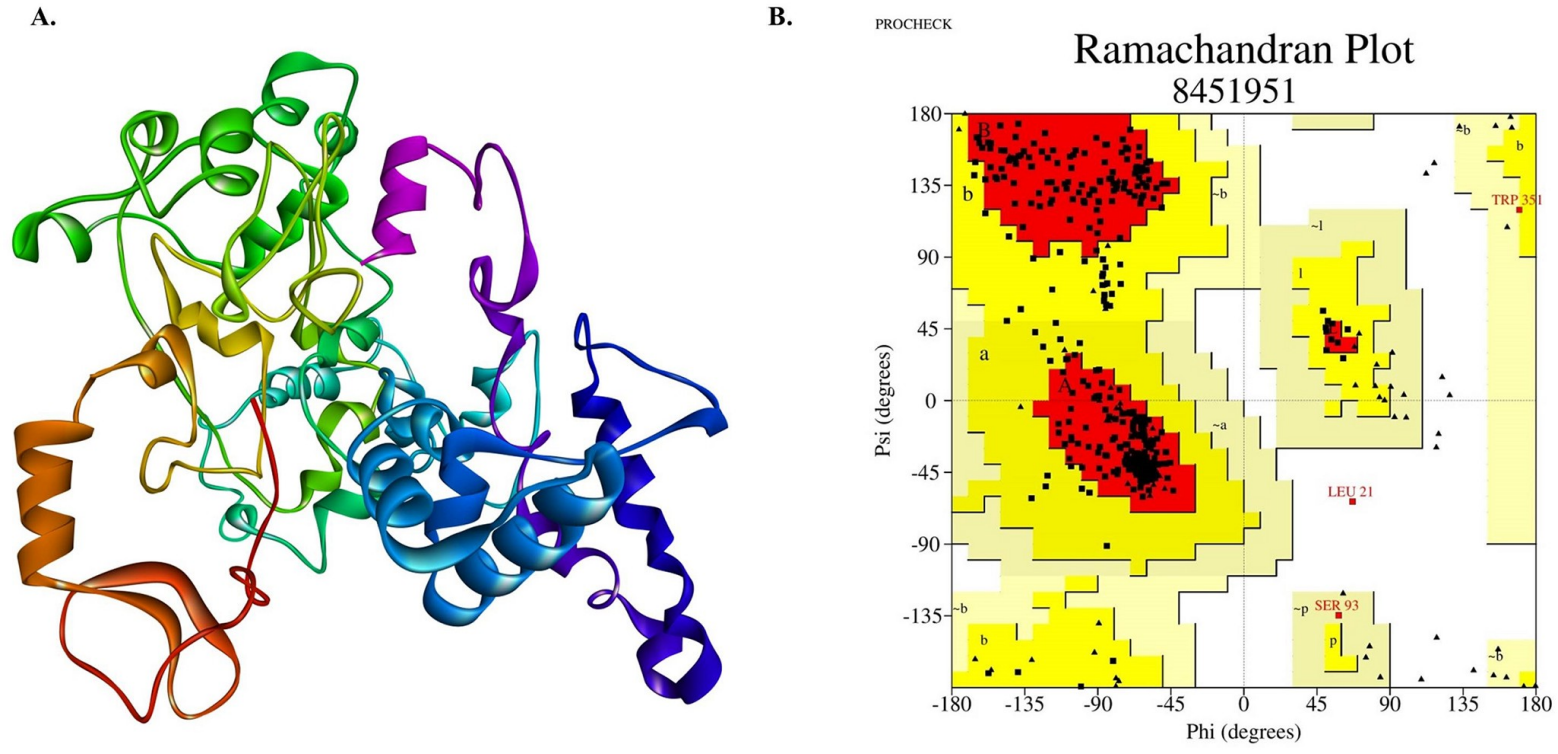
Tertiary structure and Ramachndran plot assessment of the refined vaccine protein. a) Model-5 from Robetta server (Homology modelling server) after refinement; b) Ramachandran plot analysis of refined vaccine protein by PROCHECK server. Refined model contains 88.1% residues in most favored region, 5.3% residues in additionally allowed region, 0.5% in generously allowed region and only 0.3% in disallowed region.

ROBETTA server generated 5 models (3D) of vaccine protein using comparative modeling. Among those, pre-refinement quality analysis through PROCHECK tool revealed model no. 5 as the best candidate as it had highest number of residues in most favored region (83.1%). To improve the standard of predicted 3D modeled structure beyond the accuracy, refinement was performed using ModRefiner. Refined structure showed RMSD value of 1.268 and TM-score 0.9755 to initial model (model no. 5). Post-refinement quality analysis through PROCHECK tool revealed improved Ramachandran plot as 88.1% residues are in most favored regions, 11.1% in additionally allowed region, 0.5% in generously allowed region, and only 0.3% in disallowed region (Fig 6 and S1 Table).

### Conformational B cell epitope prediction

ElliPro at IEDB was utilized for identifying the discontinuous B-cell epitopes. The PDB structure of the vaccine protein was used as an input for the prediction of conformational epitopes in the tertiary structure of the vaccine construct. ElliPro identified seven epitopes on the three-dimensional structure of the vaccine construct where highest predicted score was 0.735 with residual position of 359–386 (S1 Fig and S6 File).

### Docking analysis of the vaccine construct with toll like receptor (TLR)

**Fig 7:**
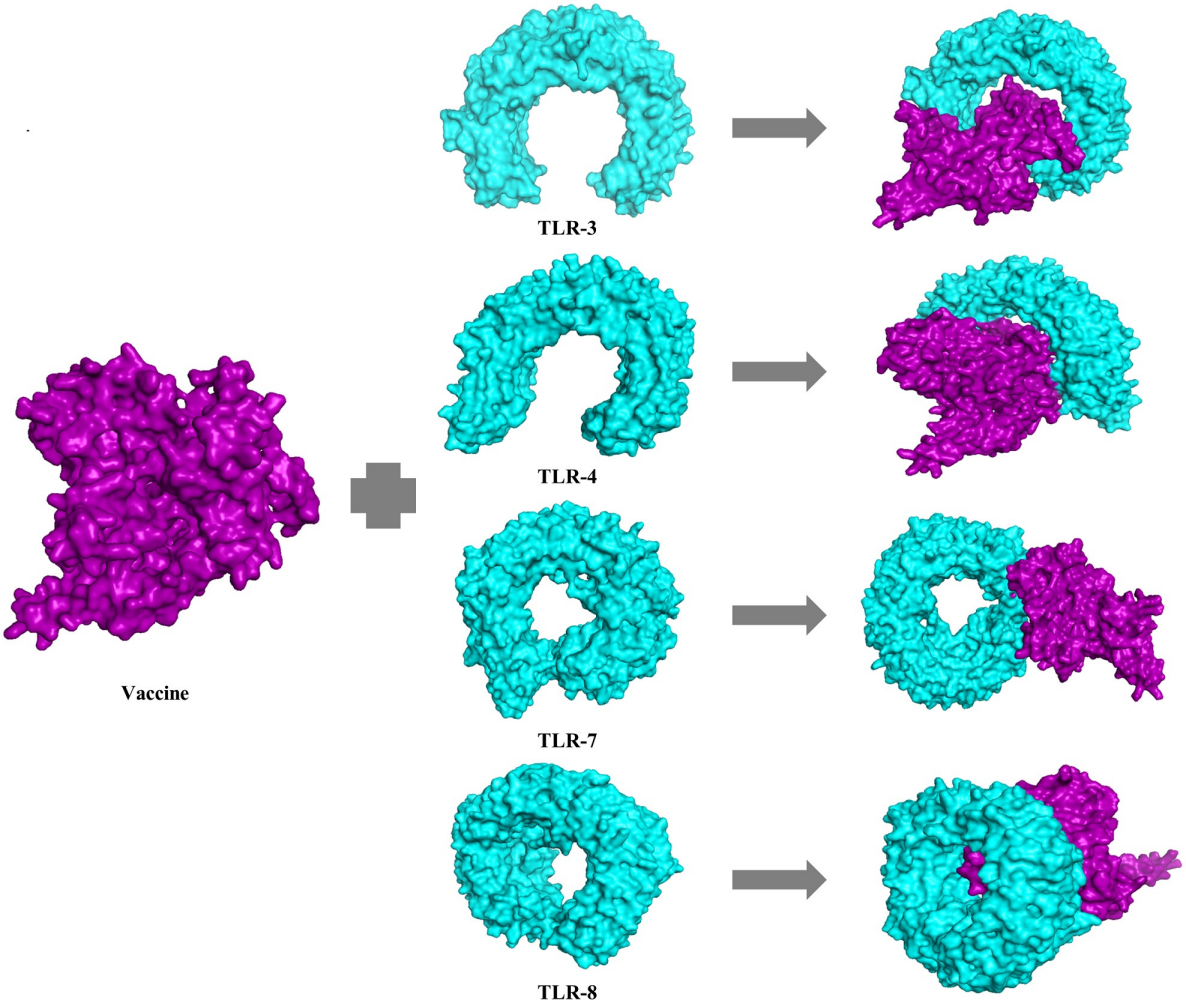
Ligand-receptor docked complex of vaccine protein with human toll like receptor (TLR)-3, 4, 7 and 8. Vaccine protein is represented as purple color while TLR-3, 4, 7, 8 as aqua color. The global energy of vaccine protein with other toll like receptors were found -22.08 for TLR3, -26.96 for TLR4, -0.06 for TLR7 and -6.29 for TLR respectively.

Vaccine construct was docked with human TLR- 3, 4, 7, and 8 receptors (Fig 7). PatchDock webservers generated a total of 20 best protein-ligand complexes of vaccine protein and all the different TLRs as output along with respective free binding energy. Patchdock server ranked top ten docked models based on the protein’s surface as well as electrostatic complementarity. FireDock web tool was further utilized to refine and rescore the docked complexes. After refinement, solution 2 was ranked as the best model for TLR-4 and vaccine complex whereas solution 1 for TLR-3, solution 5 for TLR-7 and solution 1 for TLR-8 and vaccine complex. However, the best docked complex was found to be TLR-4 and vaccine complex with global energy of -26.96 which was selected for further molecular dynamics simulation. Other parameters like atomic contact energy (ACE), electrostatic interactions, and Van Der Waals associations were 14.10, 28.65, and -42.76 respectively. The global energy of vaccine protein with other TLRs were -22.08 for TLR3, -0.06 for TLR7, and -6.29 for TLR8. The lowest binding indicates that the docked complex signifies the highest binding affinity between vaccine construct and TLR-4.

### Molecular dynamics simulation of receptor ligand complex

**Fig 8:**
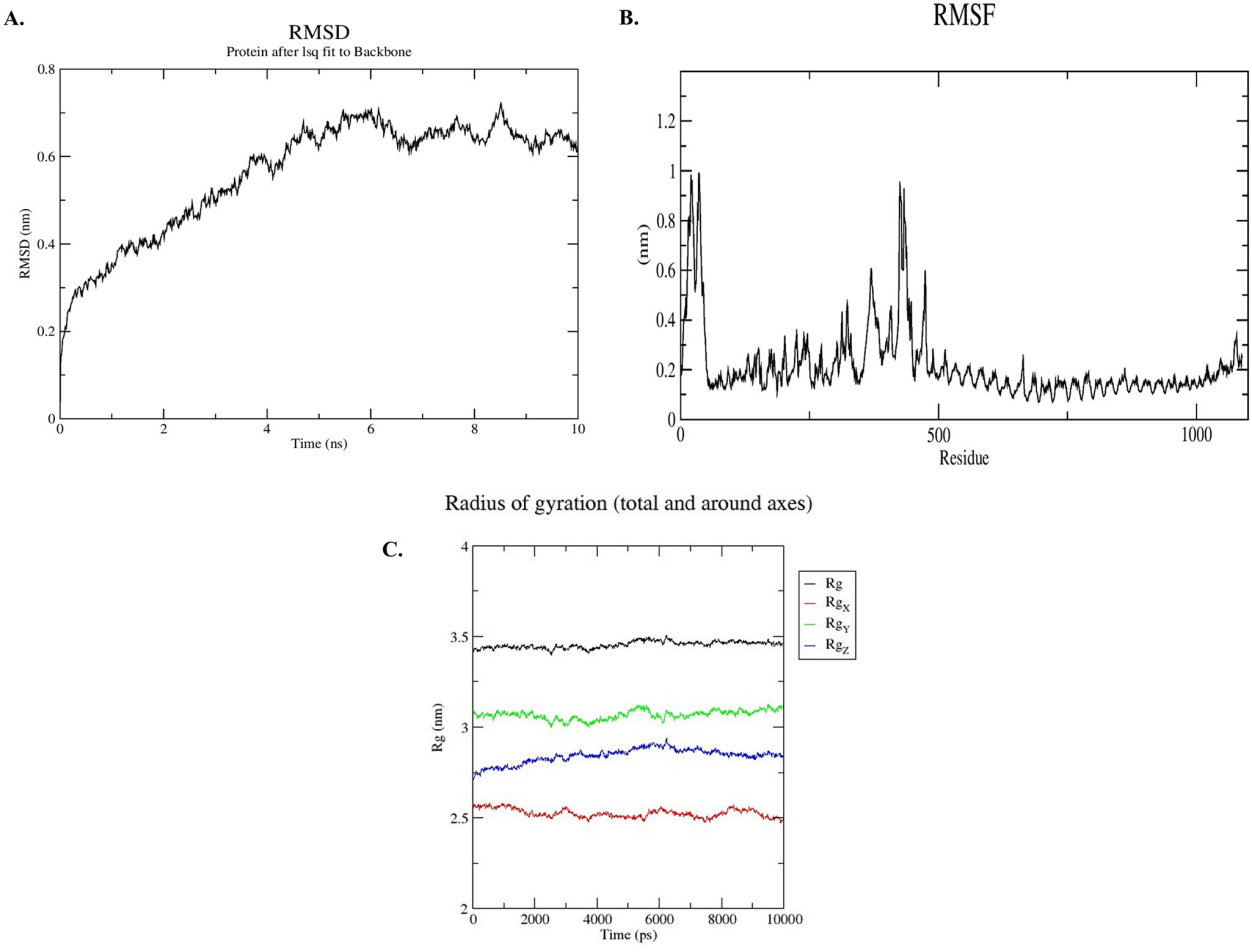
Molecular dynamics simulation of the vaccine protein with human toll like receptor-4. (A) RMSD-Root Mean Square Deviation of docked complex shows very minor deviation reflecting the stable interaction between vaccine protein and TLR-4 molecule. (B) RMSF-Root Mean Square Fluctuation plot of docked protein complex remained fairly flat reflecting the flexibility of side chain of docked protein complex. (C) Rg plot of docked complex shows a relatively flat curve suggesting stable vaccine-receptor complex.

The molecular dynamics simulation was performed for 10ns timescale and RMSD, RMSF, and Rg plots were drawn to check the flexibility and stability of the vaccine-receptor complex. The RMSD plot showed significant results highlighting the stability of the vaccine-receptor complex (Fig 8A). The RMSD of the protein fitting to its backbone showed deviations initially which was later stabilized after 5ns. The vaccine-receptor complex showed deviation of less than 0.2 nm. The deviations higher than that correlate to instability. The vaccine-receptor showed less deviation after 5ns mark indicates the stability of the complex.

Root means square fluctuations (RMSF) helps to explain the protein area that fluctuate throughout the simulation. The flexibility of each residue is therefore measured in order to provide a deeper insight into the degree to which protein binding influences complex’s flexibility. The RMSF plot showed very less fluctuations of the atoms in the complex (Fig 8B); suggesting that the amino acid residues of the protein remained stable throughout the simulation. This strengthens the fact that the complex was stable after the docking of the vaccine on to the receptor.

As the radius of gyration shows the compactness of the complex structure, there should be fewer movements or deviation of the positions of the residues of the complex in the simulation trajectory in order to deem the complex to be stable. The Rg plot (Fig 8C) showed less deviation throughout the simulation process and the relatively flat curve suggesting the compactness and stability of the complex.

The molecular dynamics simulation results showed significant structural flexibility and stability of the vaccine-receptor complex; thus implies a positive response by the immune receptor.

### *In silico* cloning in adenovirus vector and mRNA secondary structure analysis

GenScript Codon Optimization tool (https://www.genscript.com/) was utilized to generate reversely transcribed nucleotide sequences of recombinant vaccine protein for human cell expression system. GC content of obtained cDNA (1467 bp) was 62.25% whereas CAI value was calculated as 0.92. These values above indicate efficient expression of optimized codon in human host cell. This sequence was inserted as GOI under strong CMV promoter in pAdTrack-CMV vector through SnapGene v5.1 software. *BglII* restriction site was added at the 5’ end followed by Kozak sequence which ensures maximal expression of the desired gene. Stop codon TAA was added at 3’ end of the optimized codon followed by *EcoRV* restriction site. Stop codon would ensure termination of gene translation (S2A Fig). Total size of recombinant plasmid contained 10688 base pairs.

mRNA secondary structure was predicted through aforementioned RNA fold server. The minimum free energy -624.40 kcal/mol indicates thermodynamic stability of mRNA structure. Furthermore, first 10 nucleotides of mRNA secondary structure were free from forming any pseudoknot or long stable hairpin thereby ensuring efficient translation initiation from mRNA structure (S2B Fig).

## Discussion

West Nile Virus is recognized as a reemerging global pathogen and now endemic in African continent and subcontinent of Asia and Europe [89,90]. The virus is also endemic of encephalitis and meningitis in humans in United States [91]. Along with other deadliest mosquito borne virus such as Chikungunya virus and Zika virus, WNV is enlisted as the B category pathogen by National Institute of Allergy and Infectious Diseases (NIAID) [92]. However, there is no FDA-approved therapeutic treatment available for the diseases caused by WNV and even no commercial vaccine has developed so far [93]. While economically insignificant market and high cost-to-benefit ratio hinder the development and commercialization of WNV vaccine for human use [94], researchers have conducted several studies to find out immunodominant epitopes [7,95–97].

Recent advances in computational biology, immunoinformatics, and reverse vaccinology can facilitate in designing safe and efficient vaccines in a time and cost-effective manner [29,98]. Biological data such as genomics and proteomics are being used to mine potential epitopes, design long lasting immunogenic subunit vaccine and laboratory validation of that candidate [99,100]. Therefore, the present study was undertaken to screen out the potential immunodominant B cell as well as CTL and HTL epitopes from all different structural and non-structural proteins of WNV virus through immunoinformatics approach to design a safe, chimeric multi-subunit vaccine against the human host.

In this study, Linear B cell epitopes were screened only from structural proteins (E, PrM, and Capsid C) of WNV. Six epitopes, two from each protein (E_30-45_: CVTIMSKDKPTIDVKM, E_383-398_: YIVVGRGEQQINHHWH, PrM_56-71_: LSAGNDPEDIDCWCTK, PrM_93-108_: SLTVQTHGESTLANKK, Capsid C_2-17_: SKKPGGPGKSRAVNML Capsid C_17-32_: LKRGMPRVLSLIGLKR), showed potential antigenicity, surface accessibility, non-toxicity, and non-allergenicity. These epitopes may induce production of WNV specific antibodies in human. Neutralizing antibodies (IgM and IgG) appear to terminate viremia by controlling hematogenous spread of the WNV virus to Central Nervous System [101]. It was reported that highly immunogenic E protein of WNV, specifically part of domain II and III, showed promising results to generate neutralizing antibodies in clinical trial [102]. Domain III of E protein consists of many neutralizing epitopes [89,103–107] such as amino acid residues of E_302-309_, E_303-333_, E_333-365_, and E_389-391_ [108]. Even passively transferred E protein antisera eliminated viremia in mice and provided protection against WNV infection [109,110]. Passively transferred WNV immune sera thus protected B cell deficient mice from WNV infection [109]. One of our selected BCL E epitopes (E_383-398_: YIVVGRGEQQINHHWH) for vaccine construction contains experimentally proved amino acid residues capable of inducing neutralizing antibodies. Likewise E antigens, PrM antigens incorporated in ChimeriVax-WN02 vaccine also induced neutralizing antibodies and untill now are thought to be the most promising candidate as a human vaccine for WNV [111]. Although antibodies play crucial role in terminating viremia by controlling viral replication, T cell mediated responses are also vital and essential components in eradication of WNV from the body [101,112–117]. CTLs targets E, M, Capsid C, NS3, NS4A, and NS4B proteins of WNV [118]. In our study, we screened all structural and non-structural proteins of WNV to find out immunogenic CD8+ T cell epitopes. MHC class I epitopes selected for vaccine construction (E_480-488_: SIALTFLAV, PrM _153–161_: VVFVVLLLL, Capsid C_51-59_: LAFFRFTAI, NS1_120-128_: KSILFAPEL, NS2A_9-17_: FQLGLLVVF, NS2B_13-21_: LMFAIVGGL, NS3_358-366_: KTVWFVPSV, NS4A_63-71_: MTMGVFFLL, NS5_486-494_: LEFEALGFL) were immunogenic, non-toxic, and non-allergenic. These were predicted to bind with human alleles and at least one mouse allele. From published studies, we found an epitope (E_480-488_: SIALTFLAV) of E protein overlapped with experimentally validated MHC class I binding epitope (E_481-488_: IALTFLAV) in mouse model [95]. MHC class I molecule or T cell deficient mice have shown increased mortality while surviving mice have exhibited sustained and higher WNV burdens in both CNS and spleen [101,114]. Several studies reported that patients suffering from acquired deficiencies, genetic diseases with impaired T cell function and hematologic malignancies, have increased risk of developing West Nile Neuro-invasive Disease [119,120]. CTLs recognizing a WNV infected cell, proliferate and release different pro-inflammatory cytokines to directly lyse the infected cell [115,121,122]. Secretion of Interferon-γ by αβ and γδ T cells, a pro-inflammatory cytokine, showed reduced WNV load in mice brains. Adoptive transfer of these cells also protected mice by reducing the susceptibility to lethal WNV infection [119–121]. Hence, considering these aspects, selected CTL epitopes may trigger potential T cell immune responses *in vivo*.

CD4+ T cell (HTL) is another key immune cell type that acquires Th1 or Th2 phenotypes and stimulates immune responses [123,124]. CD8+ T cell, natural killer cell, and macrophages are activated by Th1 response. Whereas Th2 is responsible for the activation of B cell, isotype switching of B cells, affinity maturation, and antibody production which eliminates extracellular pathogens [115,116,125,126]. In our study, to induce HTL responses through vaccination, screening of potential HTL epitopes were performed. Shortlisted MHC class II epitopes from all structural and non-structural proteins of WNV were antigenic, non-toxic, and non-allergenic that can induce strong HTL immune responses. Importance of CD4+ T cell responses in WND were experimentally evaluated. Experiments in mouse models with CD4+ T cell and MHC class II deficiency aided in longer WNV persistence in brain and spinal cord. Levels of IgM and IgG were significantly decreased in MHC class II and HTL deficient mouse after a certain period and prolonged the viral persistence [127]. Only IFN-γ inducing immunogenic HTL epitopes (Capsid C_52-66_: AFFRFTAIAPTRAVL, PrM _129–143_: LRNPGYALVAAVIGW, E_356-370_: VTVNPFVSVATANAK, NS2A_85-99_: IQPVFMVASFLKARW, NS2B_103-117_: VCLAISAYTPWAILP, NS3_544-558_: PVWLAYKVAAAGVSY, NS4A_68-82_: FFLLMQRKGIGKIGL, NS4B_34-48_: LDLRPATAWSLYAVT, NS5_475-489_: AIWFMWLGARFLEFE) were used in vaccine construct since IFN-γ induces MHC class II expression and activation of macrophages [128]. It is well known that γδ T cells also play an important role in WNV infection by releasing IFN-γ molecule in a MHC independent restriction manner [118].

Conservancy analysis of epitopes in different WNV serovars suggested universal candidacy of the designed vaccine. Also, population coverage calculated for conserved MHC class I and II epitopes showed >99.99% worldwide coverage in a combined manner. EAAAK linker used in the constructed vaccine would ensure *in vivo* separation of individual epitopes effectively in natural environment [67,80]. As GGGS linker is superior to AAY linker [129] in epitope based vaccine, we used this linker to separate CTL epitopes from PADRE sequence as well as individual CTL epitopes. HLA alleles are highly polymorphic in different populations, PADRE sequence was incorporated in the vaccine construct to overcome the problem. PADRE sequence binds to a wide variety of DR alleles [130] and ensure maximum efficacy and potency of subunit vaccines and also provide better CTL responses than PADRE deficient vaccines [131,132]. The adjuvant used in our study (Beta-defensin-3) demonstrated efficiency as an immune-stimulator in wet lab experiments [133–136]. The invasin molecule used in the vaccine construct can enhance the efficiency of human adenovirus based vaccines [137]. Finally, several predicted physiochemical parameters ensured immunogenicity, stability of vaccine construct and solubility in *E*. *coli* heterologous expression system [32]. The constructed multi-subunit vaccine showed higher binding affinity to human TLR-4 than TLR-3, TLR-7 and TLR-8. Molecular Dynamics simulation proved the stability of TLR-4 bound vaccine-receptor complex in cellular environment. Moreover, reversely transcribed optimized nucleotides of Adenovirus based subunit vaccine were found ideal for better heterologous expression. Kozak sequence incorporated at the upstream of optimized cDNA facilitate *in vivo* expression of stable mRNA [138] and may aid its recognition by eukaryotic ribosomes for efficient translation [139]. In recent years, new platforms of vaccine development such as virus like particles (VLPs), DNA, and mRNA-based vaccines are gaining attention. Vaccines are produced in both non-mammalian expression systems (bacterial, yeast, plant, and insect) and mammalian expression systems (including human cell lines) [140]. Mammalian expression system promote proper folding and post-translational modification (PTM) of designed vaccine, generally the preferred platform [140,141]. VLPs, however, do not favor mammalian expression systems for subunit vaccines due to its high production costs and low production yield [142]. On the contrary, adenoviral vector based DNA vaccines have been widely studied and extensively evaluated [143,144]. Adenoviral vaccines can be easily generated with higher tier at relatively low production cost [145].

Adenovirus based vaccines showed promising results in clinical trials against several infectious diseases such as HIV, Malaria, Hepatitis C virus, Ebola virus, Tuberculosis, Rotavirus and also cancers such as Prostate cancer, Lymphoma, melanoma, and others [146]. It is noteworthy that several Adenovirus based COVID-19 vaccines have already been approved for human use such as ChAdOx1 nCoV-19 or AZD1222 by University of Oxford and AstraZeneca, AD26.COV.2.S or JNJ-78436735 by Johnson & Johnson, Sputnik V by Gamaleya and Ad5-nCoV by CanSino Biologics Inc [147]. Johnson & Johnson have developed Adenovirus based Ebola Vaccine (Ad26.ZEBOV) which is currently in use with another vaccine as second dose [148]. They are also conducting clinical trial on Adenovirus based vaccine for Zika virus. Hence, Adenovirus based vaccine would be a potential candidate for eukaryotic expression system and many pharmaceutical companies have great interest for in Adenovirus based commercial vaccine development. Although our proposed multi-subunit vaccine showed promising results, it is based only on computational and reverse vaccinomics approaches. Therefore, to find out the most efficient candidate against WNV infection, we recommend further laboratory validation of the proposed vaccine construct and also comparison of the effectivity between protein vaccine and Adenovirus based vaccine.

## Conclusion

West Nile virus (WNV) is continuously spreading across Europe and other continents, i.e. North and South America, Asia, and many other regions of the world. Using immunoinformatics approach, we designed a multi epitope-based subunit vaccine against WNV. Human (HLA) and Mice (H-2) allele specific immunogenic epitopes including Cytotoxic T-lymphocytes (CTL), Helper T-lymphocytes (HTL), and B cell lymphomas (BCL) were screened and selected for vaccine design. Final vaccine constructs consist of B and T-cell epitopes, Universal pan-HLA DR or PADRE (13 mer) sequence, β-defensin-3 adjuvant (45 mer), and Invasin molecule at N-terminal of the construct with fusion protein linkers. Whereas, Invasin molecule (16 mer) was added at the C-terminal end to elicit strong immune responses. Conservancy and population coverage analysis of the promiscuous epitopes revealed the robust immune response against multiple serovars of the WNV and various ethnicities. Molecular docking revealed stronger binding affinity with human TLR-4; later confirmed by molecular dynamics simulation. Our computational analysis suggests that the constructed vaccine was highly immunogenic, safe, non-toxic, and stable in nature. Eventually, *in silico* cloning approaches of the vaccine construct ensure high expression within microbial system.

## Supporting information

S1 Fig Discontinuous B cell epitope (residual position of 359–386 highlighted in purple color) in the 3D model of Vaccine protein. The Highest predicted score was obtained 0.735.
(TIFF)

S2 Fig *In silico* restriction cloning of the gene sequence of final vaccine construct into pAdV-Track-CMV vector. a) Gene of Interest was inserted between *Bgl II* and *EcoRV* restriction sites (red marked). Recombinant plasmid consists of 10688 base pairs. b) Predicted secondary structure of mRNA for vaccine expressed through pAdVTrack-CMV. The 5’ end of predicted mRNA structure does not contain any pseudoknot or hairpin.
(TIF)

S1 Table Quality assessment of vaccine models (3D structures) and refined structure by PROCHECK server. (DOCX)

S1 File Linear B Lymphocytes (BCL) epitope screening from structural proteins (Envelope, PrM, Capsid C) of WNV. (XLSX)

S2 File Cytotoxic T Lymphocytes (CTL) epitope screening from structural and non-structural proteins of WNV. (XLSX)

S3 File Helper T Lymphocytes (HTL) epitope screening from structural and non-structural proteins of WNV. (XLSX)

S4 File Dataset of the sequences used in the study. (DOCX)

S5 File Sequence composition of the vaccine construct (Amino acid sequences and Optimized codons). (DOCX)

S6 File Discontinuous epitopes predicted in the final vaccine structure. (XLSX)

S1 Graphical abstract (TIF)
