# Supplementary figures and images for: Immunoinformatics and molecular dynamics approaches: Next generation vaccine design against West Nile virus
Source: PLoS One. 2021 Jun 17;16(6):e0253393. doi: 10.1371/journal.pone.0253393 (PMC8211291; doi:10.1371/journal.pone.0253393)

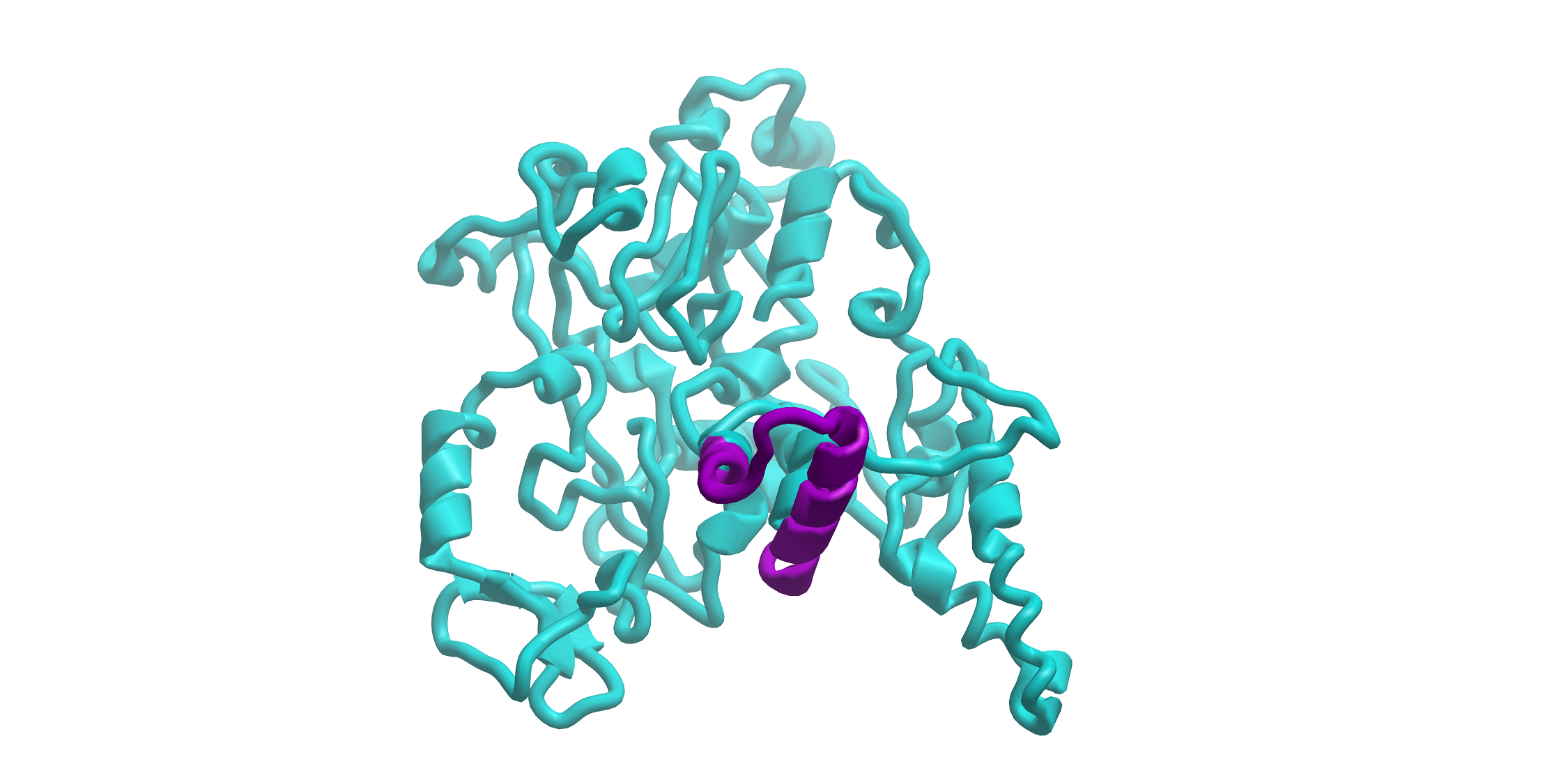

Supplement: S1 Fig — The Highest predicted score was obtained 0.735. (TIFF) [file pone.0253393.s001.tiff]

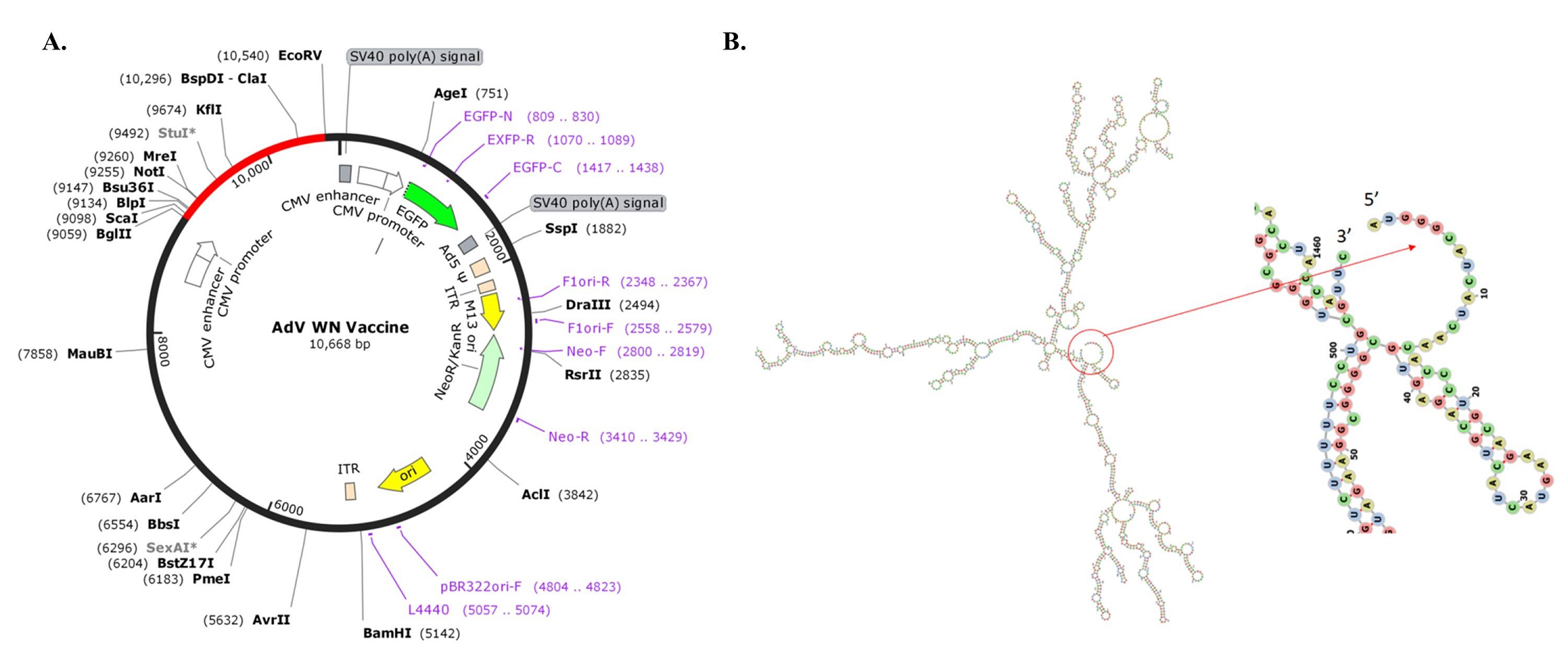

Supplement: S2 Fig — a) Gene of Interest was inserted between Bgl II and EcoRV restriction sites (red marked). Recombinant plasmid consists of 10688 base pairs. b) Predicted secondary structure of mRNA for vaccine expressed through pAdVTrack-CMV. The 5’ end of predicted mRNA structure does not contain any pseudoknot or hairpin. (TIF) [file pone.0253393.s002.tif]

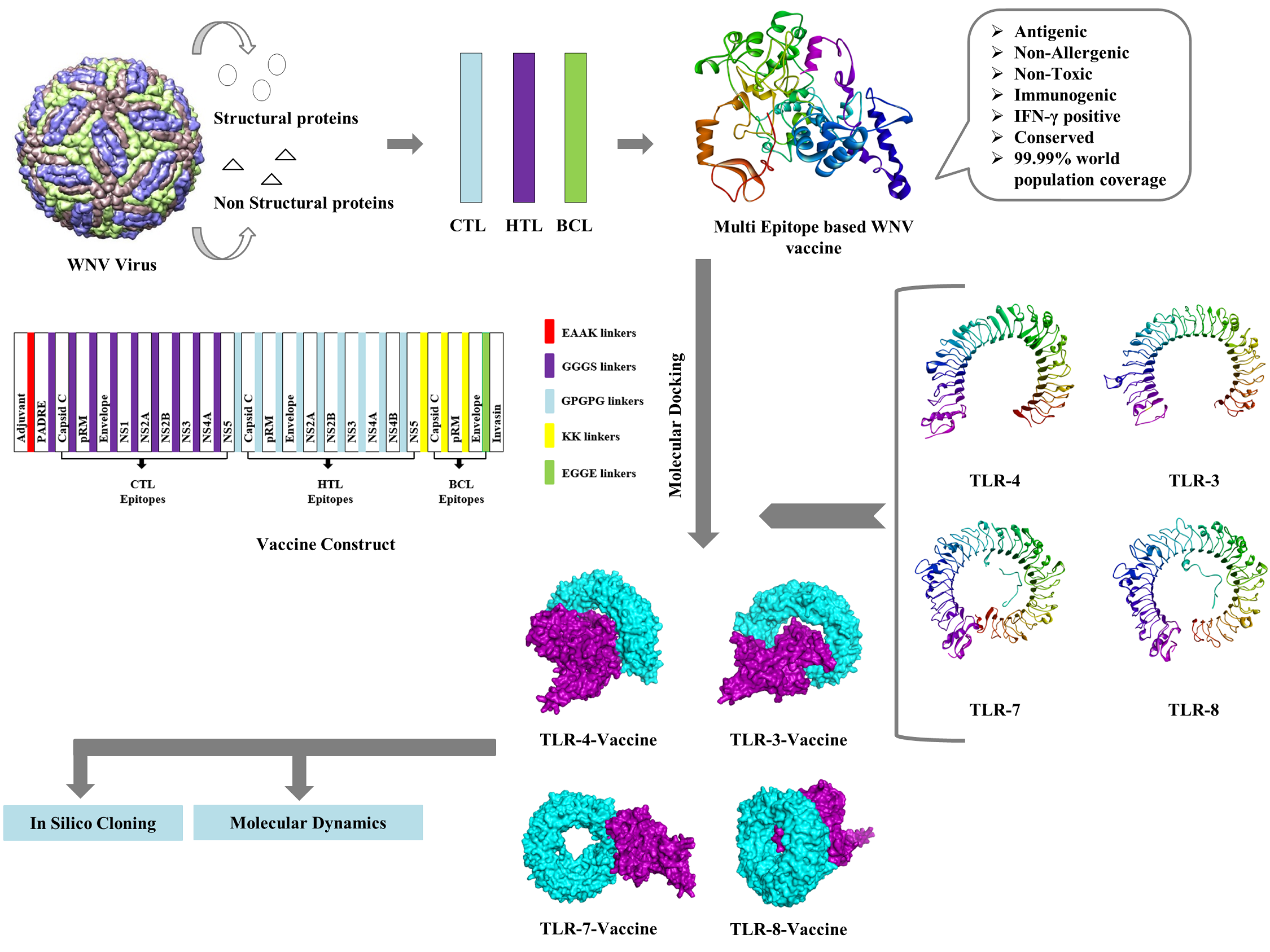

Supplement: S1 Graphical abstract — (TIF) [file pone.0253393.s010.tif]
