# Supplementary material for: Immunoinformatics and molecular dynamics approaches: Next generation vaccine design against West Nile virus
Source: PLoS One. 2021 Jun 17;16(6):e0253393. doi: 10.1371/journal.pone.0253393 (PMC8211291; doi:10.1371/journal.pone.0253393)
Supplement: S1 Table — (DOCX) [file pone.0253393.s003.docx]

**S1 Table. Quality analysis of modelled 3D structures of vaccine construct through PROCHECK server**

| **Model name** | **PROCHECK** | | | |
| --- | --- | --- | --- | --- |
|  | Most Favored Region  % (aa residue number) | Additionally Allowed  Region % (aa residue number) | Generously Allowed Region  % (aa residue number) | Disallowed Region  % (aa residue number) |
| Model 1 | 80.3 (297) | 17 (63) | 0.8 (3) | 1.9 (7) |
| Model 2 | 75.9 (281) | 20.5 (76) | 1.4 (5) | 2.2 (8) |
| Model 3 | 81.6 (302) | 15.7 (58) | 1.9 (7) | 0.8 (3) |
| Model 4 | 78.1 (289) | 20.3 (75) | 1.1 (4) | 0.5 (2) |
| Model 5 | 83.8 (310) | 15.4 (57) | 0.5 (2) | 0.3 (1) |
| Model 5 refined | 88.1 (326) | 11.1 (41) | 0.5 (2) | 0.3 (1) |
