# Supplementary material for: Immunoinformatics and molecular dynamics approaches: Next generation vaccine design against West Nile virus
Source: PLoS One. 2021 Jun 17;16(6):e0253393. doi: 10.1371/journal.pone.0253393 (PMC8211291; doi:10.1371/journal.pone.0253393)
Supplement: S4 File — (DOCX) [file pone.0253393.s007.docx]

**Supplementary File 4:** Dataset of the sequences used in the study

**Dengue Virus-Type 1 (Polyprotein- - 3449 AA)**

>NCBI accession no: **NP_059433.1 (Ref_Seq)**

MNNQRKKTGRPSFNMLKRARNRVSTVSQLAKRFSKGLLSGQGPMKLVMAFIAFLRFLAIP

PTAGILARWGSFKKNGAIKVLRGFKKEISNMLNIMNRRKRSVTMLLMLLPTALAFHLTTR

GGEPHMIVSKQERGKSLLFKTSAGVNMCTLIAMDLGELCEDTMTYKCPRITETEPDDVDC

WCNATETWVTYGTCSQTGEHRRDKRSVALAPHVGLGLETRTETWMSSEGAWKQIQKVETW

ALRHPGFTVIALFLAHAIGTSITQKGIIFILLMLVTPSMAMRCVGIGNRDFVEGLSGATW

VDVVLEHGSCVTTMAKDKPTLDIELLKTEVTNPAVLRKLCIEAKISNTTTDSRCPTQGEA

TLVEEQDTNFVCRRTFVDRGWGNGCGLFGKGSLITCAKFKCVTKLEGKIVQYENLKYSVI

VTVHTGDQHQVGNETTEHGTTATITPQAPTSEIQLTDYGALTLDCSPRTGLDFNEMVLLT

MEKKSWLVHKQWFLDLPLPWTSGASTSQETWNRQDLLVTFKTAHAKKQEVVVLGSQEGAM

HTALTGATEIQTSGTTTIFAGHLKCRLKMDKLTLKGMSYVMCTGSFKLEKEVAETQHGTV

LVQVKYEGTDAPCKIPFSSQDEKGVTQNGRLITANPIVTDKEKPVNIEAEPPFGESYIVV

GAGEKALKLSWFKKGSSIGKMFEATARGARRMAILGDTAWDFGSIGGVFTSVGKLIHQIF

GTAYGVLFSGVSWTMKIGIGILLTWLGLNSRSTSLSMTCIAVGMVTLYLGVMVQADSGCV

INWKGRELKCGSGIFVTNEVHTWTEQYKFQADSPKRLSAAIGKAWEEGVCGIRSATRLEN

IMWKQISNELNHILLENDMKFTVVVGDVSGILAQGKKMIRPQPMEHKYSWKSWGKAKIIG

ADVQNTTFIIDGPNTPECPDNQRAWNIWEVEDYGFGIFTTNIWLKLRDSYTQVCDHRLMS

AAIKDSKAVHADMGYWIESEKNETWKLARASFIEVKTCIWPKSHTLWSNGVLESEMIIPK

IYGGPISQHNYRPGYFTQTAGPWHLGKLELDFDLCEGTTVVVDEHCGNRGPSLRTTTVTG

KTIHEWCCRSCTLPPLRFKGEDGCWYGMEIRPVKEKEENLVKSMVSAGSGEVDSFSLGLL

CISIMIEEVMRSRWSRKMLMTGTLAVFLLLTMGQLTWNDLIRLCIMVGANASDKMGMGTT

YLALMATFRMRPMFAVGLLFRRLTSREVLLLTVGLSLVASVELPNSLEELGDGLAMGIMM

LKLLTDFQSHQLWATLLSLTFVKTTFSLHYAWKTMAMILSIVSLFPLCLSTTSQKTTWLP

VLLGSLGCKPLTMFLITENKIWGRKSWPLNEGIMAVGIVSILLSSLLKNDVPLAGPLIAG

GMLIACYVISGSSADLSLEKAAEVSWEEEAEHSGASHNILVEVQDDGTMKIKDEERDDTL

TILLKATLLAISGVYPMSIPATLFVWYFWQKKKQRSGVLWDTPSPPEVERAVLDDGIYRI

LQRGLLGRSQVGVGVFQEGVFHTMWHVTRGAVLMYQGKRLEPSWASVKKDLISYGGGWRF

QGSWNAGEEVQVIAVEPGKNPKNVQTAPGTFKTPEGEVGAIALDFKPGTSGSPIVNREGK

IVGLYGNGVVTTSGTYVSAIAQAKASQEGPLPEIEDEVFRKRNLTIMDLHPGSGKTRRYL

PAIVREAIRRNVRTLVLAPTRVVASEMAEALKGMPIRYQTTAVKSEHTGKEIVDLMCHAT

FTMRLLSPVRVPNYNMIIMDEAHFTDPASIAARGYISTRVGMGEAAAIFMTATPPGSVEA

FPQSNAVIQDEERDIPERSWNSGYDWITDFPGKTVWFVPSIKSGNDIANCLRKNGKRVVQ

LSRKTFDTEYQKTKNNDWDYVVTTDISEMGANFRADRVIDPRRCLKPVILKDGPERVILA

GPMPVTVASAAQRRGRIGRNQNKEGDQYIYMGQPLNNDEDHAHWTEAKMLLDNINTPEGI

IPALFEPEREKSAAIDGEYRLRGEARKTFVELMRRGDLPVWLSYKVASEGFQYSDRRWCF

DGERNNQVLEENMDVEIWTKEGERKKLRPRWLDARTYSDPLALREFKEFAAGRRSVSGDL

ILEIGKLPQHLTQRAQNALDNLVMLHNSEQGGKAYRHAMEELPDTIETLMLLALIAVLTG

GVTLFFLSGRGLGKTSIGLLCVIASSALLWMASVEPHWIAASIILEFFLMVLLIPEPDRQ

RTPQDNQLAYVVIGLLFMILTAAANEMGLLETTKKDLGIGHAAAENHHHAAMLDVDLHPA

SAWTLYAVATTIITPMMRHTIENTTANISLTAIANQAAILMGLDKGWPISKMDIGVPLLA

LGCYSQVNPLTLTAAVFMLVAHYAIIGPGLQAKATREAQKRTAAGIMKNPTVDGIVAIDL

DPVVYDAKFEKQLGQIMLLILCTSQILLMRTTWALCESITLATGPLTTLWEGSPGKFWNT

TIAVSMANIFRGSYLAGAGLAFSLMKSLGGGRRGTGAQGETLGEKWKRQLNQLSKSEFNT

YKRSGIIEVDRSEAKEGLKRGEPTKHAVSRGTAKLRWFVERNLVKPEGKVIDLGCGRGGW

SYYCAGLKKVTEVKGYTKGGPGHEEPIPMATYGWNLVKLYSGKDVFFTPPEKCDTLLCDI

GESSPNPTIEEGRTLRVLKMVEPWLRGNQFCIKILNPYMPSVVETLEQMQRKHGGMLVRN

PLSRNSTHEMYWVSCGTGNIVSAVNMTSRMLLNRFTMAHRKPTYERDVDLGAGTRHVAVE

PEVANLDIIGQRIENIKNGHKSTWHYDEDNPYKTWAYHGSYEVKPSGSASSMVNGVVRLL

TKPWDVIPMVTQIAMTDTTPFGQQRVFKEKVDTRTPKAKRGTAQIMEVTARWLWGFLSRN

KKPRICTREEFTRKVRSNAAIGAVFVDENQWNSAKEAVEDERFWDLVHRERELHKQGKCA

TCVYNMMGKREKKLGEFGKAKGSRAIWYMWLGARFLEFEALGFMNEDHWFSRENSLSGVE

GEGLHKLGYILRDISKIPGGNMYADDTAGWDTRITEDDLQNEAKITDIMEPEHALLATSI

FKLTYQNKVVRVQRPAKNGTVMDVISRRDQRGSGQVGTYGLNTFTNMEAQLIRQMESEGI

FSPSELETPNLAERVLDWLKKHGTERLKRMAISGDDCVVKPIDDRFATALTALNDMGKVR

KDIPQWEPSKGWNDWQQVPFCSHHFHQLIMKDGREIVVPCRNQDELVGRARVSQGAGWSL

RETACLGKSYAQMWQLMYFHRRDLRLAANAICSAVPVDWVPTSRTTWSIHAHHQWMTTED

MLSVWNRVWIEENPWMEDKTHVSSWEDVPYLGKREDRWCGSLIGLTARATWATNIQVAIN

QVRRLIGNENYLDFMTSMKRFKNESDPEGALW

**Dengue Virus-Type 2 (Polyprotein- 3448 AA)**

> NCBI accession no: **NP_056776.2** **(Ref_Seq)**

MNNQRKKAKNTPFNMLKRERNRVSTVQQLTKRFSLGMLQGRGPLKLFMALVAFLRFLTIP

PTAGILKRWGTIKKSKAINVLRGFRKEIGRMLNILNRRRRSAGMIIMLIPTVMAFHLTTR

NGEPHMIVSRQEKGKSLLFKTEDGVNMCTLMAMDLGELCEDTITYKCPLLRQNEPEDIDC

WCNSTSTWVTYGTCTTMGEHRREKRSVALVPHVGMGLETRTETWMSSEGAWKHVQRIETW

ILRHPGFTMMAAILAYTIGTTHFQRALIFILLTAVTPSMTMRCIGMSNRDFVEGVSGGSW

VDIVLEHGSCVTTMAKNKPTLDFELIKTEAKQPATLRKYCIEAKLTNTTTESRCPTQGEP

SLNEEQDKRFVCKHSMVDRGWGNGCGLFGKGGIVTCAMFRCKKNMEGKVVQPENLEYTIV

ITPHSGEEHAVGNDTGKHGKEIKITPQSSITEAELTGYGTVTMECSPRTGLDFNEMVLLQ

MENKAWLVHRQWFLDLPLPWLPGADTQGSNWIQKETLVTFKNPHAKKQDVVVLGSQEGAM

HTALTGATEIQMSSGNLLFTGHLKCRLRMDKLQLKGMSYSMCTGKFKVVKEIAETQHGTI

VIRVQYEGDGSPCKIPFEIMDLEKRHVLGRLITVNPIVTEKDSPVNIEAEPPFGDSYIII

GVEPGQLKLNWFKKGSSIGQMFETTMRGAKRMAILGDTAWDFGSLGGVFTSIGKALHQVF

GAIYGAAFSGVSWTMKILIGVIITWIGMNSRSTSLSVTLVLVGIVTLYLGVMVQADSGCV

VSWKNKELKCGSGIFITDNVHTWTEQYKFQPESPSKLASAIQKAHEEGICGIRSVTRLEN

LMWKQITPELNHILSENEVKLTIMTGDIKGIMQAGKRSLRPQPTELKYSWKTWGKAKMLS

TESHNQTFLIDGPETAECPNTNRAWNSLEVEDYGFGVFTTNIWLKLKEKQDVFCDSKLMS

AAIKDNRAVHADMGYWIESALNDTWKIEKASFIEVKNCHWPKSHTLWSNGVLESEMIIPK

NLAGPVSQHNYRPGYHTQITGPWHLGKLEMDFDFCDGTTVVVTEDCGNRGPSLRTTTASG

KLITEWCCRSCTLPPLRYRGEDGCWYGMEIRPLKEKEENLVNSLVTAGHGQVDNFSLGVL

GMALFLEEMLRTRVGTKHAILLVAVSFVTLITGNMSFRDLGRVMVMVGATMTDDIGMGVT

YLALLAAFKVRPTFAAGLLLRKLTSKELMMTTIGIVLLSQSTIPETILELTDALALGMMV

LKMVRNMEKYQLAVTIMAILCVPNAVILQNAWKVSCTILAVVSVSPLLLTSSQQKTDWIP

LALTIKGLNPTAIFLTTLSRTSKKRSWPLNEAIMAVGMVSILASSLLKNDIPMTGPLVAG

GLLTVCYVLTGRSADLELERAADVKWEDQAEISGSSPILSITISEDGSMSIKNEEEEQTL

TILIRTGLLVISGLFPVSIPITAAAWYLWEVKKQRAGVLWDVPSPPPMGKAELEDGAYRI

KQKGILGYSQIGAGVYKEGTFHTMWHVTRGAVLMHKGKRIEPSWADVKKDLISYGGGWKL

EGEWKEGEEVQVLALEPGKNPRAVQTKPGLFKTNAGTIGAVSLDFSPGTSGSPIIDKKGK

VVGLYGNGVVTRSGAYVSAIAQTEKSIEDNPEIEDDIFRKRRLTIMDLHPGAGKTKRYLP

AIVREAIKRGLRTLILAPTRVVAAEMEEALRGLPIRYQTPAIRAEHTGREIVDLMCHATF

TMRLLSPVRVPNYNLIIMDEAHFTDPASIAARGYISTRVEMGEAAGIFMTATPPGSRDPF

PQSNAPIIDEEREIPERSWNSGHEWVTDFKGKTVWFVPSIKAGNDIAACLRKNGKKVIQL

SRKTFDSEYVKTRTNDWDFVVTTDISEMGANFKAERVIDPRRCMKPVILTDGEERVILAG

PMPVTHSSAAQRRGRIGRNPKNENDQYIYMGEPLENDEDCAHWKEAKMLLDNINTPEGII

PSMFEPEREKVDAIDGEYRLRGEARKTFVDLMRRGDLPVWLAYRVAAEGINYADRRWCFD

GVKNNQILEENVEVEIWTKEGERKKLKPRWLDARIYSDPLALKEFKEFAAGRKSLTLNLI

TEMGRLPTFMTQKARDALDNLAVLHTAEAGGRAYNHALSELPETLETLLLLTLLATVTGG

IFLFLMSGRGIGKMTLGMCCIITASILLWYAQIQPHWIAASIILEFFLIVLLIPEPEKQR

TPQDNQLTYVVIAILTVVAATMANEMGFLEKTKKDLGLGSIATQQPESNILDIDLRPASA

WTLYAVATTFVTPMLRHSIENSSVNVSLTAIANQATVLMGLGKGWPLSKMDIGVPLLAIG

CYSQVNPITLTAALFLLVAHYAIIGPGLQAKATREAQKRAAAGIMKNPTVDGITVIDLDP

IPYDPKFEKQLGQVMLLVLCVTQVLMMRTTWALCEALTLATGPISTLWEGNPGRFWNTTI

AVSMANIFRGSYLAGAGLLFSIMKNTTNTRRGTGNIGETLGEKWKSRLNALGKSEFQIYK

KSGIQEVDRTLAKEGIKRGETDHHAVSRGSAKLRWFVERNMVTPEGKVVDLGCGRGGWSY

YCGGLKNVREVKGLTKGGPGHEEPIPMSTYGWNLVRLQSGVDVFFIPPEKCDTLLCDIGE

SSPNPTVEAGRTLRVLNLVENWLNNNTQFCIKVLNPYMPSVIEKMEALQRKYGGALVRNP

LSRNSTHEMYWVSNASGNIVSSVNMISRMLINRFTMRYKKATYEPDVDLGSGTRNIGIES

EIPNLDIIGKRIEKIKQEHETSWHYDQDHPYKTWAYHGSYETKQTGSASSMVNGVVRLLT

KPWDVVPMVTQMAMTDTTPFGQQRVFKEKVDTRTQEPKEGTKKLMKITAEWLWKELGKKK

TPRMCTREEFTRKVRSNAALGAIFTDENKWKSAREAVEDSRFWELVDKERNLHLEGKCET

CVYNMMGKREKKLGEFGKAKGSRAIWYMWLGARFLEFEALGFLNEDHWFSRENSLSGVEG

EGLHKLGYILRDVSKKEGGAMYADDTAGWDTRITLEDLKNEEMVTNHMEGEHKKLAEAIF

KLTYQNKVVRVQRPTPRGTVMDIISRRDQRGSGQVGTYGLNTFTNMEAQLIRQMEGEGVF

KSIQHLTITEEIAVQNWLARVGRERLSRMAISGDDCVVKPLDDRFASALTALNDMGKIRK

DIQQWEPSRGWNDWTQVPFCSHHFHELIMKDGRVLVVPCRNQDELIGRARISQGAGWSLR

ETACLGKSYAQMWSLMYFHRRDLRLAANAICSAVPSHWVPTSRTTWSIHAKHEWMTTEDM

LTVWNRVWIQENPWMEDKTPVESWEEIPYLGKREDQWCGSLIGLTSRATWAKNIQAAINQ

VRSLIGNEEYTDYMPSMKRFRREEEEAGVLW

**Dengue Virus-Type 3 (Polyprotein- 3447 AA)**

> NCBI accession no: **YP_001621843.1** **(Ref_Seq)**

MNNQRKKTGKPSINMLKRVRNRVSTGSQLAKRFSKGLLNGQGPMKLVMAFIAFLRFLAIP

PTAGVLARWGTFKKSGAIKVLKGFKKEISNMLSIINQRKKTSLCLMMILPAALAFHLTSR

DGEPRMIVGKNERGKSLLFKTASGINMCTLIAMDLGEMCDDTVTYKCPHITEVEPEDIDC

WCNLTSTWVTYGTCNQAGEHRRDKRSVALAPHVGMGLDTRTQTWMSAEGAWRQVEKVETW

ALRHPGFTILALFLAHYIGTSLTQKVVIFILLMLVTPSMTMRCVGVGNRDFVEGLSGATW

VDVVLEHGGCVTTMAKNKPTLDIELQKTEATQLATLRKLCIEGKITNITTDSRCPTQGEA

VLPEEQDQNYVCKHTYVDRGWGNGCGLFGKGSLVTCAKFQCLEPIEGKVVQYENLKYTVI

ITVHTGDQHQVGNETQGVTAEITPQASTTEAILPEYGTLGLECSPRTGLDFNEMILLTMK

NKAWMVHRQWFFDLPLPWASGATTETPTWNRKELLVTFKNAHAKKQEVVVLGSQEGAMHT

ALTGATEIQNSGGTSIFAGHLKCRLKMDKLELKGMSYAMCTNTFVLKKEVSETQHGTILI

KVEYKGEDAPCKIPFSTEDGQGKAHNGRLITANPVVTKKEEPVNIEAEPPFGESNIVIGI

GDNALKINWYKKGSSIGKMFEATERGARRMAILGDTAWDFGSVGGVLNSLGKMVHQIFGS

AYTALFSGVSWVMKIGIGVLLTWIGLNSKNTSMSFSCIAIGIITLYLGAVVQADMGCVIN

WKGKELKCGSGIFVTNEVHTWTEQYKFQADSPKRLATAIAGAWENGVCGIRSTTRMENLL

WKQIANELNYILWENNIKLTVVVGDTLGVLEQGKRTLTPQPMELKYSWKTWGKAKIVTAE

TQNSSFIIDGPNTPECPSASRAWNVWEVEDYGFGVFTTNIWLKLREVYTQLCDHRLMSAA

VKDERAVHADMGYWIESQKNGSWKLEKASLIEVKTCTWPKSHTLWTNGVLESDMIIPKSL

AGPISQHNYRPGYHTQTAGPWHLGKLELDFNYCEGTTVVITESCGTRGPSLRTTTVSGKL

IHEWCCRSCTLPPLRYMGEDGCWYGMEIRPISEKEENMVKSLVSAGSGKVDNFTMGVLCL

AILFEEVLRGKFGKKHMIAGVFFTFVLLLSGQITWRDMAHTLIMIGSNASDRMGMGVTYL

ALIATFKIQPFLALGFFLRKLTSRENLLLGVGLAMATTLQLPEDIEQMANGVALGLMALK

LITQFETYQLWTALVSLTCSNTIFTLTVAWRTATLILAGVSLLPVCQSSSMRKTDWLPMT

VAAMGVPPLPLFIFSLKDTLKRRSWPLNEGVMAVGLVSILASSLLRNDVPMAGPLVAGGL

LIACYVITGTSADLTVEKAPDVTWEEEAEQTGVSHNLMITVDDDGTMRIKDDETENILTV

LLKTALLIVSGIFPYSIPATLLVWHTWQKQTQRSGVLWDVPSPPETQKAELEEGVYRIKQ

QGIFGKTQVGVGVQKEGVFHTMWHVTRGAVLTHNGKRLEPNWASVKKDLISYGGGWRLSA

QWQKGEEVQVIAVEPGKNPKNFQTTPGTFQTTTGEIGAIALDFKPGTSGSPIINREGKVV

GLYGNGVVTKNGGYVSGIAQTNAEPDGPTPELEEEMFKKRNLTIMDLHPGSGKTRKYLPA

IVREAIKRRLRTLILAPTRVVAAEMEEALKGLPIRYQTTATKSEHTGREIVDLMCHATFT

MRLLSPVRVPNYNLIIMDEAHFTDPASIAARGYISTRVGMGEAAAIFMTATPPGTADAFP

QSNAPIQDEERDIPERSWNSGNEWITDFAGKTVWFVPSIKAGNDIANCLRKNGKKVIQLS

RKTFDTEYQKTKLNDWDFVVTTDISEMGANFKADRVIDPRRCLKPVILTDGPERVILAGP

MPVTAASAAQRRGRVGRNPQKENDQYIFTGQPLNNDEDHAHWTEAKMLLDNINTPEGIIP

ALFEPEREKSAAIDGEYRLKGESRKTFVELMRRGDLPVWLAHKVASEGIKYTDRKWCFDG

QRNNQILEENMDVEIWTKEGEKKKLRPRWLDARTYSDPLALKEFKDFAAGRKSIALDLVT

EIGRVPSHLAHRTRNALDNLVMLHTSEDGGRAYRHAVEELPETMETLLLLGLMILLTGGA

MLFLISGKGIGKTSIGLICVIASSGMLWMAEVPLQWIASAIVLEFFMMVLLIPEPEKQRT

PQDNQLAYVVIGILTLAATIAANEMGLLETTKRDLGMSKEPGVVSPTSYLDVDLHPASAW

TLYAVATTVITPMLRHTIENSTANVSLAAIANQAVVLMGLDKGWPISKMDLGVPLLALGC

YSQVNPLTLTAAVLLLITHYAIIGPGLQAKATREAQKRTAAGIMKNPTVDGIMTIDLDSV

IFDSKFEKQLGQVMLLVLCAVQLLLMRTSWALCEALTLATGPITTLWEGSPGKFWNTTIA

VSMANIFRGSYLAGAGLAFSIMKSVGTGKRGTGSQGETLGEKWKKKLNQLSRKEFDLYKK

SGITEVDRTEAKEGLKRGETTHHAVSRGSAKLQWFVERNMVVPEGRVIDLGCGRGGWSYY

CAGLKKVTEVRGYTKGGPGHEEPVPMSTYGWNIVKLMSGKDVFYLPPEKCDTLLCDIGES

SPSPTVEESRTIRVLKMVEPWLKNNQFCIKVLNPYMPTVIEHLERLQRKHGGMLVRNPLS

RNSTHEMYWISNGTGNIVSSVNMVSRLLLNRFTMTHRRPTIEKDVDLGAGTRHVNAEPET

PNMDVIGERIKRIKEEHNSTWHYDDENPYKTWAYHGSYEVKATGSASSMINGVVKLLTKP

WDVVPMVTQMAMTDTTPFGQQRVFKEKVDTRTPRPMPGTRKAMEITAEWLWRTLGRNKRP

RLCTREEFTKKVRTNAAMGAVFTEENQWDSAKAAVEDEEFWKLVDRERELHKLGKCGSCV

YNMMGKREKKLGEFGKAKGSRAIWYMWLGARYLEFEALGFLNEDHWFSRENSYSGVEGEG

LHKLGYILRDISKIPGGAMYADDTAGWDTRITEDDLHNEEKIIQQMDPEHRQLANAIFKL

TYQNKVVKVQRPTPTGTVMDIISRKDQRGSGQLGTYGLNTFTNMEAQLVRQMEGEGVLTK

ADLENPHLLEKKITQWLETKGVERLKRMAISGDDCVVKPIDDRFANALLALNDMGKVRKD

IPQWQPSKGWHDWQQVPFCSHHFHELIMKDGRKLVVPCRPQDELIGRARISQGAGWSLRE

TACLGKAYAQMWSLMYFHRRDLRLASNAICSAVPVHWVPTSRTTWSIHAHHQWMTTEDML

TVWNRVWIEENPWMEDKTPVTTWENVPYLGKREDQWCGSLIGLTSRATWAQNIPTAIQQV

RSLIGNEEFLDYMPSMKRFRKEEESEGAIW

**Dengue Virus-Type 4 (Polyprotein- 3444 AA)**

> NCBI accession no: **NP_073286.1** **(Ref_Seq)**

MNQRKKVVRPPFNMLKRERNRVSTPQGLVKRFSTGLFSGKGPLRMVLAFITFLRVLSIPP

TAGILKRWGQLKKNKAIKILIGFRKEIGRMLNILNGRKRSTITLLCLIPTVMAFSLSTRD

GEPLMIVAKHERGRPLLFKTTEGINKCTLIAMDLGEMCEDTVTYKCPLLVNTEPEDIDCW

CNLTSTWVMYGTCTQSGERRREKRSVALTPHSGMGLETRAETWMSSEGAWKHAQRVESWI

LRNPGFALLAGFMAYMIGQTGIQRTVFFVLMMLVAPSYGMRCVGVGNRDFVEGVSGGAWV

DLVLEHGGCVTTMAQGKPTLDFELTKTTAKEVALLRTYCIEASISNITTATRCPTQGEPY

LKEEQDQQYICRRDVVDRGWGNGCGLFGKGGVVTCAKFSCSGKITGNLVQIENLEYTVVV

TVHNGDTHAVGNDTSNHGVTAMITPRSPSVEVKLPDYGELTLDCEPRSGIDFNEMILMKM

KKKTWLVHKQWFLDLPLPWTAGADTSEVHWNYKERMVTFKVPHAKRQDVTVLGSQEGAMH

SALAGATEVDSGDGNHMFAGHLKCKVRMEKLRIKGMSYTMCSGKFSIDKEMAETQHGTTV

VKVKYEGAGAPCKVPIEIRDVNKEKVVGRIISSTPLAENTNSVTNIELEPPFGDSYIVIG

VGNSALTLHWFRKGSSIGKMFESTYRGAKRMAILGETAWDFGSVGGLFTSLGKAVHQVFG

SVYTTMFGGVSWMIRILIGFLVLWIGTNSRNTSMAMTCIAVGGITLFLGFTVQADMGCVA

SWSGKELKCGSGIFVVDNVHTWTEQYKFQPESPARLASAILNAHKDGVCGIRSTTRLENV

MWKQITNELNYVLWEGGHDLTVVAGDVKGVLTKGKRALTPPVSDLKYSWKTWGKAKIFTP

EARNSTFLIDGPDTSECPNERRAWNSLEVEDYGFGMFTTNIWMKFREGSSEVCDHRLMSA

AIKDQKAVHADMGYWIESSKNQTWQIEKASLIEVKTCLWPKTHTLWSNGVLESQMLIPKS

YAGPFSQHNYRQGYATQTVGPWHLGKLEIDFGECPGTTVTIQEDCDHRGPSLRTTTASGK

LVTQWCCRSCTMPPLRFLGEDGCWYGMEIRPLSEKEENMVKSQVTAGQGTSETFSMGLLC

LTLFVEECLRRRVTRKHMILVVVITLCAIILGGLTWMDLLRALIMLGDTMSGRIGGQIHL

AIMAVFKMSPGYVLGVFLRKLTSRETALMVIGMAMTTVLSIPHDLMELIDGISLGLILLK

IVTQFDNTQVGTLALSLTFIRSTMPLVMAWRTIMAVLFVVTLIPLCRTSCLQKQSHWVEI

TALILGAQALPVYLMTLMKGASRRSWPLNEGIMAVGLVSLLGSALLKNDVPLAGPMVAGG

LLLAAYVMSGSSADLSLEKAANVQWDEMADITGSSPIIEVKQDEDGSFSIRDVEETNMIT

LLVKLALITVSGLYPLAIPVTMTLWYMWQVKTQRSGALWDVPSPAATKKAALSEGVYRIM

QRGLFGKTQVGVGIHMEGVFHTMWHVTRGSVICHETGRLEPSWADVRNDMISYGGGWRLG

DKWDKEEDVQVLAIEPGKNPKHVQTKPGLFKTLTGEIGAVTLDFKPGTSGSPIINRKGKV

IGLYGNGVVTKSGDYVSAITQAERIGEPDYEVDEDIFRKKRLTIMDLHPGAGKTKRILPS

IVREALKRRLRTLILAPTRVVAAEMEEALRGLPIRYQTPAVKSEHTGREIVDLMCHATFT

TRLLSSTRVPNYNLIVMDEAHFTDPSSVAARGYISTRVEMGEAAAIFMTATPPGATDPFP

QSNSPIEDIEREIPERSWNTGFDWITDYQGKTVWFVPSIKAGNDIANCLRKSGKKVIQLS

RKTFDTEYPKTKLTDWDFVVTTDISEMGANFRAGRVIDPRRCLKPVILPDGPERVILAGP

IPVTPASAAQRRGRIGRNPAQEDDQYVFSGDPLKNDEDHAHWTEAKMLLDNIYTPEGIIP

TLFGPEREKTQAIDGEFRLRGEQRKTFVELMRRGDLPVWLSYKVASAGISYKDREWCFTG

ERNNQILEENMEVEIWTREGEKKKLRPRWLDARVYADPMALKDFKEFASGRKSITLDILT

EIASLPTYLSSRAKLALDNIVMLHTTERGGRAYQHALNELPESLETLMLVALLGAMTAGI

FLFFMQGKGIGKLSMGLITIAVASGLLWVAEIQPQWIAASIILEFFLMVLLIPEPEKQRT

PQDNQLIYVILTILTIIGLIAANEMGLIEKTKTDFGFYQVKTETTILDVDLRPASAWTLY

AVATTILTPMLRHTIENTSANLSLAAIANQAAVLMGLGKGWPLHRMDLGVPLLAMGCYSQ

VNPTTLTASLVMLLVHYAIIGPGLQAKATREAQKRTAAGIMKNPTVDGITVIDLEPISYD

PKFEKQLGQVMLLVLCAGQLLLMRTTWAFCEVLTLATGPILTLWEGNPGRFWNTTIAVST

ANIFRGSYLAGAGLAFSLIKNAQTPRRGTGTTGETLGEKWKRQLNSLDRKEFEEYKRSGI

LEVDRTEAKSALKDGSKIKHAVSRGSSKIRWIVERGMVKPKGKVVDLGCGRGGWSYYMAT

LKNVTEVKGYTKGGPGHEEPIPMATYGWNLVKLHSGVDVFYKPTEQVDTLLCDIGESSSN

PTIEEGRTLRVLKMVEPWLSSKPEFCIKVLNPYMPTVIEELEKLQRKHGGNLVRCPLSRN

STHEMYWVSGASGNIVSSVNTTSKMLLNRFTTRHRKPTYEKDVDLGAGTRSVSTETEKPD

MTIIGRRLQRLQEEHKETWHYDQENPYRTWAYHGSYEAPSTGSASSMVNGVVKLLTKPWD

VIPMVTQLAMTDTTPFGQQRVFKEKVDTRTPQPKPGTRMVMTTTANWLWALLGKKKNPRL

CTREEFISKVRSNAAIGAVFQEEQGWTSASEAVNDSRFWELVDKERALHQEGKCESCVYN

MMGKREKKLGEFGRAKGSRAIWYMWLGARFLEFEALGFLNEDHWFGRENSWSGVEGEGLH

RLGYILEEIDKKDGDLMYADDTAGWDTRITEDDLQNEELITEQMAPHHKILAKAIFKLTY

QNKVVKVLRPTPRGAVMDIISRKDQRGSGQVGTYGLNTFTNMEVQLIRQMEAEGVITQDD

MQNPKGLKERVEKWLKECGVDRLKRMAISGDDCVVKPLDERFGTSLLFLNDMGKVRKDIP

QWEPSKGWKNWQEVPFCSHHFHKIFMKDGRSLVVPCRNQDELIGRARISQGAGWSLRETA

CLGKAYAQMWSLMYFHRRDLRLASMAICSAVPTEWFPTSRTTWSIHAHHQWMTTEDMLKV

WNRVWIEDNPNMTDKTPVHSWEDIPYLGKREDLWCGSLIGLSSRATWAKNIHTAITQVRN

LIGKEEYVDYMPVMKRYSAPSESEGVL

**Japanese encephalitis virus (Polyprotein- 1198 AA)**

>UniprotKB: **P0DOH7** **(Ref_Seq)**

MTKKPGGPGKNRAINMLKRGLPRVFPLVGVKRVVMSLLDGRGPVRFVLALITFFKFTALA

PTKALLGRWKAVEKSVAMKHLTSFKRELGTLIDAVNKRGRKQNKRGGNEGSIMWLASLAV

VIACAGAMKLSNFQGKLLMTINNTDIADVIVIPTSKGENRCWVRAIDVGYMCEDTITYEC

PKLTMGNDPEDVDCWCDNQEVYVQYGRCTRTRHSKRSRRSVSVQTHGESSLVNKKEAWLD

STKATRYLMKTENWIIRNPGYAFLAAVLGWMLGSNNGQRVVFTILLLLVAPAYSFNCLGM

GNRDFIEGASGATWVDLVLEGDSCLTIMANDKPTLDVRMINIEASQLAEVRSYCYHASVT

DISTVARCPTTGEAHNEKRADSSYVCKQGFTDRGWGNGCGLFGKGSIDTCAKFSCTSKAI

GRTIQPENIKYEVGIFVHGTTTSENHGNYSAQVGASQAAKFTVTPNAPSITLKLGDYGEV

TLDCEPRSGLNTEAFYVMTVGSKSFLVHREWFHDLALPWTSPSSTAWRNRELLMEFEGAH

ATKQSVVALGSQEGGLHQALAGAIVVEYSSSVKLTSGHLKCRLKMDKLALKGTTYGMCTE

KFSFAKNPVDTGHGTVVIELSYSGSDGPCKIPIVSVASLNDMTPVGRLVTVNPFVATSSA

NSKVLVEMEPPFGDSYIVVGRGDKQINHHWHKAGSTLGKAFSTTLKGAQRLAALGDTAWD

FGSIGGVFNSIGRAVHQVFGGAFRTLFGGMSWITQGLMGALLLWMGVNARDRSIALAFLA

TGGVLVFLATNVHADTGCAIDITRKEMRCGSGIFVHNDVEAWVDRYKYLPETPRSLAKIV

HKAHKEGVCGVRSVTRLEHQMWEAVRDELNVLLKENAVDLSVVVNKPVGRYRSAPKRLSM

TQEKFEMGWKAWGKSILFAPELANSTFVVDGPETKECPDEHRAWNSMQIEDFGFGITSTR

VWLKIREESTDECDGAIIGTAVKGHVAVHSDLSYWIESRYNDTWKLERAVFGEVKSCTWP

ETHTLWGDDVEESELIIPHTIAGPKSKHNRREGYKTQNQGPWDENGIVLDFDYCPGTKVT

ITEDCSKRGPSVRTTTDSGKLITDWCCRSCSLPPLRFRTENGCWYGMEIRPVMHDETTLV

RSQVDAFKGEMVDPFSAGPSGDVSGHPGSPSQEVDGQIDHSCGFGGPTCADAWGYHLH

**All the representative sequences of WNV from all variants/genotypes**

>AYV87261.1 polyprotein [West Nile virus]

MSKKPGGPGKSRAVNMLKRGMPRVLSLIGLKRAMLSLIDGKGPIRFVLALLAFFRFTAIAPTRAVLDRWRGVNKQTAMKHLLSFKKELGTLTSAINRRSSKQKKRGGKTGIAVMIGLIASVGAVTLSNFQGKVMMTVNATDVTDVITIPTAAGKNLCIVRAMDVGYMCDDTITYECPVLSAGNDPEDIDCWCTKSAVYVRYGRCTKTRHSRRSRRSLTVQTHGESTLANKKGAWMDSTKATRYLVKTESWILRNPGYALVAAVIGWMLGSNTMQRVVFVVLLLLVAPAYSFNCLGMSNRDFLEGVSGATWVDLVLEGDSCVTIMSKDKPTIDVKMMNMEAANLAEVRSYCYLATVSDLSTKAACPTMGEAHNDKRADPAFVCRQGVVDRGWGNGCGLFGKGSIDTCAKFACSTKAIGRTILKENIKYEVAIFVHGPTTVESHGNYSTQVGATQAGRFSITPAAPSYTLKLGEYGEVTVDCEPRSGIDTNAYYVMTVGTKTFLVHREWFMDLNLPWSSAGSTVWRNRETLMEFEEPHATKQSVIALGSQEGALHQALAGAIPVEFSSNTVKLTSGHLKCRVKMEKLQLKGTTYGVCSKAFKFLGTPADTGHGTVVLELQYTGTDGPCKVPISSVASLNDLTPVGRLVTVNPFVSVATANAKVLIELEPPFGDSYIVVGRGEQQINHHWHKSGSSIGKAFTTTLKGAQRLAALGDTAWDFGSVGGVFTSVGKAVHQVFGGAFRSLFGGMSWITQGLLGALLLWMGINARDRSIALTFLAVGGVLLFLSVNVHADTGCAIDISRQELRCGSGVFIHNDVEAWMDRYKYYPETPQGLAKIIQKAHKEGVCGLRSVSRLEHQMWEAVKDELNTLLKENGVDLSVVVEKQEGMYKSAPKRLTATTEKLEIGWKAWGKSILFAPELANNTFVVDGPETKECPTQNRAWNSLEVEDFGFGLTSTRMFLKVRESNTTECDSKIIGTAVKNNLAIHSDLSYWIESRLNDTWKLERAVLGEVKSCTWPETHTLWGDGILESDLIIPVTLAGPRSNHNRRPGYKTQNQGPWDEGRVEIDFDYCPGTTVTLSESCGHRGPATRTTTESGKLITDWCCRSCTLPPLRYQTDSGCWYGMEIRPQRHDEKTLVQSQVNAYNADMIDPFQLGLLVVFLATQEVLRKRWTAKISMPAILIALLVLVFGGITYTDVLRYVILVGAAFAESNSGGDVVHLALMATFKIQPVFMVASFLKARWTNQENILLMLAAVFFQMAYHDARQILLWEIPDVLNSLAVAWMILRAITFTTTSNVVVPLLALLTPGLRCLNLDVYRILLLMVGIGSLIREKRSAAAKKKGASLLCLALASTGLFNPMILAAGLIACDPNRKRGWPATEVMTAVGLMFAIVGGLAELDIDSMAIPMTIAGLMFAAFVISGKSTDMWIERTADISWESDAEITGSSERVDVRLDDDGNFQLMNDPGAPWKIWMLRMVCLAVSAYTPWAILPSVVGFWITLQYTKRGGVLWDTPSPKEYKKGDTTTGVYRIMTRGLLGSYQAGAGVMVEGVFHTLWHTTKGAALMSGEGRLDPYWGSVKEDRLCYGGPWKLQHKWNGQDEVQMIVVEPGKNVKNVQTKPGVFKTPEGEIGAVTLDFPTGTSGSPIVDKNGDVIGLYGNGVIMPNGSYISAIVQGERMDEPIPAGFEPEMLRKKQITVLDLHPGAGKTRRILPQIIKEAINRRLRTAVLAPTRVVAAEMAEALRGLPIRYQTSAVPREHNGNEIVDVMCHATLTHRLMSPHRVPNYNLFVMDEAHFTDPASIAARGYISTKVELGEAAAIFMTATPPGTSDPFPESNSPISDLQTEIPDRAWNSGYEWITEYTGKTVWFVPSVKMGNEIALCLQRAGKKVVQLNRKSYETEYPKCKNDDWDFVITTDISEMGANFKASRVIDSRKSVKPTIITEGEGRVILGEPSAVTAASAAQRRGRIGRNPSQVGDEYCYGGHTNEDDSNFAHWTEARIMLDNINMPNGLIAQFYQPEREKVYTMDGEYRLRGEERKNFLELLRTADLPVWLAYKVAAAGVSYHDRRWCFDGPRTNTILEDNNEVEVITKLGERKILRPRWIDARVYSDHQALKAFKDFASGKRSQIGLIEVLGKMPEHFMGKTWEALDTMYVVATAEKGGRAHRMALEELPDALQTIALIALLSVMTMGVFFLLMQRKGIGKIGLGGAVLGVATFFCWMAEVPGTKIAGMLLLSLLLMIVLIPEPEKQRSQTDNQLAVFLICVMTLVSAVAANEMGWLDKTKSDISSLFGQRIEVKENFSMGEFLLDLRPATAWSLYAVTTAVLTPLLKHLITSDYINTSLTSINVQASALFTLARGFPFVDVGVSALLLAAGCWGQVTLTVTVTAATLLFCHYAYMVPGWQAEAMRSAQRRTAAGIMKNAVVDGIVATDVPELERTTPIMQKKVGQIMLILVSLAAVVVNPSVKTVREAGILITAAAVTLWENGASSVWNATTAIGLCHIMRGGWLSCLSITWTLIKNMEKPGLKRGGAKGRTLGEVWKERLNQMTKEEFTRYRKEAIIEVDRSAAKHARKEGNVTGGHPVSRGTAKLRWLVERRFLEPVGKVIDLGCGRGGWCYYMATQKRVQEVRGYTKGGPGHEEPQLVQSYGWNIVTMKSGVDVFYRPSECCDTLLCDIGESSSSAEVEEHRTIRVLEMVEDWLHRGPREFCVKVLCPYMPKVIEKMELLQRRYGGGLVRNPLSRNSTHEMYWVSRASGNVVHSVNMTSQVLLGRMEKRTWKGPQYEEDVNLGSGTRAVGKPLLNSDTSKIKNRIERLRREYSSTWHHDENHPYRTWNYHGSYDVKPTGSASSLVNGVVRLLSKPWDTITNVTTMAMTDTTPFGQQRVFKEKVDTKAPEPPEGVKYVLNETTNWLWAFLAREKRPRMCSREEFIRKVNSNAALGAMFEEQNQWRSAREAVEDPKFWEMVDEEREAHLRGECHTCIYNMMGKREKKPGEFGKAKGSRAIWFMWLGARFLEFEALGFLNEDHWLGRKNSGGGVEGLGLQKLGYILREVGTRPGGKIYADDTAGWDTRITRADLENEAKVLELLDGEHRRLARAIIELTYRHKVVKVMRPAADGRTVMDVISREDQRGSGQVVTYALNTFTNLAVQLVRMMEGEGVIGPDDVEKLTKGKGPKVRTWLFENGEERLSRMAVSGDDCVVKPLDDRFATSLHFLNAMSKVRKDIQEWKPSTGWYDWQQVPFCSNHFTELIMKDGRTLVVPCRGQDELVGRARISPGAGWNVRDTACLAKSYAQMWLLLYFHRRDLRLMANAICSAVPVNWVPTGRTTWSIHAGGEWMTTEDMLEVWNRVWIEENEWMEDKTPVEKWSDVPYSGKREDIWCGSLIGTRARATWAENIQVAINQVRAIIGDEKYVDYMSSLKRYEDTTLVEDTVL

>ANW69361.1 genome polyprotein [West Nile virus]

MSKKPGGPGKSRAVNMLKRGMPRVLSLIGLKRAMLSLIDGKGPIRFVLALLAFFRFTAIAPTRAVLDRWRGVNKQTAMKHLLSFKKELGTLTSAINRRSSKQKKRGGKTGIAVMIGLIASVGAVTLSNFQGKVMMTVNATDVTDVITIPTAAGKNLCIVRAMDVGYMCDDTITYECPVLSAGNDPEDIDCWCTKSAVYVRYGRCTKTRHSRRSRRSLTVQTHGESTLANKKGAWMDSTKATRYLVKTESWILRNPGYALVAAVIGWMLGSNTMQRVVFVVLLLLVAPAYSFNCLGMSNRDFLEGVSGATWVDLVLEGDSCVTIMSKDKPTIDVKMMNMEAANLAEVRSYCYLATVSDLSTKAACPTMGEAHNDKRADPAFVCRQGVVDRGWGNGCGLFGKGSIDTCAKFACSTKAIGRTILKENIKYEVAIFVHGPTTVESHGNYSTQVGATQAGRFSITPAAPSYTLKLGEYGEVTVDCEPRSGIDTNAYYVMTVGTKTFLVHREWFMDLNLPWSSAGSTVWRNRETLMEFEEPHATKQSVIALGSQEGALHQALAGAIPVEFSSNTVKLTSGHLKCRVKMEKLQLKGTTYGVCSKAFKFLGTPADTGHGTVVLELQYTGTDGPCKVPISSVASLNDLTPVGRLVTVNPFVSVATANAKVLIELEPPFGDSYIVVGRGEQQINHHWHKSGSSIGKAFTTTLKGAQRLAALGDTAWDFGSVGGVFTSVGKAVHQVFGGAFRSLFGGMSWITQGLLGALLLWMGINARDRSIALTFLAVGGVLLFLSVNVHADTGCAIDISRQELRCGSGVFIHNDVEAWMDRYKYYPETPQGLAKIIQKAHKEGVCGLRSVSRLEHQMWEAVKDELNTLLKENGVDLSVVVEKQEGMYKSAPKRLTATTEKLEIGWKAWGKSILFAPELANNTFVVDGPETKECPTQNRAWNSLEVEDFGFGLTSTRMFLKVRESNTTECDSKIIGTAVKNNLAIHSDLSYWIESRLNDTWKLERAVLGEVKSCTWPETHTLWGDGILESDLIIPVTLAGPRSNHNRRPGYKTQNQGPWDEGRVEIDFDYCPGTTVTLSESCGHRGPATRTTTESGKLITDWCCRSCTLPPLRYQTDSGCWYGMEIRPQRHDEKTLVQSQVNAYNADMIDPFQLGLLVVFLATQEVLRKRWTAKISMPAILIALLVLVFGGITYTDVLRYVILVGAAFAESNSGGDVVHLALMATFKIQPVFMVASFLKARWTNQENILLMLAAVFFQMAYHDARQILLWEIPDVLNSLAVAWMILRAITFTTTSNVVVPLLALLTPGLRCLNLDVYRILLLMVGIGSLIREKRSAAAKKKGASLLCLALASTGLFNPMILAAGLIACDPNRKRGWPATEVMTAVGLMFAIVGGLAELDIDSMAIPMTIAGLMFAAFVISGKSTDMWIERTADISWESDAEITGSSERVDVRLDDDGNFQLMNDPGAPWKIWMLRMVCLAISAYTPWAILPSVVGFWITLQYTKRGGVLWDTPSPKEYKKGDTTTGVYRIMTRGLLGSYQAGAGVMVEGVFHTLWHTTKGAALMSGEGRLDPYWGSVKEDRLCYGGPWKLQHKWNGQDEVQMIVVEPGKNVKNVQTKPGVFKTPEGEIGAVTLDFPTGTSGSPIVDKNGDVIGLYGNGVIMPNGSYISAIVQGERMDEPIPAGFEPEMLRKKQITVLDLHPGAGKTRRILPQIIKEAINRRLRTAVLAPTRVVAAEMAEALRGLPIRYQTSAVPREHNGNEIVDVMCHATLTHRLMSPHRVPNYNLFVMDEAHFTDPASIAARGYISTKVELGEAAAIFMTATPPGTSDPFPESNSPISDLQTEIPDRAWNSGYEWITEYTGKTVWFVPSVKMGNEIALCLQRAGKKVVQLNRKSYETEYPKCKNDDWDFVITTDISEMGANFKASRVIDSRKSVKPTIITEGEGRVVLGEPSAVTAASAAQRRGRIGRNPSQVGDEYCYGGHTNEDDSNFAHWTEARIMLDNINMPNGLIAQFYQPEREKVYTMDGEYRLRGEERKNFLELLRTADLPVWLAYKVAAAGVSYHDRRWCFDGPRTNTILEDNNEVEVITKLGERKILRPRWIDARVYSDHQALKAFKDFASGKRSQIGLIEVLGKMPEHFMGKTWEALDTMYVVATAEKGGRAHRMALEELPDALQTIALIALLSVMTMGVFFLLMQRKGIGKIGLGGAVLGVATFFCWMAEVPGTKIAGMLLLSLLLMIVLIPEPEKQRSQTDNQLAVFLICVMTLVSAVAANEMGWLDKTKSDISSLFGQRIEVKENFSMGEFLLDLRPATAWSLYAVTTAVLTPLLKHLITSDYINTSLTSINVQASALFTLARGFPFVDVGVSALLLAAGCWGQVTLTVTVTAATLLFCHYAYMVPGWQAEAMRSAQRRTAAGIMKNAVVDGIVATDVPELERTTPIMQKKVGQIMLILVSLAAVVVNPSVKTVREAGILITAAAVTLWENGASSVWNATTAIGLCHIMRGGWLSCLSITWTLIKNMEKPGLKRGGAKGRTLGEVWKERLNQMTKEEFTRYRKEAIIEVDRSAAKHARKEGNVTGGHPVSRGTAKLRWLVERRFLEPVGKVIDLGCGRGGWCYYMATQKRVQEVRGYTKGGPGHEEPQLVQSYGWNIVTMKSGVDVFYRPSECCDTLLCDIGESSSSAEVEEHRTIRVLEMVEDWLHRGPREFCVKVLCPYMPKVIEKMELLQRRYGGGLVRNPLSRNSTHEMYWVSRASGNVVHSVNMTSQVLLGRMEKRTWKGPQYEEDVNLGSGTRAVGKPLLNSDTSKIKNRIERLRREYSSTWHHDENHPYRTWNYHGSYDVKPTGSASSLVNGVVRLLSKPWDTITNVTTMAMTDTTPFGQQRVFKEKVDTKAPEPPEGVKYVLNETTNWLWAFLAREKRPRMCSREEFIRKVNSNAALGAMFEEQNQWRSAREAVEDPKFWEMVDEEREAHLRGECHTCIYNMMGKREKKPGEFGKAKGSRAIWFMWLGARFLEFEALGFLNEDHWLGRKNSGGGVEGLGLQKLGYILREVGTRPGGKIYADDTAGWDTRITRADLENEAKVLELLDGEHRRLARAIIELTYRHKVVKVMRPAADGRTVMDVISREDQRGSGQVVTYALNTFTNLAVQLVRMMEGEGVIGPDDVEKLTKGKGPKVRTWLFENGEERLSRMAVSGDDCVVKPLDDRFATSLHFLNAMSKVRKDIQEWKPSTGWYDWQQVPFCSNHFTELIMKDGRTLVVPCRGQDELVGRARISPGAGWNVRDTACLAKSYAQMWLLLYFHRRDLRLMANAICSAVPVNWVPTGRTTWSIHAGGEWMTTEDMLEVWNRVWIEENEWMEDKTPVEKWSDVPYSGKREDIWCGSLIGTRARATWAENIQVAINQVRAIIGDEKYVDYMSSLKRYEDTTLVEDTVL

>ADL27942.1 polyprotein [West Nile virus]

MSKKPGGPGKSRAVNMLKRGMPRVLSLIGLKRAMLSLIDGKGPIRFVLALLAFFRFTAIAPTRAVLDRWRGVNKQTAMKHLLSFKKELGTLTSAINRRSSKQKKRGGKTGIAVMIGLIASVGAVTLSNFQGKVMMTVNATDVTDVITIPTAAGKNLCIVRAMDVGYMCDDTITYECPVLSAGNDPEDIDCWCTKSAVYVRYGRCTKTRHSRRSRRSLTVQTHGESTLANKKGAWMDSTKATRYLVKTESWILRNPGYALVAAVIGWMLGSNTMQRVVFVVLLLLVAPAYSFNCLGMSNRDFLEGVSGATWVDLVLEGDSCVTIMSKDKPTIDVKMMNMEAANLAEVRSYCYLATVSDLSTKAACPTMGEAHNDKRADPAFVCRQGVVDRGWGNGCGLFGKGSIDTCAKFACSTKAIGRTILKENIKYEVAIFVHGPTTVESHGNYSTQVGATQAGRFSITPAAPSYTLKLGEYGEVTVDCEPRSGIDTNAYYVMTVGTKTFLVHREWFMDLNLPWSSAGSTVWRNRETLMEFEEPHATKQSVIALGSQEGALHQALAGAIPVEFSSNTVKLTSGHLKCRVKMEKLQLKGTTYGVCSKAFKFLGTPADTGHGTVVLELQYTGTDGPCKVPISSVASLNDLTPVGRLVTVNPFVSVATANAKVLIELEPPFGDSYIVVGRGEQQINHHWHKSGSSIGKAFTTTLKGAQRLAALGDTAWDFGSVGGVFTSVGKAVHQVFGGAFRSLFGGMSWITQGLLGALLLWMGINARDRSIALTFLAVGGVLLFLSVNVHADTGCAIDISRQELRCGSGVFIHNDVEAWMDRYKYYPETPQGLAKIIQKAHKEGVCGLRSVSRLEHQMWEAVKDELNTLLKENGVDLSVVVEKQEGMYKSAPKRLTATTEKLEIGWKAWGKSILFAPELANNTFVVDGPETKECPTQNRAWNSLEVEDFGFGLTSTRMFLKVRESNTTECDSKIIGTAVKNNLAIHSDLSYWIESRLNDTWKLERAVLGEVKSCTWPETHTLWGDGILESDLIIPVTLAGPRSNHNRRPGYKTQNQGPWDEGRVEIDFDYCPGTTVTLSESCGHRGPATRTTTESGKLITDWCCRSCTLPPLRYQTDSGCWYGMEIRPQRHDEKTLVQSQVNAYNADMIDPFQLGLLVVFLATQEVLRKRWTAKISMPAILIALLVLVFGGITYTDVLRYVILVGAAFAESNSGGDVVHLALMATFKIQPVFMVASFLKARWTNQENILLMLAAVFFQMAYHDARQILLWEIPDVLNSLAVAWMILRAITFTTTSNVVVPLLALLTPGLRCLNLDVYRILLLMVGIGSLIREKRSAAAKKKGASLLCLALASTGLFNPMILAAGLIACDPNRKRGWPATEVMTAVGLMFAIVGGLAELDIDSMAIPMTIAGLMFAAFVISGKSTDMWIERTADISWESDAEITGSSERVDVRLDDDGNFQLMNDPGAPWKIWMLRMVCLAISAYTPWAILPSVVGFWITLQYTKRGGVLWDTPSPKEYKKGDTTTGVYRIMTRGLLGSYQAGAGVMVEGVFHTLWHTTKGAALMSGEGRLDPYWGSVKEDRLCYGGPWKLQHKWNGQDEVQMIVVEPGKNVKNVQTKPGVFKTPEGEIGAVTLDFPTGTSGSPIVDKNGDVIGLYGNGVIMPNGSYISAIVQGERMDEPIPAGFEPEMLRKKQITVLDLHPGAGKTRRILPQIIKEAINRRLRTAVLAPTRVVAAEMAEALRGLPIRYQTSAVPREHNGNEIVDVMCHATLTHRLMSPHRVPNYNLFVMDEAHFTDPASIAARGYISTKVELGEAAAIFMTATPPGTSDPFPESNSPISDLQTEIPDRAWNSGYEWITEYTGKTVWFVPSVKMGNEIALCLQRAGKKVVQLNRKSYETEYPKCKNDDWDFVITTDISEMGANFKASRVIDSRKSVKPTIITEGEGRVILGEPSAVTAASAAQRRGRIGRNPSQVGDEYCYGGHTNEDDSNFAHWTEARIMLDNINMPNGLIAQFYQPEREKVYTMDGEYRLRGEERKNFLELLRTADLPVWLAYKVAAAGVSYHDRRWCFDGPRTNTILEDNNEVEVITKLGERKILRPRWIDARVYSDHQALKAFKDFASGKRSQIGLIEVLGKMPEHFMGKTWEALDTMYVVATAEKGGRAHRMALEELPDALQTIALIALLSVMTMGVFFLLMQRKGIGKIGLGGAVLGVATFFCWMAEVPGTKIAGMLLLSLLLMIVLIPEPEKQRSQTDNQLAVFLICVMTLVSAVAANEMGWLDKTKSDISSLFGQRIEVKENFSMGEFLLDLRPATAWSLYAVTTAVLTPLLKHLITSDYINTSLTSINVQASALFTLARGFPFVDVGVSALLLAAGCWGQVTLTVTVTAATLLFCHYAYMVPGWQAEAMRSAQRRTAAGIMKNAVVDGIVATDVPELERTTPIMQKKVGQIMLILVSLAAVVVNPSVKTVREAGILITAAAVTLWENGASSVWNATTAIGLCHIMRGGWLSCLSITWTLVKNMEKPGLKRGGAKGRTLGEVWKERLNQMTKEEFTRYRKEAIIEVDRSAAKHARKEGNVTGGHPVSRGTAKLRWLVERRFLEPVGKVIDLGCGRGGWCYYMATQKRVQEVRGYTKGGPGHEEPQLVQSYGWNIVTMKSGVDVFYRPSECCDTLLCDIGESSSSAEVEEHRTIRVLEMVEDWLHRGPREFCVKVLCPYMPKVIEKMELLQRRYGGGLVRNPLSRNSTHEMYWVSRASGNVVHSVNMTSQVLLGRMEKRTWKGPQYEEDVNLGSGTRAVGKPLLNSDTSKIKNRIERLRREYSSTWHHDENHPYRTWNYHGSYDVKPTGSASSLVNGVVRLLSKPWDTITNVTTMAMTDTTPFGQQRVFKEKVDTKAPEPPEGVKYVLNETTNWLWAFLAREKRPRMCSREEFIRKVNSNAALGAMFEEQNQWRSAREAVEDPKFWEMVDEEREAHLRGECHTCIYNMMGKREKKPGEFGKAKGSRAIWFMWLGARFLEFEALGFLNEDHWLGRKNSGGGVEGLGLQKLGYILREVGTRPGGKIYADDTAGWDTRITRADLENEAKVLELLDGEHRRLARAIIELTYRHKVVKVMRPAADGRTVMDVISREDQRGSGQVVTYALNTFTNLAVQLVRMMEGEGVIGPDDVEKLTKGKGPKVRTWLFENGEERLSRMAVSGDDCVVKPLDDRFATSLHFLNAMSKVRKDIQEWKPSTGWYDWQQVPFCSNHFTELIMKDGRTLVVPCRGQDELVGRARISPGAGWNVRDTACLAKSYAQMWLLLYFHRRDLRLMANAICSAVPVNWVPTGRTTWSIHAGGEWMTTEDMLEVWNRVWIEENEWMEDKTPVEKWSDVPYSGKREDIWCGSLIGTRARATWAENIQVAINQVRAIIGDEKYVDYMSSLKRYEDTTLVEDTVL

>ABA54580.1 polyprotein precursor [West Nile virus]

MSKKPGGPGKSRAVNMLKRGMPRVLSLIGLKRAMLSLIDGKGPIRFVLALLAFFRFTAIAPTRAVLDRWRGVNKQTAMKHLLSFKKELGTLTSAINRRSSKQKKRGGKTGIAVMIGLIASVGAVTLSNFQGKVMMTVNATDVTDVITIPTAAGKNLCIVRAMDVGYMCDDTITYECPVLSAGNDPEDIDCWCTKSAVYVRYGRCTKTRHSRRSRRSLTVQTHGESTLANKKGAWMDSTKATRYLVKTESWILRNPGYALVAAVIGWMLGSNTMQRVVFVVLLLLVAPAYSFNCLGMSNRDFLEGVSGATWVDLVLEGDSCVTIMSKDKPTIDVKMMNMEAANLAEVRSYCYLATVSDLSTKAACPTMGEAHNDKRADPAFVCRQGVVDRGWGNGCGLFGKGSIDTCAKFACSTKAIGRTILKENIKYEVAIFVHGPTTVESHGNYSTQAGATQAGRFSITPAAPSYTLKLGEYGEVTVDCEPRSGIDTNAYYVMTVGTKTFLVHREWFMDLNLPWSSAGSTVWRNRETLMEFEEPHATKQSVIALGSQEGALHQALAGAIPVEFSSNTVKLTSGHLKCRVKMEKLQLKGTTYGVCSKAFKFLGTPADTGHGTVVLELQYTGTDGPCKVPISSVASLNDLTPVGRLVTVNPFVSVATANAKVLIELEPPFGDSYIVVGRGEQQINHHWHKSGSSIGKAFTTTLKGAQRLAALGDTAWDFGSVGGVFTSVGKAVHQVFGGAFRSLFGGMSWITQGLLGALLLWMGINARDRSIALTFLAVGGVLLFLSVNVHADTGCAIDISRQELRCGSGVFIHNDVEAWMDRYKYYPETPQGLAKIIQKAHKEGVCGLRSVSRLEHQMWEAVKDELNTLLKENGVDLSVVVEKQEGMYKSAPKRLTATTEKLEIGWKAWGKSILFAPELANNTFVVDGPETKECPTQNRAWNSLEVEDFGFGLTSTRMFLKVRESNTTECDSKIIGTAVKNNLAIHSDLSYWIESRLNDTWKLERAVLGEVKSCTWPETHTLWGDGILESDLIIPVTLAGPRSNHNRRPGYKTQNQGPWDEGRVEIDFDYCPGTTVTLSESCGHRGPATRTTTESGKLITDWCCRSCTLPPLRYQTDSGCWYGMEIRPQRHDEKTLVQSQVNAYNADMIDPFQLGLLVVFLATQEVLRKRWTAKISMPAILIALLVLVFGGITYTDVLRYVILVGAAFAESNSGGDVVHLALMATFKIQPVFMVASFLKARWTNQENILLMLAAVFFQMAYHDARQILLWEIPDVLNSLAVAWMILRAITFTTTSNVVVPLLALLTPGLRCLNLDVYRILLLMVGIGSLIREKRSAAAKKKGASLLCLALASTGLFNPMILAAGLIACDPNRKRGWPATEVMTAVGLMFAIVGGLAELDIDSMAIPMTIAGLMFAAFVISGKSTDMWIERTADISWESDAEITGSSERVDVRLDDDGNFQLMNDPGAPWKIWMLRMVCLAISAYTPWAILPSVVGFWITLQYTKRGGVLWDTPSPKEYKKGDTTTGVYRIMTRGLLGSYQAGAGVMVEGVFHTLWHTTKGAALMSGEGRLDPYWGSVKEDRLCYGGPWKLQHKWNGQDEVQMIVVEPGKNVKNVQTKPGVFKTPEGEIGAVTLDFPTGTSGSPIVDKNGDVIGLYGNGVIMPNGSYISAIVQGERMDEPIPAGFEPEMLRKKQITVLDLHPGAGKTRRILPQIIKEAINRRLRTAVLAPTRVVAAEMAEALRGLPIRYQTSAVPREHNGNEIVDVMCHATLTHRLMSPHRVPNYNLFVMDEAHFTDPASIAARGYISTKVELGEAAAIFMTATPPGTSDPFPESNSPISDLQTEIPDRAWNSGYEWITEYTGKTVWFVPSVKMGNEIALCLQRAGKKVVQLNRKSYETEYPKCKNDDWDFVITTDISEMGANFKASRVIDSRKSVKPTIITEGEGRVILGEPSAVTAASAAQRRGRIGRNPSQVGDEYCYGGHTNEDDSNFAHWTEARIMLDNINMPNGLIAQFYQPEREKVYTMDGEYRLRGEERKNFLELLRTADLPVWLAYKVAAAGVSYHDRRWCFDGPRTNTILEDNNEVEVITKLGERKILRPRWIDARVYSDHQALKAFKDFASGKRSQIGLIEVLGKMPEHFMGKTWEALDTMYVVATAEKGGRAHRMALEELPDALQTIALIALLSVMTMGVFFLLMQRKGIGKIGLGGAVLGVATFFCWMAEVPGTKIAGMLLLSLLLMIVLIPEPEKQRSQTDNQLAVFLICVMTLVSAVAANEMGWLDKTKSDISSLFGQRIEVKENFSMGEFLLDLRPATAWSLYAVTTAVLTPLLKHLITSDYINTSLTSINVQASALFTLARGFPFVDVGVSALLLAAGCWGQVTLTVTVTAATLLFCHYAYMVPGWQAEAMRSAQRRTAAGIMKNAVVDGIVATDVPELERTTPIMQKKVGQIMLILVSLAAVVVNPSVKTVREAGILITAAAVTLWENGASSVWNATTAIGLCHIMRGGWLSCLSITWTLIKNMEKPGLKRGGAKGRTLGEVWKERLNQMTKEEFTRYRKEAIIEVDRSAAKHARKEGNVTGGHPVSRGTAKLRWLVERRFLEPVGKVIDLGCGRGGWCYYMATQKRVQEVRGYTKGGPGHEEPQLVQSYGWNIVTMKSGVDVFYRPSECCDTLLCDIGESSSSAEVEEHRTIRVLEMVEDWLHRGPREFCVKVLCPYMPKVIEKMELLQRRYGGGLVRNPLSRNSTHEMYWVSRASGNVVHSVNMTSQVLLGRMEKRTWKGPQYEEDVNLGSGTRAVGKPLLNSDTSKIKNRIERLRREYSSTWHHDENHPYRTWNYHGSYDVKPTGSASSLVNGVVRLLSKPWDTITNVTTMAMTDTTPFGQQRVFKEKVDTKAPEPPEGVKYVLNETTNWLWAFLAREKRPRMCSREEFIRKVNSNAALGAMFEEQNQWRSAREAVEDPKFWEMVDEEREAHLRGECHTCIYNMMGKREKKPGEFGKAKGSRAIWFMWLGARFLEFEALGFLNEDHWLGRKNSGGGVEGLGLQKLGYILREVGTRPGGKIYADDTAGWDTRITRADLENEAKVLELLDGEHRRLARAIIELTYRHKVVKVMRPAADGRTVMDVISREDQRGSGQVVTYALNTFTNLAVQLVRMMEGEGVIGPDDVEKLTKGKGPKVRTWLFENGEERLSRMAVSGDDCVVKPLDDRFATSLHFLNAMSKVRKDIQEWKPSTGWYDWQQVPFCSNHFTELIMKDGRTLVVPCRGQDELVGRARISPGAGWNVRDTACLAKSYAQMWLLLYFHRRDLRLMANAICSAVPVNWVPTGRTTWSIHAGGEWMTTEDMLEVWNRVWIEENEWMEDKTPVEKWSDVPYSGKREDIWCGSLIGTRARATWAENIQVAINQVRAIIGDEKYVDYMSSLKRYEDTTLVEDTVL

>AHW48567.1 polyprotein [West Nile virus]

MSKKPGGPGKSRAVNMLKRGMPRVLSLIGLKRAMLSLIDGKGPIRFVLALLAFFRFTAIAPTRAVLDRWRGVNKQTAMKHLLSFKKELGTLTSAINRRSSKQKKRGGKTGIAVMIGLIASVGAVTLSNFQGKVMMTVNATDVTDVITIPTAAGKNLCIVRAMDVGYMCDDTITYECPVLSAGNDPEDIDCWCTKSAVYVRYGRCTKTRHSRRSRRSLTVQTHGESTLANKKGAWMDSTKATRYLVKTESWILRNPGYALVAAVIGWMLGSNTMQRVVFVVLLLLVAPAYSFNCLGMSNRDFLEGVSGATWVDLVLEGDSCVTIMSKDKPTIDVKMMNMEAANLAEVRSYCYLATVSDLSTKAACPTMGEAHNDKRADPAFVCRQGVVDRGWGNGCGLFGKGSIDTCAKFACSTKAIGRTILKENIKYEVAIFVHGPTTVESHGNYSTQAGATQAGRFSITPAAPSYTLKLGEYGEVTVDCEPRSGIDTNAYYVMTVGTKTFLVHREWFMDLNLPWSSAGSTVWRNRETLMEFEEPHATKQSVIALGSQEGALHQALAGAIPVEFSSNTVKLTSGHLKCRVKMEKLQLKGTTYGVCSKAFKFLGTPADTGHGTVVLELQYTGTDGPCKVPISSVASLNDLTPVGRLVTVNPFVSVATANAKVLIELEPPFGDSYIVVGRGEQQINHHWHKSGSSIGKAFTTTLKGAQRLAALGDTAWDFGSVGGVFTSVGKAVHQVFGGAFRSLFGGMSWITQGLLGALLLWMGINARDRSIALTFLAVGGVLLFLSVNVHADTGCAIDISRQELRCGSGVFIHNDVEAWMDRYKYYPETPQGLAKIIQKAHKEGVCGLRSVSRLEHQMWEAVKDELNTLLKENGVDLSVVVEKQEGMYKSAPKRLTATTEKLEIGWKAWGKSILFAPELANNTFVVDGPETKECPTQNRAWNSLEVEDFGFGLTSTRMFLKVRESNTTECDSKIIGTAVKNNLAIHSDLSYWIESRLNDTWKLERAVLGEVKSCTWPETHTLWGDGILESDLIIPVTLAGPRSNHNRRPGYKTQNQGPWDEGRVEIDFDYCPGTTVTLSESCGHRGPATRTTTESGKLITDWCCRSCTLPPLRYQTDSGCWYGMEIRPQRHDEKTLVQSQVNAYNADMIDPFQLGLLVVFLATQEVLRKRWTAKISMPAILIALLVLVFGGITYTDVLRYVILVGAAFAESNSGGDVVHLALMATFKIQPVFMVASFLKARWTNQENILLMLAAVFFQMAYHDARQILLWEIPDVLNSLAVAWMILRAITFTTTSNVVVPLLALLTPGLRCLNLDVYRILLLMVGIGSLIREKRSAAAKKKGASLLCLALASTGLFNPMILAAGLIACDPNRKRGWPATEVMTAVGLMFAIVGGLAELDVDSMAIPMTIAGLMFAAFVISGKSTDMWIERTADISWESDAEITGSSERVDVRLDDDGNFQLMNDPGAPWKIWMLRMVCLAISAYTPWAILPSVVGFWITLQYTKRGGVLWDTPSPKEYKKGDTTTGVYRIMTRGLLGSYQAGAGVMVEGVFHTLWHTTKGAALMSGEGRLDPYWGSVKEDRLCYGGPWKLQHKWNGQDEVQMIVVEPGKNVKNVQTKPGVFKTPEGEIGAVTLDFPTGTSGSPIVDKNGDVIGLYGNGVIMPNGSYISAIVQGERMDEPIPAGFEPEMLRKKQITVLDLHPGAGKTRRILPQIIKEAINRRLRTAVLAPTRVVAAEMAEALRGLPIRYQTSAVPREHNGNEIVDVMCHATLTHRLMSPHRVPNYNLFVMDEAHFTDPASIAARGYISTKVELGEAAAIFMTATPPGTSDPFPESNSPISDLQTEIPDRAWNSGYEWITEYTGKTVWFVPSVKMGNEIALCLQRAGKKVVQLNRKSYETEYPKCKNDDWDFVITTDISEMGANFKASRVIDSRKSVKPTIITEGEGRVILGEPSAVTAASAAQRRGRIGRNPSQVGDEYCYGGHTNEDDSNFAHWTEARIMLDNINMPNGLIAQFYQPEREKVYTMDGEYRLRGEERKNFLELLRTADLPVWLAYKVAAAGVSYHDRRWCFDGPRTNTILEDNNEVEVITKLGERKILRPRWIDARVYSDHQALKAFKDFASGKRSQIGLIEVLGKMPEHFMGKTWEALDTMYVVATAEKGGRAHRMALEELPDALQTIALIALLSVMTMGVFFLLMQRKGIGKIGLGGAVLGVATFFCWMAEVPGTKIAGMLLLSLLLMIVLIPEPEKQRSQTDNQLAVFLICVMTLVSAVAANEMGWLDKTKSDISSLFGQRIEVKENFSMGEFLLDLRPATAWSLYAVTTAVLTPLLKHLITSDYINTSLTSINVQASALFTLARGFPFVDVGVSALLLAAGCWGQVTLTVTVTAATLLFCHYAYMVPGWQAEAMRSAQRRTAAGIMKNAVVDGIVATDVPELERTTPIMQKKVGQIMLILVSLAAVVVNPSVKTVREAGILITAAAVTLWENGASSVWNATTAIGLCHIMRGGWLSCLSITWTLIKNMEKPGLKRGGAKGRTLGEVWKERLNQMTKEEFTRYRKEAIIEVDRSAAKHARKEGNVTGGHPVSRGTAKLRWLVERRFLEPVGKVIDLGCGRGGWCYYMATQKRVQEVRGYTKGGPGHEEPQLVQSYGWNIVTMKSGVDVFYRPSECCDTLLCDIGESSSSAEVEEHRTIRVLEMVEDWLHRGPREFCVKVLCPYMPKVIEKMELLQRRYGGGLVRNPLSRNSTHEMYWVSRASGNVVHSVNMTSQVLLGRMEKRTWKGPQYEEDVNLGSGTRAVGKPLLNSDTSKIKNRIERLRREYSSTWHHDENHPYRTWNYHGSYDVKPTGSASSLVNGVVRLLSKPWDTITNVTTMAMTDTTPFGQQRVFKEKVDTKAPEPPEGVKYVLNETTNWLWAFLAREKRPRMCSREEFIRKVNSNAALGAMFEEQNQWRSAREAVEDPKFWEMVDEEREAHLRGECHTCIYNMMGKREKKPGEFGKAKGSRAIWFMWLGARFLEFEALGFLNEDHWLGRKNSGGGVEGLGLQKLGYILREVGTRPGGKIYADDTAGWDTRITRADLENEAKVLELLDGEHRRLARAIIELTYRHKVVKVMRPAADGRTVMDVISREDQRGSGQVVTYALNTFTNLAVQLVRMMEGEGVIGPDDVEKLTKGKGPKVRTWLFENGEERLSRMAVSGDDCVVKPLDDRFATSLHFLNAMSKVRKDIQEWKPSTGWYDWQQVPFCSNHFTELIMKDGRTLVVPCRGQDELVGRARISPGAGWNVRDTACLAKSYAQMWLLLYFHRRDLRLMANAICSAVPVNWVPTGRTTWSIHAGGEWMTTEDMLEVWNRVWIEENEWMEDKTPVEKWSDVPYSGKREDIWCGSLIGTRARATWAENIQVAINQVRAIIGDEKYVDYMSSLKRYEDTTLVEDTVL

>AFB76634.1 polyprotein [West Nile virus]

MSKKPGGPGKSRAVNMLKRGMPRVLSLIGLKRAMLSLIDGKGPIRFVLALLAFFRFTAIAPTRAVLDRWRGVNKQTAMKHLLSFKKELGTLTSAINRRSSKQKKRGGKTGIAVMIGLIASVGAVTLSNFQGKVMMTVNATDVTDVITIPTAAGKNLCIVRAMDVGYMCDDTITYECPVLSAGNDPEDIDCWCTKSAVYVRYGRCTKTRHSRRSRRSLTVQTHGESTLANKKGAWMDSTKATRYLVKTESWILRNPGYALVAAVIGWMLGSNTMQRVVFVVLLLLVAPAYSFNCLGMSNRDFLEGVSGATWVDLVLEGDSCVTIMSKDKPTIDVKMMNMEAANLAEVRSYCYLATVSDLSTKAACPTMGEAHNDKRADPAFVCRQGVVDRGWGNGCGLFGKGSIDTCAKFACSTKAIGRTILKENIKYEVAIFVHGPTTVESHGNYSTQVGATQAGRFSITPAAPSYTLKLGEYGEVTVDCEPRSGIDTNAYYVMTVGTKTFLVHREWFMDLNLPWSSAGSTVWRNRETLMEFEEPHATKQSVIALGSQEGALHQALAGAIPVEFSSNTVKLTSGHLKCRVKMEKLQLKGTTYGVCSKAFKFLGTPADTGHGTVVLELQYTGTDGPCKVPISSVASLNDLTPVGRLVTVNPFVSVATANAKVLIELEPPFGDSYIVVGRGEQQINHHWHKSGSSIGKAFTTTLKGAQRLAALGDTAWDFGSVGGVFTSVGKAVHQVFGGAFRSLFGGMSWITQGLLGALLLWMGINARDRSIALTFLAVGGVLLFLSVNVHADTGCAIDISRQELRCGSGVFIHNDVEAWMDRYKYYPETPQGLAKIIQKAHKEGVCGLRSVSRLEHQMWEAVKDELNTLLKENGVDLSVVVEKQEGMYKSAPKRLTATTEKLEIGWKAWGKSILFAPELANNTFVVDGPETKECPTQNRAWNSLEVEDFGFGLTSTRMFLKVRESNTTECDSKIIGTAVKNNLAIHSDLSYWIESRLNDTWKLERAVLGEVKSCTWPETHTLWGDGILESDLIIPVTLAGPRSNHNRRPGYKTQNQGPWDEGRVEIDFDYCPGTTVTLSESCGHRGPATRTTTESGKLITDWCCRSCTLPPLRYQTDSGCWYGMEIRPQRHDEKTLVQSQVNAYNADMIDPFQLGLLVVFLATQEVLRKRWTAKISMPAILIALLVLVFGGITYTDVLRYVILVGAAFAESNSGGDVVHLALMATFKIQPVFMVASFLKARWTNQENILLMLAAVFFQMAYHDARQILLWEIPDVLNSLAVAWMILRAITFTTTSNVVVPLLALLTPGLRCLNLDVYRILLLMVGIGSLIREKRSAAAKKKGASLLCLALASTGLFNPMILAAGLIACDPNRKRGWPATEVMTAVGLMFAIVGGLAELDIDSMAIPMTIAGLMFAAFVISGKSTDMWIERTADISWESDAEITGSSERVDVRLDDDGNFQLMNDPGAPWKIWMLRMVCLAISAYTPWAILPSVVGFWITLQYTKRGGVLWDTPSPKEYKKGDTTTGVYRIMTRGLLGSYQAGAGVMVEGVFHTLWHTTKGAALMSGEGRLDPYWGSVKEDRLCYGGPWKLQHKWNGQDEVQMIVVEPGKNVKNVQTKPGVFKTPEGEIGAVTLDFPTGTSGSPIVDKNGDVIGLYGNGVIMPNGSYVSAIVQGERMDEPIPAGFEPEMLRKKQITVLDLHPGAGKTRRILPQIIKEAINRRLRTAVLAPTRVVAAEMAEALRGLPIRYQTSAVPREHNGNEIVDVMCHATLTHRLMSPHRVPNYNLFVMDEAHFTDPASIAARGYISTKVELGEAAAIFMTATPPGTSDPFPESNSPISDLQTEIPDRAWNSGYEWITEYTGKTVWFVPSVKMGNEIALCLQRAGKKVVQLNRKSYETEYPKCKNDDWDFVITTDISEMGANFKASRVIDSRKSVKPTIITEGEGRVILGEPSAVTAASAAQRRGRIGRNPSQVGDEYCYGGHTNEDDSNFAHWTEARIMLDNINMPNGLIAQFYQPEREKVYTMDGEYRLRGEERKNFLELLRTADLPVWLAYKVAAAGVSYHDRRWCFDGPRTNTILEDNNEVEVITKLGERKILRPRWIDARVYSDHQALKAFKDFASGKRSQIGLIEVLGKMPEHFMGKTWEALDTMYVVATAEKGGRAHRMALEELPDALQTIALIALLSVMTMGVFFLLMQRKGIGKIGLGGTVLGVATFFCWMAEVPGTKIAGMLLLSLLLMIVLIPEPEKQRSQTDNQLAVFLICVMTLVSAVAANEMGWLDKTKSDISSLFGQRIEVKENFSMGEFLLDLRPATAWSLYAVTTAVLTPLLKHLITSDYINTSLTSINVQASALFTLARGFPFVDVGVSALLLAAGCWGQVTLTVTVTAATLLFCHYAYMVPGWQAEAMRSAQRRTAAGIMKNAVVDGIVATDVPELERTTPIMQKKVGQIMLILVSLAAVVVNPSVKTVREAGILITAAAVTLWENGASSVWNATTAIGLCHIMRGGWLSCLSITWTLIKNMEKPGLKRGGAKGRTLGEVWKERLNQMTKEEFTRYRKEAIIEVDRSAAKHARKEGNVTGGHPVSRGTAKLRWLVERRFLEPVGKVIDLGCGRGGWCYYMATQKRVQEVRGYTKGGPGHEEPQLVQSYGWNIVTMKSGVDVFYRPSECCDTLLCDIGESSSSAEVEEHRTIRVLEMVEDWLHRGPREFCVKVLCPYMPKVIEKMELLQRRYGGGLVRNPLSRNSTHEMYWVSRASGNVVHSVNMTSQVLLGRMEKRTWKGPQYEEDVNLGSGTRAVGKPLLNSDTSKIKNRIERLRREYSSTWHHDENHPYRTWNYHGSYDVKPTGSASSLVNGVVRLLSKPWDTITNVTTMAMTDTTPFGQQRVFKEKVDTKAPEPPEGVKYVLNETTNWLWAFLAREKRPRMCSREEFIRKVNSNAALGAMFEEQNQWRSAREAVEDPKFWEMVDEEREAHLRGECHTCIYNMMGKREKKPGEFGKAKGSRAIWFMWLGARFLEFEALGFLNEDHWLGRKNSGGGVEGLGLQKLGYILREVGTRPGGKIYADDTAGWDTRITRADLENEAKVLELLDGEHRRLARAIIELTYRHKVVKVMRPAADGRTVMDVISREDQRGSGQVVTYALNTFTNLAVQLVRMMEGEGVIGPDDVEKLTKGKGPKVRTWLFENGEERLSRMAVSGDDCVVKPLDDRFATSLHFLNAMSKVRKDIQEWKPSTGWYDWQQVPFCSNHFTELIMKDGRTLVVPCRGQDELVGRARISPGAGWNVRDTACLAKSYAQMWLLLYFHRRDLRLMANAICSAVPVNWVPTGRTTWSIHAGGEWMTTEDMLEVWNRVWIEENEWMEDKTPVEKWSDVPYSGKREDIWCGSLIGTRARATWAENIQVAINQVRAIIGDEKYVDYMSSLKRYEDTTLVEDTVL

>AHW48566.1 polyprotein [West Nile virus]

MSKKPGGPGKSRAVNMLKRGMPRVLSLIGLKRAMLSLIDGKGPIRFVLALLAFFRFTAIAPTRAVLDRWRGVNKQTAMKHLLSFKKELGTLTSAINRRSSKQKKRGGKTGIAVMIGLIASVGAVTLSNFQGKVMMTVNATDVTDVITIPTAAGKNLCIVRAMDVGYMCDDTITYECPVLSAGNDPEDIDCWCTKSAVYVRYGRCTKTRHSRRSRRSLTVQTHGESTLANKKGAWMDSTKATRYLVKTESWILRNPGYALVAAVIGWMLGSNTMQRVVFVVLLLLVAPAYSFNCLGMSNRDFLEGVSGATWVDLVLEGDSCVTIMSKDKPTIDVKMMNMEAANLAEVRSYCYLATVSDLSTKAACPTMGEAHNDKRADPAFVCRQGVVDRGWGNGCGLFGKGSIDTCAKFACSTKAIGRTILKENIKYEVAIFVHGPTTVESHGNYSTQAGATQAGRFSITPAAPSYTLKLGEYGEVTVDCEPRSGIDTNAYYVMTVGTKTFLVHREWFMDLNLPWSSAGSTVWRNRETLMEFEEPHATKQSVIALGSQEGALHQALAGAIPVEFSSNTVKLTSGHLKCRVKMEKLQLKGTTYGVCSKAFKFLGTPADTGHGTVVLELQYTGTDGPCKVPISSVASLNDLTPVGRLVTVNPFVSVATANAKVLIELEPPFGDSYIVVGRGEQQINHHWHKSGSSIGKAFTTTLKGAQRLAALGDTAWDFGSVGGVFTSVGKAVHQVFGGAFRSLFGGMSWITQGLLGALLLWMGINARDRSIALTFLAVGGVLLFLSVNVHADTGCAIDISRQELRCGSGVFIHNDVEAWMDRYKYYPETPQGLAKIIQKAHKEGVCGLRSVSRLEHQMWEAVKDELNTLLKENGVDLSVVVEKQEGMYKSAPKRLTATTEKLEIGWKAWGKSILFAPELANNTFVVDGPETKECPTQNRAWNSLEVEDFGFGLTSTRMFLKVRESNTTECDSKIIGTAVKNNLAIHSDLSYWIESRLNDTWKLERAVLGEVKSCTWPETHTLWGDGILESDLIIPVTLAGPRSNHNRRPGYKTQNQGPWDEGRVEIDFDYCPGTTVTLSESCGHRGPATRTTTESGKLITDWCCRSCTLPPLRYQTDSGCWYGMEIRPQRHDEKTLVQSQVNAYNADMIDPFQLGLLVVFLATQEVLRKRWTAKISMPAVLIALLVLVFGGITYTDVLRYVILVGAAFAESNSGGDVVHLALMATFKIQPVFMVASFLKARWTNQENILLMLAAVFFQMAYHDARQILLWEIPDVLNSLAVAWMILRAITFTTTSNVVVPLLALLTPGLRCLNLDVYRILLLMVGIGSLIREKRSAAAKKKGASLLCLALASTGLFNPMILAAGLIACDPNRKRGWPATEVMTAVGLMFAIVGGLAELDIDSMAIPMTIAGLMFAAFVISGKSTDMWIERTADISWESDAEITGSSERVDVRLDDDGNFQLMNDPGAPWKIWMLRMVCLAISAYTPWAILPSVVGFWITLQYTKRGGVLWDTPSPKEYKKGDTTTGVYRIMTRGLLGSYQAGAGVMVEGVFHTLWHTTKGAALMSGEGRLDPYWGSVKEDRLCYGGPWKLQHKWNGQDEVQMIVVEPGKNVKNVQTKPGVFKTPEGEIGAVTLDFPTGTSGSPIVDKNGDVIGLYGNGVIMPNGSYISAIVQGERMDEPIPAGFEPEMLRKKQITVLDLHPGAGKTRRILPQIIKEAINRRLRTAVLAPTRVVAAEMAEALRGLPIRYQTSAVPREHNGNEIVDVMCHATLTHRLMSPHRVPNYNLFVMDEAHFTDPASIAARGYISTKVELGEAAAIFMTATPPGTSDPFPESNSPISDLQTEIPDRAWNSGYEWITEYTGKTVWFVPSVKMGNEIALCLQRAGKKVVQLNRKSYETEYPKCKNDDWDFVITTDISEMGANFKASRVIDSRKSVKPTIITEGEGRVILGEPSAVTAASAAQRRGRIGRNPSQVGDEYCYGGHTNEDDSNFAHWTEARIMLDNINMPNGLIAQFYQPEREKVYTMDGEYRLRGEERKNFLELLRTADLPVWLAYKVAAAGVSYHDRRWCFDGPRTNTILEDNNEVEVITKLGERKILRPRWIDARVYSDHQALKAFKDFASGKRSQIGLIEVLGKMPEHFMGKTWEALDTMYVVATAEKGGRAHRMALEELPDALQTIALIALLSVMTMGVFFLLMQRKGIGKIGLGGAVLGVATFFCWMAEVPGTKIAGMLLLSLLLMIVLIPEPEKQRSQTDNQLAVFLICVMTLVSAVAANEMGWLDKTKSDISSLFGQRIEVKENFSMGEFLLDLRPATAWSLYAVTTAVLTPLLKHLITSDYINTSLTSINVQASALFTLARGFPFVDVGVSALLLAAGCWGQVTLTVTVTAATLLFCHYAYMVPGWQAEAMRSAQRRTAAGIMKNAVVDGIVATDVPELERTTPIMQKKVGQIMLILVSLAAVVVNPSVKTVREAGILITAAAVTLWENGASSVWNATTAIGLCHIMRGGWLSCLSITWTLIKNMEKPGLKRGGAKGRTLGEVWKERLNQMTKEEFTRYRKEAIIEVDRSAAKHARKEGNVTGGHPVSRGTAKLRWLVERRFLEPVGKVIDLGCGRGGWCYYMATQKRVQEVRGYTKGGPGHEEPQLVQSYGWNIVTMKSGVDVFYRPSECCDTLLCDIGESSSSAEVEEHRTIRVLEMVEDWLHRGPREFCVKVLCPYMPKVIEKMELLQRRYGGGLVRNPLSRNSTHEMYWVSRASGNVVHSVNMTSQVLLGRMEKRTWKGPQYEEDVNLGSGTRAVGKPLLNSDTSKIKNRIERLRREYSSTWHHDENHPYRTWNYHGSYDVKPTGSASSLVNGVVRLLSKPWDTITNVTTMAMTDTTPFGQQRVFKEKVDTKAPEPPEGVKYVLNETTNWLWAFLAREKRPRMCSREEFIRKVNSNAALGAMFEEQNQWRSAREAVEDPKFWEMVDEEREAHLRGECHTCIYNMMGKREKKPGEFGKAKGSRAIWFMWLGARFLEFEALGFLNEDHWLGRKNSGGGVEGLGLQKLGYILREVGTRPGGKIYADDTAGWDTRITRADLENEAKVLELLDGEHRRLARAIIELTYRHKVVKVMRPAADGRTVMDVISREDQRGSGQVVTYALNTFTNLAVQLVRMMEGEGVIGPDDVEKLTKGKGPKVRTWLFENGEERLSRMAVSGDDCVVKPLDDRFATSLHFLNAMSKVRKDIQEWKPSTGWYDWQQVPFCSNHFTELIMKDGRTLVVPCRGQDELVGRARISPGAGWNVRDTACLAKSYAQMWLLLYFHRRDLRLMANAICSAVPVNWVPTGRTTWSIHAGGEWMTTEDMLEVWNRVWIEENEWMEDKTPVEKWSDVPYSGKREDIWCGSLIGTRARATWAENIQVAINQVRAIIGDEKYVDYMSSLKRYEDTTLVEDTVL

>ADK62437.1 polyprotein [West Nile virus]

MSKKPGGPGKSRAVNMLKRGMPRVLSLIGLKRAMLSLIDGKGPIRFVLALLAFFRFTAIAPTRAVLDRWRGVNKQTAMKHLLSFKKELGTLTSAINRRSSKQKKRGGKTGIAVMIGLIASVGAVTLSNFQGKVMMTVNATDVTDVITIPTAAGKNLCIVRAMDVGYMCDDTITYECPVLSAGNDPEDIDCWCTKSAVYVRYGRCTKTRHSRRSRRSLTVQTHGESTLANKKGAWMDSTKATRYLVKTESWILRNPGYALVAAVIGWMLGSNTMQRVVFVVLLLLVAPAYSFNCLGMSNRDFLEGVSGATWVDLVLEGDSCVTIMSKDKPTIDVKMMNMEAANLAEVRSYCYLATVSDLSTKAACPTMGEAHNDKRADPAFVCRQGVVDRGWGNGCGLFGKGSIDTCAKFACSTKAIGRTILKENIKYEVAIFVHGPTTVESHGNYSTQAGATQAGRFSITPAAPSYTLKLGEYGEVTVDCEPRSGIDTNAYYVMTVGTKTFLVHREWFMDLNLPWSSAGSTVWRNRETLMEFEEPHATKQSVIALGSQEGALHQALAGAIPVEFSSNTVKLTSGHLKCRVKMEKLQLKGTTYGVCSKAFKFLGTPADTGHGTVVLELQYTGTDGPCKVPISSVASLNDLTPVGRLVTVNPFVSVATANAKVLIELEPPFGDSYIVVGRGEQQINHHWHKSGSSIGKAFTTTLKGAQRLAALGDTAWDFGSVGGVFTSVGKAVHQVFGGAFRSLFGGMSWITQGLLGALLLWMGINARDRSIALTFLAVGGVLLFLSVNVHADTGCAIDISRQELRCGSGVFIHNDVEAWMDRYKYYPETPQGLAKIIQKAHKEGVCGLRSVSRLEHQMWEAVKDELNTLLKENGVDLSVVVEKQEGMYKSAPKRLTATTEKLEIGWKAWGKSILFAPELANNTFVVDGPETKECPTQNRAWNSLEVEDFGFGLTSTRMFLKVRESNTTECDSKIIGTAVKNNLAIHSDLSYWIESRLNDTWKLERAVLGEVKSCTWPETHTLWGDGILESDLIIPVTLAGPRSNHNRRPGYKTQNQGPWDEGRVEIDFDYCPGTTVTLSESCGHRGPATRTTTESGKLITDWCCRSCTLPPLRYQTDSGCWYGMEIRPQRHDEKTLVQSQVNAYNADMIDPFQLGLLVVFLATQEVLRKRWTAKISMPAILIALLVLVFGGITYTDVLRYVILVGAAFAESNSGGDVVHLALMATFKIQPVFMVASFLKARWTNQENILLMLAAVFFQMAYHDARQILLWEIPDVLNSLAVAWMILRAITFTTTSNVVVPLLALLTPGMRCLNLDVYRILLLMVGIGSLIREKRSAAAKKKGASLLCLALASTGLFNPMILAAGLIACDPNRKRGWPATEVMTAVGLMFAIVGGLAELDIDSMAIPMTIAGLMFAAFVISGKSTDMWIERTADISWESDAEITGSSERVDVRLDDDGNFQLMNDPGAPWKIWMLRMVCLAISAYTPWAILPSVVGFWITLQYTKRGGVLWDTPSPKEYKKGDTTTGVYRIMTRGLLGSYQAGAGVMVEGVFHTLWHTTKGAALMSGEGRLDPYWGSVKEDRLCYGGPWKLQHKWNGQDEVQMIVVEPGKNVKNVQTKPGVFKTPEGEIGAVTLDFPTGTSGSPIVDKNGDVIGLYGNGVIMPNGSYISAIVQGERMDEPIPAGFEPEMLRKKQITVLDLHPGAGKTRRILPQIIKEAINRRLRTAVLAPTRVVAAEMAEALRGLPIRYQTSAVPREHNGNEIVDVMCHATLTHRLMSPHRVPNYNLFVMDEAHFTDPASIAARGYISTKVELGEAAAIFMTATPPGTSDPFPESNSPISDLQTEIPDRAWNSGYEWITEYTGKTVWFVPSVKMGNEIALCLQRAGKKVVQLNRKSYETEYPKCKNDDWDFVITTDISEMGANFKASRVIDSRKSVKPTIITEGEGRVILGEPSAVTAASAAQRRGRIGRNPSQVGDEYCYGGHTNEDDSNFAHWTEARIMLDNINMPNGLIAQFYQPEREKVYTMDGEYRLRGEERKNFLELLRTADLPVWLAYKVAAAGVSYHDRRWCFDGPRTNTILEDNNEVEVITKLGERKILRPRWIDARVYSDHQALKAFKDFASGKRSQIGLIEVLGKMPEHFMGKTWEALDTMYVVATAEKGGRAHRMALEELPDALQTIALIALLSVMTMGVFFLLMQRKGIGKIGLGGAVLGVATFFCWMAEVPGTKIAGMLLLSLLLMIVLIPEPEKQRSQTDNQLAVFLICVMTLVSAVAANEMGWLDKTKSDISSLFGQRIEVKENFSMGEFLLDLRPATAWSLYAVTTAVLTPLLKHLITSDYINTSLTSINVQASALFTLARGFPFVDVGVSALLLAAGCWGQVTLTVTVTAATLLFCHYAYMVPGWQAEAMRSAQRRTAAGIMKNAVVDGIVATDVPELERTTPIMQKKVGQIMLILVSLAAVVVNPSVKTVREAGILITAAAVTLWENGASSVWNATTAIGLCHIMRGGWLSCLSITWTLIKNMEKPGLKRGGAKGRTLGEVWKERLNQMTKEEFTRYRKEAIIEVDRSAAKHARKEGNVTGGHPVSRGTAKLRWLVERRFLEPVGKVIDLGCGRGGWCYYMATQKRVQEVRGYTKGGPGHEEPQLVQSYGWNIVTMKSGVDVFYRPSECCDTLLCDIGESSSSAEVEEHRTIRVLEMVEDWLHRGPREFCVKVLCPYMPKVIEKMELLQRRYGGGLVRNPLSRNSTHEMYWVSRASGNVVHSVNMTSQVLLGRMEKRTWKGPQYEEDVNLGSGTRAVGKPLLNSDTSKIKNRIERLRREYSSTWHHDENHPYRTWNYHGSYDVKPTGSASSLVNGVVRLLSKPWDTITNVTTMAMTDTTPFGQQRVFKEKVDTKAPEPPEGVKYVLNETTNWLWAFLAREKRPRMCSREEFIRKVNSNAALGAMFEEQNQWRSAREAVEDPKFWEMVDEEREAHLRGECHTCIYNMMGKREKKPGEFGKAKGSRAIWFMWLGARFLEFEALGFLNEDHWLGRKNSGGGVEGLGLQKLGYILREVGTRPGGKIYADDTAGWDTRITRADLENEAKVLELLDGEHRRLARAIIELTYRHKVVKVMRPAADGRTVMDVISREDQRGSGQVVTYALNTFTNLAVQLVRMMEGEGVIGPDDVEKLTKGKGPKVRTWLFENGEERLSRMAVSGDDCVVKPLDDRFATSLHFLNAMSKVRKDIQEWKPSTGWYDWQQVPFCSNHFTELIMKDGRTLVVPCRGQDELVGRARISPGAGWNVRDTACLAKSYAQMWLLLYFHRRDLRLMANAICSAVPVNWVPTGRTTWSIHAGGEWMTTEDMLEVWNRVWIEENEWMEDKTPVEKWSDVPYSGKREDIWCGSLIGTRARATWAENIQVAINQVRAIIGDEKYVDYMSSLKRYEDTTLVEDTVL

>ADU76266.1 polyprotein [West Nile virus]

MSKKPGGPGKSRAVNMLKRGMPRVLSLIGLKRAMLSLIDGKGPIRFVLALLAFFRFTAIAPTRAVLDRWRGVNKQTAMKHLLSFKKELGTLTSAINRRSSKQKKRGGKTGIAVMIGLIASVGAVTLSNFQGKVMMTVNATDVTDVITIPTAAGKNLCIVRAMDVGYMCDDTITYECPVLSAGNDPEDIDCWCTKSAVYVRYGRCTKTRHSRRSRRSLTVQTHGESTLANKKGAWMDSTKATRYLVKTESWILRNPGYALVAAVIGWMLGSNTMQRVVFVVLLLLVAPAYSFNCLGMSNRDFLEGVSGATWVDLVLEGDSCVTIMSKDKPTIDVKMMNMEAANLAEVRSYCYLATVSDLSTKAACPTMGEAHNDKRADPAFVCRQGVVDRGWGNGCGLFGKGSIDTCAKFACSTKAVGRTILKENIKYEVAIFVHGPTTVESHGNYSTQAGATQAGRFSITPAAPSYTLKLGEYGEVTVDCEPRSGIDTNAYYVMTVGTKTFLVHREWFMDLNLPWSSAGSTVWRNRETLMEFEEPHATKQSVIALGSQEGALHQALAGAIPVEFSSNTVKLTSGHLKCRVKMEKLQLKGTTYGVCSKAFKFLGTPADTGHGTVVLELQYTGTDGPCKVPISSVASLNDLTPVGRLVTVNPFVSVATANAKVLIELEPPFGDSYIVVGRGEQQINHHWHKSGSSIGKAFTTTLKGAQRLAALGDTAWDFGSVGGVFTSVGKAVHQVFGGAFRSLFGGMSWITQGLLGALLLWMGINARDRSIALTFLAVGGVLLFLSVNVHADTGCAIDISRQELRCGSGVFIHNDVEAWMDRYKYYPETPQGLAKIIQKAHKEGVCGLRSVSRLEHQMWEAVKDELNTLLKENGVDLSVVVEKQEGMYKSAPKRLTATTEKLEIGWKAWGKSILFAPELANNTFVVDGPETKECPTQNRAWNSLEVEDFGFGLTSTRMFLKVRESNTTECDSKIIGTAVKNNLAIHSDLSYWIESRLNDTWKLERAVLGEVKSCTWPETHTLWGDGILESDLIIPVTLAGPRSNHNRRPGYKTQNQGPWDEGRVEIDFDYCPGTTVTLSESCGHRGPATRTTTESGKLITDWCCRSCTLPPLRYQTDSGCWYGMEIRPQRHDEKTLVQSQVNAYNADMIDPFQLGLLVVFLATQEVLRKRWTAKISMPAILIALLVLVFGGITYTDVLRYVILVGAAFAESNSGGDVVHLALMATFKIQPVFMVASFLKARWTNQENILLMLAAVFFQMAYHDARQILLWEIPDVLNSLAVAWMILRAITFTTTSNVVVPLLALLTPGLRCLNLDVYRILLLMVGIGSLIREKRSAAAKKKGASLLCLALASTGLFNPMILAAGLIACDPNRKRGWPATEVMTAVGLMFAIVGGLAELDIDSMAIPMTIAGLMFAAFVISGKSTDMWIERTADISWESDAEITGSSERVDVRLDDDGNFQLMNDPGAPWKIWMLRMVCLAISAYTPWAILPSVVGFWITLQYTKRGGVLWDTPSPKEYKKGDTTTGVYRIMTRGLLGSYQAGAGVMVEGVFHTLWHTTKGAALMSGEGRLDPYWGSVKEDRLCYGGPWKLQHKWNGQDEVQMIVVEPGKNVKNVQTKPGVFKTPEGEIGAVTLDFPTGTSGSPIVDKNGDVIGLYGNGVIMPNGSYISAIVQGERMDEPIPAGFEPEMLRKKQITVLDLHPGAGKTRRILPQIIKEAINRRLRTAVLAPTRVVAAEMAEALRGLPIRYQTSAVPREHNGNEIVDVMCHATLTHRLMSPHRVPNYNLFVMDEAHFTDPASIAARGYISTKVELGEAAAIFMTATPPGTSDPFPESNSPISDLQTEIPDRAWNSGYEWITEYTGKTVWFVPSVKMGNEIALCLQRAGKKVVQLNRKSYETEYPKCKNDDWDFVITTDISEMGANFKASRVIDSRKSVKPTIITEGEGRVILGEPSAVTAASAAQRRGRIGRNPSQVGDEYCYGGHTNEDDSNFAHWTEARIMLDNINMPNGLIAQFYQPEREKVYTMDGEYRLRGEERKNFLELLRTADLPVWLAYKVAAAGVSYHDRRWCFDGPRTNTILEDNNEVEVITKLGERKILRPRWIDARVYSDHQALKAFKDFASGKRSQIGLIEVLGKMPEHFMGKTWEALDTMYVVATAEKGGRAHRMALEELPDALQTIALIALLSVMTMGVFFLLMQRKGIGKIGLGGAVLGVATFFCWMAEVPGTKIAGMLLLSLLLMIVLIPEPEKQRSQTDNQLAVFLICVMTLVSAVAANEMGWLDKTKSDISSLFGQRIEVKENFSMGEFLLDLRPATAWSLYAVTTAVLTPLLKHLITSDYINTSLTSINVQASALFTLARGFPFVDVGVSALLLAAGCWGQVTLTVTVTAATLLFCHYAYMVPGWQAEAMRSAQRRTAAGIMKNAVVDGIVATDVPELERTTPIMQKKVGQIMLILVSLAAVVVNPSVKTVREAGILITAAAVTLWENGASSVWNATTAIGLCHIMRGGWLSCLSITWTLIKNMEKPGLKRGGAKGRTLGEVWKERLNQMTKEEFTRYRKEAIIEVDRSAAKHARKEGNVTGGHPVSRGTAKLRWLVERRFLEPVGKVIDLGCGRGGWCYYMATQKRVQEVRGYTKGGPGHEEPQLVQSYGWNIVTMKSGVDVFYRPSECCDTLLCDIGESSSSAEVEEHRTIRVLEMVEDWLHRGPREFCVKVLCPYMPKVIEKMELLQRRYGGGLVRNPLSRNSTHEMYWVSRASGNVVHSVNMTSQVLLGRMEKRTWKGPQYEEDVNLGSGTRAVGKPLLNSDTSKIKNRIERLRREYSSTWHHDENHPYRTWNYHGSYDVKPTGSASSLVNGVVRLLSKPWDTITNVTTMAMTDTTPFGQQRVFKEKVDTKAPEPPEGVKYVLNETTNWLWAFLAREKRPRMCSREEFIRKVNSNAALGAMFEEQNQWRSAREAVEDPKFWEMVDEEREAHLRGECHTCIYNMMGKREKKPGEFGKAKGSRAIWFMWLGARFLEFEALGFLNEDHWLGRKNSGGGVEGLGLQKLGYILREVGTRPGGKIYADDTAGWDTRITRADLENEAKVLELLDGEHRRLARAIIELTYRHKVVKVMRPAADGRTVMDVISREDQRGSGQVVTYALNTFTNLAVQLVRMMEGEGVIGPDDVEKLTKGKGPKVRTWLFENGEERLSRMAVSGDDCVVKPLDDRFATSLHFLNAMSKVRKDIQEWKPSTGWYDWQQVPFCSNHFTELIMKDGRTLVVPCRGQDELVGRARISPGAGWNVRDTACLAKSYAQMWLLLYFHRRDLRLMANAICSAVPVNWVPTGRTTWSIHAGGEWMTTEDMLEVWNRVWIEENEWMEDKTPVEKWSDVPYSGKREDIWCGSLIGTRARATWAENIQVAINQVRAIIGDEKYVDYMSSLKRYEDTTLVEDTVL

>ABA54582.1 polyprotein precursor [West Nile virus]

MSKKPGGPGKSRAVNMLKRGMPRVLSLIGLKRAMLSLIDGKGPIRFVLALLAFFRFTAVAPTRAVLDRWRGVNKQTAMKHLLSFKKELGTLTSAINRRSSKQKKRGGKTGIAVMIGLIASVGAVTLSNFQGKVMMTVNATDVTDVITIPTAAGKNLCIVRAMDVGYMCDDTITYECPVLSAGNDPEDIDCWCTKSAVYVRYGRCTKTRHSRRSRRSLTVQTHGESTLANKKGAWMDSTKATRYLVKTESWILRNPGYALVAAVIGWMLGSNTMQRVVFVVLLLLVAPAYSFNCLGMSNRDFLEGVSGATWVDLVLEGDSCVTIMSKDKPTIDVKMMNMEAANLAEVRSYCYLATVSDLSTKAACPTMGEAHNDKRADPAFVCRQGVVDRGWGNGCGLFGKGSIDTCAKFACSTKAIGRTILKENIKYEVAIFVHGPTTVESHGNYSTQAGATQAGRFSITPAAPSYTLKLGEYGEVTVDCEPRSGIDTNAYYVMTVGTKTFLVHREWFMDLNLPWSSAGSTVWRNRETLMEFEEPHATKQSVIALGSQEGALHQALAGAIPVEFSSNTVKLTSGHLKCRVKMEKLQLKGTTYGVCSKAFKFLGTPADTGHGTVVLELQYTGTDGPCKVPISSVASLNDLTPVGRLVTVNPFVSVATANAKVLIELEPPFGDSYIVVGRGEQQINHHWHKSGSSIGKAFTTTLKGAQRLAALGDTAWDFGSVGGVFTSVGKAVHQVFGGAFRSLFGGMSWITQGLLGALLLWMGINARDRSIALTFLAVGGVLLFLSVNVHADTGCAIDISRQELRCGSGVFIHNDVEAWMDRYKYYPETPQGLAKIIQKAHKEGVCGLRSVSRLEHQMWEAVKDELNTLLKENGVDLSVVVEKQEGMYKSAPKRLTATTEKLEIGWKAWGKSILFAPELANNTFVVDGPETKECPTQNRAWNSLEVEDFGFGLTSTRMFLKVRESNTTECDSKIIGTAVKNNLAIHSDLSYWIESRLNDTWKLERAVLGEVKSCTWPETHTLWGDGILESDLIIPVTLAGPRSNHNRRPGYKTQNQGPWDEGRVEIDFDYCPGTTVTLSESCGHRGPATRTTTESGKLITDWCCRSCTLPPLRYQTDSGCWYGMEIRPQRHDEKTLVQSQVNAYNADMIDPFQLGLLVVFLATQEVLRKRWTAKISMPAILIALLVLVFGGITYTDVLRYVILVGAAFAESNSGGDVVHLALMATFKIQPVFMVASFLKARWTNQENILLMLAAVFFQMAYHDARQILLWEIPDVLNSLAVAWMILRAITFTTTSNVVVPLLALLTPGLRCLNLDVYRILLLMVGIGSLIREKRSAAAKKKGASLLCLALASTGLFNPMILAAGLIACDPNRKRGWPATEVMTAVGLMFAIVGGLAELDIDSMAIPMTIAGLMFAAFVISGKSTDMWIERTADISWESDAEITGSSERVDVRLDDDGNFQLMNDPGAPWKIWMLRMVCLAISAYTPWAILPSVVGFWITLQYTKRGGVLWDTPSPKEYKKGDTTTGVYRIMTRGLLGSYQAGAGVMVEGVFHTLWHTTKGAALMSGEGRLDPYWGSVKEDRLCYGGPWKLQHKWNGQDEVQMIVVEPGKNVKNVQTKPGVFKTPEGEIGAVTLDFPTGTSGSPIVDKNGDVIGLYGNGVIMPNGSYISAIVQGERMDEPIPAGFEPEMLRKKQITVLDLHPGAGKTRRILPQIIKEAINRRLRTAVLAPTRVVAAEMAEALRGLPIRYQTSAVPREHNGNEIVDVMCHATLTHRLMSPHRVPNYNLFVMDEAHFTDPASIAARGYISTKVELGEAAAIFMTATPPGTSDPFPESNSPISDLQTEIPDRAWNSGYEWITEYTGKTVWFVPSVKMGNEIALCLQRAGKKVVQLNRKSYETEYPKCKNDDWDFVITTDISEMGANFKASRVIDSRKSVKPTIITEGEGRVILGEPSAVTAASAAQRRGRIGRNPSQVGDEYCYGGHTNEDDSNFAHWTEARIMLDNINMPNGLIAQFYQPEREKVYTMDGEYRLRGEERKNFLELLRTADLPVWLAYKVAAAGVSYHDRRWCFDGPRTNTILEDNNEVEVITKLGERKILRPRWIDARVYSDHQALKAFKDFASGKRSQIGLIEVLGKMPEHFMGKTWEALDTMYVVATAEKGGRAHRMALEELPDALQTIALIALLSVMTMGVFFLLMQRKGIGKIGLGGAVLGVATFFCWMAEVPGTKIAGMLLLSLLLMIVLIPEPEKQRSQTDNQLAVFLICVMTLVSAVAANEMGWLDKTKSDISSLFGQRIEVKENFSMGEFLLDLRPATAWSLYAVTTAVLTPLLKHLITSDYINTSLTSINVQASALFTLARGFPFVDVGVSALLLAAGCWGQVTLTVTVTAATLLFCHYAYMVPGWQAEAMRSAQRRTAAGIMKNAVVDGIVATDVPELERTTPIMQKKVGQIMLILVSLAAVVVNPSVKTVREAGILITAAAVTLWENGASSVWNATTAIGLCHIMRGGWLSCLSITWTLIKNMEKPGLKRGGAKGRTLGEVWKERLNQMTKEEFTRYRKEAIIEVDRSAAKHARKEGNVTGGHPVSRGTAKLRWLVERRFLEPVGKVIDLGCGRGGWCYYMATQKRVQEVRGYTKGGPGHEEPQLVQSYGWNIVTMKSGVDVFYRPSECCDTLLCDIGESSSSAEVEEHRTIRVLEMVEDWLHRGPREFCVKVLCPYMPKVIEKMELLQRRYGGGLVRNPLSRNSTHEMYWVSRASGNVVHSVNMTSQVLLGRMEKRTWKGPQYEEDVNLGSGTRAVGKPLLNSDTSKIKNRIERLRREYSSTWHHDENHPYRTWNYHGSYDVKPTGSASSLVNGVVRLLSKPWDTITNVTTMAMTDTTPFGQQRVFKEKVDTKAPEPPEGVKYVLNETTNWLWAFLAREKRPRMCSREEFIRKVNSNAALGAMFEEQNQWRSAREAVEDPKFWEMVDEEREAHLRGECHTCIYNMMGKREKKPGEFGKAKGSRAIWFMWLGARFLEFEALGFLNEDHWLGRKNSGGGVEGLGLQKLGYILREVGTRPGGKIYADDTAGWDTRITRADLENEAKVLELLDGEHRRLARAIIELTYRHKVVKVMRPAADGRTVMDVISREDQRGSGQVVTYALNTFTNLAVQLVRMMEGEGVIGPDDVEKLTKGKGPKVRTWLFENGEERLSRMAVSGDDCVVKPLDDRFATSLHFLNAMSKVRKDIQEWKPSTGWYDWQQVPFCSNHFTELIMKDGRTLVVPCRGQDELVGRARISPGAGWNVRDTACLAKSYAQMWLLLYFHRRDLRLMANAICSAVPVNWVPTGRTTWSIHAGGEWMTTEDMLEVWNRVWIEENEWMEDKTPVEKWSDVPYSGKREDIWCGSLIGTRARATWAENIQVAINQVRAIIGDEKYVDYMSSLKRYEDTTLVEDTVL

>ANW68968.1 genome polyprotein [West Nile virus]

MSKKPGGPGKSRAVNMLKRGMPRVLSLIGLKRAMLSLVDGKGPIRFVLALLAFFRFTAIAPTRAVLDRWRGVNKQTAMKHLLSFKKELGTLTSAINRRSSKQKKRGGKTGIAVMIGLIASVGAVTLSNFQGKVMMTVNATDVTDVITIPTAAGKNLCIVRAMDVGYMCDDTITYECPVLSAGNDPEDIDCWCTKSAVYVRYGRCTKTRHSRRSRRSLTVQTHGESTLANKKGAWMDSTKATRYLVKTESWILRNPGYALVAAVIGWMLGSNTMQRVVFVVLLLLVAPAYSFNCLGMSNRDFLEGVSGATWVDLVLEGDSCVTIMSKDKPTIDVKMMNMEAANLAEVRSYCYLATVSDLSTKAACPTMGEAHNDKRADPAFVCRQGVVDRGWGNGCGLFGKGSIDTCAKFACSTKAIGRTILKENIKYEVAIFVHGPTTVESHGNYSTQAGATQAGRFSITPAAPSYTLKLGEYGEVTVDCEPRSGIDTNAYYVMTVGTKTFLVHREWFMDLNLPWSSAGSTVWRNRETLMEFEEPHATKQSVIALGSQEGALHQALAGAIPVEFSSNTVKLTSGHLKCRVKMEKLQLKGTTYGVCSKAFKFLGTPADTGHGTVVLELQYTGTDGPCKVPISSVASLNDLTPVGRLVTVNPFVSVATANAKVLIELEPPFGDSYIVVGRGEQQINHHWHKSGSSIGKAFTTTLKGAQRLAALGDTAWDFGSVGGVFTSVGKAVHQVFGGAFRSLFGGMSWITQGLLGALLLWMGINARDRSIALTFLAVGGVLLFLSVNVHADTGCAIDISRQELRCGSGVFIHNDVEAWMDRYKYYPETPQGLAKIIQKAHKEGVCGLRSVSRLEHQMWEAVKDELNTLLKENGVDLSVVVEKQEGMYKSAPKRLTATTEKLEIGWKAWGKSILFAPELANNTFVVDGPETKECPTQNRAWNSLEVEDFGFGLTSTRMFLKVRESNTTECDSKIIGTAVKNNLAIHSDLSYWIESRLNDTWKLERAVLGEVKSCTWPETHTLWGDGILESDLIIPVTLAGPRSNHNRRPGYKTQNQGPWDEGRVEIDFDYCPGTTVTLSESCGHRGPATRTTTESGKLITDWCCRSCTLPPLRYQTDSGCWYGMEIRPQRHDEKTLVQSQVNAYNADMIDPFQLGLLVVFLATQEVLRKRWTAKISMPAILIALLVLVFGGITYTDVLRYVILVGAAFAESNSGGDVVHLALMATFKIQPVFMVASFLKARWTNQENILLMLAAVFFQMAYHDARQILLWEIPDVLNSLAVAWMILRAITFTTTSNVVVPLLALLTPGLRCLNLDVYRILLLMVGIGSLIREKRSAAAKKKGASLLCLALASTGLFNPMILAAGLIACDPNRKRGWPATEVMTAVGLMFAIVGGLAELDIDSMAIPMTIAGLMFAAFVISGKSTDMWIERTADISWESDAEITGSSERVDVRLDDDGNFQLMNDPGAPWKIWMLRMVCLAISAYTPWAILPSVVGFWITLQYTKRGGVLWDTPSPKEYKKGDTTTGVYRIMTRGLLGSYQAGAGVMVEGVFHTLWHTTKGAALMSGEGRLDPYWGSVKEDRLCYGGPWKLQHKWNGQDEVQMIVVEPGKNVKNVQTKPGVFKTPEGEIGAVTLDFPTGTSGSPIVDKNGDVIGLYGNGVIMPNGSYISAIVQGERMDEPIPAGFEPEMLRKKQITVLDLHPGAGKTRRILPQIIKEAINRRLRTAVLAPTRVVAAEMAEALRGLPIRYQTSAVPREHNGNEIVDVMCHATLTHRLMSPHRVPNYNLFVMDEAHFTDPASIAARGYISTKVELGEAAAIFMTATPPGTSDPFPESNSPISDLQTEIPDRAWNSGYEWITEYTGKTVWFVPSVKMGNEIALCLQRAGKKVVQLNRKSYETEYPKCKNDDWDFVITTDISEMGANFKASRVIDSRKSVKPTIITEGEGRVILGEPSAVTAASAAQRRGRIGRNPSQVGDEYCYGGHTNEDDSNFAHWTEARIMLDNINMPNGLIAQFYQPEREKVYTMDGEYRLRGEERKNFLELLRTADLPVWLAYKVAAAGVSYHDRRWCFDGPRTNTILEDNNEVEVITKLGERKILRPRWIDARVYSDHQALKAFKDFASGKRSQIGLIEVLGKMPEHFMGKTWEALDTMYVVATAEKGGRAHRMALEELPDALQTIALIALLSVMTMGVFFLLMQRKGIGKIGLGGAVLGVATFFCWMAEVPGTKIAGMLLLSLLLMIVLIPEPEKQRSQTDNQLAVFLICVMTLVSAVAANEMGWLDKTKSDISSLFGQRIEVKENFSMGEFLLDLRPATAWSLYAVTTAVLTPLLKHLITSDYINTSLTSINVQASALFTLARGFPFVDVGVSALLLAAGCWGQVTLTVTVTAATLLFCHYAYMVPGWQAEAMRSAQRRTAAGIMKNAVVDGIVATDVPELERTTPIMQKKVGQIMLILVSLAAVVVNPSVKTVREAGILITAAAVTLWENGASSVWNATTAIGLCHIMRGGWLSCLSITWTLIKNMEKPGLKRGGAKGRTLGEVWKERLNQMTKEEFTRYRKEAIIEVDRSAAKHARKEGNVTGGHPVSRGTAKLRWLVERRFLEPVGKVIDLGCGRGGWCYYMATQKRVQEVRGYTKGGPGHEEPQLVQSYGWNIVTMKSGVDVFYRPSECCDTLLCDIGESSSSAEVEEHRTIRVLEMVEDWLHRGPREFCVKVLCPYMPKVIEKMELLQRRYGGGLVRNPLSRNSTHEMYWVSRASGNVVHSVNMTSQVLLGRMEKRTWKGPQYEEDVNLGSGTRAVGKPLLNSDTSKIKNRIERLRREYSSTWHHDENHPYRTWNYHGSYDVKPTGSASSLVNGVVRLLSKPWDTITNVTTMAMTDTTPFGQQRVFKEKVDTKAPEPPEGVKYVLNETTNWLWAFLAREKRPRMCSREEFIRKVNSNAALGAMFEEQNQWRSAREAVEDPKFWEMVDEEREAHLRGECHTCIYNMMGKREKKPGEFGKAKGSRAIWFMWLGARFLEFEALGFLNEDHWLGRKNSGGGVEGLGLQKLGYILREVGTRPGGKIYADDTAGWDTRITRADLENEAKVLELLDGEHRRLARAIIELTYRHKVVKVMRPAADGRTVMDVISREDQRGSGQVVTYALNTFTNLAVQLVRMMEGEGVIGPDDVEKLTKGKGPKVRTWLFENGEERLSRMAVSGDDCVVKPLDDRFATSLHFLNAMSKVRKDIQEWKPSTGWYDWQQVPFCSNHFTELIMKDGRTLVVPCRGQDELVGRARISPGAGWNVRDTACLAKSYAQMWLLLYFHRRDLRLMANAICSAVPVNWVPTGRTTWSIHAGGEWMTTEDMLEVWNRVWIEENEWMEDKTPVEKWSDVPYSGKREDIWCGSLIGTRARATWAENIQVAINQVRAIIGDEKYVDYMSSLKRYEDTTLVEDTVL

>ANW68479.1 genome polyprotein [West Nile virus]

MSKKPGGPGKSRAVNMLKRGMPRVLSLIGLKRAMLSLIDGKGPIRFVLALLAFFRFTAIAPTRAVLDRWRGVNKQTAMKHLLSFKKELGTLTSAINRRSSKQKKRGGKTGIAVMIGLIASVGAVTLSNFQGKVMMTVNATDVTDVITIPTAAGKNLCIVRAMDVGYMCDDTITYECPVLSAGNDPEDIDCWCTKSAVYVRYGRCTKTRHSRRSRRSLTVQTHGESTLANKKGAWMDSTKATRYLVKTESWILRNPGYALVAAVIGWMLGSNTMQRVVFVVLLLLVAPAYSFNCLGMSNRDFLEGVSGATWVDLVLEGDSCVTIMSKDKPTIDVKMMNMEAANLAEVRSYCYLATVSDLSTKAACPTMGEAHNDKRADPAFVCRQGVVDRGWGNGCGLFGKGSIDTCAKFACSTKAIGRTILKENIKYEVAIFVHGPTTVESHGNYSTQAGATQAGRFSITPAAPSYTLKLGEYGEVTVDCEPRSGIDTNAYYVMTVGTKTFLVHREWFMDLNLPWSSAGSTVWRNRETLMEFEEPHATKQSVIALGSQEGALHQALAGAIPVEFSSNTVKLTSGHLKCRVKMEKLQLKGTTYGVCSKAFKFLGTPADTGHGTVVLELQYTGTDGPCKVPISSVASLNDLTPVGRLVTVNPFVSVATANAKVLIELEPPFGDSYIVVGRGEQQINHHWHKSGSSIGKAFTTTLKGAQRLAALGDTAWDFGSVGGVFTSVGKAVHQVFGGAFRSLFGGMSWITQGLLGALLLWMGINARDRSIALTFLAVGGVLLFLSVNVHADTGCAIDISRQELRCGSGVFIHNDVEAWMDRYKYYPETPQGLAKIIQKAHKEGVCGLRSVSRLEHQMWEAVKDELNTLLKENGVDLSVVVEKQEGMYKSAPKRLTATTEKLEIGWKAWGKSILFAPELANNTFVVDGPETKECPTQNRAWNSLEVEDFGFGLTSTRMFLKVRESNTTECDSKIIGTAVKNNLAIHSDLSYWIESRLNDTWKLERAVLGEVKSCTWPETHTLWGDGILESDLIIPVTLAGPRSNHNRRPGYKTQNQGPWDEGRVEIDFDYCPGTTVTLSESCGHRGPATRTTTESGKLITDWCCRSCTLPPLRYQTDSGCWYGMEIRPQRHDEKTLVQSQVNAYNADMIDPFQLGLLVVFLATQEVLRKRWTAKISMPAILIALLVLVFGGITYTDVLRYVILVGAAFAESNSGGDVVHLALMATFKIQPVFMVASFLKARWTNQENILLMLAAVFFQMAYHDARQILLWEIPDVLNSLAVAWMILRAITFTTTSNVVVPLLALLTPGLRCLNLDVYRILLLMVGIGSLIREKRSAAAKKKGASLLCLALASTGLFNPMILAAGLIACDPNRKRGWPATEVMTAVGLMFAIVGGLAELDIDSMAIPMTIAGLMFAAFVISGKSTDMWIERTADISWESDAEITGSSERVDVRLDDDGNFQLMNDPGAPWKIWMLRMVCLAISAYTPWAILPSVVGFWITLQYTKRGGVLWDTPSPKEYKKGDTTTGVYRIMTRGLLGSYQAGAGVMVEGVFHTLWHTTKGAALMSGEGRLDPYWGSVKEDRLCYGGPWKLQHKWNGQDEVQMIVVEPGKNVKNVQTKPGVFKTPEGEIGAVTLDFPTGTSGSPIVDKNGDVIGLYGNGVIMPNGSYISAIVQGERMDEPIPAGFEPEMLRKKQITVLDLHPGAGKTRRILPQIIKEAINRRLRTAVLAPTRVVAAEMAEALRGLPIRYQTSAVPREHNGNEIVDVMCHATLTHRLMSPHRVPNYNLFVMDEAHFTDPASIAARGYISTKVELGEAAAIFMTATPPGTSDPFPESNSPISDLQTEIPDRAWNSGYEWITEYTGKTVWFVPSVKMGNEIALCLQRAGKKVVQLNRKSYETEYPKCKNDDWDFVITTDISEMGANFKASRVIDSRKSVKPTIITEGEGRVILGEPSAVTAASAAQRRGRIGRNPSQVGDEYCYGGHTNEDDSNFAHWTEARIMLDNINMPNGLVAQFYQPEREKVYTMDGEYRLRGEERKNFLELLRTADLPVWLAYKVAAAGVSYHDRRWCFDGPRTNTILEDNNEVEVITKLGERKILRPRWIDARVYSDHQALKAFKDFASGKRSQIGLIEVLGKMPEHFMGKTWEALDTMYVVATAEKGGRAHRMALEELPDALQTIALIALLSVMTMGVFFLLMQRKGIGKIGLGGAVLGVATFFCWMAEVPGTKIAGMLLLSLLLMIVLIPEPEKQRSQTDNQLAVFLICVMTLVSAVAANEMGWLDKTKSDISSLFGQRIEVKENFSMGEFLLDLRPATAWSLYAVTTAVLTPLLKHLITSDYINTSLTSINVQASALFTLARGFPFVDVGVSALLLAAGCWGQVTLTVTVTAATLLFCHYAYMVPGWQAEAMRSAQRRTAAGIMKNAVVDGIVATDVPELERTTPIMQKKVGQIMLILVSLAAVVVNPSVKTVREAGILITAAAVTLWENGASSVWNATTAIGLCHIMRGGWLSCLSITWTLIKNMEKPGLKRGGAKGRTLGEVWKERLNQMTKEEFTRYRKEAIIEVDRSAAKHARKEGNVTGGHPVSRGTAKLRWLVERRFLEPVGKVIDLGCGRGGWCYYMATQKRVQEVRGYTKGGPGHEEPQLVQSYGWNIVTMKSGVDVFYRPSECCDTLLCDIGESSSSAEVEEHRTIRVLEMVEDWLHRGPREFCVKVLCPYMPKVIEKMELLQRRYGGGLVRNPLSRNSTHEMYWVSRASGNVVHSVNMTSQVLLGRMEKRTWKGPQYEEDVNLGSGTRAVGKPLLNSDTSKIKNRIERLRREYSSTWHHDENHPYRTWNYHGSYDVKPTGSASSLVNGVVRLLSKPWDTITNVTTMAMTDTTPFGQQRVFKEKVDTKAPEPPEGVKYVLNETTNWLWAFLAREKRPRMCSREEFIRKVNSNAALGAMFEEQNQWRSAREAVEDPKFWEMVDEEREAHLRGECHTCIYNMMGKREKKPGEFGKAKGSRAIWFMWLGARFLEFEALGFLNEDHWLGRKNSGGGVEGLGLQKLGYILREVGTRPGGKIYADDTAGWDTRITRADLENEAKVLELLDGEHRRLARAIIELTYRHKVVKVMRPAADGRTVMDVISREDQRGSGQVVTYALNTFTNLAVQLVRMMEGEGVIGPDDVEKLTKGKGPKVRTWLFENGEERLSRMAVSGDDCVVKPLDDRFATSLHFLNAMSKVRKDIQEWKPSTGWYDWQQVPFCSNHFTELIMKDGRTLVVPCRGQDELVGRARISPGAGWNVRDTACLAKSYAQMWLLLYFHRRDLRLMANAICSAVPVNWVPTGRTTWSIHAGGEWMTTEDMLEVWNRVWIEENEWMEDKTPVEKWSDVPYSGKREDIWCGSLIGTRARATWAENIQVAINQVRAIIGDEKYVDYMSSLKRYEDTTLVEDTVL

>AHW48685.1 polyprotein [West Nile virus]

MSKKPGGPGKSRAVNMLKRGMPRVLSLIGLKRAMLSLIDGKGPIRFVLALLAFFRFTAIAPTRAVLDRWRGVNKQTAMKHLLSFKKELGTLTSAINRRSSKQKKRGGKTGIAVMIGLIASVGAVTLSNFQGKVMMTVNATDVTDVITIPTAAGKNLCIVRAMDVGYMCDDTITYECPVLSAGNDPEDIDCWCTKSAVYVRYGRCTKTRHSRRSRRSLTVQTHGESTLANKKGAWMDSTKATRYLVKTESWILRNPGYALVAAVIGWMLGSNTMQRVVFVVLLLLVAPAYSFNCLGMSNRDFLEGVSGATWVDLVLEGDSCVTIMSKDKPTIDVKMMNMEAANLAEVRSYCYLATVSDLSTKAACPTMGEAHNDKRADPAFVCRQGVVDRGWGNGCGLFGKGSIDTCAKFACSTKAIGRTILKENIKYEVAIFVHGPTTVESHGNYSTQAGATQAGRFSITPAAPSYTLKLGEYGEVTVDCEPRSGIDTNAYYVMTVGTKTFLVHREWFMDLNLPWSSAGSTVWRNRETLMEFEEPHATKQSVIALGSQEGALHQALAGAIPVEFSSNTVKLTSGHLKCRVKMEKLQLKGTTYGVCSKAFKFLGTPADTGHGTVVLELQYTGTDGPCKVPISSVASLNDLTPVGRLVTVNPFVSVATANAKVLIELEPPFGDSYIVVGRGEQQINHHWHKSGSSIGKAFTTTLKGAQRLAALGDTAWDFGSVGGVFTSVGKAVHQVFGGAFRSLFGGMSWITQGLLGALLLWMGINARDRSIALTFLAVGGVLLFLSVNVHADTGCAIDISRQELRCGSGVFIHNDVEAWMDRYKYYPETPQGLAKIIQKAHKEGVCGLRSVSRLEHQMWEAVKDELNTLLKENGVDLSVVVEKQEGMYKSAPKRLTATTEKLEIGWKAWGKSILFAPELANNTFVVDGPETKECPTQNRAWNSLEVEDFGFGLTSTRMFLKVRESNTTECDSKIIGTAVKNNLAIHSDLSYWIESRLNDTWKLERAVLGEVKSCTWPETHTLWGDGILESDLIIPVTLAGPRSNHNRRPGYKTQNQGPWDEGRVEIDFDYCPGTTVTLSESCGHRGPATRTTTESGKLITDWCCRSCTLPPLRYQTDSGCWYGMEIRPQRHDEKTLVQSQVNAYNADMIDPFQLGLLVVFLATQEVLRKRWTAKISMPAILIALLVLVFGGITYTDVLRYVILVGAAFAESNSGGDVVHLALMATFKIQPVFMVASFLKARWTNQENILLMLAAVFFQMAYHDARQILLWEIPDVLNSLAVAWMILRAITFTTTSNVVVPLLALLTPGLRCLNLDVYRILLLMVGIGSLIREKRSAAAKKKGASLLCLALASTGLFNPMILAAGLIACDPNRKRGWPATEVMTAVGLMFAIVGGLAELDIDSMAIPMTIAGLMFAAFVISGKSTDMWIERTADISWESDAEITGSSERVDVRLDDDGNFQLMNDPGAPWKIWMLRMVCLAISAYTPWAILPSVVGFWITLQYTKRGGVLWDTPSPKEYKKGDTTTGVYRIMTRGLLGSYQAGAGVMVEGVFHTLWHTTKGAALMSGEGRLDPYWGSVKEDRLCYGGPWKLQHKWNGQDEVQMIVVEPGKNVKNVQTKPGVFKTPEGEIGAVTLDFPTGTSGSPIVDKNGDVIGLYGNGVIMPNGSYISAIVQGERMDEPIPAGFEPEMLRKKQITVLDLHPGAGKTRRILPQIIKEAINRRLRTAVLAPTRVVAAEMAEALRGLPIRYQTSAVPREHNGNEIVDVMCHATLTHRLMSPHRVPNYNLFVMDEAHFTDPASIAARGYISTKVELGEAAAIFMTATPPGTSDPFPESNSPISDLQTEIPDRAWNSGYEWITEYTGKTVWFVPSVKMGNEIALCLQRAGKKVVQLNRKSYETEYPKCKNDDWDFVITTDISEMGANFKASRVIDSRKSVKPTIITEGEGRVILGEPSAVTAASAAQRRGRIGRNPSQVGDEYCYGGHTNEDDSNFAHWTEARIMLDNINMPNGLIAQFYQPEREKVYTMDGEYRLRGEERKNFLELLRTADLPVWLAYKVAAAGVSYHDRRWCFDGPRTNTVLEDNNEVEVITKLGERKILRPRWIDARVYSDHQALKAFKDFASGKRSQIGLIEVLGKMPEHFMGKTWEALDTMYVVATAEKGGRAHRMALEELPDALQTIALIALLSVMTMGVFFLLMQRKGIGKIGLGGAVLGVATFFCWMAEVPGTKIAGMLLLSLLLMIVLIPEPEKQRSQTDNQLAVFLICVMTLVSAVAANEMGWLDKTKSDISSLFGQRIEVKENFSMGEFLLDLRPATAWSLYAVTTAVLTPLLKHLITSDYINTSLTSINVQASALFTLARGFPFVDVGVSALLLAAGCWGQVTLTVTVTAATLLFCHYAYMVPGWQAEAMRSAQRRTAAGIMKNAVVDGIVATDVPELERTTPIMQKKVGQIMLILVSLAAVVVNPSVKTVREAGILITAAAVTLWENGASSVWNATTAIGLCHIMRGGWLSCLSITWTLIKNMEKPGLKRGGAKGRTLGEVWKERLNQMTKEEFTRYRKEAIIEVDRSAAKHARKEGNVTGGHPVSRGTAKLRWLVERRFLEPVGKVIDLGCGRGGWCYYMATQKRVQEVRGYTKGGPGHEEPQLVQSYGWNIVTMKSGVDVFYRPSECCDTLLCDIGESSSSAEVEEHRTIRVLEMVEDWLHRGPREFCVKVLCPYMPKVIEKMELLQRRYGGGLVRNPLSRNSTHEMYWVSRASGNVVHSVNMTSQVLLGRMEKRTWKGPQYEEDVNLGSGTRAVGKPLLNSDTSKIKNRIERLRREYSSTWHHDENHPYRTWNYHGSYDVKPTGSASSLVNGVVRLLSKPWDTITNVTTMAMTDTTPFGQQRVFKEKVDTKAPEPPEGVKYVLNETTNWLWAFLAREKRPRMCSREEFIRKVNSNAALGAMFEEQNQWRSAREAVEDPKFWEMVDEEREAHLRGECHTCIYNMMGKREKKPGEFGKAKGSRAIWFMWLGARFLEFEALGFLNEDHWLGRKNSGGGVEGLGLQKLGYILREVGTRPGGKIYADDTAGWDTRITRADLENEAKVLELLDGEHRRLARAIIELTYRHKVVKVMRPAADGRTVMDVISREDQRGSGQVVTYALNTFTNLAVQLVRMMEGEGVIGPDDVEKLTKGKGPKVRTWLFENGEERLSRMAVSGDDCVVKPLDDRFATSLHFLNAMSKVRKDIQEWKPSTGWYDWQQVPFCSNHFTELIMKDGRTLVVPCRGQDELVGRARISPGAGWNVRDTACLAKSYAQMWLLLYFHRRDLRLMANAICSAVPVNWVPTGRTTWSIHAGGEWMTTEDMLEVWNRVWIEENEWMEDKTPVEKWSDVPYSGKREDIWCGSLIGTRARATWAENIQVAINQVRAIIGDEKYVDYMSSLKRYEDTTLVEDTVL

>AHW48584.1 polyprotein [West Nile virus]

MSKKPGGPGKSRAVNMLKRGMPRVLSLIGLKRAMLSLIDGKGPIRFVLALLAFFRFTAIAPTRAVLDRWRGVNKQTAMKHLLSFKKELGTLTSAINRRSSKQKKRGGKTGIAVMIGLIASVGAVTLSNFQGKVMMTVNATDVTDVITIPTAAGKNLCIVRAMDVGYMCDDTITYECPVLSAGNDPEDIDCWCTKSAVYVRYGRCTKTRHSRRSRRSLTVQTHGESTLANKKGAWMDSTKATRYLVKTESWILRNPGYALVAAVIGWMLGSNTMQRVVFVVLLLLVAPAYSFNCLGMSNRDFLEGVSGATWVDLVLEGDSCVTIMSKDKPTIDVKMMNMEAANLAEVRSYCYLATVSDLSTKAACPTMGEAHNDKRADPAFVCRQGVVDRGWGNGCGLFGKGSIDTCAKFACSTKAIGRTILKENIKYEVAIFVHGPTTVESHGNYSTQAGATQAGRFSITPAAPSYTLKLGEYGEVTVDCEPRSGIDTNAYYVMTVGTKTFLVHREWFMDLNLPWSSAGSTVWRNRETLMEFEEPHATKQSVIALGSQEGALHQALAGAIPVEFSSNTVKLTSGHLKCRVKMEKLQLKGTTYGVCSKAFKFLGTPADTGHGTVVLELQYTGTDGPCKVPISSVASLNDLTPVGRLVTVNPFVSVATANAKVLIELEPPFGDSYIVVGRGEQQINHHWHKSGSSIGKAFTTTLKGAQRLAALGDTAWDFGSVGGVFTSVGKAVHQVFGGAFRSLFGGMSWITQGLLGALLLWMGINARDRSIALTFLAVGGVLLFLSVNVHADTGCAIDISRQELRCGSGVFIHNDVEAWMDRYKYYPETPQGLAKIIQKAHKEGVCGLRSVSRLEHQMWEAVKDELNTLLKENGVDLSVVVEKQEGMYKSAPKRLTATTEKLEIGWKAWGKSILFAPELANNTFVVDGPETKECPTQNRAWNSLEVEDFGFGLTSTRMFLKVRESNTTECDSKIIGTAVKNNLAIHSDLSYWIESRLNDTWKLERAVLGEVKSCTWPETHTLWGDGILESDLIIPVTLAGPRSNHNRRPGYKTQNQGPWDEGRVEIDFDYCPGTTVTLSESCGHRGPATRTTTESGKLITDWCCRSCTLPPLRYQTDSGCWYGMEIRPQRHDEKTLVQSQVNAYNADMIDPFQLGLLVVFLATQEVLRKRWTAKISMPAILIALLVLVFGGITYTDVLRYVILVGAAFAESNSGGDVVHLALMATFKIQPVFMVASFLKARWTNQENILLMLAAVFFQMAYHDARQILLWEIPDVLNSLAVAWMILRAITFTTTSNVVVPLLALLTPGLRCLNLDVYRILLLMVGIGSLIREKRSAAAKKKGASLLCLALASTGLFNPMILAAGLIACDPNRKRGWPATEVMTAVGLMFAIVGGLAELDIDSMAIPMTIAGLMFAAFVISGKSTDMWIERTADISWESDAEITGSSERVDVRLDDDGNFQLMNDPGAPWKIWMLRMVCLAISAYTPWAILPSVVGFWITLQYTKRGGVLWDTPSPKEYKKGDTTTGVYRIMTRGLLGSYQAGAGVMVEGVFHTLWHTTKGAALMSGEGRLDPYWGSVKEDRLCYGGPWKLQHKWNGQDEVQMIVVEPGKNVKNVQTKPGVFKTPEGEIGAVTLDFPTGTSGSPIVDKNGDVIGLYGNGVIMPNGSYISAIVQGERMDEPIPAGFEPEMLRKKQITVLDLHPGAGKTRRILPQIIKEAINRRLRTAVLAPTRVVAAEMAEALRGLPIRYQTSAVPREHNGNEIVDVMCHATLTHRLMSPHRVPNYNLFVMDEAHFTDPASIAARGYISTKVELGEAAAIFMTATPPGTSDPFPESNSPISDLQTEIPDRAWNSGYEWITEYTGKTVWFVPSVKMGNEIALCLQRAGKKVVQLNRKSYETEYPKCKNDDWDFVITTDISEMGANFKASRVIDSRKSVKPTIITEGEGRVILGEPSAVTAASAAQRRGRIGRNPSQVGDEYCYGGHTNEDDSNFAHWTEARIMLDNINMPNGLIAQFYQPEREKVYTMDGEYRLRGEERKNFLELLRTADLPVWLAYKVAAAGVSYHDRRWCFDGPRTNTILEDNNEVEVITKLGERKILRPRWIDARVYSDHQALKAFKDFASGKRSQIGLIEVLGKMPEHFMGKTWEALDTMYVVATAEKGGRAHRMALEELPDALQTIALIALLSVMTMGVFFLLMQRKGIGKIGLGGAVLGVATFFCWMAEVPGTKIAGMLLLSLLLMIVLIPEPEKQRSQTDNQLAVFLICVMTLVSAVAANEMGWLDKTKSDISSLFGQRIEVKENFSMGEFLLDLRPATAWSLYAVTTAVLTPLLKHLITSDYINTSLTSINVQASALFTLARGFPFVDVGVSALLLAAGCWGQVTLTVTVTAATLLFCHYAYMVPGWQAEAMRSAQRRTAAGIMKNAVVDGIVATDVPELERTTPIMQKKVGQIMLILVSLAAVVVNPSVKTVREAGILITAAAVTLWENGASSVWNATTAIGLCHIMRGGWLSCLSITWTLVKNMEKPGLKRGGAKGRTLGEVWKERLNQMTKEEFTRYRKEAIIEVDRSAAKHARKEGNVTGGHPVSRGTAKLRWLVERRFLEPVGKVIDLGCGRGGWCYYMATQKRVQEVRGYTKGGPGHEEPQLVQSYGWNIVTMKSGVDVFYRPSECCDTLLCDIGESSSSAEVEEHRTIRVLEMVEDWLHRGPREFCVKVLCPYMPKVIEKMELLQRRYGGGLVRNPLSRNSTHEMYWVSRASGNVVHSVNMTSQVLLGRMEKRTWKGPQYEEDVNLGSGTRAVGKPLLNSDTSKIKNRIERLRREYSSTWHHDENHPYRTWNYHGSYDVKPTGSASSLVNGVVRLLSKPWDTITNVTTMAMTDTTPFGQQRVFKEKVDTKAPEPPEGVKYVLNETTNWLWAFLAREKRPRMCSREEFIRKVNSNAALGAMFEEQNQWRSAREAVEDPKFWEMVDEEREAHLRGECHTCIYNMMGKREKKPGEFGKAKGSRAIWFMWLGARFLEFEALGFLNEDHWLGRKNSGGGVEGLGLQKLGYILREVGTRPGGKIYADDTAGWDTRITRADLENEAKVLELLDGEHRRLARAIIELTYRHKVVKVMRPAADGRTVMDVISREDQRGSGQVVTYALNTFTNLAVQLVRMMEGEGVIGPDDVEKLTKGKGPKVRTWLFENGEERLSRMAVSGDDCVVKPLDDRFATSLHFLNAMSKVRKDIQEWKPSTGWYDWQQVPFCSNHFTELIMKDGRTLVVPCRGQDELVGRARISPGAGWNVRDTACLAKSYAQMWLLLYFHRRDLRLMANAICSAVPVNWVPTGRTTWSIHAGGEWMTTEDMLEVWNRVWIEENEWMEDKTPVEKWSDVPYSGKREDIWCGSLIGTRARATWAENIQVAINQVRAIIGDEKYVDYMSSLKRYEDTTLVEDTVL

>ANW69118.1 genome polyprotein [West Nile virus]

MSKKPGGPGKSRAVNMLKRGMPRVLSLIGLKRAMLSLIDGKGPIRFVLALLAFFRFTAIAPTRAVLDRWRGVNKQTAMKHLLSFKKELGTLTSAINRRSSKQKKRGGKTGIAVMIGLIASVGAVTLSNFQGKVMMTVNATDVTDVITIPTAAGKNLCIVRAMDVGYMCDDTITYECPVLSAGNDPEDIDCWCTKSAVYVRYGRCTKTRHSRRSRRSLTVQTHGESTLANKKGAWMDSTKATRYLVKTESWILRNPGYALVAAVIGWMLGSNTMQRVVFVVLLLLVAPAYSFNCLGMSNRDFLEGVSGATWVDLVLEGDSCVTIMSKDKPTIDVKMMNMEAANLAEVRSYCYLATVSDLSTKAACPTMGEAHNDKRADPAFVCRQGVVDRGWGNGCGLFGKGSIDTCAKFACSTKAIGRTILKENIKYEVAIFVHGPTTVESHGNYSTQVGATQAGRFSITPAAPSYTLKLGEYGEVTVDCEPRSGIDTNAYYVMTVGTKTFLVHREWFMDLNLPWSSAGSTVWRNRETLMEFEEPHATKQSVIALGSQEGALHQALAGAIPVEFSSNTVKLTSGHLKCRVKMEKLQLKGTTYGVCSKAFKFLGTPADTGHGTVVLELQYTGTDGPCKVPISSVASLNDLTPVGRLVTVNPFVSVATANAKVLIELEPPFGDSYIVVGRGEQQINHHWHKSGSSIGKAFTTTLKGAQRLAALGDTAWDFGSVGGVFTSVGKAVHQVFGGAFRSLFGGMSWITQGLLGALLLWMGINARDRSIALTFLAVGGVLLFLSVNVHADTGCAIDISRQELRCGSGVFIHNDVEAWMDRYKYYPETPQGLAKIIQKAHKEGVCGLRSVSRLEHQMWEAVKDELNTLLKENGVDLSVVVEKQEGMYKSAPKRLTATTEKLEIGWKAWGKSILFAPELANNTFVVDGPETKECPTQNRAWNSLEVEDFGFGLTSTRMFLKVRESNTTECDSKIIGTAVKNNLAIHSDLSYWIESRLNDTWKLERAVLGEVKSCTWPETHTLWGDGILESDLIIPVTLAGPRSNHNRRPGYKTQNQGPWDEGRVEIDFDYCPGTTVTLSESCGHRGPATRTTTESGKLITDWCCRSCTLPPLRYQTDSGCWYGMEIRPQRHDEKTLVQSQVNAYNADMIDPFQLGLLVVFLATQEVLRKRWTAKISMPAILIALLVLVFGGITYTDVLRYVILVGAAFAESNSGGDVVHLALMATFKIQPVFMVASFLKARWTNQENILLMLAAVFFQMAYHDARQILLWEIPDVLNSLAVAWMILRAITFTTTSNVVVPLLALLTPGLRCLNLDVYRILLLMVGIGSLIREKRSAAAKKKGASLLCLALASTGLFNPMILAAGLIACDPNRKRGWPATEVMTAVGLMFAIVGGLAELDIDSMAIPMTIAGLMFAAFVISGKSTDMWIERTADISWESDAEITGSSERVDVRLDDDGNFQLMNDPGAPWKIWMLRMVCLAISAYTPWAILPSVVGFWITLQYTKRGGVLWDTPSPKEYKKGDTTTGVYRIMTRGLLGSYQAGAGVMVEGVFHTLWHTTKGAALMSGEGRLDPYWGSVKEDRLCYGGPWKLQHKWNGQDEVQMIVVEPGKNVKNVQTKPGVFKTPEGEIGAVTLDFPTGTSGSPIVDKNGDVIGLYGNGVIMPNGSYISAIVQGERMDEPIPAGFEPEMLRKKQITVLDFHPGAGKTRRILPQIIKEAINRRLRTAVLAPTRVVAAEMAEALRGLPIRYQTSAVPREHNGNEIVDVMCHATLTHRLMSPHRVPNYNLFVMDEAHFTDPASIAARGYISTKVELGEAAAIFMTATPPGTSDPFPESNSPISDLQTEIPDRAWNSGYEWITEYTGKTVWFVPSVKMGNEIALCLQRAGKKVVQLNRKSYETEYPKCKNDDWDFVITTDISEMGANFKASRVIDSRKSVKPTIITEGEGRVILGEPSAVTAASAAQRRGRIGRNPSQVGDEYCYGGHTNEDDSNFAHWTEARIMLDNINMPNGLIAQFYQPEREKVYTMDGEYRLRGEERKNFLELLRTADLPVWLAYKVAAAGVSYHDRRWCFDGPRTNTILEDNNEVEVITKLGERKILRPRWIDARVYSDHQALKAFKDFASGKRSQIGLIEVLGKMPEHFMGKTWEALDTMYVVATAEKGGRAHRMALEELPDALQTIALIALLSVMTMGVFFLLMQRKGIGKIGLGGAVLGVATFFCWMAEVPGTKIAGMLLLSLLLMIVLIPEPEKQRSQTDNQLAVFLICVMTLVSAVAANEMGWLDKTKSDISSLFGQRIEVKENFSMGEFLLDLRPATAWSLYAVTTAVLTPLLKHLITSDYINTSLTSINVQASALFTLARGFPFVDVGVSALLLAAGCWGQVTLTVTVTAATLLFCHYAYMVPGWQAEAMRSAQRRTAAGIMKNAVVDGIVATDVPELERTTPIMQKKVGQIMLILVSLAAVVVNPSVKTVREAGILITAAAVTLWENGASSVWNATTAIGLCHIMRGGWLSCLSITWTLIKNMEKPGLKRGGAKGRTLGEVWKERLNQMTKEEFTRYRKEAIIEVDRSAAKHARKEGNVTGGHPVSRGTAKLRWLVERRFLEPVGKVIDLGCGRGGWCYYMATQKRVQEVRGYTKGGPGHEEPQLVQSYGWNIVTMKSGVDVFYRPSECCDTLLCDIGESSSSAEVEEHRTIRVLEMVEDWLHRGPREFCVKVLCPYMPKVIEKMELLQRRYGGGLVRNPLSRNSTHEMYWVSRASGNVVHSVNMTSQVLLGRMEKRTWKGPQYEEDVNLGSGTRAVGKPLLNSDTSKIKNRIERLRREYSSTWHHDENHPYRTWNYHGSYDVKPTGSASSLVNGVVRLLSKPWDTITNVTTMAMTDTTPFGQQRVFKEKVDTKAPEPPEGVKYVLNETTNWLWAFLAREKRPRMCSREEFIRKVNSNAALGAMFEEQNQWRSAREAVEDPKFWEMVDEEREAHLRGECHTCIYNMMGKREKKPGEFGKAKGSRAIWFMWLGARFLEFEALGFLNEDHWLGRKNSGGGVEGLGLQKLGYILREVGTRPGGKIYADDTAGWDTRITRADLENEAKVLELLDGEHRRLARAIIELTYRHKVVKVMRPAADGRTVMDVISREDQRGSGQVVTYALNTFTNLAVQLVRMMEGEGVIGPDDVEKLTKGKGPKVRTWLFENGEERLSRMAVSGDDCVVKPLDDRFATSLHFLNAMSKVRKDIQEWKPSTGWYDWQQVPFCSNHFTELIMKDGRTLVVPCRGQDELVGRARISPGAGWNVRDTACLAKSYAQMWLLLYFHRRDLRLMANAICSAVPVNWVPTGRTTWSIHAGGEWMTTEDMLEVWNRVWIEENEWMEDKTPVEKWSDVPYSGKREDIWCGSLIGTRARATWAENIQVAINQVRAIIGDEKYVDYMSSLKRYEDTTLVEDTVL

>ADK62433.1 polyprotein [West Nile virus]

MSKKPGGPGKSRAVNMLKRGMPRVLSLIGLKRAMLSLIDGKGPIRFVLALLAFFRFTAIAPTRAVLDRWRGVNKQTAMKHLLSFKKELGTLTSAINRRSSKQKKRGGKTGIAVMIGLIASVGAVTLSNFQGKVMMTVNATDVTDVITIPTAAGKNLCIVRAMDVGYMCDDTITYECPVLSAGNDPEDIDCWCTKSAVYVRYGRCTKTRHSRRSRRSLTVQTHGESTLANKKGAWMDSTKATRYLVKTESWILRNPGYALVAAVIGWMLGSNTMQRVVFVVLLLLVAPAYSFNCLGMSNRDFLEGVSGATWVDLVLEGDSCVTIMSKDKPTIDVKMMNMEAANLAEVRSYCYLATVSDLSTKAACPTMGEAHNDKRADPAFVCRQGVVDRGWGNGCGLFGKGSIDTCAKFACSTKAIGRTILKENIKYEVAIFVHGPTTVESHGNYSTQAGATQAGRFSITPAAPSYTLKLGEYGEVTVDCEPRSGIDTNAYYVMTVGTKTFLVHREWFMDLNLPWSSAGSTVWRNRETLMEFEEPHATKQSVIALGSQEGALHQALAGAIPVEFSSNTVKLTSGHLKCRVKMEKLQLKGTTYGVCSKAFKFLGTPADTGHGTVVLELQYTGTDGPCKVPISSVASLNDLTPVGRLVTVNPFVSVATANAKVLIELEPPFGDSYIVVGRGEQQINHHWHKSGSSIGKAFTTTLKGAQRLAALGDTAWDFGSVGGVFTSVGKAVHQVFGGAFRSLFGGMSWITQGLLGALLLWMGINARDRSIALTFLAVGGVLLFLSVNVHADTGCAIDISRQELRCGSGVFIHNDVEAWMDRYKYYPETPQGLAKIIQKAHKEGVCGLRSVSRLEHQMWEAVKDELNTLLKENGVDLSVVVEKQEGMYKSAPKRLTATTEKLEIGWKAWGKSILFAPELANNTFVVDGPETKECPTQNRAWNSLEVEDFGFGLTSTRMFLKVRESNTTECDSKIIGTAVKNNLAIHSDLSYWIESRLNDTWKLERAVLGEVKSCTWPETHTLWGDGILESDLIIPVTLAGPRSNHNRRPGYKTQNQGPWDEGRVEIDFDYCPGTTVTLSESCGHRGPATRTTTESGKLITDWCCRSCTLPPLRYQTDSGCWYGMEIRPQRHDEKTLVQSQVNAYNADMIDPFQLGLLVVFLATQEVLRKRWTAKISMPAILIALLVLVFGGITYTDVLRYVILVGAAFAESNSGGDVVHLALMATFKIQPVFMVASFLKARWTNQENILLMLAAVFFQMAYHDARQILLWEIPDVLNSLAVAWMILRAITFTTTSNVVVPLLALLTPGLRCLNLDVYRILLLMVGIGSLIREKRSAAAKKKGASLLCLALASTGLFNPMILAAGLIACDPNRKRGWPATEVMTAVGLMFAIVGGLAELDIDSMAIPMTIAGLMFAAFVISGKSTDMWIERTADISWESDAEITGSSERVDVRLDDDGNFQLMNDPGAPWKIWMLRMVCLAISAYTPWAILPSVVGFWITLQYTKRGGVLWDTPSPKEYKKGDTTTGVYRIMTRGLLGSYQAGAGVMVEGVFHTLWHTTKGAALMSGEGRLDPYWGSVKEDRLCYGGPWKLQHKWNGQDEVQMIVVEPGKNVKNVQTKPGVFKTPEGEIGAVTLDFPTGTSGSPIVDKNGDVIGLYGNGVIMPNGSYISAIVQGERMDEPVPAGFEPEMLRKKQITVLDLHPGAGKTRRILPQIIKEAINRRLRTAVLAPTRVVAAEMAEALRGLPIRYQTSAVPREHNGNEIVDVMCHATLTHRLMSPHRVPNYNLFVMDEAHFTDPASIAARGYISTKVELGEAAAIFMTATPPGTSDPFPESNSPISDLQTEIPDRAWNSGYEWITEYTGKTVWFVPSVKMGNEIALCLQRAGKKVVQLNRKSYETEYPKCKNDDWDFVITTDISEMGANFKASRVIDSRKSVKPTIITEGEGRVILGEPSAVTAASAAQRRGRIGRNPSQVGDEYCYGGHTNEDDSNFAHWTEARIMLDNINMPNGLIAQFYQPEREKVYTMDGEYRLRGEERKNFLELLRTADLPVWLAYKVAAAGVSYHDRRWCFDGPRTNTILEDNNEVEVITKLGERKILRPRWIDARVYSDHQALKAFKDFASGKRSQIGLIEVLGKMPEHFMGKTWEALDTMYVVATAEKGGRAHRMALEELPDALQTIALIALLSVMTMGVFFLLMQRKGIGKIGLGGAVLGVATFFCWMAEVPGTKIAGMLLLSLLLMIVLIPEPEKQRSQTDNQLAVFLICVMTLVSAVAANEMGWLDKTKSDISSLFGQRIEVKENFSMGEFLLDLRPATAWSLYAVTTAVLTPLLKHLITSDYINTSLTSINVQASALFTLARGFPFVDVGVSALLLAAGCWGQVTLTVTVTAATLLFCHYAYMVPGWQAEAMRSAQRRTAAGIMKNAVVDGIVATDVPELERTTPIMQKKVGQIMLILVSLAAVVVNPSVKTVREAGILITAAAVTLWENGASSVWNATTAIGLCHIMRGGWLSCLSITWTLIKNMEKPGLKRGGAKGRTLGEVWKERLNQMTKEEFTRYRKEAIIEVDRSAAKHARKEGNVTGGHPVSRGTAKLRWLVERRFLEPVGKVIDLGCGRGGWCYYMATQKRVQEVRGYTKGGPGHEEPQLVQSYGWNIVTMKSGVDVFYRPSECCDTLLCDIGESSSSAEVEEHRTIRVLEMVEDWLHRGPREFCVKVLCPYMPKVIEKMELLQRRYGGGLVRNPLSRNSTHEMYWVSRASGNVVHSVNMTSQVLLGRMEKRTWKGPQYEEDVNLGSGTRAVGKPLLNSDTSKIKNRIERLRREYSSTWHHDENHPYRTWNYHGSYDVKPTGSASSLVNGVVRLLSKPWDTITNVTTMAMTDTTPFGQQRVFKEKVDTKAPEPPEGVKYVLNETTNWLWAFLAREKRPRMCSREEFIRKVNSNAALGAMFEEQNQWRSAREAVEDPKFWEMVDEEREAHLRGECHTCIYNMMGKREKKPGEFGKAKGSRAIWFMWLGARFLEFEALGFLNEDHWLGRKNSGGGVEGLGLQKLGYILREVGTRPGGKIYADDTAGWDTRITRADLENEAKVLELLDGEHRRLARAIIELTYRHKVVKVMRPAADGRTVMDVISREDQRGSGQVVTYALNTFTNLAVQLVRMMEGEGVIGPDDVEKLTKGKGPKVRTWLFENGEERLSRMAVSGDDCVVKPLDDRFATSLHFLNAMSKVRKDIQEWKPSTGWYDWQQVPFCSNHFTELIMKDGRTLVVPCRGQDELVGRARISPGAGWNVRDTACLAKSYAQMWLLLYFHRRDLRLMANAICSAVPVNWVPTGRTTWSIHAGGEWMTTEDMLEVWNRVWIEENEWMEDKTPVEKWSDVPYSGKREDIWCGSLIGTRARATWAENIQVAINQVRAIIGDEKYVDYMSSLKRYEDTTLVEDTVL

>ABA54585.1 polyprotein precursor [West Nile virus]

MSKKPGGPGKSRAVNMLKRGMPRVLSLIGLKRAMLSLIDGKGPIRFVLALLAFFRFTAIAPTRAVLDRWRGVNKQTAMKHLLSFKKELGTLTSAINRRSSKQKKRGGKTGIAVMIGLIASVGAVTLSNFQGKVMMTVNATDVTDVITIPTAAGKNLCIVRAMDVGYMCDDTITYECPVLSAGNDPEDIDCWCTKSAVYVRYGRCTKTRHSRRSRRSLTVQTHGESTLANKKGAWMDSTKATRYLVKTESWILRNPGYALVAAVIGWMLGSNTMQRVVFVVLLLLVAPAYSFNCLGMSNRDFLEGVSGATWVDLVLEGDSCVTIMSKDKPTIDVKMMNMEAANLAEVRSYCYLATVSDLSTKAACPTMGEAHNDKRADPAFVCRQGVVDRGWGNGCGLFGKGSIDTCAKFACSTKAIGRTILKENIKYEVAIFVHGPTTVESHGNYSTQAGATQAGRFSITPAAPSYTLKLGEYGEVTVDCEPRSGIDTNAYYVMTVGTKTFLVHREWFMDLNLPWSSAGSTVWRNRETLMEFEEPHATKQSVIALGSQEGALHQALAGAIPVEFSSNTVKLTSGHLKCRVKMEKLQLKGTTYGVCSKAFKFLGTPADTGHGTVVLELQYTGTDGPCKVPISSVASLNDLTPVGRLVTVNPFVSVATANAKVLIELEPPFGDSYIVVGRGEQQINHHWHKSGSSIGKAFTTTLKGAQRLAALGDTAWDFGSVGGVFTSVGKAVHQVFGGAFRSLFGGMSWITQGLLGALLLWMGINARDRSIALTFLAVGGVLLFLSVNVHADTGCAIDISRQELRCGSGVFIHNDVEAWMDRYKYYPETPQGLAKIIQKAHKEGVCGLRSVSRLEHQMWEAVKDELNTLLKENGVDLSVVVEKQEGMYKSAPKRLTATTEKLEIGWKAWGKSILFAPELANNTFVVDGPETKECPTQNRAWNSLEVEDFGFGLTSTRMFLKVRESNTTECDSKIIGTAVKNNLAIHSDLSYWIESRLNDTWKLERAVLGEVKSCTWPETHTLWGDGILESDLIIPVTLAGPRSNHNRRPGYKTQNQGPWDEGRVEIDFDYCPGTTVTLSESCGHRGPATRTTTESGKLITDWCCRSCTLPPLRYQTDSGCWYGMEIRPQRHDEKTLVQSQVNAYNADMIDPFQLGLLVVFLATQEVLRKRWTAKISMPAILIALLVLVFGGITYTDVLRYVILVGAAFAESNSGGDVVHLALMATFKIQPVFMVASFLKARWTNQENILLMLAAVFFQMAYHDARQILLWEIPDVLNSLAVAWMILRAITFTTTSNVVVPLLALLTPGLRCLNLDVYRILLLMVGIGSLIREKRSAAAKKKGASLLCLALASTGLFNPMILAAGLIACDPNRKRGWPATEVMTAVGLMFAIVGGLAELDIDSMAIPMTIAGLMFAAFVISGKSTDMWIERTADISWESDAEITGSSERVDVRLDDDGNFQLMNDPGAPWKIWMLRMVCLAISAYTPWAILPSVVGFWITLQYTKRGGVLWDTPSPKEYKKGDTTTGVYRIMTRGLLGSYQAGAGVMVEGVFHTLWHTTKGAALMSGEGRLDPYWGSVKEDRLCYGGPWKLQHKWNGQDEVQMIVVEPGKNVKNVQTKPGVFKTPEGEIGAVTLDFPTGTSGSPIVDKNGDVIGLYGNGVIMPNGSYISAIVQGERMDEPIPAGFEPEMLRKKQITVLDLHPGAGKTRRILPQIIKEAINRRLRTAVLAPTRVVAAEMAEALRGLPIRYQTSAVPREHNGNEIVDVMCHATLTHRLMSPHRVPNYNLFVMDEAHFTDPASIAARGYISTKVELGEAAAIFMTATPPGTSDPFPESNSPISDLQTEIPDRAWNSGYEWITEYTGKTVWFVPSVKMGNEIALCLQRAGKKVVQLNRKSYETEYPKCKNDDWDFVITTDISEMGANFKASRVIDSRKSVKPTIITEGEGRVILGEPSAVTAASAAQRRGRIGRNPSQVGDEYCYGGHTNEDDSNFAHWTEARIMLDNINMPNGLIAQFYQPEREKVYTMDGEYRLRGEERKNFLELLKTADLPVWLAYKVAAAGVSYHDRRWCFDGPRTNTILEDNNEVEVITKLGERKILRPRWIDARVYSDHQALKAFKDFASGKRSQIGLIEVLGKMPEHFMGKTWEALDTMYVVATAEKGGRAHRMALEELPDALQTIALIALLSVMTMGVFFLLMQRKGIGKIGLGGAVLGVATFFCWMAEVPGTKIAGMLLLSLLLMIVLIPEPEKQRSQTDNQLAVFLICVMTLVSAVAANEMGWLDKTKSDISSLFGQRIEVKENFSMGEFLLDLRPATAWSLYAVTTAVLTPLLKHLITSDYINTSLTSINVQASALFTLARGFPFVDVGVSALLLAAGCWGQVTLTVTVTAATLLFCHYAYMVPGWQAEAMRSAQRRTAAGIMKNAVVDGIVATDVPELERTTPIMQKKVGQIMLILVSLAAVVVNPSVKTVREAGILITAAAVTLWENGASSVWNATTAIGLCHIMRGGWLSCLSITWTLIKNMEKPGLKRGGAKGRTLGEVWKERLNQMTKEEFTRYRKEAIIEVDRSAAKHARKEGNVTGGHPVSRGTAKLRWLVERRFLEPVGKVIDLGCGRGGWCYYMATQKRVQEVRGYTKGGPGHEEPQLVQSYGWNIVTMKSGVDVFYRPSECCDTLLCDIGESSSSAEVEEHRTIRVLEMVEDWLHRGPREFCVKVLCPYMPKVIEKMELLQRRYGGGLVRNPLSRNSTHEMYWVSRASGNVVHSVNMTSQVLLGRMEKRTWKGPQYEEDVNLGSGTRAVGKPLLNSDTSKIKNRIERLRREYSSTWHHDENHPYRTWNYHGSYDVKPTGSASSLVNGVVRLLSKPWDTITNVTTMAMTDTTPFGQQRVFKEKVDTKAPEPPEGVKYVLNETTNWLWAFLAREKRPRMCSREEFIRKVNSNAALGAMFEEQNQWRSAREAVEDPKFWEMVDEEREAHLRGECHTCIYNMMGKREKKPGEFGKAKGSRAIWFMWLGARFLEFEALGFLNEDHWLGRKNSGGGVEGLGLQKLGYILREVGTRPGGKIYADDTAGWDTRITRADLENEAKVLELLDGEHRRLARAIIELTYRHKVVKVMRPAADGRTVMDVISREDQRGSGQVVTYALNTFTNLAVQLVRMMEGEGVIGPDDVEKLTKGKGPKVRTWLFENGEERLSRMAVSGDDCVVKPLDDRFATSLHFLNAMSKVRKDIQEWKPSTGWYDWQQVPFCSNHFTELIMKDGRTLVVPCRGQDELVGRARISPGAGWNVRDTACLAKSYAQMWLLLYFHRRDLRLMANAICSAVPVNWVPTGRTTWSIHAGGEWMTTEDMLEVWNRVWIEENEWMEDKTPVEKWSDVPYSGKREDIWCGSLIGTRARATWAENIQVAINQVRAIIGDEKYVDYMSSLKRYEDTTLVEDTVL

>ANW69505.1 genome polyprotein [West Nile virus]

MSKKPGGPGKSRAVNMLKRGMPRVLSLIGLKRAMLSLIDGKGPIRFVLALLAFFRFTAIAPTRAVLDRWRGVNKQTAMKHLLSFKKELGTLTSAINRRSSKQKKRGGKTGIAVMIGLIASVGAVTLSNFQGKVMMTVNATDVTDVITIPTAAGKNLCIVRAMDVGYMCDDTITYECPVLSAGNDPEDIDCWCTKSAVYVRYGRCTKTRHSRRSRRSLTVQTHGESTLANKKGAWMDSTKATRYLVKTESWILRNPGYALVAAVIGWMLGSNTMQRVVFVVLLLLVAPAYSFNCLGMSNRDFLEGVSGATWVDLVLEGDSCVTIMSKDKPTIDVKMMNMEAANLAEVRSYCYLATVSDLSTKAACPTMGEAHNDKRADPAFVCRQGVVDRGWGNGCGLFGKGSIDTCAKFACSTKAIGRTILKENIKYEVAIFVHGPTTVESHGNYSTQAGATQAGRFSITPAAPSYTLKLGEYGEVTVDCEPRSGIDTNAYYVMTVGTKTFLVHREWFMDLNLPWSSAGSTVWRNRETLMEFEEPHATKQSVIALGSQEGALHQALAGAIPVEFSSNTVKLTSGHLKCRVKMEKLQLKGTTYGVCSKAFKFLGTPADTGHGTVVLELQYTGTDGPCKVPISSVASLNDLTPVGRLVTVNPFVSVATANAKVLIELEPPFGDSYIVVGRGEQQINHHWHKSGSSIGKAFTTTLKGAQRLAALGDTAWDFGSVGGVFTSVGKAVHQVFGGAFRSLFGGMSWITQGLLGALLLWMGINARDRSIALTFLAVGGVLLFLSVNVHADTGCAIDISRQELRCGSGVFIHNDVEAWMDRYKYYPETPQGLAKIIQKAHKEGVCGLRSVSRLEHQMWEAVKDELNTLLKENGVDLSVVVEKQEGMYKSAPKRLTATTEKLEIGWKAWGKSIIFAPELANNTFVVDGPETKECPTQNRAWNSLEVEDFGFGLTSTRMFLKVRESNTTECDSKIIGTAVKNNLAIHSDLSYWIESRLNDTWKLERAVLGEVKSCTWPETHTLWGDGILESDLIIPVTLAGPRSNHNRRPGYKTQNQGPWDEGRVEIDFDYCPGTTVTLSESCGHRGPATRTTTESGKLITDWCCRSCTLPPLRYQTDSGCWYGMEIRPQRHDEKTLVQSQVNAYNADMIDPFQLGLLVVFLATQEVLRKRWTAKISMPAILIALLVLVFGGITYTDVLRYVILVGAAFAESNSGGDVVHLALMATFKIQPVFMVASFLKARWTNQENILLMLAAVFFQMAYHDARQILLWEIPDVLNSLAVAWMILRAITFTTTSNVVVPLLALLTPGLRCLNLDVYRILLLMVGIGSLIREKRSAAAKKKGASLLCLALASTGLFNPMILAAGLIACDPNRKRGWPATEVMTAVGLMFAIVGGLAELDIDSMAIPMTIAGLMFAAFVISGKSTDMWIERTADISWESDAEITGSSERVDVRLDDDGNFQLMNDPGAPWKIWMLRMVCLAISAYTPWAILPSVVGFWITLQYTKRGGVLWDTPSPKEYKKGDTTTGVYRIMTRGLLGSYQAGAGVMVEGVFHTLWHTTKGAALMSGEGRLDPYWGSVKEDRLCYGGPWKLQHKWNGQDEVQMIVVEPGKNVKNVQTKPGVFKTPEGEIGAVTLDFPTGTSGSPIVDKNGDVIGLYGNGVIMPNGSYISAIVQGERMDEPIPAGFEPEMLRKKQITVLDLHPGAGKTRRILPQIIKEAINRRLRTAVLAPTRVVAAEMAEALRGLPIRYQTSAVPREHNGNEIVDVMCHATLTHRLMSPHRVPNYNLFVMDEAHFTDPASIAARGYISTKVELGEAAAIFMTATPPGTSDPFPESNSPISDLQTEIPDRAWNSGYEWITEYTGKTVWFVPSVKMGNEIALCLQRAGKKVVQLNRKSYETEYPKCKNDDWDFVITTDISEMGANFKASRVIDSRKSVKPTIITEGEGRVILGEPSAVTAASAAQRRGRIGRNPSQVGDEYCYGGHTNEDDSNFAHWTEARIMLDNINMPNGLIAQFYQPEREKVYTMDGEYRLRGEERKNFLELLRTADLPVWLAYKVAAAGVSYHDRRWCFDGPRTNTILEDNNEVEVITKLGERKILRPRWIDARVYSDHQALKAFKDFASGKRSQIGLIEVLGKMPEHFMGKTWEALDTMYVVATAEKGGRAHRMALEELPDALQTIALIALLSVMTMGVFFLLMQRKGIGKIGLGGAVLGVATFFCWMAEVPGTKIAGMLLLSLLLMIVLIPEPEKQRSQTDNQLAVFLICVMTLVSAVAANEMGWLDKTKSDISSLFGQRIEVKENFSMGEFLLDLRPATAWSLYAVTTAVLTPLLKHLITSDYINTSLTSINVQASALFTLARGFPFVDIGVSALLLAAGCWGQVTLTVTVTAATLLFCHYAYMVPGWQAEAMRSAQRRTAAGIMKNAVVDGIVATDVPELERTTPIMQKKVGQIMLILVSLAAVVVNPSVKTVREAGILITAAAVTLWENGASSVWNATTAIGLCHIMRGGWLSCLSITWTLIKNMEKPGLKRGGAKGRTLGEVWKERLNQMTKEEFTRYRKEAIIEVDRSAAKHARKEGNVTGGHPVSRGTAKLRWLVERRFLEPVGKVIDLGCGRGGWCYYMATQKRVQEVRGYTKGGPGHEEPQLVQSYGWNIVTMKSGVDVFYRPSECCDTLLCDIGESSSSAEVEEHRTIRVLEMVEDWLHRGPREFCVKVLCPYMPKVIEKMELLQRRYGGGLVRNPLSRNSTHEMYWVSRASGNVVHSVNMTSQVLLGRMEKRTWKGPQYEEDVNLGSGTRAVGKPLLNSDTSKIKNRIERLRREYSSTWHHDENHPYRTWNYHGSYDVKPTGSASSLVNGVVRLLSKPWDTITNVTTMAMTDTTPFGQQRVFKEKVDTKAPEPPEGVKYVLNETTNWLWAFLAREKRPRMCSREEFIRKVNSNAALGAMFEEQNQWRSAREAVEDPKFWEMVDEEREAHLRGECHTCIYNMMGKREKKPGEFGKAKGSRAIWFMWLGARFLEFEALGFLNEDHWLGRKNSGGGVEGLGLQKLGYILREVGTRPGGKIYADDTAGWDTRITRADLENEAKVLELLDGEHRRLARAIIELTYRHKVVKVMRPAADGRTVMDVISREDQRGSGQVVTYALNTFTNLAVQLVRMMEGEGVIGPDDVEKLTKGKGPKVRTWLFENGEERLSRMAVSGDDCVVKPLDDRFATSLHFLNAMSKVRKDIQEWKPSTGWYDWQQVPFCSNHFTELIMKDGRTLVVPCRGQDELVGRARISPGAGWNVRDTACLAKSYAQMWLLLYFHRRDLRLMANAICSAVPVNWVPTGRTTWSIHAGGEWMTTEDMLEVWNRVWIEENEWMEDKTPVEKWSDVPYSGKREDIWCGSLIGTRARATWAENIQVAINQVRAIIGDEKYVDYMSSLKRYEDTTLVEDTVL

>ADK62460.1 polyprotein [West Nile virus]

MSKKPGGPGKSRAVNMLKRGMPRVLSLIGLKRAMLSLIDGKGPIRFVLALLAFFRFTAIAPTRAVLDRWRGVNKQTAMKHLLSFKKELGTLTSAINRRSSKQKKRGGKTGIAVMIGLIASVGAVTLSNFQGKVMMTVNATDVTDVITIPTAAGKNLCIVRAMDVGYMCDDTITYECPVLSAGNDPEDIDCWCTKSAVYVRYGRCTKTRHSRRSRRSLTVQTHGESTLANKKGAWMDSTKATRYLVKTESWILRNPGYALVAAVIGWMLGSNTMQRVVFVVLLLLVAPAYSFNCLGMSNRDFLEGVSGATWVDLVLEGDSCVTIMSKDKPTIDVKMMNMEAANLAEVRSYCYLATVSDLSTKAACPTMGEAHNDKRADPAFVCRQGVVDRGWGNGCGLFGKGSIDTCAKFACSTKAIGRTILKENIKYEVAIFVHGPTTVESHGNYSTQAGATQAGRFSITPAAPSYTLKLGEYGEVTVDCEPRSGIDTNAYYVMTVGTKTFLVHREWFMDLNLPWSSAGSTVWRNRETLMEFEEPHATKQSVIALGSQEGALHQALAGAIPVEFSSNTVKLTSGHLKCRVKMEKLQLKGTTYGVCSKAFKFLGTPADTGHGTVVLELQYTGTDGPCKVPISSVASLNDLTPVGRLVTVNPFVSVATANAKVLIELEPPFGDSYIVVGRGEQQINHHWHKSGSSIGKAFTTTLKGAQRLAALGDTAWDFGSVGGVFTSVGKAVHQVFGGAFRSLFGGMSWITQGLLGALLLWMGINARDRSIALTFLAVGGVLLFLSVNVHADTGCAIDISRQELRCGSGVFIHNDVEAWMDRYKYYPETPQGLAKIIQKAHKEGVCGLRSVSRLEHQMWEAVKDELNTLLKENGVDLSVVVEKQEGMYKSAPKRLTATTEKLEIGWKAWGKSILFAPELANNTFVVDGPETKECPTQNRAWNSLEVEDFGFGLTSTRMFLKVRESNTTECDSKIIGTAVKNNLAIHSDLSYWIESRLNDTWKLERAVLGEVKSCTWPETHTLWGDGILESDLIIPVTLAGPRSNHNRRPGYKTQNQGPWDEGRVEIDFDYCPGTTVTLSESCGHRGPATRTTTESGKLITDWCCRSCTLPPLRYQTDSGCWYGMEIRPQRHDEKTLVQSQVNAYNADMIDPFQLGLLVVFLATQEVLRKRWTAKISMPAILIALLVLVFGGITYTDVLRYVILVGAAFAESNSGGDVVHLALMATFKIQPVFMVASFLKARWTNQENILLMLAAVFFQMAYHDARQILLWEIPDVLNSLAVAWMILRAITFTTTSNVVVPLLALLTPGLRCLNLDVYRILLLMVGIGSLIKEKRSAAAKKKGASLLCLALASTGLFNPMILAAGLIACDPNRKRGWPATEVMTAVGLMFAIVGGLAELDIDSMAIPMTIAGLMFAAFVISGKSTDMWIERTADISWESDAEITGSSERVDVRLDDDGNFQLMNDPGAPWKIWMLRMVCLAISAYTPWAILPSVVGFWITLQYTKRGGVLWDTPSPKEYKKGDTTTGVYRIMTRGLLGSYQAGAGVMVEGVFHTLWHTTKGAALMSGEGRLDPYWGSVKEDRLCYGGPWKLQHKWNGQDEVQMIVVEPGKNVKNVQTKPGVFKTPEGEIGAVTLDFPTGTSGSPIVDKNGDVIGLYGNGVIMPNGSYISAIVQGERMDEPIPAGFEPEMLRKKQITVLDLHPGAGKTRRILPQIIKEAINRRLRTAVLAPTRVVAAEMAEALRGLPIRYQTSAVPREHNGNEIVDVMCHATLTHRLMSPHRVPNYNLFVMDEAHFTDPASIAARGYISTKVELGEAAAIFMTATPPGTSDPFPESNSPISDLQTEIPDRAWNSGYEWITEYTGKTVWFVPSVKMGNEIALCLQRAGKKVVQLNRKSYETEYPKCKNDDWDFVITTDISEMGANFKASRVIDSRKSVKPTIITEGEGRVILGEPSAVTAASAAQRRGRIGRNPSQVGDEYCYGGHTNEDDSNFAHWTEARIMLDNINMPNGLIAQFYQPEREKVYTMDGEYRLRGEERKNFLELLRTADLPVWLAYKVAAAGVSYHDRRWCFDGPRTNTILEDNNEVEVITKLGERKILRPRWIDARVYSDHQALKAFKDFASGKRSQIGLIEVLGKMPEHFMGKTWEALDTMYVVATAEKGGRAHRMALEELPDALQTIALIALLSVMTMGVFFLLMQRKGIGKIGLGGAVLGVATFFCWMAEVPGTKIAGMLLLSLLLMIVLIPEPEKQRSQTDNQLAVFLICVMTLVSAVAANEMGWLDKTKSDISSLFGQRIEVKENFSMGEFLLDLRPATAWSLYAVTTAVLTPLLKHLITSDYINTSLTSINVQASALFTLARGFPFVDVGVSALLLAAGCWGQVTLTVTVTAATLLFCHYAYMVPGWQAEAMRSAQRRTAAGIMKNAVVDGIVATDVPELERTTPIMQKKVGQIMLILVSLAAVVVNPSVKTVREAGILITAAAVTLWENGASSVWNATTAIGLCHIMRGGWLSCLSITWTLIKNMEKPGLKRGGAKGRTLGEVWKERLNQMTKEEFTRYRKEAIIEVDRSAAKHARKEGNVTGGHPVSRGTAKLRWLVERRFLEPVGKVIDLGCGRGGWCYYMATQKRVQEVRGYTKGGPGHEEPQLVQSYGWNIVTMKSGVDVFYRPSECCDTLLCDIGESSSSAEVEEHRTIRVLEMVEDWLHRGPREFCVKVLCPYMPKVIEKMELLQRRYGGGLVRNPLSRNSTHEMYWVSRASGNVVHSVNMTSQVLLGRMEKRTWKGPQYEEDVNLGSGTRAVGKPLLNSDTSKIKNRIERLRREYSSTWHHDENHPYRTWNYHGSYDVKPTGSASSLVNGVVRLLSKPWDTITNVTTMAMTDTTPFGQQRVFKEKVDTKAPEPPEGVKYVLNETTNWLWAFLAREKRPRMCSREEFIRKVNSNAALGAMFEEQNQWRSAREAVEDPKFWEMVDEEREAHLRGECHTCIYNMMGKREKKPGEFGKAKGSRAIWFMWLGARFLEFEALGFLNEDHWLGRKNSGGGVEGLGLQKLGYILREVGTRPGGKIYADDTAGWDTRITRADLENEAKVLELLDGEHRRLARAIIELTYRHKVVKVMRPAADGRTVMDVISREDQRGSGQVVTYALNTFTNLAVQLVRMMEGEGVIGPDDVEKLTKGKGPKVRTWLFENGEERLSRMAVSGDDCVVKPLDDRFATSLHFLNAMSKVRKDIQEWKPSTGWYDWQQVPFCSNHFTELIMKDGRTLVVPCRGQDELVGRARISPGAGWNVRDTACLAKSYAQMWLLLYFHRRDLRLMANAICSAVPVNWVPTGRTTWSIHAGGEWMTTEDMLEVWNRVWIEENEWMEDKTPVEKWSDVPYSGKREDIWCGSLIGTRARATWAENIQVAINQVRAIIGDEKYVDYMSSLKRYEDTTLVEDTVL

>ANW69646.1 genome polyprotein [West Nile virus]

MSKKPGGPGKSRAVNMLKRGMPRVLSLIGLKRAMLSLIDGKGPIRFVLALLAFFRFTAIAPTRAVLDRWRGVNKQTAMKHLLSFKKELGTLTSAINRRSSKQKKRGGKTGIAVMIGLIASVGAVTLSNFQGKVMMTVNATDVTDVITIPTAAGKNLCIVRAMDVGYMCDDTITYECPVLSAGNDPEDIDCWCTKSAVYVRYGRCTKTRHSRRSRRSLTVQTHGESTLANKKGAWMDSTKATRYLVKTESWILRNPGYALVAAVIGWMLGSNTMQRVVFVVLLLLVAPAYSFNCLGMSNRDFLEGVSGATWVDLVLEGDSCVTIMSKDKPTIDVKMMNMDAANLAEVRSYCYLATVSDLSTKAACPTMGEAHNDKRADPAFVCRQGVVDRGWGNGCGLFGKGSIDTCAKFACSTKAIGRTILKENIKYEVAIFVHGPTTVESHGNYSTQAGATQAGRFSITPAAPSYTLKLGEYGEVTVDCEPRSGIDTNAYYVMTVGTKTFLVHREWFMDLNLPWSSAGSTVWRNRETLMEFEEPHATKQSVIALGSQEGALHQALAGAIPVEFSSNTVKLTSGHLKCRVKMEKLQLKGTTYGVCSKAFKFLGTPADTGHGTVVLELQYTGTDGPCKVPISSVASLNDLTPVGRLVTVNPFVSVATANAKVLIELEPPFGDSYIVVGRGEQQINHHWHKSGSSIGKAFTTTLKGAQRLAALGDTAWDFGSVGGVFTSVGKAVHQVFGGAFRSLFGGMSWITQGLLGALLLWMGINARDRSIALTFLAVGGVLLFLSVNVHADTGCAIDISRQELRCGSGVFIHNDVEAWMDRYKYYPETPQGLAKIIQKAHKEGVCGLRSVSRLEHQMWEAVKDELNTLLKENGVDLSVVVEKQEGMYKSAPKRLTATTEKLEIGWKAWGKSILFAPELANNTFVVDGPETKECPTQNRAWNSLEVEDFGFGLTSTRMFLKVRESNTTECDSKIIGTAVKNNLAIHSDLSYWIESRLNDTWKLERAVLGEVKSCTWPETHTLWGDGILESDLIIPVTLAGPRSNHNRRPGYKTQNQGPWDEGRVEIDFDYCPGTTVTLSESCGHRGPATRTTTESGKLITDWCCRSCTLPPLRYQTDSGCWYGMEIRPQRHDEKTLVQSQVNAYNADMIDPFQLGLLVVFLATQEVLRKRWTAKISMPAILIALLVLVFGGITYTDVLRYVILVGAAFAESNSGGDVVHLALMATFKIQPVFMVASFLKARWTNQENILLMLAAVFFQMAYHDARQILLWEIPDVLNSLAVAWMILRAITFTTTSNVVVPLLALLTPGLRCLNLDVYRILLLMVGIGSLIREKRSAAAKKKGASLLCLALASTGLFNPMILAAGLIACDPNRKRGWPATEVMTAVGLMFAIVGGLAELDIDSMAIPMTIAGLMFAAFVISGKSTDMWIERTADISWESDAEITGSSERVDVRLDDDGNFQLMNDPGAPWKIWMLRMVCLAISAYTPWAILPSVVGFWITLQYTKRGGVLWDTPSPKEYKKGDTTTGVYRIMTRGLLGSYQAGAGVMVEGVFHTLWHTTKGAALMSGEGRLDPYWGSVKEDRLCYGGPWKLQHKWNGQDEVQMIVVEPGKNVKNVQTKPGVFKTPEGEIGAVTLDFPTGTSGSPIVDKNGDVIGLYGNGVIMPNGSYISAIVQGERMDEPIPAGFEPEMLRKKQITVLDLHPGAGKTRRILPQIIKEAINRRLRTAVLAPTRVVAAEMAEALRGLPIRYQTSAVPREHNGNEIVDVMCHATLTHRLMSPHRVPNYNLFVMDEAHFTDPASIAARGYISTKVELGEAAAIFMTATPPGTSDPFPESNSPISDLQTEIPDRAWNSGYEWITEYTGKTVWFVPSVKMGNEIALCLQRAGKKVVQLNRKSYETEYPKCKNDDWDFVITTDISEMGANFKASRVIDSRKSVKPTIITEGEGRVILGEPSAVTAASAAQRRGRIGRNPSQVGDEYCYGGHTNEDDSNFAHWTEARIMLDNINMPNGLIAQFYQPEREKVYTMDGEYRLRGEERKNFLELLRTADLPVWLAYKVAAAGVSYHDRRWCFDGPRTNTILEDNNEVEVITKLGERKILRPRWIDARVYSDHQALKAFKDFASGKRSQIGLIEVLGKMPEHFMGKTWEALDTMYVVATAEKGGRAHRMALEELPDALQTIALIALLSVMTMGVFFLLMQRKGIGKIGLGGAVLGVATFFCWMAEVPGTKIAGMLLLSLLLMIVLIPEPEKQRSQTDNQLAVFLICVMTLVSAVAANEMGWLDKTKSDISSLFGQRIEVKENFSMGEFLLDLRPATAWSLYAVTTAVLTPLLKHLITSDYINTSLTSINVQASALFTLARGFPFVDVGVSALLLAAGCWGQVTLTVTVTAATLLFCHYAYMVPGWQAEAMRSAQRRTAAGIMKNAVVDGIVATDVPELERTTPIMQKKVGQIMLILVSLAAVVVNPSVKTVREAGILITAAAVTLWENGASSVWNATTAIGLCHIMRGGWLSCLSITWTLIKNMEKPGLKRGGAKGRTLGEVWKERLNQMTKEEFTRYRKEAIIEVDRSAAKHARKEGNVTGGHPVSRGTAKLRWLVERRFLEPVGKVIDLGCGRGGWCYYMATQKRVQEVRGYTKGGPGHEEPQLVQSYGWNIVTMKSGVDVFYRPSECCDTLLCDIGESSSSAEVEEHRTIRVLEMVEDWLHRGPREFCVKVLCPYMPKVIEKMELLQRRYGGGLVRNPLSRNSTHEMYWVSRASGNVVHSVNMTSQVLLGRMEKRTWKGPQYEEDVNLGSGTRAVGKPLLNSDTSKIKNRIERLRREYSSTWHHDENHPYRTWNYHGSYDVKPTGSASSLVNGVVRLLSKPWDTITNVTTMAMTDTTPFGQQRVFKEKVDTKAPEPPEGVKYVLNETTNWLWAFLAREKRPRMCSREEFIRKVNSNAALGAMFEEQNQWRSAREAVEDPKFWEMVDEEREAHLRGECHTCIYNMMGKREKKPGEFGKAKGSRAIWFMWLGARFLEFEALGFLNEDHWLGRKNSGGGVEGLGLQKLGYILREVGTRPGGKIYADDTAGWDTRITRADLENEAKVLELLDGEHRRLARAIIELTYRHKVVKVMRPAADGRTVMDVISREDQRGSGQVVTYALNTFTNLAVQLVRMMEGEGVIGPDDVEKLTKGKGPKVRTWLFENGEERLSRMAVSGDDCVVKPLDDRFATSLHFLNAMSKVRKDIQEWKPSTGWYDWQQVPFCSNHFTELIMKDGRTLVVPCRGQDELVGRARISPGAGWNVRDTACLAKSYAQMWLLLYFHRRDLRLMANAICSAVPVNWVPTGRTTWSIHAGGEWMTTEDMLEVWNRVWIEENEWMEDKTPVEKWSDVPYSGKREDIWCGSLIGTRARATWAENIQVAINQVRAIIGDEKYVDYMSSLKRYEDTTLVEDTVL

>ABA54584.1 polyprotein precursor [West Nile virus]

MSKKPGGPGKSRAVNMLKRGMPRVLSLIGLKRAMLSLIDGKGPIRFVLALLAFFRFTAIAPTRAVLDRWRGVNKQTAMKHLLSFKKELGTLTSAINRRSSKQKKRGGKTGIAVMIGLIASVGAVTLSNFQGKVMMTVNATDVTDVITIPTAAGKNLCIVRAMDVGYMCDDTITYECPVLSAGNDPEDIDCWCTKSAVYVRYGRCTKTRHSRRSRRSLTVQTHGESTLANKKGAWMDSTKATRYLVKTESWILRNPGYALVAAVIGWMLGSNTMQRVVFVVLLLLVAPAYSFNCLGMSNRDFLEGVSGATWVDLVLEGDSCVTIMSKDKPTIDVKMMNMEAANLAEVRSYCYLATVSDLSTKAACPTMGEAHNDKRADPAFVCRQGVVDRGWGNGCGLFGKGSIDTCAKFACSTKAIGRTILKENIKYEVAIFVHGPTTVESHGNYSTQAGATQAGRFSITPAAPSYTLKLGEYGEVTVDCEPRSGIDTNAYYVMTVGTKTFLVHREWFMDLNLPWSSAGSTVWRNRETLMEFEEPHATKQSVIALGSQEGALHQALAGAIPVEFSSNTVKLTSGHLKCRVKMEKLQLKGTTYGVCSKAFKFLGTPADTGHGTVVLELQYTGTDGPCKVPISSVASLNDLTPVGRLVTVNPFVSVATANAKVLIELEPPFGDSYIVVGRGEQQINHHWHKSGSSIGKAFTTTLKGAQRLAALGDTAWDFGSVGGVFTSVGKAVHQVFGGAFRSLFGGMSWITQGLLGALLLWMGINARDRSIALTFLAVGGVLLFLSVNVHADTGCAIDISRQELRCGSGVFIHNDVEAWMDRYKYYPETPQGLAKIIQKAHKEGVCGLRSVSRLEHQMWEAVKDELNTLLKENGVDLSVVVEKQEGMYKSAPKRLTATTEKLEIGWKAWGKSILFAPELANNTFVVDGPETKECPTQNRAWNSLEVEDFGFGLTSTRMFLKVRESNTTECDSKIIGTAVKNNLAIHSDLSYWIESRLNDTWKLERAVLGEVKSCTWPETHTLWGDGILESDLIIPVTLAGPRSNHNRRPGYKTQNQGPWDEGRVEIDFDYCPGTTVTLSESCGHRGPATRTTTESGKLITDWCCRSCTLPPLRYQTDSGCWYGMEIRPQRHDEKTLVQSQVNAYNADMIDPFQLGLLVVFLATQEVLRKRWTAKISMPAILIALLVLVFGGITYTDVLRYVILVGAAFAESNSGGDVVHLALMATFKIQPVFMVASFLKARWTNQENILLMLAAVFFQMAYHDARQILLWEIPDVLNSLAVAWMILRAITFTTTSNVVVPLLALLTPGLRCLNLDVYRILLLMVGIGSLIREKRSAAAKKKGASLLCLALASTGLFNPMILAAGLIACDPNRKRGWPATEVMTAVGLMFAIVGGLAELDIDSMAIPMTIAGLMFAAFVISGKSTDMWIERTADISWESDAEITGSSERVDVRLDDDGNFQLMNDPGAPWKIWMLRMVCLAISAYTPWAILPSVVGFWITLQYTKRGGVLWDTPSPKEYKKGDTTTGVYRIMTRGLLGSYQAGAGVMVEGVFHTLWHTTKGAALMSGEGRLDPYWGSVKEDRLCYGGPWKLQHKWNGQDEVQMIVVEPGKNVKNVQTKPGVFKTPEGEIGAVTLDFPTGTSGSPIVDKNGDVIGLYGNGVIMPNGSYISAIVQGERMDEPIPAGFEPEMLRKKQITVLDLHPGAGKTRRILPQIIKEAINRRLRTAVLAPTRVVAAEMAEALRGLPIRYQTSAVPREHNGNEIVDVMCHATLTHRLMSPHRVPNYNLFVMDEAHFTDPASIAARGYISTKVELGEAAAIFMTATPPGTSDPFPESNSPISDLQTEIPDRAWNSGYEWITEYTGKTVWFVPSVKMGNEIALCLQRAGKKVVQLNRKSYETEYPKCKNDDWDFVITTDISEMGANFKASRVIDSRKSVKPTIITEGEGRVILGEPSAVTAASAAQRRGRIGRNPSQVGDEYCYGGHTNEDDSNFAHWTEARIMLDNINMPNGLIAQFYQPEREKVYTMDGEYRLRGEERKNFLELLRTADLPVWLAYKVAAAGVSYHDRRWCFDGPRTNTILEDNNEVEVITKLGERKILRPRWIDARVYSDHQALKAFKDFASGKRSQMGLIEVLGKMPEHFMGKTWEALDTMYVVATAEKGGRAHRMALEELPDALQTIALIALLSVMTMGVFFLLMQRKGIGKIGLGGAVLGVATFFCWMAEVPGTKIAGMLLLSLLLMIVLIPEPEKQRSQTDNQLAVFLICVMTLVSAVAANEMGWLDKTKSDISSLFGQRIEVKENFSMGEFLLDLRPATAWSLYAVTTAVLTPLLKHLITSDYINTSLTSINVQASALFTLARGFPFVDVGVSALLLAAGCWGQVTLTVTVTAATLLFCHYAYMVPGWQAEAMRSAQRRTAAGIMKNAVVDGIVATDVPELERTTPIMQKKVGQIMLILVSLAAVVVNPSVKTVREAGILITAAAVTLWENGASSVWNATTAIGLCHIMRGGWLSCLSITWTLIKNMEKPGLKRGGAKGRTLGEVWKERLNQMTKEEFTRYRKEAIIEVDRSAAKHARKEGNVTGGHPVSRGTAKLRWLVERRFLEPVGKVIDLGCGRGGWCYYMATQKRVQEVRGYTKGGPGHEEPQLVQSYGWNIVTMKSGVDVFYRPSECCDTLLCDIGESSSSAEVEEHRTIRVLEMVEDWLHRGPREFCVKVLCPYMPKVIEKMELLQRRYGGGLVRNPLSRNSTHEMYWVSRASGNVVHSVNMTSQVLLGRMEKRTWKGPQYEEDVNLGSGTRAVGKPLLNSDTSKIKNRIERLRREYSSTWHHDENHPYRTWNYHGSYDVKPTGSASSLVNGVVRLLSKPWDTITNVTTMAMTDTTPFGQQRVFKEKVDTKAPEPPEGVKYVLNETTNWLWAFLAREKRPRMCSREEFIRKVNSNAALGAMFEEQNQWRSAREAVEDPKFWEMVDEEREAHLRGECHTCIYNMMGKREKKPGEFGKAKGSRAIWFMWLGARFLEFEALGFLNEDHWLGRKNSGGGVEGLGLQKLGYILREVGTRPGGKIYADDTAGWDTRITRADLENEAKVLELLDGEHRRLARAIIELTYRHKVVKVMRPAADGRTVMDVISREDQRGSGQVVTYALNTFTNLAVQLVRMMEGEGVIGPDDVEKLTKGKGPKVRTWLFENGEERLSRMAVSGDDCVVKPLDDRFATSLHFLNAMSKVRKDIQEWKPSTGWYDWQQVPFCSNHFTELIMKDGRTLVVPCRGQDELVGRARISPGAGWNVRDTACLAKSYAQMWLLLYFHRRDLRLMANAICSAVPVNWVPTGRTTWSIHAGGEWMTTEDMLEVWNRVWIEENEWMEDKTPVEKWSDVPYSGKREDIWCGSLIGTRARATWAENIQVAINQVRAIIGDEKYVDYMSSLKRYEDTTLVEDTVL

>AEZ56182.1 polyprotein [West Nile virus]

MSKKPGGPGKSRAVNMLKRGMPRVLSLIGLKRAMLSLIDGKGPIRFVLALLAFFRFTAIAPTRAVLDRWRGVNKQTAMKHLLSFKKELGTLTSAINRRSSKQKKRGGKTGIAVMIGLIASVGAVTLSNFQGKVMMTVNATDVTDVITIPTAAGKNLCIVRAMDVGYMCDDTITYECPVLSAGNDPEDIDCWCTKSAVYVRYGRCTKTRHSRRSRRSLTVQTHGESTLANKKGAWMDSTKATRYLVKTESWILRNPGYALVAAVIGWMLGSNTMQRVVFVVLLLLVAPAYSFNCLGMSNRDFLEGVSGATWVDLVLEGDSCVTIMSKDKPTIDVKMMNMEAANLAEVRSYCYLATVSDLSTKAACPTMGEAHNDKRADPAFVCRQGVVDRGWGNGCGLFGKGSIDTCAKFACSTKAIGRTILKENIKYEVAIFVHGPTTVESHGNYSTQAGATQAGRFSITPAAPSYTLKLGEYGEVTVDCEPRSGIDTNAYYVMTVGTKTFLVHREWFMDLNLPWSSAGSTVWRNRETLMEFEEPHATKQSVIALGSQEGALHQALAGAIPVEFSSNTVKLTSGHLKCRVKMEKLQLKGTTYGVCSKAFKFLGTPADTGHGTVVLELQYTGTDGPCKVPISSVASLNDLTPVGRLVTVNPFVSVATANAKVLIELEPPFGDSYIVVGRGEQQINHHWHKSGSSIGKAFTTTLKGAQRLAALGDTAWDFGSVGGVFTSVGKAVHQVFGGAFRSLFGGMSWITQGLLGALLLWMGINARDRSIALTFLAVGGVLLFLSVNVHADTGCAIDISRQELRCGSGVFIHNDVEAWMDRYKYYPETPQGLAKIIQKAHKEGVCGLRSVSRLEHQMWEAVKDELNTLLKENGVDLSVVVEKQEGMYKSAPKRLTATTEKLEIGWKAWGKSILFAPELANNTFVVDGPETKECPTQNRAWNSLEVEDFGFGLTSTRMFLKVRESNTTECDSKIIGTAVKNNLAIHSDLSYWIESRLNDTWKLERAVLGEVKSCTWPETHTLWGDGILESDLIIPVTLAGPRSNHNRRPGYKTQNQGPWDEGRVEIDFDYCPGTTVTLSESCGHRGPATRTTTESGKLITDWCCRSCTLPPLRYQTDSGCWYGMEIRPQRHDEKTLVQSQVNAYNADMIDPFQLGLLVVFLATQEVLRKRWTAKISMPAILIALLVLVFGGITYTDVLRYVILVGAAFAESNSGGDVVHLALMATFKIQPVFMVASFLKARWTNQENILLMLAAVFFQMAYHDARQILLWEIPDVLNSLAVAWMILRAITFTTTSNVVVPLLALLTPGLRCLNLDVYRILLLMVGIGSLIREKRSAAAKKKGASLLCLALASTGLFNPMILAAGLIACDPNRKRGWPATEVMTAVGLMFAIVGGLAELDIDSMAIPMTIAGLMFAAFVISGKSTDMWIERTADISWESDAEITGSSERVDVRLDDDGNFQLMNDPGAPWKIWMLRMVCLAISAYTPWAILPSVVGFWITLQYTKRGGVLWDTPSPKEYKKGDTTTGVYRIMTRGLLGSYQAGAGVMVEGVFHTLWHTTKGAALMSGEGRLDPYWGSVKEDRLCYGGPWKLQHKWNGQDEVQMIVVEPGKNVKNVQTKPGVFKTPEGEIGAVTLDFPTGTSGSPIVDKNGDVIGLYGNGVIMPNGSYISAIVQGERMDEPIPAGFEPEMLRKKQITVLDLHPGAGKTRRILPQIIKEAINRRLRTAVLAPTRVVAAEMAEALRGLPIRYQTSAVPREHNGNEIVDVMCHATLTHRLMSPHRVPNYNLFVMDEAHFTDPASIAARGYISTKVELGEAAAIFMTATPPGTSDPFPESNSPISDLQTEIPDRAWNSGYEWITEYTGKTVWFVPSVKMGNEIALCLQRAGKKVVQLNRKSYETEYPKCKNDDWDFVITTDISEMGANFKASRVIDSRKSVKPTIITEGEGRVILGEPSAVTAASAAQRRGRIGRNPSQVGDEYCYGGHTNEDDSNFAHWTEARIMLDNINMPNGLIAQFYQPEREKVYTMDGEYRLRGEERKNFLELLRTADLPVWLAYKVAAAGVSYHDRRWCFDGPRTNTILEDNNEVEVITKLGERKILRPRWIDARVYSDHQALKAFKDFASGKRSQIGLIEVLGKMPEHFMGKTWEALDTMYVVATAEKGGRAHRMALEELPDALQTIALIALLSVMTMGVFFLLMQRKGIGKIGLGGAVLGVATFFCWMAEVPGTKIAGMLLLSLLLMIVLIPEPEKQRSQTDNQLAVFLICVMTLVSAVAANEMGWLDKTKSDISSLFGQRIEVKENFSMGEFLLDLRPATAWSLYAVTTAVLTPLLKHLITSDYINTSLTSINVQASALFTLARGFPFVDVGVSALLLAAGCWGQVTLTVTVTAATLLFCHYAYMVPGWQAEAMRSAQRRTAAGIMKNAVVDGIVATDVPELERTTPIMQKKVGQIMLILVSLAAVVVNPSVKTVREAGILITAAAVTLWDNGASSVWNATTAIGLCHIMRGGWLSCLSITWTLIKNMEKPGLKRGGAKGRTLGEVWKERLNQMTKEEFTRYRKEAIIEVDRSAAKHARKEGNVTGGHPVSRGTAKLRWLVERRFLEPVGKVIDLGCGRGGWCYYMATQKRVQEVRGYTKGGPGHEEPQLVQSYGWNIVTMKSGVDVFYRPSECCDTLLCDIGESSSSAEVEEHRTIRVLEMVEDWLHRGPREFCVKVLCPYMPKVIEKMELLQRRYGGGLVRNPLSRNSTHEMYWVSRASGNVVHSVNMTSQVLLGRMEKRTWKGPQYEEDVNLGSGTRAVGKPLLNSDTSKIKNRIERLRREYSSTWHHDENHPYRTWNYHGSYDVKPTGSASSLVNGVVRLLSKPWDTITNVTTMAMTDTTPFGQQRVFKEKVDTKAPEPPEGVKYVLNETTNWLWAFLAREKRPRMCSREEFIRKVNSNAALGAMFEEQNQWRSAREAVEDPKFWEMVDEEREAHLRGECHTCIYNMMGKREKKPGEFGKAKGSRAIWFMWLGARFLEFEALGFLNEDHWLGRKNSGGGVEGLGLQKLGYILREVGTRPGGKIYADDTAGWDTRITRADLENEAKVLELLDGEHRRLARAIIELTYRHKVVKVMRPAADGRTVMDVISREDQRGSGQVVTYALNTFTNLAVQLVRMMEGEGVIGPDDVEKLTKGKGPKVRTWLFENGEERLSRMAVSGDDCVVKPLDDRFATSLHFLNAMSKVRKDIQEWKPSTGWYDWQQVPFCSNHFTELIMKDGRTLVVPCRGQDELVGRARISPGAGWNVRDTACLAKSYAQMWLLLYFHRRDLRLMANAICSAVPVNWVPTGRTTWSIHAGGEWMTTEDMLEVWNRVWIEENEWMEDKTPVEKWSDVPYSGKREDIWCGSLIGTRARATWAENIQVAINQVRAIIGDEKYVDYMSSLKRYEDTTLVEDTVL

>AHW48576.1 polyprotein [West Nile virus]

MSKKPGGPGKSRAVNMLKRGMPRVLSLIGLKRAMLSLIDGKGPIRFVLALLAFFRFTAIAPTRAVLDRWRGVNKQTAMKHLLSFKKELGTLTSAINRRSSKQKKRGGKTGIAVMIGLIASVGAVTLSNFQGKVMMTVNATDVTDVITIPTAAGKNLCIVRAMDVGYMCDDTITYECPVLSAGNDPEDIDCWCTKSAVYVRYGRCTKTRHSRRSRRSLTVQTHGESTLSNKKGAWMDSTKATRYLVKTESWILRNPGYALVAAVIGWMLGSNTMQRVVFVVLLLLVAPAYSFNCLGMSNRDFLEGVSGATWVDLVLEGDSCVTIMSKDKPTIDVKMMNMEAANLAEVRSYCYLATVSDLSTKAACPTMGEAHNDKRADPAFVCRQGVVDRGWGNGCGLFGKGSIDTCAKFACSTKAIGRTILKENIKYEVAIFVHGPTTVESHGNYSTQAGATQAGRFSITPAAPSYTLKLGEYGEVTVDCEPRSGIDTNAYYVMTVGTKTFLVHREWFMDLNLPWSSAGSTVWRNRETLMEFEEPHATKQSVIALGSQEGALHQALAGAIPVEFSSNTVKLTSGHLKCRVKMEKLQLKGTTYGVCSKAFKFLGTPADTGHGTVVLELQYTGTDGPCKVPISSVASLNDLTPVGRLVTVNPFVSVATANAKVLIELEPPFGDSYIVVGRGEQQINHHWHKSGSSIGKAFTTTLKGAQRLAALGDTAWDFGSVGGVFTSVGKAVHQVFGGAFRSLFGGMSWITQGLLGALLLWMGINARDRSIALTFLAVGGVLLFLSVNVHADTGCAIDISRQELRCGSGVFIHNDVEAWMDRYKYYPETPQGLAKIIQKAHKEGVCGLRSVSRLEHQMWEAVKDELNTLLKENGVDLSVVVEKQEGMYKSAPKRLTATTEKLEIGWKAWGKSILFAPELANNTFVVDGPETKECPTQNRAWNSLEVEDFGFGLTSTRMFLKVRESNTTECDSKIIGTAVKNNLAIHSDLSYWIESRLNDTWKLERAVLGEVKSCTWPETHTLWGDGILESDLIIPVTLAGPRSNHNRRPGYKTQNQGPWDEGRVEIDFDYCPGTTVTLSESCGHRGPATRTTTESGKLITDWCCRSCTLPPLRYQTDSGCWYGMEIRPQRHDEKTLVQSQVNAYNADMIDPFQLGLLVVFLATQEVLRKRWTAKISMPAILIALLVLVFGGITYTDVLRYVILVGAAFAESNSGGDVVHLALMATFKIQPVFMVASFLKARWTNQENILLMLAAVFFQMAYHDARQILLWEIPDVLNSLAVAWMILRAITFTTTSNVVVPLLALLTPGLRCLNLDVYRILLLMVGIGSLIREKRSAAAKKKGASLLCLALASTGLFNPMILAAGLIACDPNRKRGWPATEVMTAVGLMFAIVGGLAELDIDSMAIPMTIAGLMFAAFVISGKSTDMWIERTADISWESDAEITGSSERVDVRLDDDGNFQLMNDPGAPWKIWMLRMVCLAISAYTPWAILPSVVGFWITLQYTKRGGVLWDTPSPKEYKKGDTTTGVYRIMTRGLLGSYQAGAGVMVEGVFHTLWHTTKGAALMSGEGRLDPYWGSVKEDRLCYGGPWKLQHKWNGQDEVQMIVVEPGKNVKNVQTKPGVFKTPEGEIGAVTLDFPTGTSGSPIVDKNGDVIGLYGNGVIMPNGSYISAIVQGERMDEPIPAGFEPEMLRKKQITVLDLHPGAGKTRRILPQIIKEAINRRLRTAVLAPTRVVAAEMAEALRGLPIRYQTSAVPREHNGNEIVDVMCHATLTHRLMSPHRVPNYNLFVMDEAHFTDPASIAARGYISTKVELGEAAAIFMTATPPGTSDPFPESNSPISDLQTEIPDRAWNSGYEWITEYTGKTVWFVPSVKMGNEIALCLQRAGKKVVQLNRKSYETEYPKCKNDDWDFVITTDISEMGANFKASRVIDSRKSVKPTIITEGEGRVILGEPSAVTAASAAQRRGRIGRNPSQVGDEYCYGGHTNEDDSNFAHWTEARIMLDNINMPNGLIAQFYQPEREKVYTMDGEYRLRGEERKNFLELLRTADLPVWLAYKVAAAGVSYHDRRWCFDGPRTNTILEDNNEVEVITKLGERKILRPRWIDARVYSDHQALKAFKDFASGKRSQIGLIEVLGKMPEHFMGKTWEALDTMYVVATAEKGGRAHRMALEELPDALQTIALIALLSVMTMGVFFLLMQRKGIGKIGLGGAVLGVATFFCWMAEVPGTKIAGMLLLSLLLMIVLIPEPEKQRSQTDNQLAVFLICVMTLVSAVAANEMGWLDKTKSDISSLFGQRIEVKENFSMGEFLLDLRPATAWSLYAVTTAVLTPLLKHLITSDYINTSLTSINVQASALFTLARGFPFVDVGVSALLLAAGCWGQVTLTVTVTAATLLFCHYAYMVPGWQAEAMRSAQRRTAAGIMKNAVVDGIVATDVPELERTTPIMQKKVGQIMLILVSLAAVVVNPSVKTVREAGILITAAAVTLWENGASSVWNATTAIGLCHIMRGGWLSCLSITWTLIKNMEKPGLKRGGAKGRTLGEVWKERLNQMTKEEFTRYRKEAIIEVDRSAAKHARKEGNVTGGHPVSRGTAKLRWLVERRFLEPVGKVIDLGCGRGGWCYYMATQKRVQEVRGYTKGGPGHEEPQLVQSYGWNIVTMKSGVDVFYRPSECCDTLLCDIGESSSSAEVEEHRTIRVLEMVEDWLHRGPREFCVKVLCPYMPKVIEKMELLQRRYGGGLVRNPLSRNSTHEMYWVSRASGNVVHSVNMTSQVLLGRMEKRTWKGPQYEEDVNLGSGTRAVGKPLLNSDTSKIKNRIERLRREYSSTWHHDENHPYRTWNYHGSYDVKPTGSASSLVNGVVRLLSKPWDTITNVTTMAMTDTTPFGQQRVFKEKVDTKAPEPPEGVKYVLNETTNWLWAFLAREKRPRMCSREEFIRKVNSNAALGAMFEEQNQWRSAREAVEDPKFWEMVDEEREAHLRGECHTCIYNMMGKREKKPGEFGKAKGSRAIWFMWLGARFLEFEALGFLNEDHWLGRKNSGGGVEGLGLQKLGYILREVGTRPGGKIYADDTAGWDTRITRADLENEAKVLELLDGEHRRLARAIIELTYRHKVVKVMRPAADGRTVMDVISREDQRGSGQVVTYALNTFTNLAVQLVRMMEGEGVIGPDDVEKLTKGKGPKVRTWLFENGEERLSRMAVSGDDCVVKPLDDRFATSLHFLNAMSKVRKDIQEWKPSTGWYDWQQVPFCSNHFTELIMKDGRTLVVPCRGQDELVGRARISPGAGWNVRDTACLAKSYAQMWLLLYFHRRDLRLMANAICSAVPVNWVPTGRTTWSIHAGGEWMTTEDMLEVWNRVWIEENEWMEDKTPVEKWSDVPYSGKREDIWCGSLIGTRARATWAENIQVAINQVRAIIGDEKYVDYMSSLKRYEDTTLVEDTVL

>ANW68749.1 genome polyprotein [West Nile virus]

MSKKPGGPGKSRAVNMLKRGMPRVLSLIGLKRAMLSLIDGKGPIRFVLALLAFFRFTAIAPTRAVLDRWRGVNKQTAMKHLLSFKKELGTLTSAINRRSSKQKKRGGKTGIAVMIGLIASVGAVTLSNFQGKVMMTVNATDVTDVITIPTAAGKNLCIVRAMDVGYMCDDTITYECPVLSAGNDPEDIDCWCTKSAVYVRYGRCTKTRHSRRSRRSLTVQTHGESTLANKKGAWMDSTKATRYLVKTESWILRNPGYALVAAVIGWMLGSNTMQRVVFVVLLLLVAPAYSFNCLGMSNRDFLEGVSGATWVDLVLEGDSCVTIMSKDKPTIDVKMMNMEAANLAEVRSYCYLATVSDLSTKAACPTMGEAHNDKRADPAFVCRQGVVDRGWGNGCGLFGKGSIDTCAKFACSTKAIGRTILKENIKYEVAIFVHGPTTVESHGNYSTQVGATQAGRFSITPAAPSYTLKLGEYGEVTVDCEPRSGIDTNAYYVMTVGTKTFLVHREWFMDLNLPWSSAGSTVWRNRETLMEFEEPHATKQSVIALGSQEGALHQALAGAIPVEFSSNTVKLTSGHLKCRVKMEKLQLKGTTYGVCSKAFKFLGTPADTGHGTVVLELQYTGTDGPCKVPISSVASLNDLTPVGRLVTVNPFVSVATANAKVLIELEPPFGDSYIVVGRGEQQINHHWHKSGSSIGKAFTTTLKGAQRLAALGDTAWDFGSVGGVFTSVGKAVHQVFGGAFRSLFGGMSWITQGLLGALLLWMGINARDRSIALTFLAVGGVLLFLSVNVHADTGCAIDISRQELRCGSGVFIHNDVEAWMDRYKYYPETPQGLAKIIQKAHKEGVCGLRSVSRLEHQMWEAVKDELNTLLKENGVDLSVVVEKQEGMYKSAPKRLTATTEKLEIGWKAWGKSILFAPELANNTFVVDGPETKECPTQNRAWNSLEVEDFGFGLTSTRMFLKVRESNTTECDSKIIGTAVKNNLAIHSDLSYWIESRLNDTWKLERAVLGEVKSCTWPETHTLWGDGILESDLIIPVTLAGPRSNHNRRPGYKTQNQGPWDEGRVEIDFDYCPGTTVTLSESCGHRGPATRTTTESGKLITDWCCRSCTLPPLRYQTDSGCWYGMEIRPQRHDEKTLVQSQVNAYNADMIDPFQLGLLVVFLATQEVLRKRWTAKISMPAILIALLVLVFGGITYTDVLRYVILVGAAFAESNSGGDVVHLALMATFKIQPVFMVASFLKARWTNQENILLMLAAVFFQMAYHDARQILLWEIPDVLNSLAVAWMILRAITFTTTSNVVVPLLALLTPGLRCLNLDVYRILLLMVGIGSLIREKRSAAAKKKGASLLCLALASTGLFNPMILAAGLIACDPNRKRGWPATEVMTAVGLMFAIVGGLAELDIDSMAIPMTIAGLMFAAFVISGKSTDMWIERTADISWESDAEITGSSERVDVRLDDDGNFQLMNDPGAPWKIWMLRMVCLAISAYTPWAILPSIVGFWITLQYTKRGGVLWDTPSPKEYKKGDTTTGVYRIMTRGLLGSYQAGAGVMVEGVFHTLWHTTKGAALMSGEGRLDPYWGSVKEDRLCYGGPWKLQHKWNGQDEVQMIVVEPGKNVKNVQTKPGVFKTPEGEIGAVTLDFPTGTSGSPIVDKNGDVIGLYGNGVIMPNGSYISAIVQGERMDEPIPAGFEPEMLRKKQITVLDLHPGAGKTRRILPQIIKEAINRRLRTAVLAPTRVVAAEMAEALRGLPIRYQTSAVPREHNGNEIVDVMCHATLTHRLMSPHRVPNYNLFVMDEAHFTDPASIAARGYISTKVELGEAAAIFMTATPPGTSDPFPESNSPISDLQTEIPDRAWNSGYEWITEYTGKTVWFVPSVKMGNEIALCLQRAGKKVVQLNRKSYETEYPKCKNDDWDFVITTDISEMGANFKASRVIDSRKSVKPTIITEGEGRVILGEPSAVTAASAAQRRGRIGRNPSQVGDEYCYGGHTNEDDSNFAHWTEARIMLDNINMPNGLIAQFYQPEREKVYTMDGEYRLRGEERKNFLELLRTADLPVWLAYKVAAAGVSYHDRRWCFDGPRTNTILEDNNEVEVITKLGERKILRPRWIDARVYSDHQALKAFKDFASGKRSQIGLIEVLGKMPEHFMGKTWEALDTMYVVATAEKGGRAHRMALEELPDALQTIALIALLSVMTMGVFFLLMQRKGIGKIGLGGAVLGVATFFCWMAEVPGTKIAGMLLLSLLLMIVLIPEPEKQRSQTDNQLAVFLICVMTLVSAVAANEMGWLDKTKSDISSLFGQRIEVKENFSMGEFLLDLRPATAWSLYAVTTAVLTPLLKHLITSDYINTSLTSINVQASALFTLARGFPFVDVGVSAFLLAAGCWGQVTLTVTVTAATLLFCHYAYMVPGWQAEAMRSAQRRTAAGIMKNAVVDGIVATDVPELERTTPIMQKKVGQIMLILVSLAAVVVNPSVKTVREAGILITAAAVTLWENGASSVWNATTAIGLCHIMRGGWLSCLSITWTLIKNMEKPGLKRGGAKGRTLGEVWKERLNQMTKEEFTRYRKEAIIEVDRSAAKHARKEGNVTGGHPVSRGTAKLRWLVERRFLEPVGKVIDLGCGRGGWCYYMATQKRVQEVRGYTKGGPGHEEPQLVQSYGWNIVTMKSGVDVFYRPSECCDTLLCDIGESSSSAEVEEHRTIRVLEMVEDWLHRGPREFCVKVLCPYMPKVIEKMELLQRRYGGGLVRNPLSRNSTHEMYWVSRASGNVVHSVNMTSQVLLGRMEKRTWKGPQYEEDVNLGSGTRAVGKPLLNSDTSKIKNRIERLRREYSSTWHHDENHPYRTWNYHGSYDVKPTGSASSLVNGVVRLLSKPWDTITNVTTMAMTDTTPFGQQRVFKEKVDTKAPEPPEGVKYVLNETTNWLWAFLAREKRPRMCSREEFIRKVNSNAALGAMFEEQNQWRSAREAVEDPKFWEMVDEEREAHLRGECHTCIYNMMGKREKKPGEFGKAKGSRAIWFMWLGARFLEFEALGFLNEDHWLGRKNSGGGVEGLGLQKLGYILREVGTRPGGKIYADDTAGWDTRITRADLENEAKVLELLDGEHRRLARAIIELTYRHKVVKVMRPAADGRTVMDVISREDQRGSGQVVTYALNTFTNLAVQLVRMMEGEGVIGPDDVEKLTKGKGPKVRTWLFENGEERLSRMAVSGDDCVVKPLDDRFATSLHFLNAMSKVRKDIQEWKPSTGWYDWQQVPFCSNHFTELIMKDGRTLVVPCRGQDELVGRARISPGAGWNVRDTACLAKSYAQMWLLLYFHRRDLRLMANAICSAVPVNWVPTGRTTWSIHAGGEWMTTEDMLEVWNRVWIEENEWMEDKTPVEKWSDVPYSGKREDIWCGSLIGTRARATWAENIQVAINQVRAIIGDEKYVDYMSSLKRYEDTTLVEDTVL

>ADK62440.1 polyprotein [West Nile virus]

MSKKPGGPGKSRAVNMLKRGMPRVLSLIGLKRAMLSLIDGKGPIRFVLALLAFFRFTAIAPTRAVLDRWRGVNKQTAMKHLLSFKKELGTLTSAINRRSSKQKKRGGKTGIAVMIGLIASVGAVTLSNFQGKVMMTVNATDVTDVITIPTAAGKNLCIVRAMDVGYMCDDTITYECPVLSAGNDPEDIDCWCTKSAVYVRYGRCTKTRHSRRSRRSLTVQTHGESTLANKKGAWMDSTKATRYLVKTESWILRNPGYALVAAVIGWMLGSNTMQRVVFVVLLLLVAPAYSFNCLGMSNRDFLEGVSGATWVDLVLEGDSCVTIMSKDKPTIDVKMMNMEAANLAEVRSYCYLATVSDLSTKAACPTMGEAHNDKRADPAFVCRQGVVDRGWGNGCGLFGKGSIDTCAKFACSTKAIGRTILKENIKYEVAIFVHGPTTVESHGNYSTQAGATQAGRFSITPAAPSYTLKLGEYGEVTVDCEPRSGIDTNAYYVMTVGTKTFLVHREWFMDLNLPWSSAGSTVWRNRETLMEFEEPHATKQSVIALGSQEGALHQALAGAIPVEFSSNTVKLTSGHLKCRVKMEKLQLKGTTYGVCSKAFKFLGTPADTGHGTVVLELQYTGTDGPCKVPISSVASLNDLTPVGRLVTVNPFVSVATANAKVLIELEPPFGDSYIVVGRGEQQINHHWHKSGSSIGKAFTTTLKGAQRLAALGDTAWDFGSVGGVFTSVGKAVHQVFGGAFRSLFGGMSWITQGLLGALLLWMGINARDRSIALTFLAVGGVLLFLSVNVHADTGCAIDISRQELRCGSGVFIHNDVEAWMDRYKYYPETPQGLAKIIQKAHKEGVCGLRSVSRLEHQMWEAVKDELNTLLKENGVDLSVVVEKQEGMYKSAPKRLTATTEKLEIGWKAWGKSILFAPELANNTFVVDGPETKECPTQNRAWNSLEVEDFGFGLTSTRMFLKVRESNTTECDSKIIGTAVKNNLAIHSDLSYWIESRLNDTWKLERAVLGEVKSCTWPETHTLWGDGILESDLIIPVTLAGPRSNHNRRPGYKTQNQGPWDEGRVEIDFDYCPGTTVTLSESCGHRGPATRTTTESGKLITDWCCRSCTLPPLRYQTDSGCWYGMEIRPQRHDEKTLVQSQVNAYNADMIDPFQLGLLVVFLATQEVLRKRWTAKISMPAILIALLVLVFGGITYTDVLRYVILVGAAFAESNSGGDVVHLALMATFKIQPVFMVASFLKARWTNQENILLMLAAVFFQMAYHDARQILLWEIPDVLNSLAVAWMILRAITFTTTSNVVVPLLALLTPGLRCLNLDVYRILLLMVGIGSLIREKRSAAAKKKGASLLCLALASTGLFNPMILAAGLIACDPNRKRGWPATEVMTAVGLMFAIVGGLAELDIDSMAIPMTIAGLMFAAFVISGKSTDMWIERTADISWESDAEITGSSERVDVRLDDDGNFQLMNDPGAPWKIWMLRMVCLAISAYTPWAILPSVVGFWITLQYTKRGGVLWDTPSPKEYKKGDTTTGVYRIMTRGLLGSYQAGAGVMVEGVFHTLWHTTKGAALMSGEGRLDPYWGSVKEDRLCYGGPWKLQHKWNGQDEVQMIVVEPGKNVKNVQTKPGVFKTPEGEIGAVTLDFPTGTSGSPIVDKNGDVIGLYGNGVIMPNGSYISAIVQGERMDEPIPAGFEPEMLRKKQITVLDLHPGAGKTRRILPQIIKEAINRRLRTAVLAPTRVVAAEMAEALRGLPIRYQTSAVPREHNGNEIVDVMCHATLTHRLMSPHRVPNYNLFVMDEAHFTDPASIAARGYISTKVELGEAAAIFMTATPPGTSDPFPESNSPISDLQTEIPDRAWNSGYEWITEYTGKTVWFVPSVKMGNEIALCLQRAGKKVVQLNRKSYETEYPKCKNDDWDFVITTDISEMGANFKASRVIDSRKSVKPTIITEGEGRVILGEPSAVTAASAAQRRGRIGRNPSQVGDEYCYGGHTNEDDSNFAHWTEARIMLDNINMPNGLIAQFYQPEREKVYTMDGEYRLRGEERKNFLELLRTADLPVWLAYKVAAAGVSYHDRRWCFDGPRTNTILEDNNEVEVITKLGERKILRPRWIDARVYSDHQALKAFKDFASGKRSQIGLIEVLGKMPEHFMGKTWEALDTMYVVATAEKGGRAHRMALEELPDALQTIALIALLSVMTMGVFFLLMQRKGIGKIGLGGAVLGVATFFCWMAEVPGTKIAGMLLLSLLLMIVLIPEPEKQRSQTDNQLAVFLICVMTLVSAVAANEMGWLDKTKSDISSLFGQRIEVKENFSMGEFLLDLRPATAWSLYAVTTAVLTPLLKHLITSDYINTSLTSINVQASALFTLSRGFPFVDVGVSALLLAAGCWGQVTLTVTVTAATLLFCHYAYMVPGWQAEAMRSAQRRTAAGIMKNAVVDGIVATDVPELERTTPIMQKKVGQIMLILVSLAAVVVNPSVKTVREAGILITAAAVTLWENGASSVWNATTAIGLCHIMRGGWLSCLSITWTLIKNMEKPGLKRGGAKGRTLGEVWKERLNQMTKEEFTRYRKEAIIEVDRSAAKHARKEGNVTGGHPVSRGTAKLRWLVERRFLEPVGKVIDLGCGRGGWCYYMATQKRVQEVRGYTKGGPGHEEPQLVQSYGWNIVTMKSGVDVFYRPSECCDTLLCDIGESSSSAEVEEHRTIRVLEMVEDWLHRGPREFCVKVLCPYMPKVIEKMELLQRRYGGGLVRNPLSRNSTHEMYWVSRASGNVVHSVNMTSQVLLGRMEKRTWKGPQYEEDVNLGSGTRAVGKPLLNSDTSKIKNRIERLRREYSSTWHHDENHPYRTWNYHGSYDVKPTGSASSLVNGVVRLLSKPWDTITNVTTMAMTDTTPFGQQRVFKEKVDTKAPEPPEGVKYVLNETTNWLWAFLAREKRPRMCSREEFIRKVNSNAALGAMFEEQNQWRSAREAVEDPKFWEMVDEEREAHLRGECHTCIYNMMGKREKKPGEFGKAKGSRAIWFMWLGARFLEFEALGFLNEDHWLGRKNSGGGVEGLGLQKLGYILREVGTRPGGKIYADDTAGWDTRITRADLENEAKVLELLDGEHRRLARAIIELTYRHKVVKVMRPAADGRTVMDVISREDQRGSGQVVTYALNTFTNLAVQLVRMMEGEGVIGPDDVEKLTKGKGPKVRTWLFENGEERLSRMAVSGDDCVVKPLDDRFATSLHFLNAMSKVRKDIQEWKPSTGWYDWQQVPFCSNHFTELIMKDGRTLVVPCRGQDELVGRARISPGAGWNVRDTACLAKSYAQMWLLLYFHRRDLRLMANAICSAVPVNWVPTGRTTWSIHAGGEWMTTEDMLEVWNRVWIEENEWMEDKTPVEKWSDVPYSGKREDIWCGSLIGTRARATWAENIQVAINQVRAIIGDEKYVDYMSSLKRYEDTTLVEDTVL

>AEB66121.1 polyprotein [West Nile virus]

MSKKPGGPGKSRAVNMLKRGMPRVLSLIGLKRAMLSLIDGKGPIRFVLALLAFFRFTAIAPTRAVLDRWRGVNKQTAMKHLLSFKKELGTLTSAINRRSSKQKKRGGKTGIAVMIGLIASVGAVTLSNFQGKVMMTVNATDVTDVITIPTAAGKNLCIVRAMDVGYMCDDTITYECPVLSAGNDPEDIDCWCTKSAVYVRYGRCTKTRHSRRSRRSLTVQTHGESTLANKKGAWMDSTKATRYLVKTESWILRNPGYALVAAVIGWMLGSNTMQRVVFVVLLLLVAPAYSFNCLGMSNRDFLEGVSGATWVDLVLEGDSCVTIMSKDKPTIDVKMMNMEAANLAEVRSYCYLATVSDLSTKAACPTMGEAHNDKRADPAFVCRQGVVDRGWGNGCGLFGKGSIDTCAKFACSTKAIGRTILKENIKYEVAIFVHGPTTVESHGNYSTQAGATQAGRFSITPAAPSYTLKLGEYGEVTVDCEPRSGIDTNAYYVMTVGTKTFLVHREWFMDLNLPWSSAGSTVWRNRETLMEFEEPHATKQSVIALGSQEGALHQALAGAIPVEFSSNTVKLTSGHLKCRVKMEKLQLKGTTYGVCSKAFKFLGTPADTGHGTVVLELQYTGTDGPCKVPISSVASLNDLTPVGRLVTVNPFVSVATANAKVLIELEPPFGDSYIVVGRGEQQINHHWHKSGSSIGKAFTTTLKGAQRLAALGDTAWDFGSVGGVFTSVGKAVHQVFGGAFRSLFGGMSWITQGLLGALLLWMGINARDRSIALTFLAVGGVLLFLSVNVHADTGCAIDISRQELRCGSGVFIHNDVEAWMDRYKYYPETPQGLAKIIQKAHKEGVCGLRSVSRLEHQMWEAVKDELNTLLKENGVDLSVVVEKQEGMYKSAPKRLTATTEKLEIGWKAWGKSILFAPELANNTFVVDGPETKECPTQNRAWNSLEVEDFGFGLTSTRMFLKVRESNTTECDSKIIGTAVKNNLAIHSDLSYWIESRLNDTWKLERAVLGEVKSCTWPETHTLWGDGILESDLIIPVTLAGPRSNHNRRPGYKTQNQGPWDEGRVEIDFDYCPGTTVTLSESCGHRGPATRTTTESGKLITDWCCRSCTLPPLRYQTDSGCWYGMEIRPQRHDEKTLVQSQVNAYNADMIDPFQLGLLVVFLATQEVLRKRWTAKISMPAILIALLVLVFGGITYTDVLRYVILVGAAFAESNSGGDVVHLALMATFKIQPVFMVASFLKARWTNQENILLMLAAVFFQMAYHDARQILLWEIPDVLNSLAVAWMILRAITFTTTSNVVVPLLALLTPGLRCLNLDVYRILLLMVGIGSLIREKRSAAAKKKGASLLCLALASTGLFNPMILAAGLIACDPNRKRGWPATEVMTAVGLMFAIVGGLAELDIDSMAIPMTIAGLMFAAFVISGKSTDMWIERTADISWESDAEITGSSERVDVRLDDDGNFQLMNDPGAPWKIWMLRMVCLAISAYTPWAILPSVVGFWITLQYTKRGGVLWDTPSPKEYKKGDTTTGVYRIMTRGLLGSYQAGAGVMVEGVFHTLWHTTKGAALMSGEGRLDPYWGSVKEDRLCYGGPWKLQHKWNGQDEVQMIVVEPGKNVKNVQTKPGVFKTPEGEIGAVTLDFPTGTSGSPIVDKNGDVIGLYGNGVIMPNGSYISAIVQGERMDEPIPAGFEPEMLRKKQITVLDLHPGAGKTRRILPQIIKEAINRRLRTAVLAPTRVVAAEMAEALRGLPIRYQTSAVPREHNGNEIVDVMCHATLTHRLMSPHRVPNYNLFVMDEAHFTDPASIAARGYISTKVELGEAAAIFMTATPPGTSDPFPESNSPISDLQTEIPDRAWNSGYEWITEYTGKTVWFVPSVKMGNEIALCLQRAGKKVVQLNRKSYETEYPKCKNDDWDFVITTDISEMGANFKASRVIDSRKSVKPTIITEGEGRVILGEPSAVTAASAAQRRGRIGRNPSQVGDEYCYGGHTNEDDSNYAHWTEARIMLDNINMPNGLIAQFYQPEREKVYTMDGEYRLRGEERKNFLELLRTADLPVWLAYKVAAAGVSYHDRRWCFDGPRTNTILEDNNEVEVITKLGERKILRPRWIDARVYSDHQALKAFKDFASGKRSQIGLIEVLGKMPEHFMGKTWEALDTMYVVATAEKGGRAHRMALEELPDALQTIALIALLSVMTMGVFFLLMQRKGIGKIGLGGAVLGVATFFCWMAEVPGTKIAGMLLLSLLLMIVLIPEPEKQRSQTDNQLAVFLICVMTLVSAVAANEMGWLDKTKSDISSLFGQRIEVKENFSMGEFLLDLRPATAWSLYAVTTAVLTPLLKHLITSDYINTSLTSINVQASALFTLARGFPFVDVGVSALLLAAGCWGQVTLTVTVTAATLLFCHYAYMVPGWQAEAMRSAQRRTAAGIMKNAVVDGIVATDVPELERTTPIMQKKVGQIMLILVSLAAVVVNPSVKTVREAGILITAAAVTLWENGASSVWNATTAIGLCHIMRGGWLSCLSITWTLIKNMEKPGLKRGGAKGRTLGEVWKERLNQMTKEEFTRYRKEAIIEVDRSAAKHARKEGNVTGGHPVSRGTAKLRWLVERRFLEPVGKVIDLGCGRGGWCYYMATQKRVQEVRGYTKGGPGHEEPQLVQSYGWNIVTMKSGVDVFYRPSECCDTLLCDIGESSSSAEVEEHRTIRVLEMVEDWLHRGPREFCVKVLCPYMPKVIEKMELLQRRYGGGLVRNPLSRNSTHEMYWVSRASGNVVHSVNMTSQVLLGRMEKRTWKGPQYEEDVNLGSGTRAVGKPLLNSDTSKIKNRIERLRREYSSTWHHDENHPYRTWNYHGSYDVKPTGSASSLVNGVVRLLSKPWDTITNVTTMAMTDTTPFGQQRVFKEKVDTKAPEPPEGVKYVLNETTNWLWAFLAREKRPRMCSREEFIRKVNSNAALGAMFEEQNQWRSAREAVEDPKFWEMVDEEREAHLRGECHTCIYNMMGKREKKPGEFGKAKGSRAIWFMWLGARFLEFEALGFLNEDHWLGRKNSGGGVEGLGLQKLGYILREVGTRPGGKIYADDTAGWDTRITRADLENEAKVLELLDGEHRRLARAIIELTYRHKVVKVMRPAADGRTVMDVISREDQRGSGQVVTYALNTFTNLAVQLVRMMEGEGVIGPDDVEKLTKGKGPKVRTWLFENGEERLSRMAVSGDDCVVKPLDDRFATSLHFLNAMSKVRKDIQEWKPSTGWYDWQQVPFCSNHFTELIMKDGRTLVVPCRGQDELVGRARISPGAGWNVRDTACLAKSYAQMWLLLYFHRRDLRLMANAICSAVPVNWVPTGRTTWSIHAGGEWMTTEDMLEVWNRVWIEENEWMEDKTPVEKWSDVPYSGKREDIWCGSLIGTRARATWAENIQVAINQVRAIIGDEKYVDYMSSLKRYEDTTLVEDTVL

>AHW48482.1 polyprotein [West Nile virus]

MSKKPGGPGKSRAVNMLKRGMPRVLSLIGLKRAMLSLIDGKGPIRFVLALLAFFRFTAIAPTRAVLDRWRGVNKQTAMKHLLSFKKELGTLTSAINRRSSKQKKRGGKTGIAVMIGLIASVGAVTLSNFQGKVMMTVNATDVTDVITIPTAAGKNLCIVRAMDVGYMCDDTITYECPVLSAGNDPEDIDCWCTKSAVYVRYGRCTKTRHSRRSRRSLTVQTHGESTLANKKGAWMDSTKATRYLVKTESWILRNPGYALVAAVIGWMLGSNTMQRVVFVVLLLLVAPAYSFNCLGMSNRDFLEGVSGATWVDLVLEGDSCVTIMSKDKPTIDVKMMNMEAANLAEVRSYCYLATVSDLSTKAACPTMGEAHNDKRADPAFVCRQGVVDRGWGNGCGLFGKGSIDTCAKFACSTKAIGRTILKENIKYEVAIFVHGPTTVESHGNYSTQAGATQAGRFSITPAAPSYTLKLGEYGEVTVDCEPRSGIDTNAYYVMTVGTKTFLVHREWFMDLNLPWSSAGSTVWRNRETLMEFEEPHATKQSVIALGSQEGALHQALAGAIPVEFSSNTVKLTSGHLKCRVKMEKLQLKGTTYGVCSKAFKFLGTPADTGHGTVVLELQYTGTDGPCKVPISSVASLNDLTPVGRLVTVNPFVSVATANAKVLIELEPPFGDSYIVVGRGEQQINHHWHKSGSSIGKAFTTTLKGAQRLAALGDTAWDFGSVGGVFTSVGKAVHQVFGGAFRSLFGGMSWITQGLLGALLLWMGINARDRSIALTFLAVGGVLLFLSVNVHADTGCAIDISRQELRCGSGVFIHNDVEAWMDRYKYYPETPQGLAKIIQKAHKEGVCGLRSVSRLEHQMWEAVKDELNTLLKENGVDLSVVVEKQEGMYKSAPKRLTATTEKLEIGWKAWGKSILFAPELANNTFVVDGPETKECPTQNRAWNSLEVEDFGFGLTSTRMFLKVRESNTTECDSKIIGTAVKNNLAIHSDLSYWIESRLNDTWKLERAVLGEVKSCTWPETHTLWGDGILESDLIIPVTLAGPRSNHNRRPGYKTQNQGPWDEGRVEIDFDYCPGTTVTLSESCGHRGPATRTTTESGKLITDWCCRSCTLPPLRYQTDSGCWYGMEIRPQRHDEKTLVQSQVNAYNADMIDPFQLGLLVVFLATQEVLRKRWTAKISMPAILIALLVLVFGGITYTDVLRYVILVGAAFAESNSGGDVVHLALMATFKIQPVFMVASFLKARWTNQENILLMLAAVFFQMAYHDARQILLWEIPDVLNSLAVAWMILRAITFTTTSNVVVPLLALLTPGLRCLNLDVYRILLLMVGIGSLIREKRSAAAKKKGASLLCLALASTGLFNPMILAAGLIACDPNRKRGWPATEVMTAVGLMFAIVGGLAELDIDSMAIPMTIAGLMFAAFVISGKSTDMWIERTADISWESDAEITGSSERVDVRLDDDGNFQLMNDPGAPWKIWMLRMVCLAISAYTPWAILPSVVGFWITLQYTKRGGVLWDTPSPKEYKKGDTTTGVYRIMTRGLLGSYQAGAGVMVEGVFHTLWHTTKGAALMSGEGRLDPYWGSVKEDRLCYGGPWKLQHKWNGQDEVQMIVVEPGKNVKNVQTKPGVFKTPEGEIGAVTLDFPTGTSGSPIVDKNGDVIGLYGNGVIMPNGSYISAIVQGERMDEPIPAGFEPEMLRKKQITVLDLHPGAGKTRRILPQIIKEAINRRLRTAVLAPTRVVAAEMAEALRGLPIRYQTSAVPREHNGNEIVDVMCHATLTHRLMSPHRVPNYNLFVMDEAHFTDPASIAARGYISTKVELGEAAAIFMTATPPGTSDPFPESNSPISDLQTEIPDRAWNSGYEWITEYTGKTVWFVPSVKMGNEIALCLQRAGKKVVQLNRKSYETEYPKCKNDDWDFVITTDISEMGANFKASRVIDSRKSVKPTIITEGEGRVILGEPSAVTAASAAQRRGRIGRNPSQVGDEYCYGGHTNEDDSNFAHWTEARIMLDNINMPNGLIAQFYQPEREKVYTMDGEYRLRGEERKNFLELLRTADLPVWLAYKVAAAGVSYHDRRWCFDGPRTNTILEDNNEVEVITKLGERKILRPRWIDARVYSDHQALKAFKDFASGKRSQIGLIEVLGKMPEHFMGKTWEALDTMYVVATAEKGGRAHRMALEELPDALQTIALIALLSVMTMGVFFLLMQRKGIGKIGLGGAVLGVATFFCWMAEVPGTKIAGMLLLSLLLMIVLIPEPEKQRSQTDNQLAVFLICVMTLVSAVAANEMGWLDKTKSDISSLFGQRIEVKENFSMGEFLLDLRPATAWSLYAVTTAVLTPLLKHLITSDYINTSLTSINVQASALFTLARGFPFVDVGVSALLLAAGCWGQVTLTVTVTAATLLFCHYAYMVPGWQAEAMRSAQRRTAAGIMKNAVVDGIVATDVPELERTTPIMQKKVGQIMLILVSLAAVVVNPSVKTVREAGILITAAAVTLWENGASSVWNATTAIGLCHIMRGGWLSCLSMTWTLIKNMEKPGLKRGGAKGRTLGEVWKERLNQMTKEEFTRYRKEAIIEVDRSAAKHARKEGNVTGGHPVSRGTAKLRWLVERRFLEPVGKVIDLGCGRGGWCYYMATQKRVQEVRGYTKGGPGHEEPQLVQSYGWNIVTMKSGVDVFYRPSECCDTLLCDIGESSSSAEVEEHRTIRVLEMVEDWLHRGPREFCVKVLCPYMPKVIEKMELLQRRYGGGLVRNPLSRNSTHEMYWVSRASGNVVHSVNMTSQVLLGRMEKRTWKGPQYEEDVNLGSGTRAVGKPLLNSDTSKIKNRIERLRREYSSTWHHDENHPYRTWNYHGSYDVKPTGSASSLVNGVVRLLSKPWDTITNVTTMAMTDTTPFGQQRVFKEKVDTKAPEPPEGVKYVLNETTNWLWAFLAREKRPRMCSREEFIRKVNSNAALGAMFEEQNQWRSAREAVEDPKFWEMVDEEREAHLRGECHTCIYNMMGKREKKPGEFGKAKGSRAIWFMWLGARFLEFEALGFLNEDHWLGRKNSGGGVEGLGLQKLGYILREVGTRPGGKIYADDTAGWDTRITRADLENEAKVLELLDGEHRRLARAIIELTYRHKVVKVMRPAADGRTVMDVISREDQRGSGQVVTYALNTFTNLAVQLVRMMEGEGVIGPDDVEKLTKGKGPKVRTWLFENGEERLSRMAVSGDDCVVKPLDDRFATSLHFLNAMSKVRKDIQEWKPSTGWYDWQQVPFCSNHFTELIMKDGRTLVVPCRGQDELVGRARISPGAGWNVRDTACLAKSYAQMWLLLYFHRRDLRLMANAICSAVPVNWVPTGRTTWSIHAGGEWMTTEDMLEVWNRVWIEENEWMEDKTPVEKWSDVPYSGKREDIWCGSLIGTRARATWAENIQVAINQVRAIIGDEKYVDYMSSLKRYEDTTLVEDTVL

>AGW25443.1 polyprotein [West Nile virus]

MSKKPGGPGKSRAVNMLKRGMPRVLSLIGLKRAMLSLIDGKGPIRFVLALLAFFRFTAIAPTRAVLDRWRGVNKQTAMKHLLSFKKELGTLTSAINRRSSKQKKRGGKTGIAVMIGLIASVGAVTLSNFQGKVMMTVNATDVTDVITIPTAAGKNLCIVRAMDVGYMCDDTITYECPVLSAGNDPEDIDCWCTKSAVYVRYGRCTKTRHSRRSRRSLTVQTHGESTLANKKGAWMDSTKATRYLVKTESWILRNPGYALVAAVIGWMLGSNTMQRVVFVVLLLLVAPAYSFNCLGMSNRDFLEGVSGATWVDLVLEGDSCVTIMSKDKPTIDVKMMNMEAANLAEVRSYCYLATVSDLSTKAACPTMGEAHNDKRADPAFVCRQGVVDRGWGNGCGLFGKGSIDTCAKFACSTKAIGRTILKENIKYEVAIFVHGPTTVESHGNYSTQAGATQAGRFSITPAAPSYTLKLGEYGEVTVDCEPRSGIDTNAYYVMTVGTKTFLVHREWFMDLNLPWSSAGSTVWRNRETLMEFEEPHATKQSVIALGSQEGALHQALAGAIPVEFSSNTVKLTSGHLKCRVKMEKLQLKGTTYGVCSKAFKFLGTPADTGHGTVVLELQYTGTDGPCKVPISSVASLNDLTPVGRLVTVNPFVSVATANAKVLIELEPPFGDSYIVVGRGEQQINHHWHKSGSSIGKAFTTTLKGAQRLAALGDTAWDFGSVGGVFTSVGKAVHQVFGGAFRSLFGGMSWITQGLLGALLLWMGINARDRSIALTFLAVGGVLLFLSVNVHADTGCAIDISRQELRCGSGVFIHNDVEAWMDRYKYYPETPQGLAKIIQKAHKEGVCGLRSVSRLEHQMWEAVKDELNTLLKENGVDLSVVVEKQEGMYKSAPKRLTATTEKLEIGWKAWGKSILFAPELANNTFVVDGPETKECPTQNRAWNSLEVEDFGFGLTSTRMFLKVRESNTTECDSKIIGTAVKNNLAIHSDLSYWIESRLNDTWKLERAVLGEVKSCTWPETHTLWGDGILESDLIIPVTLAGPRSNHNRRPGYKTQNQGPWDEGRVEIDFDYCPGTTVTLSESCGHRGPATRTTTESGKLITDWCCRSCTLPPLRYQTDSGCWYGMEIRPQRHDEKTLVQSQVNAYNADMIDPFQLGLLVVFLATQEVLRKRWTAKISMPAILIALLVLVFGGITYTDVLRYVILVGAAFAESNSGGDVVHLALMATFKIQPVFMVASFLKARWTNQENILLMLAAVFFQMAYHDARQILLWEIPDVLNSLAVAWMILRAITFTTTSNVVVPLLALLTPGLRCLNLDVYRILLLMVGIGSLIREKRSAAAKKKGASLLCLALASTGLFNPMILAAGLIACDPNRKRGWPATEVMTAVGLMFAIVGGLAELDIDSMAIPMTIAGLMFAAFVISGKSTDMWIERTADISWESDAEITGSSERVDVRLDDDGNFQLMNDPGAPWKIWMLRMVCLAISAYTPWAILPSVVGFWITLQYTKRGGVLWDTPSPKEYKKGDTTTGVYRIMTRGLLGSYQAGAGVMVEGVFHTLWHTTKGAALMSGEGRLDPYWGSVKEDRLCYGGPWKLQHKWNGQDEVQMIVVEPGKNVKNVQTKPGVFKTPEGEIGAVTLDFPTGTSGSPIVDKNGDVIGLYGNGVIMPNGSYISAIVQGERMDEPIPAGFDPEMLRKKQITVLDLHPGAGKTRRILPQIIKEAINRRLRTAVLAPTRVVAAEMAEALRGLPIRYQTSAVPREHNGNEIVDVMCHATLTHRLMSPHRVPNYNLFVMDEAHFTDPASIAARGYISTKVELGEAAAIFMTATPPGTSDPFPESNSPISDLQTEIPDRAWNSGYEWITEYTGKTVWFVPSVKMGNEIALCLQRAGKKVVQLNRKSYETEYPKCKNDDWDFVITTDISEMGANFKASRVIDSRKSVKPTIITEGEGRVILGEPSAVTAASAAQRRGRIGRNPSQVGDEYCYGGHTNEDDSNFAHWTEARIMLDNINMPNGLIAQFYQPEREKVYTMDGEYRLRGEERKNFLELLRTADLPVWLAYKVAAAGVSYHDRRWCFDGPRTNTILEDNNEVEVITKLGERKILRPRWIDARVYSDHQALKAFKDFASGKRSQIGLIEVLGKMPEHFMGKTWEALDTMYVVATAEKGGRAHRMALEELPDALQTIALIALLSVMTMGVFFLLMQRKGIGKIGLGGAVLGVATFFCWMAEVPGTKIAGMLLLSLLLMIVLIPEPEKQRSQTDNQLAVFLICVMTLVSAVAANEMGWLDKTKSDISSLFGQRIEVKENFSMGEFLLDLRPATAWSLYAVTTAVLTPLLKHLITSDYINTSLTSINVQASALFTLARGFPFVDVGVSALLLAAGCWGQVTLTVTVTAATLLFCHYAYMVPGWQAEAMRSAQRRTAAGIMKNAVVDGIVATDVPELERTTPIMQKKVGQIMLILVSLAAVVVNPSVKTVREAGILITAAAVTLWENGASSVWNATTAIGLCHIMRGGWLSCLSITWTLIKNMEKPGLKRGGAKGRTLGEVWKERLNQMTKEEFTRYRKEAIIEVDRSAAKHARKEGNVTGGHPVSRGTAKLRWLVERRFLEPVGKVIDLGCGRGGWCYYMATQKRVQEVRGYTKGGPGHEEPQLVQSYGWNIVTMKSGVDVFYRPSECCDTLLCDIGESSSSAEVEEHRTIRVLEMVEDWLHRGPREFCVKVLCPYMPKVIEKMELLQRRYGGGLVRNPLSRNSTHEMYWVSRASGNVVHSVNMTSQVLLGRMEKRTWKGPQYEEDVNLGSGTRAVGKPLLNSDTSKIKNRIERLRREYSSTWHHDENHPYRTWNYHGSYDVKPTGSASSLVNGVVRLLSKPWDTITNVTTMAMTDTTPFGQQRVFKEKVDTKAPEPPEGVKYVLNETTNWLWAFLAREKRPRMCSREEFIRKVNSNAALGAMFEEQNQWRSAREAVEDPKFWEMVDEEREAHLRGECHTCIYNMMGKREKKPGEFGKAKGSRAIWFMWLGARFLEFEALGFLNEDHWLGRKNSGGGVEGLGLQKLGYILREVGTRPGGKIYADDTAGWDTRITRADLENEAKVLELLDGEHRRLARAIIELTYRHKVVKVMRPAADGRTVMDVISREDQRGSGQVVTYALNTFTNLAVQLVRMMEGEGVIGPDDVEKLTKGKGPKVRTWLFENGEERLSRMAVSGDDCVVKPLDDRFATSLHFLNAMSKVRKDIQEWKPSTGWYDWQQVPFCSNHFTELIMKDGRTLVVPCRGQDELVGRARISPGAGWNVRDTACLAKSYAQMWLLLYFHRRDLRLMANAICSAVPVNWVPTGRTTWSIHAGGEWMTTEDMLEVWNRVWIEENEWMEDKTPVEKWSDVPYSGKREDIWCGSLIGTRARATWAENIQVAINQVRAIIGDEKYVDYMSSLKRYEDTTLVEDTVL

>ANW69076.1 genome polyprotein [West Nile virus]

MSKKPGGPGKSRAVNMLKRGMPRVLSLIGLKRAMLSLIDGKGPIRFVLALLAFFRFTAIAPTRAVLDRWRGVNKQTAMKHLLSFKKELGTLTSAINRRSSKQKKRGGKTGIAVMIGLIASVGAVTLSNFQGKVMMTVNATDVTDVITIPTAAGKNLCIVRAMDVGYMCDDTITYECPVLSAGNDPEDIDCWCTKSAVYVRYGRCTKTRHSRRSRRSLTVQTHGESTLANKKGAWMDSTKATRYLVKTESWILRNPGYALVAAVIGWMLGSNTMQRVVFVVLLLLVAPAYSFNCLGMSNRDFLEGVSGATWVDLVLEGDSCVTIMSKDKPTIDVKMMNMEAANLAEVRSYCYLATVSDLSTKAACPTMGEAHNDKRADPAFVCRQGVVDRGWGNGCGLFGKGSIDTCAKFACSTKAIGRTILKENIKYEVAIFVHGPTTVESHGNYSTQAGATQAGRFSITPAAPSYTLKLGEYGEVTVDCEPRSGIDTNAYYVMTVGTKTFLVHREWFMDLNLPWSSAGSTVWRNRETLMEFEEPHATKQSVIALGSQEGALHQALAGAIPVEFSSNTVKLTSGHLKCRVKMEKLQLKGTTYGVCSKAFKFLGTPADTGHGTVVLELQYTGTDGPCKVPISSVASLNDLTPVGRLVTVNPFVSVATANAKVLIELEPPFGDSYIVVGRGEQQINHHWHKSGSSIGKAFTTTLKGAQRLAALGDTAWDFGSVGGVFTSVGKAVHQVFGGAFRSLFGGMSWITQGLLGALLLWMGINARDRSIALTFLAVGGVLLFLSVNVHADTGCAIDISRQELRCGSGVFIHNDVEAWMDRYKYYPETPQGLAKIIQKAHKEGVCGLRSVSRLEHQMWEAVKDELNTLLKENGVDLSVVVEKQEGMYKSAPKRLTATTEKLEIGWKAWGKSILFAPELANNTFVVDGPETKECPTQNRAWNSLEVEDFGFGLTSTRMFLKVRESNTTECDSKIIGTAVKNNLAIHSDLSYWIESRLNDTWKLERAVLGEVKSCTWPETHTLWGDGILESDLIIPVTLAGPRSNHNRRPGYKTQNQGPWDEGRVEIDFDYCPGTTVTLSESCGHRGPATRTTTESGKLITDWCCRSCTLPPLRYQTDSGCWYGMEIRPQRHDEKTLVQSQVNAYNADMIDPFQLGLLVVFLATQEVLRKRWTAKISMPAILIALLVLVFGGITYTDVLRYVILVGAAFAESNSGGDVVHLALMATFKIQPVFMVASFLKARWTNQENILLMLAAVFFQMAYHDARQILLWEIPDVLNSLAVAWMILRAITFTTTSNVVVPLLALLTPGLRCLNLDVYRILLLMVGIGSLIREKRSAAAKKKGASLLCLALASTGLFNPMILAAGLIACDPNRKRGWPATEVMTAVGLMFAIVGGLAELDIDSMAIPMTIAGLMFAAFVISGKSTDMWIERTADISWESDAEITGSSERVDVRLDDDGNFQLMNDPGAPWKIWMLRMVCLAISAYTPWAILPSVVGFWITLQYTKRGGVLWDTPSPKEYKKGDTTTGVYRIMTRGLLGSYQAGAGVMVEGVFHTLWHTTKGAALMSGEGRLDPYWGSVKEDRLCYGGPWKLQHKWNGQDEVQMIVVEPGKNVKNVQTKPGVFKTPEGEIGAVTLDFPTGTSGSPIVDKNGDVIGLYGNGVIMPNGSYISAIVQGERMDEPIPAGFEPEMLRKKQITVLDLHPGAGKTRRILPQIIKEAINRRLRTAVLAPTRVVAAEMAEALRGLPIRYQTSAVPREHNGNEIVDVMCHATLTHRLMSPHRVPNYNLFVMDEAHFTDPASIAARGYISTKVELGEAAAIFMTATPPGTSDPFPESNTPISDLQTEIPDRAWNSGYEWITEYTGKTVWFVPSVKMGNEIALCLQRAGKKVVQLNRKSYETEYPKCKNDDWDFVITTDISEMGANFKASRVIDSRKSVKPTIITEGEGRVILGEPSAVTAASAAQRRGRIGRNPSQVGDEYCYGGHTNEDDSNFAHWTEARIMLDNINMPNGLIAQFYQPEREKVYTMDGEYRLRGEERKNFLELLRTADLPVWLAYKVAAAGVSYHDRRWCFDGPRTNTILEDNNEVEVITKLGERKILRPRWIDARVYSDHQALKAFKDFASGKRSQIGLIEVLGKMPEHFMGKTWEALDTMYVVATAEKGGRAHRMALEELPDALQTIALIALLSVMTMGVFFLLMQRKGIGKIGLGGAVLGVATFFCWMAEVPGTKIAGMLLLSLLLMIVLIPEPEKQRSQTDNQLAVFLICVMTLVSAVAANEMGWLDKTKSDISSLFGQRIEVKENFSMGEFLLDLRPATAWSLYAVTTAVLTPLLKHLITSDYINTSLTSINVQASALFTLARGFPFVDVGVSALLLAAGCWGQVTLTVTVTAATLLFCHYAYMVPGWQAEAMRSAQRRTAAGIMKNAVVDGIVATDVPELERTTPIMQKKVGQIMLILVSLAAVVVNPSVKTVREAGILITAAAVTLWENGASSVWNATTAIGLCHIMRGGWLSCLSITWTLIKNMEKPGLKRGGAKGRTLGEVWKERLNQMTKEEFTRYRKEAIIEVDRSAAKHARKEGNVTGGHPVSRGTAKLRWLVERRFLEPVGKVIDLGCGRGGWCYYMATQKRVQEVRGYTKGGPGHEEPQLVQSYGWNIVTMKSGVDVFYRPSECCDTLLCDIGESSSSAEVEEHRTIRVLEMVEDWLHRGPREFCVKVLCPYMPKVIEKMELLQRRYGGGLVRNPLSRNSTHEMYWVSRASGNVVHSVNMTSQVLLGRMEKRTWKGPQYEEDVNLGSGTRAVGKPLLNSDTSKIKNRIERLRREYSSTWHHDENHPYRTWNYHGSYDVKPTGSASSLVNGVVRLLSKPWDTITNVTTMAMTDTTPFGQQRVFKEKVDTKAPEPPEGVKYVLNETTNWLWAFLAREKRPRMCSREEFIRKVNSNAALGAMFEEQNQWRSAREAVEDPKFWEMVDEEREAHLRGECHTCIYNMMGKREKKPGEFGKAKGSRAIWFMWLGARFLEFEALGFLNEDHWLGRKNSGGGVEGLGLQKLGYILREVGTRPGGKIYADDTAGWDTRITRADLENEAKVLELLDGEHRRLARAIIELTYRHKVVKVMRPAADGRTVMDVISREDQRGSGQVVTYALNTFTNLAVQLVRMMEGEGVIGPDDVEKLTKGKGPKVRTWLFENGEERLSRMAVSGDDCVVKPLDDRFATSLHFLNAMSKVRKDIQEWKPSTGWYDWQQVPFCSNHFTELIMKDGRTLVVPCRGQDELVGRARISPGAGWNVRDTACLAKSYAQMWLLLYFHRRDLRLMANAICSAVPVNWVPTGRTTWSIHAGGEWMTTEDMLEVWNRVWIEENEWMEDKTPVEKWSDVPYSGKREDIWCGSLIGTRARATWAENIQVAINQVRAIIGDEKYVDYMSSLKRYEDTTLVEDTVL

>ANW69610.1 genome polyprotein [West Nile virus]

MSKKPGGPGKSRAVNMLKRGMPRVLSLIGLKRAMLSLIDGKGPIRFVLALLAFFRFTAIAPTRAVLDRWRGVNKQTAMKHLLSFKKELGTLTSAINRRSSKQKKRGGKTGIAVMIGLIASVGAVTLSNFQGKVMMTVNATDVTDVITIPTAAGKNLCIVRAMDVGYMCDDTITYECPVLSAGNDPEDIDCWCTKSAVYVRYGRCTKTRHSRRSRRSLTVQTHGESTLANKKGAWMDSTKATRYLVKTESWILRNPGYALVAAVIGWMLGSNTMQRVVFVVLLLLVAPAYSFNCLGMSNRDFLEGVSGATWVDLVLEGDSCVTIMSKDKPTIDVKMMNMEAANLAEVRSYCYLATVSDLSTKAACPTMGEAHNDKRADPAFVCRQGVVDRGWGNGCGLFGKGSIDTCAKFACSTKAIGRTILKENIKYEVAIFVHGPTTVESHGNYSTQAGATQAGRFSITPAAPSYTLKLGEYGEVTVDCEPRSGIDTNAYYVMTVGTKTFLVHREWFMDLNLPWSSAGSTVWRNRETLMEFEEPHATKQSVIALGSQEGALHQALAGAIPVEFSSNTVKLTSGHLKCRVKMEKLQLKGTTYGVCSKAFKFLGTPADTGHGTVVLELQYTGTDGPCKVPISSVASLNDLTPVGRLVTVNPFVSVATANAKVLIELEPPFGDSYIVVGRGEQQINHHWHKSGSSIGKAFTTTLKGAQRLAALGDTAWDFGSVGGVFTSVGKAVHQVFGGAFRSLFGGMSWITQGLLGALLLWMGINARDRSIALTFLAVGGVLLFLSVNVHADTGCAIDISRQELRCGSGVFIHNDVEAWMDRYKYYPETPQGLAKIIQKAHKEGVCGLRSVSRLEHQMWEAVKDELNTLLKENGVDLSVVVEKQEGMYKSAPKRLTATTEKLEIGWKAWGKSILFAPELANNTFVVDGPETKECPTQNRAWNSLEVEDFGFGLTSTRMFLKVRESNTTECDSKIIGTAVKNNLAIHSDLSYWIESRLNDTWKLERAVLGEVKSCTWPETHTLWGDGILESDLIIPVTLAGPRSNHNRRPGYKTQNQGPWDEGRVEIDFDYCPGTTVTLSESCGHRGPATRTTTESGKLITDWCCRSCTLPPLRYQTDSGCWYGMEIRPQRHDEKTLVQSQVNAYNADMIDPFQLGLLVVFLATQEVLRKRWTAKISMPAILIALLVLVFGGITYTDVLRYVILVGAAFAESNSGGDVVHLALMATFKIQPVFMVASFLKARWTNQENILLMLAAVFFQMAYHDARQILLWEIPDVLNSLAVAWMILRAITFTTTSNVVVPLLALLTPGLRCLNLDVYRILLLMVGIGSLIREKRSAAAKKKGASLLCLALASTGLFNPMILAAGLIACDPNRKRGWPATEVMTAVGLMFAIVGGLAELDIDSMAIPMTIAGLMFAAFVISGKSTDMWIERTADISWESDAEITGSSERVDVRLDDDGNFQLMNDPGAPWKIWMLRMVCLAISAYTPWAILPSVVGFWITLQYTKRGGVLWDTPSPKEYKKGDTTTGVYRIMTRGLLGSYQAGAGVMVEGVFHTLWHTTKGAALMSGEGRLDPYWGSVKEDRLCYGGPWKLQHKWNGQDEVQMIVVEPGKNVKNVQTKPGVFKTPEGEIGAVTLDFPTGTSGSPIVDKNGDVIGLYGNGVIMPNGSYISAIVQGERMDEPIPAGFEPEMLRKKQITVLDLHPGAGKTRRILPQIIKEAINRRLRTAVLAPTRVVAAEMAEALRGLPIRYQTSAVPREHNGNEIVDVMCHATLTHRLMSPHRVPNYNLFVMDEAHFTDPASIAARGYISTKVELGEAAAIFMTATPPGTSDPFPESNSPISDLQTEIPDRAWNSGYEWITEYTGKTVWFVPSVKMGNEIALCLQRAGKKVVQLNRKSYETEYPKCKNDDWDFVITTDISEMGANFKASRVIDSRKSVKPTIITEGEGRVILGEPSAVTAASAAQRRGRIGRNPSQVGDEYCYGGHTNEDDSNFAHWTEARIMLDNINMPNGLIAQFYQPEREKVYTMDGEYRLRGEERKNFLELLRTADLPVWLAYKVAAAGVSYHDRRWCFDGPRTNTILEDNNEVEVITKLGERKILRPRWIDARVYSDHQALKAFKDFASGKRSQIGLIEVLGKMPEHFMGKTWEALDTMYVVATAEKGGRAHRMALEELPDALQTIALIALLSVMTMGVFFLLMQRKGIGKIGLGGAVLGVATFFCWMAEVPGTKIAGMLLLSLLLMIVLIPEPEKQRSQTDNQLAVFLICVMTLVSAVAANEMGWLDKTKSDISSLFGQRIEVKENFSMGEFLLDLRPATAWSLYAVTTAVLTPLLKHLITSDYINTSLTSINVQASALFTLARGFPFVDVGVSALLLAAGCWGQVTLTVTVTAATLLFCHYAYMVPGWQAEAMRSAQRRTAAGIMKNAVVDGIVATDVPELERTTPIMQKKVGQIMLILVSLAAVVVNPSVKTVREAGILITAAAVTLWENGASSVWNATTAIGLCHIMRGGWLSCLSITWTLIKNMEKPGLKRGGAKGRTLGEVWKERLNQMTKEEFTRYRKEAIIEVDRSAAKHARKEGNVTGGHPVSRGTAKLRWLVERRFLEPVGKVIDLGCGRGGWCYYMATQKRVQEVRGYTKGGPGHEEPQLVQSYGWNIVTMKSGVDVFYRPSECCDTLLCDIGESSSSAEVEEHRTIRVLEMVEDWLHRGPREFCVKVLCPYMPKVIEKMELLQRRYGGGLVRNPLSRNSTHEMYWVSRASGNVVHSVNMTSQVLLGRMEKRTWKGPQYEEDVNLGSGTRAVGKPLLNSDTSKIKNRIERLRREYSSTWHHDENHPYRTWNYHGSYDVKPTGSASSLVNGVVRLLSKPWDTITNVTTMAMTDTTPFGQQRVFKEKVDTKAPEPPEGVKYVLNETTNWLWAFLAREKRPRMCSREEFIRKVNSNAALGAMFEEQNQWKSAREAVEDPKFWEMVDEEREAHLRGECHTCIYNMMGKREKKPGEFGKAKGSRAIWFMWLGARFLEFEALGFLNEDHWLGRKNSGGGVEGLGLQKLGYILREVGTRPGGKIYADDTAGWDTRITRADLENEAKVLELLDGEHRRLARAIIELTYRHKVVKVMRPAADGRTVMDVISREDQRGSGQVVTYALNTFTNLAVQLVRMMEGEGVIGPDDVEKLTKGKGPKVRTWLFENGEERLSRMAVSGDDCVVKPLDDRFATSLHFLNAMSKVRKDIQEWKPSTGWYDWQQVPFCSNHFTELIMKDGRTLVVPCRGQDELVGRARISPGAGWNVRDTACLAKSYAQMWLLLYFHRRDLRLMANAICSAVPVNWVPTGRTTWSIHAGGEWMTTEDMLEVWNRVWIEENEWMEDKTPVEKWSDVPYSGKREDIWCGSLIGTRARATWAENIQVAINQVRAIIGDEKYVDYMSSLKRYEDTTLVEDTVL

>ADU76269.1 polyprotein [West Nile virus]

MSKKPGGPGKSRAVNMLKRGMPRVLSLIGLKRAMLSLIDGKGPIRFVLALLAFFRFTAIAPTRAVLDRWRGVNKQTAMKHLLSFKKELGTLTSAINRRSSKQKKRGGKTGIAVMIGLIASVGAVTLSNFQGKVMMTVNATDVTDVITIPTAAGKNLCIVRAMDVGYMCDDTITYECPVLSAGNDPEDIDCWCTKSAVYVRYGRCTKTRHSRRSRRSLTVQTHGESTLANKKGAWMDSTKATRYLVKTESWILRNPGYALVAAVIGWMLGSNTMQRVVFVVLLLLVAPAYSFNCLGMSNRDFLEGVSGATWVDLVLEGDSCVTIMSKDKPTIDVKMMNMEAANLAEVRSYCYLATVSDLSTKAACPTMGEAHNDKRADPAFVCRQGVVDRGWGNGCGLFGKGSIDTCAKFACSTKAIGRTILKENIKYEVAIFVHGPTTVESHGNYSTQAGATQAGRFSITPAAPSYTLKLGEYGEVTVDCEPRSGIDTNAYYVMTVGTKTFLVHREWFMDLNLPWSSAGSTVWRNRETLMEFEEPHATKQSVIALGSQEGALHQALAGAIPVEFSSNTVKLTSGHLKCRVKMEKLQLKGTTYGVCSKAFKFLGTPADTGHGTVVLELQYTGTDGPCKVPISSVASLNDLTPVGRLVTVNPFVSVATANAKVLIELEPPFGDSYIVVGRGEQQINHHWHKSGSSIGKAFTTTLKGAQRLAALGDTAWDFGSVGGVFTSVGKAVHQVFGGAFRSLFGGMSWITQGLLGALLLWMGINARDRSIALTFLAVGGVLLFLSVNVHADTGCAIDISRQELRCGSGVFIHNDVEAWMDRYKYYPETPQGLAKIIQKAHKEGVCGLRSVSRLEHQMWEAVKDELNTLLKENGVDLSVVVEKQEGMYKSAPKRLTATTEKLEIGWKAWGKSILFAPELANNTFVVDGPETKECPTQNRAWNSLEVEDFGFGLTSTRMFLKVRESNTTECDSKIIGTAVKNNLAIHSDLSYWIESRLNDTWKLERAVLGEVKSCTWPETHTLWGDGILESDLIIPVTLAGPRSNHNRRPGYKTQNQGPWDEGRVEIDFDYCPGTTVTLSESCGHRGPATRTTTESGKLITDWCCRSCTLPPLRYQTDSGCWYGMEIRPQRHDEKTLVQSQVNAYNADMIDPFQLGLLVVFLATQEVLRKRWTAKISMPAILIALLVLVFGGITYTDVLRYVILVGAAFAESNSGGDVVHLALMATFKIQPVFMVASFLKARWTNQENILLMLAAVFFQMAYHDARQILLWDIPDVLNSLAVAWMILRAITFTTTSNVVVPLLALLTPGLRCLNLDVYRILLLMVGIGSLIREKRSAAAKKKGASLLCLALASTGLFNPMILAAGLIACDPNRKRGWPATEVMTAVGLMFAIVGGLAELDIDSMAIPMTIAGLMFAAFVISGKSTDMWIERTADISWESDAEITGSSERVDVRLDDDGNFQLMNDPGAPWKIWMLRMVCLAISAYTPWAILPSVVGFWITLQYTKRGGVLWDTPSPKEYKKGDTTTGVYRIMTRGLLGSYQAGAGVMVEGVFHTLWHTTKGAALMSGEGRLDPYWGSVKEDRLCYGGPWKLQHKWNGQDEVQMIVVEPGKNVKNVQTKPGVFKTPEGEIGAVTLDFPTGTSGSPIVDKNGDVIGLYGNGVIMPNGSYISAIVQGERMDEPIPAGFEPEMLRKKQITVLDLHPGAGKTRRILPQIIKEAINRRLRTAVLAPTRVVAAEMAEALRGLPIRYQTSAVPREHNGNEIVDVMCHATLTHRLMSPHRVPNYNLFVMDEAHFTDPASIAARGYISTKVELGEAAAIFMTATPPGTSDPFPESNSPISDLQTEIPDRAWNSGYEWITEYTGKTVWFVPSVKMGNEIALCLQRAGKKVVQLNRKSYETEYPKCKNDDWDFVITTDISEMGANFKASRVIDSRKSVKPTIITEGEGRVILGEPSAVTAASAAQRRGRIGRNPSQVGDEYCYGGHTNEDDSNFAHWTEARIMLDNINMPNGLIAQFYQPEREKVYTMDGEYRLRGEERKNFLELLRTADLPVWLAYKVAAAGVSYHDRRWCFDGPRTNTILEDNNEVEVITKLGERKILRPRWIDARVYSDHQALKAFKDFASGKRSQIGLIEVLGKMPEHFMGKTWEALDTMYVVATAEKGGRAHRMALEELPDALQTIALIALLSVMTMGVFFLLMQRKGIGKIGLGGAVLGVATFFCWMAEVPGTKIAGMLLLSLLLMIVLIPEPEKQRSQTDNQLAVFLICVMTLVSAVAANEMGWLDKTKSDISSLFGQRIEVKENFSMGEFLLDLRPATAWSLYAVTTAVLTPLLKHLITSDYINTSLTSINVQASALFTLARGFPFVDVGVSALLLAAGCWGQVTLTVTVTAATLLFCHYAYMVPGWQAEAMRSAQRRTAAGIMKNAVVDGIVATDVPELERTTPIMQKKVGQIMLILVSLAAVVVNPSVKTVREAGILITAAAVTLWENGASSVWNATTAIGLCHIMRGGWLSCLSITWTLIKNMEKPGLKRGGAKGRTLGEVWKERLNQMTKEEFTRYRKEAIIEVDRSAAKHARKEGNVTGGHPVSRGTAKLRWLVERRFLEPVGKVIDLGCGRGGWCYYMATQKRVQEVRGYTKGGPGHEEPQLVQSYGWNIVTMKSGVDVFYRPSECCDTLLCDIGESSSSAEVEEHRTIRVLEMVEDWLHRGPREFCVKVLCPYMPKVIEKMELLQRRYGGGLVRNPLSRNSTHEMYWVSRASGNVVHSVNMTSQVLLGRMEKRTWKGPQYEEDVNLGSGTRAVGKPLLNSDTSKIKNRIERLRREYSSTWHHDENHPYRTWNYHGSYDVKPTGSASSLVNGVVRLLSKPWDTITNVTTMAMTDTTPFGQQRVFKEKVDTKAPEPPEGVKYVLNETTNWLWAFLAREKRPRMCSREEFIRKVNSNAALGAMFEEQNQWRSAREAVEDPKFWEMVDEEREAHLRGECHTCIYNMMGKREKKPGEFGKAKGSRAIWFMWLGARFLEFEALGFLNEDHWLGRKNSGGGVEGLGLQKLGYILREVGTRPGGKIYADDTAGWDTRITRADLENEAKVLELLDGEHRRLARAIIELTYRHKVVKVMRPAADGRTVMDVISREDQRGSGQVVTYALNTFTNLAVQLVRMMEGEGVIGPDDVEKLTKGKGPKVRTWLFENGEERLSRMAVSGDDCVVKPLDDRFATSLHFLNAMSKVRKDIQEWKPSTGWYDWQQVPFCSNHFTELIMKDGRTLVVPCRGQDELVGRARISPGAGWNVRDTACLAKSYAQMWLLLYFHRRDLRLMANAICSAVPVNWVPTGRTTWSIHAGGEWMTTEDMLEVWNRVWIEENEWMEDKTPVEKWSDVPYSGKREDIWCGSLIGTRARATWAENIQVAINQVRAIIGDEKYVDYMSSLKRYEDTTLVEDTVL

>ADL27983.1 polyprotein [West Nile virus]

MSKKPGGPGKSRAVNMLKRGMPRVLSLIGLKRAMLSLIDGKGPIRFVLALLAFFRFTAIAPTRAVLDRWRGVNKQTAMKHLLSFKKELGTLTSAINRRSSKQKKRGGKTGIAVMIGLIASVGAVTLSNFQGKVMMTVNATDVTDVITIPTAAGKNLCIVRAMDVGYMCDDTITYECPVLSAGNDPEDIDCWCTKSAVYVRYGRCTKTRHSRRSRRSLTVQTHGESTLANKKGAWMDSTKATRYLVKTESWILRNPGYALVAAVIGWMLGSNTMQRVVFVVLLLLVAPAYSFNCLGMSNRDFLEGVSGATWVDLVLEGDSCVTIMSKDKPTIDVKMMNMEAANLAEVRSYCYLATVSDLSTKAACPTMGEAHNDKRADPAFVCRQGVVDRGWGNGCGLFGKGSIDTCAKFACSTKAIGRTILKENIKYEVAIFVHGPTTVESHGNYSTQAGATQAGRFSITPAAPSYTLKLGEYGEVTVDCEPRSGIDTNAYYVMTVGTKTFLVHREWFMDLNLPWSSAGSTVWRNRETLMEFEEPHATKQSVIALGSQEGALHQALAGAIPVEFSSNTVKLTSGHLKCRVKMEKLQLKGTTYGVCSKAFKFLGTPADTGHGTVVLELQYTGTDGPCKVPISSVASLNDLTPVGRLVTVNPFVSVATANAKVLIELEPPFGDSYIVVGRGEQQINHHWHKSGSSIGKAFTTTLKGAQRLAALGDTAWDFGSVGGVFTSVGKAVHQVFGGAFRSLFGGMSWITQGLLGALLLWMGINARDRSIALTFLAVGGVLLFLSVNVHADTGCAIDISRQELRCGSGVFIHNDVEAWMDRYKYYPETPQGLAKIIQKAHKEGVCGLRSVSRLEHQMWEAVKDELNTLLKENGVDLSVVVEKQEGMYKSAPKRLTATTEKLEIGWKAWGKSILFAPELANNTFVVDGPETKECPTQNRAWNSLEVEDFGFGLTSTRMFLKVRESNTTECDSKIIGTAVKNNLAIHSDLSYWIESRLNDTWKLERAVLGEVKSCTWPETHTLWGDGILESDLIIPVTLAGPRSNHNRRPGYKTQNQGPWDEGRVEIDFDYCPGTTVTLSESCGHRGPATRTTTESGKLITDWCCRSCTLPPLRYQTDSGCWYGMEIRPQRHDEKTLVQSQVNAYNADMIDPFQLGLLVVFLATQEVLRKRWTAKISMPAILIALLVLVFGGITYTDVLRYVILVGAAFAESNSGGDVVHLALMATFKIQPVFMVASFLKARWTNQENILLMLAAVFFQMAYHDARQILLWEIPDVLNSLAVAWMILRAITFTTTSNVVVPLLALLTPGLRCLNLDVYRILLLMVGIGSLIREKRSAAAKKKGASLLCLALASTGLFNPMILAAGLIACDPNRKRGWPATEVMTAVGLMFAIVGGLAELDIDSMAIPMTIAGLMFAAFVISGKSTDMWIERTADISWESDAEITGSSERVDVRLDDDGNFQLMNDPGAPWKIWMLRMVCLAISAYTPWAILPSVVGFWITLQYTKRGGVLWDTPSPKEYKKGDTTTGVYRIMTRGLLGSYQAGAGVMVEGVFHTLWHTTKGAALMSGEGRLDPYWGSVKEDRLCYGGPWKLQHKWNGQDEVQMIVVEPGKNVKNVQTKPGVFKTPEGEIGAVTLDFPTGTSGSPIVDKNGDVIGLYGNGVIMPNGSYISAIVQGERMDEPIPAGFEPEMLRKKQITVLDLHPGAGKTRRILPQIIKEAINRRLRTAVLAPTRVVAAEMAEALRGLPIRYQTSAVPREHNGNEIVDVMCHATLTHRLMSPHRVPNYNLFVMDEAHFTDPASIAARGYISTKVELGEAAAIFMTATPPGTSDPFPESNSPISDLQTEIPDRAWNSGYEWITEYTGKTVWFVPSVKMGNEIALCLQRAGKKVVQLNRKSYETEYPKCKNDDWDFVITTDISEMGANFKASRVIDSRKSVKPTIITEGEGRVILGEPSAVTAASAAQRRGRIGRNPSQVGDEYCYGGHTNEDDSNFAHWTEARIMLDNINMPNGLIAQFYQPEREKVYTMDGEYRLRGEERKNFLELLRTADLPVWLAYKVAAAGVSYHDRRWCFDGPRTNTILEDNNEVEVITKLGERKILRPRWIDARVYSDHQALKAFKDFASGKRSQIGLIEVLGKMPEHFMGKTWQALDTMYVVATAEKGGRAHRMALEELPDALQTIALIALLSVMTMGVFFLLMQRKGIGKIGLGGAVLGVATFFCWMAEVPGTKIAGMLLLSLLLMIVLIPEPEKQRSQTDNQLAVFLICVMTLVSAVAANEMGWLDKTKSDISSLFGQRIEVKENFSMGEFLLDLRPATAWSLYAVTTAVLTPLLKHLITSDYINTSLTSINVQASALFTLARGFPFVDVGVSALLLAAGCWGQVTLTVTVTAATLLFCHYAYMVPGWQAEAMRSAQRRTAAGIMKNAVVDGIVATDVPELERTTPIMQKKVGQIMLILVSLAAVVVNPSVKTVREAGILITAAAVTLWENGASSVWNATTAIGLCHIMRGGWLSCLSITWTLIKNMEKPGLKRGGAKGRTLGEVWKERLNQMTKEEFTRYRKEAIIEVDRSAAKHARKEGNVTGGHPVSRGTAKLRWLVERRFLEPVGKVIDLGCGRGGWCYYMATQKRVQEVRGYTKGGPGHEEPQLVQSYGWNIVTMKSGVDVFYRPSECCDTLLCDIGESSSSAEVEEHRTIRVLEMVEDWLHRGPREFCVKVLCPYMPKVIEKMELLQRRYGGGLVRNPLSRNSTHEMYWVSRASGNVVHSVNMTSQVLLGRMEKRTWKGPQYEEDVNLGSGTRAVGKPLLNSDTSKIKNRIERLRREYSSTWHHDENHPYRTWNYHGSYDVKPTGSASSLVNGVVRLLSKPWDTITNVTTMAMTDTTPFGQQRVFKEKVDTKAPEPPEGVKYVLNETTNWLWAFLAREKRPRMCSREEFIRKVNSNAALGAMFEEQNQWRSAREAVEDPKFWEMVDEEREAHLRGECHTCIYNMMGKREKKPGEFGKAKGSRAIWFMWLGARFLEFEALGFLNEDHWLGRKNSGGGVEGLGLQKLGYILREVGTRPGGKIYADDTAGWDTRITRADLENEAKVLELLDGEHRRLARAIIELTYRHKVVKVMRPAADGRTVMDVISREDQRGSGQVVTYALNTFTNLAVQLVRMMEGEGVIGPDDVEKLTKGKGPKVRTWLFENGEERLSRMAVSGDDCVVKPLDDRFATSLHFLNAMSKVRKDIQEWKPSTGWYDWQQVPFCSNHFTELIMKDGRTLVVPCRGQDELVGRARISPGAGWNVRDTACLAKSYAQMWLLLYFHRRDLRLMANAICSAVPVNWVPTGRTTWSIHAGGEWMTTEDMLEVWNRVWIEENEWMEDKTPVEKWSDVPYSGKREDIWCGSLIGTRARATWAENIQVAINQVRAIIGDEKYVDYMSSLKRYEDTTLVEDTVL

>ANW69703.1 genome polyprotein [West Nile virus]

MSKKPGGPGKSRAVNMLKRGMPRVLSLIGLKRAMLSLIDGKGPIRFVLALLAFFRFTAIAPTRAVLDRWRGVNKQTAMKHLLSFKKELGTLTSAINRRSSKQKKRGGKTGIAVMIGLIASVGAVTLSNFQGKVMMTVNATDVTDVITIPTAAGKNLCIVRAMDVGYMCDDTITYECPVLSAGNDPEDIDCWCTKSAVYVRYGRCTKTRHSRRSRRSLTVQTHGESTLANKKGAWMDSTKATRYLVKTESWILRNPGYALVAAVIGWMLGSNTMQRVVFVVLLLLVAPAYSFNCLGMSNRDFLEGVSGATWVDLVLEGDSCVTIMSKDKPTIDVKMMNMEAANLAEVRSYCYLATVSDLSTKAACPTMGEAHNDKRADPAFVCRQGVVDRGWGNGCGLFGKGSIDTCAKFACSTKAIGRTILKENIKYEVAIFVHGPTTVESHGNYSTQAGATQAGRFSITPAAPSYTLKLGEYGEVTVDCEPRSGIDTNAYYVMTVGTKTFLVHREWFMDLNLPWSSAGSTVWRNRETLMEFEEPHATKQSVIALGSQEGALHQALAGAIPVEFSSNTVKLTSGHLKCRVKMEKLQLKGTTYGVCSKAFKFLGTPADTGHGTVVLELQYTGTDGPCKVPISSVASLNDLTPVGRLVTVNPFVSVATANAKVLIELEPPFGDSYIVVGRGEQQINHHWHKSGSSIGKAFTTTLKGAQRLAALGDTAWDFGSVGGVFTSVGKAVHQVFGGAFRSLFGGMSWITQGLLGALLLWMGINARDRSIALTFLAVGGVLLFLSVNVHADTGCAIDISRQELRCGSGVFIHNDVEAWMDRYKYYPETPQGLAKIIQKAHKEGVCGLRSVSRLEHQMWEAVKDELNTLLKENGVDLSVVVEKQEGMYKSAPKRLTATTEKLEIGWKAWGKSILFAPELANNTFVVDGPETKECPTQNRAWNSLEVEDFGFGLTSTRMFLKVRESNTTECDSKIIGTAVKNNLAIHSDLSYWIESRLNDTWKLERAVLGEVKSCTWPETHTLWGDGILESDLIIPVTLAGPRSNHNRRPGYKTQNQGPWDEGRVEIDFDYCPGTTVTLSESCGHRGPATRTTTESGKLITDWCCRSCTLPPLRYQTDSGCWYGMEIRPQRHDEKTLVQSQVNAYNADMIDPFQLGLLVVFLATQEVLRKRWTAKISMPAILIALLVLVFGGITYTDVLRYVILVGAAFAESNSGGDVVHLALMATFKIQPVFMVASFLKARWTNQENILLMLAAVFFQMAYHDARQILLWEIPDVLNSLAVAWMILRAITFTTTSNVVVPLLALLTPGLRCLNLDVYRILLLMVGIGSLIREKRSAAAKKKGASLLCLALASTGLFNPMILAAGLIACDPNRKRGWPATEVMTAVGLMFAIVGGLAELDIDSMAIPMTIAGLMFAAFVISGKSTDMWIERTADISWESDAEITGSSERVDVRLDDDGNFQLMNDPGAPWKIWMLRMVCLAISAYTPWAILPSVVGFWITLQYTKRGGVLWDTPSPKEYKKGDTTTGVYRIMTRGLLGSYQAGAGVMVEGVFHTLWHTTKGAALMSGEGRLDPYWGSVKEDRLCYGGPWKLQHKWNGQDEVQMIVVEPGKNVKNVQTKPGVFKTPEGEIGAVTLDFPTGTSGSPIVDKNGDVIGLYGNGVIMPNGSYISAIVQGERMDEPIPAGFEPEMLRKKQITVLDLHPGAGKTRRILPQIIKEAINRRLRTAVLAPTRVVAAEMAEALRGLPIRYQTSAVPREHNGNEIVDVMCHATLTHRLMSPHRVPNYNLFVMDEAHFTDPASIAARGYISTKVELGEAAAIFMTATPPGTSDPFPESNSPISDLQTEIPDRAWNSGYEWITEYTGKTVWFVPSVKMGNEIALCLQRAGKKVVQLNRKSYETEYPKCKNDDWDFVITTDISEMGANFKASRVIDSRKSVKPTIITEGEGRVILGEPSAVTAASAAQRRGRIGRNPSQVGDEYCYGGHTNEDDSNFAHWTEARIMLDNINMPNGLIAQFYQPEREKVYTMDGEYRLRGEERKNFLELLRTADLPVWLAYKVAAAGVSYHDRRWCFDGPRTNTILEDNNEVEVITKLGERKILRPRWIDARVYSDHQALKAFKDFASGKRSQIGLIEVLGKMPEHFMGKTWEALDTMYVVATADKGGRAHRMALEELPDALQTIALIALLSVMTMGVFFLLMQRKGIGKIGLGGAVLGVATFFCWMAEVPGTKIAGMLLLSLLLMIVLIPEPEKQRSQTDNQLAVFLICVMTLVSAVAANEMGWLDKTKSDISSLFGQRIEVKENFSMGEFLLDLRPATAWSLYAVTTAVLTPLLKHLITSDYINTSLTSINVQASALFTLARGFPFVDVGVSALLLAAGCWGQVTLTVTVTAATLLFCHYAYMVPGWQAEAMRSAQRRTAAGIMKNAVVDGIVATDVPELERTTPIMQKKVGQIMLILVSLAAVVVNPSVKTVREAGILITAAAVTLWENGASSVWNATTAIGLCHIMRGGWLSCLSITWTLIKNMEKPGLKRGGAKGRTLGEVWKERLNQMTKEEFTRYRKEAIIEVDRSAAKHARKEGNVTGGHPVSRGTAKLRWLVERRFLEPVGKVIDLGCGRGGWCYYMATQKRVQEVRGYTKGGPGHEEPQLVQSYGWNIVTMKSGVDVFYRPSECCDTLLCDIGESSSSAEVEEHRTIRVLEMVEDWLHRGPREFCVKVLCPYMPKVIEKMELLQRRYGGGLVRNPLSRNSTHEMYWVSRASGNVVHSVNMTSQVLLGRMEKRTWKGPQYEEDVNLGSGTRAVGKPLLNSDTSKIKNRIERLRREYSSTWHHDENHPYRTWNYHGSYDVKPTGSASSLVNGVVRLLSKPWDTITNVTTMAMTDTTPFGQQRVFKEKVDTKAPEPPEGVKYVLNETTNWLWAFLAREKRPRMCSREEFIRKVNSNAALGAMFEEQNQWRSAREAVEDPKFWEMVDEEREAHLRGECHTCIYNMMGKREKKPGEFGKAKGSRAIWFMWLGARFLEFEALGFLNEDHWLGRKNSGGGVEGLGLQKLGYILREVGTRPGGKIYADDTAGWDTRITRADLENEAKVLELLDGEHRRLARAIIELTYRHKVVKVMRPAADGRTVMDVISREDQRGSGQVVTYALNTFTNLAVQLVRMMEGEGVIGPDDVEKLTKGKGPKVRTWLFENGEERLSRMAVSGDDCVVKPLDDRFATSLHFLNAMSKVRKDIQEWKPSTGWYDWQQVPFCSNHFTELIMKDGRTLVVPCRGQDELVGRARISPGAGWNVRDTACLAKSYAQMWLLLYFHRRDLRLMANAICSAVPVNWVPTGRTTWSIHAGGEWMTTEDMLEVWNRVWIEENEWMEDKTPVEKWSDVPYSGKREDIWCGSLIGTRARATWAENIQVAINQVRAIIGDEKYVDYMSSLKRYEDTTLVEDTVL

>AYV87348.1 polyprotein [West Nile virus]

MSKKPGGPGKSRAVNMLKRGMPRVLSLIGLKRAMLSLIDGKGPIRFVLALLAFFRFTAIAPTRAVLDRWRGVNKQTAMKHLLSFKKELGTLTSAINRRSSKQKKRGGKTGIAVMIGLIASVGAVTLSNFQGKVMMTVNATDVTDVITIPTAAGKNLCIVRAMDVGYMCDDTITYECPVLSAGNDPEDIDCWCTKSAVYVRYGRCTKTRHSRRSRRSLTVQTHGESTLANKKGAWMDSTKATRYLVKTESWILRNPGYALVAAVIGWMLGSNTMQRVVFVVLLLLVAPAYSFNCLGMSNRDFLEGVSGATWVDLVLEGDSCVTIMSKDKPTIDVKMMNMEAANLAEVRSYCYLATVSDLSTKAACPTMGEAHNDKRADPAFVCRQGVVDRGWGNGCGLFGKGSIDTCAKFACSTKAIGRTILKENIKYEVAIFVHGPTTVESHGNYSTQAGATQAGRFSITPAAPSYTLKLGEYGEVTVDCEPRSGIDTNAYYVMTVGTKTFLVHREWFMDLNLPWSSAGSTVWRNRETLMEFEEPHATKQSVIALGSQEGALHQALAGAIPVEFSSNTVKLTSGHLKCRVKMEKLQLKGTTYGVCSKAFKFLGTPADTGHGTVVLELQYTGTDGPCKVPISSVASLNDLTPVGRLVTVNPFVSVATANAKVLIELEPPFGDSYIVVGRGEQQINHHWHKSGSSIGKAFTTTLKGAQRLAALGDTAWDFGSVGGVFTSVGKAVHQVFGGAFRSLFGGMSWITQGLLGALLLWMGINARDRSIALTFLAVGGVLLFLSVNVHADTGCAIDISRQELRCGSGVFIHNDVEAWMDRYKYYPETPQGLAKIIQKAHKEGVCGLRSVSRLEHQMWEAVKDELNTLLKENGVDLSVVVEKQEGMYKSAPKRLTATTEKLEIGWKAWGKSILFAPELANNTFVVDGPETKECPTQNRAWNSLEVEDFGFGLTSTRMFLKVRESNTTECDSKIIGTAVKNNLAIHSDLSYWIESRLNDTWKLERAVLGEVKSCTWPETHTLWGDGILESDLIIPVTLAGPRSNHNRRPGYKTQNQGPWDEGRVEIDFDYCPGTTVTLSESCGHRGPATRTTTESGKLITDWCCRSCTLPPLRYQTDSGCWYGMEIRPQRHDEKTLVQSQVNAYNADMIDPFQLGLLVVFLATQEVLRKRWTAKISMPAILIALLVLVFGGITYTDVLRYVILVGAAFAESNSGGDVVHLALMATFKIQPVFMVASFLKARWTNQENILLMLAAVFFQMAYHDARQILLWEIPDVLNSLAVAWMILRAITFTTTSNVVVPLLALLTPGLRCLNLDVYRILLLMVGIGSLIREKRSAAAKKKGASLLCLALASTGLFNPMILAAGLIACDPNRKRGWPATEVMTAVGLMFAIVGGLAELDIDSMAIPMTIAGLMFAAFVISGKSTDMWIERTADISWESDAEITGSSERVDVRLDDDGNFQLMNDPGAPWKIWMLRMVCLAISAYTPWAILPSVVGFWITLQYTKRGGVLWDTPSPKEYKKGDTTTGVYRIMTRGLLGSYQAGAGVMVEGVFHTLWHTTKGAALMSGEGRLDPYWGSVKEDRLCYGGPWKLQHKWNGQDEVQMIVVEPGKNVKNVQTKPGVFKTPEGEIGAVTLDFPTGTSGSPIVDKNGDVIGLYGNGVIMPNGSYMSAIVQGERMDEPIPAGFEPEMLRKKQITVLDLHPGAGKTRRILPQIIKEAINRRLRTAVLAPTRVVAAEMAEALRGLPIRYQTSAVPREHNGNEIVDVMCHATLTHRLMSPHRVPNYNLFVMDEAHFTDPASIAARGYISTKVELGEAAAIFMTATPPGTSDPFPESNSPISDLQTEIPDRAWNSGYEWITEYTGKTVWFVPSVKMGNEIALCLQRAGKKVVQLNRKSYETEYPKCKNDDWDFVITTDISEMGANFKASRVIDSRKSVKPTIITEGEGRVILGEPSAVTAASAAQRRGRIGRNPSQVGDEYCYGGHTNEDDSNFAHWTEARIMLDNINMPNGLIAQFYQPEREKVYTMDGEYRLRGEERKNFLELLRTADLPVWLAYKVAAAGVSYHDRRWCFDGPRTNTILEDNNEVEVITKLGERKILRPRWIDARVYSDHQALKAFKDFASGKRSQIGLIEVLGKMPEHFMGKTWEALDTMYVVATAEKGGRAHRMALEELPDALQTIALIALLSVMTMGVFFLLMQRKGIGKIGLGGAVLGVATFFCWMAEVPGTKIAGMLLLSLLLMIVLIPEPEKQRSQTDNQLAVFLICVMTLVSAVAANEMGWLDKTKSDISSLFGQRIEVKENFSMGEFLLDLRPATAWSLYAVTTAVLTPLLKHLITSDYINTSLTSINVQASALFTLARGFPFVDVGVSALLLAAGCWGQVTLTVTVTAATLLFCHYAYMVPGWQAEAMRSAQRRTAAGIMKNAVVDGIVATDVPELERTTPIMQKKVGQIMLILVSLAAVVVNPSVKTVREAGILITAAAVTLWENGASSVWNATTAIGLCHIMRGGWLSCLSITWTLIKNMEKPGLKRGGAKGRTLGEVWKERLNQMTKEEFTRYRKEAIIEVDRSAAKHARKEGNVTGGHPVSRGTAKLRWLVERRFLEPVGKVIDLGCGRGGWCYYMATQKRVQEVRGYTKGGPGHEEPQLVQSYGWNIVTMKSGVDVFYRPSECCDTLLCDIGESSSSAEVEEHRTIRVLEMVEDWLHRGPREFCVKVLCPYMPKVIEKMELLQRRYGGGLVRNPLSRNSTHEMYWVSRASGNVVHSVNMTSQVLLGRMEKRTWKGPQYEEDVNLGSGTRAVGKPLLNSDTSKIKNRIERLRREYSSTWHHDENHPYRTWNYHGSYDVKPTGSASSLVNGVVRLLSKPWDTITNVTTMAMTDTTPFGQQRVFKEKVDTKAPEPPEGVKYVLNETTNWLWAFLAREKRPRMCSREEFIRKVNSNAALGAMFEEQNQWRSAREAVEDPKFWEMVDEEREAHLRGECHTCIYNMMGKREKKPGEFGKAKGSRAIWFMWLGARFLEFEALGFLNEDHWLGRKNSGGGVEGLGLQKLGYILREVGTRPGGKIYADDTAGWDTRITRADLENEAKVLELLDGEHRRLARAIIELTYRHKVVKVMRPAADGRTVMDVISREDQRGSGQVVTYALNTFTNLAVQLVRMMEGEGVIGPDDVEKLTKGKGPKVRTWLFENGEERLSRMAVSGDDCVVKPLDDRFATSLHFLNAMSKVRKDIQEWKPSTGWYDWQQVPFCSNHFTELIMKDGRTLVVPCRGQDELVGRARISPGAGWNVRDTACLAKSYAQMWLLLYFHRRDLRLMANAICSAVPVNWVPTGRTTWSIHAGGEWMTTEDMLEVWNRVWIEENEWMEDKTPVEKWSDVPYSGKREDIWCGSLIGTRARATWAENIQVAINQVRAIIGDEKYVDYMSSLKRYEDTTLVEDTVL

>AHW48745.1 polyprotein [West Nile virus]

MSKKPGGPGKSRAVNMLKRGMPRVLSLIGLKRAMLSLIDGKGPIRFVLALLAFFRFTAIAPTRAVLDRWRGVNKQTAMKHLLSFKKELGTLTSAINRRSSKQKKRGGKTGIAVMIGLIASVGAVTLSNFQGKVMMTVNATDVTDVITIPTAAGKNLCIVRAMDVGYMCDDTITYECPVLSAGNDPEDIDCWCTKSAVYVRYGRCTKTRHSRRSRRSLTVQTHGESTLANKKGAWMDSTKATRYLVKTESWILRNPGYALVAAVIGWMLGSNTMQRVVFVVLLLLVAPAYSFNCLGMSNRDFLEGVSGATWVDLVLEGDSCVTIMSKDKPTIDVKMMNMEAANLAEVRSYCYLATVSDLSTKAACPTMGEAHNDKRADPAFVCRQGVVDRGWGNGCGLFGKGSIDTCAKFACSTKAIGRTILKENIKYEVAIFVHGPTTVESHGNYSTQAGATQAGRFSITPAAPSYTLKLGEYGEVTVDCEPRSGIDTNAYYVMTVGTKTFLVHREWFMDLNLPWSSAGSTVWRNRETLMEFEEPHATKQSVIALGSQEGALHQALAGAIPVEFSSNTVKLTSGHLKCRVKMEKLQLKGTTYGVCSKAFKFLGTPADTGHGTVVLELQYTGTDGPCKVPISSVASLNDLTPVGRLVTVNPFVSVATANAKVLIELEPPFGDSYIVVGRGEQQINHHWHKSGSSIGKAFTTTLKGAQRLAALGDTAWDFGSVGGVFTSVGKAVHQVFGGAFRSLFGGMSWITQGLLGALLLWMGINARDRSIALTFLAVGGVLLFLSVNVHADTGCAIDISRQELRCGSGVFIHNDVEAWMDRYKYYPETPQGLAKIIQKAHKEGVCGLRSVSRLEHQMWEAVKDELNTLLKENGVDLSVVVEKQEGMYKSAPKRLTATTEKLEIGWKAWGKSILFAPELANNTFVVDGPETKECPTQNRAWNSLEVEDFGFGLTSTRMFLKVRESNTTECDSKIIGTAVKNNLAIHSDLSYWIESRLNDTWKLERAVLGEVKSCTWPETHTLWGDGILESDLIIPVTLAGPRSNHNRRPGYKTQNQGPWDEGRVEIDFDYCPGTTVTLSESCGHRGPATRTTTESGKLITDWCCRSCTLPPLRYQTDSGCWYGMEIRPQRHDEKTLVQSQVNAYNADMIDPFQLGLLVVFLATQEVLRKRWTAKISMPAILIALLVLVFGGITYTDVLRYVILVGAAFAESNSGGDVVHLALMATFKIQPVFMVASFLKARWTNQENILLMLAAVFFQMAYHDARQILLWEIPDVLNSLAVAWMILRAITFTTTSNVVVPLLALLTPGLRCLNLDVYRILLLMVGIGSLIREKRSAAAKKKGASLLCLALASTGLFNPMILAAGLIACDPNRKRGWPATEVMTAVGLMFAIVGGLAELDIDSMAIPMTIAGLMFAAFVISGKSTDMWIERTADISWESDAEITGSSERVDVRLDDDGNFQLMNDPGAPWKIWMLRMVCLAISAYTPWAILPSVVGFWITLQYTKRGGVLWDTPSPKEYKKGDTTTGVYRIMTRGLLGSYQAGAGVMVEGVFHTLWHTTKGAALMSGEGRLDPYWGSVKEDRLCYGGPWKLQHKWNGQDEVQMIVVEPGKNVKNVQTKPGVFKTPEGEIGAVTLDFPTGTSGSPIVDKNGDVIGLYGNGVIMPNGSYISAIVQGERMDEPIPAGFEPEMLRKKQITVLDLHPGAGKTRRILPQIIKEAINRRLRTAVLAPTRVVAAEMAEALRGLPIRYQTSAVPREHNGNEIVDVMCHATLTHRLMSPHRVPNYNLFVMDEAHFTDPASIAARGYISTKVELGEAAAIFMTATPPGTSDPFPESNSPISDLQTEIPDRAWNSGYEWITEYTGKTVWFVPSVKMGNEIALCLQRAGKKVVQLNRKSYETEYPKCKNDDWDFVITTDISEMGANFKASRVIDSRKSVKPTIITEGEGRVILGEPSAVTAASAAQRRGRIGRNPSQVGDEYCYGGHTNEDDSNFAHWTEARIMLDNINMPNGLIAQFYQPEREKVYTMDGEYRLRGEERKNFLELLRTADLPVWLAYKVAAAGVSYHDRRWCFDGPRTNTILEDNNEVEVITKLGERKILRPRWIDARVYSDHQALKAFKDFASGKRSQIGLIEVLGKMPEHFMGKTWEALDTMYVVATAEKGGRAHRMALEELPDALQTITLIALLSVMTMGVFFLLMQRKGIGKIGLGGAVLGVATFFCWMAEVPGTKIAGMLLLSLLLMIVLIPEPEKQRSQTDNQLAVFLICVMTLVSAVAANEMGWLDKTKSDISSLFGQRIEVKENFSMGEFLLDLRPATAWSLYAVTTAVLTPLLKHLITSDYINTSLTSINVQASALFTLARGFPFVDVGVSALLLAAGCWGQVTLTVTVTAATLLFCHYAYMVPGWQAEAMRSAQRRTAAGIMKNAVVDGIVATDVPELERTTPIMQKKVGQIMLILVSLAAVVVNPSVKTVREAGILITAAAVTLWENGASSVWNATTAIGLCHIMRGGWLSCLSITWTLIKNMEKPGLKRGGAKGRTLGEVWKERLNQMTKEEFTRYRKEAIIEVDRSAAKHARKEGNVTGGHPVSRGTAKLRWLVERRFLEPVGKVIDLGCGRGGWCYYMATQKRVQEVRGYTKGGPGHEEPQLVQSYGWNIVTMKSGVDVFYRPSECCDTLLCDIGESSSSAEVEEHRTIRVLEMVEDWLHRGPREFCVKVLCPYMPKVIEKMELLQRRYGGGLVRNPLSRNSTHEMYWVSRASGNVVHSVNMTSQVLLGRMEKRTWKGPQYEEDVNLGSGTRAVGKPLLNSDTSKIKNRIERLRREYSSTWHHDENHPYRTWNYHGSYDVKPTGSASSLVNGVVRLLSKPWDTITNVTTMAMTDTTPFGQQRVFKEKVDTKAPEPPEGVKYVLNETTNWLWAFLAREKRPRMCSREEFIRKVNSNAALGAMFEEQNQWRSAREAVEDPKFWEMVDEEREAHLRGECHTCIYNMMGKREKKPGEFGKAKGSRAIWFMWLGARFLEFEALGFLNEDHWLGRKNSGGGVEGLGLQKLGYILREVGTRPGGKIYADDTAGWDTRITRADLENEAKVLELLDGEHRRLARAIIELTYRHKVVKVMRPAADGRTVMDVISREDQRGSGQVVTYALNTFTNLAVQLVRMMEGEGVIGPDDVEKLTKGKGPKVRTWLFENGEERLSRMAVSGDDCVVKPLDDRFATSLHFLNAMSKVRKDIQEWKPSTGWYDWQQVPFCSNHFTELIMKDGRTLVVPCRGQDELVGRARISPGAGWNVRDTACLAKSYAQMWLLLYFHRRDLRLMANAICSAVPVNWVPTGRTTWSIHAGGEWMTTEDMLEVWNRVWIEENEWMEDKTPVEKWSDVPYSGKREDIWCGSLIGTRARATWAENIQVAINQVRAIIGDEKYVDYMSSLKRYEDTTLVEDTVL

>AAZ32736.1 polyprotein precursor [West Nile virus]

MSKKPGGPGKSRAVNMLKRGMPRVLSLIGLKRAMLSLIDGKGPIRFVLALLAFFRFTAIAPTRAVLDRWRGVNKQTAMKHLLSFKKELGTLTSAINRRSSKQKKRGGKTGIAVMIGLIASVGAVTLSNFQGKVMMTVNATDVTDVITIPTAAGKNLCIVRAMDVGYMCDDTITYECPVLSAGNDPEDIDCWCTKSAVYVRYGRCTKTRHSRRSRRSLTVQTHGESTLANKKGAWMDSTKATRYLVKTESWILRNPGYALVAAVIGWMLGSNTMQRVVFVVLLLLVAPAYSFNCLGMSNRDFLEGVSGATWVDLVLEGDSCVTIMSKDKPTIDVKMMNMEAANLAEVRSYCYLATVSDLSTKAACPTMGEAHNDKRADPAFVCRQGVVDRGWGNGCGLFGKGSIDTCAKFACSTKAIGRTILKENIKYEVAIFVHGPTTVESHGNYSTQAGATQAGRFSITPAAPSYTLKLGEYGEVTVDCEPRSGIDTNAYYVMTVGTKTFLVHREWFMDLNLPWSSAGSTVWRNRETLMEFEEPHATKQSVIALGSQEGALHQALAGAIPVEFSSNTVKLTSGHLKCRVKMEKLQLKGTTYGVCSKAFKFLGTPADTGHGTVVLELQYTGTDGPCKVPISSVASLNDLTPVGRLVTVNPFVSVATANAKVLIELEPPFGDSYIVVGRGEQQINHHWHKSGSSIGKAFTTTLKGAQRLAALGDTAWDFGSVGGVFTSVGKAVHQVFGGAFRSLFGGMSWITQGLLGALLLWMGINARDRSIALTFLAVGGVLLFLSVNVHADTGCAIDISRQELRCGSGVFIHNDVEAWMDRYKYYPETPQGLAKIIQKAHKEGVCGLRSVSRLEHQMWEAVKDELNTLLKENGVDLSVVVEKQEGMYKSAPKRLTATTEKLEIGWKAWGKSILFAPELANNTFVVDGPETKECPTQNRAWNSLEVEDFGFGLTSTRMFLKVRESNTTECDSKIIGTAVKNNLAIHSDLSYWIESRLNDTWKLERAVLGEVKSCTWPETHTLWGDGILESDLIIPVTLAGPRSNHNRRPGYKTQNQGPWDEGRVEIDFDYCPGTTVTLSESCGHRGPATRTTTESGKLITDWCCRSCTLPPLRYQTDSGCWYGMEIRPQRHDEKTLVQSQVNAYNADMIDPFQLGLLVVFLATQEVLRKRWTAKISMPAILIALLVLVFGGITYTDVLRYVILVGAAFAESNSGGDVVHLALMATFKIQPVFMVASFLKARWTNQENILLMLAAVFFQMAYHDARQILLWEIPDVLNSLAVAWMILRAITFTTTSNVVVPLLALLTPGLRCLNLDVYRILLLMVGIGSLIREKRSAAAKKKGASLLCLALASTGLFNPMILAAGLIACDPNRKRGWPATEVMTAVGLMFAIVGGLAELDIDSMAIPMTIAGLMFAAFVISGKSTDMWIERTADISWESDAEITGSSERVDVRLDDDGNFQLMNDPGAPWKIWMLRMVCLAISAYTPWAILPSVVGFWITLQYTKRGGVLWDTPSPKEYEKGDTTTGVYRIMTRGLLGSYQAGAGVMVEGVFHTLWHTTKGAALMSGEGRLDPYWGSVKEDRLCYGGPWKLQHKWNGQDEVQMIVVEPGKNVKNVQTKPGVFKTPEGEIGAVTLDFPTGTSGSPIVDKNGDVIGLYGNGVIMPNGSYISAIVQGERMDEPIPAGFEPEMLRKKQITVLDLHPGAGKTRRILPQIIKEAINRRLRTAVLAPTRVVAAEMAEALRGLPIRYQTSAVPREHNGNEIVDVMCHATLTHRLMSPHRVPNYNLFVMDEAHFTDPASIAARGYISTKVELGEAAAIFMTATPPGTSDPFPESNSPISDLQTEIPDRAWNSGYEWITEYTGKTVWFVPSVKMGNEIALCLQRAGKKVVQLNRKSYETEYPKCKNDDWDFVITTDISEMGANFKASRVIDSRKSVKPTIITEGEGRVILGEPSAVTAASAAQRRGRIGRNPSQVGDEYCYGGHTNEDDSNFAHWTEARIMLDNINMPNGLIAQFYQPEREKVYTMDGEYRLRGEERKNFLELLRTADLPVWLAYKVAAAGVSYHDRRWCFDGPRTNTILEDNNEVEVITKLGERKILRPRWIDARVYSDHQALKAFKDFASGKRSQIGLIEVLGKMPEHFMGKTWEALDTMYVVATAEKGGRAHRMALEELPDALQTIALIALLSVMTMGVFFLLMQRKGIGKIGLGGAVLGVATFFCWMAEVPGTKIAGMLLLSLLLMIVLIPEPEKQRSQTDNQLAVFLICVMTLVSAVAANEMGWLDKTKSDISSLFGQRIEVKENFSMGEFLLDLRPATAWSLYAVTTAVLTPLLKHLITSDYINTSLTSINVQASALFTLARGFPFVDVGVSALLLAAGCWGQVTLTVTVTAATLLFCHYAYMVPGWQAEAMRSAQRRTAAGIMKNAVVDGIVATDVPELERTTPIMQKKVGQIMLILVSLAAVVVNPSVKTVREAGILITAAAVTLWENGASSVWNATTAIGLCHIMRGGWLSCLSITWTLIKNMEKPGLKRGGAKGRTLGEVWKERLNQMTKEEFTRYRKEAIIEVDRSAAKHARKEGNVTGGHPVSRGTAKLRWLVERRFLEPVGKVIDLGCGRGGWCYYMATQKRVQEVRGYTKGGPGHEEPQLVQSYGWNIVTMKSGVDVFYRPSECCDTLLCDIGESSSSAEVEEHRTIRVLEMVEDWLHRGPREFCVKVLCPYMPKVIEKMELLQRRYGGGLVRNPLSRNSTHEMYWVSRASGNVVHSVNMTSQVLLGRMEKRTWKGPQYEEDVNLGSGTRAVGKPLLNSDTSKIKNRIERLRREYSSTWHHDENHPYRTWNYHGSYDVKPTGSASSLVNGVVRLLSKPWDTITNVTTMAMTDTTPFGQQRVFKEKVDTKAPEPPEGVKYVLNETTNWLWAFLAREKRPRMCSREEFIRKVNSNAALGAMFEEQNQWRSAREAVEDPKFWEMVDEEREAHLRGECHTCIYNMMGKREKKPGEFGKAKGSRAIWFMWLGARFLEFEALGFLNEDHWLGRKNSGGGVEGLGLQKLGYILREVGTRPGGKIYADDTAGWDTRITRADLENEAKVLELLDGEHRRLARAIIELTYRHKVVKVMRPAADGRTVMDVISREDQRGSGQVVTYALNTFTNLAVQLVRMMEGEGVIGPDDVEKLTKGKGPKVRTWLFENGEERLSRMAVSGDDCVVKPLDDRFATSLHFLNAMSKVRKDIQEWKPSTGWYDWQQVPFCSNHFTELIMKDGRTLVVPCRGQDELVGRARISPGAGWNVRDTACLAKSYAQMWLLLYFHRRDLRLMANAICSAVPVNWVPTGRTTWSIHAGGEWMTTEDMLEVWNRVWIEENEWMEDKTPVEKWSDVPYSGKREDIWCGSLIGTRARATWAENIQVAINQVRAIIGDEKYVDYMSSLKRYEDTTLVEDTVL

>AHW48548.1 polyprotein [West Nile virus]

MSKKPGGPGKSRAVNMLKRGMPRVLSLIGLKRAMLSLIDGKGPIRFVLALLAFFRFTAIAPTRAVLDRWRGVNKQTAMKHLLSFKKELGTLTSAINRRSSKQKKRGGKTGIAVMIGLIASVGAVTLSNFQGKVMMTVNATDVTDVITIPTAAGKNLCIVRAMDVGYMCDDTITYECPVLSAGNDPEDIDCWCTKSAVYVRYGRCTKTRHSRRSRRSLTVQTHGESTLANKKGAWMDSTKATRYLVKTESWILRNPGYALVAAVIGWMLGSNTMQRVVFVVLLLLVAPAYSFNCLGMSNRDFLEGVSGATWVDLVLEGDSCVTIMSKDKPTIDVKMMNMEAANLAEVRSYCYLATVSDLSTKAACPTMGEAHNDKRADPAFVCRQGVVDRGWGNGCGLFGKGSIDTCAKFACSTKAIGRTILKENIKYEVAIFVHGPTTVESHGNYSTQAGATQAGRFSITPAAPSYTLKLGEYGEVTVDCEPRSGIDTNAYYVMTVGTKTFLVHREWFMDLNLPWSSAGSTVWRNRETLMEFEEPHATKQSVIALGSQEGALHQALAGAIPVEFSSNTVKLTSGHLKCRVKMEKLQLKGTTYGVCSKAFKFLGTPADTGHGTVVLELQYTGTDGPCKVPISSVASLNDLTPVGRLVTVNPFVSVATANAKVLIELEPPFGDSYIVVGRGEQQINHHWHKSGSSIGKAFTTTLKGAQRLAALGDTAWDFGSVGGVFTSVGKAVHQVFGGAFRSLFGGMSWITQGLLGALLLWMGINARDRSIALTFLAVGGVLLFLSVNVHADTGCAIDISRQELRCGSGVFIHNDVEAWMDRYKYYPETPQGLAKIIQKAHKEGVCGLRSVSRLEHQMWEAVKDELNTLLKENGVDLSVVVEKQEGMYKSAPKRLTATTEKLEIGWKAWGKSILFAPELANNTFVVDGPETKECPTQNRAWNSLEVEDFGFGLTSTRMFLKVRESNTTECDSKIIGTAVKNNLAIHSDLSYWIESRLNDTWKLERAVLGEVKSCTWPETHTLWGDGILESDLIIPVTLAGPRSNHNRRPGYKTQNQGPWDEGRVEIDFDYCPGTTVTLSESCGHRGPATRTTTESGKLITDWCCRSCTLPPLRYQTDSGCWYGMEIRPQRHDEKTLVQSQVNAYNADMIDPFQLGLLVVFLATQEVLRKRWTAKISMPAILIALLVLVFGGITYTDVLRYVILVGAAFAESNSGGDVVHLALMATFKIQPVFMVASFLKARWTNQENILLMLAAVFFQMAYHDARQILLWEIPDVLNSLAVAWMILRAITFTTTSNVVVPLLALLTPGLRCLNLDVYRILLLMVGIGSLIREKRSAAAKKKGASLLCLALASTGLFNPMILAAGLIACDPNRKRGWPATEVMTAVGLMFAIVGGLAELDIDSMAIPMTIAGLMFAAFVISGKSTDMWIERTADISWESDAEITGSSERVDVRLDDDGNFQLMNDPGAPWKIWMLRMVCLAISAYTPWAILPSVVGFWITLQYTKRGGVLWDTPSPKEYKKGDTTTGVYRIMTRGLLGSYQAGAGVMVEGVFHTLWHTTKGAALMSGEGRLDPYWGSVKEDRLCYGGPWKLQHKWNGQDEVQMIVVEPGKNVKNVQTKPGVFKTPEGEIGAVTLDFPTGTSGSPIVDKNGDVIGLYGNGVIMPNGSYISAIVQGERMDEPIPAGFEPEMLRKKQITVLDLHPGAGKTRRILPQIIKEAINRRLRTAVLAPTRVVAAEMAEALRGLPIRYQTSAVPREHNGNEIVDVMCHATLTHRLMSPHRVPNYNLFVMDEAHFTDPASIAARGYISTKVELGEAAAIFMTATPPGTSDPFPESNSPISDLQTEIPDRAWNSGYEWITEYTGKTVWFVPSVKMGNEIALCLQRAGKKVVQLNRKSYETEYPKCKNDDWDFVITTDISEMGANFKASRVIDSRKSVKPTIITEGEGRVILGEPSAVTAASAAQRRGRIGRNPSQVGDEYCYGGHTNEDDSNFAHWTEARIMLDNINMPNGLIAQFYQPEREKVYTMDGEYRLRGEERKNFLELLRTADLPVWLAYKVAAAGVSYHDRRWCFDGPRTNTILEDNNEVEVITKLGERKILRPRWIDARVYSDHQALKAFKDFASGKRSQIGLIEVLGKMPEHFMGKTWEALDTMYVVATAEKGGRAHRMALEELPDALQTIALIALLSVMTMGVFFLLMQRKGIGKIGLGGAVLGVATFFCWMAEVPGTKIAGMLLLSLLLMIVLIPEPEKQRSQTDNQLAVFLICVMTLVSAVAANEMGWLDKTKSDISSLFGQRIEVKENFSIGEFLLDLRPATAWSLYAVTTAVLTPLLKHLITSDYINTSLTSINVQASALFTLARGFPFVDVGVSALLLAAGCWGQVTLTVTVTAATLLFCHYAYMVPGWQAEAMRSAQRRTAAGIMKNAVVDGIVATDVPELERTTPIMQKKVGQIMLILVSLAAVVVNPSVKTVREAGILITAAAVTLWENGASSVWNATTAIGLCHIMRGGWLSCLSITWTLIKNMEKPGLKRGGAKGRTLGEVWKERLNQMTKEEFTRYRKEAIIEVDRSAAKHARKEGNVTGGHPVSRGTAKLRWLVERRFLEPVGKVIDLGCGRGGWCYYMATQKRVQEVRGYTKGGPGHEEPQLVQSYGWNIVTMKSGVDVFYRPSECCDTLLCDIGESSSSAEVEEHRTIRVLEMVEDWLHRGPREFCVKVLCPYMPKVIEKMELLQRRYGGGLVRNPLSRNSTHEMYWVSRASGNVVHSVNMTSQVLLGRMEKRTWKGPQYEEDVNLGSGTRAVGKPLLNSDTSKIKNRIERLRREYSSTWHHDENHPYRTWNYHGSYDVKPTGSASSLVNGVVRLLSKPWDTITNVTTMAMTDTTPFGQQRVFKEKVDTKAPEPPEGVKYVLNETTNWLWAFLAREKRPRMCSREEFIRKVNSNAALGAMFEEQNQWRSAREAVEDPKFWEMVDEEREAHLRGECHTCIYNMMGKREKKPGEFGKAKGSRAIWFMWLGARFLEFEALGFLNEDHWLGRKNSGGGVEGLGLQKLGYILREVGTRPGGKIYADDTAGWDTRITRADLENEAKVLELLDGEHRRLARAIIELTYRHKVVKVMRPAADGRTVMDVISREDQRGSGQVVTYALNTFTNLAVQLVRMMEGEGVIGPDDVEKLTKGKGPKVRTWLFENGEERLSRMAVSGDDCVVKPLDDRFATSLHFLNAMSKVRKDIQEWKPSTGWYDWQQVPFCSNHFTELIMKDGRTLVVPCRGQDELVGRARISPGAGWNVRDTACLAKSYAQMWLLLYFHRRDLRLMANAICSAVPVNWVPTGRTTWSIHAGGEWMTTEDMLEVWNRVWIEENEWMEDKTPVEKWSDVPYSGKREDIWCGSLIGTRARATWAENIQVAINQVRAIIGDEKYVDYMSSLKRYEDTTLVEDTVL

>AHW48714.1 polyprotein [West Nile virus]

MSKKPGGPGKSRAVNMLKRGMPRVLSLIGLKRAMLSLIDGKGPIRFVLALLAFFRFTAIAPTRAVLDRWRGVNKQTAMKHLLSFKKELGTLTSAINRRSSKQKKRGGKTGIAVMIGLIASVGAVTLSNFQGKVMMTVNATDVTDVITIPTAAGKNLCIVRAMDVGYMCDDTITYECPVLSAGNDPEDIDCWCTKSAVYVRYGRCTKTRHSRRSRRSLTVQTHGESTLANKKGAWMDSTKATRYLVKTESWILRNPGYALVAAVIGWMLGSNTMQRVVFVVLLLLVAPAYSFNCLGMSNRDFLEGVSGATWVDLVLEGDSCVTIMSKDKPTIDVKMMNMEAANLAEVRSYCYLATVSDLSTKAACPTMGEAHNDKRADPAFVCRQGVVDRGWGNGCGLFGKGSIDTCAKFACSTKAIGRTILKENIKYEVAIFVHGPTTVESHGNYSTQAGATQAGRFSITPAAPSYTLKLGEYGEVTVDCEPRSGIDTNAYYVMTVGTKTFLVHREWFMDLNLPWSSAGSTVWRNRETLMEFEEPHATKQSVIALGSQEGALHQALAGAIPVEFSSNTVKLTSGHLKCRVKMEKLQLKGTTYGVCSKAFKFLGTPADTGHGTVVLELQYTGTDGPCKVPISSVASLNDLTPVGRLVTVNPFVSVATANAKVLIELEPPFGDSYIVVGRGEQQINHHWHKSGSSIGKAFTTTLKGAQRLAALGDTAWDFGSVGGVFTSVGKAVHQVFGGAFRSLFGGMSWITQGLLGALLLWMGINARDRSIALTFLAVGGVLLFLSVNVHADTGCAIDISRQELRCGSGVFIHNDVEAWMDRYKYYPETPQGLAKIIQKAHKEGVCGLRSVSRLEHQMWEAVKDELNTLLKENGVDLSVVVEKQEGMYKSAPKRLTATTEKLEIGWKAWGKSILFAPELANNTFVVDGPETKECPTQNRAWNSLEVEDFGFGLTSTRMFLKVRESNTTECDSKIIGTAVKNNLAIHSDLSYWIESRLNDTWKLERAVLGEVKSCTWPETHTLWGDGILESDLIIPVTLAGPRSNHNRRPGYKTQNQGPWDEGRVEIDFDYCPGTTVTLSESCGHRGPATRTTTESGKLITDWCCRSCTLPPLRYQTDSGCWYGMEIRPQRHDEKTLVQSQVNAYNADMIDPFQLGLLVVFLATQEVLRKRWTAKISMPAILIALLVLVFGGITYTDVLRYVILVGAAFAESNSGGDVVHLALMATFKIQPVFMVASFLKARWTNQENILLMLAAVFFQMAYHDARQILLWEIPDVLNSLAVAWMILRAITFTTTSNVVVPLLALLTPGLRCLNLDVYRILLLMVGIGSLIREKRSAAAKKKGASLLCLALASTGLFNPMILAAGLITCDPNRKRGWPATEVMTAVGLMFAIVGGLAELDIDSMAIPMTIAGLMFAAFVISGKSTDMWIERTADISWESDAEITGSSERVDVRLDDDGNFQLMNDPGAPWKIWMLRMVCLAISAYTPWAILPSVVGFWITLQYTKRGGVLWDTPSPKEYKKGDTTTGVYRIMTRGLLGSYQAGAGVMVEGVFHTLWHTTKGAALMSGEGRLDPYWGSVKEDRLCYGGPWKLQHKWNGQDEVQMIVVEPGKNVKNVQTKPGVFKTPEGEIGAVTLDFPTGTSGSPIVDKNGDVIGLYGNGVIMPNGSYISAIVQGERMDEPIPAGFEPEMLRKKQITVLDLHPGAGKTRRILPQIIKEAINRRLRTAVLAPTRVVAAEMAEALRGLPIRYQTSAVPREHNGNEIVDVMCHATLTHRLMSPHRVPNYNLFVMDEAHFTDPASIAARGYISTKVELGEAAAIFMTATPPGTSDPFPESNSPISDLQTEIPDRAWNSGYEWITEYTGKTVWFVPSVKMGNEIALCLQRAGKKVVQLNRKSYETEYPKCKNDDWDFVITTDISEMGANFKASRVIDSRKSVKPTIITEGEGRVILGEPSAVTAASAAQRRGRIGRNPSQVGDEYCYGGHTNEDDSNFAHWTEARIMLDNINMPNGLIAQFYQPEREKVYTMDGEYRLRGEERKNFLELLRTADLPVWLAYKVAAAGVSYHDRRWCFDGPRTNTILEDNNEVEVITKLGERKILRPRWIDARVYSDHQALKAFKDFASGKRSQIGLIEVLGKMPEHFMGKTWEALDTMYVVATAEKGGRAHRMALEELPDALQTIALIALLSVMTMGVFFLLMQRKGIGKIGLGGAVLGVATFFCWMAEVPGTKIAGMLLLSLLLMIVLIPEPEKQRSQTDNQLAVFLICVMTLVSAVAANEMGWLDKTKSDISSLFGQRIEVKENFSMGEFLLDLRPATAWSLYAVTTAVLTPLLKHLITSDYINTSLTSINVQASALFTLARGFPFVDVGVSALLLAAGCWGQVTLTVTVTAATLLFCHYAYMVPGWQAEAMRSAQRRTAAGIMKNAVVDGIVATDVPELERTTPIMQKKVGQIMLILVSLAAVVVNPSVKTVREAGILITAAAVTLWENGASSVWNATTAIGLCHIMRGGWLSCLSITWTLIKNMEKPGLKRGGAKGRTLGEVWKERLNQMTKEEFTRYRKEAIIEVDRSAAKHARKEGNVTGGHPVSRGTAKLRWLVERRFLEPVGKVIDLGCGRGGWCYYMATQKRVQEVRGYTKGGPGHEEPQLVQSYGWNIVTMKSGVDVFYRPSECCDTLLCDIGESSSSAEVEEHRTIRVLEMVEDWLHRGPREFCVKVLCPYMPKVIEKMELLQRRYGGGLVRNPLSRNSTHEMYWVSRASGNVVHSVNMTSQVLLGRMEKRTWKGPQYEEDVNLGSGTRAVGKPLLNSDTSKIKNRIERLRREYSSTWHHDENHPYRTWNYHGSYDVKPTGSASSLVNGVVRLLSKPWDTITNVTTMAMTDTTPFGQQRVFKEKVDTKAPEPPEGVKYVLNETTNWLWAFLAREKRPRMCSREEFIRKVNSNAALGAMFEEQNQWRSAREAVEDPKFWEMVDEEREAHLRGECHTCIYNMMGKREKKPGEFGKAKGSRAIWFMWLGARFLEFEALGFLNEDHWLGRKNSGGGVEGLGLQKLGYILREVGTRPGGKIYADDTAGWDTRITRADLENEAKVLELLDGEHRRLARAIIELTYRHKVVKVMRPAADGRTVMDVISREDQRGSGQVVTYALNTFTNLAVQLVRMMEGEGVIGPDDVEKLTKGKGPKVRTWLFENGEERLSRMAVSGDDCVVKPLDDRFATSLHFLNAMSKVRKDIQEWKPSTGWYDWQQVPFCSNHFTELIMKDGRTLVVPCRGQDELVGRARISPGAGWNVRDTACLAKSYAQMWLLLYFHRRDLRLMANAICSAVPVNWVPTGRTTWSIHAGGEWMTTEDMLEVWNRVWIEENEWMEDKTPVEKWSDVPYSGKREDIWCGSLIGTRARATWAENIQVAINQVRAIIGDEKYVDYMSSLKRYEDTTLVEDTVL

>AYV87285.1 polyprotein [West Nile virus]

MSKKPGGPGKSRAVNMLKRGMPRVLSLIGLKRAMLSLIDGKGPIRFVLALLAFFRFTAIAPTRAVLDRWRGVNKQTAMKHLLSFKKELGTLTSAINRRSSKQKKRGGKTGIAVIIGLIASVGAVTLSNFQGKVMMTVNATDVTDVITIPTAAGKNLCIVRAMDVGYMCDDTITYECPVLSAGNDPEDIDCWCTKSAVYVRYGRCTKTRHSRRSRRSLTVQTHGESTLANKKGAWMDSTKATRYLVKTESWILRNPGYALVAAVIGWMLGSNTMQRVVFVVLLLLVAPAYSFNCLGMSNRDFLEGVSGATWVDLVLEGDSCVTIMSKDKPTIDVKMMNMEAANLAEVRSYCYLATVSDLSTKAACPTMGEAHNDKRADPAFVCRQGVVDRGWGNGCGLFGKGSIDTCAKFACSTKAIGRTILKENIKYEVAIFVHGPTTVESHGNYSTQAGATQAGRFSITPAAPSYTLKLGEYGEVTVDCEPRSGIDTNAYYVMTVGTKTFLVHREWFMDLNLPWSSAGSTVWRNRETLMEFEEPHATKQSVIALGSQEGALHQALAGAIPVEFSSNTVKLTSGHLKCRVKMEKLQLKGTTYGVCSKAFKFLGTPADTGHGTVVLELQYTGTDGPCKVPISSVASLNDLTPVGRLVTVNPFVSVATANAKVLIELEPPFGDSYIVVGRGEQQINHHWHKSGSSIGKAFTTTLKGAQRLAALGDTAWDFGSVGGVFTSVGKAVHQVFGGAFRSLFGGMSWITQGLLGALLLWMGINARDRSIALTFLAVGGVLLFLSVNVHADTGCAIDISRQELRCGSGVFIHNDVEAWMDRYKYYPETPQGLAKIIQKAHKEGVCGLRSVSRLEHQMWEAVKDELNTLLKENGVDLSVVVEKQEGMYKSAPKRLTATTEKLEIGWKAWGKSILFAPELANNTFVVDGPETKECPTQNRAWNSLEVEDFGFGLTSTRMFLKVRESNTTECDSKIIGTAVKNNLAIHSDLSYWIESRLNDTWKLERAVLGEVKSCTWPETHTLWGDGILESDLIIPVTLAGPRSNHNRRPGYKTQNQGPWDEGRVEIDFDYCPGTTVTLSESCGHRGPATRTTTESGKLITDWCCRSCTLPPLRYQTDSGCWYGMEIRPQRHDEKTLVQSQVNAYNADMIDPFQLGLLVVFLATQEVLRKRWTAKISMPAILIALLVLVFGGITYTDVLRYVILVGAAFAESNSGGDVVHLALMATFKIQPVFMVASFLKARWTNQENILLMLAAVFFQMAYHDARQILLWEIPDVLNSLAVAWMILRAITFTTTSNVVVPLLALLTPGLRCLNLDVYRILLLMVGIGSLIREKRSAAAKKKGASLLCLALASTGLFNPMILAAGLIACDPNRKRGWPATEVMTAVGLMFAIVGGLAELDIDSMAIPMTIAGLMFAAFVISGKSTDMWIERTADISWESDAEITGSSERVDVRLDDDGNFQLMNDPGAPWKIWMLRMVCLAISAYTPWAILPSVVGFWITLQYTKRGGVLWDTPSPKEYKKGDTTTGVYRIMTRGLLGSYQAGAGVMVEGVFHTLWHTTKGAALMSGEGRLDPYWGSVKEDRLCYGGPWKLQHKWNGQDEVQMIVVEPGKNVKNVQTKPGVFKTPEGEIGAVTLDFPTGTSGSPIVDKNGDVIGLYGNGVIMPNGSYISAIVQGERMDEPIPAGFEPEMLRKKQITVLDLHPGAGKTRRILPQIIKEAINRRLRTAVLAPTRVVAAEMAEALRGLPIRYQTSAVPREHNGNEIVDVMCHATLTHRLMSPHRVPNYNLFVMDEAHFTDPASIAARGYISTKVELGEAAAIFMTATPPGTSDPFPESNSPISDLQTEIPDRAWNSGYEWITEYTGKTVWFVPSVKMGNEIALCLQRAGKKVVQLNRKSYETEYPKCKNDDWDFVITTDISEMGANFKASRVIDSRKSVKPTIITEGEGRVILGEPSAVTAASAAQRRGRIGRNPSQVGDEYCYGGHTNEDDSNFAHWTEARIMLDNINMPNGLIAQFYQPEREKVYTMDGEYRLRGEERKNFLELLRTADLPVWLAYKVAAAGVSYHDRRWCFDGPRTNTILEDNNEVEVITKLGERKILRPRWIDARVYSDHQALKAFKDFASGKRSQIGLIEVLGKMPEHFMGKTWEALDTMYVVATAEKGGRAHRMALEELPDALQTIALIALLSVMTMGVFFLLMQRKGIGKIGLGGAVLGVATFFCWMAEVPGTKIAGMLLLSLLLMIVLIPEPEKQRSQTDNQLAVFLICVMTLVSAVAANEMGWLDKTKSDISSLFGQRIEVKENFSMGEFLLDLRPATAWSLYAVTTAVLTPLLKHLITSDYINTSLTSINVQASALFTLARGFPFVDVGVSALLLAAGCWGQVTLTVTVTAATLLFCHYAYMVPGWQAEAMRSAQRRTAAGIMKNAVVDGIVATDVPELERTTPIMQKKVGQIMLILVSLAAVVVNPSVKTVREAGILITAAAVTLWENGASSVWNATTAIGLCHIMRGGWLSCLSITWTLIKNMEKPGLKRGGAKGRTLGEVWKERLNQMTKEEFTRYRKEAIIEVDRSAAKHARKEGNVTGGHPVSRGTAKLRWLVERRFLEPVGKVIDLGCGRGGWCYYMATQKRVQEVRGYTKGGPGHEEPQLVQSYGWNIVTMKSGVDVFYRPSECCDTLLCDIGESSSSAEVEEHRTIRVLEMVEDWLHRGPREFCVKVLCPYMPKVIEKMELLQRRYGGGLVRNPLSRNSTHEMYWVSRASGNVVHSVNMTSQVLLGRMEKRTWKGPQYEEDVNLGSGTRAVGKPLLNSDTSKIKNRIERLRREYSSTWHHDENHPYRTWNYHGSYDVKPTGSASSLVNGVVRLLSKPWDTITNVTTMAMTDTTPFGQQRVFKEKVDTKAPEPPEGVKYVLNETTNWLWAFLAREKRPRMCSREEFIRKVNSNAALGAMFEEQNQWRSAREAVEDPKFWEMVDEEREAHLRGECHTCIYNMMGKREKKPGEFGKAKGSRAIWFMWLGARFLEFEALGFLNEDHWLGRKNSGGGVEGLGLQKLGYILREVGTRPGGKIYADDTAGWDTRITRADLENEAKVLELLDGEHRRLARAIIELTYRHKVVKVMRPAADGRTVMDVISREDQRGSGQVVTYALNTFTNLAVQLVRMMEGEGVIGPDDVEKLTKGKGPKVRTWLFENGEERLSRMAVSGDDCVVKPLDDRFATSLHFLNAMSKVRKDIQEWKPSTGWYDWQQVPFCSNHFTELIMKDGRTLVVPCRGQDELVGRARISPGAGWNVRDTACLAKSYAQMWLLLYFHRRDLRLMANAICSAVPVNWVPTGRTTWSIHAGGEWMTTEDMLEVWNRVWIEENEWMEDKTPVEKWSDVPYSGKREDIWCGSLIGTRARATWAENIQVAINQVRAIIGDEKYVDYMSSLKRYEDTTLVEDTVL

>ANW69271.1 genome polyprotein [West Nile virus]

MSKKPGGPGKSRAVNMLKRGMPRVLSLIGLKRAMLSLIDGKGPIRFVLALLAFFRFTAIAPTRAVLDRWRGVNKQTAMKHLLSFKKELGTLTSAINRRSSKQKKRGGKTGIAVMIGLIASVGAVTLSNFQGKVMMTVNATDVTDVITIPTAAGKNLCIVRAMDVGYMCDDTITYECPVLSAGNDPEDIDCWCTKSAVYVRYGRCTKTRHSRRSRRSLTVQTHGESTLANKKGAWMDSTKATRYLVKTESWILRNPGYALVAAVIGWMLGSNTMQRVVFVVLLLLVAPAYSFNCLGMSNRDFLEGVSGATWVDLVLEGDSCVTIMSKDKPTIDVKMMNMEAANLAEVRSYCYLATVSDLSTKAACPTMGEAHNDKRADPAFVCRQGVVDRGWGNGCGLFGKGSIDTCAKFACSTKAIGRTILKENIKYEVAIFVHGPTTVESHGNYSTQAGATQAGRFSITPAAPSYTLKLGEYGEVTVDCEPRSGIDTNAYYVMTVGTKTFLVHREWFMDLNLPWSSAGSTVWRNRETLMEFEEPHATKQSVIALGSQEGALHQALAGAIPVEFSSNTVKLTSGHLKCRVKMEKLQLKGTTYGVCSKAFKFLGTPADTGHGTVVLELQYTGTDGPCKVPISSVASLNDLTPVGRLVTVNPFVSVATANAKVLIELEPPFGDSYIVVGRGEQQINHHWHKSGSSIGKAFTTTLKGAQRLAALGDTAWDFGSVGGVFTSVGKAVHQVFGGAFRSLFGGMSWITQGLLGALLLWMGINARDRSIALTFLAVGGVLLFLSVNVHADTGCAIDISRQELRCGSGVFIHNDVEAWMDRYKYYPETPQGLAKIIQKAHKEGVCGLRSVSRLEHQMWEAVKDELNTLLKENGVDLSVVVEKQEGMYKSAPKRLTATTEKLEIGWKAWGKSILFAPELANNTFVVDGPETKECPTQNRAWNSLEVEDFGFGLTSTRMFLKVRESNTNECDSKIIGTAVKNNLAIHSDLSYWIESRLNDTWKLERAVLGEVKSCTWPETHTLWGDGILESDLIIPVTLAGPRSNHNRRPGYKTQNQGPWDEGRVEIDFDYCPGTTVTLSESCGHRGPATRTTTESGKLITDWCCRSCTLPPLRYQTDSGCWYGMEIRPQRHDEKTLVQSQVNAYNADMIDPFQLGLLVVFLATQEVLRKRWTAKISMPAILIALLVLVFGGITYTDVLRYVILVGAAFAESNSGGDVVHLALMATFKIQPVFMVASFLKARWTNQENILLMLAAVFFQMAYHDARQILLWEIPDVLNSLAVAWMILRAITFTTTSNVVVPLLALLTPGLRCLNLDVYRILLLMVGIGSLIREKRSAAAKKKGASLLCLALASTGLFNPMILAAGLIACDPNRKRGWPATEVMTAVGLMFAIVGGLAELDIDSMAIPMTIAGLMFAAFVISGKSTDMWIERTADISWESDAEITGSSERVDVRLDDDGNFQLMNDPGAPWKIWMLRMVCLAISAYTPWAILPSVVGFWITLQYTKRGGVLWDTPSPKEYKKGDTTTGVYRIMTRGLLGSYQAGAGVMVEGVFHTLWHTTKGAALMSGEGRLDPYWGSVKEDRLCYGGPWKLQHKWNGQDEVQMIVVEPGKNVKNVQTKPGVFKTPEGEIGAVTLDFPTGTSGSPIVDKNGDVIGLYGNGVIMPNGSYISAIVQGERMDEPIPAGFEPEMLRKKQITVLDLHPGAGKTRRILPQIIKEAINRRLRTAVLAPTRVVAAEMAEALRGLPIRYQTSAVPREHNGNEIVDVMCHATLTHRLMSPHRVPNYNLFVMDEAHFTDPASIAARGYISTKVELGEAAAIFMTATPPGTSDPFPESNSPISDLQTEIPDRAWNSGYEWITEYTGKTVWFVPSVKMGNEIALCLQRAGKKVVQLNRKSYETEYPKCKNDDWDFVITTDISEMGANFKASRVIDSRKSVKPTIITEGEGRVILGEPSAVTAASAAQRRGRIGRNPSQVGDEYCYGGHTNEDDSNFAHWTEARIMLDNINMPNGLIAQFYQPEREKVYTMDGEYRLRGEERKNFLELLRTADLPVWLAYKVAAAGVSYHDRRWCFDGPRTNTILEDNNEVEVITKLGERKILRPRWIDARVYSDHQALKAFKDFASGKRSQIGLIEVLGKMPEHFMGKTWEALDTMYVVATAEKGGRAHRMALEELPDALQTIALIALLSVMTMGVFFLLMQRKGIGKIGLGGAVLGVATFFCWMAEVPGTKIAGMLLLSLLLMIVLIPEPEKQRSQTDNQLAVFLICVMTLVSAVAANEMGWLDKTKSDISSLFGQRIEVKENFSMGEFLLDLRPATAWSLYAVTTAVLTPLLKHLITSDYINTSLTSINVQASALFTLARGFPFVDVGVSALLLAAGCWGQVTLTVTVTAATLLFCHYAYMVPGWQAEAMRSAQRRTAAGIMKNAVVDGIVATDVPELERTTPIMQKKVGQIMLILVSLAAVVVNPSVKTVREAGILITAAAVTLWENGASSVWNATTAIGLCHIMRGGWLSCLSITWTLIKNMEKPGLKRGGAKGRTLGEVWKERLNQMTKEEFTRYRKEAIIEVDRSAAKHARKEGNVTGGHPVSRGTAKLRWLVERRFLEPVGKVIDLGCGRGGWCYYMATQKRVQEVRGYTKGGPGHEEPQLVQSYGWNIVTMKSGVDVFYRPSECCDTLLCDIGESSSSAEVEEHRTIRVLEMVEDWLHRGPREFCVKVLCPYMPKVIEKMELLQRRYGGGLVRNPLSRNSTHEMYWVSRASGNVVHSVNMTSQVLLGRMEKRTWKGPQYEEDVNLGSGTRAVGKPLLNSDTSKIKNRIERLRREYSSTWHHDENHPYRTWNYHGSYDVKPTGSASSLVNGVVRLLSKPWDTITNVTTMAMTDTTPFGQQRVFKEKVDTKAPEPPEGVKYVLNETTNWLWAFLAREKRPRMCSREEFIRKVNSNAALGAMFEEQNQWRSAREAVEDPKFWEMVDEEREAHLRGECHTCIYNMMGKREKKPGEFGKAKGSRAIWFMWLGARFLEFEALGFLNEDHWLGRKNSGGGVEGLGLQKLGYILREVGTRPGGKIYADDTAGWDTRITRADLENEAKVLELLDGEHRRLARAIIELTYRHKVVKVMRPAADGRTVMDVISREDQRGSGQVVTYALNTFTNLAVQLVRMMEGEGVIGPDDVEKLTKGKGPKVRTWLFENGEERLSRMAVSGDDCVVKPLDDRFATSLHFLNAMSKVRKDIQEWKPSTGWYDWQQVPFCSNHFTELIMKDGRTLVVPCRGQDELVGRARISPGAGWNVRDTACLAKSYAQMWLLLYFHRRDLRLMANAICSAVPVNWVPTGRTTWSIHAGGEWMTTEDMLEVWNRVWIEENEWMEDKTPVEKWSDVPYSGKREDIWCGSLIGTRARATWAENIQVAINQVRAIIGDEKYVDYMSSLKRYEDTTLVEDTVL

>ADL27934.1 polyprotein [West Nile virus]

MSKKPGGPGKSRAVNMLKRGMPRVLSLIGLKRAMLSLIDGKGPIRFVLALLAFFRFTAIAPTRAVLDRWRGVNKQTAMKHLLSFKKELGTLTSAINRRSSKQKKRGGKTGIAVMIGLIASVGAVTLSNFQGKVMMTVNATDVTDVITIPTAAGKNLCIVRAMDVGYMCDDTITYECPVLSAGNDPEDIDCWCTKSAVYVRYGRCTKTRHSRRSRRSLTVQTHGESTLANKKGAWMDSTKATRYLVKTESWILRNPGYALVAAVIGWMLGSNTMQRVVFVVLLLLVAPAYSFNCLGMSNRDFLEGVSGATWVDLVLEGDSCVTIMSKDKPTIDVKMMNMEAANLAEVRSYCYLATVSDLSTKAACPTMGEAHNDKRADPAFVCRQGVVDRGWGNGCGLFGKGSIDTCAKFACSTKAIGRTILKENIKYEVAIFVHGPTTVESHGNYSTQAGATQTGRFSITPAAPSYTLKLGEYGEVTVDCEPRSGIDTNAYYVMTVGTKTFLVHREWFMDLNLPWSSAGSTVWRNRETLMEFEEPHATKQSVIALGSQEGALHQALAGAIPVEFSSNTVKLTSGHLKCRVKMEKLQLKGTTYGVCSKAFKFLGTPADTGHGTVVLELQYTGTDGPCKVPISSVASLNDLTPVGRLVTVNPFVSVATANAKVLIELEPPFGDSYIVVGRGEQQINHHWHKSGSSIGKAFTTTLKGAQRLAALGDTAWDFGSVGGVFTSVGKAVHQVFGGAFRSLFGGMSWITQGLLGALLLWMGINARDRSIALTFLAVGGVLLFLSVNVHADTGCAIDISRQELRCGSGVFIHNDVEAWMDRYKYYPETPQGLAKIIQKAHKEGVCGLRSVSRLEHQMWEAVKDELNTLLKENGVDLSVVVEKQEGMYKSAPKRLTATTEKLEIGWKAWGKSILFAPELANNTFVVDGPETKECPTQNRAWNSLEVEDFGFGLTSTRMFLKVRESNTTECDSKIIGTAVKNNLAIHSDLSYWIESRLNDTWKLERAVLGEVKSCTWPETHTLWGDGILESDLIIPVTLAGPRSNHNRRPGYKTQNQGPWDEGRVEIDFDYCPGTTVTLSESCGHRGPATRTTTESGKLITDWCCRSCTLPPLRYQTDSGCWYGMEIRPQRHDEKTLVQSQVNAYNADMIDPFQLGLLVVFLATQEVLRKRWTAKISMPAILIALLVLVFGGITYTDVLRYVILVGAAFAESNSGGDVVHLALMATFKIQPVFMVASFLKARWTNQENILLMLAAVFFQMAYHDARQILLWEIPDVLNSLAVAWMILRAITFTTTSNVVVPLLALLTPGLRCLNLDVYRILLLMVGIGSLIREKRSAAAKKKGASLLCLALASTGLFNPMILAAGLIACDPNRKRGWPATEVMTAVGLMFAIVGGLAELDIDSMAIPMTIAGLMFAAFVISGKSTDMWIERTADISWESDAEITGSSERVDVRLDDDGNFQLMNDPGAPWKIWMLRMVCLAISAYTPWAILPSVVGFWITLQYTKRGGVLWDTPSPKEYKKGDTTTGVYRIMTRGLLGSYQAGAGVMVEGVFHTLWHTTKGAALMSGEGRLDPYWGSVKEDRLCYGGPWKLQHKWNGQDEVQMIVVEPGKNVKNVQTKPGVFKTPEGEIGAVTLDFPTGTSGSPIVDKNGDVIGLYGNGVIMPNGSYISAIVQGERMDEPIPAGFEPEMLRKKQITVLDLHPGAGKTRRILPQIIKEAINRRLRTAVLAPTRVVAAEMAEALRGLPIRYQTSAVPREHNGNEIVDVMCHATLTHRLMSPHRVPNYNLFVMDEAHFTDPASIAARGYISTKVELGEAAAIFMTATPPGTSDPFPESNSPISDLQTEIPDRAWNSGYEWITEYTGKTVWFVPSVKMGNEIALCLQRAGKKVVQLNRKSYETEYPKCKNDDWDFVITTDISEMGANFKASRVIDSRKSVKPTIITEGEGRVILGEPSAVTAASAAQRRGRIGRNPSQVGDEYCYGGHTNEDDSNFAHWTEARIMLDNINMPNGLIAQFYQPEREKVYTMDGEYRLRGEERKNFLELLRTADLPVWLAYKVAAAGVSYHDRRWCFDGPRTNTILEDNNEVEVITKLGERKILRPRWIDARVYSDHQALKAFKDFASGKRSQIGLIEVLGKMPEHFMGKTWEALDTMYVVATAEKGGRAHRMALEELPDALQTIALIALLSVMTMGVFFLLMQRKGIGKIGLGGAVLGVATFFCWMAEVPGTKIAGMLLLSLLLMIVLIPEPEKQRSQTDNQLAVFLICVMTLVSAVAANEMGWLDKTKSDISSLFGQRIEVKENFSMGEFLLDLRPATAWSLYAVTTAVLTPLLKHLITSDYINTSLTSINVQASALFTLARGFPFVDVGVSALLLAAGCWGQVTLTVTVTAATLLFCHYAYMVPGWQAEAMRSAQRRTAAGIMKNAVVDGIVATDVPELERTTPIMQKKVGQIMLILVSLAAVVVNPSVKTVREAGILITAAAVTLWENGASSVWNATTAIGLCHIMRGGWLSCLSITWTLIKNMEKPGLKRGGAKGRTLGEVWKERLNQMTKEEFTRYRKEAIIEVDRSAAKHARKEGNVTGGHPVSRGTAKLRWLVERRFLEPVGKVIDLGCGRGGWCYYMATQKRVQEVRGYTKGGPGHEEPQLVQSYGWNIVTMKSGVDVFYRPSECCDTLLCDIGESSSSAEVEEHRTIRVLEMVEDWLHRGPREFCVKVLCPYMPKVIEKMELLQRRYGGGLVRNPLSRNSTHEMYWVSRASGNVVHSVNMTSQVLLGRMEKRTWKGPQYEEDVNLGSGTRAVGKPLLNSDTSKIKNRIERLRREYSSTWHHDENHPYRTWNYHGSYDVKPTGSASSLVNGVVRLLSKPWDTITNVTTMAMTDTTPFGQQRVFKEKVDTKAPEPPEGVKYVLNETTNWLWAFLAREKRPRMCSREEFIRKVNSNAALGAMFEEQNQWRSAREAVEDPKFWEMVDEEREAHLRGECHTCIYNMMGKREKKPGEFGKAKGSRAIWFMWLGARFLEFEALGFLNEDHWLGRKNSGGGVEGLGLQKLGYILREVGTRPGGKIYADDTAGWDTRITRADLENEAKVLELLDGEHRRLARAIIELTYRHKVVKVMRPAADGRTVMDVISREDQRGSGQVVTYALNTFTNLAVQLVRMMEGEGVIGPDDVEKLTKGKGPKVRTWLFENGEERLSRMAVSGDDCVVKPLDDRFATSLHFLNAMSKVRKDIQEWKPSTGWYDWQQVPFCSNHFTELIMKDGRTLVVPCRGQDELVGRARISPGAGWNVRDTACLAKSYAQMWLLLYFHRRDLRLMANAICSAVPVNWVPTGRTTWSIHAGGEWMTTEDMLEVWNRVWIEENEWMEDKTPVEKWSDVPYSGKREDIWCGSLIGTRARATWAENIQVAINQVRAIIGDEKYVDYMSSLKRYEDTTLVEDTVL

>ADL27948.1 polyprotein [West Nile virus]

MSKKPGGPGKSRAVNMLKRGMPRVLSLIGLKRAMLSLIDGKGPIRFVLALLAFFRFTAIAPTRAVLDRWRGVNKQTAMKHLLSFKKELGTLTSAINRRSSKQKKRGGKTGIAVMIGLIASVGAVTLSNFQGKVMMTVNATDVTDVITIPTAAGKNLCIVRAMDVGYMCDDTITYECPVLSAGNDPEDIDCWCTKSAVYVRYGRCTKTRHSRRSRRSLTVQTHGESTLANKKGAWMDSTKATRYLVKTESWILRNPGYALVAAVIGWILGSNTMQRVVFVVLLLLVAPAYSFNCLGMSNRDFLEGVSGATWVDLVLEGDSCVTIMSKDKPTIDVKMMNMEAANLAEVRSYCYLATVSDLSTKAACPTMGEAHNDKRADPAFVCRQGVVDRGWGNGCGLFGKGSIDTCAKFACSTKAIGRTILKENIKYEVAIFVHGPTTVESHGNYSTQAGATQAGRFSITPAAPSYTLKLGEYGEVTVDCEPRSGIDTNAYYVMTVGTKTFLVHREWFMDLNLPWSSAGSTVWRNRETLMEFEEPHATKQSVIALGSQEGALHQALAGAIPVEFSSNTVKLTSGHLKCRVKMEKLQLKGTTYGVCSKAFKFLGTPADTGHGTVVLELQYTGTDGPCKVPISSVASLNDLTPVGRLVTVNPFVSVATANAKVLIELEPPFGDSYIVVGRGEQQINHHWHKSGSSIGKAFTTTLKGAQRLAALGDTAWDFGSVGGVFTSVGKAVHQVFGGAFRSLFGGMSWITQGLLGALLLWMGINARDRSIALTFLAVGGVLLFLSVNVHADTGCAIDISRQELRCGSGVFIHNDVEAWMDRYKYYPETPQGLAKIIQKAHKEGVCGLRSVSRLEHQMWEAVKDELNTLLKENGVDLSVVVEKQEGMYKSAPKRLTATTEKLEIGWKAWGKSILFAPELANNTFVVDGPETKECPTQNRAWNSLEVEDFGFGLTSTRMFLKVRESNTTECDSKIIGTAVKNNLAIHSDLSYWIESRLNDTWKLERAVLGEVKSCTWPETHTLWGDGILESDLIIPVTLAGPRSNHNRRPGYKTQNQGPWDEGRVEIDFDYCPGTTVTLSESCGHRGPATRTTTESGKLITDWCCRSCTLPPLRYQTDSGCWYGMEIRPQRHDEKTLVQSQVNAYNADMIDPFQLGLLVVFLATQEVLRKRWTAKISMPAILIALLVLVFGGITYTDVLRYVILVGAAFAESNSGGDVVHLALMATFKIQPVFMVASFLKARWTNQENILLMLAAVFFQMAYHDARQILLWEIPDVLNSLAVAWMILRAITFTTTSNVVVPLLALLTPGLRCLNLDVYRILLLMVGIGSLIREKRSAAAKKKGASLLCLALASTGLFNPMILAAGLIACDPNRKRGWPATEVMTAVGLMFAIVGGLAELDIDSMAIPMTIAGLMFAAFVISGKSTDMWIERTADISWESDAEITGSSERVDVRLDDDGNFQLMNDPGAPWKIWMLRMVCLAISAYTPWAILPSVVGFWITLQYTKRGGVLWDTPSPKEYKKGDTTTGVYRIMTRGLLGSYQAGAGVMVEGVFHTLWHTTKGAALMSGEGRLDPYWGSVKEDRLCYGGPWKLQHKWNGQDEVQMIVVEPGKNVKNVQTKPGVFKTPEGEIGAVTLDFPTGTSGSPIVDKNGDVIGLYGNGVIMPNGSYISAIVQGERMDEPIPAGFEPEMLRKKQITVLDLHPGAGKTRRILPQIIKEAINRRLRTAVLAPTRVVAAEMAEALRGLPIRYQTSAVPREHNGNEIVDVMCHATLTHRLMSPHRVPNYNLFVMDEAHFTDPASIAARGYISTKVELGEAAAIFMTATPPGTSDPFPESNSPISDLQTEIPDRAWNSGYEWITEYTGKTVWFVPSVKMGNEIALCLQRAGKKVVQLNRKSYETEYPKCKNDDWDFVITTDISEMGANFKASRVIDSRKSVKPTIITEGEGRVILGEPSAVTAASAAQRRGRIGRNPSQVGDEYCYGGHTNEDDSNFAHWTEARIMLDNINMPNGLIAQFYQPEREKVYTMDGEYRLRGEERKNFLELLRTADLPVWLAYKVAAAGVSYHDRRWCFDGPRTNTILEDNNEVEVITKLGERKILRPRWIDARVYSDHQALKAFKDFASGKRSQIGLIEVLGKMPEHFMGKTWEALDTMYVVATAEKGGRAHRMALEELPDALQTIALIALLSVMTMGVFFLLMQRKGIGKIGLGGAVLGVATFFCWMAEVPGTKIAGMLLLSLLLMIVLIPEPEKQRSQTDNQLAVFLICVMTLVSAVAANEMGWLDKTKSDISSLFGQRIEVKENFSMGEFLLDLRPATAWSLYAVTTAVLTPLLKHLITSDYINTSLTSINVQASALFTLARGFPFVDVGVSALLLAAGCWGQVTLTVTVTAATLLFCHYAYMVPGWQAEAMRSAQRRTAAGIMKNAVVDGIVATDVPELERTTPIMQKKVGQIMLILVSLAAVVVNPSVKTVREAGILITAAAVTLWENGASSVWNATTAIGLCHIMRGGWLSCLSITWTLIKNMEKPGLKRGGAKGRTLGEVWKERLNQMTKEEFTRYRKEAIIEVDRSAAKHARKEGNVTGGHPVSRGTAKLRWLVERRFLEPVGKVIDLGCGRGGWCYYMATQKRVQEVRGYTKGGPGHEEPQLVQSYGWNIVTMKSGVDVFYRPSECCDTLLCDIGESSSSAEVEEHRTIRVLEMVEDWLHRGPREFCVKVLCPYMPKVIEKMELLQRRYGGGLVRNPLSRNSTHEMYWVSRASGNVVHSVNMTSQVLLGRMEKRTWKGPQYEEDVNLGSGTRAVGKPLLNSDTSKIKNRIERLRREYSSTWHHDENHPYRTWNYHGSYDVKPTGSASSLVNGVVRLLSKPWDTITNVTTMAMTDTTPFGQQRVFKEKVDTKAPEPPEGVKYVLNETTNWLWAFLAREKRPRMCSREEFIRKVNSNAALGAMFEEQNQWRSAREAVEDPKFWEMVDEEREAHLRGECHTCIYNMMGKREKKPGEFGKAKGSRAIWFMWLGARFLEFEALGFLNEDHWLGRKNSGGGVEGLGLQKLGYILREVGTRPGGKIYADDTAGWDTRITRADLENEAKVLELLDGEHRRLARAIIELTYRHKVVKVMRPAADGRTVMDVISREDQRGSGQVVTYALNTFTNLAVQLVRMMEGEGVIGPDDVEKLTKGKGPKVRTWLFENGEERLSRMAVSGDDCVVKPLDDRFATSLHFLNAMSKVRKDIQEWKPSTGWYDWQQVPFCSNHFTELIMKDGRTLVVPCRGQDELVGRARISPGAGWNVRDTACLAKSYAQMWLLLYFHRRDLRLMANAICSAVPVNWVPTGRTTWSIHAGGEWMTTEDMLEVWNRVWIEENEWMEDKTPVEKWSDVPYSGKREDIWCGSLIGTRARATWAENIQVAINQVRAIIGDEKYVDYMSSLKRYEDTTLVEDTVL

>ANW68464.1 genome polyprotein [West Nile virus]

MSKKPGGPGKSRAVNMLKRGMPRVLSLIGLKRAMLSLIDGKGPIRFVLALLAFFRFTAIAPTRAVLDRWRGVNKQTAMKHLLSFKKELGTLTSAINRRSSKQKKRGGKTGIAVMIGLIASVGAVTLSNFQGKVMMTVNATDVTDVITIPTAAGKNLCIVRAMDVGYMCDDTITYECPVLSAGNDPEDIDCWCTKSAVYVRYGRCTKTRHSRRSRRSLTVQTHGESTLANKKGAWMDSTKATRYLVKTESWILRNPGYALVAAVIGWMLGSNTMQRVVFVVLLLLVAPAYSFNCLGMSNRDFLEGVSGATWVDLVLEGDSCVTIMSKDKPTIDVKMMNMEAANLAEVRSYCYLATVSDLSTKAACPTMGEAHNDKRADPAFVCRQGVVDRGWGNGCGLFGKGSIDTCAKFACSTKAIGRTILKENIKYEVAIFVHGPTTVESHGNYSTQAGATQAGRFSITPAAPSYTLKLGEYGEVTVDCEPRSGIDTNAYYVMTVGTKTFLVHREWFMDLNLPWSSAGSTVWRNRETLMEFEEPHATKQSVIALGSQEGALHQALAGAIPVEFSSNTVKLTSGHLKCRVKMEKLQLKGTTYGVCSKAFKFLGTPADTGHGTVVLELQYTGTDGPCKVPISSVASLNDLTPVGRLVTVNPFVSVATANAKVLIELEPPFGDSYIVVGRGEQQINHHWHKSGSSIGKAFTTTLKGAQRLAALGDTAWDFGSVGGVFTSVGKAVHQVFGGAFRSLFGGMSWITQGLLGALLLWMGINARDRSIALTFLAVGGVLLFLSVNVHADTGCAIDISRQELRCGSGVFIHNDVEAWMDRYKYYPETPQGLAKIIQKAHKEGVCGLRSVSRLEHQMWEAVKDELNTLLKENGVDLSVVVEKQEGMYKSAPKRLTATTEKLEIGWKAWGKSILFAPELANNTFVVDGPETKECPTQNRAWNSLEVEDFGFGLTSTRMFLKVRESNTTECDSKIIGTAVKNNLAIHSDLSYWIESRLNDTWKLERAVLGEVKSCTWPETHTLWGDGILESDLIIPVTLAGPRSNHNRRPGYKTQNQGPWDEGRVEIDFDYCPGTTVTLSESCGHRGPATRTTTESGKLITDWCCRSCTLPPLRYQTDSGCWYGMEIRPQRHDEKTLVQSQVNAYNADMIDPFQLGLLVVFLATQEVLRKRWTAKISMPAILIALLVLVFGGITYTDVLRYVILVGAAFAESNSGGDVVHLALIATFKIQPVFMVASFLKARWTNQENILLMLAAVFFQMAYHDARQILLWEIPDVLNSLAVAWMILRAITFTTTSNVVVPLLALLTPGLRCLNLDVYRILLLMVGIGSLIREKRSAAAKKKGASLLCLALASTGLFNPMILAAGLIACDPNRKRGWPATEVMTAVGLMFAIVGGLAELDIDSMAIPMTIAGLMFAAFVISGKSTDMWIERTADISWESDAEITGSSERVDVRLDDDGNFQLMNDPGAPWKIWMLRMVCLAISAYTPWAILPSVVGFWITLQYTKRGGVLWDTPSPKEYKKGDTTTGVYRIMTRGLLGSYQAGAGVMVEGVFHTLWHTTKGAALMSGEGRLDPYWGSVKEDRLCYGGPWKLQHKWNGQDEVQMIVVEPGKNVKNVQTKPGVFKTPEGEIGAVTLDFPTGTSGSPIVDKNGDVIGLYGNGVIMPNGSYISAIVQGERMDEPIPAGFEPEMLRKKQITVLDLHPGAGKTRRILPQIIKEAINRRLRTAVLAPTRVVAAEMAEALRGLPIRYQTSAVPREHNGNEIVDVMCHATLTHRLMSPHRVPNYNLFVMDEAHFTDPASIAARGYISTKVELGEAAAIFMTATPPGTSDPFPESNSPISDLQTEIPDRAWNSGYEWITEYTGKTVWFVPSVKMGNEIALCLQRAGKKVVQLNRKSYETEYPKCKNDDWDFVITTDISEMGANFKASRVIDSRKSVKPTIITEGEGRVILGEPSAVTAASAAQRRGRIGRNPSQVGDEYCYGGHTNEDDSNFAHWTEARIMLDNINMPNGLIAQFYQPEREKVYTMDGEYRLRGEERKNFLELLRTADLPVWLAYKVAAAGVSYHDRRWCFDGPRTNTILEDNNEVEVITKLGERKILRPRWIDARVYSDHQALKAFKDFASGKRSQIGLIEVLGKMPEHFMGKTWEALDTMYVVATAEKGGRAHRMALEELPDALQTIALIALLSVMTMGVFFLLMQRKGIGKIGLGGAVLGVATFFCWMAEVPGTKIAGMLLLSLLLMIVLIPEPEKQRSQTDNQLAVFLICVMTLVSAVAANEMGWLDKTKSDISSLFGQRIEVKENFSMGEFLLDLRPATAWSLYAVTTAVLTPLLKHLITSDYINTSLTSINVQASALFTLARGFPFVDVGVSALLLAAGCWGQVTLTVTVTAATLLFCHYAYMVPGWQAEAMRSAQRRTAAGIMKNAVVDGIVATDVPELERTTPIMQKKVGQIMLILVSLAAVVVNPSVKTVREAGILITAAAVTLWENGASSVWNATTAIGLCHIMRGGWLSCLSITWTLIKNMEKPGLKRGGAKGRTLGEVWKERLNQMTKEEFTRYRKEAIIEVDRSAAKHARKEGNVTGGHPVSRGTAKLRWLVERRFLEPVGKVIDLGCGRGGWCYYMATQKRVQEVRGYTKGGPGHEEPQLVQSYGWNIVTMKSGVDVFYRPSECCDTLLCDIGESSSSAEVEEHRTIRVLEMVEDWLHRGPREFCVKVLCPYMPKVIEKMELLQRRYGGGLVRNPLSRNSTHEMYWVSRASGNVVHSVNMTSQVLLGRMEKRTWKGPQYEEDVNLGSGTRAVGKPLLNSDTSKIKNRIERLRREYSSTWHHDENHPYRTWNYHGSYDVKPTGSASSLVNGVVRLLSKPWDTITNVTTMAMTDTTPFGQQRVFKEKVDTKAPEPPEGVKYVLNETTNWLWAFLAREKRPRMCSREEFIRKVNSNAALGAMFEEQNQWRSAREAVEDPKFWEMVDEEREAHLRGECHTCIYNMMGKREKKPGEFGKAKGSRAIWFMWLGARFLEFEALGFLNEDHWLGRKNSGGGVEGLGLQKLGYILREVGTRPGGKIYADDTAGWDTRITRADLENEAKVLELLDGEHRRLARAIIELTYRHKVVKVMRPAADGRTVMDVISREDQRGSGQVVTYALNTFTNLAVQLVRMMEGEGVIGPDDVEKLTKGKGPKVRTWLFENGEERLSRMAVSGDDCVVKPLDDRFATSLHFLNAMSKVRKDIQEWKPSTGWYDWQQVPFCSNHFTELIMKDGRTLVVPCRGQDELVGRARISPGAGWNVRDTACLAKSYAQMWLLLYFHRRDLRLMANAICSAVPVNWVPTGRTTWSIHAGGEWMTTEDMLEVWNRVWIEENEWMEDKTPVEKWSDVPYSGKREDIWCGSLIGTRARATWAENIQVAINQVRAIIGDEKYVDYMSSLKRYEDTTLVEDTVL

>ADK62405.1 polyprotein [West Nile virus]

MSKKPGGPGKSRAVNMLKRGMPRVLSLIGLKRAMLSLIDGKGPIRFVLALLAFFRFTAIAPTRAVLDRWRGVNKQTAMKHLLSFKKELGTLTSAINRRSSKQKKRGGKTGIAVMIGLIASVGAVTLSNFQGKVMMTVNATDVTDVITIPTAAGKNLCIVRAMDVGYMCDDTITYECPVLSAGNDPEDIDCWCTKSAVYVRYGRCTKTRHSRRSRRSLTVQTHGESTLANKKGAWMDSTKATRYLVKTESWILRNPGYALVAAVIGWMLGSNTMQRVVFVVLLLLVAPAYSFNCLGMSNRDFLEGVSGATWVDLVLEGDSCVTIMSKDKPTIDVKMMNMEAANLAEVRSYCYLATVSDLSTKAACPTMGEAHNDKRADPAFVCRQGVVDRGWGNGCGLFGKGSIDTCAKFACSTKAIGRTILKENIKYEVAIFVHGPTTVESHGNYSTQAGATQAGRFSITPAAPSYTLKLGEYGEVTVDCEPRSGIDTNAYYVMTVGTKTFLVHREWFMDLNLPWSSAGSTVWRNRETLMEFEEPHATKQSVIALGSQEGALHQALAGAIPVEFSSNTVKLTSGHLKCRVKMEKLQLKGTTYGVCSKAFKFLGTPADTGHGTVVLELQYTGTDGPCKVPISSVASLNDLTPVGRLVTVNPFVSVATANAKVLIELEPPFGDSYIVVGRGEQQINHHWHKSGSSIGKAFTTTLKGAQRLAALGDTAWDFGSVGGVFTSVGKAVHQVFGGAFRSLFGGMSWITQGLLGALLLWMGINARDRSIALTFLAVGGVLLFLSVNVHADTGCAIDISRQELRCGSGVFIHNDVEAWMDRYKYYPETPQGLAKIIQKAHKEGVCGLRSVSRLEHQMWEAVKDELNTLLKENGVDLSVVVEKQEGMYKSAPKRLTATTEKLEIGWKAWGKSILFAPELANNTFVVDGPETKECPTQNRAWNSLEVEDFGFGLTSTRMFLKVRESNTTECDSKIIGTAVKNNLAIHSDLSYWIESRLNDTWKLERAVLGEVKSCTWPETHTLWGDGILESDLIIPVTLAGPRSNHNRRPGYKTQNQGPWDEGRVEIDFDYCPGTTVTLSESCGHRGPATRTTTESGKLITDWCCRSCTLPPLRYKTDSGCWYGMEIRPQRHDEKTLVQSQVNAYNADMIDPFQLGLLVVFLATQEVLRKRWTAKISMPAILIALLVLVFGGITYTDVLRYVILVGAAFAESNSGGDVVHLALMATFKIQPVFMVASFLKARWTNQENILLMLAAVFFQMAYHDARQILLWEIPDVLNSLAVAWMILRAITFTTTSNVVVPLLALLTPGLRCLNLDVYRILLLMVGIGSLIREKRSAAAKKKGASLLCLALASTGLFNPMILAAGLIACDPNRKRGWPATEVMTAVGLMFAIVGGLAELDIDSMAIPMTIAGLMFAAFVISGKSTDMWIERTADISWESDAEITGSSERVDVRLDDDGNFQLMNDPGAPWKIWMLRMVCLAISAYTPWAILPSVVGFWITLQYTKRGGVLWDTPSPKEYKKGDTTTGVYRIMTRGLLGSYQAGAGVMVEGVFHTLWHTTKGAALMSGEGRLDPYWGSVKEDRLCYGGPWKLQHKWNGQDEVQMIVVEPGKNVKNVQTKPGVFKTPEGEIGAVTLDFPTGTSGSPIVDKNGDVIGLYGNGVIMPNGSYISAIVQGERMDEPIPAGFEPEMLRKKQITVLDLHPGAGKTRRILPQIIKEAINRRLRTAVLAPTRVVAAEMAEALRGLPIRYQTSAVPREHNGNEIVDVMCHATLTHRLMSPHRVPNYNLFVMDEAHFTDPASIAARGYISTKVELGEAAAIFMTATPPGTSDPFPESNSPISDLQTEIPDRAWNSGYEWITEYTGKTVWFVPSVKMGNEIALCLQRAGKKVVQLNRKSYETEYPKCKNDDWDFVITTDISEMGANFKASRVIDSRKSVKPTIITEGEGRVILGEPSAVTAASAAQRRGRIGRNPSQVGDEYCYGGHTNEDDSNFAHWTEARIMLDNINMPNGLIAQFYQPEREKVYTMDGEYRLRGEERKNFLELLRTADLPVWLAYKVAAAGVSYHDRRWCFDGPRTNTILEDNNEVEVITKLGERKILRPRWIDARVYSDHQALKAFKDFASGKRSQIGLIEVLGKMPEHFMGKTWEALDTMYVVATAEKGGRAHRMALEELPDALQTIALIALLSVMTMGVFFLLMQRKGIGKIGLGGAVLGVATFFCWMAEVPGTKIAGMLLLSLLLMIVLIPEPEKQRSQTDNQLAVFLICVMTLVSAVAANEMGWLDKTKSDISSLFGQRIEVKENFSMGEFLLDLRPATAWSLYAVTTAVLTPLLKHLITSDYINTSLTSINVQASALFTLARGFPFVDVGVSALLLAAGCWGQVTLTVTVTAATLLFCHYAYMVPGWQAEAMRSAQRRTAAGIMKNAVVDGIVATDVPELERTTPIMQKKVGQIMLILVSLAAVVVNPSVKTVREAGILITAAAVTLWENGASSVWNATTAIGLCHIMRGGWLSCLSITWTLIKNMEKPGLKRGGAKGRTLGEVWKERLNQMTKEEFTRYRKEAIIEVDRSAAKHARKEGNVTGGHPVSRGTAKLRWLVERRFLEPVGKVIDLGCGRGGWCYYMATQKRVQEVRGYTKGGPGHEEPQLVQSYGWNIVTMKSGVDVFYRPSECCDTLLCDIGESSSSAEVEEHRTIRVLEMVEDWLHRGPREFCVKVLCPYMPKVIEKMELLQRRYGGGLVRNPLSRNSTHEMYWVSRASGNVVHSVNMTSQVLLGRMEKRTWKGPQYEEDVNLGSGTRAVGKPLLNSDTSKIKNRIERLRREYSSTWHHDENHPYRTWNYHGSYDVKPTGSASSLVNGVVRLLSKPWDTITNVTTMAMTDTTPFGQQRVFKEKVDTKAPEPPEGVKYVLNETTNWLWAFLAREKRPRMCSREEFIRKVNSNAALGAMFEEQNQWRSAREAVEDPKFWEMVDEEREAHLRGECHTCIYNMMGKREKKPGEFGKAKGSRAIWFMWLGARFLEFEALGFLNEDHWLGRKNSGGGVEGLGLQKLGYILREVGTRPGGKIYADDTAGWDTRITRADLENEAKVLELLDGEHRRLARAIIELTYRHKVVKVMRPAADGRTVMDVISREDQRGSGQVVTYALNTFTNLAVQLVRMMEGEGVIGPDDVEKLTKGKGPKVRTWLFENGEERLSRMAVSGDDCVVKPLDDRFATSLHFLNAMSKVRKDIQEWKPSTGWYDWQQVPFCSNHFTELIMKDGRTLVVPCRGQDELVGRARISPGAGWNVRDTACLAKSYAQMWLLLYFHRRDLRLMANAICSAVPVNWVPTGRTTWSIHAGGEWMTTEDMLEVWNRVWIEENEWMEDKTPVEKWSDVPYSGKREDIWCGSLIGTRARATWAENIQVAINQVRAIIGDEKYVDYMSSLKRYEDTTLVEDTVL

>AHW48694.1 polyprotein [West Nile virus]

MSKKPGGPGKSRAVNMLKRGMPRVLSLIGLKRAMLSLIDGKGPIRFVLALLAFFRFTAIAPTRAVLDRWRGVNKQTAMKHLLSFKKELGTLTSAINRRSSKQKKRGGKTGIAVMIGLIASVGAVTLSNFQGKVMMTVNATDVTDVITIPTAAGKNLCIVRAMDVGYMCDDTITYECPVLSAGNDPEDIDCWCTKSAVYVRYGRCTKTRHSRRSRRSLTVQTHGESTLANKKGAWMDSTKATRYLVKTESWILRNPGYALVAAVIGWMLGSNTMQRVVFVVLLLLVAPAYSFNCLGMSNRDFLEGVSGATWVDLVLEGDSCVTIMSKDKPTIDVKMMNMEAANLAEVRSYCYLATVSDLSTKAACPTMGEAHNDKRADPAFVCRQGVVDRGWGNGCGLFGKGSIDTCAKFACSTKAIGRTILKENIKYEVAIFVHGPTTVESHGNYSTQAGATQAGRFSITPAAPSYTLKLGEYGEVTVDCEPRSGIDTNAYYVMTVGTKTFLVHREWFMDLNLPWSSAGSTVWRNRETLMEFEEPHATKQSVIALGSQEGALHQALAGAIPVEFSSNTVKLTSGHLKCRVKMEKLQLKGTTYGVCSKAFKFLGTPADTGHGTVVLELQYTGTDGPCKVPISSVASLNDLTPVGRLVTVNPFVSVATANAKVLIELEPPFGDSYIVVGRGEQQINHHWHKSGSSIGKAFTTTLKGAQRLAALGDTAWDFGSVGGVFTSVGKAVHQVFGGAFRSLFGGMSWITQGLLGALLLWMGINARDRSIALTFLAVGGVLLFLSVNVHADTGCAIDISRQELRCGSGVFIHNDVEAWMDRYKYYPETPQGLAKIIQKAHKEGVCGLRSVSRLEHQMWEAVKDELNTLLKENGVDLSVVVEKQEGMYKSAPKRLTATTEKLEIGWKAWGKSILFAPELANNTFVVDGPETKECPTQNRAWNSLEVEDFGFGLTSTRMFLKVRESNTTECDSKIIGTAVKNNLAIHSDLSYWIESRLNDTWKLERAVLGEVKSCTWPETHTLWGDGILESDLIIPVTLAGPRSNHNRRPGYKTQNQGPWDEGRVEIDFDYCPGTTVTLSESCGHRGPATRTTTESGKLITDWCCRSCTLPPLRYQTDSGCWYGMEIRPQRHDEKTLVQSQVNAYNADMIDPFQLGLLVVFLATQEVLRKRWTAKISMPAILIALLVLVFGGITYTDVLRYVILVGAAFAESNSGGDVVHLALMATFKIQPVFMVASFLKARWTNQENILLMLAAVFFQMAYHDARQILLWEIPDVLNSLAVAWMILRAITFTTTSNVVVPLLALLTPGLRCLNLDVYRILLLMVGIGSLIREKRSAAAKKKGASLLCLALASTGLFNPMILAAGLIACDPNRKRGWPATEVMTAVGLMFAIVGGLAELDIDSMAIPMTIAGLMFAAFVISGKSTDMWIERTADISWESDAEITGSSERVDVRLDDDGNFQLMNDPGAPWKIWMLRMVCLAISAYTPWAILPSVVGFWITLQYTKRGGVLWDTPSPKEYKKGDTTTGVYRIMTRGLLGSYQAGAGVMVEGVFHTLWHTTKGAALMSGEGRLDPYWGSVKEDRLCYGGPWKLQHKWNGQDEVQMIVVEPGKNVKNVQTKPGVFKTPEGEIGAVTLDFPTGTSGSPIVDKNGDVIGLYGNGVIMPNGSYISAIVQGERMDEPIPAGFEPEMLRKKQITVLDLHPGAGKTRRILPQIIKEAINRRLRTAVLAPTRVVAAEMAEALRGLPIRYQTSAVPREHNGNEIVDVMCHATLTHRLMSPHRVPNYNLFVMDEAHFTDPASIAARGYISTKVELGEAAAIFMTATPPGTSDPFPESNSPISDLQTEIPDRAWNSGYEWITEYTGKTVWFVPSVKMGNEIALCLQRAGKKVVQLNRKSYETEYPKCKNDDWDFVITTDISEMGANFKASRVIDSRKSVKPTIITEGEGRVILGEPSAVTAASAAQRRGRIGRNPSQVGDEYCYGGHTNEDDSNFAHWTEARIMLDNINMPNGLIAQFYQPEREKVYTMDGEYRLRGEERKNFLELLRTADLPVWLAYKVAAAGVSYHDRRWCFDGPRTNTILEDNNEVEVITKLGERKILRPRWIDARVYSDHQALKAFKDFASGKRSQIGLIEVLGKMPEHFMGKTWEALDTMYVVATAEKGGRAHRMALEELPDALQTIALIALLSVMTMGVFFLLMQRKGIGKIGLGGTVLGVATFFCWMAEVPGTKIAGMLLLSLLLMIVLIPEPEKQRSQTDNQLAVFLICVMTLVSAVAANEMGWLDKTKSDISSLFGQRIEVKENFSMGEFLLDLRPATAWSLYAVTTAVLTPLLKHLITSDYINTSLTSINVQASALFTLARGFPFVDVGVSALLLAAGCWGQVTLTVTVTAATLLFCHYAYMVPGWQAEAMRSAQRRTAAGIMKNAVVDGIVATDVPELERTTPIMQKKVGQIMLILVSLAAVVVNPSVKTVREAGILITAAAVTLWENGASSVWNATTAIGLCHIMRGGWLSCLSITWTLIKNMEKPGLKRGGAKGRTLGEVWKERLNQMTKEEFTRYRKEAIIEVDRSAAKHARKEGNVTGGHPVSRGTAKLRWLVERRFLEPVGKVIDLGCGRGGWCYYMATQKRVQEVRGYTKGGPGHEEPQLVQSYGWNIVTMKSGVDVFYRPSECCDTLLCDIGESSSSAEVEEHRTIRVLEMVEDWLHRGPREFCVKVLCPYMPKVIEKMELLQRRYGGGLVRNPLSRNSTHEMYWVSRASGNVVHSVNMTSQVLLGRMEKRTWKGPQYEEDVNLGSGTRAVGKPLLNSDTSKIKNRIERLRREYSSTWHHDENHPYRTWNYHGSYDVKPTGSASSLVNGVVRLLSKPWDTITNVTTMAMTDTTPFGQQRVFKEKVDTKAPEPPEGVKYVLNETTNWLWAFLAREKRPRMCSREEFIRKVNSNAALGAMFEEQNQWRSAREAVEDPKFWEMVDEEREAHLRGECHTCIYNMMGKREKKPGEFGKAKGSRAIWFMWLGARFLEFEALGFLNEDHWLGRKNSGGGVEGLGLQKLGYILREVGTRPGGKIYADDTAGWDTRITRADLENEAKVLELLDGEHRRLARAIIELTYRHKVVKVMRPAADGRTVMDVISREDQRGSGQVVTYALNTFTNLAVQLVRMMEGEGVIGPDDVEKLTKGKGPKVRTWLFENGEERLSRMAVSGDDCVVKPLDDRFATSLHFLNAMSKVRKDIQEWKPSTGWYDWQQVPFCSNHFTELIMKDGRTLVVPCRGQDELVGRARISPGAGWNVRDTACLAKSYAQMWLLLYFHRRDLRLMANAICSAVPVNWVPTGRTTWSIHAGGEWMTTEDMLEVWNRVWIEENEWMEDKTPVEKWSDVPYSGKREDIWCGSLIGTRARATWAENIQVAINQVRAIIGDEKYVDYMSSLKRYEDTTLVEDTVL

>ANW69463.1 genome polyprotein [West Nile virus]

MSKKPGGPGKSRAVNMLKRGMPRVLSLIGLKRAMLSLIDGKGPIRFVLALLAFFRFTAIAPTRAVLDRWRGVNKQTAMKHLLSFKKELGTLTSAINRRSSKQKKRGGKTGIAVMIGLIASVGAVTLSNFQGKVMMTVNATDVTDVITIPTAAGKNLCIVRAMDVGYMCDDTITYECPVLSAGNDPEDIDCWCTKSAVYVRYGRCTKTRHSRRSRRSLTVQTHGESTLANKKGAWMDSTKATRYLVKTESWILRNPGYALVAAVIGWMLGSNTMQRVVFVVLLLLVAPAYSFNCLGMSNRDFLEGVSGATWVDLVLEGDSCVTIMSKDKPTIDVKMMNMEAANLAEVRSYCYLATVSDLSTKAACPTMGEAHNDKRADPAFVCRQGVVDRGWGNGCGLFGKGSIDTCAKFACSTKAIGRTILKENIKYEVAIFVHGPTTVESHGNYSTQAGATQAGRFSITPAAPSYTLKLGEYGEVTVDCEPRSGIDTNAYYVMTVGTKTFLVHREWFMDLNLPWSSAGSTVWRNRETLMEFEEPHATKQSVIALGSQEGALHQALAGAIPVEFSSNTVKLTSGHLKCRVKMEKLQLKGTTYGVCSKAFKFLGTPADTGHGTVVLELQYTGTDGPCKVPISSVASLNDLTPVGRLVTVNPFVSVATANAKVLIELEPPFGDSYIVVGRGEQQINHHWHKSGSSIGKAFTTTLKGAQRLAALGDTAWDFGSVGGVFTSVGKAVHQVFGGAFRSLFGGMSWITQGLLGALLLWMGINARDRSIALTFLAVGGVLLFLSVNVHADTGCAIDISRQELRCGSGVFIHNDVEAWMDRYKYYPETPQGLAKIIQKAHKEGVCGLRSVSRLEHQMWEAVKDELNTLLKENGVDLSVVVEKQEGMYKSAPKRLTATTEKLEIGWKAWGKSILFAPELANNTFVVDGPETKECPTQNRAWNSLEVEDFGFGLTSTRMFLKVRESNTTECDSKIIGTAVKNNLAIHSDLSYWIESRLNDTWKLERAVLGEVKSCTWPETHTLWGDGILESDLIIPVTLAGPRSNHNRRPGYKTQNQGPWDEGRVEIDFDYCPGTTVTLSESCGHRGPATRTTTESGKLITDWCCRSCTLPPLRYQTDSGCWYGMEIRPQRHDEKTLVQSQVNAYNADMIDPFQLGLLVVFLATQEVLRKRWTAKISMPAILIALLVLVFGGITYTDVLRYVILVGAAFAESNSGGDVVHLALMATFKIQPVFMVASFLKARWTNQENILLMLAAVFFQMAYHDARQILLWEIPDVLNSLAVAWMILRAITFTTTSNVVVPLLALLTPGLRCLNLDVYRILLLMVGIGSLIREKRSAAAKKKGASLLCLALASTGLFNPMILAAGLIACDPNRKRGWPATEVMTAVGLMFAIVGGLAELDIDSMAIPMTIAGLMFAAFVISGKSTDMWIERTADISWESDAEITGSSERVDVRLDDDGNFQLMNDPGAPWKIWMLRMVCLAISAYTPWAILPSVVGFWITLQYTKRGGVLWDTPSPKEYKKGDTTTGVYRIMTRGLLGSYQAGAGVMVEGVFHTLWHTTKGAALMSGEGRLDPYWGSVKEDRLCYGGPWKLQHKWNGQDEVQMIVVEPGKNVKNVQTKPGVFKTPEGEIGAVTLDFPTGTSGSPIVDKNGDVIGLYGNGVIIPNGSYISAIVQGERMDEPIPAGFEPEMLRKKQITVLDLHPGAGKTRRILPQIIKEAINRRLRTAVLAPTRVVAAEMAEALRGLPIRYQTSAVPREHNGNEIVDVMCHATLTHRLMSPHRVPNYNLFVMDEAHFTDPASIAARGYISTKVELGEAAAIFMTATPPGTSDPFPESNSPISDLQTEIPDRAWNSGYEWITEYTGKTVWFVPSVKMGNEIALCLQRAGKKVVQLNRKSYETEYPKCKNDDWDFVITTDISEMGANFKASRVIDSRKSVKPTIITEGEGRVILGEPSAVTAASAAQRRGRIGRNPSQVGDEYCYGGHTNEDDSNFAHWTEARIMLDNINMPNGLIAQFYQPEREKVYTMDGEYRLRGEERKNFLELLRTADLPVWLAYKVAAAGVSYHDRRWCFDGPRTNTILEDNNEVEVITKLGERKILRPRWIDARVYSDHQALKAFKDFASGKRSQIGLIEVLGKMPEHFMGKTWEALDTMYVVATAEKGGRAHRMALEELPDALQTIALIALLSVMTMGVFFLLMQRKGIGKIGLGGAVLGVATFFCWMAEVPGTKIAGMLLLSLLLMIVLIPEPEKQRSQTDNQLAVFLICVMTLVSAVAANEMGWLDKTKSDISSLFGQRIEVKENFSMGEFLLDLRPATAWSLYAVTTAVLTPLLKHLITSDYINTSLTSINVQASALFTLARGFPFVDVGVSALLLAAGCWGQVTLTVTVTAATLLFCHYAYMVPGWQAEAMRSAQRRTAAGIMKNAVVDGIVATDVPELERTTPIMQKKVGQIMLILVSLAAVVVNPSVKTVREAGILITAAAVTLWENGASSVWNATTAIGLCHIMRGGWLSCLSITWTLIKNMEKPGLKRGGAKGRTLGEVWKERLNQMTKEEFTRYRKEAIIEVDRSAAKHARKEGNVTGGHPVSRGTAKLRWLVERRFLEPVGKVIDLGCGRGGWCYYMATQKRVQEVRGYTKGGPGHEEPQLVQSYGWNIVTMKSGVDVFYRPSECCDTLLCDIGESSSSAEVEEHRTIRVLEMVEDWLHRGPREFCVKVLCPYMPKVIEKMELLQRRYGGGLVRNPLSRNSTHEMYWVSRASGNVVHSVNMTSQVLLGRMEKRTWKGPQYEEDVNLGSGTRAVGKPLLNSDTSKIKNRIERLRREYSSTWHHDENHPYRTWNYHGSYDVKPTGSASSLVNGVVRLLSKPWDTITNVTTMAMTDTTPFGQQRVFKEKVDTKAPEPPEGVKYVLNETTNWLWAFLAREKRPRMCSREEFIRKVNSNAALGAMFEEQNQWRSAREAVEDPKFWEMVDEEREAHLRGECHTCIYNMMGKREKKPGEFGKAKGSRAIWFMWLGARFLEFEALGFLNEDHWLGRKNSGGGVEGLGLQKLGYILREVGTRPGGKIYADDTAGWDTRITRADLENEAKVLELLDGEHRRLARAIIELTYRHKVVKVMRPAADGRTVMDVISREDQRGSGQVVTYALNTFTNLAVQLVRMMEGEGVIGPDDVEKLTKGKGPKVRTWLFENGEERLSRMAVSGDDCVVKPLDDRFATSLHFLNAMSKVRKDIQEWKPSTGWYDWQQVPFCSNHFTELIMKDGRTLVVPCRGQDELVGRARISPGAGWNVRDTACLAKSYAQMWLLLYFHRRDLRLMANAICSAVPVNWVPTGRTTWSIHAGGEWMTTEDMLEVWNRVWIEENEWMEDKTPVEKWSDVPYSGKREDIWCGSLIGTRARATWAENIQVAINQVRAIIGDEKYVDYMSSLKRYEDTTLVEDTVL

>ANW69085.1 genome polyprotein [West Nile virus]

MSKKPGGPGKSRAVNMLKRGMPRVLSLIGLKRAMLSLIDGKGPIRFVLALLAFFRFTAIAPTRAVLDRWRGVNKQTAMKHLLSFKKELGTLTSAINRRSSKQKKRGGKTGIAVMIGLIASVGAVTLSNFQGKVMMTVNATDVTDVITIPTAAGKNLCIVRAMDVGYMCDDTITYECPVLSAGNDPEDIDCWCTKSAVYVRYGRCTKTRHSRRSRRSLTVQTHGESTLANKKGAWMDSTKATRYLVKTESWILRNPGYALVAAVIGWMLGSNTMQRVVFVVLLLLVAPAYSFNCLGMSNRDFLEGVSGATWVDLVLEGDSCVTIMSKDKPTIDVKMMNMEAANLAEVRSYCYLATVSDLSTKAACPTMGEAHNDKRADPAFVCRQGVVDRGWGNGCGLFGKGSIDTCAKFACSTKAIGRTILKENIKYEVAIFVHGPTTVESHGNYSTQAGATQAGRFSITPAAPSYTLKLGEYGEVTVDCEPRSGIDTNAYYVMTVGTKTFLVHREWFMDLNLPWSSAGSTVWRNRETLMEFEEPHATKQSVIALGSQEGALHQALAGAIPVEFSSNTVKLTSGHLKCRVKMEKLQLKGTTYGVCSKAFKFLGTPADTGHGTVVLELQYTGTDGPCKVPISSVASLNDLTPVGRLVTVNPFVSVATANAKVLIELEPPFGDSYIVVGRGEQQINHHWHKSGSSIGKAFTTTLKGAQRLAALGDTAWDFGSVGGVFTSVGKAVHQVFGGAFRSLFGGMSWITQGLLGALLLWMGINARDRSIALTFLAVGGVLLFLSVNVHADTGCAIDISRQELRCGSGVFIHNDVEAWMDRYKYYPETPQGLAKIIQKAHKEGVCGLRSVSRLEHQMWEAVKDELNTLLKENGVDLSVVVEKQEGMYKSAPKRLTATTEKLEIGWKAWGKSILFAPELANNTFVVDGPETKECPTQNRAWNSLEVEDFGFGLTSTRMFLKVRESNTTECDSKIIGTAVKNNLAIHSDLSYWIESRLNDTWKLERAVLGEVKSCTWPETHTLWGDGILESDLIIPVTLAGPRSNHNRRPGYKTQNQGPWDEGRVEIDFDYCPGTTVTLSESCGHRGPATRTTTESGKLITDWCCRSCTLPPLRYQTDSGCWYGMEIRPQRHDEKTLVQSQVNAYNADMIDPFQLGLLVVFLATQEVLRKRWTAKISMPAILIALLVLVFGGITYTDVLRYVILVGAAFAESNSGGDVVHLALMATFKIQPVFMVASFLKARWTNQENILLMLAAVFFQMAYHDARQILLWEIPDVLNSLAVAWMILRAITFTTTSNVVVPLLALLTPGLRCLNLDVYRILLLMVGIGSLIREKRSAAAKKKGASLLCLALASTGLFNPMILAAGLIACDPNRKRGWPATEVMTAVGLMFAIVGGLAELDIDSMAIPMTIAGLMFAAFVISGKSTDMWIERTADISWESDAEITGSSERVDVRLDDDGNFQLMNDPGAPWKIWMLRMVCLAISAYTPWAILPSVVGFWITLQYTKRGGVLWDTPSPKEYKKGDTTTGVYRIMTRGLLGSYQAGAGVMVEGVFHTLWHTTKGAALMSGEGRLDPYWGSVKEDRLCYGGPWKLQHKWNGQDEVQMIVVEPGKNVKNVQTKPGVFKTPEGEIGAVTLDFPTGTSGSPIVDKNGDVIGLYGNGVIMPNGSYISAIVQGERMDEPIPAGFEPEMLRKKQITVLDLHPGAGKTRRILPQIIKEAINRRLRTAVLAPTRVVAAEMAEALRGLPIRYQTSAVPREHNGNEIVDVMCHATLTHRLMSPHRVPNYNLFVMDEAHFTDPASIAARGYISTKVELGEAAAIFMTATPPGTSDPFPESNSPISDLQTEIPDRAWNSGYEWITEYTGKTVWFVPSVKMGNEIALCLQRAGKKVVQLNRKSYETEYPKCKNDDWDFVITTDISEMGANFKASRVIDSRKSVKPTIITEGEGRVILGEPSAVTAASAAQRRGRIGRNPSQVGDEYCYGGHTNEDDSNFAHWTEARIMLDNINMPNGLIAQFYQPEREKVYTMDGEYRLRGEERKNFLELLRTADLPVWLAYKVAAAGVSYHDRRWCFDGPRTNTILEDNNEVEVITKLGERKILRPRWIDARVYSDHQALKAFKDFASGKRSQIGLIEVLGKMPEHFMGKTWEALDTMYVVATAEKGGRAHRMALEELPDALQTIALIALLSVMTMGVFFLLMQRKGIGKIGLGGAVLGVATFFCWMAEVPGTKIAGMLLLSLLLMIVLIPEPEKQRSQTDNQLAVFLICVMTLVSAVAANEMGWLDKTKSDISSLFGQRIEVKENFSMGEFLLDLRPATAWSLYAVTTAVLTPLLKHLITSDYINTSLTSINVQASALFTLARGFPFVDVGVSALLLAAGCWGQVTLTVTVTAATLLFCHYAYMVPGWQAEAMRSAQRRTAAGIMKNAVVDGIVATDVPELERTTPIMQKKVGQIMLILVSLAAVVVNPSVKTVREAGILITAAAVTLWENGASSVWNATTAIGLCHIMRGGWLSCLSITWTLIKNMEKPGLKRGGAKGRTLGEVWKERLNQMTKEEFTRYRKEAIIEVDRSAAKHARKEGNVTGGHPVSRGTAKLRWLVERRFLEPVGKVIDLGCGRGGWCYYMATQKRVQEVRGYTKGGPGHEEPQLVQSYGWNIVTMKSGVDVFYRPSECCDTLLCDIGESSSSAEVEEHRTIRVLEMVEDWLHRGPREFCVKVLCPYMPKVIEKMELLQRRYGGGLVRNPLSRNSTHEMYWVSRASGNVVHSVNMTSQVLLGRMEKRTWKGPQYEEDVNLGSGTRAVGKPLLNSDTSKIKNRIERLRREYSSTWHHDENHPYRTWNYHGSYDVKPTGSASSLVNGVVRLLSKPWDTITNVTTMAMTDTTPFGQQRVFKEKVDTKAPEPPEGVKYVLNETTNWLWAFLAREKRPRMCSREEFIRKVNSNAALGAMFEEQNQWRSAREAVEDPKFWEIVDEEREAHLRGECHTCIYNMMGKREKKPGEFGKAKGSRAIWFMWLGARFLEFEALGFLNEDHWLGRKNSGGGVEGLGLQKLGYILREVGTRPGGKIYADDTAGWDTRITRADLENEAKVLELLDGEHRRLARAIIELTYRHKVVKVMRPAADGRTVMDVISREDQRGSGQVVTYALNTFTNLAVQLVRMMEGEGVIGPDDVEKLTKGKGPKVRTWLFENGEERLSRMAVSGDDCVVKPLDDRFATSLHFLNAMSKVRKDIQEWKPSTGWYDWQQVPFCSNHFTELIMKDGRTLVVPCRGQDELVGRARISPGAGWNVRDTACLAKSYAQMWLLLYFHRRDLRLMANAICSAVPVNWVPTGRTTWSIHAGGEWMTTEDMLEVWNRVWIEENEWMEDKTPVEKWSDVPYSGKREDIWCGSLIGTRARATWAENIQVAINQVRAIIGDEKYVDYMSSLKRYEDTTLVEDTVL

>AHW48544.1 polyprotein [West Nile virus]

MSKKPGGPGKSRAVNMLKRGMPRVLSLIGLKRAMLSLIDGKGPIRFVLALLAFFRFTAIAPTRAVLDRWRGVNKQTAMKHLLSFKKELGTLTSAINRRSSKQKKRGGKTGIAVMIGLIASVGAVTLSNFQGKVMMTVNATDVTDVITIPTAAGKNLCIVRAMDVGYMCDDTITYECPVLSAGNDPEDIDCWCTKSAVYVRYGRCTKTRHSRRSRRSLTVQTHGESTLANKKGAWMDSTKATRYLVKTESWILRNPGYALVAAVIGWMLGSNTMQRVVFVVLLLLVAPAYSFNCLGMSNRDFLEGVSGATWVDLVLEGDSCVTIMSKDKPTIDVKMMNMEAANLAEVRSYCYLATVSDLSTKAACPTMGEAHNDKRADPAFVCRQGVVDRGWGNGCGLFGKGSIDTCAKFACSTKAIGRTILKENIKYEVAIFVHGPTTVESHGNYSTQAGATQAGRFSITPAAPSYTLKLGEYGEVTVDCEPRSGIDTNAYYVMTVGTKTFLVHREWFMDLNLPWSSAGSTVWRNRETLMEFEEPHATKQSVIALGSQEGALHQALAGAIPVEFSSNTVKLTSGHLKCRVKMEKLQLKGTTYGVCSKAFKFLGTPADTGHGTVVLELQYTGTDGPCKVPISSVASLNDLTPVGRLVTVNPFVSVATANAKVLIELEPPFGDSYIVVGRGEQQINHHWHKSGSSIGKAFTTTLKGAQRLAALGDTAWDFGSVGGVFTSVGKAVHQVFGGAFRSLFGGMSWITQGLLGALLLWMGINARDRSIALTFLAIGGVLLFLSVNVHADTGCAIDISRQELRCGSGVFIHNDVEAWMDRYKYYPETPQGLAKIIQKAHKEGVCGLRSVSRLEHQMWEAVKDELNTLLKENGVDLSVVVEKQEGMYKSAPKRLTATTEKLEIGWKAWGKSILFAPELANNTFVVDGPETKECPTQNRAWNSLEVEDFGFGLTSTRMFLKVRESNTTECDSKIIGTAVKNNLAIHSDLSYWIESRLNDTWKLERAVLGEVKSCTWPETHTLWGDGILESDLIIPVTLAGPRSNHNRRPGYKTQNQGPWDEGRVEIDFDYCPGTTVTLSESCGHRGPATRTTTESGKLITDWCCRSCTLPPLRYQTDSGCWYGMEIRPQRHDEKTLVQSQVNAYNADMIDPFQLGLLVVFLATQEVLRKRWTAKISMPAILIALLVLVFGGITYTDVLRYVILVGAAFAESNSGGDVVHLALMATFKIQPVFMVASFLKARWTNQENILLMLAAVFFQMAYHDARQILLWEIPDVLNSLAVAWMILRAITFTTTSNVVVPLLALLTPGLRCLNLDVYRILLLMVGIGSLIREKRSAAAKKKGASLLCLALASTGLFNPMILAAGLIACDPNRKRGWPATEVMTAVGLMFAIVGGLAELDIDSMAIPMTIAGLMFAAFVISGKSTDMWIERTADISWESDAEITGSSERVDVRLDDDGNFQLMNDPGAPWKIWMLRMVCLAISAYTPWAILPSVVGFWITLQYTKRGGVLWDTPSPKEYKKGDTTTGVYRIMTRGLLGSYQAGAGVMVEGVFHTLWHTTKGAALMSGEGRLDPYWGSVKEDRLCYGGPWKLQHKWNGQDEVQMIVVEPGKNVKNVQTKPGVFKTPEGEIGAVTLDFPTGTSGSPIVDKNGDVIGLYGNGVIMPNGSYISAIVQGERMDEPIPAGFEPEMLRKKQITVLDLHPGAGKTRRILPQIIKEAINRRLRTAVLAPTRVVAAEMAEALRGLPIRYQTSAVPREHNGNEIVDVMCHATLTHRLMSPHRVPNYNLFVMDEAHFTDPASIAARGYISTKVELGEAAAIFMTATPPGTSDPFPESNSPISDLQTEIPDRAWNSGYEWITEYTGKTVWFVPSVKMGNEIALCLQRAGKKVVQLNRKSYETEYPKCKNDDWDFVITTDISEMGANFKASRVIDSRKSVKPTIITEGEGRVILGEPSAVTAASAAQRRGRIGRNPSQVGDEYCYGGHTNEDDSNFAHWTEARIMLDNINMPNGLIAQFYQPEREKVYTMDGEYRLRGEERKNFLELLRTADLPVWLAYKVAAAGVSYHDRRWCFDGPRTNTILEDNNEVEVITKLGERKILRPRWIDARVYSDHQALKAFKDFASGKRSQIGLIEVLGKMPEHFMGKTWEALDTMYVVATAEKGGRAHRMALEELPDALQTIALIALLSVMTMGVFFLLMQRKGIGKIGLGGAVLGVATFFCWMAEVPGTKIAGMLLLSLLLMIVLIPEPEKQRSQTDNQLAVFLICVMTLVSAVAANEMGWLDKTKSDISSLFGQRIEVKENFSMGEFLLDLRPATAWSLYAVTTAVLTPLLKHLITSDYINTSLTSINVQASALFTLARGFPFVDVGVSALLLAAGCWGQVTLTVTVTAATLLFCHYAYMVPGWQAEAMRSAQRRTAAGIMKNAVVDGIVATDVPELERTTPIMQKKVGQIMLILVSLAAVVVNPSVKTVREAGILITAAAVTLWENGASSVWNATTAIGLCHIMRGGWLSCLSITWTLIKNMEKPGLKRGGAKGRTLGEVWKERLNQMTKEEFTKYRKEAIIEVDRSAAKHARKEGNVTGGHPVSRGTAKLRWLVERRFLEPVGKVIDLGCGRGGWCYYMATQKRVQEVRGYTKGGPGHEEPQLVQSYGWNIVTMKSGVDVFYRPSECCDTLLCDIGESSSSAEVEEHRTIRVLEMVEDWLHRGPREFCVKVLCPYMPKVIEKMELLQRRYGGGLVRNPLSRNSTHEMYWVSRASGNVVHSVNMTSQVLLGRMEKRTWKGPQYEEDVNLGSGTRAVGKPLLNSDTSKIKNRIERLRREYSSTWHHDENHPYRTWNYHGSYDVKPTGSASSLVNGVVRLLSKPWDTITNVTTMAMTDTTPFGQQRVFKEKVDTKAPEPPEGVKYVLNETTNWLWAFLAREKRPRMCSREEFIRKVNSNAALGAMFEEQNQWRSAREAVEDPKFWEMVDEEREAHLRGECHTCIYNMMGKREKKPGEFGKAKGSRAIWFMWLGARFLEFEALGFLNEDHWLGRKNSGGGVEGLGLQKLGYILREVGTRPGGKIYADDTAGWDTRITRADLENEAKVLELLDGEHRRLARAIIELTYRHKVVKVMRPAADGRTVMDVISREDQRGSGQVVTYALNTFTNLAVQLVRMMEGEGVIGPDDVEKLTKGKGPKVRTWLFENGEERLSRMAVSGDDCVVKPLDDRFATSLHFLNAMSKVRKDIQEWKPSTGWYDWQQVPFCSNHFTELIMKDGRTLVVPCRGQDELVGRARISPGAGWNVRDTACLAKSYAQMWLLLYFHRRDLRLMANAICSAVPVNWVPTGRTTWSIHAGGEWMTTEDMLEVWNRVWIEENEWMEDKTPVEKWSDVPYSGKREDIWCGSLIGTRARATWAENIQVAINQVRAIIGDEKYVDYMSSLKRYEDTTLVEDTVL

>ADK62442.1 polyprotein [West Nile virus]

MSKKPGGPGKSRAVNMLKRGMPRVLSLIGLKRAMLSLIDGKGPIRFVLALLAFFRFTAIAPTRAVLDRWRGVNKQTAMKHLLSFKKELGTLTSAINRRSSKQKKRGGKTGIAVMIGLIASVGAVTLSNFQGKVMMTVNATDVTDVITIPTAAGKNLCIVRAMDVGYMCDDTITYECPVLSAGNDPEDIDCWCTKSAVYVRYGRCTKTRHSRRSRRSLTVQTHGESTLANKKGAWMDSTKATRYLVKTESWILRNPGYALVAAVIGWMLGSNTMQRVVFVVLLLLVAPAYSFNCLGMSNRDFLEGVSGATWVDLVLEGDSCVTIMSKDKPTIDVKMMNMEAANLAEVRSYCYLATVSDLSTKAACPTMGEAHNDKRADPAFVCRQGVVDRGWGNGCGLFGKGSIDTCAKFACSTKAIGRTILKENIKYEVAIFVHGPTTVESHGNYSTQAGATQAGRFSITPAAPSYTLKLGEYGEVTVDCEPRSGIDTNAYYVMTVGTKTFLVHREWFMDLNLPWSSAGSTVWRNRETLMEFEEPHATKQSVIALGSQEGALHQALAGAIPVEFSSNTVKLTSGHLKCRVKMEKLQLKGTTYGVCSKAFKFLGTPADTGHGTVVLELQYTGSDGPCKVPISSVASLNDLTPVGRLVTVNPFVSVATANAKVLIELEPPFGDSYIVVGRGEQQINHHWHKSGSSIGKAFTTTLKGAQRLAALGDTAWDFGSVGGVFTSVGKAVHQVFGGAFRSLFGGMSWITQGLLGALLLWMGINARDRSIALTFLAVGGVLLFLSVNVHADTGCAIDISRQELRCGSGVFIHNDVEAWMDRYKYYPETPQGLAKIIQKAHKEGVCGLRSVSRLEHQMWEAVKDELNTLLKENGVDLSVVVEKQEGMYKSAPKRLTATTEKLEIGWKAWGKSILFAPELANNTFVVDGPETKECPTQNRAWNSLEVEDFGFGLTSTRMFLKVRESNTTECDSKIIGTAVKNNLAIHSDLSYWIESRLNDTWKLERAVLGEVKSCTWPETHTLWGDGILESDLIIPVTLAGPRSNHNRRPGYKTQNQGPWDEGRVEIDFDYCPGTTVTLSESCGHRGPATRTTTESGKLITDWCCRSCTLPPLRYQTDSGCWYGMEIRPQRHDEKTLVQSQVNAYNADMIDPFQLGLLVVFLATQEVLRKRWTAKISMPAILIALLVLVFGGITYTDVLRYVILVGAAFAESNSGGDVVHLALMATFKIQPVFMVASFLKARWTNQENILLMLAAVFFQMAYHDARQILLWEIPDVLNSLAVAWMILRAITFTTTSNVVVPLLALLTPGLRCLNLDVYRILLLMVGIGSLIREKRSAAAKKKGASLLCLALASTGLFNPMILAAGLIACDPNRKRGWPATEVMTAVGLMFAIVGGLAELDIDSMAIPMTIAGLMFAAFVISGKSTDMWIERTADISWESDAEITGSSERVDVRLDDDGNFQLMNDPGAPWKIWMLRMVCLAISAYTPWAILPSIVGFWITLQYTKRGGVLWDTPSPKEYKKGDTTTGVYRIMTRGLLGSYQAGAGVMVEGVFHTLWHTTKGAALMSGEGRLDPYWGSVKEDRLCYGGPWKLQHKWNGQDEVQMIVVEPGKNVKNVQTKPGVFKTPEGEIGAVTLDFPTGTSGSPIVDKNGDVIGLYGNGVIMPNGSYISAIVQGERMDEPIPAGFEPEMLRKKQITVLDLHPGAGKTRRILPQIIKEAINRRLRTAVLAPTRVVAAEMAEALRGLPIRYQTSAVPREHNGNEIVDVMCHATLTHRLMSPHRVPNYNLFVMDEAHFTDPASIAARGYISTKVELGEAAAIFMTATPPGTSDPFPESNSPISDLQTEIPDRAWNSGYEWITEYTGKTVWFVPSVKMGNEIALCLQRAGKKVVQLNRKSYETEYPKCKNDDWDFVITTDISEMGANFKASRVIDSRKSVKPTIITEGEGRVILGEPSAVTAASAAQRRGRIGRNPSQVGDEYCYGGHTNEDDSNFAHWTEARIMLDNINMPNGLIAQFYQPEREKVYTMDGEYRLRGEERKNFLELLRTADLPVWLAYKVAAAGVSYHDRRWCFDGPRTNTILEDNNEVEVITKLGERKILRPRWIDARVYSDHQALKAFKDFASGKRSQIGLIEVLGKMPEHFMGKTWEALDTMYVVATAEKGGRAHRMALEELPDALQTIALIALLSVMTMGVFFLLMQRKGIGKIGLGGAVLGVATFFCWMAEVPGTKIAGMLLLSLLLMIVLIPEPEKQRSQTDNQLAVFLICVMTLVSAVAANEMGWLDKTKSDISSLFGQRIEVKENFSMGEFLLDLRPATAWSLYAVTTAVLTPLLKHLITSDYINTSLTSINVQASALFTLARGFPFVDVGVSALLLAAGCWGQVTLTVTVTAATLLFCHYAYMVPGWQAEAMRSAQRRTAAGIMKNAVVDGIVATDVPELERTTPIMQKKVGQIMLILVSLAAVVVNPSVKTVREAGILITAAAVTLWENGASSVWNATTAIGLCHIMRGGWLSCLSITWTLIKNMEKPGLKRGGAKGRTLGEVWKERLNQMTKEEFTRYRKEAIIEVDRSAAKHARKEGNVTGGHPVSRGTAKLRWLVERRFLEPVGKVIDLGCGRGGWCYYMATQKRVQEVRGYTKGGPGHEEPQLVQSYGWNIVTMKSGVDVFYRPSECCDTLLCDIGESSSSAEVEEHRTIRVLEMVEDWLHRGPREFCVKVLCPYMPKVIEKMELLQRRYGGGLVRNPLSRNSTHEMYWVSRASGNVVHSVNMTSQVLLGRMEKRTWKGPQYEEDVNLGSGTRAVGKPLLNSDTSKIKNRIERLRREYSSTWHHDENHPYRTWNYHGSYDVKPTGSASSLVNGVVRLLSKPWDTITNVTTMAMTDTTPFGQQRVFKEKVDTKAPEPPEGVKYVLNETTNWLWAFLAREKRPRMCSREEFIRKVNSNAALGAMFEEQNQWRSAREAVEDPKFWEMVDEEREAHLRGECHTCIYNMMGKREKKPGEFGKAKGSRAIWFMWLGARFLEFEALGFLNEDHWLGRKNSGGGVEGLGLQKLGYILREVGTRPGGKIYADDTAGWDTRITRADLENEAKVLELLDGEHRRLARAIIELTYRHKVVKVMRPAADGRTVMDVISREDQRGSGQVVTYALNTFTNLAVQLVRMMEGEGVIGPDDVEKLTKGKGPKVRTWLFENGEERLSRMAVSGDDCVVKPLDDRFATSLHFLNAMSKVRKDIQEWKPSTGWYDWQQVPFCSNHFTELIMKDGRTLVVPCRGQDELVGRARISPGAGWNVRDTACLAKSYAQMWLLLYFHRRDLRLMANAICSAVPVNWVPTGRTTWSIHAGGEWMTTEDMLEVWNRVWIEENEWMEDKTPVEKWSDVPYSGKREDIWCGSLIGTRARATWAENIQVAINQVRAIIGDEKYVDYMSSLKRYEDTTLVEDTVL

>ANW68563.1 genome polyprotein [West Nile virus]

MSKKPGGPGKSRAVNMLKRGMPRVLSLIGLKRAMLSLIDGKGPIRFVLALLAFFRFTAIAPTRAVLDRWRGVNKQTAMKHLLSFKKELGTLTSAINRRSSKQKKRGGKTGIAVMIGLIASVGAVTLSNFQGKVMMTVNATDVTDVITIPTAAGKNLCIVRAMDVGYMCDDTITYECPVLSAGNDPEDIDCWCTKSAVYVRYGRCTKTRHSRRSRRSLTVQTHGESTLANKKGAWMDSTKATRYLVKTESWILRNPGYALVAAVIGWMLGSNTMQRVVFVVLLLLVAPAYSFNCLGMSNRDFLEGVSGATWVDLVLEGDSCVTIMSKDKPTIDVKMMNMEAANLAEVRSYCYLATVSDLSTKAACPTMGEAHNDKRADPAFVCRQGVVDRGWGNGCGLFGKGSIDTCAKFACSTKAIGRTILKENIKYEVAIFVHGPTTVESHGNYSTQVGATQAGRFSITPAAPSYTLKLGEYGEVTVDCEPRSGIDTNAYYVMTVGTKTFLVHREWFMDLNLPWSSAGSTVWRNRETLMEFEEPHATKQSVIALGSQEGALHQALAGAIPVEFSSNTVKLTSGHLKCRVKMEKLQLKGTTYGVCSKAFKFLGTPADTGHGTVVLELQYTGTDGPCKVPISSVASLNDLTPVGRLVTVNPFVSVATANAKVLIELEPPFGDSYIVVGRGEQQINHHWHKSGSSIGKAFTTTLKGAQRLAALGDTAWDFGSVGGVFTSVGKAVHQVFGGAFRSLFGGMSWITQGLLGALLLWMGINARDRSIALTFLAVGGVLLFLSVNVHADTGCAIDISRQELRCGSGVFIHNDVEAWMDRYKYYPETPQGLAKIIQKAHKEGVCGLRSVSRLEHQMWEAVKDELNTLLKENGVDLSVVVEKQEGMYKSAPKRLTATTEKLEIGWKAWGKSILFAPELANNTFVVDGPETKECPTQNRAWNSLEVEDFGFGLTSTRMFLKVRESNTTECDSKIIGTAVKNNLAIHSDLSYWIESRLNDTWKLERAVLGEVKSCTWPETHTLWGDGILESDLIIPVTLAGPRSNHNRRPGYKTQNQGPWDEGRVEIDFDYCPGTTVTLSESCGHRGPATRTTTESGKLITDWCCRSCTLPPLRYQTDSGCWYGMEIRPQRHDEKTLVQSQVNAYNADMIDPFQLGLLVVFLATQEVLRKRWTAKISMPAILIALLVLVFGGITYTDVLRYVILVGAAFAESNSGGDVVHLALMATFKIQPVFVVASFLKARWTNQENILLMLAAVFFQMAYHDARQILLWEIPDVLNSLAVAWMILRAITFTTTSNVVVPLLALLTPGLRCLNLDVYRILLLMVGIGSLIREKRSAAAKKKGASLLCLALASTGLFNPMILAAGLIACDPNRKRGWPATEVMTAVGLMFAIVGGLAELDIDSMAIPMTIAGLMFAAFVISGKSTDMWIERTADISWESDAEITGSSERVDVRLDDDGNFQLMNDPGAPWKIWMLRMVCLAISAYTPWAILPSVVGFWITLQYTKRGGVLWDTPSPKEYKKGDTTTGVYRIMTRGLLGSYQAGAGVMVEGVFHTLWHTTKGAALMSGEGRLDPYWGSVKEDRLCYGGPWKLQHKWNGQDEVQMIVVEPGKNVKNVQTKPGVFKTPEGEIGAVTLDFPTGTSGSPIVDKNGDVIGLYGNGVIMPNGSYISAIVQGERMDEPIPAGFEPEMLRKKQITVLDLHPGAGKTRRILPQIIKEAINRRLRTAVLAPTRVVAAEMAEALRGLPIRYQTSAVPREHNGNEIVDVMCHATLTHRLMSPHRVPNYNLFVMDEAHFTDPASIAARGYISTKVELGEAAAIFMTATPPGTSDPFPESNSPISDLQTEIPDRAWNSGYEWITEYTGKTVWFVPSVKMGNEIALCLQRAGKKVVQLNRKSYETEYPKCKNDDWDFVITTDISEMGANFKASRVIDSRKSVKPTIITEGEGRVILGEPSAVTAASAAQRRGRIGRNPSQVGDEYCYGGHTNEDDSNFAHWTEARIMLDNINMPNGLIAQFYQPEREKVYTMDGEYRLRGEERKNFLELLRTADLPVWLAYKVAAAGVSYHDRRWCFDGPRTNTILEDNNEVEVITKLGERKILRPRWIDARVYSDHQALKAFKDFASGKRSQIGLIEVLGKMPEHFMGKTWEALDTMYVVATAEKGGRAHRMALEELPDALQTIALIALLSVMTMGVFFLLMQRKGIGKIGLGGAVLGVATFFCWMAEVPGTKIAGMLLLSLLLMIVLIPEPEKQRSQTDNQLAVFLICVMTLVSAVAANEMGWLDKTKSDISSLFGQRIEVKENFSMGEFLLDLRPATAWSLYAVTTAVLTPLLKHLIASDYINTSLTSINVQASALFTLARGFPFVDVGVSALLLAAGCWGQVTLTVTVTAATLLFCHYAYMVPGWQAEAMRSAQRRTAAGIMKNAVVDGIVATDVPELERTTPIMQKKVGQIMLILVSLAAVVVNPSVKTVREAGILITAAAVTLWENGASSVWNATTAIGLCHIMRGGWLSCLSITWTLIKNMEKPGLKRGGAKGRTLGEVWKERLNQMTKEEFTRYRKEAIIEVDRSAAKHARKEGNVTGGHPVSRGTAKLRWLVERRFLEPVGKVIDLGCGRGGWCYYMATQKRVQEVRGYTKGGPGHEEPQLVQSYGWNIVTMKSGVDVFYRPSECCDTLLCDIGESSSSAEVEEHRTIRVLEMVEDWLHRGPREFCVKVLCPYMPKVIEKMELLQRRYGGGLVRNPLSRNSTHEMYWVSRASGNVVHSVNMTSQVLLGRMEKRTWKGPQYEEDVNLGSGTRAVGKPLLNSDTSKIKNRIERLRREYSSTWHHDENHPYRTWNYHGSYDVKPTGSASSLVNGVVRLLSKPWDTITNVTTMAMTDTTPFGQQRVFKEKVDTKAPEPPEGVKYVLNETTNWLWAFLAREKRPRMCSREEFIRKVNSNAALGAMFEEQNQWRSAREAVEDPKFWEMVDEEREAHLRGECHTCIYNMMGKREKKPGEFGKAKGSRAIWFMWLGARFLEFEALGFLNEDHWLGRKNSGGGVEGLGLQKLGYILREVGTRPGGKIYADDTAGWDTRITRADLENEAKVLELLDGEHRRLARAIIELTYRHKVVKVMRPAADGRTVMDVISREDQRGSGQVVTYALNTFTNLAVQLVRMMEGEGVIGPDDVEKLTKGKGPKVRTWLFENGEERLSRMAVSGDDCVVKPLDDRFATSLHFLNAMSKVRKDIQEWKPSTGWYDWQQVPFCSNHFTELIMKDGRTLVVPCRGQDELVGRARISPGAGWNVRDTACLAKSYAQMWLLLYFHRRDLRLMANAICSAVPVNWVPTGRTTWSIHAGGEWMTTEDMLEVWNRVWIEENEWMEDKTPVEKWSDVPYSGKREDIWCGSLIGTRARATWAENIQVAINQVRAIIGDEKYVDYMSSLKRYEDTTLVEDTVL

>ADL27959.1 polyprotein [West Nile virus]

MSKKPGGPGKSRAVNMLKRGMPRVLSLIGLKRAMLSLIDGKGPTRFVLALLAFFRFTAIAPTRAVLDRWRGVNKQTAMKHLLSFKKELGTLTSAINRRSSKQKKRGGKTGIAVMIGLIASVGAVTLSNFQGKVMMTVNATDVTDVITIPTAAGKNLCIVRAMDVGYMCDDTITYECPVLSAGNDPEDIDCWCTKSAVYVRYGRCTKTRHSRRSRRSLTVQTHGESTLANKKGAWMDSTKATRYLVKTESWILRNPGYALVAAVIGWMLGSNTMQRVVFVVLLLLVAPAYSFNCLGMSNRDFLEGVSGATWVDLVLEGDSCVTIMSKDKPTIDVKMMNMEAANLAEVRSYCYLATVSDLSTKAACPTMGEAHNDKRADPAFVCRQGVVDRGWGNGCGLFGKGSIDTCAKFACSTKAIGRTILKENIKYEVAIFVHGPTTVESHGNYSTQAGATQAGRFSITPAAPSYTLKLGEYGEVTVDCEPRSGIDTNAYYVMTVGTKTFLVHREWFMDLNLPWSSAGSTVWRNRETLMEFEEPHATKQSVIALGSQEGALHQALAGAIPVEFSSNTVKLTSGHLKCRVKMEKLQLKGTTYGVCSKAFKFLGTPADTGHGTVVLELQYTGTDGPCKVPISSVASLNDLTPVGRLVTVNPFVSVATANAKVLIELEPPFGDSYIVVGRGEQQINHHWHKSGSSIGKAFTTTLKGAQRLAALGDTAWDFGSVGGVFTSVGKAVHQVFGGAFRSLFGGMSWITQGLLGALLLWMGINARDRSIALTFLAVGGVLLFLSVNVHADTGCAIDISRQELRCGSGVFIHNDVEAWMDRYKYYPETPQGLAKIIQKAHKEGVCGLRSVSRLEHQMWEAVKDELNTLLKENGVDLSVVVEKQEGMYKSAPKRLTATTEKLEIGWKAWGKSILFAPELANNTFVVDGPETKECPTQNRAWNSLEVEDFGFGLTSTRMFLKVRESNTTECDSKIIGTAVKNNLAIHSDLSYWIESRLNDTWKLERAVLGEVKSCTWPETHTLWGDGILESDLIIPVTLAGPRSNHNRRPGYKTQNQGPWDEGRVEIDFDYCPGTTVTLSESCGHRGPATRTTTESGKLITDWCCRSCTLPPLRYQTDSGCWYGMEIRPQRHDEKTLVQSQVNAYNADMIDPFQLGLLVVFLATQEVLRKRWTAKISMPAILIALLVLVFGGITYTDVLRYVILVGAAFAESNSGGDVVHLALMATFKIQPVFMVASFLKARWTNQENILLMLAAVFFQMAYHDARQILLWEIPDVLNSLAVAWMILRAITFTTTSNVVVPLLALLTPGLRCLNLDVYRILLLMVGIGSLIREKRSAAAKKKGASLLCLALASTGLFNPMILAAGLIACDPNRKRGWPATEVMTAVGLMFAIVGGLAELDIDSMAIPMTIAGLMFAAFVISGKSTDMWIERTADISWESDAEITGSSERVDVRLDDDGNFQLMNDPGAPWKIWMLRMVCLAISAYTPWAILPSVVGFWITLQYTKRGGVLWDTPSPKEYKKGDTTTGVYRIMTRGLLGSYQAGAGVMVEGVFHTLWHTTKGAALMSGEGRLDPYWGSVKEDRLCYGGPWKLQHKWNGQDEVQMIVVEPGKNVKNVQTKPGVFKTPEGEIGAVTLDFPTGTSGSPIVDKNGDVIGLYGNGVIMPNGSYISAIVQGERMDEPIPAGFEPEMLRKKQITVLDLHPGAGKTRRILPQIIKEAINRRLRTAVLAPTRVVAAEMAEALRGLPIRYQTSAVPREHNGNEIVDVMCHATLTHRLMSPHRVPNYNLFVMDEAHFTDPASIAARGYISTKVELGEAAAIFMTATPPGTSDPFPESNSPISDLQTEIPDRAWNSGYEWITEYTGKTVWFVPSVKMGNEIALCLQRAGKKVVQLNRKSYETEYPKCKNDDWDFVITTDISEMGANFKASRVIDSRKSVKPTIITEGEGRVILGEPSAVTAASAAQRRGRIGRNPSQVGDEYCYGGHTNEDDSNFAHWTEARIMLDNINMPNGLIAQFYQPEREKVYTMDGEYRLRGEERKNFLELLRTADLPVWLAYKVAAAGVSYHDRRWCFDGPRTNTILEDNNEVEVITKLGERKILRPRWIDARVYSDHQALKAFKDFASGKRSQIGLIEVLGKMPEHFMGKTWEALDTMYVVATAEKGGRAHRMALEELPDALQTIALIALLSVMTMGVFFLLMQRKGIGKIGLGGAVLGVATFFCWMAEVPGTKIAGMLLLSLLLMIVLIPEPEKQRSQTDNQLAVFLICVMTLVSAVAANEMGWLDKTKSDISSLFGQRIEVKENFSMGEFLLDLRPATAWSLYAVTTAVLTPLLKHLITSDYINTSLTSINVQASALFTLARGFPFVDVGVSALLLAAGCWGQVTLTVTVTAATLLFCHYAYMVPGWQAEAMRSAQRRTAAGIMKNAVVDGIVATDVPELERTTPIMQKKVGQIMLILVSLAAVVVNPSVKTVREAGILITAAAVTLWENGASSVWNATTAIGLCHIMRGGWLSCLSITWTLIKNMEKPGLKRGGAKGRTLGEVWKERLNQMTKEEFTRYRKEAIIEVDRSAAKHARKEGNVTGGHPVSRGTAKLRWLVERRFLEPVGKVIDLGCGRGGWCYYMATQKRVQEVRGYTKGGPGHEEPQLVQSYGWNIVTMKSGVDVFYRPSECCDTLLCDIGESSSSAEVEEHRTIRVLEMVEDWLHRGPREFCVKVLCPYMPKVIEKMELLQRRYGGGLVRNPLSRNSTHEMYWVSRASGNVVHSVNMTSQVLLGRMEKRTWKGPQYEEDVNLGSGTRAVGKPLLNSDTSKIKNRIERLRREYSSTWHHDENHPYRTWNYHGSYDVKPTGSASSLVNGVVRLLSKPWDTITNVTTMAMTDTTPFGQQRVFKEKVDTKAPEPPEGVKYVLNETTNWLWAFLAREKRPRMCSREEFIRKVNSNAALGAMFEEQNQWRSAREAVEDPKFWEMVDEEREAHLRGECHTCIYNMMGKREKKPGEFGKAKGSRAIWFMWLGARFLEFEALGFLNEDHWLGRKNSGGGVEGLGLQKLGYILREVGTRPGGKIYADDTAGWDTRITRADLENEAKVLELLDGEHRRLARAIIELTYRHKVVKVMRPAADGRTVMDVISREDQRGSGQVVTYALNTFTNLAVQLVRMMEGEGVIGPDDVEKLTKGKGPKVRTWLFENGEERLSRMAVSGDDCVVKPLDDRFATSLHFLNAMSKVRKDIQEWKPSTGWYDWQQVPFCSNHFTELIMKDGRTLVVPCRGQDELVGRARISPGAGWNVRDTACLAKSYAQMWLLLYFHRRDLRLMANAICSAVPVNWVPTGRTTWSIHAGGEWMTTEDMLEVWNRVWIEENEWMEDKTPVEKWSDVPYSGKREDIWCGSLIGTRARATWAENIQVAINQVRAIIGDEKYVDYMSSLKRYEDTTLVEDTVL

>ANW68770.1 genome polyprotein [West Nile virus]

MSKKPGGPGKSRAVNMLKRGMPRVLSLIGLKRAMLSLIDGKGPIRFVLALLAFFRFTAIAPTRAVLDRWRGVNKQTAMKHLLSFKKELGTLTSAINRRSSKQKKRGGKTGIAVMIGLIASVGAVTLSNFQGKVMMTVNATDVTDVITIPTAAGKNLCIVRAMDVGYMCDDTITYECPVLSAGNDPEDIDCWCTKSAVYVRYGRCTKTRHSRRSRRSLTVQTHGESTLANKKGAWMDSTKATRYLVKTESWILRNPGYALVAAVIGWMLGSNTMQRVVFVVLLLLVAPAYSFNCLGMSNRDFLEGVSGATWVDLVLEGDSCVTIMSKDKPTIDVKMMNMEAANLAEVRSYCYLATVSDLSTKAACPTMGEAHNDKRADPAFVCRQGVVDRGWGNGCGLFGKGSIDTCAKFACSTKAIGRTILKENIKYEVAIFVHGPTTVESHGNYSTQAGATQAGRFSITPAAPSYTLKLGEYGEVTVDCEPRSGIDTNAYYVMTVGTKTFLVHREWFMDLNLPWSSAGSTVWRNRETLMEFEEPHATKQSVIALGSQEGALHQALAGAIPVEFSSNTVKLTSGHLKCRVKMEKLQLKGTTYGVCSKAFKFLGTPADTGHGTVVLELQYTGTDGPCKVPISSVASLNDLTPVGRLVTVNPFVSVATANAKVLIELEPPFGDSYIVVGRGEQQINHHWHKSGSSIGKAFTTTLKGAQRLAALGDTAWDFGSVGGVFTSVGKAVHQVFGGAFRSLFGGMSWITQGLLGALLLWMGINARDRSIALTFLAVGGVLLFLSVNVHADTGCAIDISRQELRCGSGVFIHNDVEAWMDRYKYYPETPQGLAKIIQKAHKEGVCGLRSVSRLEHQMWEAVKDELNTLLKENGVDLSVVVEKQEGMYKSAPKRLTATTEKLEIGWKAWGKSILFAPELANNTFVVDGPETKECPTQNRAWNSLEVEDFGFGLTSTRMFLKVRESNTTECDSKIIGTAVKNNLAIHSDLSYWIESRLNDTWKLERAVLGEVKSCTWPETHTLWGDGILESDLIIPVTLAGPRSNHNRRPGYKTQNQGPWDEGRVEIDFDYCPGTTVTLSESCGHRGPATRTTTESGKLITDWCCRSCTLPPLRYQTDSGCWYGMEIRPQRHDEKTLVQSQVNAYNADMIDPFQLGLLVVFLATQEVLRKRWTAKISMPAILIALLVLVFGGITYTDVLRYVILVGAAFAESNSGGDVVHLALMATFKIQPVFMVASFLKARWTNQENILLMLAAVFFQMAYHDARQILLWEIPDVLNSLAVAWMILRAITFTTTSNVVVPLLALLTPGLRCLNLDVYRILLLMVGIGSLIREKRSAAAKKKGASLLCLALASTGLFNPMILAAGLIACDPNRKRGWPATEVMTAVGLMFAIVGGLAELDIDSMAIPMTIAGLMFAAFVISGKSTDMWIERTADISWESDAEITGSSERVDVRLDDDGNFQLMNDPGAPWKIWMLRMVCLAISAYTPWAILPSVVGFWITLQYTKRGGVLWDTPSPKEYKKGDTTTGVYRIMTRGLLGSYQAGAGVMVEGVFHTLWHTTKGAALMSGEGRLDPYWGSVKEDRLCYGGPWKLQHKWNGQDEVQMIVVEPGKNVKNVQTKPGVFKTPEGEIGAVTLDFPTGTSGSPIVDKNGDVIGLYGNGVIMPNGSYISAIVQGERMDEPIPAGFEPEMLRKKQITVLDLHPGAGKTRRILPQIIKEAINRRLRTAVLAPTRVVAAEMAEALRGLPIRYQTSAVPREHNGNEIVDVMCHATLTHRLMSPHRVPNYNLFVMDEAHFTDPASIAARGYISTKVELGEAAAIFMTATPPGTSDPFPESNSPISDLQTEIPDRAWNSGYEWITEYTGKTVWFVPSVKMGNEIALCLQRAGKKVVQLNRKSYETEYPKCKNDDWDFVITTDISEMGANFKASRVIDSRKSVKPTIITEGEGRVILGEPSAVTAASAAQRRGRIGRNPSQVGDEYCYGGHTNEDDSNFAHWTEARIMLDNINMPNGLIAQFYQPEREKVYTMDGEYRLRGEERKNFLELLRTADLPVWLAYKVAAAGVSYHDRRWCFDGPRTNTILEDNNEVEVITKLGERKILRPRWIDARVYSDHQALKAFKDFASGKRSQIGLIEVLGKMPEHFMGKTWEALDTMYVVATAEKGGRAHRMALEELPDALQTIALIALLSVMTMGVFFLLMQRKGIGKIGLGGAVLGVATFFCWMAEVPGTKIAGMLLLSLLLMIVLIPEPEKQRSQTDNQLAVFLICVMTLVSAVAANEMGWLDKTKSDISSLFGQRIEVKESFSMGEFLLDLRPATAWSLYAVTTAVLTPLLKHLITSDYINTSLTSINVQASALFTLARGFPFVDVGVSALLLAAGCWGQVTLTVTVTAATLLFCHYAYMVPGWQAEAMRSAQRRTAAGIMKNAVVDGIVATDVPELERTTPIMQKKVGQIMLILVSLAAVVVNPSVKTVREAGILITAAAVTLWENGASSVWNATTAIGLCHIMRGGWLSCLSITWTLIKNMEKPGLKRGGAKGRTLGEVWKERLNQMTKEEFTRYRKEAIIEVDRSAAKHARKEGNVTGGHPVSRGTAKLRWLVERRFLEPVGKVIDLGCGRGGWCYYMATQKRVQEVRGYTKGGPGHEEPQLVQSYGWNIVTMKSGVDVFYRPSECCDTLLCDIGESSSSAEVEEHRTIRVLEMVEDWLHRGPREFCVKVLCPYMPKVIEKMELLQRRYGGGLVRNPLSRNSTHEMYWVSRASGNVVHSVNMTSQVLLGRMEKRTWKGPQYEEDVNLGSGTRAVGKPLLNSDTSKIKNRIERLRREYSSTWHHDENHPYRTWNYHGSYDVKPTGSASSLVNGVVRLLSKPWDTITNVTTMAMTDTTPFGQQRVFKEKVDTKAPEPPEGVKYVLNETTNWLWAFLAREKRPRMCSREEFIRKVNSNAALGAMFEEQNQWRSAREAVEDPKFWEMVDEEREAHLRGECHTCIYNMMGKREKKPGEFGKAKGSRAIWFMWLGARFLEFEALGFLNEDHWLGRKNSGGGVEGLGLQKLGYILREVGTRPGGKIYADDTAGWDTRITRADLENEAKVLELLDGEHRRLARAIIELTYRHKVVKVMRPAADGRTVMDVISREDQRGSGQVVTYALNTFTNLAVQLVRMMEGEGVIGPDDVEKLTKGKGPKVRTWLFENGEERLSRMAVSGDDCVVKPLDDRFATSLHFLNAMSKVRKDIQEWKPSTGWYDWQQVPFCSNHFTELIMKDGRTLVVPCRGQDELVGRARISPGAGWNVRDTACLAKSYAQMWLLLYFHRRDLRLMANAICSAVPVNWVPTGRTTWSIHAGGEWMTTEDMLEVWNRVWIEENEWMEDKTPVEKWSDVPYSGKREDIWCGSLIGTRARATWAENIQVAINQVRAIIGDEKYVDYMSSLKRYEDTTLVEDTVL

>AEZ56192.1 polyprotein [West Nile virus]

MSKKPGGPGKSRAVNMLKRGMPRVLSLIGLKRAMLSLIDGKGPIRFVLALLAFFRFTAIAPTRAVLDRWRGVNKQTAMKHLLSFKKELGTLTSAINRRSSKQKKRGGKTGIAVMIGLIASVGAVTLSNFQGKVMMTVNATDVTDVITIPTAAGKNLCIVRAMDVGYMCDDTITYECPVLSAGNDPEDIDCWCTKSAVYVRYGRCTKTRHSRRSRRSLTVQTHGESTLANKKGAWMDSTKATRYLVKTESWILRNPGYALVAAVIGWMLGSNTMQRVVFVVLLLLVAPAYSFNCLGMSNRDFLEGVSGATWVDLVLEGDSCVTIMSKDKPTIDVKMMNMEAANLAEVRSYCYLATVSDLSTKAACPTMGEAHNDKRADPAFVCRQGVVDRGWGNGCGLFGKGSIDTCAKFACSTKAIGRTILKENIKYEVAIFVHGPTTVESHGNYSTQAGATQAGRFSITPAAPSYTLKLGEYGEVTVDCEPRSGIDTNAYYVMTVGTKTFLVHREWFMDLNLPWSSAGSTVWRNRETLMEFEEPHATKQSVIALGSQEGALHQALAGAIPVEFSSNTVKLTSGHLKCRVKMEKLQLKGTTYGVCSKAFKFLGTPADTGHGTVVLELQYTGTDGPCKVPISSVASLNDLTPVGRLVTVNPFVSVATANAKVLIELEPPFGDSYIVVGRGEQQINHHWHKSGSSIGKAFTTTLKGAQRLAALGDTAWDFGSVGGVFTSVGKAVHQVFGGAFRSLFGGMSWITQGLLGALLLWMGINARDRSIALTFLAVGGVLLFLSVNVHADTGCAIDISRQELRCGSGVFIHNDVEAWMDRYKYYPETPQGLAKIIQKAHKEGVCGLRSVSRLEHQMWEAVKDELNTLLKENGVDLSVVVEKQEGMYKSAPKRLTATTEKLEIGWKAWGKSILFAPELANNTFVVDGPETKECPTQNRAWNSLEVEDFGFGLTSTRMFLKVRESNTTECDSKIIGTAVKNNLAIHSDLSYWIESRLNDTWKLERAVLGEVKSCTWPETHTLWGDGILESDLIIPVTLAGPRSNHNRRPGYKTQNQGPWDEGRVEIDFDYCPGTTVTLSESCGHRGPATRTTTESGKLITDWCCRSCTLPPLRYQTDSGCWYGMEIRPQRHDEKTLVQSQVNAYNADMIDPFQLGLLVVFLATQEVLRKRWTAKISMPAILIALLVLVFGGITYTDVLRYVILVGAAFAESNSGGDVVHLALMATFKIQPVFMVASFLKARWTNQENILLMLAAVFFQMAYHDARQILLWEIPDVLNSLAVAWMILRAITFTTTSNVVVPLLALLTPGLRCLNLDVYRILLLMVGIGSLIREKRSAAAKKKGASLLCLALASTGLFNPMILAAGLIACDPNRKRGWPATEVMTAVGLMFAIVGGLAELDIDSMAIPMTIAGLMFAAFVISGKSTDMWIERTADISWESDAEITGSSERVDVRLDDDGNFQLMNDPGAPWKIWMLRMVCLAISAYTPWAILPSVVGFWITLQYTKRGGVLWDTPSPKEYKKGDTTTGVYRIMTRGLLGSYQAGAGVMVEGVFHTLWHTTKGAALMSGEGRLDPYWGSVKEDRLCYGGPWKLQHKWNGQDEVQMIVVEPGKNVKNVQTKPGVFKTPEGEIGAVTLDFPTGTSGSPIVDKNGDVIGLYGNGVIMPNGSYISAIVQGERMDEPIPAGFEPEMLRKKQITVLDLHPGAGKTRRILPQIIKEAINRRLRTAVLAPTRVVAAEMAEALRGLPIRYQTSAVPREHNGNEIVDVMCHATLTHRLMSPHRVPNYNLFVMDEAHFTDPASIAARGYISTKVELGEAAAIFMTATPPGTSDPFPESNSPISDLQTEIPDRAWNSGYEWITEYTGKTVWFVPSVKMGNEIALCLQRAGKKVVQLNRKSYETEYPKCKNDDWDFVITTDISEMGANFKASRVIDSRKSVKPTIITEGEGRVILGEPSAVTAASAAQRRGRIGRNPSQVGDEYCYGGHTNEDDSNFAHWTEARIMLDNINMPNGLIAQFYQPEREKVYTMDGEYRLRGEERKNFLELLRTADLPVWLAYKVAAAGVSYHDRRWCFDGPRTNTILEDNNEVEVITKLGERKILRPRWIDARVYSDHQALKAFKDFASGKRSQIGLIEVLGKMPEHFMGKTWEALDTMYVVATAEKGGRAHRMALEELPDALQTIALIALLSVMTMGVFFLLMQRKGIGKIGLGGAVLGVATFFCWMAEVPGTKIAGMLLLSLLLMIVLIPEPEKQRSQTDNQLAVFLICVMTLVSAVAANEMGWLDKTKSDISSLFGQRIEVKENFSMGEFLLDLRPATAWSLYAVTTAVLTPLLKHLITSDYINTSLTSINVQASALFTLARGFPFVNVGVSALLLAAGCWGQVTLTVTVTAATLLFCHYAYMVPGWQAEAMRSAQRRTAAGIMKNAVVDGIVATDVPELERTTPIMQKKVGQIMLILVSLAAVVVNPSVKTVREAGILITAAAVTLWENGASSVWNATTAIGLCHIMRGGWLSCLSITWTLIKNMEKPGLKRGGAKGRTLGEVWKERLNQMTKEEFTRYRKEAIIEVDRSAAKHARKEGNVTGGHPVSRGTAKLRWLVERRFLEPVGKVIDLGCGRGGWCYYMATQKRVQEVRGYTKGGPGHEEPQLVQSYGWNIVTMKSGVDVFYRPSECCDTLLCDIGESSSSAEVEEHRTIRVLEMVEDWLHRGPREFCVKVLCPYMPKVIEKMELLQRRYGGGLVRNPLSRNSTHEMYWVSRASGNVVHSVNMTSQVLLGRMEKRTWKGPQYEEDVNLGSGTRAVGKPLLNSDTSKIKNRIERLRREYSSTWHHDENHPYRTWNYHGSYDVKPTGSASSLVNGVVRLLSKPWDTITNVTTMAMTDTTPFGQQRVFKEKVDTKAPEPPEGVKYVLNETTNWLWAFLAREKRPRMCSREEFIRKVNSNAALGAMFEEQNQWRSAREAVEDPKFWEMVDEEREAHLRGECHTCIYNMMGKREKKPGEFGKAKGSRAIWFMWLGARFLEFEALGFLNEDHWLGRKNSGGGVEGLGLQKLGYILREVGTRPGGKIYADDTAGWDTRITRADLENEAKVLELLDGEHRRLARAIIELTYRHKVVKVMRPAADGRTVMDVISREDQRGSGQVVTYALNTFTNLAVQLVRMMEGEGVIGPDDVEKLTKGKGPKVRTWLFENGEERLSRMAVSGDDCVVKPLDDRFATSLHFLNAMSKVRKDIQEWKPSTGWYDWQQVPFCSNHFTELIMKDGRTLVVPCRGQDELVGRARISPGAGWNVRDTACLAKSYAQMWLLLYFHRRDLRLMANAICSAVPVNWVPTGRTTWSIHAGGEWMTTEDMLEVWNRVWIEENEWMEDKTPVEKWSDVPYSGKREDIWCGSLIGTRARATWAENIQVAINQVRAIIGDEKYVDYMSSLKRYEDTTLVEDTVL

>ADK62419.1 polyprotein [West Nile virus]

MSKKPGGPGKSRAVNMLKRGMPRVLSLIGLKRAMLSLIDGKGPIRFVLALLAFFRFTAIAPTRAVLDRWRGVNKQTAMKHLLSFKKELGTLTSAINRRSSKQKKRGGKTGIAVMIGLIASVGAVTLSNFQGKVMMTVNATDVTDVITIPTAAGKNLCTVRAMDVGYMCDDTITYECPVLSAGNDPEDIDCWCTKSAVYVRYGRCTKTRHSRRSRRSLTVQTHGESTLANKKGAWMDSTKATRYLVKTESWILRNPGYALVAAVIGWMLGSNTMQRVVFVVLLLLVAPAYSFNCLGMSNRDFLEGVSGATWVDLVLEGDSCVTIMSKDKPTIDVKMMNMEAANLAEVRSYCYLATVSDLSTKAACPTMGEAHNDKRADPAFVCRQGVVDRGWGNGCGLFGKGSIDTCAKFACSTKAIGRTILKENIKYEVAIFVHGPTTVESHGNYSTQAGATQAGRFSITPAAPSYTLKLGEYGEVTVDCEPRSGIDTNAYYVMTVGTKTFLVHREWFMDLNLPWSSAGSTVWRNRETLMEFEEPHATKQSVIALGSQEGALHQALAGAIPVEFSSNTVKLTSGHLKCRVKMEKLQLKGTTYGVCSKAFKFLGTPADTGHGTVVLELQYTGTDGPCKVPISSVASLNDLTPVGRLVTVNPFVSVATANAKVLIELEPPFGDSYIVVGRGEQQINHHWHKSGSSIGKAFTTTLKGAQRLAALGDTAWDFGSVGGVFTSVGKAVHQVFGGAFRSLFGGMSWITQGLLGALLLWMGINARDRSIALTFLAVGGVLLFLSVNVHADTGCAIDISRQELRCGSGVFIHNDVEAWMDRYKYYPETPQGLAKIIQKAHKEGVCGLRSVSRLEHQMWEAVKDELNTLLKENGVDLSVVVEKQEGMYKSAPKRLTATTEKLEIGWKAWGKSILFAPELANNTFVVDGPETKECPTQNRAWNSLEVEDFGFGLTSTRMFLKVRESNTTECDSKIIGTAVKNNLAIHSDLSYWIESRLNDTWKLERAVLGEVKSCTWPETHTLWGDGILESDLIIPVTLAGPRSNHNRRPGYKTQNQGPWDEGRVEIDFDYCPGTTVTLSESCGHRGPATRTTTESGKLITDWCCRSCTLPPLRYQTDSGCWYGMEIRPQRHDEKTLVQSQVNAYNADMIDPFQLGLLVVFLATQEVLRKRWTAKISMPAILIALLVLVFGGITYTDVLRYVILVGAAFAESNSGGDVVHLALMATFKIQPVFMVASFLKARWTNQENILLMLAAVFFQMAYHDARQILLWEIPDVLNSLAVAWMILRAITFTTTSNVVVPLLALLTPGLRCLNLDVYRILLLMVGIGSLIREKRSAAAKKKGASLLCLALASTGLFNPMILAAGLIACDPNRKRGWPATEVMTAVGLMFAIVGGLAELDIDSMAIPMTIAGLMFAAFVISGKSTDMWIERTADISWESDAEITGSSERVDVRLDDDGNFQLMNDPGAPWKIWMLRMVCLAISAYTPWAILPSVVGFWITLQYTKRGGVLWDTPSPKEYKKGDTTTGVYRIMTRGLLGSYQAGAGVMVEGVFHTLWHTTKGAALMSGEGRLDPYWGSVKEDRLCYGGPWKLQHKWNGQDEVQMIVVEPGKNVKNVQTKPGVFKTPEGEIGAVTLDFPTGTSGSPIVDKNGDVIGLYGNGVIMPNGSYISAIVQGERMDEPIPAGFEPEMLRKKQITVLDLHPGAGKTRRILPQIIKEAINRRLRTAVLAPTRVVAAEMAEALRGLPIRYQTSAVPREHNGNEIVDVMCHATLTHRLMSPHRVPNYNLFVMDEAHFTDPASIAARGYISTKVELGEAAAIFMTATPPGTSDPFPESNSPISDLQTEIPDRAWNSGYEWITEYTGKTVWFVPSVKMGNEIALCLQRAGKKVVQLNRKSYETEYPKCKNDDWDFVITTDISEMGANFKASRVIDSRKSVKPTIITEGEGRVILGEPSAVTAASAAQRRGRIGRNPSQVGDEYCYGGHTNEDDSNFAHWTEARIMLDNINMPNGLIAQFYQPEREKVYTMDGEYRLRGEERKNFLELLRTADLPVWLAYKVAAAGVSYHDRRWCFDGPRTNTILEDNNEVEVITKLGERKILRPRWIDARVYSDHQALKAFKDFASGKRSQIGLIEVLGKMPEHFMGKTWEALDTMYVVATAEKGGRAHRMALEELPDALQTIALIALLSVMTMGVFFLLMQRKGIGKIGLGGAVLGVATFFCWMAEVPGTKIAGMLLLSLLLMIVLIPEPEKQRSQTDNQLAVFLICVMTLVSAVAANEMGWLDKTKSDISSLFGQRIEVKENFSMGEFLLDLRPATAWSLYAVTTAVLTPLLKHLITSDYINTSLTSINVQASALFTLARGFPFVDVGVSALLLAAGCWGQVTLTVTVTAATLLFCHYAYMVPGWQAEAMRSAQRRTAAGIMKNAVVDGIVATDVPELERTTPIMQKKVGQIMLILVSLAAVVVNPSVKTVREAGILITAAAVTLWENGASSVWNATTAIGLCHIMRGGWLSCLSITWTLIKNMEKPGLKRGGAKGRTLGEVWKERLNQMTKEEFTRYRKEAIIEVDRSAAKHARKEGNVTGGHPVSRGTAKLRWLVERRFLEPVGKVIDLGCGRGGWCYYMATQKRVQEVRGYTKGGPGHEEPQLVQSYGWNIVTMKSGVDVFYRPSECCDTLLCDIGESSSSAEVEEHRTIRVLEMVEDWLHRGPREFCVKVLCPYMPKVIEKMELLQRRYGGGLVRNPLSRNSTHEMYWVSRASGNVVHSVNMTSQVLLGRMEKRTWKGPQYEEDVNLGSGTRAVGKPLLNSDTSKIKNRIERLRREYSSTWHHDENHPYRTWNYHGSYDVKPTGSASSLVNGVVRLLSKPWDTITNVTTMAMTDTTPFGQQRVFKEKVDTKAPEPPEGVKYVLNETTNWLWAFLAREKRPRMCSREEFIRKVNSNAALGAMFEEQNQWRSAREAVEDPKFWEMVDEEREAHLRGECHTCIYNMMGKREKKPGEFGKAKGSRAIWFMWLGARFLEFEALGFLNEDHWLGRKNSGGGVEGLGLQKLGYILREVGTRPGGKIYADDTAGWDTRITRADLENEAKVLELLDGEHRRLARAIIELTYRHKVVKVMRPAADGRTVMDVISREDQRGSGQVVTYALNTFTNLAVQLVRMMEGEGVIGPDDVEKLTKGKGPKVRTWLFENGEERLSRMAVSGDDCVVKPLDDRFATSLHFLNAMSKVRKDIQEWKPSTGWYDWQQVPFCSNHFTELIMKDGRTLVVPCRGQDELVGRARISPGAGWNVRDTACLAKSYAQMWLLLYFHRRDLRLMANAICSAVPVNWVPTGRTTWSIHAGGEWMTTEDMLEVWNRVWIEENEWMEDKTPVEKWSDVPYSGKREDIWCGSLIGTRARATWAENIQVAINQVRAIIGDEKYVDYMSSLKRYEDTTLVEDTVL

>ADK62459.1 polyprotein [West Nile virus]

MSKKPGGPGKSRAVNMLKRGMPRVLSLIGLKRAMLSLIDGKGPIRFVLALLAFFRFTAIAPTRAVLDRWRGVNKQTAMKHLLSFKKELGTLTSAINRRSSKQKKRGGKTGIAVMIGLIASVGAVTLSNFQGKVMMTVNATDVTDVITIPTAAGKNLCIVRAMDVGYMCDDTITYECPVLSAGNDPEDIDCWCTKSAVYVRYGRCTKTRHSRRSRRSLTVQTHGESTLANKKGAWMDSTKATRYLVKTESWILRNPGYALVAAVIGWMLGSNTMQRVVFVVLLLLVAPAYSFNCLGMSNRDFLEGVSGATWVDLVLEGDSCVTIMSKDKPTIDVKMMNMEAANLAEVRSYCYLATVSDLSTKAACPTMGEAHNDKRADPAFVCRQGVVDRGWGNGCGLFGKGSIDTCAKFACSTKAIGRTILKENIKYEVAIFVHGPTTVESHGNYSTQAGATQAGRFSITPAAPSYTLKLGEYGEVTVDCEPRSGIDTNAYYVMTVGTKTFLVHREWFMDLNLPWSSAGSTVWRNRETLMEFEEPHATKQSVIALGSQEGALHQALAGAIPVEFSSNTVKLTSGHLKCRVKMEKLQLKGTTYGVCSKAFKFLGTPADTGHGTVVLELQYTGTDGPCKVPISSVASLNDLTPVGRLVTVNPFVSVATANAKVLIELEPPFGDSYIVVGRGEQQINHHWHKSGSSIGKAFTTTLKGAQRLAALGDTAWDFGSVGGVFTSVGKAVHQVFGGAFRSLFGGMSWITQGLLGALLLWMGINARDRSIALTFLAVGGVLLFLSVNVHADTGCAIDISRQELRCGSGVFIHNDVEAWMDRYKYYPETPQGLAKIIQKAHKEGVCGLRSVSRLEHQMWEAVKDELNTLLKENGVDLSVVVEKQEGMYKSAPKRLTATTEKLEIGWKAWGKSILFAPELANNTFVVDGPETKECPTQNRAWNSLEVEDFGFGLTSTRMFLKVRESNTTECDSKIIGTAVKNNLAIHSDLSYWIESRLNDTWKLERAVLGEVKSCTWPETHTLWGDGILESDLIIPVTLAGPRSNHNRRPGYKTQNQGPWDEGRVEIDFDYCPGTTVTLSESCGHRGPATRTTTESGKLITDWCCRSCTLPPLRYQTDSGCWYGMEIRPQRHDEKTLVQSQVNAYNADMIDPFQLGLLVVFLATQEVLRKRWTAKISMPAILIALLVLVFGGITYTDVLRYVILVGAAFAESNSGGDVVHLALMATFKIQPVFVVASFLKARWTNQENILLMLAAVFFQMAYHDARQILLWEIPDVLNSLAVAWMILRAITFTTTSNVVVPLLALLTPGLRCLNLDVYRILLLMVGIGSLIREKRSAAAKKKGASLLCLALASTGLFNPMILAAGLIACDPNRKRGWPATEVMTAVGLMFAIVGGLAELDIDSMAIPMTIAGLMFAAFVISGKSTDMWIERTADISWESDAEITGSSERVDVRLDDDGNFQLMNDPGAPWKIWMLRMVCLAISAYTPWAILPSVVGFWITLQYTKRGGVLWDTPSPKEYKKGDTTTGVYRIMTRGLLGSYQAGAGVMVEGVFHTLWHTTKGAALMSGEGRLDPYWGSVKEDRLCYGGPWKLQHKWNGQDEVQMIVVEPGKNVKNVQTKPGVFKTPEGEIGAVTLDFPTGTSGSPIVDKNGDVIGLYGNGVIMPNGSYISAIVQGERMDEPIPAGFEPEMLRKKQITVLDLHPGAGKTRRILPQIIKEAINRRLRTAVLAPTRVVAAEMAEALRGLPIRYQTSAVPREHNGNEIVDVMCHATLTHRLMSPHRVPNYNLFVMDEAHFTDPASIAARGYISTKVELGEAAAIFMTATPPGTSDPFPESNSPISDLQTEIPDRAWNSGYEWITEYTGKTVWFVPSVKMGNEIALCLQRAGKKVVQLNRKSYETEYPKCKNDDWDFVITTDISEMGANFKASRVIDSRKSVKPTIITEGEGRVILGEPSAVTAASAAQRRGRIGRNPSQVGDEYCYGGHTNEDDSNFAHWTEARIMLDNINMPNGLIAQFYQPEREKVYTMDGEYRLRGEERKNFLELLRTADLPVWLAYKVAAAGVSYHDRRWCFDGPRTNTILEDNNEVEVITKLGERKILRPRWIDARVYSDHQALKAFKDFASGKRSQIGLIEVLGKMPEHFMGKTWEALDTMYVVATAEKGGRAHRMALEELPDALQTIALIALLSVMTMGVFFLLMQRKGIGKIGLGGAVLGVATFFCWMAEVPGTKIAGMLLLSLLLMIVLIPEPEKQRSQTDNQLAVFLICVMTLVSAVAANEMGWLDKTKSDISSLFGQRIEVKENFSMGEFLLDLRPATAWSLYAVTTAVLTPLLKHLITSDYINTSLTSINVQASALFTLARGFPFVDVGVSALLLAAGCWGQVTLTVTVTAATLLFCHYAYMVPGWQAEAMRSAQRRTAAGIMKNAVVDGIVATDVPELERTTPIMQKKVGQIMLILVSLAAVVVNPSVKTVREAGILITAAAVTLWENGASSVWNATTAIGLCHIMRGGWLSCLSITWTLIKNMEKPGLKRGGAKGRTLGEVWKERLNQMTKEEFTRYRKEAIIEVDRSAAKHARKEGNVTGGHPVSRGTAKLRWLVERRFLEPVGKVIDLGCGRGGWCYYMATQKRVQEVRGYTKGGPGHEEPQLVQSYGWNIVTMKSGVDVFYRPSECCDTLLCDIGESSSSAEVEEHRTIRVLEMVEDWLHRGPREFCVKVLCPYMPKVIEKMELLQRRYGGGLVRNPLSRNSTHEMYWVSRASGNVVHSVNMTSQVLLGRMEKRTWKGPQYEEDVNLGSGTRAVGKPLLNSDTSKIKNRIERLRREYSSTWHHDENHPYRTWNYHGSYDVKPTGSASSLVNGVVRLLSKPWDTITNVTTMAMTDTTPFGQQRVFKEKVDTKAPEPPEGVKYVLNETTNWLWAFLAREKRPRMCSREEFIRKVNSNAALGAMFEEQNQWRSAREAVEDPKFWEMVDEEREAHLRGECHTCIYNMMGKREKKPGEFGKAKGSRAIWFMWLGARFLEFEALGFLNEDHWLGRKNSGGGVEGLGLQKLGYILREVGTRPGGKIYADDTAGWDTRITRADLENEAKVLELLDGEHRRLARAIIELTYRHKVVKVMRPAADGRTVMDVISREDQRGSGQVVTYALNTFTNLAVQLVRMMEGEGVIGPDDVEKLTKGKGPKVRTWLFENGEERLSRMAVSGDDCVVKPLDDRFATSLHFLNAMSKVRKDIQEWKPSTGWYDWQQVPFCSNHFTELIMKDGRTLVVPCRGQDELVGRARISPGAGWNVRDTACLAKSYAQMWLLLYFHRRDLRLMANAICSAVPVNWVPTGRTTWSIHAGGEWMTTEDMLEVWNRVWIEENEWMEDKTPVEKWSDVPYSGKREDIWCGSLIGTRARATWAENIQVAINQVRAIIGDEKYVDYMSSLKRYEDTTLVEDTVL

>AAZ32733.1 polyprotein precursor [West Nile virus]

MSKKPGGPGKSRAVNMLKRGMPRVLSLIGLKRAMLSLIDGKGPIRFVLALLAFFRFTAIAPTRAVLDRWRGVNKQTAMKHLLSFKKELGTLTSAINRRSSKQKKRGGKTGIAVMIGLIASVGAVTLSNFQGKVMMTVNATDVTDVITIPTAAGKNLCIVRAMDVGYMCDDTITYECPVLSAGNDPEDIDCWCTKSAVYVRYGRCTKTRHSRRSRRSLTVQTHGESTLANKKGAWMDSTKATRYLVKTESWILRNPGYALVAAVIGWMLGSNTMQRVVFVVLLLLVAPAYSFNCLGMSNRDFLEGVSGATWVDLVLEGDSCVTIMSKDKPTIDVKMMNMEAANLAEVRSYCYLATVSDLSTKAACPTMGEAHNDKRADPAFVCRQGVVDRGWGNGCGLFGKGSIDTCAKFACSTKAIGRTILKENIKYEVAIFVHGPTTVESHGNYSTQAGATQAGRFSITPAAPSYTLKLGEYGEVTVDCEPRSGIDTNAYYVMTVGTKTFLVHREWFMDLNLPWSSAGSTVWRNRETLMEFEEPHATKQSVIALGSQEGALHQALAGAIPVEFSSNTVKLTSGHLKCRVKMEKLQLKGTTYGVCSKAFKFLGTPADTGHGTVVLELQYTGTDGPCKVPISSVASLNDLTPVGRLVTVNPFVSVATANAKVLIELEPPFGDSYIVVGRGEQQINHHWHKSGSSIGKAFTTTLKGAQRLAALGDTAWDFGSVGGVFTSVGKAVHQVFGGAFRSLFGGMSWITQGLLGALLLWMGINARDRSIALTFLAVGGVLLFLSVNVHADTGCAIDISRQELRCGSGVFIHNDVEAWMDRYKYYPETPQGLAKIIQKAHKEGVCGLRSVSRLEHQMWEAVKDELNTLLKENGVDLSVVVEKQEGMYKSAPKRLTATTENLEIGWKAWGKSILFAPELANNTFVVDGPETKECPTQNRAWNSLEVEDFGFGLTSTRMFLKVRESNTTECDSKIIGTAVKNNLAIHSDLSYWIESRLNDTWKLERAVLGEVKSCTWPETHTLWGDGILESDLIIPVTLAGPRSNHNRRPGYKTQNQGPWDEGRVEIDFDYCPGTTVTLSESCGHRGPATRTTTESGKLITDWCCRSCTLPPLRYQTDSGCWYGMEIRPQRHDEKTLVQSQVNAYNADMIDPFQLGLLVVFLATQEVLRKRWTAKISMPAILIALLVLVFGGITYTDVLRYVILVGAAFAESNSGGDVVHLALMATFKIQPVFMVASFLKARWTNQENILLMLAAVFFQMAYHDARQILLWEIPDVLNSLAVAWMILRAITFTTTSNVVVPLLALLTPGLRCLNLDVYRILLLMVGIGSLIREKRSAAAKKKGASLLCLALASTGLFNPMILAAGLIACDPNRKRGWPATEVMTAVGLMFAIVGGLAELDIDSMAIPMTIAGLMFAAFVISGKSTDMWIERTADISWESDAEITGSSERVDVRLDDDGNFQLMNDPGAPWKIWMLRMVCLAISAYTPWAILPSVVGFWITLQYTKRGGVLWDTPSPKEYKKGDTTTGVYRIMTRGLLGSYQAGAGVMVEGVFHTLWHTTKGAALMSGEGRLDPYWGSVKEDRLCYGGPWKLQHKWNGQDEVQMIVVEPGKNVKNVQTKPGVFKTPEGEIGAVTLDFPTGTSGSPIVDKNGDVIGLYGNGVIMPNGSYISAIVQGERMDEPIPAGFEPEMLRKKQITVLDLHPGAGKTRRILPQIIKEAINRRLRTAVLAPTRVVAAEMAEALRGLPIRYQTSAVPREHNGNEIVDVMCHATLTHRLMSPHRVPNYNLFVMDEAHFTDPASIAARGYISTKVELGEAAAIFMTATPPGTSDPFPESNSPISDLQTEIPDRAWNSGYEWITEYTGKTVWFVPSVKMGNEIALCLQRAGKKVVQLNRKSYETEYPKCKNDDWDFVITTDISEMGANFKASRVIDSRKSVKPTIITEGEGRVILGEPSAVTAASAAQRRGRIGRNPSQVGDEYCYGGHTNEDDSNFAHWTEARIMLDNINMPNGLIAQFYQPEREKVYTMDGEYRLRGEERKNFLELLRTADLPVWLAYKVAAAGVSYHDRRWCFDGPRTNTILEDNNEVEVITKLGERKILRPRWIDARVYSDHQALKAFKDFASGKRSQIGLIEVLGKMPEHFMGKTWEALDTMYVVATAEKGGRAHRMALEELPDALQTIALIALLSVMTMGVFFLLMQRKGIGKIGLGGAVLGVATFFCWMAEVPGTKIAGMLLLSLLLMIVLIPEPEKQRSQTDNQLAVFLICVMTLVSAVAANEMGWLDKTKSDISSLFGQRIEVKENFSMGEFLLDLRPATAWSLYAVTTAVLTPLLKHLITSDYINTSLTSINVQASALFTLARGFPFVDVGVSALLLAAGCWGQVTLTVTVTAATLLFCHYAYMVPGWQAEAMRSAQRRTAAGIMKNAVVDGIVATDVPELERTTPIMQKKVGQIMLILVSLAAVVVNPSVKTVREAGILITAAAVTLWENGASSVWNATTAIGLCHIMRGGWLSCLSITWTLIKNMEKPGLKRGGAKGRTLGEVWKERLNQMTKEEFTRYRKEAIIEVDRSAAKHARKEGNVTGGHPVSRGTAKLRWLVERRFLEPVGKVIDLGCGRGGWCYYMATQKRVQEVRGYTKGGPGHEEPQLVQSYGWNIVTMKSGVDVFYRPSECCDTLLCDIGESSSSAEVEEHRTIRVLEMVEDWLHRGPREFCVKVLCPYMPKVIEKMELLQRRYGGGLVRNPLSRNSTHEMYWVSRASGNVVHSVNMTSQVLLGRMEKRTWKGPQYEEDVNLGSGTRAVGKPLLNSDTSKIKNRIERLRREYSSTWHHDENHPYRTWNYHGSYDVKPTGSASSLVNGVVRLLSKPWDTITNVTTMAMTDTTPFGQQRVFKEKVDTKAPEPPEGVKYVLNETTNWLWAFLAREKRPRMCSREEFIRKVNSNAALGAMFEEQNQWRSAREAVEDPKFWEMVDEEREAHLRGECHTCIYNMMGKREKKPGEFGKAKGSRAIWFMWLGARFLEFEALGFLNEDHWLGRKNSGGGVEGLGLQKLGYILREVGTRPGGKIYADDTAGWDTRITRADLENEAKVLELLDGEHRRLARAIIELTYRHKVVKVMRPAADGRTVMDVISREDQRGSGQVVTYALNTFTNLAVQLVRMMEGEGVIGPDDVEKLTKGKGPKVRTWLFENGEERLSRMAVSGDDCVVKPLDDRFATSLHFLNAMSKVRKDIQEWKPSTGWYDWQQVPFCSNHFTELIMKDGRTLVVPCRGQDELVGRARISPGAGWNVRDTACLAKSYAQMWLLLYFHRRDLRLMANAICSAVPVNWVPTGRTTWSIHAGGEWMTTEDMLEVWNRVWIEENEWMEDKTPVEKWSDVPYSGKREDIWCGSLIGTRARATWAENIQVAINQVRAIIGDEKYVDYMSSLKRYEDTTLVEDTVL

>AHW48367.1 polyprotein [West Nile virus]

MSKKPGGPGKSRAVNMLKRGMPRVLSLIGLKRAMLSLIDGKGPIRFVLALLAFFRFTAIAPTRAVLDRWRGVNKQTAMKHLLSFKKELGTLTSAINRRSSKQKKRGGKTGIAVMIGLIASVGAVTLSNFQGKVMMTVNATDVTDVITIPTAAGKNLCIVRAMDVGYMCDDTITYECPVLSAGNDPEDIDCWCTKSAVYVRYGRCTKTRHSRRSRRSLTVQTHGESTLANKKGAWMDSTKATRYLVKTESWILRNPGYALVAAVIGWMLGSNTMQRVVFVVLLLLVAPAYSFNCLGMSNRDFLEGVSGATWVDLVLEGDSCVTIMSKDKPTIDVKMMNMEAANLAEVRSYCYLATVSDLSTKAACPTMGEAHNDKRADPAFVCRQGVVDRGWGNGCGLFGKGSIDTCAKFACSTKAIGRTILKENIKYEVAIFVHGPTTVESHGNYSTQAGATQAGRFSITPAAPSYTLKLGEYGEVTVDCEPRSGIDTNAYYVMTVGTKTFLVHREWFMDLNLPWSSAGSTVWRNRETLMEFEEPHATKQSVIALGSQEGALHQALAGAIPVEFSSNTVKLTSGHLKCRVKMEKLQLKGTTYGVCSKAFKFLGTPADTGHGTVVLELQYTGTDGPCKVPISSVASLNDLTPVGRLVTVNPFVSVATANAKVLIELEPPFGDSYIVVGRGEQQINHHWHKSGSSIGKAFTTTLKGAQRLAALGDTAWDFGSVGGVFTSVGKAVHQVFGGAFRSLFGGMSWITQGLLGALLLWMGINARDRSIALTFLAVGGVLLFLSVNVHADTGCAIDISRQELRCGSGVFIHNDVEAWMDRYKYYPETPQGLAKIIQKAHKEGVCGLRSVSRLEHQMWEAVKDELNTLLKENGVDLSVVVEKQEGMYKSAPKRLTATTEKLEIGWKAWGKSILFAPELANNTFVVDGPETKECPTQNRAWNSLEVEDFGFGLTSTRMFLKVRESNTTECDSKIIGTAVKNNLAIHSDLSYWIESRLNDTWKLERAVLGEVKSCTWPETHTLWGDGILESDLIIPVTLAGPRSNHNRRPGYKTQNQGPWDEGRVEIDFDYCPGTTVTLSESCGHRGPATRTTTESGKLITDWCCRSCTLPPLRYQTDSGCWYGMEIRPQRHDEKTLVQSQVNAYNADMIDPFQLGLLVVFLATQEVLRKRWTAKISMPAILIALLVLVFGGITYTDVLRYVILVGAAFAESNSGGDVVHLALMATFKIQPVFMVASFLKARWTNQENILLMLAAVFFQMAYHDARQILLWEIPDVLNSLAVAWMILRAITFTTTSNVVVPLLALLTPGLRCLNLDVYRILLLMVGIGSLIREKRSAAAKKKGASLLCLALASTGLFNPMILAAGLIACDPNRKRGWPATEVMTAVGLMFAIVGGLAELDIDSMAIPMTIAGLMFAAFVISGKSTDMWIERTADISWESDAEITGSSERVDVRLDDDGNFQLMNDPGAPWKIWMLRMVCLAISAYTPWAILPSVVGFWITLQYTKRGGVLWDTPSPKEYKKGDTTTGVYRIMTRGLLGSYQAGAGVMVEGVFHTLWHTTKGAALMSGEGRLDPYWGSVKEDRLCYGGPWKLQHKWNGQDEVQMIVVEPGKNVKNVQTKPGVFKTPEGEIGAVTLDFPTGTSGSPIVDKNGDVIGLYGNGVIMPNGSYISAIVQGERMDEPIPAGFEPEMLRKKQITVLDLHPGAGKTRRILPQIIKEAINRRLRTAVLAPTRVVAAEMAEALRGLPIRYQTSAVPREHNGNEIVDVMCHATLTHRLMSPHRVPNYNLFVMDEAHFTDPASIAARGYISTKVELGEAAAIFMTATPPGTSDPFPESNSPISDLQTEIPDRAWNSGYEWITEYTGKTVWFVPSVKMGNEIALCLQRAGKKVVQLNRKSYETEYPKCKNDDWDFVITTDISEMGANFKASRVIDSRKSVKPTIITEGEGRVILGEPSAVTAASAAQRRGRIGRNPSQVGDEYCYGGHTNEDDSNFAHWTEARIMLDNINMPNGLIAQFYQPEREKVYTMDGEYRLRGEERKNFLELLRTADLPVWLAYKVAAAGVSYHDRRWCFDGPRTNTILEDNNEVEVITKLGERKILRPRWIDARVYSDHQALKAFKDFASGKRSQIGLIEVLGKMPEHFMGKTWEALDTMYVVATAEKGGRAHRMALEELPDALQTIALIALLSVMTMGVFFLLMQRKGIGKIGLGGAVLGVATFFCWMAEVPGTKIAGMLLLSLLLMIVLIPEPEKQRSQTDNQLAVFLICVMTLVSAVAANEMGWLDKTKSDISSLFGQRIEVKENFSMGEFLLDLRPATAWSLYAVTTAVLTPLLKHLITSDYINTSLTSINVQASALFTLARGFPFVDVGVSALLLAAGCWGQVTLTVTVTAATLLFCHYAYMVPGWQAEAMRSAQRRTAAGIMKNAVVDGIVATDVPELERTTPIMQKKVGQIMLILVSLAAVVVNPSVKTVREAGILITAAAVTLWENGASSVWNATTAIGLCHIMRGGWLSCLSITWTLIKNMEKPGLKRGGAKGRTLGEVWKERLNQMTKEEFTRYRKEAIIEVDRSAAKHARKEGNVTGGHPVSRGTAKLRWLVERRFLEPVGKVIDLGCGRGGWCYYMATQKRVQEVRGYTKGGPGHEEPQLVQSYGWNIVTMKSGVDVFYRPSECCDTLLCDIGESSSSAEVEEHRTIRVLEMVEDWLHRGPREFCVKVLCPYMPKVIEKMELLQRRYGGGLVRNPLSRNSTHEMYWVSRASGNVVHSVNMTSQVLLGRMEKRTWKGPQYEEDVNLGSGTRAVGKPLLNSDTSKIKNRIERLRREYSSTWHHDENHPYRTWNYHGSYDVNPTGSASSLVNGVVRLLSKPWDTITNVTTMAMTDTTPFGQQRVFKEKVDTKAPEPPEGVKYVLNETTNWLWAFLAREKRPRMCSREEFIRKVNSNAALGAMFEEQNQWRSAREAVEDPKFWEMVDEEREAHLRGECHTCIYNMMGKREKKPGEFGKAKGSRAIWFMWLGARFLEFEALGFLNEDHWLGRKNSGGGVEGLGLQKLGYILREVGTRPGGKIYADDTAGWDTRITRADLENEAKVLELLDGEHRRLARAIIELTYRHKVVKVMRPAADGRTVMDVISREDQRGSGQVVTYALNTFTNLAVQLVRMMEGEGVIGPDDVEKLTKGKGPKVRTWLFENGEERLSRMAVSGDDCVVKPLDDRFATSLHFLNAMSKVRKDIQEWKPSTGWYDWQQVPFCSNHFTELIMKDGRTLVVPCRGQDELVGRARISPGAGWNVRDTACLAKSYAQMWLLLYFHRRDLRLMANAICSAVPVNWVPTGRTTWSIHAGGEWMTTEDMLEVWNRVWIEENEWMEDKTPVEKWSDVPYSGKREDIWCGSLIGTRARATWAENIQVAINQVRAIIGDEKYVDYMSSLKRYEDTTLVEDTVL

>ANW69082.1 genome polyprotein [West Nile virus]

MSKKPGGPGKSRAVNMLKRGMPRVLSLIGLKRAMLSLIDGKGPIRFVLALLAFFRFTAIAPTRAVLDRWRGVNKQTAMKHLLSFKKELGTLTSAINRRSSKQKKRGGKTGIAVMIGLIASVGAVTLSNFQGKVMMTVNATDVTDVITIPTAAGKNLCIVRAMDVGYMCDDTITYECPVLSAGNDPEDIDCWCTKSAVYVRYGRCTKTRHSRRSRRSLTVQTHGESTLANKKGAWMDSTKATRYLVKTESWILRNPGYALVAAVIGWMLGSNTMQRVVFVVLLLLVAPAYSFNCLGMSNRDFLEGVSGATWVDLVLEGDSCVTIMSKDKPTIDVKMMNMEAANLAEVRSYCYLATVSDLSTKAACPTMGEAHNDKRADPAFVCRQGVVDRGWGNGCGLFGKGSIDTCAKFACSTKAIGRTILKENIKYEVAIFVHGPTTVESHGNYSTQAGATQAGRFSITPAAPSYTLKLGEYGEVTVDCEPRSGIDTNAYYVMTVGTKTFLVHREWFMDLNLPWSSAGSTVWRNRETLMEFEEPHATKQSVIALGSQEGALHQALAGAIPVEFSSNTVKLTSGHLKCRVKMEKLQLKGTTYGVCSKAFKFLGTPADTGHGTVVLELQYTGTDGPCKVPISSVASLNDLTPVGRLVTVNPFVSVATANAKVLIELEPPFGDSYIVVGRGEQQINHHWHKSGSSIGKAFTTTLKGAQRLAALGDTAWDFGSVGGVFTSVGKAVHQVFGGAFRSLFGGMSWITQGLLGALLLWMGINARDRSIALTFLAVGGVLLFLSVNVHADTGCAIDISRQELRCGSGVFIHNDVEAWMDRYKYYPETPQGLAKIIQKAHKEGVCGLRSVSRLEHQMWEAVKDELNTLLKENGVDLSVVVEKQEGMYKSAPKRLTATTEKLEIGWKAWGKSILFAPELANNTFVVDGPETKECPTQNRAWNSLEVEDFGFGLTSTRMFLKVRESNTTECDSKIIGTAVKNNLAIHSDLSYWIESRLNDTWKLERAVLGEVKSCTWPETHTLWGDGILESDLIIPVTLAGPRSNHNRRPGYKTQNQGPWDEGRVEIDFDYCPGTTVTLSESCGHRGPATRTTTESGKLITDWCCRSCTLPPLRYQTDSGCWYGMEIRPQRHDEKTLVQSQVNAYNADMIDPFQLGLLVVFLATQEVLRKRWTAKISMPAILIALLVLVFGGITYTDVLRYVILVGAAFAESNSGGDVVHLALMATFKIQPVFMVASFLKARWTNQENILLMLAAVFFQMAYHDARQILLWETPDVLNSLAVAWMILRAITFTTTSNVVVPLLALLTPGLRCLNLDVYRILLLMVGIGSLIREKRSAAAKKKGASLLCLALASTGLFNPMILAAGLIACDPNRKRGWPATEVMTAVGLMFAIVGGLAELDIDSMAIPMTIAGLMFAAFVISGKSTDMWIERTADISWESDAEITGSSERVDVRLDDDGNFQLMNDPGAPWKIWMLRMVCLAISAYTPWAILPSVVGFWITLQYTKRGGVLWDTPSPKEYKKGDTTTGVYRIMTRGLLGSYQAGAGVMVEGVFHTLWHTTKGAALMSGEGRLDPYWGSVKEDRLCYGGPWKLQHKWNGQDEVQMIVVEPGKNVKNVQTKPGVFKTPEGEIGAVTLDFPTGTSGSPIVDKNGDVIGLYGNGVIMPNGSYISAIVQGERMDEPIPAGFEPEMLRKKQITVLDLHPGAGKTRRILPQIIKEAINRRLRTAVLAPTRVVAAEMAEALRGLPIRYQTSAVPREHNGNEIVDVMCHATLTHRLMSPHRVPNYNLFVMDEAHFTDPASIAARGYISTKVELGEAAAIFMTATPPGTSDPFPESNSPISDLQTEIPDRAWNSGYEWITEYTGKTVWFVPSVKMGNEIALCLQRAGKKVVQLNRKSYETEYPKCKNDDWDFVITTDISEMGANFKASRVIDSRKSVKPTIITEGEGRVILGEPSAVTAASAAQRRGRIGRNPSQVGDEYCYGGHTNEDDSNFAHWTEARIMLDNINMPNGLIAQFYQPEREKVYTMDGEYRLRGEERKNFLELLRTADLPVWLAYKVAAAGVSYHDRRWCFDGPRTNTILEDNNEVEVITKLGERKILRPRWIDARVYSDHQALKAFKDFASGKRSQIGLIEVLGKMPEHFMGKTWEALDTMYVVATAEKGGRAHRMALEELPDALQTIALIALLSVMTMGVFFLLMQRKGIGKIGLGGAVLGVATFFCWMAEVPGTKIAGMLLLSLLLMIVLIPEPEKQRSQTDNQLAVFLICVMTLVSAVAANEMGWLDKTKSDISSLFGQRIEVKENFSMGEFLLDLRPATAWSLYAVTTAVLTPLLKHLITSDYINTSLTSINVQASALFTLARGFPFVDVGVSALLLAAGCWGQVTLTVTVTAATLLFCHYAYMVPGWQAEAMRSAQRRTAAGIMKNAVVDGIVATDVPELERTTPIMQKKVGQIMLILVSLAAVVVNPSVKTVREAGILITAAAVTLWENGASSVWNATTAIGLCHIMRGGWLSCLSITWTLIKNMEKPGLKRGGAKGRTLGEVWKERLNQMTKEEFTRYRKEAIIEVDRSAAKHARKEGNVTGGHPVSRGTAKLRWLVERRFLEPVGKVIDLGCGRGGWCYYMATQKRVQEVRGYTKGGPGHEEPQLVQSYGWNIVTMKSGVDVFYRPSECCDTLLCDIGESSSSAEVEEHRTIRVLEMVEDWLHRGPREFCVKVLCPYMPKVIEKMELLQRRYGGGLVRNPLSRNSTHEMYWVSRASGNVVHSVNMTSQVLLGRMEKRTWKGPQYEEDVNLGSGTRAVGKPLLNSDTSKIKNRIERLRREYSSTWHHDENHPYRTWNYHGSYDVKPTGSASSLVNGVVRLLSKPWDTITNVTTMAMTDTTPFGQQRVFKEKVDTKAPEPPEGVKYVLNETTNWLWAFLAREKRPRMCSREEFIRKVNSNAALGAMFEEQNQWRSAREAVEDPKFWEMVDEEREAHLRGECHTCIYNMMGKREKKPGEFGKAKGSRAIWFMWLGARFLEFEALGFLNEDHWLGRKNSGGGVEGLGLQKLGYILREVGTRPGGKIYADDTAGWDTRITRADLENEAKVLELLDGEHRRLARAIIELTYRHKVVKVMRPAADGRTVMDVISREDQRGSGQVVTYALNTFTNLAVQLVRMMEGEGVIGPDDVEKLTKGKGPKVRTWLFENGEERLSRMAVSGDDCVVKPLDDRFATSLHFLNAMSKVRKDIQEWKPSTGWYDWQQVPFCSNHFTELIMKDGRTLVVPCRGQDELVGRARISPGAGWNVRDTACLAKSYAQMWLLLYFHRRDLRLMANAICSAVPVNWVPTGRTTWSIHAGGEWMTTEDMLEVWNRVWIEENEWMEDKTPVEKWSDVPYSGKREDIWCGSLIGTRARATWAENIQVAINQVRAIIGDEKYVDYMSSLKRYEDTTLVEDTVL

>ANW69277.1 genome polyprotein [West Nile virus]

MSKKPGGPGKSRAVNMLKRGMPRVLSLIGLKRAMLSLIDGKGPIRFVLALLAFFRFTAIAPTRAVLDRWRGVNKQTAMKHLLSFKKELGTLTSAINRRSSKQKKRGGKTGIAVMIGLIASVGAVTLSNFQGKVMMTVNATDVTDVITIPTAAGKNLCIVRAMDVGYMCDDTITYECPVLSAGNDPEDIDCWCTKSAVYVRYGRCTKTRHSRRSRRSLTVQTHGESTLANKKGAWMDSTKATRYLVKTESWILRNPGYALVAAVIGWMLGSNTMQRVVFVVLLLLVAPAYSFNCLGMSNRDFLEGVSGATWVDLVLEGDSCVTIMSKDKPTIDVKMMNMEAANLAEVRSYCYLATVSDLSTKAACPTMGEAHNDKRADPAFVCRQGVVDRGWGNGCGLFGKGSIDTCAKFACSTKAIGRTILKENIKYEVAIFVHGPTTVESHGNYSTQAGATQAGRFSITPAAPSYTLKLGEYGEVTVDCEPRSGIDTNAYYVMTVGTKTFLVHREWFMDLNLPWSSAGSTVWRNRETLMEFEEPHATKQSVIALGSQEGALHQALAGAIPVEFSSNTVKLTSGHLKCRVKMEKLQLKGTTYGVCSKAFKFLGTPADTGHGTVVLELQYTGTDGPCKVPISSVASLNDLTPVGRLVTVNPFVSVATANAKVLIELEPPFGDSYIVVGRGEQQINHHWHKSGSSIGKAFTTTLKGAQRLAALGDTAWDFGSVGGVFTSVGKAVHQVFGGAFRSLFGGMSWITQGLLGALLLWMGINARDRSIALTFLAVGGVLLFLSVNVHADTGCAIDISRQELRCGSGVFIHNDVEAWMDRYKYYPETPQGLAKIIQKAHKEGVCGLRSVSRLEHQMWEAVKDELNTLLKENGVDLSVVVEKQEGMYKSAPKRLTATTEKLEIGWKAWGKSILFAPELANNTFVVDGPETKECPTQNRAWNSLEVEDFGFGLTSTRMFLKVRESNTTECDSKIIGTAVKNNLAIHSDLSYWIESRLNDTWKLERAVLGEVKSCTWPETHTLWGDGILESDLIIPVTLAGPRSNHNRRPGYKTQNQGPWDEGRVEIDFDYCPGTTVTLSESCGHRGPATRTTTESGKLITDWCCRSCTLPPLRYQTDSGCWYGMEIRPQRHDEKTLVQSQVNAYNADMIDPFQLGLLVVFLATQEVLRKRWTAKISMPAILIALLVLVFGGITYTDVLRYVILVGAAFAESNSGGDVVHLALMATFKIQPVFMVASFLKARWTNQENILLMLAAVFFQMAYHDARQILLWEIPDVLNSLAVAWMILRAITFTTTSNVVVPLLALLTPGLRCLNLDVYRILLLMVGIGSLIREKRSAAAKKKGASLLCLALASTGLFNPMILAAGLIACDPNRKRGWPATEVMTAVGLMFAIVGGLAELDIDSMAIPMTIAGLMFAAFVISGKSTDMWIERTADISWESDAEITGSSERVDVRLDDDGNFQLMNDPGAPWKIWMLRMVCLAISAYTPWAILPSVVGFWITLQYTKRGGVLWDTPSPKEYKKGDTTTGVYRIMTRGLLGSYQAGAGVMVEGVFHTLWHTTKGAALMSGEGRLDPYWGSVKEDRLCYGGPWKLQHKWNGQDEVQMIVVEPGKNVKNVQTKPGVFKTPEGEIGAVTLDFPTGTSGSPIVDKSGDVIGLYGNGVIMPNGSYISAIVQGERMDEPIPAGFEPEMLRKKQITVLDLHPGAGKTRRILPQIIKEAINRRLRTAVLAPTRVVAAEMAEALRGLPIRYQTSAVPREHNGNEIVDVMCHATLTHRLMSPHRVPNYNLFVMDEAHFTDPASIAARGYISTKVELGEAAAIFMTATPPGTSDPFPESNSPISDLQTEIPDRAWNSGYEWITEYTGKTVWFVPSVKMGNEIALCLQRAGKKVVQLNRKSYETEYPKCKNDDWDFVITTDISEMGANFKASRVIDSRKSVKPTIITEGEGRVILGEPSAVTAASAAQRRGRIGRNPSQVGDEYCYGGHTNEDDSNFAHWTEARIMLDNINMPNGLIAQFYQPEREKVYTMDGEYRLRGEERKNFLELLRTADLPVWLAYKVAAAGVSYHDRRWCFDGPRTNTILEDNNEVEVITKLGERKILRPRWIDARVYSDHQALKAFKDFASGKRSQIGLIEVLGKMPEHFMGKTWEALDTMYVVATAEKGGRAHRMALEELPDALQTIALIALLSVMTMGVFFLLMQRKGIGKIGLGGAVLGVATFFCWMAEVPGTKIAGMLLLSLLLMIVLIPEPEKQRSQTDNQLAVFLICVMTLVSAVAANEMGWLDKTKSDISSLFGQRIEVKENFSMGEFLLDLRPATAWSLYAVTTAVLTPLLKHLITSDYINTSLTSINVQASALFTLARGFPFVDVGVSALLLAAGCWGQVTLTVTVTAATLLFCHYAYMVPGWQAEAMRSAQRRTAAGIMKNAVVDGIVATDVPELERTTPIMQKKVGQIMLILVSLAAVVVNPSVKTVREAGILITAAAVTLWENGASSVWNATTAIGLCHIMRGGWLSCLSITWTLIKNMEKPGLKRGGAKGRTLGEVWKERLNQMTKEEFTRYRKEAIIEVDRSAAKHARKEGNVTGGHPVSRGTAKLRWLVERRFLEPVGKVIDLGCGRGGWCYYMATQKRVQEVRGYTKGGPGHEEPQLVQSYGWNIVTMKSGVDVFYRPSECCDTLLCDIGESSSSAEVEEHRTIRVLEMVEDWLHRGPREFCVKVLCPYMPKVIEKMELLQRRYGGGLVRNPLSRNSTHEMYWVSRASGNVVHSVNMTSQVLLGRMEKRTWKGPQYEEDVNLGSGTRAVGKPLLNSDTSKIKNRIERLRREYSSTWHHDENHPYRTWNYHGSYDVKPTGSASSLVNGVVRLLSKPWDTITNVTTMAMTDTTPFGQQRVFKEKVDTKAPEPPEGVKYVLNETTNWLWAFLAREKRPRMCSREEFIRKVNSNAALGAMFEEQNQWRSAREAVEDPKFWEMVDEEREAHLRGECHTCIYNMMGKREKKPGEFGKAKGSRAIWFMWLGARFLEFEALGFLNEDHWLGRKNSGGGVEGLGLQKLGYILREVGTRPGGKIYADDTAGWDTRITRADLENEAKVLELLDGEHRRLARAIIELTYRHKVVKVMRPAADGRTVMDVISREDQRGSGQVVTYALNTFTNLAVQLVRMMEGEGVIGPDDVEKLTKGKGPKVRTWLFENGEERLSRMAVSGDDCVVKPLDDRFATSLHFLNAMSKVRKDIQEWKPSTGWYDWQQVPFCSNHFTELIMKDGRTLVVPCRGQDELVGRARISPGAGWNVRDTACLAKSYAQMWLLLYFHRRDLRLMANAICSAVPVNWVPTGRTTWSIHAGGEWMTTEDMLEVWNRVWIEENEWMEDKTPVEKWSDVPYSGKREDIWCGSLIGTRARATWAENIQVAINQVRAIIGDEKYVDYMSSLKRYEDTTLVEDTVL

>ANW68500.1 genome polyprotein [West Nile virus]

MSKKPGGPGKSRAVNMLKRGMPRVLSLIGLKRAMLSLIDGKGPIRFVLALLAFFRFTAIAPTRAVLDRWRGVNKQTAMKHLLSFKKELGTLTSAINRRSSKQKKRGGKTGIAVMIGLIASVGAVTLSNFQGKVMMTVNATDVTDVITIPTAAGKNLCIVRAMDVGYMCDDTITYECPVLSAGNDPEDIDCWCTKSAVYVRYGRCTKTRHSRRSRRSLTVQTHGESTLANKKGAWMDSTKATRYLVKTESWILRNPGYALVAAVIGWMLGSNTMQRVVFVVLLLLVAPAYSFNCLGMSNRDFLEGVSGATWVDLVLEGDSCVTIMSKDKPTIDVKMVNMEAANLAEVRSYCYLATVSDLSTKAACPTMGEAHNDKRADPAFVCRQGVVDRGWGNGCGLFGKGSIDTCAKFACSTKAIGRTILKENIKYEVAIFVHGPTTVESHGNYSTQAGATQAGRFSITPAAPSYTLKLGEYGEVTVDCEPRSGIDTNAYYVMTVGTKTFLVHREWFMDLNLPWSSAGSTVWRNRETLMEFEEPHATKQSVIALGSQEGALHQALAGAIPVEFSSNTVKLTSGHLKCRVKMEKLQLKGTTYGVCSKAFKFLGTPADTGHGTVVLELQYTGTDGPCKVPISSVASLNDLTPVGRLVTVNPFVSVATANAKVLIELEPPFGDSYIVVGRGEQQINHHWHKSGSSIGKAFTTTLKGAQRLAALGDTAWDFGSVGGVFTSVGKAVHQVFGGAFRSLFGGMSWITQGLLGALLLWMGINARDRSIALTFLAVGGVLLFLSVNVHADTGCAIDISRQELRCGSGVFIHNDVEAWMDRYKYYPETPQGLAKIIQKAHKEGVCGLRSVSRLEHQMWEAVKDELNTLLKENGVDLSVVVEKQEGMYKSAPKRLTATTEKLEIGWKAWGKSILFAPELANNTFVVDGPETKECPTQNRAWNSLEVEDFGFGLTSTRMFLKVRESNTTECDSKIIGTAVKNNLAIHSDLSYWIESRLNDTWKLERAVLGEVKSCTWPETHTLWGDGILESDLIIPVTLAGPRSNHNRRPGYKTQNQGPWDEGRVEIDFDYCPGTTVTLSESCGHRGPATRTTTESGKLITDWCCRSCTLPPLRYQTDSGCWYGMEIRPQRHDEKTLVQSQVNAYNADMIDPFQLGLLVVFLATQEVLRKRWTAKISMPAILIALLVLVFGGITYTDVLRYVILVGAAFAESNSGGDVVHLALMATFKIQPVFMVASFLKARWTNQENILLMLAAVFFQMAYHDARQILLWEIPDVLNSLAVAWMILRAITFTTTSNVVVPLLALLTPGLRCLNLDVYRILLLMVGIGSLIREKRSAAAKKKGASLLCLALASTGLFNPMILAAGLIACDPNRKRGWPATEVMTAVGLMFAIVGGLAELDIDSMAIPMTIAGLMFAAFVISGKSTDMWIERTADISWESDAEITGSSERVDVRLDDDGNFQLMNDPGAPWKIWMLRMVCLAISAYTPWAILPSVVGFWITLQYTKRGGVLWDTPSPKEYKKGDTTTGVYRIMTRGLLGSYQAGAGVMVEGVFHTLWHTTKGAALMSGEGRLDPYWGSVKEDRLCYGGPWKLQHKWNGQDEVQMIVVEPGKNVKNVQTKPGVFKTPEGEIGAVTLDFPTGTSGSPIVDKNGDVIGLYGNGVIMPNGSYISAIVQGERMDEPIPAGFEPEMLRKKQITVLDLHPGAGKTRRILPQIIKEAINRRLRTAVLAPTRVVAAEMAEALRGLPIRYQTSAVPREHNGNEIVDVMCHATLTHRLMSPHRVPNYNLFVMDEAHFTDPASIAARGYISTKVELGEAAAIFMTATPPGTSDPFPESNSPISDLQTEIPDRAWNSGYEWITEYTGKTVWFVPSVKMGNEIALCLQRAGKKVVQLNRKSYETEYPKCKNDDWDFVITTDISEMGANFKASRVIDSRKSVKPTIITEGEGRVILGEPSAVTAASAAQRRGRIGRNPSQVGDEYCYGGHTNEDDSNFAHWTEARIMLDNINMPNGLIAQFYQPEREKVYTMDGEYRLRGEERKNFLELLRTADLPVWLAYKVAAAGVSYHDRRWCFDGPRTNTILEDNNEVEVITKLGERKILRPRWIDARVYSDHQALKAFKDFASGKRSQIGLIEVLGKMPEHFMGKTWEALDTMYVVATAEKGGRAHRMALEELPDALQTIALIALLSVMTMGVFFLLMQRKGIGKIGLGGAVLGVATFFCWMAEVPGTKIAGMLLLSLLLMIVLIPEPEKQRSQTDNQLAVFLICVMTLVSAVAANEMGWLDKTKSDISSLFGQRIEVKENFSMGEFLLDLRPATAWSLYAVTTAVLTPLLKHLITSDYINTSLTSINVQASALFTLARGFPFVDVGVSALLLAAGCWGQVTLTVTVTAATLLFCHYAYMVPGWQAEAMRSAQRRTAAGIMKNAVVDGIVATDVPELERTTPIMQKKVGQIMLILVSLAAVVVNPSVKTVREAGILITAAAVTLWENGASSVWNATTAIGLCHIMRGGWLSCLSITWTLIKNMEKPGLKRGGAKGRTLGEVWKERLNQMTKEEFTRYRKEAIIEVDRSAAKHARKEGNVTGGHPVSRGTAKLRWLVERRFLEPVGKVIDLGCGRGGWCYYMATQKRVQEVRGYTKGGPGHEEPQLVQSYGWNIVTMKSGVDVFYRPSECCDTLLCDIGESSSSAEVEEHRTIRVLEMVEDWLHRGPREFCVKVLCPYMPKVIEKMELLQRRYGGGLVRNPLSRNSTHEMYWVSRASGNVVHSVNMTSQVLLGRMEKRTWKGPQYEEDVNLGSGTRAVGKPLLNSDTSKIKNRIERLRREYSSTWHHDENHPYRTWNYHGSYDVKPTGSASSLVNGVVRLLSKPWDTITNVTTMAMTDTTPFGQQRVFKEKVDTKAPEPPEGVKYVLNETTNWLWAFLAREKRPRMCSREEFIRKVNSNAALGAMFEEQNQWRSAREAVEDPKFWEMVDEEREAHLRGECHTCIYNMMGKREKKPGEFGKAKGSRAIWFMWLGARFLEFEALGFLNEDHWLGRKNSGGGVEGLGLQKLGYILREVGTRPGGKIYADDTAGWDTRITRADLENEAKVLELLDGEHRRLARAIIELTYRHKVVKVMRPAADGRTVMDVISREDQRGSGQVVTYALNTFTNLAVQLVRMMEGEGVIGPDDVEKLTKGKGPKVRTWLFENGEERLSRMAVSGDDCVVKPLDDRFATSLHFLNAMSKVRKDIQEWKPSTGWYDWQQVPFCSNHFTELIMKDGRTLVVPCRGQDELVGRARISPGAGWNVRDTACLAKSYAQMWLLLYFHRRDLRLMANAICSAVPVNWVPTGRTTWSIHAGGEWMTTEDMLEVWNRVWIEENEWMEDKTPVEKWSDVPYSGKREDIWCGSLIGTRARATWAENIQVAINQVRAIIGDEKYVDYMSSLKRYEDTTLVEDTVL

>ANW68647.1 genome polyprotein [West Nile virus]

MSKKPGGPGKSRAVNMLKRGMPRVLSLIGLKRAMLSLIDGKGPIRFVLALLAFFRFTAIAPTRAVLDRWRGVNKQTAMKHLLSFKKELGTLTSAINRRSSKQKKRGGNTGIAVMIGLIASVGAVTLSNFQGKVMMTVNATDVTDVITIPTAAGKNLCIVRAMDVGYMCDDTITYECPVLSAGNDPEDIDCWCTKSAVYVRYGRCTKTRHSRRSRRSLTVQTHGESTLANKKGAWMDSTKATRYLVKTESWILRNPGYALVAAVIGWMLGSNTMQRVVFVVLLLLVAPAYSFNCLGMSNRDFLEGVSGATWVDLVLEGDSCVTIMSKDKPTIDVKMMNMEAANLAEVRSYCYLATVSDLSTKAACPTMGEAHNDKRADPAFVCRQGVVDRGWGNGCGLFGKGSIDTCAKFACSTKAIGRTILKENIKYEVAIFVHGPTTVESHGNYSTQAGATQAGRFSITPAAPSYTLKLGEYGEVTVDCEPRSGIDTNAYYVMTVGTKTFLVHREWFMDLNLPWSSAGSTVWRNRETLMEFEEPHATKQSVIALGSQEGALHQALAGAIPVEFSSNTVKLTSGHLKCRVKMEKLQLKGTTYGVCSKAFKFLGTPADTGHGTVVLELQYTGTDGPCKVPISSVASLNDLTPVGRLVTVNPFVSVATANAKVLIELEPPFGDSYIVVGRGEQQINHHWHKSGSSIGKAFTTTLKGAQRLAALGDTAWDFGSVGGVFTSVGKAVHQVFGGAFRSLFGGMSWITQGLLGALLLWMGINARDRSIALTFLAVGGVLLFLSVNVHADTGCAIDISRQELRCGSGVFIHNDVEAWMDRYKYYPETPQGLAKIIQKAHKEGVCGLRSVSRLEHQMWEAVKDELNTLLKENGVDLSVVVEKQEGMYKSAPKRLTATTEKLEIGWKAWGKSILFAPELANNTFVVDGPETKECPTQNRAWNSLEVEDFGFGLTSTRMFLKVRESNTTECDSKIIGTAVKNNLAIHSDLSYWIESRLNDTWKLERAVLGEVKSCTWPETHTLWGDGILESDLIIPVTLAGPRSNHNRRPGYKTQNQGPWDEGRVEIDFDYCPGTTVTLSESCGHRGPATRTTTESGKLITDWCCRSCTLPPLRYQTDSGCWYGMEIRPQRHDEKTLVQSQVNAYNADMIDPFQLGLLVVFLATQEVLRKRWTAKISMPAILIALLVLVFGGITYTDVLRYVILVGAAFAESNSGGDVVHLALMATFKIQPVFMVASFLKARWTNQENILLMLAAVFFQMAYHDARQILLWEIPDVLNSLAVAWMILRAITFTTTSNVVVPLLALLTPGLRCLNLDVYRILLLMVGIGSLIREKRSAAAKKKGASLLCLALASTGLFNPMILAAGLIACDPNRKRGWPATEVMTAVGLMFAIVGGLAELDIDSMAIPMTIAGLMFAAFVISGKSTDMWIERTADISWESDAEITGSSERVDVRLDDDGNFQLMNDPGAPWKIWMLRMVCLAISAYTPWAILPSVVGFWITLQYTKRGGVLWDTPSPKEYKKGDTTTGVYRIMTRGLLGSYQAGAGVMVEGVFHTLWHTTKGAALMSGEGRLDPYWGSVKEDRLCYGGPWKLQHKWNGQDEVQMIVVEPGKNVKNVQTKPGVFKTPEGEIGAVTLDFPTGTSGSPIVDKNGDVIGLYGNGVIMPNGSYISAIVQGERMDEPIPAGFEPEMLRKKQITVLDLHPGAGKTRRILPQIIKEAINRRLRTAVLAPTRVVAAEMAEALRGLPIRYQTSAVPREHNGNEIVDVMCHATLTHRLMSPHRVPNYNLFVMDEAHFTDPASIAARGYISTKVELGEAAAIFMTATPPGTSDPFPESNSPISDLQTEIPDRAWNSGYEWITEYTGKTVWFVPSVKMGNEIALCLQRAGKKVVQLNRKSYETEYPKCKNDDWDFVITTDISEMGANFKASRVIDSRKSVKPTIITEGEGRVILGEPSAVTAASAAQRRGRIGRNPSQVGDEYCYGGHTNEDDSNFAHWTEARIMLDNINMPNGLIAQFYQPEREKVYTMDGEYRLRGEERKNFLELLRTADLPVWLAYKVAAAGVSYHDRRWCFDGPRTNTILEDNNEVEVITKLGERKILRPRWIDARVYSDHQALKAFKDFASGKRSQIGLIEVLGKMPEHFMGKTWEALDTMYVVATAEKGGRAHRMALEELPDALQTIALIALLSVMTMGVFFLLMQRKGIGKIGLGGAVLGVATFFCWMAEVPGTKIAGMLLLSLLLMIVLIPEPEKQRSQTDNQLAVFLICVMTLVSAVAANEMGWLDKTKSDISSLFGQRIEVKENFSMGEFLLDLRPATAWSLYAVTTAVLTPLLKHLITSDYINTSLTSINVQASALFTLARGFPFVDVGVSALLLAAGCWGQVTLTVTVTAATLLFCHYAYMVPGWQAEAMRSAQRRTAAGIMKNAVVDGIVATDVPELERTTPIMQKKVGQIMLILVSLAAVVVNPSVKTVREAGILITAAAVTLWENGASSVWNATTAIGLCHIMRGGWLSCLSITWTLIKNMEKPGLKRGGAKGRTLGEVWKERLNQMTKEEFTRYRKEAIIEVDRSAAKHARKEGNVTGGHPVSRGTAKLRWLVERRFLEPVGKVIDLGCGRGGWCYYMATQKRVQEVRGYTKGGPGHEEPQLVQSYGWNIVTMKSGVDVFYRPSECCDTLLCDIGESSSSAEVEEHRTIRVLEMVEDWLHRGPREFCVKVLCPYMPKVIEKMELLQRRYGGGLVRNPLSRNSTHEMYWVSRASGNVVHSVNMTSQVLLGRMEKRTWKGPQYEEDVNLGSGTRAVGKPLLNSDTSKIKNRIERLRREYSSTWHHDENHPYRTWNYHGSYDVKPTGSASSLVNGVVRLLSKPWDTITNVTTMAMTDTTPFGQQRVFKEKVDTKAPEPPEGVKYVLNETTNWLWAFLAREKRPRMCSREEFIRKVNSNAALGAMFEEQNQWRSAREAVEDPKFWEMVDEEREAHLRGECHTCIYNMMGKREKKPGEFGKAKGSRAIWFMWLGARFLEFEALGFLNEDHWLGRKNSGGGVEGLGLQKLGYILREVGTRPGGKIYADDTAGWDTRITRADLENEAKVLELLDGEHRRLARAIIELTYRHKVVKVMRPAADGRTVMDVISREDQRGSGQVVTYALNTFTNLAVQLVRMMEGEGVIGPDDVEKLTKGKGPKVRTWLFENGEERLSRMAVSGDDCVVKPLDDRFATSLHFLNAMSKVRKDIQEWKPSTGWYDWQQVPFCSNHFTELIMKDGRTLVVPCRGQDELVGRARISPGAGWNVRDTACLAKSYAQMWLLLYFHRRDLRLMANAICSAVPVNWVPTGRTTWSIHAGGEWMTTEDMLEVWNRVWIEENEWMEDKTPVEKWSDVPYSGKREDIWCGSLIGTRARATWAENIQVAINQVRAIIGDEKYVDYMSSLKRYEDTTLVEDTVL

>ADK62475.1 polyprotein [West Nile virus]

MSKKPGGPGKSRAVNMLKRGMPRVLSLIGLKRAMLSLIDGKGPIRFVLALLAFFRFTAIAPTRAVLDRWRGVNKQTAMKHLLSFKKELGTLTSAINRRSSKQKKRGGKTGIAVMIGLIASVGAVTLSNFQGKVMMTVNATDVTDVITIPTAAGKNLCIVRAMDVGYMCDDTITYECPVLSAGNDPEDIDCWCTKSAVYVRYGRCTKTRHSRRSRRSLTVQTHGESTLANKKGAWMDSTKATRYLVKTESWILRNPGYALVAAVIGWMLGSNTMQRVVFVVLLLLVAPAYSFNCLGMSNRDFLEGVSGATWVDLVLEGDSCVTIMSKDKPTIDVKMMNMEAANLAEVRSYCYLATVSDLSTKAACPTMGEAHNDKRADPAFVCRQGVVDRGWGNGCGLFGKGSIDTCAKFACSTKAIGRTILKENIKYEVAIFVHGPTTVESHGNYSTQAGATQAGRFSITPAAPSYTLKLGEYGEVTVDCEPRSGIDTNAYYVMTVGTKTFLVHREWFMDLNLPWSSAGSTVWRNRETLMEFEEPHATKQSVIALGSQEGALHQALAGAIPVEFSSNTVKLTSGHLKCRVKMEKLQLKGTTYGVCSKAFKFLGTPADTGHGTVVLELQYTGTDGPCKVPISSVASLNDLTPVGRLVTVNPFVSVATANAKVLIELEPPFGDSYIVVGRGEQQINHHWHKSGSSIGKAFTTTLKGAQRLAALGDTAWDFGSVGGVFTSVGKAVHQVFGGAFRSLFGGMSWITQGLLGALLLWMGINARDRSIALTFLAVGGVLLFLSVNVHADTGCAIDISRQELRCGSGVFIHNDVEAWMDRYKYYPETPQGLAKIIQKAHKEGVCGLRSVSRLEHQMWEAVKDELNTLLKENGVDLSVVVEKQEGMYKSAPKRLTATTEKLEIGWKAWGKSILFAPELANNTFVVDGPETKECPTQNRAWNSLEVEDFGFGLTSTRMFLKVRESNTTECDSKIIGTAVKNNLAIHSDLSYWIESRLNDTWKLERAVLGEVKSCTWPETHTLWGDGILESDLIIPVTLAGPRSNHNRRPGYKTQNQGPWDEGRVEIDFDYCPGTTVTLSESCGHRGPATRTTTESGKLITDWCCRSCTLPPLRYQTDSGCWYGMEIRPQRHDEKTLVQSQVNAYNADMIDPFQLGLLVVFLATQEVLRKRWTAKISMPAILIALLVLVFGGITYTDVLRYVILVGAAFAESNSGGDVVHLALMATFKIQPVFMVASFLKARWTNQENILLMLAAVFFQMAYHDARQILLWEIPDVLNSLAVAWMILRAITFTTTSNVVVPLLALLTPGLRCLNLDVYRILLLMVGIGSLIREKRSAAAKKKGASLLCLALASTGLFNPMILAAGLIACDPNRKRGWPATEVMTAVGLMFAIVGGLAELDIDSMAIPMTIAGLMFAAFVISGKSTDMWIERTADISWESDAEITGSSERVDVRLDDDGNFQLMNDPGAPWKIWMLRMVCLAISAYTPWAILPSVVGFWITLQYTKRGGVLWDTPSPKEYKKGDTTTGVYRIMTRGLLGSYQAGAGVMVEGVFHTLWHTTKGAALMSGEGRLDPYWGSVKEDRLCYGGPWKLQHKWNGQDEVQMIVVEPGKNVKNVQTKPGVFKTPEGEIGAVTLDFPTGTSGSPIVDKNGDVIGLYGNGVIMPNGSYISAIVQGERMDEPIPAGFEPEMLRKKQITVLDLHPGAGKTRRILPQIIKEAINRRLRTAVLAPTRVVAAEMAEALRGLPIRYQTSAVPREHNGNEIVDVMCHATLTHRLMSPHRVPNYNLFVMDEAHFTDPASIAARGYISTKVELGEAAAIFMTATPPGTSDPFPESNSPISDLQTEIPDRAWNSGYEWITEYTGKTVWFVPSVKMGNEIALCLQRAGKKVVQLNRKSYETEYPKCKNDDWDFVITTDISEMGANFKASRVIDSRKSVKPTIITEGEGRVILGEPSAVTAASAAQRRGRIGRNPSQVGDEYCYGGHTNEDDSNFAHWTEARIMLDNINMPNGLIAQFYQPEREKVYTMDGEYRLRGEERKNFLELLRTADLPVWLAYKVAAAGVSYHDRRWCFDGPRTNTILEDNNEVEVITKLGERKILRPRWIDARVYSDHQALKAFKDFASGKRSQIGLIEVLGKMPEHFMGKTWEALDTMYVVATAEKGGRAHRMALEELPDALQTIALIALLSVMTMGVFFLLMQRKGIGKIGLGGAVLGVATFFCWMAEVPGTKIAGMLLLSLLLMIVLIPEPEKQRSQTDNQLAVFLICVMTLVSAVAANEMGWLDKTKSDISSLFGQRIEVKENFSMGEFLLDLRPATAWSLYAVTTAVLTPLLKHLITSDYINTSLTSINVQASALFTLARGFPFVDVGVSALLLAAGCWGQVTLTVTVTAATLLFCHYAYMVPGWQAEAMRSAQRRTAAGIMKNAVVDGIVATDVPELERTTPIMQKKVGQIMLILVSLAAVVVNPSVKTVREAGILITAAAVTLWENGASSVWNATTAIGLCHIMRGGWLSCLSITWTLIKNMEKPGLKRGGAKGRTLGEVWKERLNQMTKEEFTRYRKEAIIEVDRSAAKHARKEGNVTGGHPVSRGTAKLRWLVERRFLEPVGKVIDLGCGRGGWCYYMATQKRVQEVRGYTKGGPGHEEPQLVQSYGWNIVTMKSGVDVFYRPSECCDTLLCDIGESSSSAEVEEHRTIRVLEMVEDWLHRGPREFCVKVLCPYMPKVIEKMELLQRRYGGGLVRNPLSRNSTHEMYWVSRASGNVVHSVNMTSQVLLGRMEKRTWKGPQYEEDVNLGSGTRAVGKPLLNSDTSKIKNRIERLRREYSSTWHHDENHPYRTWNYHGSYDVKPTGSASSLVNGVVRLLSKPWDTITNVTTMAMTDTTPFGQQRVFKEKVDTKAPEPPEGVKYVLNETTNWLWAFLAREKRPRMCSREEFIRKVNSNAALGAMFEEQNQWRSAREAVEDPKFWEMVDEEREAHLRGECHTCIYNMMGKREKKPGEFGKAKGSRAIWFMWLGARFLEFEALGFLNEDHWLGRKNSGGGVEGLGLQKLGYILREVGTRPGGKIYADDTAGWDTRITRADLENEAKVLELLDGEHRRLARAIIELTYRHKVVKVMRPAADGRTVMDVISREDQRGSGQVVTYALNTFTNLAVQLVRMMEGEGVIGPDDVEKLTKGKGPKVRTWLFENGEERLSRMAVSGDDCVVKPLDDRFATSLHFLNAMSKVRKDIQEWKPSTGWHDWQQVPFCSNHFTELIMKDGRTLVVPCRGQDELVGRARISPGAGWNVRDTACLAKSYAQMWLLLYFHRRDLRLMANAICSAVPVNWVPTGRTTWSIHAGGEWMTTEDMLEVWNRVWIEENEWMEDKTPVEKWSDVPYSGKREDIWCGSLIGTRARATWAENIQVAINQVRAIIGDEKYVDYMSSLKRYEDTTLVEDTVL

>AVC05642.1 polyprotein [West Nile virus]

MSKKPGGPGKSRAVNMLKRGMPRVLSLIGLKRAMLSLIDGKGPIRFVLALLAFFRFTAIAPTRAVLDRWRGVNKQTAMKHLLSFKKELGTLTSAVNRRSSKQKKRGGKTGIAVMIGLIASVGAVTLSNFQGKVMMTVNATDVTDVITIPTAAGKNLCIVRAMDVGYMCDDTITYECPVLSAGNDPEDIDCWCTKSAVYVRYGRCTKTRHSRRSRRSLTVQTHGESTLANKKGAWMDSTKATRYLVKTESWILRNPGYALVAAVIGWMLGSNTMQRVVFVVLLLLVAPAYSFNCLGMSNRDFLEGVSGATWVDLVLEGDSCVTIMSKDKPTIDVKMMNMEAANLAEVRSYCYLATVSDLSTKAACPTMGEAHNDKRADPAFVCRQGVVDRGWGNGCGLFGKGSIDTCAKFACSTKAIGRTILKENIKYEVAIFVHGPTTVESHGNYSTQAGATQAGRFSITPAAPSYTLKLGEYGEVTVDCEPRSGIDTNAYYVMTVGTKTFLVHREWFMDLNLPWSSAGSTVWRNRETLMEFEEPHATKQSVIALGSQEGALHQALAGAIPVEFSSNTVKLTSGHLKCRVKMEKLQLKGTTYGVCSKAFKFLGTPADTGHGTVVLELQYTGTDGPCKVPISSVASLNDLTPVGRLVTVNPFVSVATANAKVLIELEPPFGDSYIVVGRGEQQINHHWHKSGSSIGKAFTTTLKGAQRLAALGDTAWDFGSVGGVFTSVGKAVHQVFGGAFRSLFGGMSWITQGLLGALLLWMGINARDRSIALTFLAVGGVLLFLSVNVHADTGCAIDISRQELRCGSGVFIHNDVEAWMDRYKYYPETPQGLAKIIQKAHKEGVCGLRSVSRLEHQMWEAVKDELNTLLKENGVDLSVVVEKQEGMYKSAPKRLTATTEKLEIGWKAWGKSILFAPELANNTFVVDGPETKECPTQNRAWNSLEVEDFGFGLTSTRMFLKVRESNTTECDSKIIGTAVKNNLAIHSDLSYWIESRLNDTWKLERAVLGEVKSCTWPETHTLWGDGILESDLIIPVTLAGPRSNHNRRPGYKTQNQGPWDEGRVEIDFDYCPGTTVTLSESCGHRGPATRTTTESGKLITDWCCRSCTLPPLRYQTDSGCWYGMEIRPQRHDEKTLVQSQVNAYNADMIDPFQLGLLVVFLATQEVLRKRWTAKISMPAILIALLVLVFGGITYTDVLRYVILVGAAFAESNSGGDVVHLALMATFKIQPVFMVASFLKARWTNQENILLMLAAVFFQMAYHDARQILLWEIPDVLNSLAVAWMILRAITFTTTSNVVVPLLALLTPGLRCLNLDVYRILLLMVGIGSLIREKRSAAAKKKGASLLCLALASTGLFNPMILAAGLIACDPNRKRGWPATEVMTAVGLMFAIVGGLAELDIDSMAIPMTIAGLMFAAFVISGKSTDMWIERTADISWESDAEITGSSERVDVRLDDDGNFQLMNDPGAPWKIWMLRMVCLAISAYTPWAILPSVVGFWITLQYTKRGGVLWDTPSPKEYKKGDTTTGVYRIMTRGLLGSYQAGAGVMVEGVFHTLWHTTKGAALMSGEGRLDPYWGSVKEDRLCYGGPWKLQHKWNGQDEVQMIVVEPGKNVKNVQTKPGVFKTPEGEIGAVTLDFPTGTSGSPIVDKNGDVIGLYGNGVIMPNGSYISAIVQGERMDEPIPAGFDPEMLRKKQITVLDLHPGAGKTRRILPQIIKEAINRRLRTAVLAPTRVVAAEMAEALRGLPIRYQTSAVPREHNGNEIVDVMCHATLTHRLMSPHRVPNYNLFVMDEAHFTDPASIAARGYISTKVELGEAAAIFMTATPPGTSDPFPESNSPISDLQTEIPDRAWNSGYEWITEYTGKTVWFVPSVKMGNEIALCLQRAGKKVVQLNRKSYETEYPKCKNDDWDFVITTDISEMGANFKASRVIDSRKSVKPTIITEGEGRVILGEPSAVTAASAAQRRGRIGRNPSQVGDEYCYGGHTNEDDSNFAHWTEARIMLDNINMPNGLIAQFYQPEREKVYTMDGEYRLRGEERKNFLELLRTADLPVWLAYKVAAAGVSYHDRRWCFDGPRTNTILEDNNEVEVITKLGERKILRPRWIDARVYSDHQALKAFKDFASGKRSQIGLIEVLGKMPEHFMGKTWEALDTMYVVATAEKGGRAHRMALEELPDALQTIALIALLSVMTMGVFFLLMQRKGIGKIGLGGAVLGVATFFCWMAEVPGTKIAGMLLLSLLLMIVLIPEPEKQRSQTDNQLAVFLICVMTLVSAVAANEMGWLDKTKSDISSLFGQRIEVKENFSMGEFLLDLRPATAWSLYAVTTAVLTPLLKHLITSDYINTSLTSINVQASALFTLARGFPFVDVGVSALLLAAGCWGQVTLTVTVTAATLLFCHYAYMVPGWQAEAMRSAQRRTAAGIMKNAVVDGIVATDVPELERTTPIMQKKVGQIMLILVSLAAVVVNPSVKTVREAGILITAAAVTLWENGASSVWNATTAIGLCHIMRGGWLSCLSITWTLIKNMEKPGLKRGGAKGRTLGEVWKERLNQMTKEEFTRYRKEAIIEVDRSAAKHARKEGNVTGGHPVSRGTAKLRWLVERRFLEPVGKVIDLGCGRGGWCYYMATQKRVQEVRGYTKGGPGHEEPQLVQSYGWNIVTMKSGVDVFYRPSECCDTLLCDIGESSSSAEVEEHRTIRVLEMVEDWLHRGPREFCVKVLCPYMPKVIEKMELLQRRYGGGLVRNPLSRNSTHEMYWVSRASGNVVHSVNMTSQVLLGRMEKRTWKGPQYEEDVNLGSGTRAVGKPLLNSDTSKIKNRIERLRREYSSTWHHDENHPYRTWNYHGSYDVKPTGSASSLVNGVVRLLSKPWDTITNVTTMAMTDTTPFGQQRVFKEKVDTKAPEPPEGVKYVLNETTNWLWAFLAREKRPRMCSREEFIRKVNSNAALGAMFEEQNQWRSAREAVEDPKFWEMVDEEREAHLRGECHTCIYNMMGKREKKPGEFGKAKGSRAIWFMWLGARFLEFEALGFLNEDHWLGRKNSGGGVEGLGLQKLGYILREVGTRPGGKIYADDTAGWDTRITRADLENEAKVLELLDGEHRRLARAIIELTYRHKVVKVMRPAADGRTVMDVISREDQRGSGQVVTYALNTFTNLAVQLVRMMEGEGVIGPDDVEKLTKGKGPKVRTWLFENGEERLSRMAVSGDDCVVKPLDDRFATSLHFLNAMSKVRKDIQEWKPSTGWYDWQQVPFCSNHFTELIMKDGRTLVVPCRGQDELVGRARISPGAGWNVRDTACLAKSYAQMWLLLYFHRRDLRLMANAICSAVPVNWVPTGRTTWSIHAGGEWMTTEDMLEVWNRVWIEENEWMEDKTPVEKWSDVPYSGKREDIWCGSLIGTRARATWAENIQVAINQVRAIIGDEKYVDYMSSLKRYEDTTLVEDTVL

>AHW48573.1 polyprotein [West Nile virus]

MSKKPGGPGKSRAVNMLKRGMPRVLSLIGLKRAMLSLIDGKGPIRFVLALLAFFRFTAIAPTRAVLDRWRGVNKQTAMKHLLSFKKELGTLTSAINRRSSKQKKRGGKTGIAVMIGLIASVGAVTLSNFQGKVMMTVNATDVTDVITIPTAAGKNLCIVRAMDVGYMCDDTITYECPVLSAGNDPEDIDCWCTKSAVYVRYGRCTKTRHSRRSRRSLTVQTHGESTLANKKGAWMDSTKATRYLVKTESWILRNPGYALVAAVIGWMLGSNTMQRVVFVVLLLLVAPAYSFNCLGMSNRDFLEGVSGATWVDLVLEGDSCVTIMSKDKPTIDVKMMNMEAANLAEVRSYCYLATVSDLSTKAACPTMGEAHNDKRADPAFVCRQGVVDRGWGNGCGLFGKGSIDTCAKFACSTKAIGRTILKENIKYEVAIFVHGPTTVESHGNYSTQAGATQAGRFSITPAAPSYTLKLGEYGEVTVDCEPRSGIDTNAYYVMTVGTKTFLVHREWFMDLNLPWSSAGSTVWRNRETLMEFEEPHATKQSVIALGSQEGALHQALAGAIPVEFSSNTVKLTSGHLKCRVKMEKLQLKGTTYGVCSKAFKFLGTPADTGHGTVVLELQYTGTDGPCKVPISSVASLNDLTPVGRLVTVNPFVSVATANAKVLIELEPPFGDSYIVVGRGEQQINHHWHKSGSSIGKAFTTTLKGAQRLAALGDTAWDFGSVGGVFTSVGKAVHQVFGGAFRSLFGGMSWITQGLLGALLLWMGINARDRSIALTFLAVGGVLLFLSVNVHADTGCAIDISRQELRCGSGVFIHNDVEAWMDRYKYYPETPQGLAKIIQKAHKEGVCGLRSVSRLEHQMWEAVKDELNTLLKENGVDLSVVVEKQEGMYKSAPKRLTATTEKLEIGWKAWGKSILFAPELANNTFVVDGPETKECPTQNRAWNSLEVEDFGFGLTSTRMFLKVRESNTTECDSKIIGTAVKNNLAIHSDLSYWIESRLNDTWKLERAVLGEVKSCTWPETHTLWGDGILESDLIIPVTLAGPRSNHNRRPGYKTQNQGPWDEGRVEIDFDYCPGTTVTLSESCGHRGPATRTTTESGKLITDWCCRSCTLPPLRYQTDSGCWYGMEIRPQRHDEKTLVQSQVNAYNADMIDPFQLGLLVVFLATQEVLRKRWTAKISMPAILIALLVLVFGGITYTDVLRYVILVGAAFAESNSGGDVVHLALMATFKIQPVFMVASFLKARWTNQENILLMLAAVFFQMAYHDARQILLWEIPDVLNSLAVAWMILRAITFTTTSNVVVPLLALLTPGLRCLNLDVYRILLLMVGIGSLIREKRSAAAKKKGASLLCLALASTGLFNPMILAAGLIACDPNRKRGWPATEVMTAVGLMFAIVGGLAELDIDSMAIPMTIAGLMFAAFVISGKSTDMWIERTADISWESDAEITGSSERVDVRLDDDGNFQLMNDPGAPWKIWMLRMVCLAISAYTPWAILPSVVGFWITLQYTKRGGVLWDTPSPKEYKKGDTTTGVYRIMTRGLLGSYQAGAGVMVEGVFHTLWHTTKGAALMSGEGRLDPYWGSVKEDRLCYGGPWKLQHKWNGQDEVQMIVVEPGKNVKNVQTKPGVFKTPEGEIGAVTLDFPTGTSGSPIVDKNGDVIGLYGNGVIMPNGSYISAIVQGERMDEPIPAGFEPEMLRKKQITVLDLHPGAGKTRRILPQIIKEAINRRLRTAVLAPTRVVAAEMAEALRGLPIRYQTSAVPREHNGNEIVDVMCHATLTHRLMSPHRVPNYNLFVMDEAHFTDPASIAARGYISTKVELGEAAAIFMTATPPGTSDPFPESNSPISDLQTEIPDRAWNSGYEWITEYTGKTVWFVPSVKMGNEIALCLQRAGKKVVQLNRKSYETEYPKCKNDEWDFVITTDISEMGANFKASRVIDSRKSVKPTIITEGEGRVILGEPSAVTAASAAQRRGRIGRNPSQVGDEYCYGGHTNEDDSNFAHWTEARIMLDNINMPNGLIAQFYQPEREKVYTMDGEYRLRGEERKNFLELLRTADLPVWLAYKVAAAGVSYHDRRWCFDGPRTNTILEDNNEVEVITKLGERKILRPRWIDARVYSDHQALKAFKDFASGKRSQIGLIEVLGKMPEHFMGKTWEALDTMYVVATAEKGGRAHRMALEELPDALQTIALIALLSVMTMGVFFLLMQRKGIGKIGLGGAVLGVATFFCWMAEVPGTKIAGMLLLSLLLMIVLIPEPEKQRSQTDNQLAVFLICVMTLVSAVAANEMGWLDKTKSDISSLFGQRIEVKENFSMGEFLLDLRPATAWSLYAVTTAVLTPLLKHLITSDYINTSLTSINVQASALFTLARGFPFVDVGVSALLLAAGCWGQVTLTVTVTAATLLFCHYAYMVPGWQAEAMRSAQRRTAAGIMKNAVVDGIVATDVPELERTTPIMQKKVGQIMLILVSLAAVVVNPSVKTVREAGILITAAAVTLWENGASSVWNATTAIGLCHIMRGGWLSCLSITWTLIKNMEKPGLKRGGAKGRTLGEVWKERLNQMTKEEFTRYRKEAIIEVDRSAAKHARKEGNVTGGHPVSRGTAKLRWLVERRFLEPVGKVIDLGCGRGGWCYYMATQKRVQEVRGYTKGGPGHEEPQLVQSYGWNIVTMKSGVDVFYRPSECCDTLLCDIGESSSSAEVEEHRTIRVLEMVEDWLHRGPREFCVKVLCPYMPKVIEKMELLQRRYGGGLVRNPLSRNSTHEMYWVSRASGNVVHSVNMTSQVLLGRMEKRTWKGPQYEEDVNLGSGTRAVGKPLLNSDTSKIKNRIERLRREYSSTWHHDENHPYRTWNYHGSYDVKPTGSASSLVNGVVRLLSKPWDTITNVTTMAMTDTTPFGQQRVFKEKVDTKAPEPPEGVKYVLNETTNWLWAFLAREKRPRMCSREEFIRKVNSNAALGAMFEEQNQWRSAREAVEDPKFWEMVDEEREAHLRGECHTCIYNMMGKREKKPGEFGKAKGSRAIWFMWLGARFLEFEALGFLNEDHWLGRKNSGGGVEGLGLQKLGYILREVGTRPGGKIYADDTAGWDTRITRADLENEAKVLELLDGEHRRLARAIIELTYRHKVVKVMRPAADGRTVMDVISREDQRGSGQVVTYALNTFTNLAVQLVRMMEGEGVIGPDDVEKLTKGKGPKVRTWLFENGEERLSRMAVSGDDCVVKPLDDRFATSLHFLNAMSKVRKDIQEWKPSTGWYDWQQVPFCSNHFTELIMKDGRTLVVPCRGQDELVGRARISPGAGWNVRDTACLAKSYAQMWLLLYFHRRDLRLMANAICSAVPVNWVPTGRTTWSIHAGGEWMTTEDMLEVWNRVWIEENEWMEDKTPVEKWSDVPYSGKREDIWCGSLIGTRARATWAENIQVAINQVRAIIGDEKYVDYMSSLKRYEDTTLVEDTVL

>AGW25450.1 polyprotein [West Nile virus]

MSKKPGGPGKSRAVNMLKRGMPRVLSLIGLKRAMLSLIDGKGPIRFVLALLAFFRFTAIAPTRAVLDRWRGVNKQTAMKHLLSFKKELGTLTSAINRRSSKQKKRGGKTGIAVMIGLIASVGAVTLSNFQGKVMMTVNATDVTDVITIPTAAGKNLCIVRAMDVGYMCDDTITYECPVLSAGNDPEDIDCWCTKSAVYVRYGRCTKTRHSRRSRRSLTVQTHGESTLANKKGAWMDSTKATRYLVKTESWILRNPGYALVAAVIGWMLGSNTMQRVVFVVLLLLVAPAYSFNCLGMSNRDFLEGVSGATWVDLVLEGDSCVTIMSKDKPTIDVKMMNMEAANLAEVRSYCYLATVSDLSTKAACPTMGEAHNDKRADPAFVCRQGVVDRGWGNGCGLFGKGSIDTCAKFACSTKAIGRTILKENIKYEVAIFVHGPTTVESHGNYSTQAGATQAGRFSITPAAPSYTLKLGEYGEVTVDCEPRSGIDTNAYYVMTVGTKTFLVHREWFMDLNLPWSSAGSTVWRNRETLMEFEEPHATKQSVIALGSQEGALHQALAGAIPVEFSSNTVKLTSGHLKCRVKMEKLQLKGTTYGVCSKAFKFLGTPADTGHGTVVLELQYTGTDGPCKVPISSVASLNDLTPVGRLVTVNPFVSVATANAKVLIELEPPFGDSYIVVGRGEQQINHHWHKSGSSIGKAFTTTLKGAQRLAALGDTAWDFGSVGGVFTSVGKAVHQVFGGAFRSLFGGMSWITQGLLGALLLWMGINARDRSIALTFLAVGGVLLFLSVNVHADTGCAIDISRQELRCGSGVFIHNDVEAWMDRYKYYPETPQGLAKIIQKAHKEGVCGLRSVSRLEHQMWEAVKDELNTLLKENGVDLSVVVEKQEGMYKSAPKRLTATTEKLEIGWKAWGKSILFAPELANNTFVVDGPETKECPTQNRAWNSLEVEDFGFGLTSTRMFLKVRESNTTECDSKIIGTAVKNNLAIHSDLSYWIESRLNDTWKLERAVLGEVKSCTWPETHTLWGDGILESDLIIPVTLAGPRSNHNRRPGYKTQNQGPWDEGRVEIDFDYCPGTTVTLSESCGHRGPATRTTTESGKLITDWCCRSCTLPPLRYQTDSGCWYGMEIRPQRHDEKTLVQSQVNAYNADMIDPFQLGLLVVFLATQEVLRKRWTAKISMPAILIALLVLVFGGITYTDVLRYVILVGAAFAESNSGGDVVHLALMATFKIQPVFMVASFLKARWTNQENILLMLAAVFFQMAYHDARQILLWEIPDVLNSLAVAWMILRAITFTTTSNVVVPLLALLTPGLRCLNLDVYRILLLMVGIGSLIREKRSAAAKKKGASLLCLALASTGLFNPMILAAGLIACDPNRKRGWPATEVMTAVGLMFAIVGGLAELDVDSMAIPMTIAGLMFAAFVISGKSTDMWIERTADISWESDAEITGSSERVDVRLDDDGNFQLMNDPGAPWKIWMLRMVCLAISAYTPWAILPSVVGFWITLQYTKRGGVLWDTPSPKEYKKGDTTTGVYRIMTRGLLGSYQAGAGVMVEGVFHTLWHTTKGAALMSGEGRLDPYWGSVKEDRLCYGGPWKLQHKWNGQDEVQMIVVEPGKNVKNVQTKPGVFKTPEGEIGAVTLDFPTGTSGSPIVDKNGDVIGLYGNGVIMPNGSYISAIVQGERMDEPIPAGFDPEMLRKKQITVLDLHPGAGKTRRILPQIIKEAINRRLRTAVLAPTRVVAAEMAEALRGLPIRYQTSAVPREHNGNEIVDVMCHATLTHRLMSPHRVPNYNLFVMDEAHFTDPASIAARGYISTKVELGEAAAIFMTATPPGTSDPFPESNSPISDLQTEIPDRAWNSGYEWITEYTGKTVWFVPSVKMGNEIALCLQRAGKKVVQLNRKSYETEYPKCKNDDWDFVITTDISEMGANFKASRVIDSRKSVKPTIITEGEGRVILGEPSAVTAASAAQRRGRIGRNPSQVGDEYCYGGHTNEDDSNFAHWTEARIMLDNINMPNGLIAQFYQPEREKVYTMDGEYRLRGEERKNFLELLRTADLPVWLAYKVAAAGVSYHDRRWCFDGPRTNTILEDNNEVEVITKLGERKILRPRWIDARVYSDHQALKAFKDFASGKRSQIGLIEVLGKMPEHFMGKTWEALDTMYVVATAEKGGRAHRMALEELPDALQTIALIALLSVMTMGVFFLLMQRKGIGKIGLGGAVLGVATFFCWMAEVPGTKIAGMLLLSLLLMIVLIPEPEKQRSQTDNQLAVFLICVMTLVSAVAANEMGWLDKTKSDISSLFGQRIEVKENFSMGEFLLDLRPATAWSLYAVTTAVLTPLLKHLITSDYINTSLTSINVQASALFTLARGFPFVDVGVSALLLAAGCWGQVTLTVTVTAATLLFCHYAYMVPGWQAEAMRSAQRRTAAGIMKNAVVDGIVATDVPELERTTPIMQKKVGQIMLILVSLAAVVVNPSVKTVREAGILITAAAVTLWENGASSVWNATTAIGLCHIMRGGWLSCLSITWTLIKNMEKPGLKRGGAKGRTLGEVWKERLNQMTKEEFTRYRKEAIIEVDRSAAKHARKEGNVTGGHPVSRGTAKLRWLVERRFLEPVGKVIDLGCGRGGWCYYMATQKRVQEVRGYTKGGPGHEEPQLVQSYGWNIVTMKSGVDVFYRPSECCDTLLCDIGESSSSAEVEEHRTIRVLEMVEDWLHRGPREFCVKVLCPYMPKVIEKMELLQRRYGGGLVRNPLSRNSTHEMYWVSRASGNVVHSVNMTSQVLLGRMEKRTWKGPQYEEDVNLGSGTRAVGKPLLNSDTSKIKNRIERLRREYSSTWHHDENHPYRTWNYHGSYDVKPTGSASSLVNGVVRLLSKPWDTITNVTTMAMTDTTPFGQQRVFKEKVDTKAPEPPEGVKYVLNETTNWLWAFLAREKRPRMCSREEFIRKVNSNAALGAMFEEQNQWRSAREAVEDPKFWEMVDEEREAHLRGECHTCIYNMMGKREKKPGEFGKAKGSRAIWFMWLGARFLEFEALGFLNEDHWLGRKNSGGGVEGLGLQKLGYILREVGTRPGGKIYADDTAGWDTRITRADLENEAKVLELLDGEHRRLARAIIELTYRHKVVKVMRPAADGRTVMDVISREDQRGSGQVVTYALNTFTNLAVQLVRMMEGEGVIGPDDVEKLTKGKGPKVRTWLFENGEERLSRMAVSGDDCVVKPLDDRFATSLHFLNAMSKVRKDIQEWKPSTGWYDWQQVPFCSNHFTELIMKDGRTLVVPCRGQDELVGRARISPGAGWNVRDTACLAKSYAQMWLLLYFHRRDLRLMANAICSAVPVNWVPTGRTTWSIHAGGEWMTTEDMLEVWNRVWIEENEWMEDKTPVEKWSDVPYSGKREDIWCGSLIGTRARATWAENIQVAINQVRAIIGDEKYVDYMSSLKRYEDTTLVEDTVL

>AHW48789.1 polyprotein [West Nile virus]

MSKKPGGPGKSRAVNMLKRGMPRVLSLIGLKRAMLSLIDGKGPIRFVLALLAFFRFTAIAPTRAVLDRWRGVNKQTAMKHLLSFKKELGTLTSAINRRSSKQKKRGGKTGIAVMIGLIASVGAVTLSNFQGKVMMTVNATDVTDVITIPTAAGKNLCIVRAMDVGYMCDDTITYECPVLSAGNDPEDIDCWCTKSAVYVRYGRCTKTRHSRRSRRSLTVQTHGESTLANKKGAWMDSTKATRYLVKTESWILRNPGYALVAAVIGWMLGSNTMQRVVFVVLLLLVAPAYSFNCLGMSNRDFLEGVSGATWVDLVLEGDSCVTIMSKDKPTIDVKMMNMEAANLAEVRSYCYLATVSDLSTKAACPTMGEAHNDKRADPAFVCRQGVVDRGWGNGCGLFGKGSIDTCAKFACSTKAIGRTILKENIKYEVAIFVHGPTTVESHGNYSTQAGATQAGRFSITPAAPSYTLKLGEYGEVTVDCEPRSGIDTNAYYVMTVGTKTFLVHREWFMDLNLPWSSAGSTVWRNRETLMEFEEPHATKQSVIALGSQEGALHQALAGAIPVEFSSNTVKLTSGHLKCRVKMEKLQLKGTTYGVCSKAFKFLGTPADTGHGTVVLELQYTGTDGPCKVPISSVASLNDLTPVGRLVTVNPFVSVATANAKVLIELEPPFGDSYIVVGRGEQQINHHWHKSGSSIGKAFTTTLKGAQRLAALGDTAWDFGSVGGVFTSVGKAVHQVFGGAFRSLFGGMSWITQGLLGALLLWMGINARDRSIALTFLAVGGVLLFLSVNVHADTGCAIDISRQELRCGSGVFIHNDVEAWMDRYKYYPETPQGLAKIIQKAHKEGVCGLRSVSRLEHQMWEAVKDELNTLLKENGVDLSVVVEKQEGMYKSAPKRLTATTEKLEIGWKAWGKSILFAPELANNTFVVDGPETKECPTQNRAWNSLEVEDFGFGLTSTRMFLKVRESNTTECDSKIIGTAVKNNLAIHSDLSYWIESRLNDTWKLERAVLGEVKSCTWPETHTLWGDGILESDLIIPVTLAGPRSNHNRRPGYKTQNQGPWDEGRVEIDFDYCPGTTVTLSESCGHRGPATRTTTESGKLITDWCCRSCTLPPLRYQTDSGCWYGMEIRPQRHDEKTLVQSQVNAYNADMIDPFQLGLLVVFLATQEVLRKRWTAKISMPAILIALLVLVFGGITYTDVLRYVILVGAAFAESNSGGDVVHLALMATFKIQPVFMVASFLKARWTNQENILLMLAAVFFQMAYHDARQILLWEIPDVLNSLAVAWMILRAITFTTTSNVVVPLLALLTPGLRCLNLDVYRILLLMVGIGSLIREKRSAAAKKKGASLLCLALASTGLFNPMILAAGLIACDPNRKRGWPATEVMTAVGLMFAIVGGLAELDIDSMAIPMTIAGLMFAAFVISGKSTDMWIERTADISWESDAEITGSSERVDVRLDDDGNFQLMNDPGAPWKIWMLRMVCLAISAYTPWAILPSVVGFWITLQYTKRGGVLWDTPSPKEYKKGDTTTGVYRIMTRGLLGSYQAGAGVMVEGVFHTLWHTTKGAALMSGEGRLDPYWGSVKEDRLCYGGPWKLQHKWNGQDEVQMIVVEPGKNVKNVQTKPGVFKTPEGEIGAVTLDFPTGTSGSPIVDKNGDVIGLYGNGVIMPNGSYISAIVQGERMDEPIPAGFEPEMLRKKQITVLDLHPGAGKTRRILPQIIKEAINRRLRTAVLAPTRVVAAEMAEALRGLPIRYQTSAVPREHNGNEIVDVMCHATLTHRLMSPHRVPNYNLFVMDEAHFTDPASIAARGYISTKVELGEAAAIFMTATPPGTSDPFPESNSPISDLQTEIPDRAWNSGYEWITEYTGKTVWFVPSVKMGNEIALCLQRAGKKVVQLNRKSYETEYPKCKNDDWDFVITTDISEMGANFKASRVIDSRKSVKPTIITEGEGRVILGEPSAVTAASAAQRRGRIGRNPSQVGDEYCYGGHTNEDDSNFAHWTEARIMLDNINMPNGLIAQFYQPEREKVYTMDGEYRLRGEERKNFLELLRTADLPVWLAYKVAAAGVSYHDRRWCFDGPRTNTILEDNNEVEVITKLGERKILRPRWIDARVYSDHQALKAFKDFASGKRSQIGLIEVLGKMPEHFMGKTWEALDTMYVVATAEKGGRAHRMALEELPDALQTVALIALLSVMTMGVFFLLMQRKGIGKIGLGGAVLGVATFFCWMAEVPGTKIAGMLLLSLLLMIVLIPEPEKQRSQTDNQLAVFLICVMTLVSAVAANEMGWLDKTKSDISSLFGQRIEVKENFSMGEFLLDLRPATAWSLYAVTTAVLTPLLKHLITSDYINTSLTSINVQASALYTLARGFPFVDVGVSALLLAAGCWGQVTLTVTVTAATLLFCHYAYMVPGWQAEAMRSAQRRTAAGIMKNAVVDGIVATDVPELERTTPIMQKKVGQIMLILVSLAAVVVNPSVKTVREAGILITAAAVTLWENGASSVWNATTAIGLCHIMRGGWLSCLSITWTLIKNMEKPGLKRGGAKGRTLGEVWKERLNQMTKEEFTRYRKEAIIEVDRSAAKHARKEGNVTGGHPVSRGTAKLRWLVERRFLEPVGKVIDLGCGRGGWCYYMATQKRVQEVRGYTKGGPGHEEPQLVQSYGWNIVTMKSGVDVFYRPSECCDTLLCDIGESSSSAEVEEHRTIRVLEMVEDWLHRGPREFCVKVLCPYMPKVIEKMELLQRRYGGGLVRNPLSRNSTHEMYWVSRASGNVVHSVNMTSQVLLGRMEKRTWKGPQYEEDVNLGSGTRAVGKPLLNSDTSKIKNRIERLRREYSSTWHHDENHPYRTWNYHGSYDVKPTGSASSLVNGVVRLLSKPWDTITNVTTMAMTDTTPFGQQRVFKEKVDTKAPEPPEGVKYVLNETTNWLWAFLAREKRPRMCSREEFIRKVNSNAALGAMFEEQNQWRSAREAVEDPKFWEMVDEEREAHLRGECHTCIYNMMGKREKKPGEFGKAKGSRAIWFMWLGARFLEFEALGFLNEDHWLGRKNSGGGVEGLGLQKLGYILREVGTRPGGKIYADDTAGWDTRITRADLENEAKVLELLDGEHRRLARAIIELTYRHKVVKVMRPAADGRTVMDVISREDQRGSGQVVTYALNTFTNLAVQLVRMMEGEGVIGPDDVEKLTKGKGPKVRTWLFENGEERLSRMAVSGDDCVVKPLDDRFATSLHFLNAMSKVRKDIQEWKPSTGWYDWQQVPFCSNHFTELIMKDGRTLVVPCRGQDELVGRARISPGAGWNVRDTACLAKSYAQMWLLLYFHRRDLRLMANAICSAVPVNWVPTGRTTWSIHAGGEWMTTEDMLEVWNRVWIEENEWMEDKTPVEKWSDVPYSGKREDIWCGSLIGTRARATWAENIQVAINQVRAIIGDEKYVDYMSSLKRYEDTTLVEDTVL

>ANW69721.1 genome polyprotein [West Nile virus]

MSKKPGGPGKSRAVNMLKRGMPRVLSLIGLKRAMLSLIDGKGPIRFVLALLAFFRFTAIAPTRAVLDRWRGVNKQTAMKHLLSFKKELGTLTSAINRRSSKQKKRGGKTGIAVMIGLIASVGAVTLSNFQGKVMMTVNATDVTDVITIPTAAGKNLCIVRAMDVGYMCDDTITYECPVLSAGNDPEDIDCWCTKSAVYVRYGRCTKTRHSRRSRRSLTVQTHGESTLANKKGAWMDSTKATRYLVKTESWILRNPGYALVAAVIGWMLGSNTMQRVVFVVLLLLVAPAYSFNCLGMSNRDFLEGVSGATWVDLVLEGDSCVTIMSKDKPTIDVKMMNMEAANLAEVRSYCYLATVSDLSTKAACPTMGEAHNDKRADPAFVCRQGVVDRGWGNGCGLFGKGSIDTCAKFACSTKAVGRTILKENIKYEVAIFVHGPTTVESHGNYSTQAGATQAGRFSITPAAPSYTLKLGEYGEVTVDCEPRSGIDTNAYYVMTVGTKTFLVHREWFMDLNLPWSSAGSTVWRNRETLMEFEEPHATKQSVIALGSQEGALHQALAGAIPVEFSSNTVKLTSGHLKCRVKMEKLQLKGTTYGVCSKAFKFLGTPADTGHGTVVLELQYTGTDGPCKVPISSVASLNDLTPVGRLVTVNPFVSVATANAKVLIELEPPFGDSYIVVGRGEQQINHHWHKSGSSIGKAFTTTLKGAQRLAALGDTAWDFGSVGGVFTSVGKAVHQVFGGAFRSLFGGMSWITQGLLGALLLWMGINARDRSIALTFLAVGGVLLFLSVNVHADTGCAIDISRQELRCGSGVFIHNDVEAWMDRYKYYPETPQGLAKIIQKAHKEGVCGLRSVSRLEHQMWEAVKDELNTLLKENGVDLSVVVEKQEGMYKSAPKRLTATTEKLEIGWKAWGKSILFAPELANNTFVVDGPETKECPTQNRAWNSLEVEDFGFGLTSTRMFLKVRESNTTECDSKIIGTAVKNNLAIHSDLSYWIESRLNDTWKLERAVLGEVKSCTWPETHTLWGDGILESDLIIPVTLAGPRSNHNRRPGYKTQNQGPWDEGRVEIDFDYCPGTTVTLSESCGHRGPATRTTTESGKLITDWCCRSCTLPPLRYQTDSGCWYGMEIRPQRHDEKTLVQSQVNAYNADMIDPFQLGLLVVFLATQEVLRKRWTAKISMPAILIALLVLVFGGITYTDVLRYVILVGAAFAESNSGGDVVHLALMATFKIQPVFMVASFLKARWTNQENILLMLAAVFFQMAYHDARQILLWEIPDVLNSLAVAWMILRAITFTTTSNVVVPLLALLTPGLRCLNLDVYRILLLMVGIGSLIREKRSAAAKKKGASLLCLALASTGLFNPMILAAGLIACDPNRKRGWPATEVMTAVGLMFAIVGGLAELDIDSMAIPMTIAGLMFAAFVISGKSTDMWIERTADISWESDAEITGSSERVDVRLDDDGNFQLMNDPGAPWKIWMLRMVCLAISAYTPWAILPSVVGFWITLQYTKRGGVLWDTPSPKEYKKGDTTTGVYRIMTRGLLGSYQAGAGVMVEGVFHTLWHTTKGAALMSGEGRLDPYWGSVKEDRLCYGGPWKLQHKWNGQDEVQMIVVEPGKNVKNVQTKPGVFKTPEGEIGAVTLDFPTGTSGSPIVDKNGDVIGLYGNGVIMPNGSYISAIVQGERMDEPIPAGFEPEMLRKKQITVLDLHPGAGKTRRILPQIIKEAINRRLRTAVLAPTRVVAAEMAEALRGLPIRYQTSAVPREHNGNEIVDVMCHATLTHRLMSPHRVPNYNLFVMDEAHFTDPASIAARGYISTKVELGEAAAIFMTATPPGTSDPFPESNSPISDLQTEIPDRAWNSGYEWITEYTGKTVWFVPSVKMGNEIALCLQRAGKKVVQLNRKSYETEYPKCKNDDWDFVITTDISEMGANFKASRVIDSRKSVKPTIITEGEGRVILGEPSAVTAASAAQRRGRIGRNPSQVGDEYCYGGHTNEDDSNFAHWTEARIMLDNINMPNGLIAQFYQPEREKVYTMDGEYRLRGEERKNFLELLRTADLPVWLAYKVAAAGVSYHDRRWCFDGPRTNTILEDNNEVEVITKLGERKILRPRWIDARVYSDHQALKAFKDFASGKRSQIGLIEVLGKMPEHFMGKTWEALDTMYVVATAEKGGRAHRMALEELPDALQTIALIALLSVMTMGVFFLLMQRKGIGKIGLGGAVLGVATFFCWMAEVPGTKIAGMLLLSLLLMIVLIPEPEKQRSQTDNQLAMFLICVMTLVSAVAANEMGWLDKTKSDISSLFGQRIEVKENFSMGEFLLDLRPATAWSLYAVTTAVLTPLLKHLITSDYINTSLTSINVQASALFTLARGFPFVDVGVSALLLAAGCWGQVTLTVTVTAATLLFCHYAYMVPGWQAEAMRSAQRRTAAGIMKNAVVDGIVATDVPELERTTPIMQKKVGQIMLILVSLAAVVVNPSVKTVREAGILITAAAVTLWENGASSVWNATTAIGLCHIMRGGWLSCLSITWTLIKNMEKPGLKRGGAKGRTLGEVWKERLNQMTKEEFTRYRKEAIIEVDRSAAKHARKEGNVTGGHPVSRGTAKLRWLVERRFLEPVGKVIDLGCGRGGWCYYMATQKRVQEVRGYTKGGPGHEEPQLVQSYGWNIVTMKSGVDVFYRPSECCDTLLCDIGESSSSAEVEEHRTIRVLEMVEDWLHRGPREFCVKVLCPYMPKVIEKMELLQRRYGGGLVRNPLSRNSTHEMYWVSRASGNVVHSVNMTSQVLLGRMEKRTWKGPQYEEDVNLGSGTRAVGKPLLNSDTSKIKNRIERLRREYSSTWHHDENHPYRTWNYHGSYDVKPTGSASSLVNGVVRLLSKPWDTITNVTTMAMTDTTPFGQQRVFKEKVDTKAPEPPEGVKYVLNETTNWLWAFLAREKRPRMCSREEFIRKVNSNAALGAMFEEQNQWRSAREAVEDPKFWEMVDEEREAHLRGECHTCIYNMMGKREKKPGEFGKAKGSRAIWFMWLGARFLEFEALGFLNEDHWLGRKNSGGGVEGLGLQKLGYILREVGTRPGGKIYADDTAGWDTRITRADLENEAKVLELLDGEHRRLARAIIELTYRHKVVKVMRPAADGRTVMDVISREDQRGSGQVVTYALNTFTNLAVQLVRMMEGEGVIGPDDVEKLTKGKGPKVRTWLFENGEERLSRMAVSGDDCVVKPLDDRFATSLHFLNAMSKVRKDIQEWKPSTGWYDWQQVPFCSNHFTELIMKDGRTLVVPCRGQDELVGRARISPGAGWNVRDTACLAKSYAQMWLLLYFHRRDLRLMANAICSAVPVNWVPTGRTTWSIHAGGEWMTTEDMLEVWNRVWIEENEWMEDKTPVEKWSDVPYSGKREDIWCGSLIGTRARATWAENIQVAINQVRAIIGDEKYVDYMSSLKRYEDTTLVEDTVL

>ABA54588.1 polyprotein precursor [West Nile virus]

MSKKPGGPGKSRAVNMLKRGMPRVLSLIGLKRAMLSLIDGKGPIRFVLALLAFFRFTAIAPTRAVLDRWRGVNKQTAMKHLLSFKKELGTLTSAINRRSSKQKKRGGKTGIAVMIGLIASVGAVTLSNFQGKVMMTVNATDVTDVITIPTAAGKNLCIVRAMDVGYMCDDTITYECPVLSAGNDPEDIDCWCTKSAVYVRYGRCTKTRHSRRSRRSLTVQTHGESTLANKKGAWMDSTKATRYLVKTESWILRNPGYALVAAVIGWMLGSNTMQRVVFVVLLLLVAPAYSFNCLGMSNRDFLEGVSGATWVDLVLEGDSCVTIMSKDKPTIDVKMMNMEAANLAEVRSYCYLATVSDLSTKAACPTMGEAHNDKRADPAFVCRQGVVDRGWGNGCGLFGKGSIDTCAKFACSTKAIGRTILKENIKYEVAIFVHGPTTVESHGNYSTQAGATQAGRFSITPAAPSYTLKLGEYGEVTVDCEPRSGIDTNAYYVMTVGTKTFLVHREWFMDLNLPWSSAGSTVWRNRETLMEFEEPHATKQSVIALGSQEGALHQALAGAIPVEFSSNTVKLTSGHLKCRVKMEKLQLKGTTYGVCSKAFKFLGTPADTGHGTVVLELQYTGTDGPCKVPISSVASLNDLTPVGRLVTVNPFVSVATANAKVLIELEPPFGDSYIVVGRGEQQINHHWHKSGSSIGKAFTTTLKGAQRLAALGDTAWDFGSVGGVFTSVGKAVHQVFGGAFRSLFGGMSWITQGLLGALLLWMGINARDRSIALTFLAVGGVLLFLSVNVHADTGCAIDISRQELRCGSGVFIHNDVEAWMDRYKYYPETPQGLAKIIQKAHKEGVCGLRSVSRLEHQMWEAVKDELNTLLKENGVDLSVVVEKQEGMYKSAPKRLTATTEKLEIGWKAWGKSILFAPELANNTFVVDGPETKECPTQNRAWNSLEVEDFGFGLTSTRMFLKVRESNTTECDSKIIGTAVKNNLAIHSDLSYWIESRLNDTWKLERAVLGEVKSCTWPETHTLWGDGILESDLIIPVTLAGPRSNHNRRPGYKTQNQGPWDEGRVEIDFDYCPGTTVTLSESCGHRGPATRTTTESGKLITDWCCRSCTLPPLRYQTDSGCWYGMEIRPQRHDEKTLVQSQVNAYNADMIDPFQLGLLVVFLATQEVLRKRWTAKISMPAILIALLVLVFGGITYTDVLRYVILVGAAFAESNSGGDVVHLALMATFKIQPVFMVASFLKARWTNQENILLMLAAVFFQMAYHDARQILLWEIPDVLNSLAVAWMILRAITFTTTSNVVVPLLALLTPGLRCLNLDVYRILLLMVGIGSLIREKRSAAAKKKGASLLCLALASTGLFNPMILAAGLIACDPNRKRGWPATEVMTAVGLMFAIVGGLAELDIDSMAIPMTIAGLMFAAFVISGKSTDMWIERTADISWESDAEITGSSERVDVRLDDDGNFQLMNDPGAPWKIWMLRMVCLAISAYTPWAILPSVVGFWITLQYTKRGGVLWDTPSPKEYKKGDTTTGVYRIMTRGLLGSYQAGAGVMVEGVFHTLWHTTKGAALMSGEGRLDPYWGSVKEDRLCYGGPWKLQHKWNGQDEVQMIVVEPGKNVKNVQTKPGVFKTPEGEIGAVTLDFPTGTSGSPIVDKNGDVIGLYGNGVIMPNGSYISAIVQGERMDEPIPAGFEPEMLRKKQITVLDLHPGAGKTRRILPQIIKEAINRRLRTAVLAPTRVVAAEMAEALRGLPIRYQTSAVPREHNGNEIVDVMCHATLTHRLMSPHRVPNYNLFVMDEAHFTDPASIAARGYISTKVELGEAAAIFMTATPPGTSDPFPESNSPISDLQTEIPDRAWNSGYEWITEYTGKTVWFVPSVKMGNEIALCLQRAGKKVVQLNRKSYETEYPKCKNDDWDFVITTDISEMGANFKASRVIDSRKSVKPTIITEGEGRVILGEPSAVTAASAAQRRGRIGRNPSQVGDEYCYGGHTNEDDSNFAHWTEARIMLDNINMPNGLIAQFYQPEREKVYTMDGEYRLRGEERKNFLELLRTADLPVWLAYKVAAAGVSYHDRRWCFDGPRTNTILEDNNEVEVITKLGERKILRPRWIDARVYSDHQALKAFKDFASGKRSQIGLIEVLGKMPEHFMGKTWEALDTMYVVATAEKGGRAHRMALEELPDALQTIALIALLSVMTMGVFFLLMQRKGIGKIGLGGAVLGVATFFCWMAEVPGTKIAGMLLLSLLLMIVLIPEPEKQRSQTDNQLAVFLICVMTLVSAVAANEMGWLDKTKSDISSLFGQRIEVKENFSMGEFLLDLRPATAWSLYAVTTAVLTPLLKHLITSDYINTSLTSINVQASALFTLARGFPFVDVGVSALLLAAGCWGQVTLTVTVTAATLLFCHYAYMVPGWQAEAMRSAQRRTAAGIMKNAVVDGIVATDVPELERTTPIMQKKVGQIMLILVSLAAVVVNPSVKTVREAGILITAAAVTLWENGASSVWNATTAIGLCHIMRGGWLSCLSITWTLIKNMEKPGLKRGGAKGRTLGEVWKERLNQMTKEEFTRYRKEAIIEVDRSAAKHARKEGNVTGGHPVSRGTAKLRWLVERRFLEPVGKVIDLGCGRGGWCYYMATQKRVQEVRGYTKGGPGHEEPQLVQSYGWNIVTMKSGVDVFYRPSECCDTLLCDIGESSSSAEVEEHRTIRVLEMVEDWLHRGPREFCVKVLCPYMPKVIEKMELLQRRYGGGLVRNPLSRNSTHEMYWVSRASGNVVHSVNMTSQVLLGRMEKRTWKGPQYEEDVNLGSGTRAVGKPLLNSDTSKIKNRIERLRREYSSTWHHDENHPYRTWNYHGSYEVKPTGSASSLVNGVVRLLSKPWDTITNVTTMAMTDTTPFGQQRVFKEKVDTKAPEPPEGVKYVLNETTNWLWAFLAREKRPRMCSREEFIRKVNSNAALGAMFEEQNQWRSAREAVEDPKFWEMVDEEREAHLRGECHTCIYNMMGKREKKPGEFGKAKGSRAIWFMWLGARFLEFEALGFLNEDHWLGRKNSGGGVEGLGLQKLGYILREVGTRPGGKIYADDTAGWDTRITRADLENEAKVLELLDGEHRRLARAIIELTYRHKVVKVMRPAADGRTVMDVISREDQRGSGQVVTYALNTFTNLAVQLVRMMEGEGVIGPDDVEKLTKGKGPKVRTWLFENGEERLSRMAVSGDDCVVKPLDDRFATSLHFLNAMSKVRKDIQEWKPSTGWYDWQQVPFCSNHFTELIMKDGRTLVVPCRGQDELVGRARISPGAGWNVRDTACLAKSYAQMWLLLYFHRRDLRLMANAICSAVPVNWVPTGRTTWSIHAGGEWMTTEDMLEVWNRVWIEENEWMEDKTPVEKWSDVPYSGKREDIWCGSLIGTRARATWAENIQVAINQVRAIIGDEKYVDYMSSLKRYEDTTLVEDTV

>AAN85090.1 polyprotein precursor [West Nile virus]

MSKKPGGPGKSRAVNMLKRGMPRVLSLIGLKRAMLSLIDGKGPIRFVLALLAFFRFTAIAPTRAVLDRWRGVNKQTAMKHLLSFKKELGTLTSAINRRSSKQKKRGGKTGIAVMIGLIASVGAVTLSNFQGKVMMTVNATDVTDVITIPTAAGKNLCIVRAMDVGYMCDDTITYECPVLSAGNDPEDIDCWCTKSAVYVRYGRCTKTRHSRRSRRSLTVQTHGESTLANKKGAWMDSTKATRYLVKTESWILRNPGYALVAAVIGWMLGSNTMQRVVFVVLLLLVAPAYSFNCLGMSNRDFLEGVSGATWVDLVLEGDSCVTIMSKDKPTIDVKMMNMEAANLAEVRSYCYLATVSDLSTKAACPTMGEAHNDKRADPAFVCRQGVVDRGWGNGCGLFGKGSIDTCAKFACSTKAIGRTILKENIKYEVAIFVHGPTTVESHGNYSTQVGATQAGRFSITPAAPSYTLKLGEYGEVTVDCEPRSGIDTNAYYVMTVGTKTFLVHREWFMDLNLPWSSAGSTVWRNRETLMEFEEPHATKQSVIALGSQEGALHQALAGAIPVEFSSNTVKLTSGHLKCRVKMEKLQLKGTTYGVCSKAFKFLGTPADTGHGTVVLELQYTGTDGPCKVPISSVASLNDLTPVGRLVTVNPFVSVATANAKVLIELEPPFGDSYIVVGRGEQQINHHWHKSGSSIGKAFTTTLKGAQRLAALGDTAWDFGSVGGVFTSVGKAVHQVFGGAFRSLFGGMSWITQGLLGALLLWMGINARDRSIALTFLAVGGVLLFLSVNVHADTGCAIDISRQELRCGSGVFIHNDVEAWMDRYKYYPETPQGLAKIIQKAHKEGVCGLRSVSRLEHQMWEAVKDELNTLLKENGVDLSVVVEKQEGMYKSAPKRLTATTEKLEIGWKAWGKSILFAPELANNTFVVDGPETKECPTQNRAWNSLEVEDFGFGLTSTRMFLKVRESNTTECDSKIIGTAVKNNLAIHSDLSYWIESRLNDTWKLERAVLGEVKSCTWPETHTLWGDGILESDLIIPVTLAGPRSNHNRRPGYKTQNQGPWDEGRVEIDFDYCPGTTVTLSESCGHRGPATRTTTESGKLITDWCCRSCTLPPLRYQTDSGCWYGMEIRPQRHDEKTLVQSQVNAYNADMIDPFQLGLLVVFLATQEVLRKRWTAKISMPAILIALLVLVFGGITYTDVLRYVILVGAAFAESNSGGDVVHLALMATFKIQPVFVVASFLKARWTNQENILLMLAAVFFQMAYHDARQILLWEIPDVLNSLAVAWMILRAITFTTTSNVVVPLLALLTPGLRCLNLDVYRILLLMVGIGSLIREKRSAAAKKKGASLLCLALASTGLFNPMILAAGLIACDPNRKRGWPATEVMTAVGLMFAIVGGLAELDIDSMAIPMTIAGLMFAAFVISGKSTDMWIERTADISWESDAEITGSSERVDVRLDDDGNFQLMNDPGAPWKIWMLRMVCLAISAYTPWAILPSVVGFWITLQYTKRGGVLWDTPSPKEYKKGDTTTGVYRIMTRGLLGSYQAGAGVMVEGVFHTLWHTTKGAALMSGEGRLDPYWGSVKEDRLCYGGPWKLQHKWNGQDEVQMIVVEPGKNVKNVQTKPGVFKTPEGEIGAVTLDFPTGTSGSPIVDKNGDVIGLYGNGVIMPNGSYISAIVQGERMDEPIPAGFEPEMLRKKKITVLDLHPGAGKTRRILPQIIKEAINRRLRTAVLAPTRVVAAEMAEALRGLPIRYQTSAVPREHNGNEIVDVMCHATLTHRLMSPHRVPNYNLFVMDEAHFTDPASIAARGYISTKVELGEAAAIFMTATPPGTSDPFPESNSPISDLQTEIPDRAWNSGYEWITEYTGKTVWFVPSVKMGNEIALCLQRAGKKVVQLNRKSYETEYPKCKNDDWDFVITTDISEMGANFKASRVIDSRKSVKPTIITEGEGRVILGEPSAVTAASAAQRRGRIGRNPSQVGDEYCYGGHTNEDDSNFAHWTEARIMLDNINMPNGLIAQFYQPEREKVYTMDGEYRLRGEERKNFLELLRTADLPVWLAYKVAAAGVSYHDRRWCFDGPRTNTILEDNNEVEVITKLGERKILRPRWIDARVYSDHQALKAFKDFASGKRSQIGLIEVLGKMPEHFMGKTWEALDTMYVVATAEKGGRAHRMALEELPDALQTIALIALLSVMTMGVFFLLMQRKGIGKIGLGGAVLGVATFFCWMAEVPGTKIAGMLLLSLLLMIVLIPEPEKQRSQTDNQLAVFLICVMTLVSAVAANEMGWLDKTKSDISSLFGQRIEVKENFSMGEFLLDLRPATAWSLYAVTTAVLTPLLKHLITSDYINTSLTSINVQASALFTLARGFPFVDVGVSALLLAAGCWGQVTLTVTVTAATLLFCHYAYMVPGWQAEAMRSAQRRTAAGIMKNAVVDGIVATDVPELERTTPIMQKKVGQIMLILVSLAAVVVNPSVKTVREAGILITAAAVTLWENGASSVWNATTAIGLCHIMRGGWLSCLSITWTLIKNMEKPGLKRGGAKGRTLGEVWKERLNQMTKEEFTRYRKEAIIEVDRSAAKHARKEGNVTGGHPVSRGTAKLRWLVERRFLEPVGKVIDLGCGRGGWCYYMATQKRVQEVRGYTKGGPGHEEPQLVQSYGWNIVTMKSGVDVFYRPSECCDTLLCDIGESSSSAEVEEHRTIRVLEMVEDWLHRGPREFCVKVLCPYMPKVIEKMELLQRRYGGGLVRNPLSRNSTHEMYWVSRASGNVVHSVNMTSQVLLGRMEKRTWKGPQYEEDVNLGSGTRAVGKPLLNSDTSKIKNRIERLRREYSSTWHHDENHPYRTWNYHGSYDVKPTGSASSLVNGVVRLLSKPWDTITNVTTMAMTDTTPFGQQRVFKEKVDTKAPEPPEGVKYVLNETTNWLWAFLAREKRPRMCSREEFIRKVNSNAALGAMFEEQNQWRSAREAVEDPKFWEMVDEEREAHLRGECHTCIYNMMGKREKKPGEFGKAKGSRAIWFMWLGARFLEFEALGFLNEDHWLGRKNSGGGVEGLGLQKLGYILREVGTRPGGKIYADDTAGWDTRITRADLENEAKVLELLDGEHRRLARAIIELTYRHKVVKVMRPAADGRTVMDVISREDQRGSGQVVTYALNTFTNLAVQLVRMMEGEGVIGPDDVEKLTKGKGPKVRTWLFENGEERLSRMAVSGDDCVVKPLDDRFATSLHFLNAMSKVRKDIQEWKPSTGWYDWQQVPFCSNHFTELIMKDGRTLVVPCRGQDELVGRARISPGAGWNVRDTACLAKSYAQMWLLLYFHRRDLRLMANAICSAVPVNWVPTGRTTWSIHAGGEWMTTEDMLEVWNRVWIEENEWMEDKTPVEKWSDVPYSGKREDIWCGSLIGTRARATWAENIQVAINQVRAIIGDEKYVDYMSSLKRYEDTTLVEDTVL

>Q9Q6P4.2 RecName: Full=Genome polyprotein; Contains: RecName: Full=Peptide 2k; Contains: RecName: Full=Capsid protein C; AltName: Full=Core protein; Contains: RecName: Full=Protein prM; Contains: RecName: Full=Peptide pr; Contains: RecName: Full=Small envelope protein M; AltName: Full=Matrix protein; Contains: RecName: Full=Envelope protein E; Contains: RecName: Full=Non-structural protein 1; Short=NS1; Contains: RecName: Full=Non-structural protein 2A; Short=NS2A; Contains: RecName: Full=Serine protease subunit NS2B; AltName: Full=Flavivirin protease NS2B regulatory subunit; AltName: Full=Non-structural protein 2B; Contains: RecName: Full=Serine protease NS3; AltName: Full=Flavivirin protease NS3 catalytic subunit; AltName: Full=Non-structural protein 3; Contains: RecName: Full=Non-structural protein 4A; Short=NS4A; Contains: RecName: Full=Non-structural protein 4B; Short=NS4B; Contains: RecName: Full=RNA-directed RNA polymerase NS5; AltName: Full=NS5 [West Nile virus strain NY-99]

MSKKPGGPGKSRAVNMLKRGMPRVLSLIGLKRAMLSLIDGKGPIRFVLALLAFFRFTAIAPTRAVLDRWRGVNKQTAMKHLLSFKKELGTLTSAINRRSSKQKKRGGKTGIAVMIGLIASVGAVTLSNFQGKVMMTVNATDVTDVITIPTAAGKNLCIVRAMDVGYMCDDTITYECPVLSAGNDPEDIDCWCTKSAVYVRYGRCTKTRHSRRSRRSLTVQTHGESTLANKKGAWMDSTKATRYLVKTESWILRNPGYALVAAVIGWMLGSNTMQRVVFVVLLLLVAPAYSFNCLGMSNRDFLEGVSGATWVDLVLEGDSCVTIMSKDKPTIDVKMMNMEAANLAEVRSYCYLATVSDLSTKAACPTMGEAHNDKRADPAFVCRQGVVDRGWGNGCGLFGKGSIDTCAKFACSTKAIGRTILKENIKYEVAIFVHGPTTVESHGNYSTQVGATQAGRFSITPAAPSYTLKLGEYGEVTVDCEPRSGIDTNAYYVMTVGTKTFLVHREWFMDLNLPWSSAGSTVWRNRETLMEFEEPHATKQSVIALGSQEGALHQALAGAIPVEFSSNTVKLTSGHLKCRVKMEKLQLKGTTYGVCSKAFKFLGTPADTGHGTVVLELQYTGTDGPCKVPISSVASLNDLTPVGRLVTVNPFVSVATANAKVLIELEPPFGDSYIVVGRGEQQINHHWHKSGSSIGKAFTTTLKGAQRLAALGDTAWDFGSVGGVFTSVGKAVHQVFGGAFRSLFGGMSWITQGLLGALLLWMGINARDRSIALTFLAVGGVLLFLSVNVHADTGCAIDISRQELRCGSGVFIHNDVEAWMDRYKYYPETPQGLAKIIQKAHKEGVCGLRSVSRLEHQMWEAVKDELNTLLKENGVDLSVVVEKQEGMYKSAPKRLTATTEKLEIGWKAWGKSILFAPELANNTFVVDGPETKECPTQNRAWNSLEVEDFGFGLTSTRMFLKVRESNTTECDSKIIGTAVKNNLAIHSDLSYWIESRLNDTWKLERAVLGEVKSCTWPETHTLWGDGILESDLIIPVTLAGPRSNHNRRPGYKTQNQGPWDEGRVEIDFDYCPGTTVTLSESCGHRGPATRTTTESGKLITDWCCRSCTLPPLRYQTDSGCWYGMEIRPQRHDEKTLVQSQVNAYNADMIDPFQLGLLVVFLATQEVLRKRWTAKISMPAILIALLVLVFGGITYTDVLRYVILVGAAFAESNSGGDVVHLALMATFKIQPVFMVASFLKARWTNQENILLMLAAVFFQMAYHDARQILLWEIPDVLNSLAVAWMILRAITFTTTSNVVVPLLALLTPGLRCLNLDVYRILLLMVGIGSLIREKRSAAAKKKGASLLCLALASTGLFNPMILAAGLIACDPNRKRGWPATEVMTAVGLMFAIVGGLAELDIDSMAIPMTIAGLMFAAFVISGKSTDMWIERTADISWESDAEITGSSERVDVRLDDDGNFQLMNDPGAPWKIWMLRMVCLAISAYTPWAILPSVVGFWITLQYTKRGGVLWDTPSPKEYKKGDTTTGVYRIMTRGLLGSYQAGAGVMVEGVFHTLWHTTKGAALMSGEGRLDPYWGSVKEDRLCYGGPWKLQHKWNGQDEVQMIVVEPGKNVKNVQTKPGVFKTPEGEIGAVTLDFPTGTSGSPIVDKNGDVIGLYGNGVIMPNGSYISAIVQGERMDEPIPAGFEPEMLRKKQITVLDLHPGAGKTRRILPQIIKEAINRRLRTAVLAPTRVVAAEMAEALRGLPIRYQTSAVPREHNGNEIVDVMCHATLTHRLMSPHRVPNYNLFVMDEAHFTDPASIAARGYISTKVELGEAAAIFMTATPPGTSDPFPESNSPISDLQTEIPDRAWNSGYEWITEYTGKTVWFVPSVKMGNEIALCLQRAGKKVVQLNRKSYETEYPKCKNDDWDFVITTDISEMGANFKASRVIDSRKSVKPTIITEGEGRVILGEPSAVTAASAAQRRGRIGRNPSQVGDEYCYGGHTNEDDSNFAHWTEARIMLDNINMPNGLIAQFYQPEREKVYTMDGEYRLRGEERKNFLELLRTADLPVWLAYKVAAAGVSYHDRRWCFDGPRTNTILEDNNEVEVITKLGERKILRPRWIDARVYSDHQALKAFKDFASGKRSQIGLIEVLGKMPEHFMGKTWEALDTMYVVATAEKGGRAHRMALEELPDALQTIALIALLSVMTMGVFFLLMQRKGIGKIGLGGAVLGVATFFCWMAEVPGTKIAGMLLLSLLLMIVLIPEPEKQRSQTDNQLAVFLICVMTLVSAVAANEMGWLDKTKSDISSLFGQRIEVKENFSMGEFLLDLRPATAWSLYAVTTAVLTPLLKHLITSDYINTSLTSINVQASALFTLARGFPFVDVGVSALLLAAGCWGQVTLTVTVTAATLLFCHYAYMVPGWQAEAMRSAQRRTAAGIMKNAVVDGIVATDVPELERTTPIMQKKVGQIMLILVSLAAVVVNPSVKTVREAGILITAAAVTLWENGASSVWNATTAIGLCHIMRGGWLSCLSITWTLIKNMEKPGLKRGGAKGRTLGEVWKERLNQMTKEEFTRYRKEAIIEVDRSAAKHARKEGNVTGGHPVSRGTAKLRWLVERRFLEPVGKVIDLGCGRGGWCYYMATQKRVQEVRGYTKGGPGHEEPQLVQSYGWNIVTMKSGVDVFYRPSECCDTLLCDIGESSSSAEVEEHRTIRVLEMVEDWLHRGPREFCVKVLCPYMPKVIEKMELLQRRYGGGLVRNPLSRNSTHEMYWVSRASGNVVHSVNMTSQVLLGRMEKRTWKGPQYEEDVNLGSGTRAVGKPLLNSDTSKIKNRIERLRREYSSTWHHDENHPYRTWNYHGSYDVKPTGSASSLVNGVVRLLSKPWDTITNVTTMAMTDTTPFGQQRVFKEKVDTKAPEPPEGVKYVLNETTNWLWAFLAREKRPRMCSREEFIRKVNSNAALGAMFEEQNQWRSAREAVEDPKFWEMVDEEREAHLRGECHTCIYNMMGKREKKPGEFGKAKGSRAIWFMWLGARFLEFEALGFLNEDHWLGRKNSGGGVEGLGLQKLGYILREVGTRPGGKIYADDTAGWDTRITRADLENEAKVLELLDGEHRRLARAIIELTYRHKVVKVMRPAADGRTVMDVISREDQRGSGQVVTYALNTFTNLAVQLVRMMEGEGVIGPDDVEKLTKGKGPKVRTWLFENGEERLSRMAVSGDDCVVKPLDDRFATSLHFLNAMSKVRKDIQEWKPSTGWYDWQQVPFCSNHFTELIMKDGRTLVVPCRGQDELVGRARISPGAGWNVRDTACLAKSYAQMWLLLYFHRRDLRLMANAICSAVPVNWVPTGRTTWSIHAGGEWMTTEDMLEVWNRVWIEENEWMEDKTPVEKWSDVPYSGKREDIWCGSLIGTRARATWAENIQVAINQVRAIIGDEKYVDYMSSLKRYEDTTLVEDTVL

>AYV87321.1 polyprotein [West Nile virus]

MSKKPGGPGKSRAVNMLKRGMPRVLSLIGLKRAMLSLIDGKGPIRFVLALLAFFRFTAIAPTRAVLDRWRGVNKQTAMKHLLSFKKELGTLTSAINRRSSKQKKRGGKTGIAVMIGLIASVGAVTLSNFQGKVMMTVNATDVTDVITIPTAAGKNLCIVRAMDVGYMCDDTITYECPVLSAGNDPEDIDCWCTKSAVYVRYGRCTKTRHSRRSRRSLTVQTHGESTLANKKGAWMDSTKATRYLVKTESWILRNPGYALVAAVIGWMLGSNTMQRVVFVVLLLLVAPAYSFNCLGMSNRDFLEGVSGATWVDLVLEGDSCVTIMSKDKPTIDVKMMNMEAANLAEVRSYCYLATVSDLSTKAACPTMGEAHNDKRADPAFVCRQGVVDRGWGNGCGLFGKGSIDTCAKFACSTKAIGRTILKENIKYEVAIFVHGPTTVESHGNYSTQAGATQAGRFSITPAAPSYTLKLGEYGEVTVDCEPRSGIDTNAYYVMTVGTKTFLVHREWFMDLNLPWSSAGSTVWRNRETLMEFEEPHATKQSVIALGSQEGALHQALAGAIPVEFSSNTVKLTSGHLKCRVKMEKLQLKGTTYGVCSKAFKFLGTPADTGHGTVVLELQYTGTDGPCKVPISSVASLNDLTPVGRLVTVNPFVSVATANAKVLIELEPPFGDSYIVVGRGEQQINHHWHKSGSSIGKAFTTTLKGAQRLAALGDTAWDFGSVGGVFTSVGKAVHQVFGGAFRSLFGGMSWITQGLLGALLLWMGINARDRSIALTFLAVGGVLLFLSVNVHADTGCAIDISRQELRCGSGVFIHNDVEAWMDRYKYYPETPQGLAKIIQKAHKEGVCGLRSVSRLEHQMWEAVKDELNTLLKENGVDLSVVVEKQEGMYKSAPKRLTATTEKLEIGWKAWGKSILFAPELANNTFVVDGPETKECPTQNRAWNSLEVEDFGFGLTSTRMFLKVRESNTTECDSKIIGTAVKNNLAIHSDLSYWIESRLNDTWKLERAVLGEVKSCTWPETHTLWGDGILESDLIIPVTLAGPRSNHNRRPGYKTQNQGPWDEGRVEIDFDYCPGTTVTLSESCGHRGPATRTTTESGKLITDWCCRSCTLPPLRYQTDSGCWYGMEIRPQRHDEKTLVQSQVNAYNADMIDPFQLGLLVVFLATQEVLRKRWTAKISMPAILIALLVLVFGGITYTDVLRYVILVGAAFAESNSGGDVVHLALMATFKIQPVFMVASFLKARWTNQENILLMLAAVFFQMAYHDARQILLWEIPDVLNSLAVAWMILRAITFTTTSNVVVPLLALLTPGLRCLNLDVYRILLLMVGIGSLIKEKRSAAAKKKGASLLCLALASTGLFNPMILAAGLIACDPNRKRGWPATEVMTAVGLMFAIVGGLAELDIDSMAIPMTIAGLMFAAFVISGKSTDMWIERTADISWESDAEITGSSERVDVRLDDDGNFQLMNDPGAPWKIWMLRMVCLAISAYTPWAILPSVVGFWITLQYTKRGGVLWDTPSPKEYKKGDTTTGVYRIMTRGLLGSYQAGAGVMVEGVFHTLWHTTKGAALMSGEGRLDPYWGSVKEDRLCYGGPWKLQHKWNGQDEVQMIVVEPGKNVKNVQTKPGVFKTPEGEIGAVTLDFPTGTSGSPIVDKNGDVIGLYGNGVIMPNGSYISAIVQGERMDEPIPAGFEPEMLRKKQITVLDLHPGAGKTRRILPQIIKEAINRRLRTAVLAPTRVVAAEMAEALRGLPIRYQTSAVPREHNGNEIVDVMCHATLTHRLMSPHRVPNYNLFVMDEAHFTDPASIAARGYISTKVELGEAAAIFMTATPPGTSDPFPESNSPISDLQTEIPDRAWNSGYEWITEYTGKTVWFVPSVKMGNEIALCLQRAGKKVVQLNRKSYETEYPKCKNDDWDFVITTDISEMGANFKASRVIDSRKSVKPTIITEGEGRVILGEPSAVTAASAAQRRGRIGRNPSQVGDEYCYGGHTNEDDSNFAHWTEARIMLDNINMPNGLIAQFYQPEREKVYTMDGEYRLRGEERKNFLELLRTADLPVWLAYKVAAAGVSYHDRRWCFDGPRTNTILEDNNEVEVITKLGERKILRPRWIDARVYSDHQALKAFKDFASGKRSQIGLIEVLGKMPEHFMGKTWEALDTMYVVATAEKGGRAHRMALEELPDALQTIALIALLSVMTMGVFFLLMQRKGIGKIGLGGAVLGVATFFCWMAEVPGTKIAGMLLLSLLLMIVLIPEPEKQRSQTDNQLAVFLICVMTLVSAVAANEMGWLDKTKSDISSLFGQRIEVKENFSMGEFLLDLRPATAWSLYAVTTAVLTPLLKHLITSDYINTSLTSINVQASALFTLARGFPFVDVGVSALLLAAGCWGQVTLTVTVTAATLLFCHYAYMVPGWQAEAMRSAQRRTAAGIMKNAVVDGIVATDVPELERTTPIMQKKVGQIMLILVSLAAVVVNPSVKTVREAGILITAAAVTLWENGASSVWNATTAIGLCHIMRGGWLSCLSITWTLIKNMEKPGLKRGGAKGRTLGEVWKERLNQMTKEEFTRYRKEAIIEVDRSAAKHARKEGNVTGGHPVSRGTAKLRWLVERRFLEPVGKVIDLGCGRGGWCYYMATQKRVQEVRGYTKGGPGHEEPQLVQSYGWNIVTMKSGVDVFYRPSECCDTLLCDIGESSSSAEVEEHRTIRVLEMVEDWLHRGPREFCVKVLCPYMPKVIEKMELLQRRYGGGLVRNPLSRNSTHEMYWVSRASGNVVHSVNMTSQVLLGRMEKRTWKGPQYEEDVNLGSGTRAVGKPLLNSDTSKIKNRIERLRREYSSTWHHDENHPYRTWNYHGSYDVRPTGSASSLVNGVVRLLSKPWDTITNVTTMAMTDTTPFGQQRVFKEKVDTKAPEPPEGVKYVLNETTNWLWAFLAREKRPRMCSREEFIRKVNSNAALGAMFEEQNQWRSAREAVEDPKFWEMVDEEREAHLRGECHTCIYNMMGKREKKPGEFGKAKGSRAIWFMWLGARFLEFEALGFLNEDHWLGRKNSGGGVEGLGLQKLGYILREVGTRPGGKIYADDTAGWDTRITRADLENEAKVLELLDGEHRRLARAIIELTYRHKVVKVMRPAADGRTVMDVISREDQRGSGQVVTYALNTFTNLAVQLVRMMEGEGVIGPDDVEKLTKGKGPKVRTWLFENGEERLSRMAVSGDDCVVKPLDDRFATSLHFLNAMSKVRKDIQEWKPSTGWYDWQQVPFCSNHFTELIMKDGRTLVVPCRGQDELVGRARISPGAGWNVRDTACLAKSYAQMWLLLYFHRRDLRLMANAICSAVPVNWVPTGRTTWSIHAGGEWMTTEDMLEVWNRVWIEENEWMEDKTPVEKWSDVPYSGKREDIWCGSLIGTRARATWAENIQVAINQVRAIIGDEKYVDYMSSLKRYEDTTLVEDTVL

>ADK62457.1 polyprotein [West Nile virus]

MSKKPGGPGKSRAVNMLKRGMPRVLSLIGLKRAMLSLIDGKGPIRFVLALLAFFRFTAIAPTRAVLDRWRGVNKQTAMKHLLSFKKELGTLTSAINRRSSKQKKRGGKTGIAVMIGLIASVGAVTLSNFQGKVMMTVNATDVTDVITIPTAAGKNLCIVRAMDVGYMCDDTITYECPVLSAGNDPEDIDCWCTKSAVYVRYGRCTKTRHSRRSRRSLTVQTHGESTLANKKGAWMDSTKATRYLVKTESWILRNPGYALVAAVIGWILGSNTMQRVVFVVLLLLVAPAYSFNCLGMSNRDFLEGVSGATWVDLVLEGDSCVTIMSKDKPTIDVKMMNMEAANLAEVRSYCYLATVSDLSTKAACPTMGEAHNDKRADPAFVCRQGVVDRGWGNGCGLFGKGSIDTCAKFACSTKAIGRTILKENIKYEVAIFVHGPTTVESHGNYSTQAGATQAGRFSITPAAPSYTLKLGEYGEVTVDCEPRSGIDTNAYYVMTVGTKTFLVHREWFMDLNLPWSSAGSTVWRNRETLMEFEEPHATKQSVIALGSQEGALHQALAGAIPVEFSSNTVKLTSGHLKCRVKMEKLQLKGTTYGVCSKAFKFLGTPADTGHGTVVLELQYTGTDGPCKVPISSVASLNDLTPVGRLVTVNPFVSVATANAKVLIELEPPFGDSYIVVGRGEQQINHHWHKSGSSIGKAFTTTLKGAQRLAALGDTAWDFGSVGGVFTSVGKAVHQVFGGAFRSLFGGMSWITQGLLGALLLWMGVNARDRSIALTFLAVGGVLLFLSVNVHADTGCAIDISRQELRCGSGVFIHNDVEAWMDRYKYYPETPQGLAKIIQKAHKEGVCGLRSVSRLEHQMWEAVKDELNTLLKENGVDLSVVVEKQEGMYKSAPKRLTATTEKLEIGWKAWGKSILFAPELANNTFVVDGPETKECPTQNRAWNSLEVEDFGFGLTSTRMFLKVRESNTTECDSKIIGTAVKNNLAIHSDLSYWIESRLNDTWKLERAVLGEVKSCTWPETHTLWGDGILESDLIIPVTLAGPRSNHNRRPGYKTQNQGPWDEGRVEIDFDYCPGTTVTLSESCGHRGPATRTTTESGKLITDWCCRSCTLPPLRYQTDSGCWYGMEIRPQRHDEKTLVQSQVNAYNADMIDPFQLGLLVVFLATQEVLRKRWTAKISMPAILIALLVLVFGGITYTDVLRYVILVGAAFAESNSGGDVVHLALMATFKIQPVFMVASFLKARWTNQENILLMLAAVFFQMAYHDARQILLWEIPDVLNSLAVAWMILRAITFTTTSNVVVPLLALLTPGLRCLNLDVYRILLLMVGIGSLIREKRSAAAKKKGASLLCLALASTGLFNPMILAAGLIACDPNRKRGWPATEVMTAVGLMFAIVGGLAELDIDSMAIPMTIAGLMFAAFVISGKSTDMWIERTADISWESDAEITGSSERVDVRLDDDGNFQLMNDPGAPWKIWMLRMVCLAISAYTPWAILPSVVGFWITLQYTKRGGVLWDTPSPKEYKKGDTTTGVYRIMTRGLLGSYQAGAGVMVEGVFHTLWHTTKGAALMSGEGRLDPYWGSVKEDRLCYGGPWKLQHKWNGQDEVQMIVVEPGKNVKNVQTKPGVFKTPEGEIGAVTLDFPTGTSGSPIVDKNGDVIGLYGNGVIMPNGSYISAIVQGERMDEPIPAGFEPEMLRKKQITVLDLHPGAGKTRRILPQIIKEAINRRLRTAVLAPTRVVAAEMAEALRGLPIRYQTSAVPREHNGNEIVDVMCHATLTHRLMSPHRVPNYNLFVMDEAHFTDPASIAARGYISTKVELGEAAAIFMTATPPGTSDPFPESNSPISDLQTEIPDRAWNSGYEWITEYTGKTVWFVPSVKMGNEIALCLQRAGKKVVQLNRKSYETEYPKCKNDDWDFVITTDISEMGANFKASRVIDSRKSVKPTIITEGEGRVILGEPSAVTAASAAQRRGRIGRNPSQVGDEYCYGGHTNEDDSNFAHWTEARIMLDNINMPNGLIAQFYQPEREKVYTMDGEYRLRGEERKNFLELLRTADLPVWLAYKVAAAGVSYHDRRWCFDGPRTNTILEDNNEVEVITKLGERKILRPRWIDARVYSDHQALKAFKDFASGKRSQIGLIEVLGKMPEHFMGKTWEALDTMYVVATAEKGGRAHRMALEELPDALQTIALIALLSVMTMGVFFLLMQRKGIGKIGLGGAVLGVATFFCWMAEVPGTKIAGMLLLSLLLMIVLIPEPEKQRSQTDNQLAVFLICVMTLVSAVAANEMGWLDKTKSDISSLFGQRIEVKENFSMGEFLLDLRPATAWSLYAVTTAVLTPLLKHLITSDYINTSLTSINVQASALFTLARGFPFVDVGVSALLLAAGCWGQVTLTVTVTAATLLFCHYAYMVPGWQAEAMRSAQRRTAAGIMKNAVVDGIVATDVPELERTTPIMQKKVGQIMLILVSLAAVVVNPSVKTVREAGILITAAAVTLWENGASSVWNATTAIGLCHIMRGGWLSCLSITWTLIKNMEKPGLKRGGAKGRTLGEVWKERLNQMTKEEFTRYRKEAIIEVDRSAAKHARKEGNVTGGHPVSRGTAKLRWLVERRFLEPVGKVIDLGCGRGGWCYYMATQKRVQEVRGYTKGGPGHEEPQLVQSYGWNIVTMKSGVDVFYRPSECCDTLLCDIGESSSSAEVEEHRTIRVLEMVEDWLHRGPREFCVKVLCPYMPKVIEKMELLQRRYGGGLVRNPLSRNSTHEMYWVSRASGNVVHSVNMTSQVLLGRMEKRTWKGPQYEEDVNLGSGTRAVGKPLLNSDTSKIKNRIERLRREYSSTWHHDENHPYRTWNYHGSYDVKPTGSASSLVNGVVRLLSKPWDTITNVTTMAMTDTTPFGQQRVFKEKVDTKAPEPPEGVKYVLNETTNWLWAFLAREKRPRMCSREEFIRKVNSNAALGAMFEEQNQWRSAREAVEDPKFWEMVDEEREAHLRGECHTCIYNMMGKREKKPGEFGKAKGSRAIWFMWLGARFLEFEALGFLNEDHWLGRKNSGGGVEGLGLQKLGYILREVGTRPGGKIYADDTAGWDTRITRADLENEAKVLELLDGEHRRLARAIIELTYRHKVVKVMRPAADGRTVMDVISREDQRGSGQVVTYALNTFTNLAVQLVRMMEGEGVIGPDDVEKLTKGKGPKVRTWLFENGEERLSRMAVSGDDCVVKPLDDRFATSLHFLNAMSKVRKDIQEWKPSTGWYDWQQVPFCSNHFTELIMKDGRTLVVPCRGQDELVGRARISPGAGWNVRDTACLAKSYAQMWLLLYFHRRDLRLMANAICSAVPVNWVPTGRTTWSIHAGGEWMTTEDMLEVWNRVWIEENEWMEDKTPVEKWSDVPYSGKREDIWCGSLIGTRARATWAENIQVAINQVRAIIGDEKYVDYMSSLKRYEDTTLVEDTVL

>AHW48626.1 polyprotein [West Nile virus]

MSKKPGGPGKSRAVNMLKRGMPRVLSLIGLKRAMLSLIDGKGPIRFVLALLAFFRFTAIAPTRAVLDRWRGVNKQTAMKHLLSFKKELGTLTSAINRRSSKQKKRGGKTGIAVMIGLIASVGAVTLSNFQGKVMMTVNATDVTDVITIPTAAGKNLCIVRAMDVGYMCDDTITYECPVLSAGNDPEDIDCWCTKSAVYVRYGRCTKTRHSRRSRRSLTVQTHGESTLANKKGAWMDSTKATRYLVKTESWILRNPGYALVAAVIGWMLGSNTMQRVVFVVLLLLVAPAYSFNCLGMSNRDFLEGVSGATWVDLVLEGDSCVTIMSKDKPTIDVKMMNMEAANLAEVRSYCYLATVSDLSTKAACPTMGEAHNDKRADPAFVCRQGVVDRGWGNGCGLFGKGSIDTCAKFACSTKAIGRTILKENIKYEVAIFVHGPTTVESHGNYSTQVGATQAGRFSITPAAPSYTLKLGEYGEVTVDCEPRSGIDTNAYYVMTVGTKTFLVHREWFMDLNLPWSSAGSTVWRNRETLMEFEEPHATKQSVIALGSQEGALHQALAGAIPVEFSSNTVKLTSGHLKCRVKMEKLQLKGTTYGVCSKAFKFLGTPADTGHGTVVLELQYTGTDGPCKVPISSVASLNDLTPVGRLVTVNPFVSVATANAKVLIELEPPFGDSYIVVGRGEQQINHHWHKSGSSIGKAFTTTLKGAQRLAALGDTAWDFGSVGGVFTSVGKAVHQVFGGAFRSLFGGMSWITQGLLGALLLWMGINARDRSIALTFLAVGGVLLFLSVNVHADTGCAIDISRQELRCGSGVFIHNDVEAWMDRYKYYPETPQGLAKIIQKAHKEGVCGLRSVSRLEHQMWEAVKDELNTLLKENGVDLSVVVEKQEGMYKSAPKRLTATTEKLEIGWKAWGKSILFAPELANNTFVVDGPETKECPTQNRAWNSLEVEDFGFGLTSTRMFLKVRESNTTECDSKIIGTAVKNNLAIHSDLSYWIESRLNDTWKLERAVLGEVKSCTWPETHTLWGDGILESDLIIPVTLAGPRSNHNRRPGYKTQNQGPWDEGRVEIDFDYCPGTTVTLSESCGHRGPATRTTTESGKLITDWCCRSCTLPPLRYQTDSGCWYGMEIRPQRHDEKTLVQSQVNAYNADMIDPFQLGLLVVFLATQEVLRKRWTAKISMPAILIALLVLVFGGITYTDVLRYVILVGAAFAESNSGGDVVHLALMATFKIQPVFMVASFLKARWTNQENILLMLAAVFFQMAYHDARQILLWEIPDVLNSLAVAWMILRAITFTTTSNVVVPLLALLTPGLRCLNLDVYRILLLMVGIGSLIREKRSAAAKKKGASLLCLALASTGLFNPMILAAGLIACDPNRKRGWPATEVMTAVGLMFAIVGGLAELDIDSMAIPMTIAGLMFAAFVISGKSTDMWIERTADISWESDAEITGSSERVDVRLDDDGNFQLMNDPGAPWKIWMLRMVCLAISAYTPWAILPSVVGFWITLQYTKRGGVLWDTPSPKEYKKGDTTTGVYRIMTRGLLGSYQAGAGVMVEGVFHTLWHTTKGAALMSGEGRLDPYWGSVKEDRLCYGGPWKLQHKWNGQDEVQMIVVEPGKNVKNVQTKPGVFKTPEGEIGAVTLDFPTGTSGSPIVDKNGDVIGLYGNGVIMPNGSYISAIVQGERMDEPIPAGFEPEMLRKKQITVLDLHPGAGKTRRILPQIIKEAINRRLRTAVLAPTRVVAAEMAEALRGLPIRYQTSAVPREHNGNEIVDVMCHATLTHRLMSPHRVPNYNLFVMDEAHFTDPASIAARGYISTKVELGEAAAIFMTATPPGTSDPFPESNSPISDLQTEIPDRAWNSGYEWITEYTGKTVWFVPSVKMGNEIALCLQRAGKKVVQLNRKSYETEYPKCKNDDWDFVITTDISEMGANFKASRVIDSRKSVKPTIITEGEGRVILGEPSAVTAASAAQRRGRIGRNPSQVGDEYCYGGHTNEDDSNFAHWTEARIMLDNINMPNGLIAQFYQPEREKVYTMDGEYRLRGEERKNFLELLRTADLPVWLAYKVAAAGVSYHDRRWCFDGPRTNTILEDNNEVEVITKLGERKILRPRWIDARVYSDHQALKAFKDFASGKRSQIGLIEVLGKMPEHFMGKTWEALDTMYVVATAEKGGRAHRMALEELPDALQTIALIALLSVMTMGVFFLLMQRKGIGKIGLGGAVLGVATFFCWMAEVPGTKIAGMLLLSLLLMIVLIPEPEKQRSQTDNQLAVFLICVMTLVSAVAANEMGWLDKTKSDISSLFGQRIEVKENFSMGEFLLDLRPATAWSLYAVTTAVLTPLLKHLITSDYINTSLTSINVQASALFTLSRGFPFVDVGVSALLLAAGCWGQVTLTVTVTAATLLFCHYAYMVPGWQAEAMRSAQRRTAAGIMKNAVVDGIVATDVPELERTTPIMQKKVGQIMLILVSLAAVVVNPSVKTVREAGILITAAAVTLWENGASSVWNATTAIGLCHIMRGGWLSCLSITWTLIKNMEKPGLKRGGAKGRTLGEVWKERLNQMTKEEFTRYRKEAIIEVDRSAAKHARKEGNVTGGHPVSRGTAKLRWLVERRFLEPVGKVIDLGCGRGGWCYYMATQKRVQEVRGYTKGGPGHEEPQLVQSYGWNIVTMKSGVDVFYRPSECCDTLLCDIGESSSSAEVEEHRTIRVLEMVEDWLHRGPREFCVKVLCPYMPKVIEKMELLQRRYGGGLVRNPLSRNSTHEMYWVSQASGNVVHSVNMTSQVLLGRMEKRTWKGPQYEEDVNLGSGTRAVGKPLLNSDTSKIKNRIERLRREYSSTWHHDENHPYRTWNYHGSYDVKPTGSASSLVNGVVRLLSKPWDTITNVTTMAMTDTTPFGQQRVFKEKVDTKAPEPPEGVKYVLNETTNWLWAFLAREKRPRMCSREEFIRKVNSNAALGAMFEEQNQWRSAREAVEDPKFWEMVDEEREAHLRGECHTCIYNMMGKREKKPGEFGKAKGSRAIWFMWLGARFLEFEALGFLNEDHWLGRKNSGGGVEGLGLQKLGYILREVGTRPGGKIYADDTAGWDTRITRADLENEAKVLELLDGEHRRLARAIIELTYRHKVVKVMRPAADGRTVMDVISREDQRGSGQVVTYALNTFTNLAVQLVRMMEGEGVIGPDDVEKLTKGKGPKVRTWLFENGEERLSRMAVSGDDCVVKPLDDRFATSLHFLNAMSKVRKDIQEWKPSTGWYDWQQVPFCSNHFTELIMKDGRTLVVPCRGQDELVGRARISPGAGWNVRDTACLAKSYAQMWLLLYFHRRDLRLMANAICSAVPVNWVPTGRTTWSIHAGGEWMTTEDMLEVWNRVWIEENEWMEDKTPVEKWSDVPYSGKREDIWCGSLIGTRARATWAENIQVAINQVRAIIGDEKYVDYMSSLKRYEDTTLVEDTVL

>AYV87329.1 polyprotein [West Nile virus]

MSKKPGGPGKSRAVNMLKRGMPRVLSLIGLKRAMLSLIDGKGPIRFVLALLAFFRFTAIAPTRAVLDRWRGVNKQTAMKHLLSFKKELGTLTSAINRRSSKQKKRGGKTGIAVMIGLIASVGAVTLSNFQGKVMMTVNATDVTDVITIPTAAGKNLCIVRAMDVGYMCDDTITYECPVLSAGNDPEDIDCWCTKSAVYVRYGRCTKTRHSRRSRRSLTVQTHGESTLANKKGAWMDSTKATRYLVKTESWILRNPGYALVAAVIGWMLGSNTMQRVVFVVLLLLVAPAYSFNCLGMSNRDFLEGVSGATWVDLVLEGDSCVTIMSKDKPTIDVKMVNMEAANLAEVRSYCYLATVSDLSTKAACPTMGEAHNDKRADPAFVCRQGVVDRGWGNGCGLFGKGSIDTCAKFACSTKAIGRTILKENIKYEVAIFVHGPTTVESHGNYSTQVGATQAGRFSITPAAPSYTLKLGEYGEVTVDCEPRSGIDTNAYYVMTVGTKTFLVHREWFMDLNLPWSSAGSTVWRNRETLMEFEEPHATKQSVIALGSQEGALHQALAGAIPVEFSSNTVKLTSGHLKCRVKMEKLQLKGTTYGVCSKAFKFLGTPADTGHGTVVLELQYTGTDGPCKVPISSVASLNDLTPVGRLVTVNPFVSVATANAKVLIELEPPFGDSYIVVGRGEQQINHHWHKSGSSIGKAFTTTLKGAQRLAALGDTAWDFGSVGGVFTSVGKAVHQVFGGAFRSLFGGMSWITQGLLGALLLWMGINARDRSIALTFLAVGGVLLFLSVNVHADTGCAIDISRQELRCGSGVFIHNDVEAWMDRYKYYPETPQGLAKIIQKAHKEGVCGLRSVSRLEHQMWEAVKDELNTLLKENGVDLSVVVEKQEGMYKSAPKRLTATTEKLEIGWKAWGKSILFAPELANNTFVVDGPETKECPTQNRAWNSLEVEDFGFGLTSTRMFLKVRESNTTECDSKIIGTAVKNNLAIHSDLSYWIESRLNDTWKLERAVLGEIKSCTWPETHTLWGDGILESDLIIPVTLAGPRSNHNRRPGYKTQNQGPWDEGRVEIDFDYCPGTTVTLSESCGHRGPATRTTTESGKLITDWCCRSCTLPPLRYQTDSGCWYGMEIRPQRHDEKTLVQSQVNAYNADMIDPFQLGLLVVFLATQEVLRKRWTAKISMPAILIALLVLVFGGITYTDVLRYVILVGAAFAESNSGGDVVHLALMATFKIQPVFMVASFLKARWTNQENILLMLAAVFFQMAYHDARQILLWEIPDVLNSLAVAWMILRAITFTTTSNVVVPLLALLTPGLRCLNLDVYRILLLMVGIGSLIREKRSAAAKKKGASLLCLALASTGLFNPMILAAGLIACDPNRKRGWPATEVMTAVGLMFAIVGGLAELDIDSMAIPMTIAGLMFAAFVISGKSTDMWIERTADISWESDAEITGSSERVDVRLDDDGNFQLMNDPGAPWKIWMLRMVCLAISAYTPWAILPSVVGFWVTLQYTKRGGVLWDTPSPKEYKKGDTTTGVYRIMTRGLLGSYQAGAGVMVEGVFHTLWHTTKGAALMSGEGRLDPYWGSVKEDRLCYGGPWKLQHKWNGQDEVQMIVVEPGKNVKNVQTKPGVFKTPEGEIGAVTLDFPTGTSGSPIVDKNGDVIGLYGNGVIMPNGSYISAIVQGERMDEPIPAGFEPEMLRKKQITVLDLHPGAGKTRRILPQIIKEAINRRLRTAVLAPTRVVAAEMAEALRGLPIRYQTSAVPREHNGNEIVDVMCHATLTHRLMSPHRVPNYNLFVMDEAHFTDPASIAARGYISTKVELGEAAAIFMTATPPGTSDPFPESNSPISDLQTEIPDRAWNSGYEWITEYTGKTVWFVPSVKMGNEIALCLQRAGKKVVQLNRKSYETEYPKCKNDDWDFVITTDISEMGANFKASRVIDSRKSVKPTIITEGEGRVILGEPSAVTAASAAQRRGRIGRNPSQVGDEYCYGGHTNEDDSNFAHWTEARIMLDNINMPNGLIAQFYQPEREKVYTMDGEYRLRGEERKNFLELLRTADLPVWLAYKVAAAGVSYHDRRWCFDGPRTNTILEDNNEVEVITKLGERKILRPRWIDARVYSDHQALKAFKDFASGKRSQIGLIEVLGKMPEHFMGKTWEALDTMYVVATAEKGGRAHRMALEELPDALQTIALIALLSVMTMGVFFLLMQRKGIGKIGLGGAVLGVATFFCWMAEVPGTKIAGMLLLSLLLMIVLIPEPEKQRSQTDNQLAVFLICVMTLVSAVAANEMGWLDKTKSDISSLFGQRIEVKENFSMGEFLLDLRPATAWSLYAVTTAVLTPLLKHLITSDYINTSLTSINVQASALFTLARGFPFVDVGVSALLLAAGCWGQVTLTVTVTAATLLFCHYAYMVPGWQAEAMRSAQRRTAAGIMKNAVVDGIVATDVPELERTTPIMQKKVGQIMLILVSLAAVVVNPSVKTVREAGILITAAAVTLWENGASSVWNATTAIGLCHIMRGGWLSCLSITWTLVKNMEKPGLKRGGAKGRTLGEVWKERLNQMTKEEFTRYRKEAIIEVDRSAAKHARKEGNVTGGHPVSRGTAKLRWLVERRFLEPVGKVIDLGCGRGGWCYYMATQKRVQEVRGYTKGGPGHEEPQLVQSYGWNIVTMKSGVDVFYRPSECCDTLLCDIGESSSSAEVEEHRTIRVLEMVEDWLHRGPREFCVKVLCPYMPKVIEKMELLQRRYGGGLVRNPLSRNSTHEMYWVSRASGNVVHSVNMTSQVLLGRMEKRTWKGPQYEEDVNLGSGTRAVGKPLLNSDTSKIKNRIERLRREYSSTWHHDENHPYRTWNYHGSYDVKPTGSASSLVNGVVRLLSKPWDTITNVTTMAMTDTTPFGQQRVFKEKVDTKAPEPPEGVKYVLNETTNWLWAFLAREKRPRMCSREEFIRKVNSNAALGAMFEEQNQWRSAREAVEDPKFWEMVDEEREAHLRGECHTCIYNMMGKREKKPGEFGKAKGSRAIWFMWLGARFLEFEALGFLNEDHWLGRKNSGGGVEGLGLQKLGYILREIGTRPGGKIYADDTAGWDTRITRADLENEAKVLELLDGEHRRLARAIIELTYRHKVVKVMRPAADGRTVMDVISREDQRGSGQVVTYALNTFTNLAVQLVRMMEGEGVIGPDDVEKLTKGKGPKVRTWLFENGEERLSRMAVSGDDCVVKPLDDRFATSLHFLNAMSKVRKDIQEWKPSTGWYDWQQVPFCSNHFTELIMKDGRTLVVPCRGQDELVGRARISPGAGWNVRDTACLAKSYAQMWLLLYFHRRDLRLMANAICSAVPVNWVPTGRTTWSIHAGGEWMTTEDMLEVWNRVWIEENEWMEDKTPVEKWSDVPYSGKREDIWCGSLIGTRARATWAENIQVAINQVRAIIGDEKYVDYMSSLKRYEDTTLVEDTVL

>ANW68800.1 genome polyprotein [West Nile virus]

MSKKPGGPGKSRAVNMLKRGMPRVLSLIGLKRAMLSLIDGKGPIRFVLALLAFFRFTAIAPTRAVLDRWRGVNKQTAMKHLLSFKKELGTLTSAINRRSSKQKKRGGKTGIAVMIGLIASVGAVTLSNFQGKVMMTVNATDVTDVITIPTAAGKNLCIVRAMDVGYMCDDTITYECPVLSAGNDPEDIDCWCTKSAVYVRYGRCTKTRHSRRSRRSLTVQTHGESTLANKKGAWMDSTKATRYLVKTESWILRNPGYALVAAVIGWMLGSNTMQRVVFVVLLLLVAPAYSFNCLGMSNRDFLEGVSGATWVDLVLEGDSCVTIMSKDKPTIDVKMMNMEAANLAEVRSYCYLATVSDLSTKAACPTMGEAHNDKRADPAFVCRQGVVDRGWGNGCGLFGKGSIDTCAKFACSTKAIGRTILKENIKYEVAIFVHGPTTVESHGNYSTQAGATQAGRFSITPAAPSYTLKLGEYGEVTVDCEPRSGIDTNAYYVMTVGTKTFLVHREWFMDLNLPWSSAGSTVWRNRETLMEFEEPHATKQSVIALGSQEGALHQALAGAIPVEFSSNTVKLTSGHLKCRVKMEKLQLKGTTYGVCSKAFKFLGTPADTGHGTVVLELQYTGTDGPCKVPISSVASLNDLTPVGRLVTVNPFVSVATANAKVLIELEPPFGDSYIVVGRGEQQINHHWHKSGSSIGKAFTTTLKGAQRLAALGDTAWDFGSVGGVFTSVGKAVHQVFGGAFRSLFGGMSWITQGLLGALLLWMGINARDRSIALTFLAVGGVLLFLSVNVHADTGCAIDISRQELRCGSGVFIHNDVEAWMDRYKYYPETPQGLAKIIQKAHKEGVCGLRSVSRLEHQMWEAVKDELNTLLKENGVDLSVVVEKQEGMYKSAPKRLTATTEKLEIGWKAWGKSILFAPELANNTFVVDGPETKECPTQNRAWNSLEVEDFGFGLTSTRMFLKVRESNTTECDSKIIGTAVKNNLAIHSDLSYWIESRLNDTWKLERAVLGEVKSCTWPETHTLWGDGILESDLIIPVTLAGPRSNHNRRPGYKTQNQGPWDEGRVEIDFDYCPGTTVTLSESCGHRGPATRTTTESGKLITDWCCRSCTLPPLRYQTDSGCWYGMEIRPQRHDEKTLVQSQVNAYNADMIDPFQLGLLVVFLATQEVLRKRWTAKISMPAILIALLVLVFGGITYTDVLRYVILVGAAFAESNSGGDVVHLALMATFKIQPVFMVASFLKARWTNQENILLMLAAVFFQMAYHDARQILLWEIPDVLNSLAVAWMILRAITFTTTSNVVVPLLALLTPGLRCLNLDVYRILLLMVGIGSLIREKRSAAAKKKGASLLCLALASTGLFNPMILAAGLIACDPNRKRGWPATEVMTAVGLMFAIVGGLAELDIDSMAIPMTIAGLMFAAFVISGKSTDMWIERTADISWESDAEITGSSERVDVRLDDDGNFQLMNDPGAPWKIWMLRMVCLAISAYTPWAILPSVVGFWITLQYTKRGGVLWDTPSPKEYKKGDTTTGVYRIMTRGLLGSYQAGAGVMVEGVFHTLWHTTKGAALMSGEGRLDPYWGSVKEDRLCYGGPWKLQHKWNGQDEVQMIVVEPGKNVKNVQTKPGVFKTPEGEIGAVTLDFPTGTSGSPIVDKNGDVIGLYGNGVIMPNGSYISAIVQGERMDEPIPAGFEPEMLRKKQITVLDLHPGAGKTRRILPQIIKEAINRRLRTAVLAPTRVVAAEMAEALRGLPIRYQTSAVPREHNGNEIVDVMCHATLTHRLMSPHRVPNYNLFVMDEAHFTDPASIAARGYISTKVELGEAAAIFMTATPPGTSDPFPESNSPISDLQTEIPDRAWNSGYEWITEYTGKTVWFVPSVKMGNEIALCLQRAGKKVVQLNRKSYETEYPKCKNDDWDFVITTDISEMGANFKASRVIDSRKSVKPTIITEGEGRVILGEPSAVTAASAAQRRGRIGRNPSQVGDEYCYGGHTNEDDSNFAHWTEARIMLDNINMPNGLIAQFYQPEREKVYTMDGEYRLRGEERKNFLELLRTADLPVWLAYKVAAAGVSYHDRRWCFDGPRTNTILEDNNEVEVITKLGERKILRPRWIDARVYSDHQALKAFKDFASGKRSQIGLIEVLGKMPEHFMGKTWEALDTMYVVATAEKGGRAHRMALEELPDALQTIALIALLSVMTMGVFFLLMQRKGIGKIGFGGAVLGVATFFCWMAEVPGTKIAGMLLLSLLLMIVLIPEPEKQRSQTDNQLAVFLICVMTLVSAVAANEMGWLDKTKSDISSLFGQRIEVKENFSMGEFLLDLRPATAWSLYAVTTAVLTPLLKHLITSDYINTSLTSINVQASALFTLARGFPFVDVGVSALLLAAGCWGQVTLTVTVTAATLLFCHYAYMVPGWQAEAMRSAQRRTAAGIMKNAVVDGIVATDVPELERTTPIMQKKVGQIMLILVSLAAVVVNPSVKTVREAGILITAAAVTLWENGASSVWNATTAIGLCHIMRGGWLSCLSITWTLIKNMEKPGLKRGGAKGRTLGEVWKERLNQMTKEEFTRYRKEAIIEVDRSAAKHARKEGNVTGGHPVSRGTAKLRWLVERRFLEPVGKVIDLGCGRGGWCYYMATQKRVQEVRGYTKGGPGHEEPQLVQSYGWNIVTMKSGVDVFYRPSECCDTLLCDIGESSSSAEVEEHRTIRVLEMVEDWLHRGPREFCVKVLCPYMPKVIEKMELLQRRYGGGLVRNPLSRNSTHEMYWVSRASGNVVHSVNMTSQVLLGRMEKRTWKGPQYEEDVNLGSGTRAVGKPLLNSDTSKIKNRIERLRREYSSTWHHDENHPYRTWNYHGSYDVKPTGSASSLVNGVVRLLSKPWDTITNVTTMAMTDTTPFGQQRVFKEKVDTKAPEPPEGVKYVLNETTNWLWAFLAREKRPRMCSREEFIRKVNSNAALGAMFEEQNQWRSAREAVEDPKFWEMVDEEREAHLRGECHTCIYNMMGKREKKPGEFGKAKGSRAIWFMWLGARFLEFEALGFLNEDHWLGRKNSGGGVEGLGLQKLGYILREVGTRPGGKIYADDTAGWDTRITRADLENEAKVLELLDGEHRRLARAIIELTYRHKVVKVMRPAADGRTVMDVISREDQRGSGQVVTYALNTFTNLAVQLVRMMEGEGVIGPDDVEKLTKGKGPKVRTWLFENGEERLSRMAVSGDDCVVKPLDDRFATSLHFLNAMSKVRKDIQEWKPSTGWYDWQQVPFCSNHFTELIMKDGRTLVVPCRGQDELVGRARISPGAGWNVRDTACLAKSYAQMWLLLYFHRRDLRLMANAICSAVPVNWVPTGRTTWSIHAGGEWMTTEDMLEVWNRVWIEENEWMEDKTPVEKWSDVPYSGKREDIWCGSLIGTRARATWAENIQVAINQVRAIIGDEKYVDYMSSLKRYEDTTLVEDTVL

>AHW48564.1 polyprotein [West Nile virus]

MSKKPGGPGKSRAVNMLKRGMPRVLSLIGLKRAMLSLIDGKGPIRFVLALLAFFRFTAIAPTRAVLDRWRGVNKQTAMKHLLSFKKELGTLTSAINRRSSKQKKRGGKTGIAVMIGLIASVGAVTLSNFQGKVMMTVNATDVTDVITIPTAAGKNLCIVRAMDVGYMCDDTITYECPVLSAGNDPEDIDCWCTKSAVYVRYGRCTKTRHSRRSRRSLTVQTHGESTLANKKGAWMDSTKATRYLVKTESWILRNPGYALVAAVIGWMLGSNTMQRVVFVVLLLLVAPAYSFNCLGMSNRDFLEGVSGATWVDLVLEGDSCVTIMSKDKPTIDVKMMNMEAANLAEVRSYCYLATVSDLSTKAACPTMGEAHNDKRADPAFVCRQGVVDRGWGNGCGLFGKGSIDTCAKFACSTKAIGRTILKENIKYEVAIFVHGPTTVESHGNYSTQAGATQAGRFSITPAAPSYTLKLGEYGEVTVDCEPRSGIDTNAYYVMTVGTKTFLVHREWFMDLNLPWSSAGSTVWRNRETLMEFEEPHATKQSVIALGSQEGALHQALAGAIPVEFSSNTVKLTSGHLKCRVKMEKLQLKGTTYGVCSKAFKFLGTPADTGHGTVVLELQYTGTDGPCKVPISSVASLNDLTPVGRLVTVNPFVSVATANAKVLIELEPPFGDSYIVVGRGEQQINHHWHKSGSSIGKAFTTTLKGAQRLAALGDTAWDFGSVGGVFTSVGKAVHQVFGGAFRSLFGGMSWITQGLLGALLLWMGINARDRSIALTFLAVGGVLLFLSVNVHADTGCAIDISRQELRCGSGVFIHNDVEAWMDRYKYYPETPQGLAKIIQKAHKEGVCGLRSVSRLEHQMWEAVKDELNTLLKENGVDLSVVVEKQEGMYKSAPKRLTATTEKFEIGWKAWGKSILFAPELANNTFVVDGPETKECPTQNRAWNSLEVEDFGFGLTSTRMFLKVRESNTTECDSKIIGTAVKNNLAIHSDLSYWIESRLNDTWKLERAVLGEVKSCTWPETHTLWGDGILESDLIIPVTLAGPRSNHNRRPGYKTQNQGPWDEGRVEIDFDYCPGTTVTLSESCGHRGPATRTTTESGKLITDWCCRSCTLPPLRYQTDSGCWYGMEIRPQRHDEKTLVQSQVNAYNADMIDPFQLGLLVVFLATQEVLRKRWTAKISMPAILIALLVLVFGGITYTDVLRYVILVGAAFAESNSGGDVVHLALMATFKIQPVFMVASFLKARWTNQENILLMLAAVFFQMAYHDARQILLWEIPDVLNSLAVAWMILRAITFTTTSNVVVPLLALLTPGLRCLNLDVYRILLLMVGIGSLIREKRSAAAKKKGASLLCLALASTGLFNPMILAAGLIACDPNRKRGWPATEVMTAVGLMFAIVGGLAELDIDSMAIPMTIAGLMFAAFVISGKSTDMWIERTADISWESDAEITGSSERVDVRLDDDGNFQLMNDPGAPWKIWMLRMVCLAISAYTPWAILPSVVGFWITLQYTKRGGVLWDTPSPKEYKKGDTTTGVYRIMTRGLLGSYQAGAGVMVEGVFHTLWHTTKGAALMSGEGRLDPYWGSVKEDRLCYGGPWKLQHKWNGQDEVQMIVVEPGKNVKNVQTKPGVFKTPEGEIGAVTLDFPTGTSGSPIVDKNGDVIGLYGNGVIMPNGSYISAIVQGERMDEPIPAGFEPEMLRKKQITVLDLHPGAGKTRRILPQIIKEAINRRLRTAVLAPTRVVAAEMAEALRGLPIRYQTSAVPREHNGNEIVDVMCHATLTHRLMSPHRVPNYNLFVMDEAHFTDPASIAARGYISTKVELGEAAAIFMTATPPGTSDPFPESNSPISDLQTEIPDRAWNSGYEWITEYTGKTVWFVPSVKMGNEIALCLQRAGKKVVQLNRKSYETEYPKCKNDDWDFVITTDISEMGANFKASRVIDSRKSVKPTIITEGEGRVILGEPSAVTAASAAQRRGRIGRNPSQVGDEYCYGGHTNEDDSNFAHWTEARIMLDNINMPNGLIAQFYQPEREKVYTMDGEYRLRGEERKNFLELLRTADLPVWLAYKVAAAGVSYHDRRWCFDGPRTNTILEDNNEVEVITKLGERKILRPRWIDARVYSDHQALKAFKDFASGKRSQIGLIEVLGKMPEHFMGKTWEALDTMYVVATAEKGGRAHRMALEELPDALQTIALIALLSVMTMGVFFLLMQRKGIGKIGLGGAVLGVATFFCWMAEVPGTKIAGMLLLSLLLMIVLIPEPEKQRSQTDNQLAVFLICVMTLVSAVAANEMGWLDKTKSDISSLFGQRIEVKENFSMGEFLLDLRPATAWSLYAVTTAVLTPLLKHLITSDYINTSLTSINVQASALFTLARGFPFVDVGVSALLLAAGCWGQVTLTVTVTAATLLFCHYAYMVPGWQAEAMRSAQRRTAAGIMKNAVVDGIVATDVPELERTTPIMQKKVGQIMLILVSLAAVVVNPSVKTVREAGILITAAAVTLWENGASSVWNATTAIGLCHIMRGGWLSCLSITWTLIKNMEKPGLKRGGAKGRTLGEVWKERLNQMTKEEFTRYRKEAIIEVDRSAAKHARKEGNVTGGHPVSRGTAKLRWLVERRFLEPVGKVIDLGCGRGGWCYYMATQKRVQEVRGYTKGGPGHEEPQLVQSYGWNIVTMKSGVDVFYRPSECCDTLLCDIGESSSSAEVEEHRTIRVLEMVEDWLHRGPREFCVKVLCPYMPKVIEKMELLQRRYGGGLVRNPLSRNSTHEMYWVSRASGNVVHSVNMTSQVLLGRMEKRTWKGPQYEEDVNLGSGTRAVGKPLLNSDTSKIKNRIERLRREYSSTWHHDENHPYRTWNYHGSYDVKPTGSASSLVNGVVRLLSKPWDTITNVTTMAMTDTTPFGQQRVFKEKVDTKAPEPPEGVKYVLNETTNWLWAFLAREKRPRMCSREEFIRKVNSNAALGAMFEEQNQWRSAREAVEDPKFWEMVDEEREAHLRGECHTCIYNMMGKREKKPGEFGKAKGSRAIWFMWLGARFLEFEALGFLNEDHWLGRKNSGGGVEGLGLQKLGYILREVGTRPGGKIYADDTAGWDTRITRADLENEAKVLELLDGEHRRLARAIIELTYRHKVVKVMRPAADGRTVMDVISREDQRGSGQVVTYALNTFTNLAVQLVRMMEGEGVIGPDDVEKLTKGKGPKVRTWLFENGEERLSRMAVSGDDCVVKPLDDRFATSLHFLNAMSKVRKDIQEWKPSTGWYDWQQVPFCSNHFTELIMKDGRTLVVPCRGQDELVGRARISPGAGWNVRDTACLAKSYAQMWLLLYFHRRDLRLMANAICSAVPVNWVPTGRTTWSIHAGGEWMTTEDMLEVWNRVWIEENEWMEDKTPVEKWSDVPYSGKREDIWCGSLIGTRARATWAENIQVAINQVRAIIGDEKYVDYMSSLKRYEDTTLVEDTVL

>ANW68674.1 genome polyprotein [West Nile virus]

MSKKPGGPGKSRAVNMLKRGMPRVLSLIGLKRAMLSLIDGKGPIRFVLALLAFFRFTAIAPTRAVLDRWRGVNKQTAMKHLLSFKKELGTLTSAINRRSSKQKKRGGKTGIAVMIGLIASVGAVTLSNFQGKVMMTVNATDVTDVITIPTAAGKNLCIVRAMDVGYMCDDTITYECPVLSAGNDPEDIDCWCTKSAVYVRYGRCTKTRHSRRSRRSLTVQTHGESTLANKKGAWMDSTKATRYLVKTESWILRNPGYALVAAVIGWMLGSNTMQRVVFVVLLLLVAPAYSFNCLGMSNRDFLEGVSGATWVDLVLEGDSCVTIMSKDKPTIDVKMMNMEAANLAEVRSYCYLATVSDLSTKAACPTMGEAHNDKRADPAFVCRQGVVDRGWGNGCGLFGKGSIDTCAKFACSTKAIGRTILKENIKYEVAIFVHGPTTVESHGNYSTQAGATQAGRFSITPAAPSYTLKLGEYGEVTVDCEPRSGIDTNAYYVMTVGTKTFLVHREWFMDLNLPWSSAGSTVWRNRETLMEFEEPHATKQSVIALGSQEGALHQALAGAIPVEFSSNTVKLTSGHLKCRVKMEKLQLKGTTYGVCSKAFKFLGTPADTGHGTVVLELQYTGTDGPCKVPISSVASLNDLTPVGRLVTVNPFVSVATANAKVLIELEPPFGDSYIVVGRGEQQINHHWHKSGSSIGKAFTTTLKGAQRLAALGDTAWDFGSVGGVFTSVGKAVHQVFGGAFRSLFGGMSWITQGLLGALLLWMGINARDRSIALTFLAVGGVLLFLSVNVHADTGCAIDISRQELRCGSGVFIHNDVEAWMDRYKYYPETPQGLAKIIQKAHKEGVCGLRSVSRLEHQMWEAVKDELNTLLKENGVDLSVVVEKQEGMYKSAPKRLTATTEKLEIGWKAWGKSILFAPELANNTFVVDGPETKECPTQNRAWNSLEVEDFGFGLTSTRMFLKVRESNTTECDSKIIGTAVKNNLAIHSDLSYWIESRLNDTWKLERAVLGEVKSCTWPETHTLWGDGILESDLIIPVTLAGPRSNHNRRPGYKTQNQGPWDEGRVEIDFDYCPGTTVTLSESCGHRGPATRTTTESGKLITDWCCRSCTLPPLRYQTDSGCWYGMEIRPQRHDEKTLVQSQVNAYNADMIDPFQLGLLVVFLATQEVLRKRWTAKISMPAILIALLVLVFGGITYTDVLRYVILVGAAFAESNSGGDVVHLALMATFKIQPVFMVASFLKARWTNQENILLMLAAVFFQMAYHDARQILLWEIPDVLNSLAVAWMILRAITFTTTSNVVVPLLALLTPGLRCLNLDVYRILLLMVGIGSLIREKRSAAAKKKGASLLCLALASTGFFNPMILAAGLIACDPNRKRGWPATEVMTAVGLMFAIVGGLAELDIDSMAIPMTIAGLMFAAFVISGKSTDMWIERTADISWESDAEITGSSERVDVRLDDDGNFQLMNDPGAPWKIWMLRMVCLAISAYTPWAILPSVVGFWITLQYTKRGGVLWDTPSPKEYKKGDTTTGVYRIMTRGLLGSYQAGAGVMVEGVFHTLWHTTKGAALMSGEGRLDPYWGSVKEDRLCYGGPWKLQHKWNGQDEVQMIVVEPGKNVKNVQTKPGVFKTPEGEIGAVTLDFPTGTSGSPIVDKNGDVIGLYGNGVIMPNGSYISAIVQGERMDEPIPAGFEPEMLRKKQITVLDLHPGAGKTRRILPQIIKEAINRRLRTAVLAPTRVVAAEMAEALRGLPIRYQTSAVPREHNGNEIVDVMCHATLTHRLMSPHRVPNYNLFVMDEAHFTDPASIAARGYISTKVELGEAAAIFMTATPPGTSDPFPESNSPISDLQTEIPDRAWNSGYEWITEYTGKTVWFVPSVKMGNEIALCLQRAGKKVVQLNRKSYETEYPKCKNDDWDFVITTDISEMGANFKASRVIDSRKSVKPTIITEGEGRVILGEPSAVTAASAAQRRGRIGRNPSQVGDEYCYGGHTNEDDSNFAHWTEARIMLDNINMPNGLIAQFYQPEREKVYTMDGEYRLRGEERKNFLELLRTADLPVWLAYKVAAAGVSYHDRRWCFDGPRTNTILEDNNEVEVITKLGERKILRPRWIDARVYSDHQALKAFKDFASGKRSQIGLIEVLGKMPEHFMGKTWEALDTMYVVATAEKGGRAHRMALEELPDALQTIALIALLSVMTMGVFFLLMQRKGIGKIGLGGAVLGVATFFCWMAEVPGTKIAGMLLLSLLLMIVLIPEPEKQRSQTDNQLAVFLICVMTLVSAVAANEMGWLDKTKSDISSLFGQRIEVKENFSMGEFLLDLRPATAWSLYAVTTAVLTPLLKHLITSDYINTSLTSINVQASALFTLARGFPFVDVGVSALLLAAGCWGQVTLTVTVTAATLLFCHYAYMVPGWQAEAMRSAQRRTAAGIMKNAVVDGIVATDVPELERTTPIMQKKVGQIMLILVSLAAVVVNPSVKTVREAGILITAAAVTLWENGASSVWNATTAIGLCHIMRGGWLSCLSITWTLIKNMEKPGLKRGGAKGRTLGEVWKERLNQMTKEEFTRYRKEAIIEVDRSAAKHARKEGNVTGGHPVSRGTAKLRWLVERRFLEPVGKVIDLGCGRGGWCYYMATQKRVQEVRGYTKGGPGHEEPQLVQSYGWNIVTMKSGVDVFYRPSECCDTLLCDIGESSSSAEVEEHRTIRVLEMVEDWLHRGPREFCVKVLCPYMPKVIEKMELLQRRYGGGLVRNPLSRNSTHEMYWVSRASGNVVHSVNMTSQVLLGRMEKRTWKGPQYEEDVNLGSGTRAVGKPLLNSDTSKIKNRIERLRREYSSTWHHDENHPYRTWNYHGSYDVKPTGSASSLVNGVVRLLSKPWDTITNVTTMAMTDTTPFGQQRVFKEKVDTKAPEPPEGVKYVLNETTNWLWAFLAREKRPRMCSREEFIRKVNSNAALGAMFEEQNQWRSAREAVEDPKFWEMVDEEREAHLRGECHTCIYNMMGKREKKPGEFGKAKGSRAIWFMWLGARFLEFEALGFLNEDHWLGRKNSGGGVEGLGLQKLGYILREVGTRPGGKIYADDTAGWDTRITRADLENEAKVLELLDGEHRRLARAIIELTYRHKVVKVMRPAADGRTVMDVISREDQRGSGQVVTYALNTFTNLAVQLVRMMEGEGVIGPDDVEKLTKGKGPKVRTWLFENGEERLSRMAVSGDDCVVKPLDDRFATSLHFLNAMSKVRKDIQEWKPSTGWYDWQQVPFCSNHFTELIMKDGRTLVVPCRGQDELVGRARISPGAGWNVRDTACLAKSYAQMWLLLYFHRRDLRLMANAICSAVPVNWVPTGRTTWSIHAGGEWMTTEDMLEVWNRVWIEENEWMEDKTPVEKWSDVPYSGKREDIWCGSLIGTRARATWAENIQVAINQVRAIIGDEKYVDYMSSLKRYEDTTLVEDTVL

>ANW69142.1 genome polyprotein [West Nile virus]

MSKKPGGPGKSRAVNMLKRGMPRVLSLIGLKRAMLSLIDGKGPIRFVLALLAFFRFTAIAPTRAVLDRWRGVNKQTAMKHLLSFKKELGTLTSAINRRSSKQKKRGGKTGIAVMIGLIASVGAVTLSNFQGKVMMTVNATDVTDVITIPTAAGKNLCIVRAMDVGYMCDDTITYECPVLSAGNDPEDIDCWCTKSAVYVRYGRCTKTRHSRRSRRSLTVQTHGESTLANKKGAWMDSTKATRYLVKTESWILRNPGYALVAAVIGWMLGSNTMQRVVFVVLLLLVAPAYSFNCLGMSNRDFLEGVSGATWVDLVLEGDSCVTIMSKDKPTIDVKMMNMEAANLAEVRSYCYLATVSDLSTKAACPTMGEAHNDKRADPAFVCRQGVVDRGWGNGCGLFGKGSIDTCAKFACSTKAIGRTILKENIKYEVAIFVHGPTTVESHGNYSTQAGATQAGRFSITPAAPSYTLKLGEYGEVTVDCEPRSGIDTNAYYVMTVGTKTFLVHREWFMDLNLPWSSAGSTVWRNRETLMEFEEPHATKQSVIALGSQEGALHQALAGAIPVEFSSNTVKLTSGHLKCRVKMEKLQLKGTTYGVCSKAFKFLGTPADTGHGTVVLELQYTGTDGPCKVPISSVASLNDLTPVGRLVTVNPFVSVATANAKVLIELEPPFGDSYIVVGRGEQQINHHWHKSGSSIGKAFTTTLKGAQRLAALGDTAWDFGSVGGVFTSVGKAVHQVFGGAFRSLFGGMSWITQGLLGALLLWMGINARDRSIALTFLAVGGVLLFLSVNVHADTGCAIDISRQELRCGSGVFIHNDVEAWMDRYKYYPETPQSLAKIIQKAHKEGVCGLRSVSRLEHQMWEAVKDELNTLLKENGVDLSVVVEKQEGMYKSAPKRLTATTEKLEIGWKAWGKSILFAPELANNTFVVDGPETKECPTQNRAWNSLEVEDFGFGLTSTRMFLKVRESNTTECDSKIIGTAVKNNLAIHSDLSYWIESRLNDTWKLERAVLGEVKSCTWPETHTLWGDGILESDLIIPVTLAGPRSNHNRRPGYKTQNQGPWDEGRVEIDFDYCPGTTVTLSESCGHRGPATRTTTESGKLITDWCCRSCTLPPLRYQTDSGCWYGMEIRPQRHDEKTLVQSQVNAYNADMIDPFQLGLLVVFLATQEVLRKRWTAKISMPAILIALLVLVFGGITYTDVLRYVILVGAAFAESNSGGDVVHLALMATFKIQPVFMVASFLKARWTNQENILLMLAAVFFQMAYHDARQILLWEIPDVLNSLAVAWMILRAITFTTTSNVVVPLLALLTPGLRCLNLDVYRILLLMVGIGSLIREKRSAAAKKKGASLLCLALASTGLFNPMILAAGLIACDPNRKRGWPATEVMTAVGLMFAIVGGLAELDIDSMAIPMTIAGLMFAAFVISGKSTDMWIERTADISWESDAEITGSSERVDVRLDDDGNFQLMNDPGAPWKIWMLRMVCLAISAYTPWAILPSVVGFWITLQYTKRGGVLWDTPSPKEYKKGDTTTGVYRIMTRGLLGSYQAGAGVMVEGVFHTLWHTTKGAALMSGEGRLDPYWGSVKEDRLCYGGPWKLQHKWNGQDEVQMIVVEPGKNVKNVQTKPGVFKTPEGEIGAVTLDFPTGTSGSPIVDKNGDVIGLYGNGVIMPNGSYISAIVQGERMDEPIPAGFEPEMLRKKQITVLDLHPGAGKTRRILPQIIKEAINRRLRTAVLAPTRVVAAEMAEALRGLPIRYQTSAVPREHNGNEIVDVMCHATLTHRLMSPHRVPNYNLFVMDEAHFTDPASIAARGYISTKVELGEAAAIFMTATPPGTSDPFPESNSPISDLQTEIPDRAWNSGYEWITEYTGKTVWFVPSVKMGNEIALCLQRAGKKVVQLNRKSYETEYPKCKNDDWDFVITTDISEMGANFKASRVIDSRKSVKPTIITEGEGRVILGEPSAVTAASAAQRRGRIGRNPSQVGDEYCYGGHTNEDDSNFAHWTEARIMLDNINMPNGLIAQFYQPEREKVYTMDGEYRLRGEERKNFLELLRTADLPVWLAYKVAAAGVSYHDRRWCFDGPRTNTILEDNNEVEVITKLGERKILRPRWIDARVYSDHQALKAFKDFASGKRSQIGLIEVLGKMPEHFMGKTWEALDTMYVVATAEKGGRAHRMALEELPDALQTIALIALLSVMTMGVFFLLMQRKGIGKIGLGGAVLGVATFFCWMAEVPGTKIAGMLLLSLLLMIVLIPEPEKQRSQTDNQLAVFLICVMTLVSAVAANEMGWLDKTKSDISSLFGQRIEVKENFSMGEFLLDLRPATAWSLYAVTTAVLTPLLKHLITSDYINTSLTSINVQASALFTLARGFPFVDVGVSALLLAAGCWGQVTLTVTVTAATLLFCHYAYMVPGWQAEAMRSAQRRTAAGIMKNAVVDGIVATDVPELERTTPIMQKKVGQIMLILVSLAAVVVNPSVKTVREAGILITAAAVTLWENGASSVWNATTAIGLCHIMRGGWLSCLSITWTLIKNMEKPGLKRGGAKGRTLGEVWKERLNQMTKEEFTRYRKEAIIEVDRSAAKHARKEGNVTGGHPVSRGTAKLRWLVERRFLEPVGKVIDLGCGRGGWCYYMATQKRVQEVRGYTKGGPGHEEPQLVQSYGWNIVTMKSGVDVFYRPSECCDTLLCDIGESSSSAEVEEHRTIRVLEMVEDWLHRGPREFCVKVLCPYMPKVIEKMELLQRRYGGGLVRNPLSRNSTHEMYWVSRASGNVVHSVNMTSQVLLGRMEKRTWKGPQYEEDVNLGSGTRAVGKPLLNSDTSKIKNRIERLRREYSSTWHHDENHPYRTWNYHGSYDVKPTGSASSLVNGVVRLLSKPWDTITNVTTMAMTDTTPFGQQRVFKEKVDTKAPEPPEGVKYVLNETTNWLWAFLAREKRPRMCSREEFIRKVNSNAALGAMFEEQNQWRSAREAVEDPKFWEMVDEEREAHLRGECHTCIYNMMGKREKKPGEFGKAKGSRAIWFMWLGARFLEFEALGFLNEDHWLGRKNSGGGVEGLGLQKLGYILREVGTRPGGKIYADDTAGWDTRITRADLENEAKVLELLDGEHRRLARAIIELTYRHKVVKVMRPAADGRTVMDVISREDQRGSGQVVTYALNTFTNLAVQLVRMMEGEGVIGPDDVEKLTKGKGPKVRTWLFENGEERLSRMAVSGDDCVVKPLDDRFATSLHFLNAMSKVRKDIQEWKPSTGWYDWQQVPFCSNHFTELIMKDGRTLVVPCRGQDELVGRARISPGAGWNVRDTACLAKSYAQMWLLLYFHRRDLRLMANAICSAVPVNWVPTGRTTWSIHAGGEWMTTEDMLEVWNRVWIEENEWMEDKTPVEKWSDVPYSGKREDIWCGSLIGTRARATWAENIQVAINQVRAIIGDEKYVDYMSSLKRYEDTTLVEDTVL

>AHW48696.1 polyprotein [West Nile virus]

MSKKPGGPGKSRAVNMLKRGMPRVLSLIGLKRAMLSLIDGKGPIRFVLALLAFFRFTAIAPTRAVLDRWRGVNKQTAMKHLLSFKKELGTLTSAINRRSSKQKKRGGKTGIAVMIGLIASVGAVTLSNFQGKVMMTVNATDVTDVITIPTAAGKNLCIVRAMDVGYMCDDTITYECPVLSAGNDPEDIDCWCTKSAVYVRYGRCTKTRHSRRSRRSLTVQTHGESTLANKKGAWMDSTKATRYLVKTESWILRNPGYALVAAVIGWMLGSNTMQRVVFVVLLLLVAPAYSFNCLGMSNRDFLEGVSGATWVDLVLEGDSCVTIMSKDKPTIDVKMMNMEAANLAEVRSYCYLATVSDLSTKAACPTMGEAHNDKRADPAFVCRQGVVDRGWGNGCGLFGKGSIDTCAKFACSTKAIGRTILKENIKYEVAIFVHGPTTVESHGNYSTQAGATQAGRFSITPAAPSYTLKLGEYGEVTVDCEPRSGIDTNAYYVMTVGTKTFLVHREWFMDLNLPWSSAGSTVWRNRETLMEFEEPHATKQSVIALGSQEGALHQALAGAIPVEFSSNTVKLTSGHLKCRVKMEKLQLKGTTYGVCSKAFKFLGTPADTGHGTVVLELQYTGTDGPCKVPISSVASLNDLTPVGRLVTVNPFVSVATANAKVLIELEPPFGDSYIVVGRGEQQINHHWHKSGSSIGKAFTTTLKGAQRLAALGDTAWDFGSVGGVFTSVGKAVHQVFGGAFRSLFGGMSWITQGLLGALLLWMGINARDRSIALTFLAVGGVLLFLSVNVHADTGCAIDISRQELRCGSGVFIHNDVEAWMDRYKYYPETPQGLAKIIQKAHKEGVCGLRSVSRLEHQMWEAVKDELNTLLKENGVDLSVVVEKQEGMYKSAPKRLTATTEKLEIGWKAWGKSILFAPELANNTFVVDGPETKECPTQNRAWNSLEVEDFGFGLTSTRMFLKVRESNTTECDSKIIGTAVKNNLAIHSDLSYWIESRLNDTWKLERAVLGEVKSCTWPETHTLWGDGILESDLIIPVTLAGPRSNHNRRPGYKTQNQGPWDEGRVEIDFDYCPGTTVTLSESCGHRGPATRTTTESGKLITDWCCRSCTLPPLRYQTDSGCWYGMEIRPQRHDEKTLVQSQVNAYNADMIDPFQLGLLVVFLATQEVLRKRWTAKISMPAILIALLVLVFGGITYTDVLRYVILVGAAFAESNSGGDVVHLALMATFKIQPVFMVASFLKARWTNQENILLMLAAVFFQMAYHDARQILLWEIPDVLNSLAVAWMILRAITFTTTSNVVVPLLALLTPGLRCLNLDVYRILLLMVGIGSLIKEKRSAAAKKKGASLLCLALASTGLFNPMILAAGLIACDPNRKRGWPATEVMTAVGLMFAIVGGLAELDIDSMAIPMTIAGLMFAAFVISGKSTDMWIERTADISWESDAEITGSSERVDVRLDDDGNFQLMNDPGAPWKIWMLRMVCLAISAYTPWAILPSVVGFWITLQYTKRGGVLWDTPSPKEYKKGDTTTGVYRIMTRGLLGSYQAGAGVMVEGVFHTLWHTTKGAALMSGEGRLDPYWGSVKEDRLCYGGPWKLQHKWNGQDEVQMIVVEPGKNVKNVQTKPGVFKTPEGEIGAVTLDFPTGTSGSPIVDKNGDVIGLYGNGVIMPNGSYISAIVQGERMDEPIPAGFEPEMLRKKQITVLDLHPGAGKTRRILPQIIKEAINRRLRTAVLAPTRVVAAEMAEALRGLPIRYQTSAVPREHNGNEIVDVMCHATLTHRLMSPHRVPNYNLFVMDEAHFTDPASIAARGYISTKVELGEAAAIFMTATPPGTSDPFPESNSPISDLQTEIPDRAWNSGYEWITEYTGKTVWFVPSVKMGNEIALCLQRAGKKVVQLNRKSYETEYPKCKNDDWDFVITTDISEMGANFKASRVIDSRKSVKPTIITEGEGRVILGEPSAVTAASAAQRRGRIGRNPSQVGDEYCYGGHTNEDDSNFAHWTEARIMLDNINMPNGLIAQFYQPEREKVYTMDGEYRLRGEERKNFLELLRTADLPVWLAYKVAAAGVSYHDRRWCFDGPRTNTILEDNNEVEVITKLGERKILRPRWIDARVYSDHQALKAFKDFASGKRSQIGLIEVLGKMPEHFMGKTWEALDTMYVVATAEKGGRAHRMALEELPDALQTIALIALLSVMTMGVFFLLMQRKGIGKIGLGGAVLGVATFFCWMAEVPGTKIAGMLLLSLLLMIVLIPEPEKQRSQTDNQLAVFLICVMTLVSAVAANEMGWLDKTKSDISSLFGQRIEVKENFSMGEFLLDLRPATAWSLYAVTTAVLTPLLKHLITSDYINTSLTSINVQASALFTLARGFPFVDVGVSALLLAAGCWGQVTLTVTVTAATLLFCHYAYMVPGWQAEAMRSAQRRTAAGIMKNAVVDGIVATDVPELERTTPIMQKKVGQIMLILVSLAAVVVNPSVKTVREAGILITAAAVTLWENGASSVWNATTAIGLCHIMRGGWLSCLSMTWTLIKNMEKPGLKRGGAKGRTLGEVWKERLNQMTKEEFTRYRKEAIIEVDRSAAKHARKEGNVTGGHPVSRGTAKLRWLVERRFLEPVGKVIDLGCGRGGWCYYMATQKRVQEVRGYTKGGPGHEEPQLVQSYGWNIVTMKSGVDVFYRPSECCDTLLCDIGESSSSAEVEEHRTIRVLEMVEDWLHRGPREFCVKVLCPYMPKVIEKMELLQRRYGGGLVRNPLSRNSTHEMYWVSRASGNVVHSVNMTSQVLLGRMEKRTWKGPQYEEDVNLGSGTRAVGKPLLNSDTSKIKNRIERLRREYSSTWHHDENHPYRTWNYHGSYDVKPTGSASSLVNGVVRLLSKPWDTITNVTTMAMTDTTPFGQQRVFKEKVDTKAPEPPEGVKYVLNETTNWLWAFLAREKRPRMCSREEFIRKVNSNAALGAMFEEQNQWRSAREAVEDPKFWEMVDEEREAHLRGECHTCIYNMMGKREKKPGEFGKAKGSRAIWFMWLGARFLEFEALGFLNEDHWLGRKNSGGGVEGLGLQKLGYILREVGTRPGGKIYADDTAGWDTRITRADLENEAKVLELLDGEHRRLARAIIELTYRHKVVKVMRPAADGRTVMDVISREDQRGSGQVVTYALNTFTNLAVQLVRMMEGEGVIGPDDVEKLTKGKGPKVRTWLFENGEERLSRMAVSGDDCVVKPLDDRFATSLHFLNAMSKVRKDIQEWKPSTGWYDWQQVPFCSNHFTELIMKDGRTLVVPCRGQDELVGRARISPGAGWNVRDTACLAKSYAQMWLLLYFHRRDLRLMANAICSAVPVNWVPTGRTTWSIHAGGEWMTTEDMLEVWNRVWIEENEWMEDKTPVEKWSDVPYSGKREDIWCGSLIGTRARATWAENIQVAINQVRAIIGDEKYVDYMSSLKRYEDTTLVEDTVL

>AYV87265.1 polyprotein [West Nile virus]

MSKKPGGPGKSRAVNMLKRGMPRVLSLIGLKRAMLSLIDGKGPIRFVLALLAFFRFTAIAPTRAVLDRWRGVNKQTAMKHLLSFKKELGTLTSAINRRSSKQKKRGGKTGIAVMIGLIASVGAVTLSNFQGKVMMTVNATDVTDVITIPTAAGKNLCIVRAMDVGYMCDDTITYECPVLSAGNDPEDIDCWCTKSAVYVRYGRCTKTRHSRRSRRSLTVQTHGESTLANKKGAWMDSTKATRYLVKTESWILRNPGYALVAAVIGWMLGSNTMQRVVFVVLLLLVAPAYSFNCLGMSNRDFLEGVSGATWVDLVLEGDSCVTIMSKDKPTIDVKMMNMEAANLAEVRSYCYLATVSDLSTKAACPTMGEAHNDKRADPAFVCRQGVVDRGWGNGCGLFGKGSIDTCAKFACSTKAIGRTILKENIKYEVAIFVHGPTTVESHGNYSTQVGATQAGRFSITPAAPSYTLKLGEYGEVTVDCEPRSGIDTNAYYVMTVGTKTFLVHREWFMDLNLPWSSAGSTVWRNRETLMEFEEPHATKQSVIALGSQEGALHQALAGAIPVEFSSNTVKLTSGHLKCRVKMEKLQLKGTTYGVCSKAFKFLGTPADTGHGTVVLELQYTGTDGPCKVPISSVASLNDLTPVGRLVTVNPFVSVATANAKVLIELEPPFGDSYIVVGRGEQQINHHWHKSGSSIGKAFTTTLKGAQRLAALGDTAWDFGSVGGVFTSVGKAVHQVFGGAFRSLFGGMSWITQGLLGALLLWMGINARDRSIALTFLAVGGVLLFLSVNVHADTGCAIDISRQELRCGSGVFIHNDVEAWMDRYKYYPETPQGLAKIIQKAHKEGVCGLRSVSRLEHQMWEAVKDELNTLLKENGVDLSVVVEKQEGMYKSAPKRLTATTEKLEIGWKAWGKSILFAPELANNTFVVDGPETKECPTQNRAWNSLEVEDFGFGLTSTRMFLKVRESNTTECDSKIIGTAVKNNLAIHSDLSYWIESRLNDTWKLERAVLGEVKSCTWPETHTLWGDGILESDLIIPVTLAGPRSNHNRRPGYKTQNQGPWDEGRVEIDFDYCPGTTVTLSESCGHRGPATRTTTESGKLITDWCCRSCTLPPLRYQTDSGCWYGMEIRPQRHDEKTLVQSQVNAYNADMIDPFQLGLLVVFLATQEVLRKRWTAKISMPAILIALLVLVFGGITYTDVLRYVILVGAAFAESNSGGDVVHLALMATFKIQPVFMVASFLKARWTNQENILLMLAAVFFQMAYHDARQILLWEIPDVLNSLAVAWMILRAITFTTTSNVVVPLLALLTPGLRCLNLDVYRILLLMVGIGSLIREKRSAAAKKKGASLLCLALASTGLFNPMILAAGLIACDPNRKRGWPATEVMTAVGLMFAIVGGLAELDIDSMAIPMTIAGLMFAAFVISGKSTDMWIERTADISWESDAEITGSSERVDVRLDDDGNFQLMNDPGAPWKIWMLRMVCLAISAYTPWAILPSVVGFWITLQYTKRGGVLWDTPSPKEYKKGDTTTGVYRIMTRGLLGSYQAGAGVMVEGVFHTLWHTTKGAALMSGEGRLDPYWGSVKEDRLCYGGPWKLQHKWNGQDEVQMIVVEPGKNVKNVQTKPGVFKTPEGEIGAVTLDFPTGTSGSPIVDKNGDVIGLYGNGVIMPNGSYISAIVQGERMDEPIPAGFEPEMLRKKQITVLDLHPGAGKTRRILPQIIKEAINRRLRTAVLAPTRVVAAEMAEALRGLPIRYQTSAVPREHNGNEIVDVMCHATLTHRLMSPHRVPNYNLFVMDEAHFTDPASIAARGYISTKVELGEAAAIFMTATPPGTSDPFPESNSPISDLQTEIPDRAWNSGYEWITEYTGKTVWFVPSVKMGNEIALCLQRAGKKVVQLNRKSYETEYPKCKNDDWDFVITTDISEMGANFKASRVIDSRKSVKPTIITEGEGRVILGEPSAVTAASAAQRRGRIGRNPSQVGDEYCYGGHTNEDDSNFAHWTEARIMLDNINMPNGLIAQFYQPEREKVYTMDGEYRLRGEERKNFLELLRTADLPVWLAYKVAAAGVSYHDRRWCFDGPRTNTILEDNNEVEVITKLGERKILRPRWIDARVYSDHQALKAFKDFASGKRSQIGLIEVLGKMPEHFMGKTWEALDTMYVVATAEKGGRAHRMALEELPDALQTIALIALLSVMTMGVFFLLMQRKGIGKIGLGGAVLGVATFFCWMAEVPGTKIAGMLLLSLLLMIVLIPEPEKQRSQTDNQLAVFLICVMTLVSAVAANEMGWLDKTKSDISSLFGQRIEVKENFSMGEFLLDLRPATAWSLYAVTTAVLTPLLKHLITSDYINTSLTSINVQASALFTLSRGFPFVDVGVSALLLAAGCWGQVTLTVTVTAATLLFCHYAYMVPGWQAEAMRSAQRRTAAGIMKNAVVDGIVATDVPELERTTPIMQKKVGQIMLILVSLAAVVVNPSVKTVREAGILITAAAVTLWENGASSVWNATTAIGLCHIMRGGWLSCLSITWTLIKNMEKPGLKRGGAKGRTLGEVWKERLNQMTKEEFTRYRKEAIIEVDRSAAKHARKEGNVTGGHPVSRGTAKLRWLVERRFLEPVGKVIDLGCGRGGWCYYMATQKRVQEVRGYTKGGPGHEEPQLVQSYGWNIVTMKSGVDVFYRPSECCDTLLCDIGESSSSAEVEEHRTIRVLEMVEDWLHRGPREFCVKVLCPYMPKVIEKMELLQRRYGGGLVRNPLSRNSTHEMYWVSRASGNVVHSVNMTSQVLLGRMEKRTWKGPQYEEDVNLGSGTRAVGKPLLNSDTSKIKNRIERLRREYSSTWHHDENHPYRTWNYHGSYDVKPTGSASSLVNGVVRLLSKPWDTITNVTTMAMTDTTPFGQQRVFKEKVDTKAPEPPEGVKYVLNETTNWLWAFLAREKRPRMCSREEFIRKVNSNAALGAMFEEQNQWRSAREAVEDPKFWEMVDEEREAHLRGECHTCIYNMMGKREKKPGEFGKAKGSRAIWFMWLGARFLEFEALGFLNEDHWLGRKNSGGGVEGLGLQKLGYILREVGTRPGGKIYADDTAGWDTRITRADLENEAKVLELLDGEHRRLARAIIELTYRHKVVKVMRPAADGRTVMDVISREDQRGSGQVVTYALNTFTNLAVQLVRMMEGEGVIGPDDVEKLTKGKGPKVRTWLVENGEERLSRMAVSGDDCVVKPLDDRFATSLHFLNAMSKVRKDIQEWKPSTGWYDWQQVPFCSNHFTELIMKDGRTLVVPCRGQDELVGRARISPGAGWNVRDTACLAKSYAQMWLLLYFHRRDLRLMANAICSAVPVNWVPTGRTTWSIHAGGEWMTTEDMLEVWNRVWIEENEWMEDKTPVEKWSDVPYSGKREDIWCGSLIGTRARATWAENIQVAINQVRAIIGDEKYVDYMSSLKRYEDTTLVEDTVL

>ACV90481.1 polyprotein [West Nile virus]

MSKKPGGPGKSRAVNMLKRGMPRVLSLIGLKRAMLSLIDGKGPIRFVLALLAFFRFTAIAPTRAVLDRWRGVNKQTAMKHLLSFKKELGTLTSAINRRSSKQKKRGGKTGIAVMIGLIASVGAVTLSNFQGKVMMTVNATDVTDVITIPTAAGKNLCIVRAMDVGYMCDDTITYECPVLSAGNDPEDIDCWCTKSAVYVRYGRCTKTRHSRRSRRSLTVQTHGESTLANKKGAWMDSTKATRYLVKTESWILRNPGYALVAAVIGWMLGSNTMQRVVFVVLLLLVAPAYSFNCLGMSNRDFLEGVSGATWVDLVLEGDSCVTIMSKDKPTIDVKMMNMEAANLAEVRSYCYLATVSDLSTKAACPTMGEAHNDKRADPAFVCRQGVVDRGWGNGCGLFGKGSIDTCAKFACSTKAIGRTILKENIKYEVAIFVHGPTTVESHGNYSTQAGATQAGRFSITPAAPSYTLKLGEYGEVTVDCEPRSGIDTNAYYVMTVGTKTFLVHREWFMDLNLPWSSAGSTVWRNRETLMEFEEPHATKQSVIALGSQEGALHQALAGAIPVEFSSNTVKLTSGHLKCRVKMEKLQLKGTTYGVCSKAFKFLGTPADTGHGTVVLELQYTGTDGPCKVPISSVASLNDLTPVGRLVTVNPFVSVATANAKVLIELEPPFGDSYIVVGRGEQQINHHWHKSGSSIGKAFTTTLKGAQRLAALGDTAWDFGSIGGVFTSVGKAVHQVFGGAFRSLFGGMSWITQGLLGALLLWMGINARDRSIALTFLAVGGVLLFLSVNVHADTGCAIDISRQELRCGSGVFIHNDVEAWMDRYKYYPETPQGLAKIIQKAHKEGVCGLRSVSRLEHQMWEAVKDELNTLLKENGVDLSVVVEKQEGMYKSAPKRLTATTEKLEIGWKAWGKSILFAPELANNTFVVDGPETKECPTQNRAWNSLEVEDFGFGLTSTRMFLKVRESNTTECDSKIIGTAVKNNLAIHSDLSYWIESRLNDTWKLERAVLGEVKSCTWPETHTLWGDGILESDLIIPVTLAGPRSNHNRRPGYKTQNQGPWDEGRVEIDFDYCPGTTVTLSESCGHRGPATRTTTESGKLITDWCCRSCTLPPLRYQTDSGCWYGMEIRPQRHDEKTLVQSQVNAYNADMIDPFQLGLLVVFLATQEVLRKRWTAKISMPAILIALLVLVFGGITYTDVLRYVILVGAAFAESNSGGDVVHLALMATFKIQPVFMVASFLKARWTNQENILLMLAAVFFQMAYHDARQILLWEIPDVLNSLAVAWMILRAITFTTTSNVVVPLLALLTPGLRCLNLDVYRILLLMVGIGSLIREKRSAAAKKKGASLLCLALASTGLFNPMILAAGLIACDPNRKRGWPATEVMTAVGLMFAIVGGLAELDIDSMAIPMTIAGLMFAAFVISGKSTDMWIERTADISWESDAEITGSSERVDVRLDDDGNFQLVNDPGAPWKIWMLRMVCLAISAYTPWAILPSVVGFWITLQYTKRGGVLWDTPSPKEYKKGDTTTGVYRIMTRGLLGSYQAGAGVMVEGVFHTLWHTTKGAALMSGEGRLDPYWGSVKEDRLCYGGPWKLQHKWNGQDEVQMIVVEPGKNVKNVQTKPGVFKTPEGEIGAVTLDFPTGTSGSPIVDKNGDVIGLYGNGVIMPNGSYISAIVQGERMDEPIPAGFEPEMLRKKQITVLDLHPGAGKTRRILPQIIKEAINRRLRTAVLAPTRVVAAEMAEALRGLPIRYQTSAVPREHNGNEIVDVMCHATLTHRLMSPHRVPNYNLFVMDEAHFTDPASIAARGYISTKVELGEAAAIFMTATPPGTSDPFPESNSPISDLQTEIPDRAWNSGYEWITEYTGKTVWFVPSVKMGNEIALCLQRAGKKVVQLNRKSYETEYPKCKNDDWDFVITTDISEMGANFKASRVIDSRKSVKPTIITEGEGRVILGEPSAVTAASAAQRRGRIGRNPSQVGDEYCYGGHTNEDDSNFAHWTEARIMLDNINMPNGLIAQFYQPEREKVYTMDGEYRLRGEERKNFLELLRTADLPVWLAYKVAAAGVSYHDRRWCFDGPRTNTILEDNNEVEVITKLGERKILRPRWIDARVYSDHQALKAFKDFASGKRSQIGLIEVLGKMPEHFMGKTWEALDTMYVVATAEKGGRAHRMALEELPDALQTIALIALLSVMTMGVFFLLMQRKGIGKIGLGGAVLGVATFFCWMAEVPGTKIAGMLLLSLLLMIVLIPEPEKQRSQTDNQLAVFLICVMTLVSAVAANEMGWLDKTKSDISSLFGQRIEVKENFSMGEFLLDLRPATAWSLYAVTTAVLTPLLKHLITSDYINTSLTSINVQASALFTLARGFPFVDVGVSALLLAAGCWGQVTLTVTVTAATLLFCHYAYMVPGWQAEAMRSAQRRTAAGIMKNAVVDGIVATDVPELERTTPIMQKKVGQIMLILVSLAAVVVNPSVKTVREAGILITAAAVTLWENGASSVWNATTAIGLCHIMRGGWLSCLSITWTLIKNMEKPGLKRGGAKGRTLGEVWKERLNQMTKEEFTRYRKEAIIEVDRSAAKHARKEGNVTGGHPVSRGTAKLRWLVERRFLEPVGKVIDLGCGRGGWCYYMATQKRVQEVRGYTKGGPGHEEPQLVQSYGWNIVTMKSGVDVFYRPSECCDTLLCDIGESSSSAEVEEHRTIRVLEMVEDWLHRGPREFCVKVLCPYMPKVIEKMELLQRRYGGGLVRNPLSRNSTHEMYWVSRASGNVVHSVNMTSQVLLGRMEKRTWKGPQYEEDVNLGSGTRAVGKPLLNSDTSKIKNRIERLRREYSSTWHHDENHPYRTWNYHGSYDVKPTGSASSLVNGVVRLLSKPWDTITNVTTMAMTDTTPFGQQRVFKEKVDTKAPEPPEGVKYVLNETTNWLWAFLAREKRPRMCSREEFIRKVNSNAALGAMFEEQNQWRSAREAVEDPKFWEMVDEEREAHLRGECHTCIYNMMGKREKKPGEFGKAKGSRAIWFMWLGARFLEFEALGFLNEDHWLGRKNSGGGVEGLGLQKLGYILREVGTRPGGKIYADDTAGWDTRITRADLENEAKVLELLDGEHRRLARAIIELTYRHKVVKVMRPAADGRTVMDVISREDQRGSGQVVTYALNTFTNLAVQLVRMMEGEGVIGPDDVEKLTKGKGPKVRTWLFENGEERLSRMAVSGDDCVVKPLDDRFATSLHFLNAMSKVRKDIQEWKPSTGWYDWQQVPFCSNHFTELIMKDGRTLVVPCRGQDELVGRARISPGAGWNVRDTACLAKSYAQMWLLLYFHRRDLRLMANAICSAVPVNWVPTGRTTWSIHAGGEWMTTEDMLEVWNRVWIEENEWMEDKTPVEKWSDVPYSGKREDIWCGSLIGTRARATWAENIQVAINQVRAIIGDEKYVDYMSSLKRYEDTTLVEDTVL

>AHW48623.1 polyprotein [West Nile virus]

MSKKPGGPGKSRAVNMLKRGMPRVLSLIGLKRAMLSLIDGKGPIRFVLALLAFFRFTAIAPTRAVLDRWRGVNKQTAMKHLLSFKKELGTLTSAINRRSSKQKKRGGKTGVAVMIGLIASVGAVTLSNFQGKVMMTVNATDVTDVITIPTAAGKNLCIVRAMDVGYMCDDTITYECPVLSAGNDPEDIDCWCTKSAVYVRYGRCTKTRHSRRSRRSLTVQTHGESTLANKKGAWMDSTKATRYLVKTESWILRNPGYALVAAVIGWMLGSNTMQRVVFVVLLLLVAPAYSFNCLGMSNRDFLEGVSGATWVDLVLEGDSCVTIMSKDKPTIDVKMMNVEAANLAEVRSYCYLATVSDLSTKAACPTMGEAHNDKRADPAFVCRQGVVDRGWGNGCGLFGKGSIDTCAKFACSTKAIGRTILKENIKYEVAIFVHGPTTVESHGNYSTQAGATQAGRFSITPAAPSYTLKLGEYGEVTVDCEPRSGIDTNAYYVMTVGTKTFLVHREWFMDLNLPWSSAGSTVWRNRETLMEFEEPHATKQSVIALGSQEGALHQALAGAIPVEFSSNTVKLTSGHLKCRVKMEKLQLKGTTYGVCSKAFKFLGTPADTGHGTVVLELQYTGTDGPCKVPISSVASLNDLTPVGRLVTVNPFVSVATANAKVLIELEPPFGDSYIVVGRGEQQINHHWHKSGSSIGKAFTTTLKGAQRLAALGDTAWDFGSVGGVFTSVGKAVHQVFGGAFRSLFGGMSWITQGLLGALLLWMGINARDRSIALTFLAVGGVLLFLSVNVHADTGCAIDISRQELRCGSGVFIHNDVEAWMDRYKYYPETPQGLAKIIQKAHKEGVCGLRSVSRLEHQMWEAVKDELNTLLKENGVDLSVVVEKQEGMYKSAPKRLTATTEKLEIGWKAWGKSILFAPELANNTFVVDGPETKECPTQNRAWNSLEVEDFGFGLTSTRMFLKVRESNTTECDSKIIGTAVKNNLAIHSDLSYWIESRLNDTWKLERAVLGEVKSCTWPETHTLWGDGILESDLIIPVTLAGPRSNHNRRPGYKTQNQGPWDEGRVEIDFDYCPGTTVTLSESCGHRGPATRTTTESGKLITDWCCRSCTLPPLRYQTDSGCWYGMEIRPQRHDEKTLVQSQVNAYNADMIDPFQLGLLVVFLATQEVLRKRWTAKISMPAILIALLVLVFGGITYTDVLRYVILVGAAFAESNSGGDVVHLALMATFKIQPVFMVASFLKARWTNQENILLMLAAVFFQMAYHDARQILLWEIPDVLNSLAVAWMILRAITFTTTSNVVVPLLALLTPGLRCLNLDVYRILLLMVGIGSLIREKRSAAAKKKGASLLCLALASTGLFNPMILAAGLIACDPNRKRGWPATEVMTAVGLMFAIVGGLAELDIDSMAIPMTIAGLMFAAFVISGKSTDMWIERTADISWESDAEITGSSERVDVRLDDDGNFQLMNDPGAPWKIWMLRMVCLAISAYTPWAILPSVVGFWITLQYTKRGGVLWDTPSPKEYKKGDTTTGVYRIMTRGLLGSYQAGAGVMVEGVFHTLWHTTKGAALMSGEGRLDPYWGSVKEDRLCYGGPWKLQHKWNGQDEVQMIVVEPGKNVKNVQTKPGVFKTPEGEIGAVTLDFPTGTSGSPIVDKNGDVIGLYGNGVIMPNGSYISAIVQGERMDEPIPAGFEPEMLRKKQITVLDLHPGAGKTRRILPQIIKEAINRRLRTAVLAPTRVVAAEMAEALRGLPIRYQTSAVPREHNGNEIVDVMCHATLTHRLMSPHRVPNYNLFVMDEAHFTDPASIAARGYISTKVELGEAAAIFMTATPPGTSDPFPESNSPISDLQTEIPDRAWNSGYEWITEYTGKTVWFVPSVKMGNEIALCLQRAGKKVVQLNRKSYETEYPKCKNDDWDFVITTDISEMGANFKASRVIDSRKSVKPTIITEGEGRVILGEPSAVTAASAAQRRGRIGRNPSQVGDEYCYGGHTNEDDSNFAHWTEARIMLDNINMPNGLIAQFYQPEREKVYTMDGEYRLRGEERKNFLELLRTADLPVWLAYKVAAAGVSYHDRRWCFDGPRTNTILEDNNEVEVITKLGERKILRPRWIDARVYSDHQALKAFKDFASGKRSQIGLIEVLGKMPEHFMGKTWEALDTMYVVATAEKGGRAHRMALEELPDALQTIALIALLSVMTMGVFFLLMQRKGIGKIGLGGAVLGVATFFCWMAEVPGTKIAGMLLLSLLLMIVLIPEPEKQRSQTDNQLAVFLICVMTLVSAVAANEMGWLDKTKSDISSLFGQRIEVKENFSMGEFLLDLRPATAWSLYAVTTAVLTPLLKHLITSDYINTSLTSINVQASALFTLARGFPFVDVGVSALLLAAGCWGQVTLTVTVTAATLLFCHYAYMVPGWQAEAMRSAQRRTAAGIMKNAVVDGIVATDVPELERTTPIMQKKVGQIMLILVSLAAVVVNPSVKTVREAGILITAAAVTLWENGASSVWNATTAIGLCHIMRGGWLSCLSITWTLIKNMEKPGLKRGGAKGRTLGEVWKERLNQMTKEEFTRYRKEAIIEVDRSAAKHARKEGNVTGGHPVSRGTAKLRWLVERRFLEPVGKVIDLGCGRGGWCYYMATQKRVQEVRGYTKGGPGHEEPQLVQSYGWNIVTMKSGVDVFYRPSECCDTLLCDIGESSSSAEVEEHRTIRVLEMVEDWLHRGPREFCVKVLCPYMPKVIEKMELLQRRYGGGLVRNPLSRNSTHEMYWVSRASGNVVHSVNMTSQVLLGRMEKRTWKGPQYEEDVNLGSGTRAVGKPLLNSDTSKIKNRIERLRREYSSTWHHDENHPYRTWNYHGSYDVKPTGSASSLVNGVVRLLSKPWDTITNVTTMAMTDTTPFGQQRVFKEKVDTKAPEPPEGVKYVLNETTNWLWAFLAREKRPRMCSREEFIRKVNSNAALGAMFEEQNQWRSAREAVEDPKFWEMVDEEREAHLRGECHTCIYNMMGKREKKPGEFGKAKGSRAIWFMWLGARFLEFEALGFLNEDHWLGRKNSGGGVEGLGLQKLGYILREVGTRPGGKIYADDTAGWDTRITRADLENEAKVLELLDGEHRRLARAIIELTYRHKVVKVMRPAADGRTVMDVISREDQRGSGQVVTYALNTFTNLAVQLVRMMEGEGVIGPDDVEKLTKGKGPKVRTWLFENGEERLSRMAVSGDDCVVKPLDDRFATSLHFLNAMSKVRKDIQEWKPSTGWYDWQQVPFCSNHFTELIMKDGRTLVVPCRGQDELVGRARISPGAGWNVRDTACLAKSYAQMWLLLYFHRRDLRLMANAICSAVPVNWVPTGRTTWSIHAGGEWMTTEDMLEVWNRVWIEENEWMEDKTPVEKWSDVPYSGKREDIWCGSLIGTRARATWAENIQVAINQVRAIIGDEKYVDYMSSLKRYEDTTLVEDTVL

>ADU76254.1 polyprotein [West Nile virus]

MSKKPGGPGKSRAVNMLKRGMPRVLSLIGLKRAMLSLIDGKGPIRFVLALLAFFRFTAIAPTRAVLDRWRGVNKQTAMKHLLSFKKELGTLTSAINRRSSKQKKRGGKTGIAVMIGLIASVGAVTLSNFQGKVMMTVNATDVTDVITIPTAAGKNLCIVRAMDVGYMCDDTITYECPVLSAGNDPEDIDCWCTKSAVYVRYGRCTKTRHSRRSRRSLTVQTHGESTLANKKGAWMDSTKATRYLVKTESWILRNPGYALVAAVIGWMLGSNTMQRVVFVVLLLLVAPAYSFNCLGMSNRDFLEGVSGATWVDLVLEGDSCVTIMSKDKPTIDVKMMNMEAANLAEVRSYCYLATVSDLSTKAACPTMGEAHNDKRADPAFVCRQGVVDRGWGNGCGLFGKGSIDTCAKFACSTKAIGRTILKENIKYEVAIFVHGPTTVESHGNYSTQVGATQAGRFSITPAAPSYTLKLGEYGEVTVDCEPRSGIDTNAYYVMTVGTKTFLVHREWFMDLNLPWSSAGSTVWRNRETLMEFEEPHATKQSVIALGSQEGALHQALAGAIPVEFSSNTVKLTSGHLKCRVKMEKLQLKGTTYGVCSKAFKFLGTPADTGHGTVVLELQYTGTDGPCKVPISSVASLNDLTPVGRLVTVNPFVSVATANAKVLIELEPPFGDSYIVVGRGEQQINHHWHKSGSSIGKAFTTTLKGAQRLAALGDTAWDFGSVGGVFTSVGKAVHQVFGGAFRSLFGGMSWITQGLLGALLLWMGINARDRSIALTFLAVGGVLLFLSVNVHADTGCAIDISRQELRCGSGVFIHNDVEAWMDRYKYYPETPQGLAKIIQKAHKEGVCGLRSVSRLEHQMWEAVKDELNTLLKENGVDLSVVVEKQEGMYKSAPKRLTATTEKLEIGWKAWGKSILFAPELANNTFVVDGPETKECPTQNRAWNSLEVEDFGFGLTSTRMFLKVRESNTTECDSKIIGTAVKNNLAIHSDLSYWIESRLNDTWKLERAVLGEVKSCTWPETHTLWGDGILESDLIIPVTLAGPRSNHNRRPGYKTQNQGPWDEGRVEIDFDYCPGTTVTLSESCGHRGPATRTTTESGKLITDWCCRSCTLPPLRYQTDSGCWYGMEIRPQRHDEKTLVQSQVNAYNADMIDPFQLGLLVVFLATQEVLRKRWTAKISMPAILIALLVLVFGGITYTDVLRYVILVGAAFAESNSGGDVVHLALMATFKIQPVFMVASFLKARWTNQENILLMLAAVFFQMAYHDARQILLWEIPDVLNSLAVAWMILRAITFTTTSNVVVPLLALLTPGLRCLNLDVYRILLLMVGIGSLIREKRSAAAKKKGASLLCLALASTGLFNPMILAAGLIACDPNRKRGWPATEVMTAVGLMFAIVGGLAELDIDSMAIPMTIAGLMFAAFVISGKSTDMWIERTADISWESDAEITGSSERVDVRLDDDGNFQLMNDPGAPWKIWMLRMVCLAISAYTPWAILPSVVGFWITLQYTKRGGVLWDTPSPKEYKKGDTTTGVYRIMTRGLLGSYQAGAGVMVEGVFHTLWHTTKGAALMSGEGRLDPYWGSVKEDRLCYGGPWKLQHKWNGQDEVQMIVVEPGKNVKNVQTKPGVFKTPEGEIGAVTLDFPTGTSGSPIVDKNGDVIGLYGNGVIMPNGSYISAIVQGERMDEPIPAGFEPEMLRKKQITVLDFHPGAGKTRRILPQIIKEAINRRLRTAVLAPTRVVAAEMAEALRGLPIRYQTSAVPREHNGNEIVDVMCHATLTHRLMSPHRVPNYNLFVMDEAHFTDPASIAARGYISTKVELGEAAAIFMTATPPGTSDPFPESNSPISDLQTEIPDRAWNSGYEWITEYTGKTVWFVPSVKMGNEIALCLQRAGKKVVQLNRKSYETEYPKCKNNDWDFVITTDISEMGANFKASRVIDSRKSVKPTIITEGEGRVILGEPSAVTAASAAQRRGRIGRNPSQVGDEYCYGGHTNEDDSNFAHWTEARIMLDNINMPNGLIAQFYQPEREKVYTMDGEYRLRGEERKNFLELLRTADLPVWLAYKVAAAGVSYHDRRWCFDGPRTNTILEDNNEVEVITKLGERKILRPRWIDARVYSDHQALKAFKDFASGKRSQIGLIEVLGKMPEHFMGKTWEALDTMYVVATAEKGGRAHRMALEELPDALQTIALIALLSVMTMGVFFLLMQRKGIGKIGLGGAVLGVATFFCWMAEVPGTKIAGMLLLSLLLMIVLIPEPEKQRSQTDNQLAVFLICVMTLVSAVAANEMGWLDKTKSDISSLFGQRIEVKENFSMGEFLLDLRPATAWSLYAVTTAVLTPLLKHLITSDYINTSLTSINVQASALFTLARGFPFVDVGVSALLLAAGCWGQVTLTVTVTAATLLFCHYAYMVPGWQAEAMRSAQRRTAAGIMKNAVVDGIVATDVPELERTTPIMQKKVGQIMLILVSLAAVVVNPSVKTVREAGILITAAAVTLWENGASSVWNATTAIGLCHIMRGGWLSCLSITWTLIKNMEKPGLKRGGAKGRTLGEVWKERLNQMTKEEFTRYRKEAIIEVDRSAAKHARKEGNVTGGHPVSRGTAKLRWLVERRFLEPVGKVIDLGCGRGGWCYYMATQKRVQEVRGYTKGGPGHEEPQLVQSYGWNIVTMKSGVDVFYRPSECCDTLLCDIGESSSSAEVEEHRTIRVLEMVEDWLHRGPREFCVKVLCPYMPKVIEKMELLQRRYGGGLVRNPLSRNSTHEMYWVSRASGNVVHSVNMTSQVLLGRMEKRTWKGPQYEEDVNLGSGTRAVGKPLLNSDTSKIKNRIERLRREYSSTWHHDENHPYRTWNYHGSYDVKPTGSASSLVNGVVRLLSKPWDTITNVTTMAMTDTTPFGQQRVFKEKVDTKAPEPPEGVKYVLNETTNWLWAFLAREKRPRMCSREEFIRKVNSNAALGAMFEEQNQWRSAREAVEDPKFWEMVDEEREAHLRGECHTCIYNMMGKREKKPGEFGKAKGSRAIWFMWLGARFLEFEALGFLNEDHWLGRKNSGGGVEGLGLQKLGYILREVGTRPGGKIYADDTAGWDTRITRADLENEAKVLELLDGEHRRLARAIIELTYRHKVVKVMRPAADGRTVMDVISREDQRGSGQVVTYALNTFTNLAVQLVRMMEGEGVIGPDDVEKLTKGKGPKVRTWLFENGEERLSRMAVSGDDCVVKPLDDRFATSLHFLNAMSKVRKDIQEWKPSTGWYDWQQVPFCSNHFTELIMKDGRTLVVPCRGQDELVGRARISPGAGWNVRDTACLAKSYAQMWLLLYFHRRDLRLMANAICSAVPVNWVPTGRTTWSIHAGGEWMTTEDMLEVWNRVWIEENEWMEDKTPVEKWSDVPYSGKREDIWCGSLIGTRARATWAENIQVAINQVRAIIGDEKYVDYMSSLKRYEDTTLVEDTVL

>ADL27944.1 polyprotein [West Nile virus]

MSKKPGGPGKSRAVNMLKRGMPRVLSLIGLKRAMLSLIDGKGPIRFVLALLAFFRFTAIAPTRAVLDRWRGVNKQTAMKHLLSFKKELGTLTSAINRRSSKQKKRGGKTGIAVMIGLIASVGAVTLSNFQGKVMMTVNATDVTDVITIPTAAGKNLCIVRAMDVGYMCDDTITYECPVLSAGNDPEDIDCWCTKSAVYVRYGRCTKTRHSRRSRRSLTVQTHGESTLANKKGAWMDSTKATRYLVKTESWILRNPGYALVAAVIGWMLGSNTMQRVVFVVLLLLVAPAYSFNCLGMSNRDFLEGVSGATWVDLVLEGDSCVTIMSKDKPTIDVKMMNMEAANLAEVRSYCYLATVSDLSTKAACPTMGEAHNDKRADPAFVCRQGVVDRGWGNGCGLFGKGSIDTCAKFACSTKAIGRTILKENIKYEVAIFVHGPTTVESHGNYSTQVGATQAGRFSITPAAPSYTLKLGEYGEVTVDCEPRSGIDTNAYYVMTVGTKTFLVHREWFMDLNLPWSSAGSTVWRNRETLMEFEEPHATKQSVIALGSQEGALHQALAGAIPVEFSSNTVKLTSGHLKCRVKMEKLQLKGTTYGVCSKAFKFLGTPADTGHGTVVLELQYTGTDGPCKVPISSVASLNDLTPVGRLVTVNPFVSVATANAKVLIELEPPFGDSYIVVGRGEQQINHHWHKSGSSIGKAFTTTLKGAQRLAALGDTAWDFGSVGGVFTSVGKAVHQVFGGAFRSLFGGMSWITQGLLGALLLWMGINARDRSIALTFLAVGGVLLFLSVNVHADTGCAIDISRQELRCGSGVFIHNDVEAWMDRYKYYPETPQGLAKIIQKAHKEGVCGLRSVSRLEHQMWEAVKDELNTLLKENGVDLSVVVEKQEGMYKSAPKRLTATTEKLEIGWKAWGKSILFAPELANNTFVVDGPETKECPTQNRAWNSLEVEDFGFGLTSTRMFLKVRESNTTECDSKIIGTAVKNNLAIHSDLSYWIESRFNDTWKLERAVLGEVKSCTWPETHTLWGDGILESDLIIPVTLAGPRSNHNRRPGYKTQNQGPWDEGRVEIDFDYCPGTTVTLSESCGHRGPATRTTTESGKLITDWCCRSCTLPPLRYQTDSGCWYGMEIRPQRHDEKTLVQSQVNAYNADMIDPFQLGLLVVFLATQEVLRKRWTAKISMPAILIALLVLVFGGITYTDVLRYVILVGAAFAESNSGGDVVHLALMATFKIQPVFMVASFLKARWTNQENILLMLAAVFFQMAYHDARQILLWEIPDVLNSLAVAWMILRAITFTTTSNVVVPLLALLTPGLRCLNLDVYRILLLMVGIGSLIREKRSAAAKKKGASLLCLALASTGLFNPMILAAGLIACDPNRKRGWPATEVMTAVGLMFAIVGGLAELDIDSMAIPMTIAGLMFAAFVISGKSTDMWIERTADISWESDAEITGSSERVDVRLDDDGNFQLMNDPGAPWKIWMLRMVCLAISAYTPWAILPSIVGFWITLQYTKRGGVLWDTPSPKEYKKGDTTTGVYRIMTRGLLGSYQAGAGVMVEGVFHTLWHTTKGAALMSGEGRLDPYWGSVKEDRLCYGGPWKLQHKWNGQDEVQMIVVEPGKNVKNVQTKPGVFKTPEGEIGAVTLDFPTGTSGSPIVDKNGDVIGLYGNGVIMPNGSYISAIVQGERMDEPIPAGFEPEMLRKKQITVLDLHPGAGKTRRILPQIIKEAINRRLRTAVLAPTRVVAAEMAEALRGLPIRYQTSAVPREHNGNEIVDVMCHATLTHRLMSPHRVPNYNLFVMDEAHFTDPASIAARGYISTKVELGEAAAIFMTATPPGTSDPFPESNSPISDLQTEIPDRAWNSGYEWITEYTGKTVWFVPSVKMGNEIALCLQRAGKKVVQLNRKSYETEYPKCKNDDWDFVITTDISEMGANFKASRVIDSRKSVKPTIITEGEGRVILGEPSAVTAASAAQRRGRIGRNPSQVGDEYCYGGHTNEDDSNFAHWTEARIMLDNINMPNGLIAQFYQPEREKVYTMDGEYRLRGEERKNFLELLRTADLPVWLAYKVAAAGVSYHDRRWCFDGPRTNTILEDNNEVEVITKLGERKILRPRWIDARVYSDHQALKAFKDFASGKRSQIGLIEVLGKMPEHFMGKTWEALDTMYVVATAEKGGRAHRMALEELPDALQTIALIALLSVMTMGVFFLLMQRKGIGKIGLGGAVLGVATFFCWMAEVPGTKIAGMLLLSLLLMIVLIPEPEKQRSQTDNQLAVFLICVMTLVSAVAANEMGWLDKTKSDISSLFGQRIEVKENFSMGEFLLDLRPATAWSLYAVTTAVLTPLLKHLITSDYINTSLTSINVQASALFTLARGFPFVDVGVSAFLLAAGCWGQVTLTVTVTAATLLFCHYAYMVPGWQAEAMRSAQRRTAAGIMKNAVVDGIVATDVPELERTTPIMQKKVGQIMLILVSLAAVVVNPSVKTVREAGILITAAAVTLWENGASSVWNATTAIGLCHIMRGGWLSCLSITWTLIKNMEKPGLKRGGAKGRTLGEVWKERLNQMTKEEFTRYRKEAIIEVDRSAAKHARKEGNVTGGHPVSRGTAKLRWLVERRFLEPVGKVIDLGCGRGGWCYYMATQKRVQEVRGYTKGGPGHEEPQLVQSYGWNIVTMKSGVDVFYRPSECCDTLLCDIGESSSSAEVEEHRTIRVLEMVEDWLHRGPREFCVKVLCPYMPKVIEKMELLQRRYGGGLVRNPLSRNSTHEMYWVSRASGNVVHSVNMTSQVLLGRMEKRTWKGPQYEEDVNLGSGTRAVGKPLLNSDTSKIKNRIERLRREYSSTWHHDENHPYRTWNYHGSYDVKPTGSASSLVNGVVRLLSKPWDTITNVTTMAMTDTTPFGQQRVFKEKVDTKAPEPPEGVKYVLNETTNWLWAFLAREKRPRMCSREEFIRKVNSNAALGAMFEEQNQWRSAREAVEDPKFWEMVDEEREAHLRGECHTCIYNMMGKREKKPGEFGKAKGSRAIWFMWLGARFLEFEALGFLNEDHWLGRKNSGGGVEGLGLQKLGYILREVGTRPGGKIYADDTAGWDTRITRADLENEAKVLELLDGEHRRLARAIIELTYRHKVVKVMRPAADGRTVMDVISREDQRGSGQVVTYALNTFTNLAVQLVRMMEGEGVIGPDDVEKLTKGKGPKVRTWLFENGEERLSRMAVSGDDCVVKPLDDRFATSLHFLNAMSKVRKDIQEWKPSTGWYDWQQVPFCSNHFTELIMKDGRTLVVPCRGQDELVGRARISPGAGWNVRDTACLAKSYAQMWLLLYFHRRDLRLMANAICSAVPVNWVPTGRTTWSIHAGGEWMTTEDMLEVWNRVWIEENEWMEDKTPVEKWSDVPYSGKREDIWCGSLIGTRARATWAENIQVAINQVRAIIGDEKYVDYMSSLKRYEDTTLVEDTVL

>AHW48651.1 polyprotein [West Nile virus]

MSKKPGGPGKSRAVNMLKRGMPRVLSLIGLKRAMLSLIDGKGPIRFVLALLAFFRFTAIAPTRAVLDRWRGVNKQTAMKHLLSFKKELGTLTSAINRRSSKQKKRGGKTGIAVMIGLIASVGAVTLSNFQGKVMMTVNATDVTDVITIPTAAGKNLCIVRAMDVGYMCDDTITYECPVLSAGNDPEDIDCWCTKSAVYVRYGRCTKTRHSRRSRRSLTVQTHGESTLANKKGAWMDSTKATRYLVKTESWILRNPGYALVAAVIGWMLGSNTMQRVVFVVLLLLVAPAYSFNCLGMSNRDFLEGVSGATWVDLVLEGDSCVTIMSKDKPTIDVKMMNMEAANLAEVRSYCYLATVSDLSTKAACPTMGEAHNDKRADPAFVCRQGVVDRGWGNGCGLFGKGSIDTCAKFACSTKAIGRTILKENIKYEVAIFVHGPTTVESHGNYSTQAGATQAGRFSITPAAPSYTLKLGEYGEVTVDCEPRSGIDTNAYYVMTVGTKTFLVHREWFMDLNLPWSSAGSTVWRNRETLMEFEEPHATKQSVIALGSQEGALHQALAGAIPVEFSSNTVKLTSGHLKCRVKMEKLQLKGTTYGVCSKAFKFLGTPADTGHGTVVLELQYTGTDGPCKVPISSVASLNDLTPVGRLVTVNPFVSVATANAKVLIELEPPFGDSYIVVGRGEQQINHHWHKSGSSIGKAFTTTLKGAQRLAALGDTAWDFGSVGGVFTSVGKAVHQVFGGAFRSLFGGMSWITQGLLGALLLWMGINARDRSIALTFLAVGGVLLFLSVNVHADTGCAIDISRQELRCGSGVFIHNDVEAWMDRYKYHPETPQGLAKIIQKAHKEGVCGLRSVSRLEHQMWEAVKDELNTLLKENGVDLSVVVEKQEGMYKSAPKRLTATTEKLEIGWKAWGKSILFAPELANNTFVVDGPETKECPTQNRAWNSLEVEDFGFGLTSTRMFLKVRESNTTECDSKIIGTAVKNNLAIHSDLSYWIESRLNDTWKLERAVLGEVKSCTWPETHTLWGDGILESDLIIPVTLAGPRSNHNRRPGYKTQNQGPWDEGRVEIDFDYCPGTTVTLSESCGHRGPATRTTTESGKLITDWCCRSCTLPPLRYQTDSGCWYGMEIRPQRHDEKTLVQSQVNAYNADMIDPFQLGLLVVFLATQEVLRKRWTAKISMPAILIALLVLVFGGITYTDVLRYVILVGAAFAESNSGGDVVHLALMATFKIQPVFMVASFLKARWTNQENILLMLAAVFFQMAYHDARQILLWEIPDVLNSLAVAWMILRAITFTTTSNVVVPLLALLTPGLRCLNLDVYRILLLMVGIGSLIREKRSAAAKKKGASLLCLALASTGLFNPMILAAGLIACDPNRKRGWPATEVMTAVGLMFAIVGGLAELDIDSMAIPMTIAGLMFAAFVISGKSTDMWIERTADISWESDAEITGSSERVDVRLDDDGNFQLMNDPGAPWKIWMLRMVCLAISAYTPWAILPSVVGFWITLQYTKRGGVLWDTPSPKEYKKGDTTTGVYRIMTRGLLGSYQAGAGVMVEGVFHTLWHTTKGAALMSGEGRLDPYWGSVKEDRLCYGGPWKLQHKWNGQDEVQMIVVEPGKNVKNVQTKPGVFKTPEGEIGAVTLDFPTGTSGSPIVDKNGDVIGLYGNGVIMPNGSYISAIVQGERMDEPIPAGFEPEMLRKKQITVLDLHPGAGKTRRILPQIIKEAINRRLRTAVLAPTRVVAAEMAEALRGLPIRYQTSAVPREHNGNEIVDVMCHATLTHRLMSPHRVPNYNLFVMDEAHFTDPASIAARGYISTKVELGEAAAIFMTATPPGTSDPFPESNSPISDLQTEIPDRAWNSGYEWITEYTGKTVWFVPSVKMGNEIALCLQRAGKKVVQLNRKSYETEYPKCKNDDWDFVITTDISEMGANFKASRVIDSRKSVKPTIITEGEGRVILGEPSAVTAASAAQRRGRIGRNPSQVGDEYCYGGHTNEDDSNFAHWTEARIMLDNINMPNGLIAQFYQPEREKVYTMDGEYRLRGEERKNFLELLRTADLPVWLAYKVAAAGVSYHDRRWCFDGPRTNTILEDNNEVEVITKLGERKILRPRWIDARVYSDHQALKAFKDFASGKRSQIGLIEVLGKMPEHFMGKTWEALDTMYVVATAEKGGRAHRMALEELPDALQTIALIALLSVMTMGVFFLLMQRKGIGKIGLGGAVLGVATFFCWMAEVPGTKIAGMLLLSLLLMIVLIPEPEKQRSQTDNQLAVFLICVMTLVSAVAANEMGWLDKTKSDISSLFGQRIEVKENFSMGEFLLDLRPATAWSLYAVTTAVLTPLLKHLITSDYINTSLTSINVQASALFTLARGFPFVDVGVSALLLAAGCWGQVTLTVTVTAATLLFCHYAYMVPGWQAEAMRSAQRRTAAGIMKNAVVDGIVATDVPELERTTPIMQKKVGQIMLILVSLAAVVVNPSVKTVREAGILITAAAVTLWENGASSVWNATTAIGLCHIMRGGWLSCLSITWTLIKNMEKPGLKRGGAKGRTLGEVWKERLNQMTKEEFTRYRKEAIIEVDRSAAKHARKEGNVTGGHPVSRGTAKLRWLVERRFLEPVGKVIDLGCGRGGWCYYMATQKRVQEVRGYTKGGPGHEEPQLVQSYGWNIVTMKSGVDVFYRPSECCDTLLCDIGESSSSAEVEEHRTIRVLEMVEDWLHRGPREFCVKVLCPYMPKVIEKMELLQRRYGGGLVRNPLSRNSTHEMYWVSRASGNVVHSVNMTSQVLLGRMEKRTWKGPQYEEDVNLGSGTRAVGKPLLNSDTSKIKNRIERLRREYSSTWHHDENHPYRTWNYHGSYDVKPTGSASSLVNGVVRLLSKPWDTITNVTTMAMTDTTPFGQQRVFKEKVDTKAPEPPEGVKYVLNETTNWLWAFLAREKRPRMCSREEFVRKVNSNAALGAMFEEQNQWRSAREAVEDPKFWEMVDEEREAHLRGECHTCIYNMMGKREKKPGEFGKAKGSRAIWFMWLGARFLEFEALGFLNEDHWLGRKNSGGGVEGLGLQKLGYILREVGTRPGGKIYADDTAGWDTRITRADLENEAKVLELLDGEHRRLARAIIELTYRHKVVKVMRPAADGRTVMDVISREDQRGSGQVVTYALNTFTNLAVQLVRMMEGEGVIGPDDVEKLTKGKGPKVRTWLFENGEERLSRMAVSGDDCVVKPLDDRFATSLHFLNAMSKVRKDIQEWKPSTGWYDWQQVPFCSNHFTELIMKDGRTLVVPCRGQDELVGRARISPGAGWNVRDTACLAKSYAQMWLLLYFHRRDLRLMANAICSAVPVNWVPTGRTTWSIHAGGEWMTTEDMLEVWNRVWIEENEWMEDKTPVEKWSDVPYSGKREDIWCGSLIGTRARATWAENIQVAINQVRAIIGDEKYVDYMSSLKRYEDTTLVEDTVL

>AHW48532.1 polyprotein [West Nile virus]

MSKKPGGPGKSRAVNMLKRGMPRVLSLIGLKRAMLSLIDGKGPIRFVLALLAFFRFTAIAPTRAVLDRWRGVNKQTAMKHLLSFKKELGTLTSAINRRSSKQKKRGGKTGIAVMIGLIASVGAVTLSNFQGKVMMTVNATDVTDVITIPTAAGKNLCIVRAMDVGYMCDDTITYECPVLSAGNDPEDIDCWCTKSAVYVRYGRCTKTRHSRRSRRSLTVQTHGESTLANKKGAWMDSTKATRYLVKTESWILRNPGYALVAAVIGWMLGSNTMQRVVFVVLLLLVAPAYSFNCLGMSNRDFLEGVSGATWVDLVLEGDSCVTIMSKDKPTIDVKMMNMEAANLAEVRSYCYLATVSDLSTKAACPTMGEAHNDKRADPAFVCRQGVVDRGWGNGCGLFGKGSIDTCAKFACSTKAIGRTILKENIKYEVAIFVHGPTTVESHGNYSTQVGATQAGRFSITPAAPSYTLKLGEYGEVTVDCEPRSGIDTNAYYVMTVGTKTFLVHREWFMDLNLPWSSAGSTVWRNRETLMEFEEPHATKQSVIALGSQEGALHQALAGAIPVEFSSNTVKLTSGHLKCRVKMEKLQLKGTTYGVCSKAFKFLGTPADTGHGTVVLELQYTGTDGPCKVPISSVASLNDLTPVGRLVTVNPFVSVATANAKVLIELEPPFGDSYIVVGRGEQQINHHWHKSGSSIGKAFTTTLKGAQRLAALGDTAWDFGSVGGVFTSVGKAVHQVFGGAFRSLFGGMSWITQGLLGALLLWMGINARDRSIALTFLAVGGVLLFLSVNVHADTGCAIDISRQELRCGSGVFIHNDVEAWMDRYKYYPETPQGLAKIIQKAHKEGVCGLRSVSRLEHQMWEAVKDELNTLLKENGVDLSVVVEKQEGMYKSAPKRLTATTEKLEIGWKAWGKSILFAPELANNTFVVDGPETKECPTQNRAWNSLEVEDFGFGLTSTRMFLKVRESNTTECDSKIIGTAVKNNLAIHSDLSYWIESRLNDTWKLERAVLGEVKSCTWPETHTLWGDGILESDLIIPVTLAGPRSNHNRRPGYKTQNQGPWDEGRVEIDFDYCPGTTVTLSESCGHRGPATRTTTESGKLITDWCCRSCTLPPLRYQTDSGCWYGMEIRPQRHDEKTLVQSQVNAYNADMIDPFQLGLLVVFLATQEVLRKRWTAKISMPAILIALLVLVFGGITYTDVLRYVILVGAAFAESNSGGDVVHLALMATFKIQPVFMVASFLKARWTNQENILLMLAAVFFQMAYHDARQILLWEIPDVLNSLAVAWMILRAITFTTTSNVVVPLLALLTPGLRCLNLDVYRILLLMVGIGSLIREKRSAAAKKKGASLLCLALASTGLFNPMILAAGLIACDPNRKRGWPATEVMTAVGLMFAIVGGLAELDIDSMAIPMTIAGLMFAAFVISGKSTDMWIERTADISWESDAEITGSSERVDVRLDDDGNFQLMNDPGAPWKIWMLRMVCLAISAYTPWAILPSVVGFWITLQYTKRGGVLWDTPSPKEYKKGDTTTGVYRIMTRGLLGSYQAGAGVMVEGVFHTLWHTTKGAALMSGEGRLDPYWGSVKEDRLCYGGPWKLQHKWNGQDEVQMIVVEPGKNVKNVQTKPGVFKTPEGEIGAVTLDFPTGTSGSPIVDKNGDVIGLYGNGVIMPNGSYISAIVQGERMDEPIPAGFEPEMLRKKQITVLDLHPGAGKTRRILPQIIKEAINRRLRTAVLAPTRVVAAEMAEALRGLPIRYQTSAVPREHNGNEIVDVMCHATLTHRLMSPHRVPNYNLFVMDEAHFTDPASIAARGYISTKVELGEAAAIFMTATPPGTSDPFPESNSPISDLQTEIPDRAWNSGYEWITEYTGKTVWFVPSVKMGNEIALCLQRAGKKVVQLNRKSYETEYPKCKNDDWDFVITTDISEMGANFKASRVIDSRKSVKPTIITEGEGRVILGEPSAVTAASAAQRRGRIGRNPSQVGDEYCYGGHTNEDDSNFAHWTEARIMLDNINMPNGLIAQFYQPEREKVYTMDGEYRLRGEERKNFLELLRTADLPVWLAYKVAAAGVSYHDRRWCFDGPRTNTILEDNNEVEVITKLGERKILRPRWIDARVYSDHQALKAFKDFASGKRSQIGLIEVLGKMPEHFMGKTWEALDTMYVVATAEKGGRAHRMALEELPDALQTIALIALLSVMTMGVFFLLMQRKGIGKIGLGGAVLGVATFFCWMAEVPGTKIAGMLLLSLLLMIVLIPEPEKQRSQTDNQLAVFLICVMTLVSAVAANEMGWLDKTKSDISSLFGQRIEVKENFSMGEFLLDLRPATAWSLYAVTTAVLTPLLKHLITSDYINTSLTSINVQASALFTLSRGFPFVDVGVSALLLAAGCWGQVTLTVTVTAATLLFCHYAYMVPGWQAEAMRSAQRRTAAGIMKNAVVDGIVATDVPELERTTPIMQKKVGQIMLILVSLAAVVVNPSVKTVREAGILITAAAVTLWENGASSVWNATTAIGLCHIMRGGWLSCLSITWTLIKNMEKPGLKRGGAKGRTLGEVWKERLNQMTKEEFTRYRKEAIIEVDRSAAKHARKEGNVTGGHPVSRGTAKLRWLVERRFLEPVGKVIDLGCGRGGWCYYMATQKRVQEVRGYTKGGPGHEEPQLVQSYGWNIVTMKSGVDVFYRPSECCDTLLCDIGESSSSAEVEEHRTIRVLEMVEDWLHRGPREFCVKVLCPYMPKVIEKMELLQRRYGGGLVRNPLSRNSTHEMYWVSRASGNVVHSVNMTSQVLLGRMEKRTWKGPQYEEDVNLGSGTRAVGKPLLNSDTSKIKNRIERLRREYSSTWHHDENHPYRTWNYHGSYDVKPTGSASSLVNGVVRLLSKPWDTITNVTTMAMTDTTPFGQQRVFKEKVDTKAPEPPEGVKYVLNETTNWLWAFLAREKRPRMCSREEFIRKVNSNAALGAMFEEQNQWRSAREAVEDPKFWEMVDEEREAHLRGECHTCIYNMMGKREKKPGEFGKAKGSRAIWFMWLGARFLEFEALGFLNEDHWLGRKNSGGGVEGLGLQKLGYILREVGTRPGGKIYADDTAGWDTRITRADLENEAKVLELLDGEHRRLARAIIELTYRHKVVKVMRPAADGRTVMDVISREDQRGSGQVVTYALNTFTNLAVQLVRMMEGEGVIGPDDVEKLTKGKGPKVRTWLFENGEERLSRMAVSGDDCVVKPLDDRFATSLHFLNAMSKVRKDIQEWKPSTGWYDWQQVPFCSNHFTELIMKDGRTLVVPCRGQDELVGRARISPGAGWNVRDTACLAKSYAQMWLLLYFHRRDLRLMANAICSAVPVNWVPTGRTTWSIHAGGEWMTTEDMLEVWNRVWIEENEWMEDKTPVEKWSDVPYSGKREDIWCGSLIGTRARATWAENIQVAINQVRAIIGDEKYVDYMSSLKRYEDTTLVEDTVL

>ANW68731.1 genome polyprotein [West Nile virus]

MSKKPGGPGKSRAVNMLKRGMPRVLSLIGLKRAMLSLIDGKGPIRFVLALLAFFRFTAIAPTRAVLDRWRGVNKQTAMKHLLSFKKELGTLTSAINRRSSKQKKRGGKTGIAVMIGLIASVGAVTLSNFQGKVMMTVNATDVTDVITIPTAAGKNLCIVRAMDVGYMCDDTITYECPVLSAGNDPEDIDCWCTKSAVYVRYGRCTKTRHSRRSRRSLTVQTHGESTLANKKGAWMDSTKATRYLVKTESWILRNPGYALVAAVIGWMLGSNTMQRVVFVVLLLLVAPAYSFNCLGMSNRDFLEGVSGATWVDLVLEGDSCVTIMSKDKPTIDVKMMNMEAANLAEVRSYCYLATVSDLSTKAACPTMGEAHNDKRADPAFVCRQGVVDRGWGNGCGLFGKGSIDTCAKFACSTKAIGRTILKENIKYEVAIFVHGPTTVESHGNYSTQVGATQAGRFSITPAAPSYTLKLGEYGEVTVDCEPRSGIDTNAYYVMTVGTKTFLVHREWFMDLNLPWSSAGSTVWRNRETLMEFEEPHATKQSVIALGSQEGALHQALAGAIPVEFSSNTVKLTSGHLKCRVKMEKLQLKGTTYGVCSKAFKFLGTPADTGHGTVVLELQYTGTDGPCKVPISSVASLNDLTPVGRLVTVNPFVSVATANAKVLIELEPPFGDSYIVVGRGEQQINHHWHKSGSSIGKAFTTTLKGAQRLAALGDTAWDFGSVGGVFTSVGKAVHQVFGGAFRSLFGGMSWITQGLLGALLLWMGINARDRSIALTFLAVGGVLLFLSVNVHADTGCAIDISRQELRCGSGVFIHNDVEAWMDRYKYYPETPQGLAKIIQKAHKEGVCGLRSVSRLEHQMWEAVKDELNTLLKENGVDLSVVVEKQEGMYKSAPKRLTATTEKLEIGWKAWGKSILFAPELANNTFVVDGPETKECPTQNRAWNSLEVEDFGFGLTSTRMFLKVRESNTTECDSKIIGTAVKNNLAIHSDLSYWIESRLNDTWKLERAVLGEVKSCTWPETHTLWGDGILESDLIIPVTLAGPRSNHNRRPGYKTQNQGPWDEGRVEIDFDYCPGTTVTLSESCGHRGPATRTTTESGKLITDWCCRSCTLPPLRYQTDSGCWYGMEIRPQRHDEKTLVQSQVNAYNADMIDPFQLGLLVVFLATQEVLRKRWTAKVSMPAILIALLVLVFGGITYTDVLRYVILVGAAFAESNSGGDVVHLALMATFKIQPVFMVASFLKARWTNQENILLMLAAVFFQMAYHDARQILLWEIPDVLNSLAVAWMILRAITFTTTSNVVVPLLALLTPGLRCLNLDVYRILLLMVGIGSLIREKRSAAAKKKGASLLCLALASTGLFNPMILAAGLIACDPNRKRGWPATEVMTAVGLMFAIVGGLAELDIDSMAIPMTIAGLMFAAFVISGKSTDMWIERTADISWESDAEITGSSERVDVRLDDDGNFQLMNDPGAPWKIWMLRMVCLAISAYTPWAILPSIVGFWITLQYTKRGGVLWDTPSPKEYKKGDTTTGVYRIMTRGLLGSYQAGAGVMVEGVFHTLWHTTKGAALMSGEGRLDPYWGSVKEDRLCYGGPWKLQHKWNGQDEVQMIVVEPGKNVKNVQTKPGVFKTPEGEIGAVTLDFPTGTSGSPIVDKNGDVIGLYGNGVIMPNGSYISAIVQGERMDEPIPAGFEPEMLRKKQITVLDLHPGAGKTRRILPQIIKEAINRRLRTAVLAPTRVVAAEMAEALRGLPIRYQTSAVPREHNGNEIVDVMCHATLTHRLMSPHRVPNYNLFVMDEAHFTDPASIAARGYISTKVELGEAAAIFMTATPPGTSDPFPESNSPISDLQTEIPDRAWNSGYEWITEYTGKTVWFVPSVKMGNEIALCLQRAGKKVVQLNRKSYETEYPKCKNDDWDFVITTDISEMGANFKASRVIDSRKSVKPTIITEGEGRVILGEPSAVTAASAAQRRGRIGRNPSQVGDEYCYGGHTNEDDSNFAHWTEARIMLDNINMPNGLIAQFYQPEREKVYTMDGEYRLRGEERKNFLELLRTADLPVWLAYKVAAAGVSYHDRRWCFDGPRTNTILEDNNEVEVITKLGERKILRPRWIDARVYSDHQALKAFKDFASGKRSQIGLIEVLGKMPEHFMGKTWEALDTMYVVATAEKGGRAHRMALEELPDALQTIALIALLSVMTMGVFFLLMQRKGIGKIGLGGAVLGVATFFCWMAEVPGTKIAGMLLLSLLLMIVLIPEPEKQRSQTDNQLAVFLICVMTLVSAVAANEMGWLDKTKSDISSLFGQRIEVKENFSMGEFLLDLRPATAWSLYAVTTAVLTPLLKHLITSDYINTSLTSINVQASALFTLARGFPFVDVGVSALLLAAGCWGQVTLTVTVTAATLLFCHYAYMVPGWQAEAMRSAQRRTAAGIMKNAVVDGIVATDVPELERTTPIMQKKVGQIMLILVSLAAVVVNPSVKTVREAGILITAAAVTLWENGASSVWNATTAIGLCHIMRGGWLSCLSITWTLIKNMEKPGLKRGGAKGRTLGEVWKERLNQMTKEEFTRYRKEAIIEVDRSAAKHARKEGNVTGGHPVSRGTAKLRWLVERRFLEPVGKVIDLGCGRGGWCYYMATQKRVQEVRGYTKGGPGHEEPQLVQSYGWNIVTMKSGVDVFYRPSECCDTLLCDIGESSSSAEVEEHRTIRVLEMVEDWLHRGPREFCVKVLCPYMPKVIEKMELLQRRYGGGLVRNPLSRNSTHEMYWVSRASGNVVHSVNMTSQVLLGRMEKRTWKGPQYEEDVNLGSGTRAVGKPLLNSDTSKIKNRIERLRREYSSTWHHDENHPYRTWNYHGSYDVKPTGSASSLVNGVVRLLSKPWDTITNVTTMAMTDTTPFGQQRVFKEKVDTKAPEPPEGVKYVLNETTNWLWAFLAREKRPRMCSREEFIRKVNSNAALGAMFEEQNQWRSAREAVEDPKFWEMVDEEREAHLRGECHTCIYNMMGKREKKPGEFGKAKGSRAIWFMWLGARFLEFEALGFLNEDHWLGRKNSGGGVEGLGLQKLGYILREVGTRPGGKIYADDTAGWDTRITRADLENEAKVLELLDGEHRRLARAIIELTYRHKVVKVMRPAADGRTVMDVISREDQRGSGQVVTYALNTFTNLAVQLVRMMEGEGVIGPDDVEKLTKGKGPKVRTWLFENGEERLSRMAVSGDDCVVKPLDDRFATSLHFLNAMSKVRKDIQEWKPSTGWYDWQQVPFCSNHFTELIMKDGRTLVVPCRGQDELVGRARISPGAGWNVRDTACLAKSYAQMWLLLYFHRRDLRLMANAICSAVPVNWVPTGRTTWSIHAGGEWMTTEDMLEVWNRVWIEENEWMEDKTPVEKWSDVPYSGKREDIWCGSLIGTRARATWAENIQVAINQVRAIIGDEKYVDYMSSLKRYEDTTLVEDTVL

>AHW48634.1 polyprotein [West Nile virus]

MSKKPGGPGKSRAVNMLKRGMPRVLSLIGLKRAMLSLIDGKGPIRFVLALLAFFRFTAIAPTRAVLDRWRGVNKQTAMKHLLSFKKELGTLTSAINRRSSKQKKRGGKTGIAVMIGLIASVGAVTLSNFQGKVMMTVNATDVTDVITIPTAAGKNLCIVRAMDVGYMCDDTITYECPVLSAGNDPEDIDCWCTKSAVYVRYGRCTKTRHSRRSRRSLTVQTHGESTLANKKGAWMDSTKATRYLVKTESWILRNPGYALVAAVIGWMLGSNTMQRVVFVVLLLLVAPAYSFNCLGMSNRDFLEGVSGATWVDLVLEGDSCVTIMSKDKPTIDVKMMNMEAANLAEVRSYCYLATVSDLSTKAACPTMGEAHNDKRADPAFVCRQGVVDRGWGNGCGLFGKGSIDTCAKFACSTKAIGRTILKENIKYEVAIFVHGPTTVESHGNYSTQAGATQAGRFSITPAAPSYTLKLGEYGEVTVDCEPRSGIDTNAYYVMTVGTKTFLVHREWFMDLNLPWSSAGSTVWRNRETLMEFEEPHATKQSVIALGSQEGALHQALAGAIPVEFSSNTVKLTSGHLKCRVKMEKLQLKGTTYGVCSKAFKFFGTPADTGHGTVVLELQYTGTDGPCKVPISSVASLNDLTPVGRLVTVNPFVSVATANAKVLIELEPPFGDSYIVVGRGEQQINHHWHKSGSSIGKAFTTTLKGAQRLAALGDTAWDFGSVGGVFTSVGKAVHQVFGGAFRSLFGGMSWITQGLLGALLLWMGINARDRSIALTFLAVGGVLLFLSVNVHADTGCAIDISRQELRCGSGVFIHNDVEAWMDRYKYYPETPQGLAKIIQKAHKEGVCGLRSVSRLEHQMWEAVKDELNTLLKENGVDLSVVVEKQEGMYKSAPKRLTATTEKLEIGWKAWGKSILFAPELANNTFVVDGPETKECPTQNRAWNSLEVEDFGFGLTSTRMFLKVRESNTTECDSKIIGTAVKNNLAIHSDLSYWIESRLNDTWKLERAVLGEVKSCTWPETHTLWGDGILESDLIIPVTLAGPRSNHNRRPGYKTQNQGPWDEGRVEIDFDYCPGTTVTLSESCGHRGPATRTTTESGKLITDWCCRSCTLPPLRYQTDSGCWYGMEIRPQRHDEKTLVQSQVNAYNADMIDPFQLGLLVVFLATQEVLRKRWTAKISMPAILIALLVLVFGGITYTDVLRYVILVGAAFAESNSGGDVVHLALMATFKIQPVFMVASFLKARWTNQENILLMLAAVFFQMAYHDARQILLWEIPDVLNSLAVAWMILRAITFTTTSNVVVPLLALLTPGLRCLNLDVYRILLLMVGIGSLIREKRSAAAKKKGASLLCLALASTGLFNPMILAAGLIACDPNRKRGWPATEVMTAVGLMFAIVGGLAELDIDSMAIPMTIAGLMFAAFVISGKSTDMWIERTADISWESDAEITGSSERVDVRLDDDGNFQLMNDPGAPWKIWMLRMVCLAISAYTPWAILPSIVGFWITLQYTKRGGVLWDTPSPKEYKKGDTTTGVYRIMTRGLLGSYQAGAGVMVEGVFHTLWHTTKGAALMSGEGRLDPYWGSVKEDRLCYGGPWKLQHKWNGQDEVQMIVVEPGKNVKNVQTKPGVFKTPEGEIGAVTLDFPTGTSGSPIVDKNGDVIGLYGNGVIMPNGSYISAIVQGERMDEPIPAGFEPEMLRKKQITVLDLHPGAGKTRRILPQIIKEAINRRLRTAVLAPTRVVAAEMAEALRGLPIRYQTSAVPREHNGNEIVDVMCHATLTHRLMSPHRVPNYNLFVMDEAHFTDPASIAARGYISTKVELGEAAAIFMTATPPGTSDPFPESNSPISDLQTEIPDRAWNSGYEWITEYTGKTVWFVPSVKMGNEIALCLQRAGKKVVQLNRKSYETEYPKCKNDDWDFVITTDISEMGANFKASRVIDSRKSVKPTIITEGEGRVILGEPSAVTAASAAQRRGRIGRNPSQVGDEYCYGGHTNEDDSNFAHWTEARIMLDNINMPNGLIAQFYQPEREKVYTMDGEYRLRGEERKNFLELLRTADLPVWLAYKVAAAGVSYHDRRWCFDGPRTNTILEDNNEVEVITKLGERKILRPRWIDARVYSDHQALKAFKDFASGKRSQIGLIEVLGKMPEHFMGKTWEALDTMYVVATAEKGGRAHRMALEELPDALQTIALIALLSVMTMGVFFLLMQRKGIGKIGLGGAVLGVATFFCWMAEVPGTKIAGMLLLSLLLMIVLIPEPEKQRSQTDNQLAVFLICVMTLVSAVAANEMGWLDKTKSDISSLFGQRIEVKENFSMGEFLLDLRPATAWSLYAVTTAVLTPLLKHLITSDYINTSLTSINVQASALFTLARGFPFVDVGVSALLLAAGCWGQVTLTVTVTAATLLFCHYAYMVPGWQAEAMRSAQRRTAAGIMKNAVVDGIVATDVPELERTTPIMQKKVGQIMLILVSLAAVVVNPSVKTVREAGILITAAAVTLWENGASSVWNATTAIGLCHIMRGGWLSCLSITWTLIKNMEKPGLKRGGAKGRTLGEVWKERLNQMTKEEFTRYRKEAIIEVDRSAAKHARKEGNVTGGHPVSRGTAKLRWLVERRFLEPVGKVIDLGCGRGGWCYYMATQKRVQEVRGYTKGGPGHEEPQLVQSYGWNIVTMKSGVDVFYRPSECCDTLLCDIGESSSSAEVEEHRTIRVLEMVEDWLHRGPREFCVKVLCPYMPKVIEKMELLQRRYGGGLVRNPLSRNSTHEMYWVSRASGNVVHSVNMTSQVLLGRMEKRTWKGPQYEEDVNLGSGTRAVGKPLLNSDTSKIKNRIERLRREYSSTWHHDENHPYRTWNYHGSYDVKPTGSASSLVNGVVRLLSKPWDTITNVTTMAMTDTTPFGQQRVFKEKVDTKAPEPPEGVKYVLNETTNWLWAFLAREKRPRMCSREEFIRKVNSNAALGAMFEEQNQWRSAREAVEDPKFWEMVDEEREAHLRGECHTCIYNMMGKREKKPGEFGKAKGSRAIWFMWLGARFLEFEALGFLNEDHWLGRKNSGGGVEGLGLQKLGYILREVGTRPGGKIYADDTAGWDTRITRADLENEAKVLELLDGEHRRLARAIIELTYRHKVVKVMRPAADGRTVMDVISREDQRGSGQVVTYALNTFTNLAVQLVRMMEGEGVIGPDDVEKLTKGKGPKVRTWLFENGEERLSRMAVSGDDCVVKPLDDRFATSLHFLNAMSKVRKDIQEWKPSTGWYDWQQVPFCSNHFTELIMKDGRTLVVPCRGQDELVGRARISPGAGWNVRDTACLAKSYAQMWLLLYFHRRDLRLMANAICSAVPVNWVPTGRTTWSIHAGGEWMTTEDMLEVWNRVWIEENEWMEDKTPVEKWSDVPYSGKREDIWCGSLIGTRARATWAENIQVAINQVRAIIGDEKYVDYMSSLKRYEDTTLVEDTVL

>ANW68881.1 genome polyprotein [West Nile virus]

MSKKPGGPGKSRAVNMLKRGMPRVLSLIGLKRAMLSLIDGKGPIRFVLALLAFFRFTAIAPTRAVLDRWRGVNKQTAMKHLLSFKKELGTLTSAINRRSSKQKKRGGKTGIAVMIGLIASVGAVTLSNFQGKVMMTVNATDVTDVITIPTAAGKNLCIVRAMDVGYMCDDTITYECPVLSAGNDPEDIDCWCTKSAVYVRYGRCTKTRHSRRSRRSLTVQTHGESTLANKKGAWMDSTKATRYLVKTESWILRNPGYALVAAVIGWMLGSNTMQRVVFVVLLLLVAPAYSFNCLGMSNRDFLEGVSGATWVDLVLEGDSCVTIMSKDKPTIDVKMMNMEAANLAEVRSYCYLATVSDLSTKAACPTMGEAHNDKRADPAFVCRQGVVDRGWGNGCGLFGKGSIDTCAKFACSTKAIGRTILKENIKYEVAIFVHGPTTVESHGNYSTQVGATQAGRFSITPAAPSYTLKLGEYGEVTVDCEPRSGIDTNAYYVMTVGTKTFLVHREWFMDLNLPWSSAGSTVWRNRETLMEFEEPHATKQSVIALGSQEGALHQALAGAIPVEFSSNTVKLTSGHLKCRVKMEKLQLKGTTYGVCSKAFKFLGTPADTGHGTVVLELQYTGTDGPCKVPISSVASLNDLTPVGRLVTVNPFVSVATANAKVLIELEPPFGDSYIVVGRGEQQINHHWHKSGSSIGKAFTTTLKGAQRLAALGDTAWDFGSVGGVFTSVGKAVHQVFGGAFRSLFGGMSWITQGLLGALLLWMGINARDRSIALTFLAVGGVLLFLSVNVHADTGCAIDISRQELRCGSGVFIHNDVEAWMDRYKYYPETPQGLAKIIQKAHKEGVCGLRSVSRLEHQMWEAVKDELNTLLKENGVDLSVVVEKQEGMYKSAPKRLTATTEKLEIGWKAWGKSILFAPELANNTFVVDGPETKECPTQNRAWNSLEVEDFGFGLTSTRMFLKVRESNTTECDSKIIGTAVKNNLAIHSDLSYWIESRLNDTWKLERAVLGEVKSCTWPETHTLWGDGILESDLIIPVTLAGPRSNHNRRPGYKTQNQGPWDEGRVEIDFDYCPGTTVTLSESCGHRGPATRTTTESGKLITDWCCRSCTLPPLRYQTDSGCWYGMEIRPQRHDEKTLVQSQVNAYNADMIDPFQLGLLVVFLATQEVLRKRWTAKISMPAILIALLVLVFGGITYTDVLRYVILVGAAFAESNSGGDVVHLALMATFKIQPVFMVASFLKARWTNQENILLMLAAVFFQMAYHDARQILLWEIPDVLNSLAVAWMILRAITFTTTSNVVVPLLALLTPGLRCLNLDVYRILLLMVGIGSLIREKRSAAAKKKGASLLCLALASTGLFNPMILAAGLIACDPNRKRGWPATEVMTAVGLMFAIVGGLAELDIDSMAIPMTIAGLMFAAFVISGKSTDMWIERTADISWESDAEITGSSERVDVRLDDDGNFQLMNDPGAPWKIWMLRMVCLAISAYTPWAILPSVVGFWITLQYTKRGGVLWDTPSPKEYKKGDTTTGVYRIMTRGLLGSYQAGAGVMVEGVFHTLWHTTKGAALMSGEGRLDPYWGSVKEDRLCYGGPWKLQHKWNGQDEVQMIVVEPGKNVKNVQTKPGVFKTPEGEIGAVTLDFPTGTSGSPIVDKNGDVIGLYGNGVIMPNGSYISAIVQGERMDEPIPAGFEPEMLRKKQITVLDLHPGAGKTRRILPQIIKEAINRRLRTAVLAPTRVVAAEMAEALRGLPIRYQTSAVPREHNGNEIVDVMCHATLTHRLMSPHRVPNYNLFVMDEAHFTDPASIAARGYISTKVELGEAAAIFMTATPPGTSDPFPESNSPISDLQTEIPDRAWNSGYEWITEYTGKTVWFVPSVKMGNEIALCLQRAGKKVVQLNRKSYETEYPKCKNDDWDFVITTDISEMGANFKASRVIDSRKSVKPTIITEGEGRVVLGEPSAVTAASAAQRRGRIGRNPSQVGDEYCYGGHTNEDDSNFAHWTEARIMLDNINMPNGLIAQFYQPEREKVYTMDGEYRLRGEERKNFLELLRTADLPVWLAYKVAAAGVSYHDRRWCFDGPRTNTILEDNNEVEVITKLGERKILRPRWIDARVYSDHQALKAFKDFASGKRSQIGLIEVLGKMPEHFMGKTWEALDTMYVVATAEKGGRAHRMALEELPDALQTIALIALLSVMTMGVFFLLMQRKGIGKIGLGGAVLGVATFFCWMAEVPGTKIAGMLLLSLLLMIVLIPEPEKQRSQTDNQLAVFLICVMTLVSAVAANEMGWLDKTKSDISSLFGQRIEVKENFSMGEFLLDLRPATAWSLYAVTTAVLTPLLKHLITSDYINTSLTSINVQASALFTLARGFPFVDVGVSALLLAAGCWGQVTLTVTVTAATLLFCHYAYMVPGWQAEAMRSAQRRTAAGIMKNAVVDGIVATDVPELERTTPIMQKKVGQIMLILVSLAAVVVNPSVKTVREAGILITAAAVTLWENGASSVWNATTAIGLCHIMRGGWLSCLSITWTLIKNMEKPGLKRGGAKGRTLGEVWKERLNQMTKEEFTRYRKEAIIEVDRSAAKHARKEGNITGGHPVSRGTAKLRWLVERRFLEPVGKVIDLGCGRGGWCYYMATQKRVQEVRGYTKGGPGHEEPQLVQSYGWNIVTMKSGVDVFYRPSECCDTLLCDIGESSSSAEVEEHRTIRVLEMVEDWLHRGPREFCVKVLCPYMPKVIEKMELLQRRYGGGLVRNPLSRNSTHEMYWVSRASGNVVHSVNMTSQVLLGRMEKRTWKGPQYEEDVNLGSGTRAVGKPLLNSDTSKIKNRIERLRREYSSTWHHDENHPYRTWNYHGSYDVKPTGSASSLVNGVVRLLSKPWDTITNVTTMAMTDTTPFGQQRVFKEKVDTKAPEPPEGVKYVLNETTNWLWAFLAREKRPRMCSREEFIRKVNSNAALGAMFEEQNQWRSAREAVEDPKFWEMVDEEREAHLRGECHTCIYNMMGKREKKPGEFGKAKGSRAIWFMWLGARFLEFEALGFLNEDHWLGRKNSGGGVEGLGLQKLGYILREVGTRPGGKIYADDTAGWDTRITRADLENEAKVLELLDGEHRRLARAIIELTYRHKVVKVMRPAADGRTVMDVISREDQRGSGQVVTYALNTFTNLAVQLVRMMEGEGVIGPDDVEKLTKGKGPKVRTWLFENGEERLSRMAVSGDDCVVKPLDDRFATSLHFLNAMSKVRKDIQEWKPSTGWYDWQQVPFCSNHFTELIMKDGRTLVVPCRGQDELVGRARISPGAGWNVRDTACLAKSYAQMWLLLYFHRRDLRLMANAICSAVPVNWVPTGRTTWSIHAGGEWMTTEDMLEVWNRVWIEENEWMEDKTPVEKWSDVPYSGKREDIWCGSLIGTRARATWAENIQVAINQVRAIIGDEKYVDYMSSLKRYEDTTLVEDTVL

>ACV90477.1 polyprotein [West Nile virus]

MSKKPGGPGKSRAVNMLKRGMPRVLSLIGLKRAMLSLIDGKGPIRFVLALLAFFRFTAIAPTRAVLDRWRGVNKQTAMKHLLSFKKELGTLTSAINRRSSKQKKRGGKTGIAVMIGLIASVGAVTLSNFQGKVMMTVNATDVTDVITIPTAAGKNLCIVRAMDVGYMCDDTITYECPVLSAGNDPEDIDCWCTKSAVYVRYGRCTKTRHSRRSRRSLTVQTHGESTLANKKGAWMDSTKATRYLVKTESWILRNPGYALVAAVIGWMLGSNTMQRVVFVVLLLLVAPAYSFNCLGMSNRDFLEGVSGATWVDLVLEGDSCVTIMSKDKPTIDVKMMNMEAANLAEVRSYCYLATVSDLSTKAACPTMGEAHNDKRADPAFVCRQGVVDRGWGNGCGLFGKGSIDTCAKFACSTKAIGRTILKENIKYEVAIFVHGPTTVESHGNYSTQAGATQAGRFSITPAAPSYTLKLGEYGEVTVDCEPRSGIDTNAYYVMTVGTKTFLVHREWFMDLNLPWSSAGSTVWRNRETLMEFEEPHATKQSVIALGSQEGALHQALAGAIPVEFSSNTVKLTSGHLKCRVKMEKLQLKGTTYGVCSKAFKFLGTPADTGHGTVVLELQYTGTDGPCKVPISSVASLNDLTPVGRLVTVNPFVSVATANAKVLIELEPPFGDSYIVVGRGEQQINHHWHKSGSSIGKAFTTTLKGAQRLAALGDTAWDFGSIGGVFTSVGKAVHQVFGGAFRSLFGGMSWITQGLLGALLLWMGINARDRSIALTFLAVGGVLLFLSVNVHADTGCAIDISRQELRCGSGVFIHNDVEAWMDRYKYYPETPQGLAKIIQKAHKEGVCGLRSVSRLEHQMWEAVKDELNTLLKENGVDLSVVVEKQEGMYKSAPKRLTATTEKLEIGWKAWGKSILFAPELANNTFVVDGPETKECPTQNRAWNSLEVEDFGFGLTSTRMFLKVRESNTTECDSKIIGTAVKNNLAIHSDLSYWIESRLNDTWKLERAVLGEVKSCTWPETHTLWGDGILESDLIIPVTLAGPRSNHNRRPGYKTQNQGPWDEGRVEIDFDYCPGTTVTLSESCGHRGPATRTTTESGKLITDWCCRSCTLPPLRYQTDSGCWYGMEIRPQRHDEKTLVQSQVNAYNADMIDPFQLGLLVVFLATQEVLRKRWTAKISMPAILIALLVLVFGGITYTDVLRYVILVGAAFAESNSGGDVVHLALMATFKIQPVFMVASFFKARWTNQENILLMLAAVFFQMAYHDARQILLWEIPDVLNSLAVAWMILRAITFTTTSNVVVPLLALLTPGLRCLNLDVYRILLLMVGIGSLIREKRSAAAKKKGASLLCLALASTGLFNPMILAAGLIACDPNRKRGWPATEVMTAVGLMFAIVGGLAELDIDSMAIPMTIAGLMFAAFVISGKSTDMWIERTADISWESDAEITGSSERVDVRLDDDGNFQLMNDPGAPWKIWMLRMVCLAISAYTPWAILPSVVGFWITLQYTKRGGVLWDTPSPKEYKKGDTTTGVYRIMTRGLLGSYQAGAGVMVEGVFHTLWHTTKGAALMSGEGRLDPYWGSVKEDRLCYGGPWKLQHKWNGQDEVQMIVVEPGKNVKNVQTKPGVFKTPEGEIGAVTLDFPTGTSGSPIVDKNGDVIGLYGNGVIMPNGSYISAIVQGERMDEPIPAGFEPEMLRKKQITVLDLHPGAGKTRRILPQIIKEAINRRLRTAVLAPTRVVAAEMAEALRGLPIRYQTSAVPREHNGNEIVDVMCHATLTHRLMSPHRVPNYNLFVMDEAHFTDPASIAARGYISTKVELGEAAAIFMTATPPGTSDPFPESNSPISDLQTEIPDRAWNSGYEWITEYTGKTVWFVPSVKMGNEIALCLQRAGKKVVQLNRKSYETEYPKCKNDDWDFVITTDISEMGANFKASRVIDSRKSVKPTIITEGEGRVILGEPSAVTAASAAQRRGRIGRNPSQVGDEYCYGGHTNEDDSNFAHWTEARIMLDNINMPNGLIAQFYQPEREKVYTMDGEYRLRGEERKNFLELLRTADLPVWLAYKVAAAGVSYHDRRWCFDGPRTNTILEDNNEVEVITKLGERKILRPRWIDARVYSDHQALKAFKDFASGKRSQIGLIEVLGKMPEHFMGKTWEALDTMYVVATAEKGGRAHRMALEELPDALQTIALIALLSVMTMGVFFLLMQRKGIGKIGLGGAVLGVATFFCWMAEVPGTKIAGMLLLSLLLMIVLIPEPEKQRSQTDNQLAVFLICVMTLVSAVAANEMGWLDKTKSDISSLFGQRIEVKENFSMGEFLLDLRPATAWSLYAVTTAVLTPLLKHLITSDYINTSLTSINVQASALFTLARGFPFVDVGVSALLLAAGCWGQVTLTVTVTAATLLFCHYAYMVPGWQAEAMRSAQRRTAAGIMKNAVVDGIVATDVPELERTTPIMQKKVGQIMLILVSLAAVVVNPSVKTVREAGILITAAAVTLWENGASSVWNATTAIGLCHIMRGGWLSCLSITWTLIKNMEKPGLKRGGAKGRTLGEVWKERLNQMTKEEFTRYRKEAIIEVDRSAAKHARKEGNVTGGHPVSRGTAKLRWLVERRFLEPVGKVIDLGCGRGGWCYYMATQKRVQEVRGYTKGGPGHEEPQLVQSYGWNIVTMKSGVDVFYRPSECCDTLLCDIGESSSSAEVEEHRTIRVLEMVEDWLHRGPREFCVKVLCPYMPKVIEKMELLQRRYGGGLVRNPLSRNSTHEMYWVSRASGNVVHSVNMTSQVLLGRMEKRTWKGPQYEEDVNLGSGTRAVGKPLLNSDTSKIKNRIERLRREYSSTWHHDENHPYRTWNYHGSYDVKPTGSASSLVNGVVRLLSKPWDTITNVTTMAMTDTTPFGQQRVFKEKVDTKAPEPPEGVKYVLNETTNWLWAFLAREKRPRMCSREEFIRKVNSNAALGAMFEEQNQWRSAREAVEDPKFWEMVDEEREAHLRGECHTCIYNMMGKREKKPGEFGKAKGSRAIWFMWLGARFLEFEALGFLNEDHWLGRKNSGGGVEGLGLQKLGYILREVGTRPGGKIYADDTAGWDTRITRADLENEAKVLELLDGEHRRLARAIIELTYRHKVVKVMRPAADGRTVMDVISREDQRGSGQVVTYALNTFTNLAVQLVRMMEGEGVIGPDDVEKLTKGKGPKVRTWLFENGEERLSRMAVSGDDCVVKPLDDRFATSLHFLNAMSKVRKDIQEWKPSTGWYDWQQVPFCSNHFTELIMKDGRTLVVPCRGQDELVGRARISPGAGWNVRDTACLAKSYAQMWLLLYFHRRDLRLMANAICSAVPVNWVPTGRTTWSIHAGGEWMTTEDMLEVWNRVWIEENEWMEDKTPVEKWSDVPYSGKREDIWCGSLIGTRARATWAENIQVAINQVRAIIGDEKYVDYMSSLKRYEDTTLVEDTVL

>ADU76252.1 polyprotein [West Nile virus]

MSKKPGGPGKSRAVNMLKRGMPRVLSLIGLKRAMLSLIDGKGPIRFVLALLAFFRFTAIAPTRAVLDRWRGVNKQTAMKHLLSFKKELGTLTSAINRRSSKQKKRGGKTGIAVMIGLIASVGAVTLSNFQGKVMMTVNATDVTDVITIPTAAGKNLCIVRAMDVGYMCDDTITYECPVLSAGNDPEDIDCWCTKSAVYVRYGRCTKTRHSRRSRRSLTVQTHGESTLANKKGAWMDSTKATRYLVKTESWILRNPGYALVAAVIGWMLGSNTMQRVVFVVLLLLVAPAYSFNCLGMSNRDFLEGVSGATWVDLVLEGDSCVTIMSKDKPTIDVKMMNMEAANLAEVRSYCYLATVSDLSTKAACPTMGEAHNDKRADPAFVCRQGVVDRGWGNGCGLFGKGSIDTCAKFACSTKAIGRTILKENIKYEVAIFVHGPTTVESHGNYSTQVGATQAGRFSITPAAPSYTLKLGEYGEVTVDCEPRSGIDTNAYYVMTVGTKTFLVHREWFMDLNLPWSSAGSTVWRNRETLMEFEEPHATKQSVIALGSQEGALHQALAGAIPVEFSSNTVKLTSGHLKCRVKMEKLQLKGTTYGVCSKAFKFLGTPADTGHGTVVLELQYTGTDGPCKVPISSVASLNDLTPVGRLVTVNPFVSVATANAKVLIELEPPFGDSYIVVGRGEQQINHHWHKSGSSIGKAFTTTLKGAQRLAALGDTAWDFGSVGGVFTSVGKAVHQVFGGAFRSLFGGMSWITQGLLGALLLWMGINARDRSIALTFLAVGGVLLFLSVNVHADTGCAIDISRQELRCGSGVFIHNDVEAWMDRYKYYPETPQGLAKIIQKAHKEGVCGLRSVSRLEHQMWEAVKDELNTLLKENGVDLSVVVEKQEGMYKSAPKRLTATTEKLEIGWKAWGKSILFAPELANNTFVVDGPETKECPTQNRAWNSLEVEDFGFGLTSTRMFLKVRESNTTECDSKIIGTAVKNNLAIHSDLSYWIESRLNDTWKLERAVLGEVKSCTWPETHTLWGDGILESDLIIPVTLAGPRSNHNRRPGYKTQNQGPWDEGRVEIDFDYCPGTTVTLSESCGHRGPATRTTTESGKLITDWCCRSCTLPPLRYQTDSGCWYGMEIRPQRHDEKTLVQSQVNAYNADMIDPFQLGLLVVFLATQEVLRKRWTAKISMPAILIALLVLVFGGITYTDVLRYVILVGAAFAESNSGGDVVHLALMATFKIQPVFMVASFLKARWTNQENILLMLAAVFFQMAYHDARQILLWEIPDVLNSLAVAWMILRAITFTTTSNVVVPLLALLTPGLRCLNLDVYRILLLMVGIGSLIREKRSAAAKKKGASLLCLALASTGLFNPMILAAGLIACDPNRKRGWPATEVMTAVGLMFAIVGGLAELDIDSMAIPMTIAGLMFAAFVISGKSTDMWIERTADISWESDAEITGSSERVDVRLDDDGNFQLMNDPGAPWKIWMLRMVCLAISAYTPWAILPSVVGFWITLQYTKRGGVLWDTPSPKEYKKGDTTTGVYRIMTRGLLGSYQAGAGVMVEGVFHTLWHTTKGAALMSGEGRLDPYWGSVKEDRLCYGGPWKLQHKWNGQDEVQMIVVEPGKNVKNVQTKPGVFKTPEGEIGAVTLDFPTGTSGSPIVDKNGDVIGLYGNGVIMPNGSYISAIVQGERMDEPIPAGFEPEMLRKKQITVLDLHPGAGKTRRILPQIIKEAINRRLRTAVLAPTRVVAAEMAEALRGLPIRYQTSAVPREHNGNEIVDVMCHATLTHRLMSPHRVPNYNLFVMDEAHFTDPASIAARGYISTKVELGEAAAIFMTATPPGTSDPFPESNSPISDLQTEIPDRAWNSGYEWITEYTGKTVWFVPSVKMGNEIALCLQRAGKKVVQLNRKSYETEYPKCKNDDWDFVITTDISEMGANFKASRVIDSRKSVKPTIITEGEGRVILGEPSAVTAASAAQRRGRIGRNPSQVGDEYCYGGHTNEDDSNFAHWTEARIMLDNINMPNGLIAQFYQPEREKVYTMDGEYRLRGEERKNFLELLRTADLPVWLAYKVAAAGVSYHDRRWCFDGPRTNTILEDNNEVEVITKLGERKILRPRWIDARVYSDHQALKAFKDFASGKRSQIGLIEVLGKMPEHFMGKTWEALDTMYVVATAEKGGRAHRMALEELPDALQTIALIALLSVMTMGVFFLLMQRKGIGKIGLGGAVLGVATFFCWMAEVPGTKIAGMLLLSLLLMIVLIPEPEKQRSQTDNQLAVFLICVMTLVSAVAANEMGWLDKTKSDISSLFGQRIEVKENFSMGEFLLDLRPATAWSLYAVTTAVLTPLLKHLITSDYINTSLTSINVQASALFTLARGFPFVDVGVSALLLAAGCWGQVTLTVTVTAATLLFCHYAYMVPGWQAEAMRSAQRRTAAGIMKNAVVDGIVATDVPELERTTPIMQKKVGQIMLILVSLAAVVVNPSVKTVREAGILITAAAVTLWENGASSVWNATTAIGLCHIMRGGWLSCLSITWTLIKNMEKPGLKRGGAKGRTLGEVWKERLNQMTKEEFTRYRKEAIIEVDRSAAKHARKEGNVTGGHPVSRGTAKLRWLVERRFLEPVGKVIDLGCGRGGWCYYMATQKRVQEVRGYTKGGPGHEEPQLVQSYGWNIVTMKSGVDVFYRPSECCDTLLCDIGESSSSAEVEEHRTIRVLEMVEDWLHRGPREFCVKVLCPYMPKVIEKMELLQRRYGGGLVRNPLSRNSTHEMYWVSRASGNVVHSVNMTSQVLLGRMEKRTWKGPQYEEDVNLGSGTRAVGKPLLNSDTSKIKNRIERLKREYSSTWHHDENHPYRTWNYHGSYDVKPTGSASSLVNGVVRLLSKPWDTITNVTTMAMTDTTPFGQQRVFKEKVDTKAPEPPEGVKYVLNETTNWLWAFLAREKRPRMCSREEFIRKVNSNAALGAMFEEQNQWRSAREAVEDPKFWEMVDEEREAHLRGECHTCIYNMMGKREKKPGEFGKAKGSRAIWFMWLGARFLEFEALGFLNEDHWLGRKNSGGGVEGLGLQKLGYILREVGTRPGGKIYADDTAGWDTRITRADLENEAKVLELLDGEHRRLARAIIELTYRHKVVKVMRPAADGRTVMDVISREDQRGSGQVVTYALNTFTNLAVQLVRMMEGEGVIGPDDVEKLTKGKGPKVRTWLFENGEERLSRMAVSGDDCVVKPLDDRFATSLHFLNAMSKVRKDIQEWKPSTGWYDWQQVPFCSNHFTELIMKDGRTLVVPCRGQDELVGRARISPGAGWNVRDTACLAKSYAQMWLLLYFHRRDLRLMANAICSAVPVNWVPTGRTTWSIHAGGEWMTTEDMLEVWNRVWIEENEWMEDKTPVEKWSDVPYSGKREDIWCGSLIGTRARATWAENIQVAINQVRAIIGDEKYVDYMSSLKRYEDTTLVEDTVL

>ADL27943.1 polyprotein [West Nile virus]

MSKKPGGPGKSRAVNMLKRGMPRVLSLIGLKRAMLSLIDGKGPIRFVLALLAFFRFTAIAPTRAVLDRWRGVNKQTAMKHLLSFKKELGTLTSAINRRSSKQKKRGGKTGIAVMIGLIASVGAVTLSNFQGKVMMTVNATDVTDVITIPTAAGKNLCIVRAMDVGYMCDDTITYECPVLSAGNDPEDIDCWCTKSAVYVRYGRCTKTRHSRRSRRSLTVQTHGESTLANKKGAWMDSTKATRYLVKTESWILRNPGYALVAAVIGWMLGSNTMQRVVFVVLLLLVAPAYSFNCLGMSNRDFLEGVSGATWVDLVLEGDSCVTIMSKDKPTIDVKMMNMEAANLAEVRSYCYLATVSDLSTKAACPTMGEAHNDKRADPAFVCRQGVVDRGWGNGCGLFGKGSIDTCAKFACSTKAIGRTILKENIKYEVAIFVHGPTTVESHGNYSTQVGATQAGRFSITPAAPSYTLKLGEYGEVTVDCEPRSGIDTNAYYVMTVGTKTFLVHREWFMDLNLPWSSAGSTVWRNRETLMEFEEPHATKQSVIALGSQEGALHQALAGAIPVEFSSNTVKLTSGHLKCRMKMEKLQLKGTTYGVCSKAFKFLGTPADTGHGTVVLELQYTGTDGPCKVPISSVASLNDLTPVGRLVTVNPFVSVATANAKVLIELEPPFGDSYIVVGRGEQQINHHWHKSGSSIGKAFTTTLKGAQRLAALGDTAWDFGSVGGVFTSVGKAVHQVFGGAFRSLFGGMSWITQGLLGALLLWMGINARDRSIALTFLAVGGVLLFLSVNVHADTGCAIDISRQELRCGSGVFIHNDVEAWMDRYKYYPETPQGLAKIIQKAHKEGVCGLRSVSRLEHQMWEAVKDELNTLLKENGVDLSVVVEKQEGMYKSAPKRLTATTEKLEIGWKAWGKSILFAPELANNTFVVDGPETKECPTQNRAWNSLEVEDFGFGLTSTRMFLKVRESNTTECDSKIIGTAVKNNLAIHSDLSYWIESRLNDTWKLERAVLGEVKSCTWPETHTLWGDGILESDLIIPVTLAGPRSNHNRRPGYKTQNQGPWDEGRVEIDFDYCPGTTVTLSESCGHRGPATRTTTESGKLITDWCCRSCTLPPLRYQTDSGCWYGMEIRPQRHDEKTLVQSQVNAYNADMIDPFQLGLLVVFLATQEVLRKRWTAKISMPAILIALLVLVFGGITYTDVLRYVILVGAAFAESNSGGDVVHLALMATFKIQPVFMVASFLKARWTNQENILLMLAAVFFQMAYHDARQILLWEIPDVLNSLAVAWMILRAITFTTTSNVVVPLLALLTPGLRCLNLDVYRILLLMVGIGSLIREKRSAAAKKKGASLLCLALASTGLFNPMILAAGLIACDPNRKRGWPATEVMTAVGLMFAIVGGLAELDIDSMAIPMTIAGLMFAAFVISGKSTDMWIERTADISWESDAEITGSSERVDVRLDDDGNFQLMNDPGAPWKIWMLRMVCLAISAYTPWAILPSVVGFWITLQYTKRGGVLWDTPSPKEYKKGDTTTGVYRIMTRGLLGSYQAGAGVMVEGVFHTLWHTTKGAALMSGEGRLDPYWGSVKEDRLCYGGPWKLQHKWNGQDEVQMIVVEPGKNVKNVQTKPGVFKTPEGEIGAVTLDFPTGTSGSPIVDKNGDVIGLYGNGVIMPNGSYISAIVQGERMDEPIPAGFEPEMLRKKQITVLDLHPGAGKTRRILPQIIKEAINRRLRTAVLAPTRVVAAEMAEALRGLPIRYQTSAVPREHNGNEIVDVMCHATLTHRLMSPHRVPNYNLFVMDEAHFTDPASIAARGYISTKVELGEAAAIFMTATPPGTSDPFPESNSPISDLQTEIPDRAWNSGYEWITEYTGKTVWFVPSVKMGNEIALCLQRAGKKVVQLNRKSYETEYPKCKNDDWDFVITTDISEMGANFKASRVIDSRKSVKPTIITEGEGRVILGEPSAVTAASAAQRRGRIGRNPSQVGDEYCYGGHTNEDDSNFAHWTEARIMLDNINMPNGLIAQFYQPEREKVYTMDGEYRLRGEERKNFLELLRTADLPVWLAYKVAAAGVSYHDRRWCFDGPRTNTILEDNNEVEVITKLGERKILRPRWIDARVYSDHQALKAFKDFASGKRSQIGLIEVLGKMPEHFMGKTWEALDTMYVVATAEKGGRAHRMALEELPDALQTIALIALLSVMTMGVFFLLMQRKGIGKIGLGGAVLGVATFFCWMAEVPGTKIAGMLLLSLLLMIVLIPEPEKQRSQTDNQLAVFLICVMTLVSAVAANEMGWLDKTKSDISSLFGQRIEVKENFSMGEFLLDLRPATAWSLYAVTTAVLTPLLKHLITSDYINTSLTSINVQASALFTLARGFPFVDVGVSALLLAAGCWGQVTLTVTVTAATLLFCHYAYMVPGWQAEAMRSAQRRTAAGIMKNAVVDGIVATDVPELERTTPIMQKKVGQIMLILVSLAAVVVNPSVKTVREAGILITAAAVTLWENGASSVWNATTAIGLCHIMRGGWLSCLSITWTLIKNMEKPGLKRGGAKGRTLGEVWKERLNQMTKEEFTRYRKEAIIEVDRSAAKHARKEGNVTGGHPVSRGTAKLRWLVERRFLEPVGKVIDLGCGRGGWCYYMATQKRVQEVRGYTKGGPGHEEPQLVQSYGWNIVTMKSGVDVFYRPSECCDTLLCDIGESSSSAEVEEHRTIRVLEMVEDWLHRGPREFCVKVLCPYMPKVIEKMELLQRRYGGGLVRNPLSRNSTHEMYWVSRASGNVVHSVNMTSQVLLGRMEKRTWKGPQYEEDVNLGSGTRAVGKPLLNSDTSKIKNRIERLRREYSSTWHHDENHPYRTWNYHGSYDVKPTGSASSLVNGVVRLLSKPWDTITNVTTMAMTDTTPFGQQRVFKEKVDTKAPEPPEGVKYVLNETTNWLWAFLAREKRPRMCSREEFIRKVNSNAALGAMFEEQNQWRSAREAVEDPKFWEMVDEEREAHLRGECHTCIYNMMGKREKKPGEFGKAKGSRAIWFMWLGARFLEFEALGFLNEDHWLGRKNSGGGVEGLGLQKLGYILREVGTRPGGKIYADDTAGWDTRITRADLENEAKVLELLDGEHRRLARAIIELTYRHKVVKVMRPAADGRTVMDVISREDQRGSGQVVTYALNTFTNLAVQLVRMMEGEGVIGPDDVEKLTKGKGPKVRTWLFENGEERLSRMAVSGDDCVVKPLDDRFATSLHFLNAMSKVRKDIQEWKPSTGWYDWQQVPFCSNHFTELIMKDGRTLVVPCRGQDELVGRARISPGAGWNVRDTACLAKSYAQMWLLLYFHRRDLRLMANAICSAVPVNWVPTGRTTWSIHAGGEWMTTEDMLEVWNRVWIEENEWMEDKTPVEKWSDVPYSGKREDIWCGSLIGTRARATWAENIQVAINQVRAIIGDEKYVDYMSSLKRYEDTTLVEDTVL

>ANW68569.1 genome polyprotein [West Nile virus]

MSKKPGGPGKSRAVNMQKRGMPRVLSLIGLKRAMLSLIDGKGPIRFVLALLAFFRFTAIAPTRAVLDRWRGVNKQTAMKHLLSFKKELGTLTSAINRRSSKQKKRGGKTGIAVMIGLIASVGAVTLSNFQGKVMMTVNATDVTDVITIPTAAGKNLCIVRAMDVGYMCDDTITYECPVLSAGNDPEDIDCWCTKSAVYVRYGRCTKTRHSRRSRRSLTVQTHGESTLANKKGAWMDSTKATRYLVKTESWILRNPGYALVAAVIGWMLGSNTMQRVVFVVLLLLVAPAYSFNCLGMSNRDFLEGVSGATWVDLVLEGDSCVTIMSKDKPTIDVKMMNMEAANLAEVRSYCYLATVSDLSTKAACPTMGEAHNDKRADPAFVCRQGVVDRGWGNGCGLFGKGSIDTCAKFACSTKAIGRTILKENIKYEVAIFVHGPTTVESHGNYSTQAGATQAGRFSITPAAPSYTLKLGEYGEVTVDCEPRSGIDTNAYYVMTVGTKTFLVHREWFMDLNLPWSSAGSTVWRNRETLMEFEEPHATKQSVIALGSQEGALHQALAGAIPVEFSSNTVKLTSGHLKCRVKMEKLQLKGTTYGVCSKAFKFLGTPADTGHGTVVLELQYTGTDGPCKVPISSVASLNDLTPVGRLVTVNPFVSVATANAKVLIELEPPFGDSYIVVGRGEQQINHHWHKSGSSIGKAFTTTLKGAQRLAALGDTAWDFGSVGGVFTSVGKAVHQVFGGAFRSLFGGMSWITQGLLGALLLWMGINARDRSIALTFLAVGGVLLFLSVNVHADTGCAIDISRQELRCGSGVFIHNDVEAWMDRYKYYPETPQGLAKIIQKAHKEGVCGLRSVSRLEHQMWEAVKDELNTLLKENGVDLSVVVEKQEGMYKSAPKRLTATTEKLEIGWKAWGKSILFAPELANNTFVVDGPETKECPTQNRAWNSLEVEDFGFGLTSTRMFLKVRESNTTECDSKIIGTAVKNNLAIHSDLSYWIESRLNDTWKLERAVLGEVKSCTWPETHTLWGDGILESDLIIPVTLAGPRSNHNRRPGYKTQNQGPWDEGRVEIDFDYCPGTTVTLSESCGHRGPATRTTTESGKLITDWCCRSCTLPPLRYQTDSGCWYGMEIRPQRHDEKTLVQSQVNAYNADMIDPFQLGLLVVFLATQEVLRKRWTAKISMPAILIALLVLVFGGITYTDVLRYVILVGAAFAESNSGGDVVHLALMATFKIQPVFMVASFLKARWTNQENILLMLAAVFFQMAYHDARQILLWEIPDVLNSLAVAWMILRAITFTTTSNVVVPLLALLTPGLRCLNLDVYRILLLMVGIGSLIREKRSAAAKKKGASLLCLALASTGLFNPMILAAGLIACDPNRKRGWPATEVMTAVGLMFAIVGGLAELDIDSMAIPMTIAGLMFAAFVISGKSTDMWIERTADISWESDAEITGSSERVDVRLDDDGNFQLMNDPGAPWKIWMLRMVCLAISAYTPWAILPSVVGFWITLQYTKRGGVLWDTPSPKEYKKGDTTTGVYRIMTRGLLGSYQAGAGVMVEGVFHTLWHTTKGAALMSGEGRLDPYWGSVKEDRLCYGGPWKLQHKWNGQDEVQMIVVEPGKNVKNVQTKPGVFKTPEGEIGAVTLDFPTGTSGSPIVDKNGDVIGLYGNGVIMPNGSYISAIVQGERMDEPIPAGFEPEMLRKKQITVLDLHPGAGKTRRILPQIIKEAINRRLRTAVLAPTRVVAAEMAEALRGLPIRYQTSAVPREHNGNEIVDVMCHATLTHRLMSPHRVPNYNLFVMDEAHFTDPASIAARGYISTKVELGEAAAIFMTATPPGTSDPFPESNSPISDLQTEIPDRAWNSGYEWITEYTGKTVWFVPSVKMGNEIALCLQRAGKKVVQLNRKSYETEYPKCKNDDWDFVITTDISEMGANFKASRVIDSRKSVKPTIITEGEGRVILGEPSAVTAASAAQRRGRIGRNPSQVGDEYCYGGHTNEDDSNFAHWTEARIMLDNINMPNGLIAQFYQPEREKVYTMDGEYRLRGEERKNFLELLRTADLPVWLAYKVAAAGVSYHDRRWCFDGPRTNTILEDNNEVEVITKLGERKILRPRWIDARVYSDHQALKAFKDFASGKRSQIGLIEVLGKMPEHFMGKTWEALDTMYVVATAEKGGRAHRMALEELPDALQTIALIALLSVMTMGVFFLLMQRKGIGKIGLGGAVLGVATFFCWMAEVPGTKIAGMLLLSLLLMIVLIPEPEKQRSQTDNQLAVFLICVMTLVSAVAANEMGWLDKTKSDISSLFGQRIEVKENFSMGEFLLDLRPATAWSLYAVTTAVLTPLLKHLITSDYINTSLTSINVQASALFTLARGFPFVDVGVSALLLAAGCWGQVTLTVTVTAATLLFCHYAYMVPGWQAEAMRSAQRRTAAGIMKNAVVDGIVATDVPELERTTPIMQKKVGQIMLILVSLAAVVVNPSVKTVREAGILITAAAVTLWENGASSVWNATTAIGLCHIMRGGWLSCLSITWTLIKNMEKPGLKRGGAKGRTLGEVWKERLNQMTKEEFTRYRKEAIIEVDRSAAKHARKEGNVTGGHPVSRGTAKLRWLVERRFLEPVGKVIDLGCGRGGWCYYMATQKRVQEVRGYTKGGPGHEEPQLVQSYGWNIVTMKSGVDVFYRPSECCDTLLCDIGESSSSAEVEEHRTIRVLEMVEDWLHRGPREFCVKVLCPYMPKVIEKMELLQRRYGGGLVRNPLSRNSTHEMYWVSRASGNVVHSVNMTSQVLLGRMEKRTWKGPQYEEDVNLGSGTRAVGKPLLNSDTSKIKNRIERLRREYSSTWHHDENHPYRTWNYHGSYDVKPTGSASSLVNGVVRLLSKPWDTITNVTTMAMTDTTPFGQQRVFKEKVDTKAPEPPEGVKYVLNETTNWLWAFLAREKRPRMCSREEFIRKVNSNAALGAMFEEQNQWRSAREAVEDPKFWEMVDEEREAHLRGECHTCIYNMMGKREKKPGEFGKAKGSRAIWFMWLGARFLEFEALGFLNEDHWLGRKNSGGGVEGLGLQKLGYILREVGTRPGGKIYADDTAGWDTRITRADLENEAKVLELLDGEHRRLARAIIELTYRHKVVKVMRPAADGRTVMDVISREDQRGSGQVVTYALNTFTNLAVQLVRMMEGEGVIGPDDVEKLTKGKGPKVRTWLFENGEERLSRMAVSGDDCVVKPLDDRFATSLHFLNAMSKVRKDIQEWKPSTGWYDWQQVPFCSNHFTELIMKDGRTLVVPCRGQDELVGRARISPGAGWNVRDTACLAKSYAQMWLLLYFHRRDLRLMANAICSAVPVNWVPTGRTTWSIHAGGEWMTTEDMLEVWNRVWIEENEWMEDKTPVEKWSDVPYSGKREDIWCGSLIGTRARATWAENIQVAINQVRAIIGDEKYVDYMSSLKRYEDTTLVEDTVL

>AHW48475.1 polyprotein [West Nile virus]

MSKKPGGPGKSRAVNMLKRGMPRVLSLIGLKRAMLSLIDGKGPIRFVLALLAFFRFTAIAPTRAVLDRWRGVNKQTAMKHLLSFKKELGTLTSAINRRSSKQKKRGGKTGIAVMIGLIASVGAVTLSNFQGKVMMTVNATDVTDVITIPTAAGKNLCIVRAMDVGYMCDDTITYECPVLSAGNDPEDIDCWCTKSAVYVRYGRCTKTRHSRRSRRSLTVQTHGESTLANKKGAWMDSTKATRYLVKTESWILRNPGYALVAAVIGWMLGSNTMQRVVFVVLLLLVAPAYSFNCLGMSNRDFLEGVSGATWVDLVLEGDSCVTIMSKDKPTIDVKMMNMEAANLAEVRSYCYLATVSDLSTRAACPTMGEAHNDKRADPAFVCRQGVVDRGWGNGCGLFGKGSIDTCAKFACSTKAIGRTILKENIKYEVAIFVHGPTTVESHGNYSTQAGATQAGRFSITPAAPSYTLKLGEYGEVTVDCEPRSGIDTNAYYVMTVGTKTFLVHREWFMDLNLPWSSAGSTVWRNRETLMEFEEPHATKQSVIALGSQEGALHQALAGAIPVEFSSNTVKLTSGHLKCRVKMEKLQLKGTTYGVCSKAFKFLGTPADTGHGTVVLELQYTGTDGPCKVPISSVASLNDLTPVGRLVTVNPFVSVATANAKVLIELEPPFGDSYIVVGRGEQQINHHWHKSGSSIGKAFTTTLKGAQRLAALGDTAWDFGSVGGVFTSVGKAVHQVFGGAFRSLFGGMSWITQGLLGALLLWMGINARDRSIALTFLAVGGVLLFLSVNVHADTGCAIDISRQELRCGSGVFIHNDVEAWMDRYKYYPETPQGLAKIIQKAHKEGVCGLRSVSRLEHQMWEAVKDELNTLLKENGVDLSVVVEKQEGMYKSAPKRLTATTEKLEIGWKAWGKSILFAPELANNTFVVDGPETKECPTQNRAWNSLEVEDFGFGLTSTRMFLKVRESNTTECDSKIIGTAVKNNLAIHSDLSYWIESRLNDTWKLERAVLGEVKSCTWPETHTLWGDGILESDLIIPVTLAGPRSNHNRRPGYKTQNQGPWDEGRVEIDFDYCPGTTVTLSESCGHRGPATRTTTESGKLITDWCCRSCTLPPLRYQTDSGCWYGMEIRPQRHDEKTLVQSQVNAYNADMIDPFQLGLLVVFLATQEVLRKRWTAKISMPAILIALLVLVFGGITYTDVLRYVILVGAAFAESNSGGDVVHLALMATFKIQPVFMVASFLKARWTNQENILLMLAAVFFQMAYHDARQILLWEIPDVLNSLAVAWMILRAITFTTTSNVVVPLLALLTPGLRCLNLDVYRILLLMVGIGSLIREKRSAAAKKKGASLLCLALASTGLFNPMILAAGLIACDPNRKRGWPATEVMTAVGLMFAIVGGLAELDIDSMAIPMTIAGLMFAAFVISGKSTDMWIERTADISWESDAEITGSSERVDVRLDDDGNFQLMNDPGAPWKIWMLRMVCLAISAYTPWAILPSVVGFWITLQYTKRGGVLWDTPSPKEYKKGDTTTGVYRIMTRGLLGSYQAGAGVMVEGVFHTLWHTTKGAALMSGEGRLDPYWGSVKEDRLCYGGPWKLQHKWNGQDEVQMIVVEPGKNVKNVQTKPGVFKTPEGEIGAVTLDFPTGTSGSPIVDKNGDVIGLYGNGVIMPNGSYISAIVQGERMDEPIPAGFEPEMLRKKQITVLDLHPGAGKTRRILPQIIKEAINRRLRTAVLAPTRVVAAEMAEALRGLPIRYQTSAVPREHNGNEIVDVMCHATLTHRLMSPHRVPNYNLFVMDEAHFTDPASIAARGYISTKVELGEAAAIFMTATPPGTSDPFPESNSPISDLQTEIPDRAWNSGYEWITEYTGKTVWFVPSVKMGNEIALCLQRAGKKVVQLNRKSYETEYPKCKNDDWDFVITTDISEMGANFKASRVIDSRKSVKPTIITEGEGRVILGEPSAVTAASAAQRRGRIGRNPSQVGDEYCYGGHTNEDDSNFAHWTEARIMLDNINMPNGLIAQFYQPEREKVYTMDGEYRLRGEERKNFLELLRTADLPVWLAYKVAAAGVSYHDRRWCFDGPRTNTILEDNNEVEVITKLGERKILRPRWIDARVYSDHQALKAFKDFASGKRSQIGLIEVLGKMPEHFMGKTWEALDTMYVVATAEKGGRAHRMALEELPDALQTIALIALLSVMTMGVFFLLMQRKGIGKIGLGGAVLGVATFFCWMAEVPGTKIAGMLLLSLLLMIVLIPEPEKQRSQTDNQLAVFLICVMTLVSAVAANEMGWLDKTKSDISSLFGQRIEVKENFSMGEFLLDLRPATAWSLYAVTTAVLTPLLKHLITSDYINTSLTSINVQASALFTLARGFPFVDVGVSALLLAAGCWGQVTLTVTVTAATLLFCHYAYMVPGWQAEAMRSAQRRTAAGIMKNAVVDGIVATDVPELERTTPIMQKKVGQIMLILVSLAAVVVNPSVKTVREAGILITAAAVTLWENGASSVWNATTAIGLCHIMRGGWLSCLSITWTLIKNMEKPGLKRGGAKGRTLGEVWKERLNQMTKEEFTRYRKEAIIEVDRSAAKHARKEGNVTGGHPVSRGTAKLRWLVERRFLEPVGKVIDLGCGRGGWCYYMATQKRVQEVRGYTKGGPGHEEPQLVQSYGWNIVTMKSGVDVFYRPSECCDTLLCDIGESSSSAEVEEHRTIRVLEMVEDWLHRGPREFCVKVLCPYMPKVIEKMELLQRRYGGGLVRNPLSRNSTHEMYWVSRASGNVVHSVNMTSQVLLGRMEKRTWKGPQYEEDVNLGSGTRAVGKPLLNSDTSKIKNRIERLRREYSSTWHHDENHPYRTWNYHGSYDVKPTGSASSLVNGVVRLLSKPWDTITNVTTMAMTDTTPFGQQRVFKEKVDTKAPEPPEGVKYVLNETTNWLWAFLAREKRPRMCSREEFIRKVNSNAALGAMFEEQNQWRSAREAVEDPKFWEMVDEEREAHLRGECHTCIYNMMGKREKKPGEFGKAKGSRAIWFMWLGARFLEFEALGFLNEDHWLGRKNSGGGVEGLGLQKLGYILREVGTRPGGKIYADDTAGWDTRITRADLENEAKVLELLDGEHRRLARAIIELTYRHKVVKVMRPAADGRTVMDVISREDQRGSGQVVTYALNTFTNLAVQLVRMMEGEGVIGPDDVEKLTKGKGPKVRTWLFENGEERLSRMAVSGDDCVVKPLDDRFATSLHFLNAMSKVRKDIQEWKPSTGWYDWQQVPFCSNHFTELIMKDGRTLVVPCRGQDELVGRARISPGAGWNVRDTACLAKSYAQMWLLLYFHRRDLRLMANAICSAVPVNWVPTGRTTWSIHAGGEWMTTEDMLEVWNRVWIEENEWMEDKTPVEKWSDVPYSGKREDIWCGSLIGTRARATWAENIQVAINQVRAIIGDEKYVDYMSSLKRYEDTTLVEDTVL

>AHW48754.1 polyprotein [West Nile virus]

MSKKPGGPGKSRAVNMLKRGMPRVLSLIGLKRAMLSLIDGRGPIRFVLALLAFFRFTAIAPTRAVLDRWRGVNKQTAMKHLLSFKKELGTLTSAINRRSSKQKKRGGKTGIAVMIGLIASVGAVTLSNFQGKVMMTVNATDVTDVITIPTAAGKNLCIVRAMDVGYMCDDTITYECPVLSAGNDPEDIDCWCTKSAVYVRYGRCTKTRHSRRSRRSLTVQTHGESTLANKKGAWMDSTKATRYLVKTESWILRNPGYALVAAVIGWMLGSNTMQRVVFVVLLLLVAPAYSFNCLGMSNRDFLEGVSGATWVDLVLEGDSCVTIMSKDKPTIDVKMMNMEAANLAEVRSYCYLATVSDLSTKAACPTMGEAHNDKRADPAFVCRQGVVDRGWGNGCGLFGKGSIDTCAKFACSTKAIGRTILKENIKYEVAIFVHGPTTVESHGNYSTQAGATQAGRFSITPAAPSYTLKLGEYGEVTVDCEPRSGIDTNAYYVMTVGTKTFLVHREWFMDLNLPWSSAGSTVWRNRETLMEFEEPHATKQSVIALGSQEGALHQALAGAIPVEFSSNTVKLTSGHLKCRVKMEKLQLKGTTYGVCSKAFKFLGTPADTGHGTVVLELQYTGTDGPCKVPISSVASLNDLTPVGRLVTVNPFVSVATANAKVLIELEPPFGDSYIVVGRGEQQINHHWHKSGSSIGKAFTTTLKGAQRLAALGDTAWDFGSVGGVFTSVGKAVHQVFGGAFRSLFGGMSWITQGLLGALLLWMGINARDRSIALTFLAVGGVLLFLSVNVHADTGCAIDISRQELRCGSGVFIHNDVEAWMDRYKYYPETPQGLAKIIQKAHKEGVCGLRSVSRLEHQMWEAVKDELNTLLKENGVDLSVVVEKQEGMYKSAPKRLTATTEKLEIGWKAWGKSILFAPELANNTFVVDGPETKECPTQNRAWNSLEVEDFGFGLTSTRMFLKVRESNTTECDSKIIGTAVKNNLAIHSDLSYWIESRLNDTWKLERAVLGEVKSCTWPETHTLWGDGILESDLIIPVTLAGPRSNHNRRPGYKTQNQGPWDEGRVEIDFDYCPGTTVTLSESCGHRGPATRTTTESGKLITDWCCRSCTLPPLRYQTDSGCWYGMEIRPQRHDEKTLVQSQVNAYNADMIDPFQLGLLVVFLATQEVLRKRWTAKISMPAILIALLVLVFGGITYTDVLRYVILVGAAFAESNSGGDVVHLALMATFKIQPVFMVASFLKARWTNQENILLMLAAVFFQMAYHDARQILLWEIPDVLNSLAVAWMILRAITFTTTSNVVVPLLALLTPGLRCLNLDVYRILLLMVGIGSLIREKRSAAAKKKGASLLCLALASTGLFNPMILAAGLIACDPNRKRGWPATEVMTAVGLMFAIVGGLAELDIDSMAIPMTIAGLMFAAFVISGKSTDMWIERTADISWESDAEITGSSERVDVRLDDDGNFQLMNDPGAPWKIWMLRMVCLAISAYTPWAILPSVVGFWITLQYTKRGGVLWDTPSPKEYKKGDTTTGVYRIMTRGLLGSYQAGAGVMVEGVFHTLWHTTKGAALMSGEGRLDPYWGSVKEDRLCYGGPWKLQHKWNGQDEVQMIVVEPGKNVKNVQTKPGVFKTPEGEIGAVTLDFPTGTSGSPIVDKNGDVIGLYGNGVIMPNGSYISAIVQGERMDEPIPAGFEPEMLRKKQITVLDLHPGAGKTRRILPQIIKEAINRRLRTAVLAPTRVVAAEMAEALRGLPIRYQTSAVPREHNGNEIVDVMCHATLTHRLMSPHRVPNYNLFVMDEAHFTDPASIAARGYISTKVELGEAAAIFMTATPPGTSDPFPESNSPISDLQTEIPDRAWNSGYEWITEYTGKTVWFVPSVKMGNEIALCLQRAGKKVVQLNRKSYETEYPKCKNDDWDFVITTDISEMGANFKASRVIDSRKSVKPTIITEGEGRVILGEPSAVTAASAAQRRGRIGRNPSQVGDEYCYGGHTNEDDSNFAHWTEARIMLDNINMPNGLIAQFYQPEREKVYTMDGEYRLRGEERKNFLELLRTADLPVWLAYKVAAAGVSYHDRRWCFDGPRTNTILEDNNEVEVITKLGERKILRPRWIDARVYSDHQALKAFKDFASGKRSQIGLIEVLGKMPEHFMGKTWEALDTMYVVATAEKGGRAHRMALEELPDALQTIALIALLSVMTMGVFFLLMQRKGIGKIGLGGAVLGVATFFCWMAEVPGTKIAGMLLLSLLLMIVLIPEPEKQRSQTDNQLAVFLICVMTLVSAVAANEMGWLDKTKSDISSLFGQRIEVKENFSMGEFLLDLRPATAWSLYAVTTAVLTPLLKHLITSDYINTSLTSINVQASALFTLARGFPFVDVGVSALLLAAGCWGQVTLTVTVTAATLLFCHYAYMVPGWQAEAMRSAQRRTAAGIMKNAVVDGIVATDVPELERTTPIMQKKVGQIMLILVSLAAVVVNPSVKTVREAGILITAAAVTLWENGASSVWNATTAIGLCHIMRGGWLSCLSITWTLIKNMEKPGLKRGGAKGRTLGEVWKERLNQMTKEEFTRYRKEAIIEVDRSAAKHARKEGNVTGGHPVSRGTAKLRWLVERRFLEPVGKVIDLGCGRGGWCYYMATQKRVQEVRGYTKGGPGHEEPQLVQSYGWNIVTMKSGVDVFYRPSECCDTLLCDIGESSSSAEVEEHRTIRVLEMVEDWLHRGPREFCVKVLCPYMPKVIEKMELLQRRYGGGLVRNPLSRNSTHEMYWVSRASGNVVHSVNMTSQVLLGRMEKRTWKGPQYEEDVNLGSGTRAVGKPLLNSDTSKIKNRIERLRREYSSTWHHDENHPYRTWNYHGSYDVKPTGSASSLVNGVVRLLSKPWDTITNVTTMAMTDTTPFGQQRVFKEKVDTKAPEPPEGVKYVLNETTNWLWAFLAREKRPRMCSREEFIRKVNSNAALGAMFEEQNQWRSAREAVEDPKFWEMVDEEREAHLRGECHTCIYNMMGKREKKPGEFGKAKGSRAIWFMWLGARFLEFEALGFLNEDHWLGRKNSGGGVEGLGLQKLGYILREVGTRPGGKIYADDTAGWDTRITRADLENEAKVLELLDGEHRRLARAIIELTYRHKVVKVMRPAADGRTVMDVISREDQRGSGQVVTYALNTFTNLAVQLVRMMEGEGVIGPDDVEKLTKGKGPKVRTWLFENGEERLSRMAVSGDDCVVKPLDDRFATSLHFLNAMSKVRKDIQEWKPSTGWYDWQQVPFCSNHFTELIMKDGRTLVVPCRGQDELVGRARISPGAGWNVRDTACLAKSYAQMWLLLYFHRRDLRLMANAICSAVPVNWVPTGRTTWSIHAGGEWMTTEDMLEVWNRVWIEENEWMEDKTPVEKWSDVPYSGKREDIWCGSLIGTRARATWAENIQVAINQVRAIIGDEKYVDYMSSLKRYEDTTLVEDTVL

>AAM81750.1 polyprotein precursor [West Nile virus]

MSKKPGGPGKSRAVNMLKRGMPRVLSLIGLKRAMLSLIDGKGPIRFVLALLAFFRFTAIAPTRAVLDRWRGVNKQTAMKHLLSFKKELGTLTSAINRRSSKEKKRGGKTGIAVMIGLIASVGAVTLSNFQGKVMMTVNATDVTDVITIPTAAGKNLCIVRAMDVGYMCDDTITYECPVLSAGNDPEDIDCWCTKSAVYVRYGRCTKTRHSRRSRRSLTVQTHGESTLANKKGAWMDSTKATRYLVKTESWILRNPGYALVAAVIGWMLGSNTMQRVVFVVLLLLVAPAYSFNCLGMSNRDFLEGVSGATWVDLVLEGDSCVTIMSKDKPTIDVKMMNMEAANLAEVRSYCYLATVSDLSTKAACPTMGEAHNDKRADPAFVCRQGVVDRGWGNGCGLFGKGSIDTCAKFACSTKAIGRTILKENIKYEVAIFVHGPTTVESHGNYSTQVGATQAGRFSITPAAPSYTLKLGEYGEVTVDCEPRSGIDTNAYYVMTVGTKTFLVHREWFMDLNLPWSSAGSTVWRNRETLMEFEEPHATKQSVIALGSQEGALHQALAGAIPVEFSSNTVKLTSGHLKCRVKMEKLQLKGTTYGVCSKAFKFLGTPADTGHGTVVLELQYTGTDGPCKVPISSVASLNDLTPVGRLVTVNPFVSVATANAKVLIELEPPFGDSYIVVGRGEQQINHHWHKSGSSIGKAFTTTLKGAQRLAALGDTAWDFGSVGGVFTSVGKAVHQVFGGAFRSLFGGMSWITQGLLGALLLWMGINARDRSIALTFLAVGGVLLFLSVNVHADTGCAIDISRQELRCGSGVFIHNDVEAWMDRYKYYPETPQGLAKIIQKAHKEGVCGLRSVSRLEHQMWEAVKDELNTLLKENGVDLSVVVEKQEGMYKSAPKRLTATTEKLEIGWKAWGKSILFAPELANNTFVVDGPETKECPTQNRAWNSLEVEDFGFGLTSTRMFLKVRESNTTECDSKIIGTAVKNNLAIHSDLSYWIESRLNDTWKLERAVLGEVKSCTWPETHTLWGDGILESDLIIPVTLAGPRSNHNRRPGYKTQNQGPWDEGRVEIDFDYCPGTTVTLSESCGHRGPATRTTTESGKLITDWCCRSCTLPPLRYQTDSGCWYGMEIRPQRHDEKTLVQSQVNAYNADMIDPFQLGLLVVFLATQEVLRKRWTAKISMPAILIALLVLVFGGITYTDVLRYVILVGAAFAESNSGGDVVHLALMATFKIQPVFMVASFLKARWTNQENILLMLAAVFFQMAYHDARQILLWEIPDVLNSLAVAWMILRAITFTTTSNVVVPLLALLTPGLRCLNLDVYRILLLMVGIGSLIREKRSAAAKKKGASLLCLALASTGLFNPMILAAGLIACDPNRKRGWPATEVMTAVGLMFAIVGGLAELDIDSMAIPMTIAGLMFAAFVISGKSTDMWIERTADISWESDAEITGSSERVDVRLDDDGNFQLMNDPGAPWKIWMLRMVCLAISAYTPWAILPSVVGFWITLQYTKRGGVLWDTPSPKEYKKGDTTTGVYRIMTRGLLGSYQAGAGVMVEGVFHTLWHTTKGAALMSGEGRLDPYWGSVKEDRLCYGGPWKLQHKWNGQDEVQMIVVEPGKNVKNVQTKPGVFKTPEGEIGAVTLDFPTGTSGSPIVDKNGDVIGLYGNGVIMPNGSYISAIVQGERMDEPIPAGFEPEMLRKKQITVLDLHPGAGKTRRILPQIIKEAINRRLRTAVLAPTRVVAAEMAEALRGLPIRYQTSAVPREHNGNEIVDVMCHATLTHRLMSPHRVPNYNLFVMDEAHFTDPASIAARGYISTKVELGEAAAIFMTATPPGTSDPFPESNSPISDLQTEIPDRAWNSGYEWITEYTGKTVWFVPSVKMGNEIALCLQRAGKKVVQLNRKSYETEYPKCKNDDWDFVITTDISEMGANFKASRVIDSRKSVKPTIITEGEGRVILGEPSAVTAASAAQRRGRIGRNPSQVGDEYCYGGHTNEDDSNFAHWTEARIMLDNINMPNGLIAQFYQPEREKVYTMDGEYRLRGEERKNFLELLRTADLPVWLAYKVAAAGVSYHDRRWCFDGPRTNTILEDNNEVEVITKLGERKILRPRWIDARVYSDHQALKAFKDFASGKRSQIGLIEVLGKMPEHFMGKTWEALDTMYVVATAEKGGRAHRMALEELPDALQTIALIALLSVMTMGVFFLLMQRKGIGKIGLGGAVLGVATFFCWMAEVPGTKIAGMLLLSLLLMIVLIPEPEKQRSQTDNQLAVFLICVMTLVSAVAANEMGWLDKTKSDISSLFGQRIEVKENFSMGEFLLDLRPATAWSLYAVTTAVLTPLLKHLITSDYINTSLTSINVQASALFTLARGFPFVDVGVSALLLAAGCWGQVTLTVTVTAATLLFCHYAYMVPGWQAEAMRSAQRRTAAGIMKNAVVDGIVATDVPELERTTPIMQKKVGQIMLILVSLAAVVVNPSVKTVREAGILITAAAVTLWENGASSVWNATTAIGLCHIMRGGWLSCLSITWTLIKNMEKPGLKRGGAKGRTLGEVWKERLNQMTKEEFTRYRKEAIIEVDRSAAKHARKEGNVTGGHPVSRGTAKLRWLVERRFLEPVGKVIDLGCGRGGWCYYMATQKRVQEVRGYTKGGPGHEEPQLVQSYGWNIVTMKSGVDVFYRPSECCDTLLCDIGESSSSAEVEEHRTIRVLEMVEDWLHRGPREFCVKVLCPYMPKVIEKMELLQRRYGGGLVRNPLSRNSTHEMYWVSRASGNVVHSVNMTSQVLLGRMEKRTWKGPQYEEDVNLGSGTRAVGKPLLNSDTSKIKNRIERLRREYSSTWHHDENHPYRTWNYHGSYDVKPTGSASSLVNGVVRLLSKPWDTITNVTTMAMTDTTPFGQQRVFKEKVDTKAPEPPEGVKYVLNETTNWLWAFLAREKRPRMCSREEFIRKVNSNAALGAMFEEQNQWRSAREAVEDPKFWEMVDEEREAHLRGECHTCIYNMMGKREKKPGEFGKAKGSRAIWFMWLGARFLEFEALGFLNEDHWLGRKNSGGGVEGLGLQKLGYILREVGTRPGGKIYADDTAGWDTRITRADLENEAKVLELLDGEHRRLARAIIELTYRHKVVKVMRPAADGRTVMDVISREDQRGSGQVVTYALNTFTNLAVQLVRMMEGEGVIGPDDVEKLTKGKGPKVRTWLFENGEERLSRMAVSGDDCVVKPLDDRFATSLHFLNAMSKVRKDIQEWKPSTGWYDWQQVPFCSNHFTELIMKDGRTLVVPCRGQDELVGRARISPGAGWNVRDTACLAKSYAQMWLLLYFHRRDLRLMANAICSAVPVNWVPTGRTTWSIHAGGEWMTTEDMLEVWNRVWIEENEWMEDKTPVEKWSDVPYSGKREDIWCGSLIGTRARATWAENIQVAINQVRAIIGDEKYVDYMSSLKRYEDTTLVEDTVL

>AYV87282.1 polyprotein [West Nile virus]

MSKKPGGPGKSRAVNMLKRGMPRVLSLIGLKRAMLSLIDGKGPIRFVLALLAFFRFTAIAPTRAVLDRWRGVNKQTAMKHLLSFKKELGTLTSAINRRSSKQKKRGGKTGIAVMIGLIASVGAVTLSNFQGKVMMTVNATDVTDVITIPTAAGKNLCIVRAMDVGYMCDDTITYECPVLSAGNDPEDIDCWCTKSAVYVRYGRCTKTRHSRRSRRSLTVQTHGESTLANKKGAWMDSTKATRYLVKTESWILRNPGYALVAAVIGWMLGSNTMQRVVFVVLLLLVAPAYSFNCLGMSNRDFLEGVSGATWVDLVLEGDSCVTIMSKDKPTIDVKMMNMEAANLAEVRSYCYLATVSDLSTKAACPTMGEAHNDKRADPAFVCRQGVVDRGWGNGCGLFGKGSIDTCAKFACSTKAIGRTILKENIKYEVAIFVHGPTTVESHGNYSTQVGATQAGRFSITPAAPSYTLKLGEYGEVTMDCEPRSGIDTNAYYVMTVGTKTFLVHREWFMDLNLPWSSAGSTVWRNRETLMEFEEPHATKQSVIALGSQEGALHQALAGAIPVEFSSNTVKLTSGHLKCRVKMEKLQLKGTTYGVCSKAFKFLGTPADTGHGTVVLELQYTGTDGPCKVPISSVASLNDLTPVGRLVTVNPFVSVATANAKVLIELEPPFGDSYIVVGRGEQQINHHWHKSGSSIGKAFTTTLKGAQRLAALGDTAWDFGSVGGVFTSVGKAVHQVFGGAFRSLFGGMSWITQGLLGALLLWMGINARDRSIALTFLAVGGVLLFLSVNVHADTGCAIDISRQELRCGSGVFIHNDVEAWMDRYKYYPETPQGLAKIIQKAHKEGVCGLRSVSRLEHQMWEAVKDELNTLLKENGVDLSVVVEKQEGMYKSAPKRLTATTEKLEIGWKAWGKSILFAPELANNTFVVDGPETKECPTQNRAWNSLEVEDFGFGLTSTRMFLKVRESNTTECDSKIIGTAVKNNLAIHSDLSYWIESRLNDTWKLERAVLGEVKSCTWPETHTLWGDGILESDLIIPVTLAGPRSNHNRRPGYKTQNQGPWDEGRVEIDFDYCPGTTVTLSESCGHRGPATRTTTESGKLITDWCCRSCTLPPLRYQTDSGCWYGMEIRPQRHDEKTLVQSQVNAYNADMIDPFQLGLLVVFLATQEVLRKRWTAKISMPAILIALLVLVFGGITYTDVLRYVILVGAAFAESNSGGDVVHLALMATFKIQPVFMVASFLKARWTNQENILLMLAAVFFQMAYHDARQILLWEIPDVLNSLAVAWMILRAITFTTTSNVVVPLLALLTPGLRCLNLDVYRILLLMVGIGSLIREKRSAAAKKKGASLLCLALASTGLFNPMILAAGLIACDPNRKRGWPATEVMTAVGLMFAIVGGLAELDIDSMAIPMTIAGLMFAAFVISGKSTDMWIERTADISWESDAEITGSSERVDVRLDDDGNFQLMNDPGAPWKIWMLRMVCLAISAYTPWAILPSVVGFWITLQYTKRGGVLWDTPSPKEYKKGDTTTGVYRIMTRGLLGSYQAGAGVMVEGVFHTLWHTTKGAALMSGEGRLDPYWGSVKEDRLCYGGPWKLQHKWNGQDEVQMIVVEPGKNVKNVQTKPGVFKTPEGEIGAVTLDFPTGTSGSPIVDKNGDVIGLYGNGVIMPNGSYISAIVQGERMDEPIPAGFEPEMLRKKQITVLDLHPGAGKTRRILPQIIKEAINRRLRTAVLAPTRVVAAEMAEALRGLPIRYQTSAVPREHNGNEIVDVMCHATLTHRLMSPHRVPNYNLFVMDEAHFTDPASIAARGYISTKVELGEAAAIFMTATPPGTSDPFPESNSPISDLQTEIPDRAWNSGYEWITEYTGKTVWFVPSVKMGNEIALCLQRAGKKVVQLNRKSYETEYPKCKNDDWDFVITTDISEMGANFKASRVIDSRKSVKPTIITEGEGRVILGEPSAVTAASAAQRRGRIGRNPSQVGDEYCYGGHTNEDDSNFAHWTEARIMLDNINMPNGLIAQFYQPEREKVYTMDGEYRLRGEERKNFLELLRTADLPVWLAYKVAAAGVSYHDRRWCFDGPRTNTILEDNNEVEVITKLGERKILRPRWIDARVYSDHQALKAFKDFASGKRSQIGLIEVLGKMPEHFMGKTWEALDTMYVVATAEKGGRAHRMALEELPDALQTIALIALLSVMTMGVFFLLMQRKGIGKIGLGGAVLGVATFFCWMAEVPGTKIAGMLLLSLLLMIVLIPEPEKQRSQTDNQLAVFLICVMTLVSAVAANEMGWLDKTKSDISSLFGQRIEVKENFSMGEFLLDLRPATAWSLYAVTTAVLTPLLKHLITSDYINTSLTSINVQASALFTLARGFPFVDVGVSALLLAAGCWGQVTLTVTVTAATLLFCHYAYMVPGWQAEAMRSAQRRTAAGIMKNAVVDGIVATDVPELERTTPIMQKKVGQIMLILVSLAAVVVNPSVKTVREAGILITAAAVTLWENGASSVWNATTAIGLCHIMRGGWLSCLSITWTLIKNMEKPGLKRGGAKGRTLGEVWKERLNQMTKEEFTRYRKEAIIEVDRSAAKHARKEGNVTGGHPVSRGTAKLRWLVERRFLEPVGKVIDLGCGRGGWCYYMATQKRVQEVRGYTKGGPGHEEPQLVQSYGWNIVTMKSGVDVFYRPSECCDTLLCDIGESSSSAEVEEHRTIRVLEMVEDWLHRGPREFCVKVLCPYMPKVIEKMELLQRRYGGGLVRNPLSRNSTHEMYWVSRASGNVVHSVNMTSQVLLGRMEKRTWKGPQYEEDVNLGSGTRAVGKPLLNSDTSKIKNRIERLRREYSSTWHHDENHPYRTWNYHGSYDVKPTGSASSLVNGVVRLLSKPWDTITNVTTMAMTDTTPFGQQRVFKEKVDTKAPEPPEGVKYVLNETTNWLWAFLAREKRPRMCSREEFIRKVNSNAALGAMFEEQNQWRSAREAVEDPKFWEMVDEEREAHLRGECHTCIYNMMGKREKKPGEFGKAKGSRAIWFMWLGARFLEFEALGFLNEDHWLGRKNSGGGVEGLGLQKLGYILREVGTRPGGKIYADDTAGWDTRITRADLENEAKVLELLDGEHRRLARAIIELTYRHKVVKVMRPAADGRTVMDVISREDQRGSGQVVTYALNTFTNLAVQLVRMMEGEGVIGPDDVEKLTKGKGPKVRTWLFENGEERLSRMAVSGDDCVVKPLDDRFATSLHFLNAMSKVRKDIQEWKPSTGWYDWQQVPFCSNHFTELIMKDGRTLVVPCRGQDELVGRARISPGAGWNVRDTACLAKSYAQMWLLLYFHRRDLRLMANAICSAVPVNWVPTGRTTWSIHAGGEWMTTEDMLEVWNRVWIEENEWMEDKTPVEKWSDVPYSGKREDIWCGSLIGTRARATWAENIQVAINQVRAIIGDEKYVDYMSSLKRYEDTTLVEDTVL

>AYV87341.1 polyprotein [West Nile virus]

MSKKPGGPGKSRAVNMLKRGMPRVLSLIGLKRAMLSLIDGKGPIRFVLALLAFFRFTAIAPTRAVLDRWRGVNKQTAMKHLLSFKKELGTLTSAINRRSSKQKKRGGKTGIAVMIGLIASVGAVTLSNFQGKVMMTVNATDVTDVITIPTAAGKNLCIVRAMDVGYMCDDTITYECPVLSAGNDPEDIDCWCTKSAVYVRYGRCTKTRHSRRSRRSLTVQTHGESTLANKKGAWMDSTKATRYLVKTESWILRNPGYALVAAVIGWMLGSNTMQRVVFVVLLLLVAPAYSFNCLGMSNRDFLEGVSGATWVDLVLEGDSCVTIMSKDKPTIDVKMMNMEAANLAEVRSYCYLATVSDLSTKAACPTMGEAHNDKRADPAFVCRQGVVDRGWGNGCGLFGKGSIDTCAKFACSTKAIGRTILKENIKYEVAIFVHGPTTVESHGNYSTQAGATQAGRFSITPAAPSYTLRLGEYGEVTVDCEPRSGIDTNAYYVMTVGTKTFLVHREWFMDLNLPWSSAGSTVWRNRETLMEFEEPHATKQSVIALGSQEGALHQALAGAIPVEFSSNTVKLTSGHLKCRVKMEKLQLKGTTYGVCSKAFKFLGTPADTGHGTVVLELQYTGTDGPCKVPISSVASLNDLTPVGRLVTVNPFVSVATANAKVLIELEPPFGDSYIVVGRGEQQINHHWHKSGSSIGKAFTTTLKGAQRLAALGDTAWDFGSVGGVFTSVGKAVHQVFGGAFRSLFGGMSWITQGLLGALLLWMGINARDRSIALTFLAVGGVLLFLSVNVHADTGCAIDISRQELRCGSGVFIHNDVEAWMDRYKYYPETPQGLAKIIQKAHKEGVCGLRSVSRLEHQMWEAVKDELNTLLKENGVDLSVVVEKQEGMYKSAPKRLTATTEKLEIGWKAWGKSILFAPELANNTFVVDGPETKECPTQNRAWNSLEVEDFGFGLTSTRMFLKVRESNTTECDSKIIGTAVKNNLAIHSDLSYWIESRLNDTWKLERAVLGEVKSCTWPETHTLWGDGILESDLIIPVTLAGPRSNHNRRPGYKTQNQGPWDEGRVEIDFDYCPGTTVTLSESCGHRGPATRTTTESGKLITDWCCRSCTLPPLRYQTDSGCWYGMEIRPQRHDEKTLVQSQVNAYNADMIDPFQLGLLVVFLATQEVLRKRWTAKISMPAILIALLVLVFGGITYTDVLRYVILVGAAFAESNSGGDVVHLALMATFKIQPVFMVASFLKARWTNQENILLMLAAVFFQMAYHDARQILLWEIPDVLNSLAVAWMILRAITFTTTSNVVVPLLALLTPGLRCLNLDVYRILLLMVGIGSLIREKRSAAAKKKGASLLCLALASTGLFNPMILAAGLIACDPNRKRGWPATEVMTAVGLMFAIVGGLAELDIDSMAIPMTIAGLMFAAFVISGKSTDMWIERTADISWESDAEITGSSERVDVRLDDDGNFQLMNDPGAPWKIWMLRMVCLAISAYTPWAILPSVVGFWITLQYTKRGGVLWDTPSPKEYKKGDTTTGVYRIMTRGLLGSYQAGAGVMVEGVFHTLWHTTKGAALMSGEGRLDPYWGSVKEDRLCYGGPWKLQHKWNGQDEVQMIVVEPGKNVKNVQTKPGVFKTPEGEIGAVTLDFPTGTSGSPIVDKNGDVIGLYGNGVIMPNGSYISAIVQGERMDEPIPAGFEPEMLRKKQITVLDLHPGAGKTRRILPQIIKEAINRRLRTAVLAPTRVVAAEMAEALRGLPIRYQTSAVPREHNGNEIVDVMCHATLTHRLMSPHRVPNYNLFVMDEAHFTDPASIAARGYISTKVELGEAAAIFMTATPPGTSDPFPESNSPISDLQTEIPDRAWNSGYEWITEYTGKTVWFVPSVKMGNEIALCLQRAGKKVVQLNRKSYETEYPKCKNDDWDFVITTDISEMGANFKASRVIDSRKSVKPTIITEGEGRVILGEPSAVTAASAAQRRGRIGRNPSQVGDEYCYGGHTNEDDSNFAHWTEARIMLDNINMPNGLIAQFYQPEREKVYTMDGEYRLRGEERKNFLELLRTADLPVWLAYKVAAAGVSYHDRRWCFDGPRTNTILEDNNEVEVITKLGERKILRPRWIDARVYSDHQALKAFKDFASGKRSQIGLIEVLGKMPEHFMGKTWEALDTMYVVATAEKGGRAHRMALEELPDALQTIALIALLSVMTMGVFFLLMQRKGIGKIGLGGAVLGVATFFCWMAEVPGTKIAGMLLLSLLLMIVLIPEPEKQRSQTDNQLAVFLICVMTLVSAVAANEMGWLDKTKSDISSLFGQRIEVKENFSMGEFLLDLRPATAWSLYAVTTAVLTPLLKHLITSDYINTSLTSINVQASALFTLARGFPFVDVGVSALLLAAGCWGQVTLTVTVTAATLLFCHYAYMVPGWQAEAMRSAQRRTAAGIMKNAVVDGIVATDVPELERTTPIMQKKVGQIMLILVSLAAVVVNPSVKTVREAGILITAAAVTLWENGASSVWNATTAIGLCHIMRGGWLSCLSITWTLIKNMEKPGLKRGGAKGRTLGEVWKERLNQMTKEEFTRYRKEAIIEVDRSAAKHARKEGNVTGGHPVSRGTAKLRWLVERRFLEPVGKVIDLGCGRGGWCYYMATQKRVQEVRGYTKGGPGHEEPQLVQSYGWNIVTMKSGVDVFYRPSECCDTLLCDIGESSSSAEVEEHRTIRVLEMVEDWLHRGPREFCVKVLCPYMPKVIEKMELLQRRYGGGLVRNPLSRNSTHEMYWVSRASGNVVHSVNMTSQVLLGRMEKRTWKGPQYEEDVNLGSGTRAVGKPLLNSDTSKIKNRIERLRREYSSTWHHDENHPYRTWNYHGSYDVKPTGSASSLVNGVVRLLSKPWDTITNVTTMAMTDTTPFGQQRVFKEKVDTKAPEPPEGVKYVLNETTNWLWAFLAREKRPRMCSREEFIRKVNSNAALGAMFEEQNQWRSAREAVEDPKFWEMVDEEREAHLRGECHTCIYNMMGKREKKPGEFGKAKGSRAIWFMWLGARFLEFEALGFLNEDHWLGRKNSGGGVEGLGLQKLGYILREVGTRPGGKIYADDTAGWDTRITRADLENEAKVLELLDGEHRRLARAIIELTYRHKVVKVMRPAADGRTVMDVISREDQRGSGQVVTYALNTFTNLAVQLVRMMEGEGVIGPDDVEKLTKGKGPKVRTWLFENGEERLSRMAVSGDDCVVKPLDDRFATSLHFLNAMSKVRKDIQEWKPSTGWYDWQQVPFCSNHFTELIMKDGRTLVVPCRGQDELVGRARISPGAGWNVRDTACLAKSYAQMWLLLYFHRRDLRLMANAICSAVPVNWVPTGRTTWSIHAGGEWMTTEDMLEVWNRVWIEENEWMEDKTPVEKWSDVPYSGKREDIWCGSLIGTRARATWAENIQVAINQVRAIIGDEKYVDYMSSLKRYEDTTLVEDTVL

>ANW69004.1 genome polyprotein [West Nile virus]

MSKKPGGPGKSRAVNMLKRGMPRVLSLIGLKRAMLSLIDGKGPIRFVLALLAFFRFTAIAPTRAVLDRWRGVNKQTAMKHLLSFKKELGTLTSAINRRSSKQKKRGGKTGIAVMIGLIASVGAVTLSNFQGKVMMTVNATDVTDVITIPTAAGKNLCIVRAMDVGYMCDDTITYECPVLSAGNDPEDIDCWCTKSAVYVRYGRCTKTRHSRRSRRSLTVQTHGESTLANKKGAWMDSTKATRYLVKTESWILRNPGYALVAAVIGWMLGSNTMQRVVFVVLLLLVAPAYSFNCLGMSNRDFLEGVSGATWVDLVLEGDSCVTIMSKDKPTIDVKMMNMEAANLAEVRSYCYLATVSDLSTKAACPTMGEAHNDKRADPAFVCRQGVVDRGWGNGCGLFGKGSIDTCAKFACSTKAIGRTILKENIKYEVAIFVHGPTTVESHGNYSTQAGATQAGRFSITPAAPSYTLKLGEYGEVTVDCEPRSGIDTNAYYVMTVGTKTFLVHREWFMDLNLPWSSAGSTVWRNRETLMEFEEPHATKQSVIALGSQEGALHQALAGAIPVEFSSNTVKLTSGHLKCRVKMEKLQLKGTTYGVCSKAFKFLGTPADTGHGTVVLELQYTGTDGPCKVPISSVASLNDLTPVGRLVTVNPFVSVATANAKVLIELEPPFGDSYIVVGRGEQQINHHWHKSGSSIGKAFTTTLKGAQRLAALGDTAWDFGSVGGVFTSVGKAVHQVFGGAFRSLFGGMSWITQGLLGALLLWMGINARDRSIALTFLAVGGVLLFLSVNVHADTGCAIDISRQELRCGSGVFIHNDVEAWMDRYKYYPETPQGLAKIIQKAHKEGVCGLRSVSRLEHQMWEAVKDELNTLLKENGVDLSVVVEKQEGMYKSAPKRLTATTEKLEIGWKAWGKSILFAPELANNTFVVDGPETKECPTQNRAWNSLEVEDFGFGLTSTRMFLKVRESNTTECDSKIIGTAVKNNLAIHSDLSYWIESRLNDTWKLERAVLGEVKSCTWPETHTLWGDGILESDLIIPVTLAGPRSNHNRRPGYKTQNQGPWDEGRVEIDFDYCPGTTVTLSESCGHRGPATRTTTESGKLITDWCCRSCTLPPLRYQTDSGCWYGMEIRPQRHDEKTLVQSQVNAYNADMIDPFQLGLLVVFLATQEVLRKRWTAKISMPAILIALLVLVFGGITYTDVLRYVILVGAAFAESNSGGDVVHLALMATFKIQPVFMVASFLKARWTNQENILLMLAAVFFQMAYHDARQILLWEIPDVLNSLAVAWMILRAITFTTTSNVVVPLLALLTPGLRCLNLDVYRILLLMVGIGSLIREKRSAAAKKKGASLLCLALASTGLFNPMILAAGLIACDPNRKRGWPATEVMTAVGLMFAIVGGLAELDIDSMAIPMTIAGLMFAAFVISGKSTDMWIERTADISWESDAEITGSSERVDVRLDDDGNFQLMNDPGAPWKIWMLRMVCLAISAYTPWAILPSVVGFWITLQYTKRGGVLWDTPSPKEYKKGDTTTGVYRIMTRGLLGSYQAGAGVMVEGVFHTLWHTTKGAALMSGEGRLDPYWGSVKEDRLCYGGPWKLQHKWNGQDEVQMIVVEPGKNVKNVQTKPGVFKTPEGEIGAVTLDFPTGTSGSPIVDKNGDVIGLYGNGVIMPNGSYISAIVQGERMDEPIPAGFEPEMLRKKQITVLDLHPGAGKTRRILPQIIKEAINRRLRTAVLAPTRVVAAEMAEALRGLPIRYQTSAVPREHNGNEIVDVMCHATLTHRLMSPHRVPNYNLFVMDEAHFTDPASIAARGYISTKVELGEAAAIFMTATPPGTSDPFPESNSPISDLQTEIPDRAWNSGYEWITEYTGKTVWFVPSVKMGNEIALCLQRAGKKVVQLNRKSYETEYPKCKNDDWDFVITTDISEMGANFKASRVIDSRKSVKPTIITEGEGRVILGEPSAVTAASAAQRRGRIGRNPSQVGDEYCYGGHTNEDDSNFAHWTEARIMLDNINMPNGLIAQFYQPEREKVYTMDGEYRLRGEERKNFLELLRTADLPVWLAYKVAAAGVSYHDRRWCFDGPRTNTILEDNNEVEVITKLGERKILRPRWIDARVYSDHQALKAFKDFASGKRSQIGLIEVLGKMPEHFMGKTWEALDTMYVVATAEKGGRAHRMALEELPDALQTIALIALLSVMTMGVFFLLMQRKGIGKIGLGGAVLGVATFFCWMAEVPGTKIAGMLLLSLLLMIVLIPEPERQRSQTDNQLAVFLICVMTLVSAVAANEMGWLDKTKSDISSLFGQRIEVKENFSMGEFLLDLRPATAWSLYAVTTAVLTPLLKHLITSDYINTSLTSINVQASALFTLARGFPFVDVGVSALLLAAGCWGQVTLTVTVTAATLLFCHYAYMVPGWQAEAMRSAQRRTAAGIMKNAVVDGIVATDVPELERTTPIMQKKVGQIMLILVSLAAVVVNPSVKTVREAGILITAAAVTLWENGASSVWNATTAIGLCHIMRGGWLSCLSITWTLIKNMEKPGLKRGGAKGRTLGEVWKERLNQMTKEEFTRYRKEAIIEVDRSAAKHARKEGNVTGGHPVSRGTAKLRWLVERRFLEPVGKVIDLGCGRGGWCYYMATQKRVQEVRGYTKGGPGHEEPQLVQSYGWNIVTMKSGVDVFYRPSECCDTLLCDIGESSSSAEVEEHRTIRVLEMVEDWLHRGPREFCVKVLCPYMPKVIEKMELLQRRYGGGLVRNPLSRNSTHEMYWVSRASGNVVHSVNMTSQVLLGRMEKRTWKGPQYEEDVNLGSGTRAVGKPLLNSDTSKIKNRIERLRREYSSTWHHDENHPYRTWNYHGSYDVKPTGSASSLVNGVVRLLSKPWDTITNVTTMAMTDTTPFGQQRVFKEKVDTKAPEPPEGVKYVLNETTNWLWAFLAREKRPRMCSREEFIRKVNSNAALGAMFEEQNQWRSAREAVEDPKFWEMVDEEREAHLRGECHTCIYNMMGKREKKPGEFGKAKGSRAIWFMWLGARFLEFEALGFLNEDHWLGRKNSGGGVEGLGLQKLGYILREVGTRPGGKIYADDTAGWDTRITRADLENEAKVLELLDGEHRRLARAIIELTYRHKVVKVMRPAADGRTVMDVISREDQRGSGQVVTYALNTFTNLAVQLVRMMEGEGVIGPDDVEKLTKGKGPKVRTWLFENGEERLSRMAVSGDDCVVKPLDDRFATSLHFLNAMSKVRKDIQEWKPSTGWYDWQQVPFCSNHFTELIMKDGRTLVVPCRGQDELVGRARISPGAGWNVRDTACLAKSYAQMWLLLYFHRRDLRLMANAICSAVPVNWVPTGRTTWSIHAGGEWMTTEDMLEVWNRVWIEENEWMEDKTPVEKWSDVPYSGKREDIWCGSLIGTRARATWAENIQVAINQVRAIIGDEKYVDYMSSLKRYEDTTLVEDTVL

>ADK62507.1 polyprotein [West Nile virus]

MSKKPGGPGKSRAVNMLKRGMPRVLSLIGLKRAMLSLIDGKGPIRFVLALLAFFRFTAIAPTRAVLDRWRGVNKQTAMKHLLSFKKELGTLTSAINRRSSKQKKRGGKTGIAVMIGLIASVGAVTLSNFQGKVMMTVNATDVTDVITIPTAAGKNLCIVRAMDVGYMCDDTITYECPVLSAGNDPEDIDCWCTKSAVYVRYGRCTKTRHSRRSRRSLTVQTHGESTLANKKGAWMDSTKATRYLVKTESWILRNPGYALVAAVIGWMLGSNTMQRVVFVVLLLLVAPAYSFNCLGMSNRDFLEGVSGATWVDLVLEGDSCVTIMSRDKPTIDVKMMNMEAANLAEVRSYCYLATVSDLSTKAACPTMGEAHNDKRADPAFVCRQGVVDRGWGNGCGLFGKGSIDTCAKFACSTKAIGRTILKENIKYEVAIFVHGPTTVESHGNYSTQAGATQAGRFSITPAAPSYTLKLGEYGEVTVDCEPRSGIDTNAYYVMTVGTKTFLVHREWFMDLNLPWSSAGSTVWRNRETLMEFEEPHATKQSVIALGSQEGALHQALAGAIPVEFSSNTVKLTSGHLKCRVKMEKLQLKGTTYGVCSKAFKFLGTPADTGHGTVVLELQYTGTDGPCKVPISSVASLNDLTPVGRLVTVNPFVSVATANAKVLIELEPPFGDSYIVVGRGEQQINHHWHKSGSSIGKAFTTTLKGAQRLAALGDTAWDFGSVGGVFTSVGKAVHQVFGGAFRSLFGGMSWITQGLLGALLLWMGINARDRSIALTFLAVGGVLLFLSVNVHADTGCAIDISRQELRCGSGVFIHNDVEAWMDRYKYYPETPQGLAKIIQKAHKEGVCGLRSVSRLEHQMWEAVKDELNTLLKENGVDLSVVVEKQEGMYKSAPKRLTATTEKLEIGWKAWGKSILFAPELANNTFVVDGPETKECPTQNRAWNSLEVEDFGFGLTSTRMFLKVRESNTTECDSKIIGTAVKNNLAIHSDLSYWIESRLNDTWKLERAVLGEVKSCTWPETHTLWGDGILESDLIIPVTLAGPRSNHNRRPGYKTQNQGPWDEGRVEIDFDYCPGTTVTLSESCGHRGPATRTTTESGKLITDWCCRSCTLPPLRYQTDSGCWYGMEIRPQRHDEKTLVQSQVNAYNADMIDPFQLGLLVVFLATQEVLRKRWTAKISMPAILIALLVLVFGGITYTDVLRYVILVGAAFAESNSGGDVVHLALMATFKIQPVFMVASFLKARWTNQENILLMLAAVFFQMAYHDARQILLWEIPDVLNSLAVAWMILRAITFTTTSNVVVPLLALLTPGLRCLNLDVYRILLLMVGIGSLIREKRSAAAKKKGASLLCLALASTGLFNPMILAAGLIACDPNRKRGWPATEVMTAVGLMFAIVGGLAELDIDSMAIPMTIAGLMFAAFVISGKSTDMWIERTADISWESDAEITGSSERVDVRLDDDGNFQLMNDPGAPWKIWMLRMVCLAISAYTPWAILPSVVGFWITLQYTKRGGVLWDTPSPKEYKKGDTTTGVYRIMTRGLLGSYQAGAGVMVEGVFHTLWHTTKGAALMSGEGRLDPYWGSVKEDRLCYGGPWKLQHKWNGQDEVQMIVVEPGKNVKNVQTKPGVFKTPEGEIGAVTLDFPTGTSGSPIVDKNGDVIGLYGNGVIMPNGSYISAIVQGERMDEPIPAGFEPEMLRKKQITVLDLHPGAGKTRRILPQIIKEAINRRLRTAVLAPTRVVAAEMAEALRGLPIRYQTSAVPREHNGNEIVDVMCHATLTHRLMSPHRVPNYNLFVMDEAHFTDPASIAARGYISTKVELGEAAAIFMTATPPGTSDPFPESNSPISDLQTEIPDRAWNSGYEWITEYTGKTVWFVPSVKMGNEIALCLQRAGKKVVQLNRKSYETEYPKCKNDDWDFVITTDISEMGANFKASRVIDSRKSVKPTIITEGEGRVILGEPSAVTAASAAQRRGRIGRNPSQVGDEYCYGGHTNEDDSNFAHWTEARIMLDNINMPNGLIAQFYQPEREKVYTMDGEYRLRGEERKNFLELLRTADLPVWLAYKVAAAGVSYHDRRWCFDGPRTNTILEDNNEVEVITKLGERKILRPRWIDARVYSDHQALKAFKDFASGKRSQIGLIEVLGKMPEHFMGKTWEALDTMYVVATAEKGGRAHRMALEELPDALQTIALIALLSVMTMGVFFLLMQRKGIGKIGLGGAVLGVATFFCWMAEVPGTKIAGMLLLSLLLMIVLIPEPEKQRSQTDNQLAVFLICVMTLVSAVAANEMGWLDKTKSDISSLFGQRIEVKENFSMGEFLLDLRPATAWSLYAVTTAVLTPLLKHLITSDYINTSLTSINVQASALFTLARGFPFVDVGVSALLLAAGCWGQVTLTVTVTAATLLFCHYAYMVPGWQAEAMRSAQRRTAAGIMKNAVVDGIVATDVPELERTTPIMQKKVGQIMLILVSLAAVVVNPSVKTVREAGILITAAAVTLWENGASSVWNATTAIGLCHIMRGGWLSCLSITWTLIKNMEKPGLKRGGAKGRTLGEVWKERLNQMTKEEFTRYRKEAIIEVDRSAAKHARKEGNVTGGHPVSRGTAKLRWLVERRFLEPVGKVIDLGCGRGGWCYYMATQKRVQEVRGYTKGGPGHEEPQLVQSYGWNIVTMKSGVDVFYRPSECCDTLLCDIGESSSSAEVEEHRTIRVLEMVEDWLHRGPREFCVKVLCPYMPKVIEKMELLQRRYGGGLVRNPLSRNSTHEMYWVSRASGNVVHSVNMTSQVLLGRMEKRTWKGPQYEEDVNLGSGTRAVGKPLLNSDTSKIKNRIERLRREYSSTWHHDENHPYRTWNYHGSYDVKPTGSASSLVNGVVRLLSKPWDTITNVTTMAMTDTTPFGQQRVFKEKVDTKAPEPPEGVKYVLNETTNWLWAFLAREKRPRMCSREEFIRKVNSNAALGAMFEEQNQWRSAREAVEDPKFWEMVDEEREAHLRGECHTCIYNMMGKREKKPGEFGKAKGSRAIWFMWLGARFLEFEALGFLNEDHWLGRKNSGGGVEGLGLQKLGYILREVGTRPGGKIYADDTAGWDTRITRADLENEAKVLELLDGEHRRLARAIIELTYRHKVVKVMRPAADGRTVMDVISREDQRGSGQVVTYALNTFTNLAVQLVRMMEGEGVIGPDDVEKLTKGKGPKVRTWLFENGEERLSRMAVSGDDCVVKPLDDRFATSLHFLNAMSKVRKDIQEWKPSTGWYDWQQVPFCSNHFTELIMKDGRTLVVPCRGQDELVGRARISPGAGWNVRDTACLAKSYAQMWLLLYFHRRDLRLMANAICSAVPVNWVPTGRTTWSIHAGGEWMTTEDMLEVWNRVWIEENEWMEDKTPVEKWSDVPYSGKREDIWCGSLIGTRARATWAENIQVAINQVRAIIGDEKYVDYMSSLKRYEDTTLVEDTVL
