# Supplementary material for: Immunoinformatics and molecular dynamics approaches: Next generation vaccine design against West Nile virus
Source: PLoS One. 2021 Jun 17;16(6):e0253393. doi: 10.1371/journal.pone.0253393 (PMC8211291; doi:10.1371/journal.pone.0253393)
Supplement: S5 File — (DOCX) [file pone.0253393.s008.docx]

**Protein Sequences of Vaccine:**

>GIINTLQKYYCRVRGGRCAVLSCLPKEEQIGKCSTRGRKCCRRKKEAAAKAKFVAAWTLKAAAGGGSLAFFRFTAIGGGSVVFVVLLLLGGGSSIALTFLAVGGGSKSILFAPELGGGSFQLGLLVVFGGGSLMFAIVGGLGGGSKTVWFVPSVGGGSMTMGVFFLLGGGSLEFEALGFLGPGPGAFFRFTAIAPTRAVLGPGPGLRNPGYALVAAVIGWGPGPGVTVNPFVSVATANAKGPGPGIQPVFMVASFLKARWGPGPGVCLAISAYTPWAILPGPGPGPVWLAYKVAAAGVSYGPGPGFFLLMQRKGIGKIGLGPGPGLDLRPATAWSLYAVTGPGPGAIWFMWLGARFLEFEKKSKKPGGPGKSRAVNMLKKLKRGMPRVLSLIGLKRKKLSAGNDPEDIDCWCTKKKSLTVQTHGESTLANKKKKCVTIMSKDKPTIDVKMKKYIVVGRGEQQINHHWHEGGETAKSKKFPSYTATYQF

**Optimized Codon of Constructed Vaccine:**

>ATGGGCATCATCAACACCCTGCAGAAGTACTACTGCAGAGTGCGGGGCGGAAGATGCGCC
GTGCTGAGCTGCCTGCCTAAGGAGGAACAGATCGGCAAGTGTAGCACTAGAGGAAGAAAG
TGTTGTCGGAGAAAGAAGGAAGCCGCTGCCAAGGCCAAGTTCGTGGCCGCCTGGACCCTG
AAGGCGGCCGCCGGCGGCGGCTCTCTGGCCTTCTTCAGATTCACCGCCATCGGCGGCGGC
AGCGTGGTGTTCGTGGTGCTGCTGCTGCTGGGCGGCGGGAGCAGCATCGCTCTTACATTT
CTGGCCGTGGGCGGCGGAAGCAAGTCTATCCTGTTCGCCCCTGAGCTGGGCGGAGGTTCT
TTTCAGCTGGGCCTGCTGGTGGTGTTCGGCGGAGGTAGCCTGATGTTCGCCATTGTGGGA
GGCCTGGGCGGAGGCAGCAAGACAGTGTGGTTTGTTCCTAGCGTGGGCGGAGGCAGCATG
ACCATGGGCGTCTTTTTCCTGCTGGGCGGCGGCAGCCTGGAATTCGAGGCCCTGGGATTT
CTGGGCCCCGGGCCAGGCGCCTTTTTCCGGTTCACCGCTATCGCCCCTACCCGCGCCGTG
CTGGGACCCGGACCTGGCCTGAGAAACCCCGGATACGCCCTGGTGGCCGCTGTGATCGGC
TGGGGCCCTGGCCCAGGAGTCACCGTGAATCCTTTCGTGTCCGTGGCTACAGCCAACGCC
AAAGGCCCAGGCCCTGGCATTCAGCCTGTGTTTATGGTGGCCAGCTTCCTGAAGGCCAGA
TGGGGACCTGGCCCCGGCGTGTGCCTGGCCATCAGCGCCTACACCCCTTGGGCCATCCTG
CCTGGCCCTGGCCCAGGCCCCGTGTGGCTGGCCTACAAGGTGGCCGCTGCCGGCGTGTCT
TACGGACCCGGCCCTGGCTTCTTCCTCCTGATGCAACGGAAGGGCATCGGCAAGATCGGG
CTGGGCCCCGGTCCTGGCCTTGATCTGCGGCCTGCCACAGCCTGGTCCCTGTACGCCGTG
ACAGGCCCTGGCCCTGGCGCTATCTGGTTCATGTGGCTGGGAGCTAGGTTCCTGGAATTC
GAGAAGAAGAGCAAGAAGCCCGGCGGCCCAGGCAAATCAAGAGCCGTTAACATGCTGAAG
AAGTTGAAGCGGGGCATGCCTAGAGTGCTGAGTCTGATCGGCCTGAAAAGAAAAAAACTG
AGCGCCGGAAATGACCCCGAGGACATCGATTGCTGGTGCACCAAGAAAAAAAGCCTGACC
GTGCAGACCCACGGCGAATCTACCCTGGCCAACAAGAAAAAGAAGTGCGTGACCATCATG
AGCAAGGACAAGCCTACAATCGACGTGAAGATGAAGAAATACATCGTGGTCGGCAGAGGC
GAGCAGCAGATCAACCACCATTGGCACGAGGGCGGCGAGACAGCTAAGTCCAAAAAGTTC
CCCAGCTATACAGCCACCTACCAGTTC
